# Synthesis of multiply fluorinated *N*-acetyl-D-glucosamine and D-galactosamine analogs via the corresponding deoxyfluorinated glucosazide and galactosazide phenyl thioglycosides

**DOI:** 10.3762/bjoc.17.85

**Published:** 2021-05-11

**Authors:** Vojtěch Hamala, Lucie Červenková Šťastná, Martin Kurfiřt, Petra Cuřínová, Martin Dračínský, Jindřich Karban

**Affiliations:** 1 Department of Bioorganic Compounds and Nanocomposites, Institute of Chemical Process Fundamentals of the CAS, v. v. i., Rozvojová 135, 16502 Praha 6, Czech Republic; 2 University of Chemistry and Technology Prague, Technická 5, 16628 Praha 6, Czech Republic; 3 NMR Spectroscopy group, Institute of Organic Chemistry and Biochemistry of the CAS, Flemingovo náměstí 542/2, 16000 Praha, Czech Republic

**Keywords:** amino sugars, deoxyfluorination, fluorinated carbohydrates, hexosamine hemiacetals, thioglycosides

## Abstract

Multiple fluorination of glycostructures has emerged as an attractive way of modulating their protein affinity, metabolic stability, and lipophilicity. Here we described the synthesis of a series of mono-, di- and trifluorinated *N*-acetyl-ᴅ-glucosamine and ᴅ-galactosamine analogs. The key intermediates are the corresponding multiply fluorinated glucosazide and galactosazide thioglycosides prepared from deoxyfluorinated 1,6-anhydro-2-azido-β-ᴅ-hexopyranose precursors by ring-opening reaction with phenyl trimethylsilyl sulfide. Nucleophilic deoxyfluorination at C4 and C6 by reaction with DAST, thioglycoside hydrolysis and azide/acetamide transformation completed the synthesis.

## Introduction

Fluorinated carbohydrates are versatile carbohydrate mimetics used to probe or manipulate the recognition of carbohydrates by carbohydrate-binding proteins or carbohydrate-processing enzymes [1–7]. The introduction of additional fluorine atoms into a monofluorinated carbohydrate is an attractive way of modulating the binding affinity and pharmacokinetic properties of fluorinated glycomimetics. Hydrophobic segments incorporating multiple C–F bonds could (1) reduce the desolvation penalty associated with binding of hydrophilic natural carbohydrates [8], and (2) create additional contacts with the binding cavity via electrostatic and dipolar interactions with C–F bonds [9–10], new intermolecular hydrogen bonds [11], or rearrangement of hydrogen bond-mediating water molecules [12]. The fluorination of sugars is also a promising strategy to improve unfavorable pharmacokinetic properties of natural carbohydrates such as low lipophilicity [13–16] and fast metabolic degradation [17–19]. Over the last few years, considerable effort has been expended on the synthesis of unprotected multiply-deoxyfluorinated monosaccharides, including a complete series of mono-, di-, and trifluorinated ᴅ-glucose [15], difluorinated [20] and tetrafluorinated [13] ᴅ-galactose, and 2,3,4-trifluoro analogs of ᴅ-mannose, ᴅ-galactose, ᴅ-allose, ᴅ-talose, and ᴅ-altrose [13,21].

Unprotected multiply-deoxyfluorinated *N*-acetyl-ᴅ-glucosamine (GlcNAc) and *N*-acetyl-ᴅ-galactosamine (GalNAc) have not yet been described except for a 4,6-difluoro-GalNAc analog [22], although GlcNAc is the most abundant monosaccharide component of mammalian glycans [23], and GalNAc occurs in important glycan structures including the T_N_ and T antigen and their sialylated forms [24]. A complete series of O-protected monofluorinated [22,25–32] and several difluorinated [22,26,33–34] GlcNAc/GalNAc analogs have been prepared. Some acylated mono- and difluorinated analogs have potential in biomedical applications due to their ability to inhibit the glycan and glycosaminoglycan biosynthesis [34–37]. The fluorine substituent has typically been introduced into these GlcNAc and GalNAc analogues using nucleophilic fluorination. The primary position (C6 hydroxy group) was fluorinated by reaction with diethylamino sulfurtrifluoride (DAST) [27]. This reaction was greatly improved by microwave irradiation, especially in the GalNAc series [31–32]. The deoxyfluorination of the secondary hydroxy groups at the 3- and 4-positions was accomplished using a treatment of the C3/4 hydroxy groups with DAST [22,25–26,30,35], or reaction of C3/C4 methanesulfonate or trifluoromethanesulfonate esters with a source of nucleophilic fluorine, such as TBAF or KF [22,25,34]. Although these fluorinations usually proceeded with inversion of configuration, the acetylated 3-fluoro-GlcNAc analogue was most conveniently accessed using retentive DAST fluorination of 2-azido-4-*O*-benzyl-2-deoxy-1,6-anhydro-β-ᴅ-glucopyranose [26]. Preparation of glycostructures comprising multifluorinated GlcNAc and GalNAc will be greatly facilitated if synthetic routes to the corresponding glycosyl donors are developed. Here we describe the synthesis of a complete series of unprotected GlcNAc and GalNAc analogs systematically deoxyfluorinated at all non-anomeric hydroxy positions. The key synthetic intermediates are multifluorinated glucosazide and galactosazide thioglycosides and hemiacetals, which are also valuable glycosyl donors for the installation of a 1,2-*cis*-linked multifluorinated GlcNAc and GalNAc moiety.

## Results and Discussion

**Scheme 1:**
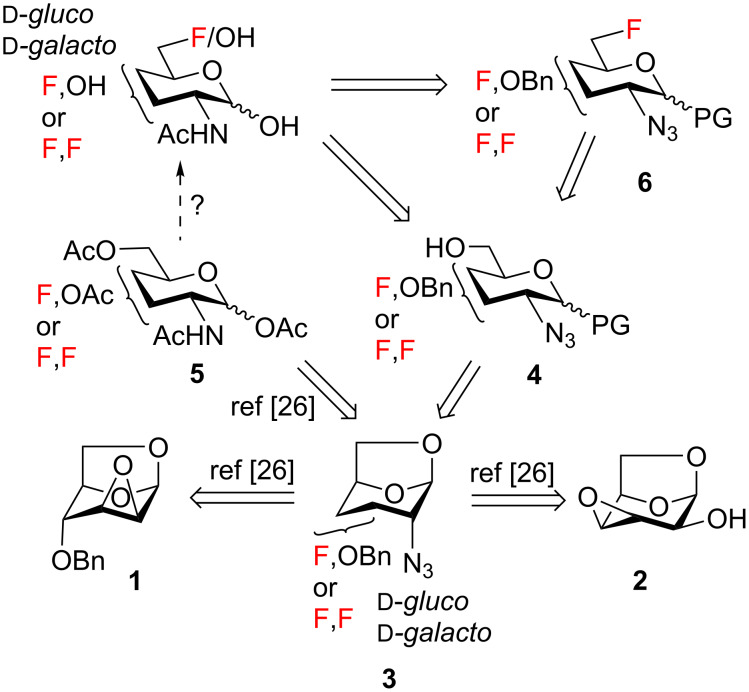
Retrosynthetic analysis of the target fluoro analogs.

Our approach to the synthesis is summarized in Scheme 1. Challenging regio- and stereoselective introduction of fluorine at C3 and C4 of the pyranose ring, together with azide installation at C2, can be accomplished by nucleophilic fluorination and azidolysis starting from dianhydro derivatives **1** and **2** as we described previously [26]. The resulting intermediates **3** can be transformed into 2-azidohexopyranosides **4** by cleavage of the internal acetal and protection of the anomeric position. Deoxyfluorination at C6 should then afford intermediates **6**. Protecting-group manipulation of intermediates **4** and **6** should deliver the required fluoro analogs. The initially contemplated conversion of intermediates **3** into acetates **5** [26], followed by base-catalyzed O-deacetylation, led to substantial decomposition. These observations are consistent with the recently reported instability of O-acylated GlcNAc under basic conditions due to elimination reactions of transient hemiacetal intermediates [38]. This instability of amino sugar hemiacetals underscores the requirement to both protect the anomeric position with a robust protecting group and to conduct final deprotection under neutral conditions. After initial experimentation with benzyl glycosides (Scheme 1, PG = OBn), phenyl thioglycosides (Scheme 1, PG = SPh), readily available from 1,6-anhydropyranoses [39] as we described earlier [40] were found to fulfill this requirement satisfactorily.

**Scheme 2:**
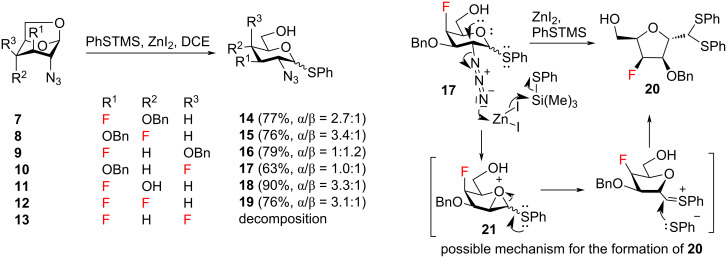
Conversion of 1,6-anhydro derivatives into thioglycosides, and a possible mechanism for the formation of *C*-furanosides by ring contraction.

Accordingly, the synthesis started from known fluorinated 1,6-anhydro-2-azidohexopyranoses **7**–**13** (Scheme 2) [26,40]. Reaction of compounds **7**–**10** with phenyl trimethylsilyl sulfide (PhSTMS) and ZnI_2_ delivered phenyl thioglycosides **14–17** [40]. 1,6-Anhydropyranoses **11** and **12** under these conditions produced the expected thioglycosides **18** and **19**, respectively. Difluorinated derivative **13** decomposed on reaction with the PhSTMS/ZnI_2_ system. The separation of the anomers of products **14**–**19** was attempted because of the risk of thiophenyl migration in the subsequent C6 deoxyfluorination, which would likely occur with the β-anomers of **14**–**19** [41]. The complete separation of the α-anomer by conventional silica gel column chromatography was possible for thioglycosides **14**, **16**, **17**, and **19**, while the products **15** and **18** were obtainable as enriched α-anomers (α/β ≥ 3.3:1). Cleavage of the internal acetal with PhSTMS was accompanied by the formation of low quantities of side-products detectable by TLC and separable by careful chromatography except for the cleavage of **12** where the side products co-eluted with the fraction containing the β-anomer of the product. In the case of the cleavage of 1,6-anhydro derivative **10**, we were able to isolate one of the side-products in sufficient purity and quantity to assign the structure of *C*-furanoside **20** (Scheme 2). This compound resulted from pyranose ring contraction probably caused by intramolecular displacement of the C2 azide aided by coordination of ZnI_2_. When the α-anomer of thioglycoside **17** was separately subjected to the reaction conditions, the byproduct **20** started to form in trace amounts in accordance with the suggested mechanism. The ring contraction may involve formation of a transient oxiranium cation as suggested in Scheme 2 [42–45]. Analogous ring-contraction reactions have been described for substrates possessing a good C2 leaving group [42,46–50].

**Scheme 3:**
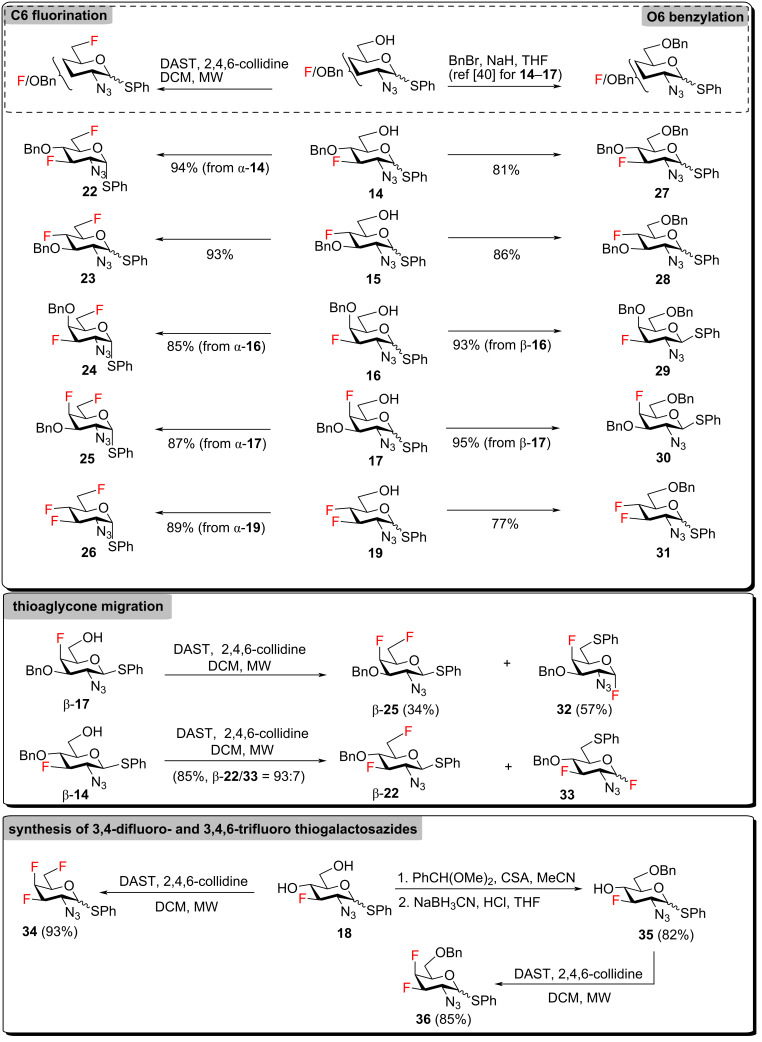
Deoxyfluorination and O-benzylation of thioglycosides and thioaglycone migration.

We initially considered converting thioglycosides **14**–**19** to benzyl glycosides because thioglycosides give glycosyl fluorides on reaction with diethylamino sulfurtrifluoride (DAST) [51], but our experiments revealed that DAST-mediated C6-deoxyfluorination of thioglycosides **14**–**17** and **19** proceeded satisfactorily under microwave irradiation, on condition that pure or substantially enriched α-anomers were subjected to reaction with DAST, yielding thioglycosides **22**–**26** (Scheme 3). Reaction of β-thiogalactosides possessing an unprotected C6 hydroxy group with DAST was accompanied by migration of the anomeric thio-aglycone to C6 [41,52] as shown for β-thiogalactoside β-**17**, which mostly delivered migration product **32** (Scheme 3, see also the synthesis of compound **24** in the Supporting Information File 1). However, β-thioglucoside β-**14** yielded only 6% of migration product **33** together with the main C6-fluoro product β-**22**, suggesting that starting fluorinated 2-azido-thioglucosides were significantly less prone to thiophenyl migration than 2-azidothiogalactosides were. This was convenient because thioglucosides **15** and **18** (vide infra) were available for deoxyfluorination only as enriched anomeric mixtures α/β ≥ 3.3:1 and any traces of the migration products were removed in the subsequent thioaglycone hydrolysis. Thioglycosides **14**–**17** and **19** were also O6-benzylated [40] to thioglycosides **27**–**31** (Scheme 3).

**Scheme 4:**
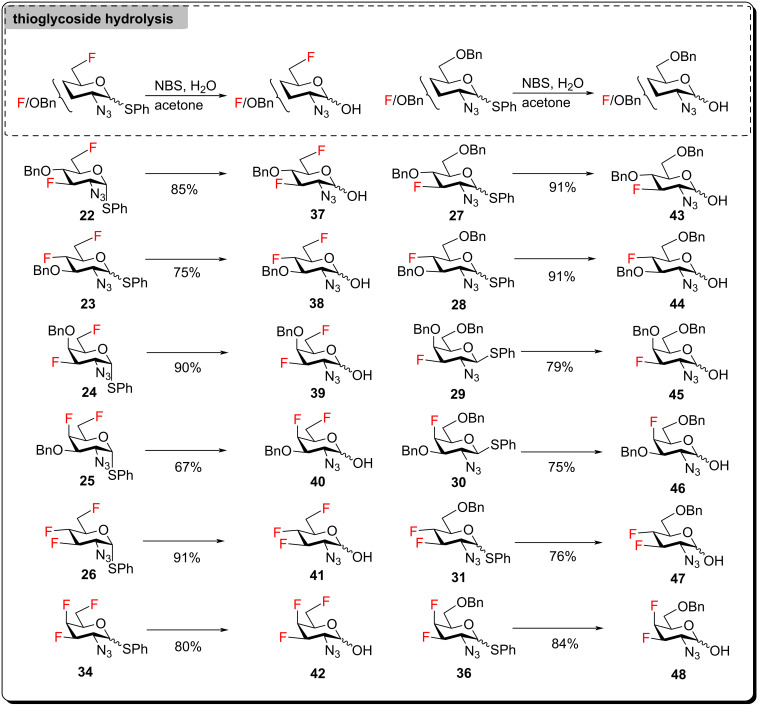
Thioglycoside hydrolysis.

As the 3,4-difluorinated thiogalactoside could not be accessed from compound **13** by reaction with PhSTMS/ZnI_2_ (Scheme 2), it was necessary to obtain 3,4-difluoro and 3,4,6-trifluoro analogs of GalNAc from 3-fluoro-4,6-diol **18**. According to precedents in the literature [53], deoxyfluorination of the C4-hydroxy group in compound **18** was expected to occur with inversion of configuration to give the desired *galacto*-configured 4-fluoro products. Accordingly, treatment of diol **18** with DAST resulted in deoxyfluorination of both hydroxy groups to yield trifluoro thiogalactosazide **34** (Scheme 3). 4,6-O-Benzylidenation of diol **18** followed by regioselective opening of the benzylidene acetal produced compound **35**. Subsequent DAST deoxyfluorination delivered the desired thioglycoside **36** (Scheme 3). For both compounds **18** and **35**, deoxyfluorination of the C4 hydroxy group occurred with inversion of configuration. Thioglycosides **22**–**31**, **34** and **36** were then hydrolyzed into the corresponding hemiacetals **37**–**48** using treatment with NBS in acetone/water (Scheme 4).

**Scheme 5:**
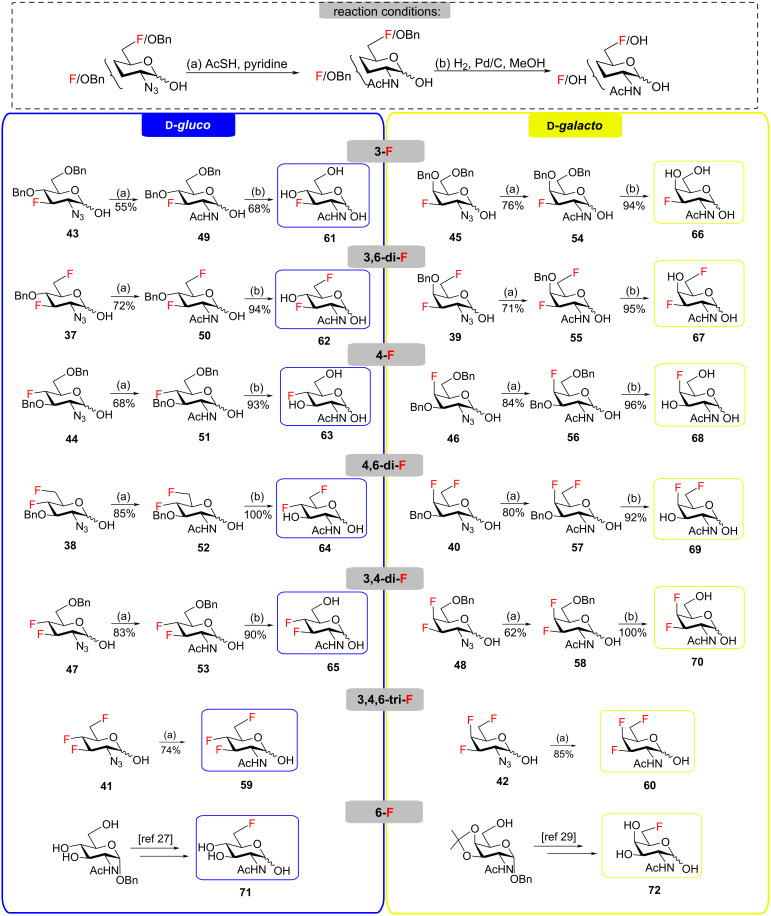
Synthesis of the target compounds by azide/acetamide conversion and debenzylation.

To obtain the target fluoro analogs, the hemiacetals **37**–**48** were debenzylated and their azide group converted to an acetamide. Although palladium-catalyzed hydrogenolysis in ethanol/acetic anhydride appeared to be a logical deprotection step [26], the desired fluoro sugars were contaminated with varying quantities of unidentified byproducts. However, clean debenzylation was achieved by first converting the azide to an acetamide on reaction with thioacetic acid [54–55]. Hence, the hemiacetals were reacted with thioacetic acid in pyridine to give acetamides **49**–**58** (Scheme 5) and the target trifluoro analogs **59** and **60**. Reversing the order of hemiacetal and acetamide formation was not an option because NBS-promoted hydrolysis of 2-acetamido thioglycosides was sluggish and incomplete. Protecting the primary hydroxy group at C6 by O-benzylation (Scheme 3, compounds **27**–**31**) was essential before treatment with thioacetic acid; otherwise, an O6-acetylated byproduct was formed. Acetylation of the anomeric hydroxy group occurred only to a very limited degree upon reaction with AcSH in pyridine and traces of O1 acetates were removed by chromatography or recrystallization. Palladium-catalyzed hydrogenolytic debenzylation of **49**–**58** then yielded the target fluoro analogs **61**–**70**. To complete the series of fluorinated analogs for the purpose of comparing their NMR spectra, the known C6 monodeoxyfluorinated compounds **71** [27–28] and **72** [29] were prepared by published procedures [27,29].

The magnitudes of the vicinal ^3^*J*_H-H_, ^3^*J*_H-F_, ^3^*J*_C-F_, geminal ^2^*J*_H-F_, ^2^*J*_C-F_, and one-bond ^1^*J*_C-F_ coupling constants confirmed the expected fluorination pattern and ᴅ-*gluco* or ᴅ-*galacto* configuration for all fluoro analogs **59**–**72**. The values of the coupling constants correlated with the ^4^*C*_1_ conformation adopted by the target fluoro analogs in solution (Table 1 and Table S1 in Supporting Information File 1). For example, the magnitude of the germinal fluorine–carbon coupling ^2^*J*_C5-F4_ indicated an antiperiplanar (^2^*J*_C5-F4_ = 23.2–24.2 Hz, ᴅ-*gluco* configuration, F4 equatorial) or a gauche (^2^*J*_C5-F4_ = 17.5–18.1 Hz, ᴅ-*galacto* configuration, F4 axial) relationship between the C4‒F and C5‒O bonds [56]. Similarly, the values of ^3^*J*_H3/H5-F4_ coupling constants reflected an axial (^3^*J*_H3/H5-F_ = 25.5–30.3 Hz) or equatorial (^3^*J*_H3-F4_ = 14.8–16.8 Hz, ^3^*J*_H5-F4_ = 2.5–4.8 Hz) position of the C4 fluorine substituent [57]. Moreover, evaluation of ^3^*J*_H5-F6_ coupling constants revealed that 6-fluoro ᴅ-*gluco* analogs **59**, **62**, **64**, and **71** assumed preferentially *gauche,gauche* (*gg*) conformation of the exocyclic C5‒C6 bond in solution (^3^*J*_H5-F6_ = 24.6–27.1 Hz), whereas the corresponding ᴅ-*galacto*-configured analogs **60**, **67**, **69**, and **72** adopted *gauche,trans* (*gt*) or *trans,gauche* (*tg*) conformations to a significant degree (^3^*J*_H5-F6_ = 12.7–14.6 Hz). These findings were in accordance with the previous reports by Giguѐre [13,15,58].

**Table 1:** The values [Hz] of selected coupling constants. Boldfaced values illustrate the trends discussed in the text.

compound	^2^*J*_C3-F4_	^1^*J*_C4-F4_	^2^*J*_C5-F4_			^3^*J*_H3-F4_	^2^*J*_H4-F4_	^3^*J*_H5-F4_

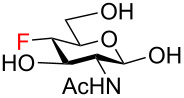 β-**63**	18.6	180.9	**24.2**			**15.6**	50.8	**2.5**
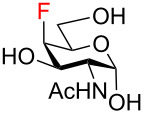 α-**68**	19.1	180.1	**18.1**			**28.9**	50.5	**30.3**

	^4^*J*_C3-F6_	^3^*J*_C4-F6_	^2^*J*_C5-F6_	^1^*J*_C6-F6_		^3^*J*_H5-F6_	^2^*J*_H6-F6_	^2^*J*_H6'-F6_

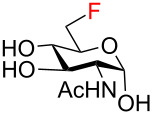 α-**71**	0.7	7	17.9	171.3		**27.1**	48.2	48.2
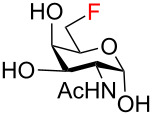 α-**72**	1.1	2.5	13.0	166.8		**14.6**	46.4	48.1

	^3^*J*_C2-F4_	^2^*J*_C3-F4_	^1^*J*_C4-F4_	^2^*J*_C5-F4_		^3^*J*_C4-F6_	^2^*J*_C5-F6_	^1^*J*_C6-F6_

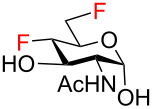 α-**64**	8.0	18.5	181.2	**23.7**		7.4	18.2	172.5
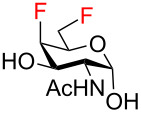 α-**69**	2.7	18.8	180.1	**17.7**		5.9	23.1	168.1

	^5^*J*_H1-F4_	^3^*J*_H3-F4_	^2^*J*_H4-F4_	^3^*J*_H5-F4_		^3^*J*_H5-F6_	^2^*J*_H6-F6_	^2^*J*_H6'-F6_

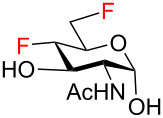 α-**64**	3.3	**14.8**	50.6	**4.1**		**26.6**	48.1	47.5
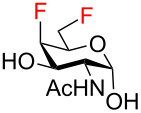 α-**69**	n/a	**28.9**	51.0	**30.3**		**12.7**	47.3	46.2

## Conclusion

In summary, we have demonstrated that multiply deoxyfluorinated GlcNAc and GalNAc are accessible via the corresponding multifluorinated 1-thiophenyl gluco- and galactosazides. Installation of the thiophenyl aglycone permits C6 deoxyfluorination and circumvents the problems resulting from the low stability of amino sugar hemiacetals. The prepared polyfluorinated thiodonors and hemiacetals are valuable intermediates in oligosaccharide synthesis and their utility in glycosylation is currently being studied in our group.

## Supporting Information

File 1 Experimental procedures and spectral data.

File 2 Copies of ^1^H, ^13^C, ^19^F, and 2D NMR spectra for new compounds.
