# Supplementary material for: Synthesis of multiply fluorinated N-acetyl-D-glucosamine and D-galactosamine analogs via the corresponding deoxyfluorinated glucosazide and galactosazide phenyl thioglycosides
Source: Beilstein J Org Chem. 2021 May 11;17:1086–95. doi: 10.3762/bjoc.17.85 (PMC8144920; doi:10.3762/bjoc.17.85)
Supplement: File 1 — Experimental procedures and spectral data. [file Beilstein_J_Org_Chem-17-1086-s001.pdf]

BEILSTEIN JOURNAL OF ORGANIC CHEMISTRY

Supporting Information

for

Synthesis of multiply fluorinated *N*-acetyl-D-glucosamine and D-galactosamine analogs via the corresponding deoxyfluorinated glucosazide and galactosazide phenyl thioglycosides

Vojtěch Hamala, Lucie Červenková Šťastná, Martin Kurfiřt, Petra Cuřínová,
Martin Dračínský and Jindřich Karban

Beilstein J. Org. Chem. **2021,** *17,* 1086–1095. doi:10.3762/bjoc.17.85

Experimental procedures and spectral data

License and Terms: This is a supporting information file under the terms of the Creative Commons Attribution License (https://creativecommons.org/licenses/by/4.0). Please note that the reuse, redistribution and reproduction in particular requires that the author(s) and source are credited and that individual graphics may be subject to special legal provisions.
The license is subject to the *Beilstein Journal of Organic Chemistry* terms and conditions: (https://www.beilstein-journals.org/bjoc/terms)

General methods

Chemicals were used as received. DAST was obtained from Acros Organics. Dichloromethane was dried by distillation from CaH_2 and stored over molecular sieves 3 Å, pyridine was dried by standing over NaOH. Ethyl acetate and petroleum ether (fraction with boiling point 40–65 °C) were distilled before use. TLC was carried out with Sigma-Aldrich TLC Silica gel 60 F254 and spots were detected with an anisaldehyde solution in EtOH/AcOH/H_2SO_4. UV detection at 254 nm was also used where appropriate. Column chromatography was performed with silica gel 60 (70–230 mesh, Material Harvest). Preparative TLC chromatography was performed using 20 cm × 20 cm glass plates covered with TLC-Silica gel 60 GF_{254} (20 g, mean particle size 15 µm, containing 12–13.5% $CaSO_4·0.5\ H_2O$ and fluorescent indicator, Merck). The maximum loading used was approximately 70 mg per one plate. If necessary, the plates were developed repeatedly. The solutions were concentrated at temperatures below 45 °C. Anhydrous sodium sulfate was used to dry solutions after aqueous workup. Reactions requiring microwave irradiation were conducted using Anton Paar Monowave 300 microwave reactor equipped with simultaneous temperature measurement with IR and a fiber optic sensor. The ^{1}H (400.1 MHz), ^{13}C (100.6 MHz), and ^{19}F (376.4 MHz) NMR spectra were measured on a Bruker Avance 400 spectrometer at 25 °C. The ^{1}H and ^{13}C NMR spectra were referenced to the line of the solvent (δ/ppm; δ_H/δ_C: $CDCl_3$, 7.26/77.16, MeOH-d_4, 3.31/49.00). The ^{19}F spectra were referenced to the line of internal standard hexafluorobenzene (δ/ppm; −163.00 in $CDCl_3$, −166.62 in MeOH-d_4). Structural assignments were made with additional information from COSY, HSQC, and HMBC experiments. HRMS analyses were done using Bruker MicrOTOF-QIII, using APCI ionization in positive mode and a TOF mass analyzer, the m/z value of the $[M – N_2 + H]^+$ adduct is reported for 2-azido sugars because the molecular ion adducts were undetectable or extremely weak in abundance. Starting compounds **7**–**13**, and **14–17** were prepared following the published procedures [1,2]. EtOAc stands for ethyl acetate, PE for petroleum ether, MTBE for methyl *tert*-butyl ether.

General procedure for reactions of 1,6-anhydropyranoses with phenyl trimethylsilyl sulfide

To a solution of the starting deoxyfluorinated 1,6-anhydrohexopyranose in dry 1,2-dichloroethane ($c \approx 0.2–0.3$ mol dm^{-3}) phenyl trimethylsilyl sulfide (PhSTMS, 3.3 equiv) and ZnI_2 (1.5 equiv) were added sequentially under argon atmosphere and the reaction was stirred vigorously with the exclusion of light and moisture at rt for about 24–120 h, until TLC indicated full consumption of the starting compound. Spots of C6-OH products in varying intensity can also be detected near the TLC origin. The reaction was diluted with dichloromethane, filtered, washed with water and the water phase was extracted with dichloromethane (3×). The organic extracts were combined, dried and concentrated. The crude product was then dissolved in methanol ($c \approx 0.1$ mmol dm^{-3}) acidified by a few drops of AcOH and stirred at rt for about 1–2 h to remove 6-*O*-trimethylsilyl group (indicated by TLC), concentrated and purified by column chromatography.

General procedure for C6 or C4 deoxyfluorination

Diethylaminosulfur trifluoride (1.3 equiv per reacting OH group) and 2,4,6-collidine (2.6 equiv per reacting OH group) were added dropwise to a solution of the starting alcohol in dichloromethane (*c* 0.1–0.2 mol dm^{-3}) and the reaction was stirred, and heated to 80 °C under microwave irradiation, and kept at this temperature for 1 h in a 30 mL sealed glass vial. The reaction mixture was quenched by the addition of MeOH, diluted with dichloromethane and washed with a 1% aqueous solution of HCl. The water phase was extracted with dichloromethane (3×). Organic extracts were combined, dried, and concentrated. The crude product was purified by column chromatography.

General procedure for thioglycoside hydrolysis

A solution of the starting phenyl thioglycoside and *N*-bromosuccinimide (NBS, 4 equiv) in acetone/water 9/1 (*c* 0.05–0.1 mol dm^{-3}) was stirred at rt for about 1 h. The reaction mixture turned red and then gradually became colorless. When TLC indicated full consumption of the starting compound, the reaction was quenched by the addition of an aqueous solution of $Na_2S_2O_3$, diluted with dichloromethane and washed with water. The water phase was extracted with dichloromethane (3×). Organic extracts were combined, dried, and concentrated. The crude product was purified by column chromatography.

General procedure for azide/acetamide conversion

2-Azidohexose was dissolved in pyridine (0.1 mL per 1 mmol of the 2-azidohexose) and thioacetic acid (0.1 mL per 1 mmol of the 2-azidohexose) and the resulting solution was stirred overnight. The reaction mixture may turned into a thick paste during this period. It was concentrated, co-distilled with toluene and dry-loaded onto a chromatographic column for purification.

General procedure for debenzylation

An *O*-benzylated hexose was dissolved in a given volume of methanol and 10% palladium on carbon was added under argon atmosphere. The reaction mixture was degassed and purged with hydrogen (three cycles). Reaction was stirred under H_2 atmosphere until TLC indicated the complete absence of the starting material and the presence of a more polar product (usually for 24–72 h). The reaction mixture was filtered, concentrated and recrystallized.

1,6-Anhydro-2-azido-4-*O*-benzyl-2,3-dideoxy-3-fluoro-β-D-glucopyranose (7)

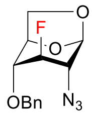

7

Compound **7** was prepared as described in ref 1.

1,6-Anhydro-2-azido-3-*O*-benzyl-2,4-dideoxy-4-fluoro-β-D-glucopyranose (8)

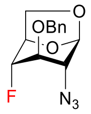

8

Compound **8** was prepared as described in ref 2.

1,6-Anhydro-2-azido-4-*O*-benzyl-2,3-dideoxy-3-fluoro-β-D-galactopyranose (9)

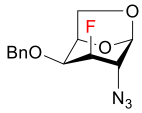

9

Compound **9** was prepared as described in ref 2.

1,6-Anhydro-2-azido-2,4-dideoxy-4-fluoro-β-D-galactopyranose (10)

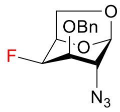

10

Galactopyranose **10** was prepared according to ref 2.

1,6-Anhydro-2-azido-2,3-dideoxy-3-fluoro-β-D-glucopyranose (11)

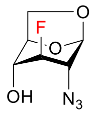

11

Glucopyranose **11** was prepared according to ref 1.

1,6-Anhydro-2-azido-2,3,4-trideoxy-3,4-difluoro-β-D-glucopyranose (12)

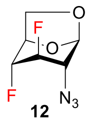

12

Glucopyranose **12** was prepared according to ref 1.

Phenyl 2-Azido-2,3-dideoxy-3-fluoro-4-*O*-benzyl-1-thio-α/β-D-glucopyranoside (14)

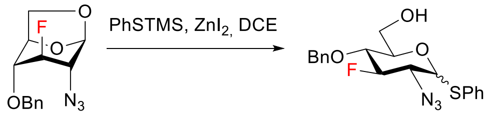

7 **14**

Thioglycoside **14** was prepared from **7** in 77% yield according to ref 2.

Phenyl 2-Azido-2,4-dideoxy-4-fluoro-3-*O*-benzyl-1-thio-α/β-D-glucopyranoside (15)

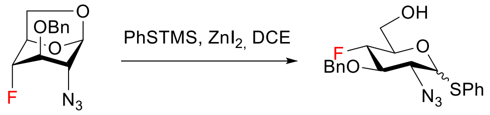

8 **15**

Thioglycoside **15** was prepared from **8** in 76% yield according to ref 2.

Phenyl 2-Azido-2,3-dideoxy-3-fluoro-4-*O*-benzyl-1-thio-α/β-D-galactopyranoside (16)

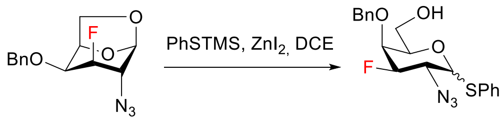

9 **16**

Thioglycoside **16** was prepared from **9** in 79% yield according to ref 2.

Phenyl 2-Azido-2,4-dideoxy-4-fluoro-3-*O*-benzyl-1-thio-α/β-D-galactopyranoside (17)

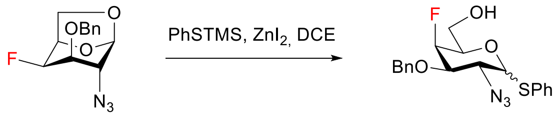

10 **17**

Thioglycoside **17** was prepared from **10** in 63% yield according to ref 2. See also the synthesis of compound **20**.

Phenyl 2-Azido-2,3-dideoxy-3-fluoro-1-thio-α/β-D-glucopyranoside (18)

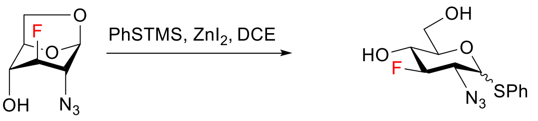

11 **18**

Thioglycoside **18** was prepared by the reaction of **11** (1.00 g, 5.29 mmol) with PhSTMS (3.00 mL, 15.84 mmol) and ZnI_2 (2.50 g, 7.83 mmol) in 1,2-dichloroethane (20 mL) according to the general procedure. The reaction was completed in 48 h when TLC (EtOAc/PE 1:2) showed the absence of compound **11** (R_f 0.27) and the presence of one major product (R_f 0.80). Chromatography of the residue after work-up (see the general procedure) in EtOAc/PE 2:1 afforded **18** as a colorless gel-like mixture of anomers (1.42 g, 90%), which crystallized after standing in a fridge as a white crystalline solid, mp 82–84 °C (dec, from heptane/MTBE, α/β 3.3:1). R_f 0.38 (EtOAc/PE 1:1). NMR data for the α-anomer: ^{1}H NMR ($CDCl_3$, 400 MHz, ^{1}H {^{19}F}, H-H COSY): δ 7.49–7.47 (m, 2H, CH_{arom}), 7.36–7.32 (m, 3H, CH_{arom}), 5.57 (dd, 1H, J = 5.7, 3.2 Hz, H-1), 4.70 (ddd, 1H, J = 52.9, 10.1, 8.6 Hz, H-3), 4.22 (dt, 1H, J = 10.0, 3.4 Hz, H-5), 3.99 (ddd, 1H, J = 11.0, 10.1, 5.7 Hz, H-2), 3.95–3.74 (m, 3H, H-4, 2H-6), 3.23, 2.09 (2 × br s, 2 × 1H, OH). ^{13}C{^{1}H} NMR ($CDCl_3$, 101 MHz, HSQC): δ 132.7 (2CH_{arom}), 132.6 (C_q), 129.4 (2CH_{arom}), 128.4 (CH_{arom}), 94.5 (d, ^1J = 184.5 Hz, C-3), 86.9 (d, ^3J = 8.0 Hz, C-1), 71.9 (d, ^3J = 6.8 Hz, C-5), 69.1 (d, ^2J = 18.0 Hz, C-2), 62.1 (d, ^2J = 17.0 Hz, C-4), 61.5 (d, ^4J = 1.3 Hz, C-6). ^{19}F NMR ($CDCl_3$, 376 MHz): –194.44 (dddd, ^2J = 53.0 Hz, ^3J = 13.7, 11.0 Hz, ^4J = 3.2 Hz). NMR data for the β-anomer: ^{1}H NMR ($CDCl_3$, 400 MHz, ^{1}H {^{19}F}, H-H COSY): δ 7.55–7.53 (m, 2H, CH_{arom}), 7.36–7.32 (m, 3H, CH_{arom}), 4.47 (dd, 1H, J = 10.2, 0.9 Hz, H-1), 4.36 (ddd, 1H, J = 51.7, 9.1 8.9 Hz, H-3), 3.95–3.74 (m, 3H, H-4, 2H-6), 3.43 (ddd, 1H, J = 12.3, 10.2, 9.1 Hz, H-2), 3.35 (dddd, 1H, J = 9.8, 4.4, 3.1, 1.2 Hz, H-5), 3.18, 1.81 (2 × br s, 2 × 1H, OH). ^{13}C{^{1}H} NMR ($CDCl_3$, 101 MHz, HSQC): δ 133.5 (2CH_{arom}), 130.9 (C_q), 129.4 (2CH_{arom}), 128.9 (CH_{arom}), 96.8 (d, ^1J = 187.1 Hz, C-3), 85.9 (d, ^3J = 7.1 Hz, C-1), 78.6 (d, ^3J = 7.2 Hz, C-5), 68.5 (d, ^2J = 18.1 Hz, C-4), 63.3 (d, ^2J = 17.4 Hz, C-2), 61.8 (d, ^4J = 1.7 Hz, C-6). ^{19}F NMR ($CDCl_3$, 376 MHz): –189.40 (ddd, ^2J = 51.7 Hz, ^3J = 13.5, 12.3 Hz). HRMS-APCI (m/z): $[M - N_2 + H]^+$ calcd for $C_{12}H_{15}FNO_3S$, 272.0751; found, 272.0750.

Phenyl 2-Azido-2,3,4-trideoxy-3,4-difluoro-1-thio-α-D-glucopyranoside (α-19)

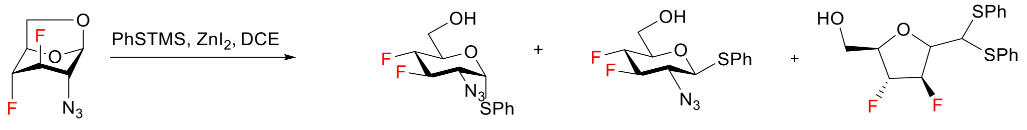

12 α-**19** β-**19** **S1**

Thioglycoside α-**19** was prepared by reaction of **12** (670 mg, 3.51 mmol) with PhSTMS (1.65 mL, 8.71 mmol) and ZnI_2 (1.50 g, 4.70 mmol) in 1,2-dichloroethane (12 mL) according to the general procedure. The reaction was completed in 48 h when TLC (EtOAc/PE 1:4) showed the absence of **12** (R_f 0.29) and the presence of one major product (R_f 0.65). Chromatography of the residue after workup (see the general procedure) in Et_2O/PE 2:5 → Et_2O/PE

1:1 first afforded the β-anomer of the product β-**19** (224 mg, 21%) in 84% purity (due to the presence of an inseparable product of the ring contraction, see below) as a colorless syrup followed by the α-anomer (α-**19**) (577 mg, 55%) as a thick colorless syrup. Data for α-**19**: R_f 0.08 (EtOAc/PE 1:4), $[\alpha]_D^{20}$ +190 (*c* 1.44, $CHCl_3$). ^{1}H NMR ($CDCl_3$, 400 MHz, ^{1}H {^{19}F}, H-H COSY): δ 7.50–7.48 (m, 2H, CH_{arom}), 7.36–7.32 (m, 3H, CH_{arom}), 5.58 (dt, 1H, *J* = 5.7, 2.8 Hz, H-1), 4.89 (dddd, 1H, *J* = 53.0, 15.4, 10.0, 8.3 Hz, H-3), 4.70 (dddd, 1H, *J* = 50.9, 15.2, 9.9, 8.3 Hz, H-4), from ^{1}H {^{19}F} 4.39 (ddd, 1H, *J* = 9.9, 3.6, 2.7 Hz, H-5), 4.03 (dddd, 1H, *J* = 11.2, 10.0, 5.7, 0.9 Hz, H-2), 3.89–3.80 (m, 2H, H-6), 1.69 (t, 1H, *J* = 6.5 Hz, O*H*). ^{13}C{^{1}H} NMR ($CDCl_3$, 101 MHz, HSQC): δ 132.8 (2CH_{arom}), 132.1 (C_q), 129.5 (2CH_{arom}), 128.6 (CH_{arom}), 91.8 (dd, ^1J = 188.9 Hz, ^2J = 19.8 Hz, C-3), 86.7 (dd, ^1J = 186.2 Hz, ^2J = 18.7 Hz, C-4), 86.5 (dd, ^3J = 7.6 Hz, ^4J = 1.0 Hz, C-1), 70.2 (dd, ^2J = 25.2 Hz, ^3J = 6.2 Hz, C-5), 62.0 (dd, ^2J = 17.6 Hz, ^3J = 7.0 Hz, C-2), 60.7 (C-6). ^{19}F NMR ($CDCl_3$, 376 MHz): –193.75 (ddddd, ^2J = 53.0 Hz, ^3J = 15.2, 13.6, 11.2 Hz, ^4J = 2.8 Hz, F-3), –199.63 (dddd, ^2J = 50.9 Hz, ^3J = 15.4, 13.6 Hz, ^5J = 2.8 Hz, F-4). HRMS-APCI (*m/z*): $[M - N_2 + H]^+$ calcd for $C_{12}H_{14}F_2NO_2S$, 274.0707; found, 274.0700.

Data for β-1**9:** R_f 0.10 (EtOAc/PE 1:4). ^{1}H NMR ($CDCl_3$, 400 MHz, ^{1}H {^{19}F}, H-H COSY): δ 7.56–7.54 (m, 2H, CH_{arom}), 7.39–7.35 (m, 3H, CH_{arom}), 4.65–4.47 (m, 2H, H-3, H-4), 4.47 (dd, 1H, *J* = 10.2, 0.9 Hz, H-1), 3.97 (dt, 1H, *J* = 12.3, 2.1 Hz, H-6´), 3.79 (dd, 1H, *J* = 12.3, 3.5 Hz, H-6), 3.51–3.42 (m, 2H, H-2, H-5). ^{13}C{^{1}H} NMR ($CDCl_3$, 101 MHz, HSQC): δ 133.8 (2CH_{arom}), 130.4 (C_q), 129.5 (2CH_{arom}), 129.2 (CH_{arom}), 94.0 (dd, ^1J = 191.5 Hz, ^2J = 19.5 Hz, C-3), 86.0 (dd, ^1J = 186.0 Hz, ^2J = 18.5 Hz, C-4), 85.8 (dd, ^3J = 6.6 Hz, ^4J = 1.4 Hz, C-1), overlapped with $CDCl_3$ (C-5), 63.2 (dd, ^2J = 18.0 Hz, ^3J = 7.4 Hz, C-2), 61.1 (d, ^3J = 4.4 Hz, C-6). ^{19}F NMR ($CDCl_3$, 376 MHz): –188.79 (m, F-3), –200.73 (m, F-4). HRMS-APCI (*m/z*): $[M - N_2 + H]^+$ calcd for $C_{12}H_{14}F_2NO_2S$, 274.0707; found, 274.0703.

The β-anomer was contaminated by a co-eluting product of the ring contraction (approximately 16%) that was assigned the structure of 2,3-dideoxy-2,3-difluoro-*C*-α- or β-D-arabinofuranosyl-formaldehyde diphenyl dithioacetal **S1**. The configuration at C1 was not determined.

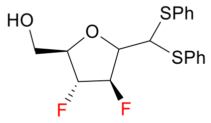

S1

Data for **S1**: ^{1}H NMR ($CDCl_3$, 400 MHz, ^{1}H {^{19}F}, H-H COSY): δ 7.50–7.46 (m, 3H, CH_{arom}), 7.34–7.31 (m, 7H, CH_{arom}), 5.54 (dddd, 1H, *J* = 51.8, 16.1, 3.6, 2.7 Hz, H-2), 5.19 (dddd, 1H, *J* = 52.6, 17.2, 4.7, 2.7 Hz, H-3), 4.53 (d, 1H, *J* = 7.1 Hz, C*H*$(SPh)_2$), 4.36 (ddd, 1H, *J* = 20.2, 7.1, 3.6 Hz, H-1), 4.24 (ddt, 1H, *J* = 20.9, 4.7, 4.4 Hz, H-4), 3.87–3.80 (m, 1H, H-5), 3.69 (dd, 1H, *J* = 12.1, 4.4 Hz, H-5´), 2.00 (br s, 1H, O*H*). ^{13}C{^{1}H} NMR ($CDCl_3$, 101 MHz, HSQC): δ 133.7, 133.6 (2 × 2CH_{arom}), 133.2, 132.7 (2 × C_q), 129.3, 129.2 (2 × 2CH_{arom}), 128.7, 128.5 (2 × CH_{arom}), 97.1 (dd, ^1J = 186.9 Hz, ^2J = 27.5 Hz, C-2), 95.3 (dd, ^1J = 184.8 Hz, ^2J = 28.1 Hz, C-3), 83.9 (dd, ^2J = 26.6 Hz, ^3J = 4.6 Hz, C-1), 82.8 (dd, ^2J = 25.9 Hz, ^3J = 4.3 Hz, C-4), 61.3 (dd, ^3J = 5.8 Hz, ^4J = 0.8 Hz, C-5), 60.4 (dd, ^3J = 6.6 Hz, ^4J = 1.5 Hz, *C*H$(SPh)_2$). ^{19}F NMR ($CDCl_3$, 376 MHz): –191.15 (dddd, ^2J = 51.8 Hz, ^3J = 20.2, 17.2, 8.7 Hz, F-2), –195.68 (dddd, ^2J = 52.6 Hz, ^3J = 20.9, 16.1, 8.7 Hz, F-2).

2-O-benzyl-3-deoxy-3-fluoro-*C*-α-D-lyxofuranosyl-formaldehyde diphenyl dithioacetal (20)

(2,5-Anhydro-4-deoxy-4-fluoro-3-*O*-benzyl-D-talose diphenyl dithioacetal)

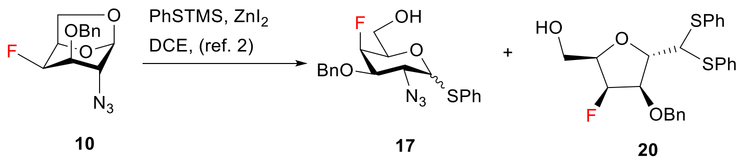

10 **17** **20**

Reaction of **10** (850 mg, 3.05 mmol) with PhSTMS (1.90 mL, 10.03 mmol) and ZnI_2 (1.70 g, 5.33 mmol) yielded known phenyl thioglycoside **17** (745 mg, 63%) as described in ref 2. Chromatography in EtOAc/PE 1:4 also provided a colorless oily by-product assigned as dithioacetal **20** (64 mg, 5%), R_f 0.12 (EtOAc/PE 1:3), $[\alpha]_D^{20}$ +24 (*c* 2.28, $CHCl_3$). ^{1}H NMR ($CDCl_3$, 400 MHz, ^{1}H {^{19}F}, H-H COSY, ROESY): δ 7.45–7.42 (m, 2H, CH_{arom}), 7.34–7.32 (m, 7H, CH_{arom}), 7.29–7.26 (m, 6H, CH_{arom}), 5.08 (ddd, 1H, *J* = 55.0, 3.7, 2.9 Hz, H-3), 4.67 (d, 1H, *J* = 11.7, C*H*H Bn), 4.60 (d, 1H, *J* = 2.2 Hz, C*H*$(SPh)_2$), 4.55 (ddd, 1H, *J* = 8.1, 2.2, 1.2 Hz, H-1), 4.51 (d, 1H, *J* = 11.7, CH*H* Bn), 4.38 (ddd, 1H, *J* = 22.7, 8.1, 3.7 Hz, H-2), 4.33 (dddd, 1H, *J* = 29.0, 6.7, 6.0, 2.9 Hz, H-4), 3.91–3.80 (m, 2H, H-5), 1.90 (dd, 1H, *J* = 6.3, 5.2 Hz, O*H*). ^{13}C{^{1}H} NMR ($CDCl_3$, 101 MHz, HSQC, HMBC): δ 137.2, 134.5, 134.3 (3 × C_q), 132.9, 132.1, 129.2, 129.1, 128.7 (5 × $2CH_{arom}$), 128.3 (CH_{arom}), 128.12 ($2CH_{arom}$), 128.1, 127.8 (2 × CH_{arom}), 89.9 (d, ^1J = 190.6 Hz, C-3), 82.1 (C-1), 81.4 (d, ^2J = 17.7 Hz, C-4), 80.3 (d, ^2J = 16.3 Hz, C-2), 72.8 (*C*H_2 Bn), 62.1 (*C*H$(SPh)_2$), 60.8 (d, ^3J = 12.1 Hz, C-5). ^{19}F NMR ($CDCl_3$, 376 MHz): –215.37 (ddd, ^2J = 54.7 Hz, ^3J = 29.0, 21.8 Hz). The 1,2-*trans* configuration of C1 and C2 substituent was established with the help of NMR spectroscopy. The vicinal coupling constant between hydrogen atoms in the positions 1 and 2 is 8.2 Hz, which indicates their almost antiperiplanar arrangement. Furthermore, a cross-peak indicating spatial proximity between H1 and H5 was observed in a ROESY spectrum. HRMS-ESI (*m/z*): $[M + Na]^+$ calcd. for $C_{25}H_{25}FO_3S_2Na$ 479.1121, found 479.1118. HRMS-APCI (*m/z*): $[M - SPh]^+$ calcd for $C_{19}H_{20}FO_3S$, 347.1111; found, 347.1111.

Phenyl 2-Azido-4-*O*-benzyl-2,3,6-trideoxy-3,6-difluoro-1-thio-α-D-glucopyranoside (22)

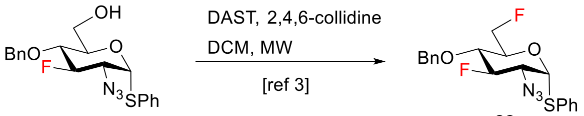

14 **22**

Phenyl 2-azido-4-*O*-benzyl-2,3,6-trideoxy-3,6-difluoro-1-thio-α-D-glucopyranoside **(22)** was prepared from α-**14** in 94% yield as described in ref 3.

Phenyl 2-Azido-3-*O*-benzyl-2,4,6-trideoxy-4,6-difluoro-1-thio-α/β-D-glucopyranoside (23)

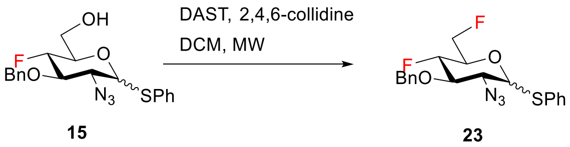

Thioglycoside **15** (380 mg, 0.98 mmol) was subjected to reaction with diethylaminosulfur trifluoride (170 μL, 1.29 mmol) and 2,4,6-collidine (340 μL, 2.57 mmol) in dichloromethane (8 mL) according to the general procedure. Chromatography of the crude product in EtOAc/PE 1:6 afforded phenyl 2-azido-3-*O*-benzyl-2,4,6-trideoxy-4,6-difluoro-1-thio-α/β-D-glucopyranoside (**23**) (355 mg, 93%) as a colorless syrupy mixture of anomers. R_f 0.83 (EtOAc/PE 1:3). For the characterization purposes, a small quantity of the anomeric mixture was separated using preparative TLC in EtOAc/PE 1:6. NMR data for the α-anomer: ^{1}H NMR ($CDCl_3$, 400 MHz, ^{1}H {^{19}F}, H-H COSY): δ 7.50–7.47 (m, 2H, CH_{arom}), 7.44–7.31 (m, 8H, CH_{arom}), 5.58 (dd, 1H, J = 4.7, 2.8 Hz, H-1), 4.93 (dd, 1H, J = 10.9, 1.2 Hz, C*H*H Bn), 4.83 (d, 1H, J = 10.9 Hz, C*H*H Bn), 4.76–4.63 (m, 3H, H-4, H-5, H-6´), 4.62 (ddt, 1H, J = 47.8, 10.6, 1.8 Hz, H-6), 3.98–3.88 (m, 2H, H-2, H-3). ^{13}C{^{1}H} NMR ($CDCl_3$, 101 MHz, HSQC): δ 137.3, 132.8 (2 × C_q), 132.1, 129.4, 128.7, 128.4 (4 × 2CH_{arom}), 128.3, 128.2 (2 × CH_{arom}), 89.5 (dd, ^1J = 184.9 Hz, ^3J = 6.9 Hz, C-4), 87.0 (d, ^4J = 1.4 Hz, C-1), 80.8 (d, ^1J = 175.4 Hz, C-6), 79.1 (d, ^2J = 17.8 Hz, C-3), 75.2 (d, ^4J = 2.7 Hz, CH_2 Bn), 69.4 (dd, ^2J = 25.2, 18.2 Hz, C-5), 62.6 (d, ^3J = 8.7 Hz, C-2). ^{19}F NMR ($CDCl_3$, 376 MHz): −195.75 (m, F-4), −236.22 (ddd, ^2J = 47.8, 46.8 Hz, ^3J = 26.2 Hz, F-6). NMR data for the β-anomer: 7.59–7.56 (m, 2H, CH_{arom}), 7.39–7.30 (m, 8H, CH_{arom}), 4.87 (dd, 1H, J = 10.9, 1.1 Hz, C*H*H Bn), 4.77 (d, 1H, J = 10.9 Hz, C*H*H Bn), 4.69 (ddt, 1H, J = 47.4, 10.4, 1.8 Hz, H-6), 4.61 (dddd, 1H, J = 46.1, 10.4, 3.9, 1.8 Hz, H-6´), 4.48 (ddd, 1H, J = 50.4, 10.0, 8.5 Hz, H-4), 4.41 (d, 1H, J = 10.2 Hz, H-1), from ^{1}H {^{19}F} 3.62 (dd, 1H, J = 9.3, 8.5 Hz, H-3), 3.67–3.54 (m, 1H, H-5), 3.31 (ddd, 1H, J = 10.2, 9.3, 0.8 Hz, H-2). ^{13}C{^{1}H} NMR ($CDCl_3$, 101 MHz, HSQC): δ 137.2 (C_q), 134.2 (2CH_{arom}), 137.2 (C_q), 129.3 (2CH_{arom}), 129.0 (CH_{arom}), 128.7, 128.5 (2 × 2CH_{arom}), 128.4 (CH_{arom}), 88.6 (dd, ^1J = 184.6 Hz, ^3J = 7.2 Hz, C-4), 86.2 (d, ^4J = 1.5 Hz, C-1), 82.1 (dd, ^2J = 17.6 Hz, ^4J = 0.6 Hz, C-3), 80.9 (d, ^1J = 175.9 Hz, C-6), 76.4 (dd, ^2J = 23.4, 18.8 Hz, C-5), 75.3 (d, ^4J = 2.7 Hz, *C*H_2 Bn), 63.9 (d, ^3J = 8.9 Hz, C-2). ^{19}F NMR ($CDCl_3$, 376 MHz): −197.07 (dd, ^2J = 50.4 Hz, ^3J = 14.5 Hz F-4), −236.00 (ddd, ^2J = 47.4, 46.1 Hz, ^3J = 23.8 Hz, F-6). HRMS-APCI (*m/z*): $[M - N_2 + H]^+$ calcd for $C_{19}H_{20}F_2NO_2S$, 364.1177; found, 364.1176.

Phenyl 2-Azido-4-*O*-benzyl-2,3,6-trideoxy-3,6-difluoro-1-thio-α-D-galactopyranoside (α-24)

Phenyl 2-Azido-4-*O*-benzyl-2,3,6-trideoxy-3,6-difluoro-1-thio-β-D-galactopyranoside (β-24)

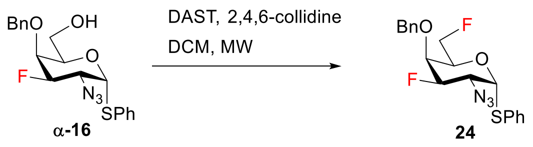

Thioglycoside α-**16** (245 mg, 0.63 mmol) was subjected to reaction with diethylaminosulfur trifluoride (100 μL, 0.76 mmol) and 2,4,6-collidine (200 μL, 1.51 mmol) in dichloromethane (5 mL) according to the general procedure. Chromatography of the crude product in EtOAc/PE 1:6 afforded phenyl 2-azido-4-*O*-benzyl-2,3,6-trideoxy-3,6-difluoro-1-thio-α-D-galactopyranoside (α-**24**) (210 mg, 85%) as a colorless syrup.

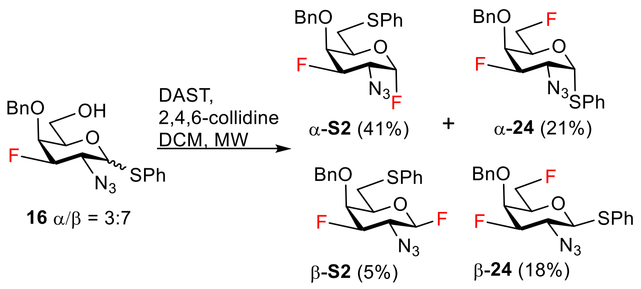

In another experiment, a mixture of anomers of thioglycoside **16** (α/β 3:7, 500 mg, 1.29 mmol) was subjected to reaction with diethylaminosulfur trifluoride (213 μL, 1.61 mmol) and 2,4,6-collidine (425 μL, 3.22 mmol) in dichloromethane (10 mL) according to the general procedure. Chromatography of the crude product in EtOAc/PE 1:6 afforded sequentially 2-azido-4-*O*-benzyl-2,3,6-trideoxy-3-fluoro-6-*S*-phenyl-6-thio-α-D-galactopyranosyl fluoride (α-**S2**) (205 mg, 41%) as a white crystalline solid, phenyl 2-azido-4-*O*-benzyl-2,3,6-trideoxy-3,6-difluoro-1-thio-α-D-galactopyranoside (α-**24**) (107 mg, 21%) as a colorless syrup, 2-azido-4-*O*-benzyl-2,3,6-trideoxy-3-fluoro-6-*S*-phenyl-6-thio-β-D-galactopyranosyl fluoride (β-**S2**) (27 mg, 5%) as a colorless syrup and phenyl 2-azido-4-*O*-benzyl-2,3,6-trideoxy-3,6-difluoro-1-thio-β-D-galactopyranoside (β-**24**) (90 mg, 18%) as a colorless syrup. Data for α-**24:** R_f 0.85 (EtOAc/PE 1:3).^{1}H NMR ($CDCl_3$, 400 MHz, ^{1}H {^{19}F}, H-H COSY): δ 7.50–7.47 (m, 2H, CH_{arom}), 7.40–7.29 (m, 8H, CH_{arom}), 5.62 (dd, 1H, J = 5.6, 4.3 Hz, H-1), 4.92 (d, 1H, J = 11.2 Hz, C*H*H Bn), 4.49 (ddd, 1H, J = 48.0, 10.4, 3.1 Hz, H-3), 4.60 (d, 1H, J = 11.2 Hz, CH*H* Bn), 4.60–4.50 (m, 1H, H-5), from ^{1}H {^{19}F} 4.52 (dd, 1H, J = 10.4, 5.6 Hz, H-2), 4.47 (dddd, 1H, J = 46.8, 9.3, 6.4, 1.1 Hz, H-6´), 4.41 (ddd, 1H, J = 46.1, 9.4, 5.9 Hz, H-6), 4.11 (ddd, 1H, J = 6.6, 3.1, 1.3 Hz, H-4). ^{13}C{^{1}H} NMR ($CDCl_3$, 101 MHz, HSQC): δ 137.4 (C_q), 132.69 (2CH_{arom}), 132.67 (C_q), 129.3, 128.7, 128.5 (3 × 2CH_{arom}), 128.4, 128.3 (2 × CH_{arom}), 91.8 (d, ^1J = 190.8 Hz, C-3), 87.3 (d, ^3J = 7.7 Hz, C-1), 81.4 (dd, ^1J = 169.1, ^4J = 2.8 Hz, C-6), 75.3 (d, ^4J = 4.2 Hz, CH_2 Bn), 73.6 (dd, ^2J = 15.5 Hz, ^3J = 5.4 Hz, C-4), 69.4 (dd, ^2J = 24.6 Hz, ^3J = 7.0 Hz, C-5), 59.6 (d, ^2J = 18.0 Hz, C-2). ^{19}F NMR ($CDCl_3$, 376 MHz): –196.89 (dddd, ^2J = 48.0 Hz, ^3J = 9.6, 6.6 Hz, ^4J = 4.3 Hz, F-3), –231.60 (dddd, ^2J = 46.8, 46.1 Hz, ^3J = 11.9 Hz, J = 2.0 Hz, F-6). HRMS-APCI (*m/z*): $[M - N_2 + H]^+$ calcd for $C_{19}H_{20}F_2NO_2S$, 364.1177; found, 364.1178.

Data for β-**24**: R_f 0.42 (Et_2O/PE 1:6), ^{1}H NMR ($CDCl_3$, 400 MHz, ^{1}H {^{19}F}, H-H COSY): δ 7.59–7.57 (m, 2H, CH_{arom}), 7.37–7.25 (m, 8H, CH_{arom}), 4.87 (d, 1H, *J* = 11.4 Hz, C*H*H Bn), 4.58 (dddd, 1H, *J* = 46.8, 9.3, 6.2, 1.3 Hz, H-6´), 4.57 (d, 1H, *J* = 11.4 Hz, C*H*H Bn), 4.49 (ddd, 1H, *J* = 47.4, 9.6, 3.1 Hz, H-3), 4.41 (dd, 1H, *J* = 10.1, 0.9 Hz, H-1), 4.43 (ddd, 1H, *J* = 46.0, 9.3, 6.4 Hz, H-6), 4.00 (ddd, 1H, *J* = 6.4, 3.1, 1.2 Hz, H-4), 3.92 (ddd, 1H, *J* = 11.0, 10.1, 9.6 Hz, H-2), 3.71 (ddddd, 1H, *J* = 9.6, 6.4, 6.2, 1.5, 1.2 Hz, H-5). ^{13}C{^{1}H} NMR ($CDCl_3$, 101 MHz, HSQC): δ 137.6 (C_q), 133.4 (2CH_{arom}), 131.2 (C_q), 129.2, 128.6 (2 × 2CH_{arom}), 128.5, 128.1 (2 × CH_{arom}), 128.1 (2CH_{arom}), 94.4 (d, ^1J = 193.1 Hz, C-3), 86.2 (d, ^3J = 6.9 Hz, C-1), 81.0 (dd, ^1J = 169.1, ^4J = 2.9 Hz, C-6), 75.6 (dd, ^2J = 24.4 Hz, ^3J = 7.7 Hz, C-5), 74.8 (d, ^4J = 4.0 Hz, *C*H_2 Bn), 72.5 (dd, ^2J = 15.8 Hz, ^3J = 4.7 Hz, C-4), 60.7 (d, ^2J = 18.2 Hz, C-2). ^{19}F NMR ($CDCl_3$, 376 MHz): –190.12 (ddd, ^2J = 47.4 Hz, ^3J = 11.0, 6.4 Hz, F-3), –231.82 (ddd, ^2J = 46.8, 46.0 Hz, ^3J = 9.6 Hz, F-6). HRMS-APCI (*m/z*): $[M - N_2 + H]^+$ calcd for $C_{19}H_{20}F_2NO_2S$, 364.1177; found, 364.1179.

Data for α-**S2**: R_f 0.50 (Et_2O/PE 1:6), ^{1}H NMR ($CDCl_3$, 400 MHz, ^{1}H {^{19}F}, H-H COSY): δ 7.37–7.23 (m, 10H, CH_{arom}), 5.66 (ddd, 1H, *J* = 52.7, 5.4, 2.8 Hz, H-1), 4.94 (ddd, 1H, *J* = 48.5, 10.4, 3.0 Hz, H-3), 4.93, 4.54 (2 × d, 2 × 1H, *J* = 11.1 Hz, C*H*H Bn), 4.34 (ddd, 1H, *J* = 7.4, 3.0, 1.3 Hz, H-4), 4.03 (dddd, 1H, *J* = 25.3, 10.4, 9.6, 2.8 Hz, H-2), from ^{1}H {^{19}F-3} 3.99 (ddd, 1H, *J* = 8.8, 5.4, 1.3 Hz, H-5), 3.20 (ddd, 1H, *J* = 13.7, 5.4, 2.0 Hz, H-6´), 3.10 (ddd, 1H, *J* = 13.7, 8.8, 0.8 Hz, H-6). ^{13}C{^{1}H} NMR ($CDCl_3$, 101 MHz, HSQC): δ 137.5, 134.8 (2 × C_q), 129.9, 129.4, 128.7, 128.5 (4 × 2CH_{arom}), 128.3, 127.0 (2 × CH_{arom}), 106.6 (dd, ^1J = 227.3 Hz, ^3J = 9.8 Hz, C-1), 90.6 (d, ^1J = 189.6 Hz, C-3), 75.4 (d, ^4J = 4.2 Hz, *C*H_2 Bn), 73.8 (d, ^2J = 15.8 Hz, C-4), 71.9 (dd, ^3J = 7.0, 3.3 Hz, C-5), 58.9 (dd, ^2J = 23.4, 18.4 Hz, C-2), 33.2 (d, ^3J = 2.1 Hz, C-6). ^{19}F NMR ($CDCl_3$, 376 MHz): –147.65 (dd, ^2J = 52.7 Hz, ^3J = 25.3 Hz, F-1), –199.58 (dddd, ^2J = 48.5 Hz, ^3J = 9.6, 7.4 Hz, ^4J = 5.4 Hz, F-3). HRMS-APCI (*m/z*): $[M - N_2 + H]^+$ calcd for $C_{19}H_{20}F_2NO_2S$, 364.1177; found, 364.1179.

Data for β-**S2**: R_f 0.46 (Et_2O/PE 1:6), ^{1}H NMR ($CDCl_3$, 400 MHz, ^{1}H {^{19}F}, H-H COSY): δ 7.38–7.27 (m, 10H, CH_{arom}), 4.93 (d, 1H, *J* = 11.3 Hz, C*H*H Bn), 4.93 (dd, 1H, *J* = 52.2, 7.4 Hz, H-1), 4.55 (d, 1H, *J* = 11.3 Hz, C*H*H Bn), 4.41 (dddd, 1H, *J* = 47.2, 10.1, 3.2, 1.1 Hz, H-3), from ^{1}H {^{19}F-3} 4.20 (d, 1H, *J* = 3.2 Hz, H-4), 4.14–3.99 (m, 1H, H-2), from ^{1}H {^{19}F-3} 3.47 (dd, 1H, *J* = 8.8, 5.1 Hz, H-5), 3.25 (ddd, 1H, *J* = 13.9, 5.1, 1.9 Hz, H-6´), 3.15 (dd, 1H, *J* = 13.9, 8.8 Hz, H-6). ^{13}C{^{1}H} NMR ($CDCl_3$, 101 MHz, HSQC): δ 137.4, 134.7 (2 × C_q), 129.8, 129.4, 128.7, 128.5 (4 × 2CH_{arom}), 128.3, 127.1 (2 × CH_{arom}), 107.7 (dd, ^1J = 216.1 Hz, ^3J = 11.3 Hz, C-1), 92.7 (dd, ^1J = 192.5 Hz, ^3J = 10.9 Hz, C-3), 75.3 (d, ^4J = 4.1 Hz, *C*H_2 Bn), 72.8 (dd, ^3J = 7.7, 4.3 Hz, C-5), 72.5 (dd, ^2J = 15.5 Hz, ^4J = 1.0 Hz, C-4), 62.3 (dd, ^2J = 22.3, 18.5 Hz, C-2), 33.1 (d, ^3J = 2.4 Hz, C-6). ^{19}F NMR ($CDCl_3$, 376 MHz): –141.26 (dddd, ^2J = 52.2 Hz, ^3J = 13.2 Hz, ^4J = 3.4, 2.6 Hz, F-1), –197.09 (ddddd, ^2J = 47.2 Hz, ^3J = 9.0, 5.9 Hz, ^4J = 3.4, 1.5 Hz, F-3). HRMS-APCI (*m/z*): $[M - N_2 + H]^+$ calcd for $C_{19}H_{20}F_2NO_2S$, 364.1177; found, 364.1175.

Phenyl 2-Azido-3-*O*-benzyl-2,4,6-trideoxy-4,6-difluoro-1-thio-α-D-galactopyranoside (25)

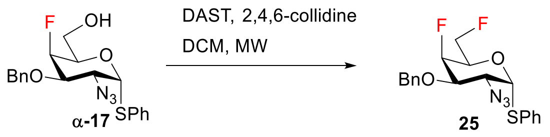

Thioglycoside α-**17** (345 mg, 0.89 mmol) was subjected to reaction with diethylaminosulfur trifluoride (150 μL, 1.14 mmol) and 2,4,6-collidine (300 μL, 2.27 mmol) in dichloromethane (7 mL) according to the general procedure. Chromatography of the crude product in EtOAc/PE 1:6 afforded phenyl 2-azido-3-*O*-benzyl-2,4,6-trideoxy-4,6-difluoro-1-thio-α-D-galactopyranoside (**25**) (300 mg, 87%) as a yellowish syrup. R_f 0.78 (EtOAc/PE 1:3). ^{1}H NMR ($CDCl_3$, 400 MHz, ^{1}H {^{19}F}, H-H COSY): δ 7.51–7.48 (m, 2H, CH_{arom}), 7.44–7.30 (m, 8H, CH_{arom}), 5.61 (d, 1H, J = 5.4 Hz, H-1), 4.90 (dd, 1H, J = 50.3, 2.5 Hz, H-4), 4.80, 4.76 (2 × d, 2 × 1H, J = 11.7 Hz, C*H*H Bn), 4.67–4.53 (m, 2H, H-5, H-6´), 4.51 from ^{1}H {^{19}F-6} (ddd, 1H, J = 8.1, 5.5, 1.4 Hz, H-6), 4.30 (dd, 1H, J = 10.6, 5.4 Hz, H-2), 3.76 (ddd, 1H, J = 26.2, 10.6, 2.5 Hz, H-3). ^{13}C{^{1}H} NMR ($CDCl_3$, 101 MHz, HSQC): δ 136.9 (C_q), 132.7 ($2CH_{arom}$), 132.6 (C_q), 129.4, 128.8 (2 × $2CH_{arom}$), 128.4, 128.3 (2 × CH_{arom}), 128.1 ($2CH_{arom}$), 87.4 (C-1), 85.0 (dd, ^1J = 185.9 Hz, ^3J = 5.0 Hz, C-4), 80.7 (dd, ^1J = 170.0 Hz, ^3J = 6.3 Hz, C-6), 75.9 (d, ^2J = 17.8 Hz, C-3), 72.1 (CH_2 Bn), 68.7 (dd, ^2J = 24.6, 18.0 Hz, C-5), 59.7 (d, ^3J = 1.9 Hz, C-2). ^{19}F NMR ($CDCl_3$, 376 MHz): –218.13 (ddd, ^2J = 50.3 Hz, ^3J = 30.2, 26.2 Hz, F-4), –232.28 (m, F-6). HRMS-APCI (*m/z*): $[M - N_2 + H]^+$ calcd for $C_{19}H_{20}F_2NO_2S$, 364.1177; found, 364.1177.

Phenyl 2-Azido-2,3,4,6-tetradeoxy-3,4,6-trifluoro-1-thio-α-D-galactopyranoside (26)

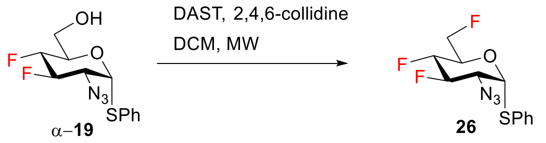

Thioglycoside α-**19** (400 mg, 1.33 mmol) was subjected to reaction with diethylaminosulfur trifluoride (190 μL, 1.44 mmol) and 2,4,6-collidine (380 μL, 2.88 mmol) in dichloromethane (8 mL) according to the general procedure. Chromatography of the crude product in EtOAc/PE 1:5 afforded the phenyl 2-azido-2,3,4,6-tetradeoxy-3,4,6-trifluoro-1-thio-α-D-glucopyranoside (**26**) (359 mg, 89%) as a colorless syrup. R_f 0.78 (EtOAc/PE 1:3). ^{1}H NMR ($CDCl_3$, 400 MHz, ^{1}H {^{19}F}, H-H COSY): δ 7.48–7.46 (m, 2H, CH_{arom}), 7.36–7.32 (m, 3H, CH_{arom}), 5.61 (dt, 1H, J = 5.8, 2.8 Hz, H-1), 4.89 (dddd, 1H, J = 52.8, 15.3, 10.1, 8.2 Hz, H-3), from ^{1}H {^{19}F-6} 4.70 (ddd, 1H, J = 10.7, 3.3, 1.5 Hz, H-6´), from ^{1}H {^{19}F-6} 4.59 (dq, 1H, J = 10.7, 1.7 Hz, H-6), 4.80–4.42 (m, 2H, H-4, H-5), 4.06 (dddd, 1H, J = 11.1, 10.0, 5.8, 0.9 Hz, H-2). ^{13}C{^{1}H} NMR ($CDCl_3$, 101 MHz, HSQC): δ 132.3 ($2CH_{arom}$), 132.2 (C_q), 129.5 ($2CH_{arom}$), 128.5 (CH_{arom}), 91.6 (ddd, ^1J = 189.1 Hz, ^2J = 19.7 Hz, ^4J = 0.8 Hz, C-3), 86.5 (dd, ^3J = 7.5 Hz, ^4J = 1.3 Hz, C-1), 86.0 (ddd, ^1J = 187.4 Hz, ^2J = 18.7 Hz, ^3J = 7.7 Hz, C-4), 80.5 (dt, ^1J = 173.4 Hz, $^{3,4}J$ = 0.9 Hz, C-6), 69.0 (ddd, ^2J = 24.8, 18.2 Hz, ^3J = 6.9 Hz, C-5), 61.8 (dd, ^2J = 17.6 Hz, ^3J = 6.7 Hz, C-2). ^{19}F NMR ($CDCl_3$, 376 MHz):

–193.64 (dddddd, ^2J = 52.8 Hz, ^3J = 14.8, 13.5, 11.1 Hz, ^4J = 2.8 Hz, ^5J = 1.7 Hz, F-3), –199.57 (ddddd, ^2J = 51.0 Hz, ^3J = 15.3, 13.5, 3.9 Hz, ^5J = 2.8 Hz, F-4), –235.58 (td, ^2J = 47.2 Hz, ^3J = 26.7 Hz, F-6). HRMS-APCI (*m/z*): $[M - N_2 + H]^+$ calcd for $C_{12}H_{13}F_3NOS$, 276.0664; found, 276.0667.

Phenyl 2-azido-4,6-di-*O*-benzyl-2,3-dideoxy-3-fluoro-1-thio-α/β-D-glucopyranoside (27)

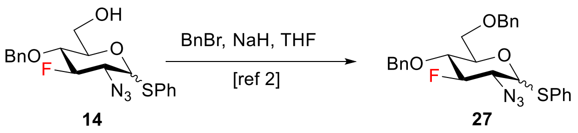

Phenyl 2-azido-4,6-di-*O*-benzyl-2,3-dideoxy-3-fluoro-1-thio-α/β-D-glucopyranoside (**27**) was prepared from **14** in 81% yield as described in ref 2.

Phenyl 2-Azido-3,6-di-*O*-benzyl-2,4-dideoxy-4-fluoro-1-thio-α/β-D-glucopyranoside (28)

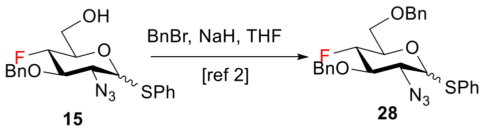

Phenyl 2-azido-3,6-di-*O*-benzyl-2,4-dideoxy-4-fluoro-1-thio-α/β-D-glucopyranoside (**28**) was prepared from **15** in 86% yield as described in ref 2.

Phenyl 2-Azido-4,6-di-*O*-benzyl-2,3-dideoxy-3-fluoro-1-thio-β-D-galactopyranoside (29)

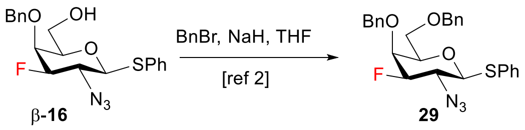

Phenyl 2-azido-4,6-di-*O*-benzyl-2,3-dideoxy-3-fluoro-1-thio-β-D-galactopyranoside (**29**) was prepared from β-**16** in 93% yield as described in ref 2.

Phenyl 2-Azido-3,6-di-*O*-benzyl-2,4-dideoxy-4-fluoro-1-thio-β-D-galactopyranoside (30)

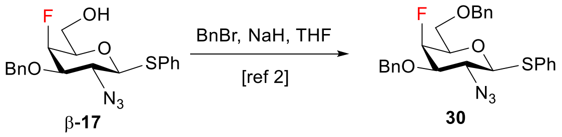

Phenyl 2-azido-3,6-di-*O*-benzyl-2,4-dideoxy-4-fluoro-1-thio-β-D-galactopyranoside (**30**) was prepared from β-**17** in 95% yield as described in ref 2.

Phenyl 2-Azido-6-*O*-benzyl-2,3,4-trideoxy-3,4-difluoro-1-thio-α/β-D-galactopyranoside (31)

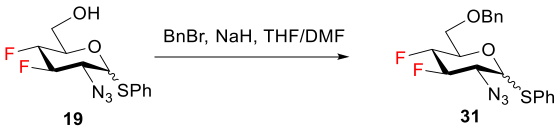

Thioglycoside **19** (285 mg, 0.95 mmol) was dissolved in THF/DMF 10:1 (5.5 mL) and the resulting solution was cooled to −25 °C. Sodium hydride (60% suspension in oil, 49 mg, 1.23 mmol) was added, the reaction mixture was stirred for 5 min, and then benzyl bromide (180 μL, 1.51 mmol) and tetrabutylammonium iodide (cat.) were added. The temperature was allowed to reach rt and the reaction mixture was stirred overnight. TLC (EtOAc/PE 1:4) indicated the absence of the starting material. The reaction was cooled to 0 °C, MeOH (0.3 mL) was added to quench the reaction. After stirring for additional 30 min was the reaction mixture diluted with chloroform, washed with water, the water phase was extracted with chloroform, the combined chloroform phases were dried and concentrated. Chromatography of the residue in EtOAc/PE 2:15 afforded phenyl 2-azido-6-*O*-benzyl-2,3,4-trideoxy-3,4-difluoro-1-thio-α/β-D-glucopyranoside (**31**) (285 mg, 77%) as a colorless syrup, R_f 0.2 (EtOAc/PE 2:15). NMR data for the α-anomer: ^{1}H NMR ($CDCl_3$, 400 MHz, ^{1}H {^{19}F}, H-H COSY): δ 7.61–7.59 (m, 1H, CH_{arom}), 7.51–7.49 (m, 2H, CH_{arom}), 7.38–7.27 (m, 7H, CH_{arom}), 5.60 (dt, 1H, J = 5.8, 2.9 Hz, H-1), 4.86 (dddd, 1H, J = 53.0, 15.5, 10.1, 8.2 Hz, H-3), 4.75 from ^{1}H {^{19}F} (dd, 1H, J = 9.9, 8.2 Hz, H-4), 4.64, 4.53 (2 × d, 2 × 1H, J = 12.0 Hz, C*H*H Bn), 4.55–4.47 (m, 1H, H-5), from ^{1}H {^{19}F} 4.06 (dd, 1H, J = 10.1, 5.8 Hz, H-2), 3.78 (ddt, 1H, J = 11.2, 9.3, 6.4, 1.1 Hz, H-6´), 3.73 (dd, 1H, J = 11.2, 1.5 Hz, H-6). ^{13}C{^{1}H} NMR ($CDCl_3$, 101 MHz, HSQC, HMBC): δ 137.7 (C_q), 133.9 (CH_{arom}), 132.5 (2CH_{arom}), 132.4 (C_q), 129.4, 128.6 (2 × 2CH_{arom}), 128.4 (CH_{arom}), 127.8 (2CH_{arom}), 91.9 (dd, ^{1}J = 188.7 Hz, ^{2}J = 19.7 Hz, C-3), 87.0 (dd, ^{1}J = 186.6 Hz, ^{2}J = 18.2 Hz, C-4), 86.5 (dd, ^{3}J = 7.7 Hz, ^{4}J = 0.9 Hz,C-1), 73.8 (*C*H_2 Bn), 69.5 (dd, ^{2}J = 24.3 Hz, ^{3}J = 6.5 Hz, C-5), 67.6 (dd, ^{3}J = 1.0, ^{4}J = 0.9 Hz, C-6), 62.0 (dd, ^{2}J = 17.4 Hz, ^{3}J = 6.9 Hz, C-2). ^{19}F NMR ($CDCl_3$, 376 MHz): –193.48 (dq, ^{2}J = 53.0 Hz, ^{3}J = 13.6 Hz, F-3), –199.26 (m, F-4). NMR data for the β-anomer: ^{1}H NMR ($CDCl_3$, 400 MHz, ^{1}H {^{19}F}, H-H COSY): δ 7.61–7.59 (m, 1H, CH_{arom}), 7.38–7.27 (m, 9H, CH_{arom}), 4.64, 4.60 (2 × d, 2 × 1H, J = 11.2 Hz, C*H*H Bn), 4.73–4.43 (m, 2H, H-3, H-4), 4.43 (dd, 1H, J = 10.2, 0.8 Hz, H-1), 3.84 (ddt, 1H, J = 11.4, 2.3, 2.0 Hz, H-6´), 3.73 (ddd, 1H, J = 11.4, 4.6, 2.3 Hz, H-6), 3.58 (dddd, 1H, J = 9.1, 4.6, 2.3, 2.0 Hz, H-5), 4.48 (ddd, 1H, J = 13.0, 10.2, 8.8 Hz, H-2). ^{13}C{^{1}H} NMR ($CDCl_3$, 101 MHz, HSQC, HMBC): δ 138.0 (C_q), 133.9 (CH_{arom}), 130.6 (C_q), 129.4 (2CH_{arom}), 129.0 (CH_{arom}), 128.6 (2CH_{arom}), 128.0, 127.9 (2 × CH_{arom}), 127.7 (2CH_{arom}), 94.1 (dd, ^{1}J = 191.3 Hz, ^{2}J = 19.5 Hz, C-3), 86.3 (dd, ^{1}J = 186.7 Hz, ^{2}J = 18.4 Hz, C-4), 85.7 (dd, ^{3}J = 6.8 Hz, ^{4}J = 1.4 Hz, C-1), 76.6 (dd, ^{2}J = 22.7 Hz, ^{3}J = 6.9 Hz, C-5), 73.8 (*C*H_2 Bn), 68.1 (d, ^{3}J = 1.6 Hz, C-6), 63.1 (dd, ^{2}J = 17.9 Hz, ^{3}J = 7.3 Hz, C-2). ^{19}F NMR ($CDCl_3$, 376 MHz): –188.64 (dddd, ^{2}J = 51.5 Hz, ^{3}J = 13.7, 13.3, 13.0 Hz, F-3), –201.15 (dddq, ^{2}J = 51.8 Hz, ^{3}J = 16.2, 13.7, 2.3 Hz, F-4). HRMS-APCI (*m/z*): $[M - N_2 + H]^+$ calcd for $C_{19}H_{20}F_2NO_2S$, 364.1177; found, 364.1171.

Phenyl 2-Azido-3-*O*-benzyl-2,4,6-trideoxy-4,6-difluoro-β-D-galactopyranoside (β-25)

2-Azido-3-*O*-benzyl-2,4,6-trideoxy-4-fluoro-6-*S*-phenyl-6-thio-α-D-galactopyranosyl fluoride (32)

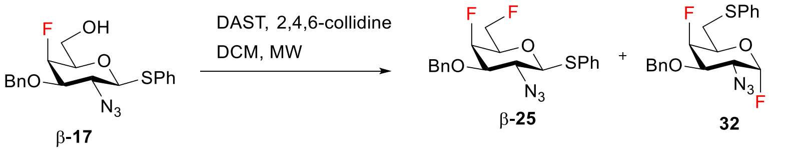

Thioglycoside β-**17** (50 mg, 0.13 mmol) was subjected to reaction with diethylaminosulfur trifluoride (21 μL, 0.17 mmol) and 2,4,6-collidine (41 μL, 0.31 mmol) in dichloromethane (1 mL) according to the general procedure. Chromatography of the crude product in EtOAc/PE 1:7 first afforded fluoride **32** (29 mg, 57%) as a colorless syrup, followed by thioglycoside β-**25** (17 mg, 34%) as a colorless syrup.

Data for β-**25**: R_f 0.62 (EtOAc/PE 1:3), $[\alpha]_D^{20}$ −62 (*c* 0.97, $CHCl_3$). ^{1}H NMR ($CDCl_3$, 400 MHz, ^{1}H {^{19}F}, H-H COSY): δ 7.58–7.56 (m, 2H, CH_{arom}), 7.38–7.32 (m, 8H, CH_{arom}), 4.79 (dd, 1H, *J* = 50.0, 2.5 Hz, H-4), 4.77, 4.70 (2 × d, 2 × 1H, *J* = 11.7 Hz, C*H*H Bn), 4.59 (dddd, 1H, *J* = 46.4, 9.4, 6.0, 1.0 Hz, H-6), 4.57 (ddd, 1H, *J* = 46.1, 9.4, 6.6 Hz, H-6´), 4.40 (dd, 1H, *J* = 10.1, 0.8 Hz, H-1), 3.71 (dddd, 1H, *J* = 26.6, 9.9, 6.6, 6.0 Hz, H-5), 3.66 (ddd, 1H, *J* = 10.1, 9.7, 1.0 Hz, H-2), 3.42 (ddd, 1H, *J* = 27.6, 9.7, 2.5 Hz, H-3). ^{13}C{^{1}H} NMR ($CDCl_3$, 101 MHz, HSQC): δ 136.8 (C_q), 133.9 ($2CH_{arom}$), 130.8 (C_q), 129.3 ($2CH_{arom}$), 128.8 ($3CH_{arom}$), 128.5 (CH_{arom}), 128.2 ($2CH_{arom}$), 86.5 (C-1), 83.9 (dd, ^1J = 185.6 Hz, ^3J = 4.3 Hz, C-4), 80.3 (dd, ^1J = 170.1 Hz, ^3J = 5.8 Hz, C-6), 79.1 (d, ^2J = 17.9 Hz, C-3), 75.2 (dd, ^2J = 24.3, 18.2 Hz, C-5), 72.2 (*C*H_2 Bn), 61.0 (d, ^3J = 1.1 Hz, C-2). ^{19}F NMR ($CDCl_3$, 376 MHz): –219.21 (ddd, ^2J = 50.0 Hz, ^3J = 27.6, 26.6 Hz, F-4), –232.58 (ddd, ^2J = 46.4, 46.1 Hz, ^3J = 9.9 Hz, F-6). HRMS-APCI (*m/z*): $[M - N_2 + H]^+$ calcd for $C_{19}H_{20}F_2NO_2S$, 364.1177; found, 364.1179.

Data for **32**: R_f 0.67 (EtOAc/PE 1:3), ^{1}H NMR ($CDCl_3$, 400 MHz, ^{1}H {^{19}F}, H-H COSY): δ 7.40–7.30 (m, 8H, CH_{arom}), 7.27–7.23 (m, 2H, CH_{arom}), 5.65 (dd, 1H, *J* = 52.6, 2.1 Hz, H-1), 5.04 (dd, 1H, *J* = 49.5, 1.9 Hz, H-4), 4.76, 4.71 (2 × d, 2 × 1H, *J* = 11.6 Hz, C*H*H Bn), 3.94 (ddd, 1H, *J* = 27.3, 8.6, 5.8 Hz, H-5), 3.89–3.78 (m, 2H, H-2, H-3), 3.23 (ddd, 1H, *J* = 14.1, 5.8, 1.0 Hz, H-6), 3.16 (dd, 1H, *J* = 14.1, 8.6 Hz, H-6´). ^{13}C{^{1}H} NMR ($CDCl_3$, 101 MHz, HSQC): δ 136.8, 134.5 (2 × C_q), 130.0, 129.4, 128.8 (3 × $2CH_{arom}$), 128.5 (CH_{arom}), 128.2 ($2CH_{arom}$), 127.2 (CH_{arom}), 106.2 (d, ^1J = 227.7 Hz, C-1), 84.9 (d, ^1J = 186.7 Hz, C-4), 74.3 (d, ^2J = 18.2 Hz, C-3), 71.9 (*C*H_2 Bn), 71.0 (dd, ^2J = 18.9 Hz, ^3J = 3.3 Hz, C-5), 58.9 (dd, ^2J = 23.6 Hz, ^3J = 2.7 Hz, C-2), 32.9 (d, ^3J = 4.9 Hz, C-6). ^{19}F NMR ($CDCl_3$, 376 MHz): –148.74 (m, F-1), –221.17 (m, F-4). HRMS-APCI (*m/z*): $[M - N_2 + H]^+$ calcd for $C_{19}H_{20}F_2NO_2S$, 364.1177; found, 364.1175.

Deoxyfluorination of the β-anomer of thioglycoside 14

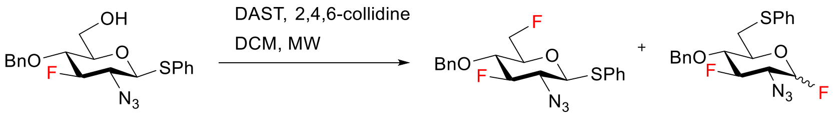

Thioglycoside β-**14** (50 mg, 0.13 mmol) obtained from compound **7** as described in ref 2 was subjected to reaction with diethylaminosulfur trifluoride (21 μL, 0.17 mmol) and 2,4,6-collidine (41 μL, 0.31 mmol) in dichloromethane (1 mL) according to the general procedure. Chromatography of the crude product in EtOAc/PE 1:7 afforded phenyl 2-azido-4-*O*-benzyl-2,3,6-trideoxy-3,6-difluoro-β-D-glucopyranoside (β-**22**) (43 mg, 85%) as a colorless syrup containing 2-azido-4-*O*-benzyl-2,3,6-trideoxy-3-fluoro-6-*S*-phenyl-6-thio-α/β-D-glucopyranosyl fluoride (**33**) (**33**/β-**22** *ca* 7:93), inseparable under given chromatographic conditions, R_f 0.79 (EtOAc/PE 1:3).

Data for β-**22**: ^{1}H NMR ($CDCl_3$, 400 MHz, ^{1}H {^{19}F}, H-H COSY): δ 7.62–7.60 (m, 2H, CH_{arom}), 7.39–7.32 (m, 8H, CH_{arom}), 4.86 (dd, 1H, *J* = 11.1, 1.3 Hz, C*H*H Bn), 4.64 (dddd, 1H, *J* = 47.6, 10.3, 2.0, 1.6 Hz, H-6), 4.62 (d, 1H, *J* = 11.1 Hz, CH*H* Bn), 4.62 (ddd, 1H, *J* = 46.9, 10.3, 3.4 Hz, H-6´), 4.56 (ddd, 1H, *J* = 51.4, 9.1, 8.6 Hz, H-3), 4.41 (dd, 1H, *J* = 10.2, 0.8 Hz, H-1), 3.68 (ddd, 1H, *J* = 12.5, 10.0, 8.6 Hz, H-4), 3.46 (ddd, 1H, *J* = 13.0, 10.2, 9.1 Hz, H-2), 3.44 (dddt, 1H, *J* = 26.2, 10.0, 3.4, 1.6 Hz, H-5). ^{13}C{^{1}H} NMR ($CDCl_3$, 101 MHz, HSQC, HMBC): δ 137.3 (C_q), 134.1 (2CH_{arom}), 130.5 (C_q), 129.3 (2CH_{arom}), 129.0 (CH_{arom}), 128.7 (2CH_{arom}), 128.3 (CH_{arom}), 129.3 (2CH_{arom}), 97.7 (dd, ^{1}J = 189.2 Hz, ^{3}J = 0.9 Hz, C-3), 85.5 (d, ^{3}J = 7.3 Hz, C-1), 81.4 (dd, ^{1}J = 174.9, ^{4}J = 1.5 Hz, C-6), 77.2 (dd, ^{2}J = 18.5 Hz, ^{3}J = 9.7 Hz, C-5), 74.8 (d, ^{4}J = 2.9 Hz, CH_2 Bn), 74.1 (dd, ^{2}J = 17.2 Hz, ^{3}J = 7.0 Hz, C-4), 63.3 (d, ^{2}J = 18.0 Hz, C-2). ^{19}F NMR ($CDCl_3$, 376 MHz): –184.65 (ddd, ^{2}J = 51.4 Hz, ^{3}J = 13.0, 12.5 Hz, F-3), –235.47 (ddd, ^{2}J = 47.6, 46.9 Hz, ^{3}J = 26.2 Hz, F-6). HRMS-APCI (*m/z*): $[M + H]^+$ calcd for $C_{19}H_{20}F_2N_3O_2S$, 392.1232; found, 392.1231.

Resolved signals for α-**33**: ^{1}H NMR ($CDCl_3$, 400 MHz, ^{1}H {^{19}F}, H-H COSY): δ 5.62 (ddd, 1H, *J* = 52.0, 4.6, 2.8 Hz, H-1), 4.98 (ddd, 1H, *J* = 52.8, 9.9, 8.4 Hz, H-3), 4.11 (ddd, 1H, *J* = 10.0, 5.9, 2.6 Hz, H-5), 3.82 (ddd, 1H, *J* = 12.7, 10.0, 8.4 Hz, H-4), 3.58 (m, 1H, H-2), 3.13 (dd, 1H, *J* = 14.1, 5.9 Hz, H-6). ^{13}C{^{1}H} NMR ($CDCl_3$, 101 MHz, HSQC, HMBC): δ 93.7 (d, ^{1}J = 174.0 Hz, C-1), 35.6 (C-6). ^{19}F NMR ($CDCl_3$, 376 MHz): –146.82 (dd, ^{2}J = 52.0 Hz, ^{3}J = 25.3 Hz, F-1), –193.87 (dddd, ^{2}J = 52.8 Hz, ^{3}J = 12.7, 11.1 Hz, ^{4}J = 4.6 Hz, F-3).

Resolved signals for β-**33**: ^{1}H NMR ($CDCl_3$, 400 MHz, ^{1}H {^{19}F}, H-H COSY): δ 5.03 (dd, 1H, *J* = 51.5, 7.4 Hz, H-1), 3.58 (m, 1H, H-5), 3.07 (dd, 1H, *J* = 14.2, 6.9 Hz, H-6). ^{13}C{^{1}H} NMR ($CDCl_3$, 101 MHz, HSQC, HMBC): δ 35.5 (C-6). ^{19}F NMR ($CDCl_3$, 376 MHz): –141.80 (ddd, ^{2}J = 51.5 Hz, ^{3}J = 13.0, 3.1 Hz, F-1), –189.64 (dt, ^{2}J = 51.4 Hz, ^{3}J = 13.4 Hz, F-3).

Phenyl 2-azido-2,3,4,6-tetradeoxy-3,4,6-trifluoro-1-thio-α/β-D-galactopyranoside (34)

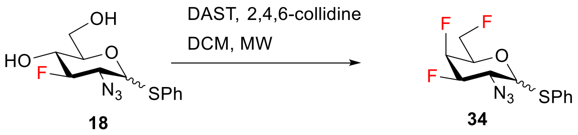

Thioglycoside **18** (750 mg, 2.51 mmol) was subjected to reaction with diethylaminosulfur trifluoride (1000 µL, 7.57 mmol) and 2,4,6-collidine (1630 µL, 12.33 mmol) in dichloromethane (15 mL) according to the general procedure. Chromatography of the crude product in EtOAc/PE 1:6 first afforded phenyl 2-azido-2,3,4,6-tetradeoxy-3,4,6-trifluoro-1-thio-α-D-galactopyranoside (α-**34**) (570 mg, 75%) as a colorless syrup followed by phenyl 2-azido-2,3,4,6-tetradeoxy-3,4,6-trifluoro-1-thio-β-D-galactopyranoside (β-**34**) (135 mg, 18%).

Data for α-**34**: R_f 0.38 (EtOAc/PE 1:5). ^{1}H NMR ($CDCl_3$, 400 MHz, ^{1}H {^{19}F}, H-H COSY): δ 7.50–7.48 (m, 2H, CH_{arom}), 7.36–7.33 (m, 3H, CH_{arom}), 5.66 (dd, 1H, J = 5.6, 4.4 Hz, H-1), 5.06 (ddd, 1H, J = 51.0, 7.4, 2.8 Hz, H-4), 4.77 (dddd, 1H, J = 47.0, 25.2, 10.5, 2.8 Hz, H-3), 4.74–4.47 (m, 3H, H-5, 2H-6), 4.42 (dddd, 1H, J = 11.0, 10.5, 5.6, 0.9 Hz, H-2). ^{13}C{^{1}H} NMR ($CDCl_3$, 101 MHz, HSQC): δ 132.8 (2CH_{arom}), 132.0 (C_q), 129.5 (2CH_{arom}), 128.6 (CH_{arom}), 88.4 (ddd, ^1J = 193.7 Hz, ^2J = 17.9 Hz, ^4J = 1.0 Hz, C-3), 86.9 (d, ^3J = 7.4 Hz, C-1), 85.8 (ddd, ^1J = 187.0 Hz, ^2J = 16.6 Hz, ^3J = 5.4 Hz, C-4), 80.2 (ddd, ^1J = 170.8 Hz, ^3J = 6.2 Hz, ^4J = 2.5 Hz, C-6), 68.3 (ddd, ^2J = 25.0, 18.0 Hz, ^3J = 5.3 Hz, C-5), 59.0 (dd, ^2J = 18.3 Hz, ^3J = 1.8 Hz, C-2). ^{19}F NMR ($CDCl_3$, 376 MHz): from ^{19}F {^{1}H} –199.81 (dd, ^3J = 14.4 Hz, ^5J = 1.9 Hz, F-3), –218.86 (dddd, ^2J = 51.0 Hz, ^3J = 28.5, 25.2, 14.4 Hz, F-4), –232.61 (ddd, ^2J = 45.9, 45.4 Hz, ^3J = 10.7 Hz, F-6). HRMS-APCI (*m/z*): $[M - N_2 + H]^+$ calcd for $C_{12}H_{13}F_3NOS$, 276.0664; found, 276.0660. Data for β-**34:** R_f 0.34 (EtOAc/PE 1:5). ^{1}H NMR ($CDCl_3$, 400 MHz, ^{1}H {^{19}F}, H-H COSY): δ 7.60–7.57 (m, 2H, CH_{arom}), 7.38–7.34 (m, 3H, CH_{arom}), 4.95 (ddd, 1H, J = 50.7, 7.1, 2.7 Hz, H-4), 4.64 (dddt, 1H, J = 46.1, 9.4, 6.2, 1.1 Hz, H-6), 4.60 (ddd, 1H, J = 46.0, 9.4, 6.7 Hz, H-6´), 4.44 (dddd, 1H, J = 45.9, 26.6, 9.5, 2.7 Hz, H-3), 4.43 (dd, 1H, J = 10.1, 0.8 Hz, H-1), 3.79 (ddddd, 1H, J = 25.8, 9.8, 6.7, 6.2, 1.9 Hz, H-5), 3.78 (dddd, 1H, J = 11.0, 10.1, 9.5, 1.1 Hz, H-2). ^{13}C{^{1}H} NMR ($CDCl_3$, 101 MHz, HSQC, HMBC): δ 133.9 (2CH_{arom}), 130.4 (C_q), 129.4 (2CH_{arom}), 129.1 (CH_{arom}), 90.9 (ddd, ^1J = 195.8 Hz, ^2J = 18.0 Hz, ^4J = 0.9 Hz, C-3), 86.1 (d, ^3J = 6.5 Hz, C-1), 84.7 (ddd, ^1J = 186.5 Hz, ^2J = 16.7 Hz, ^3J = 4.7 Hz, C-4), 79.8 (ddd, ^1J = 170.9 Hz, ^3J = 5.7 Hz, ^4J = 2.6 Hz, C-6), 74.4 (ddd, ^2J = 24.3, 18.1 Hz, ^3J = 5.8 Hz, C-5), 60.2 (dd, ^2J = 18.4 Hz, ^3J = 1.0 Hz, C-2). ^{19}F NMR ($CDCl_3$, 376 MHz): –193.34 (dddd, ^2J = 49.5 Hz, ^3J = 15.7, 11.0, 7.1 Hz, F-3), –219.95 (dddd, ^2J = 50.7 Hz, ^3J = 26.6, 25.8, 15.7 Hz, F-4), –232.87 (ddd, ^2J = 46.1, 46.0 Hz, ^3J = 9.8 Hz, F-6). HRMS-APCI (*m/z*): $[M - N_2 + H]^+$ calcd for $C_{12}H_{13}F_3NOS$, 276.0664; found, 276.0660.

Phenyl 2-Azido-6-*O*-benzyl-2,3-dideoxy-3-fluoro-1-thio-α/β-D-glucopyranoside (35)

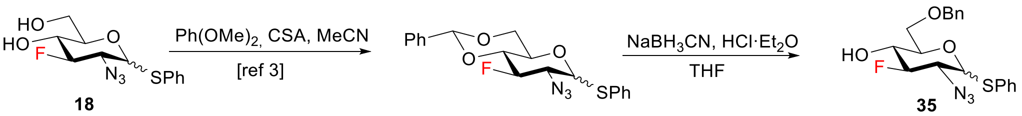

Sodium cyanoborohydride (841 mg, 13.38 mmol) and 3 Å molecular sieves (2 g) were added to a solution of phenyl 2-azido-4,6-*O*-benzylidene-2,3-dideoxy-3-fluoro-1-thio-α/β-D-glucopyranoside (525 mg, 1.36 mmol, prepared from the **18** as described in ref 3, in anhydrous THF (15 mL) under Ar atmosphere. The mixture was cooled to 0 °C and stirred for 5 min. Hydrogen chloride solution in diethyl ether (1M, 14.0 mL, 14.00 mmol) was carefully added dropwise and the reaction mixture was stirred for 15 min, filtered through a layer of celite, neutralized by saturated aqueous $NaHCO_3$, extracted with dichloromethane, the dichloromethane solution was dried and concentrated. Chromatography in EtOAc/PE 1:4 first afforded the α-anomer α-**35** (290 mg, 55%) as a colorless syrup followed by a mixture of both anomers (α/β 1:5, 145 mg, 27%) as a thick colorless syrup.

Data for α-**35**: R_f 0.29 (EtOAc/PE 1:3). ^{1}H NMR ($CDCl_3$, 400 MHz, ^{1}H {^{19}F}, H-H COSY): δ 7.50–7.48 (m, 2H, CH_{arom}), 7.36–7.27 (m, 8H, CH_{arom}), 5.58 (dt, 1H, *J* = 5.7, 3.3 Hz, H-1), 4.68 (ddd, 1H, *J* = 52.8, 10.1, 8.5 Hz, H-3), 4.61, 4.52 (2 × d, 2 × 1H, *J* = 11.9 Hz, C*H*H Bn), 4.35 (ddd, 1H, *J* = 9.8, 4.3, 3.7 Hz, H-5), 4.02 (ddd, 1H, *J* = 11.1, 10.1, 5.7 Hz, H-2), 3.94 (dddd, 1H, *J* = 14.2, 9.8, 8.5, 3.0 Hz, H-4), 3.80 (dd, 1H, *J* = 10.6, 4.3 Hz, H-6), 3.71 (ddd, 1H, *J* = 10.6, 3.7, 1.6 Hz, H-6´), 2.78 (d, 1H, *J* = 3.0 Hz, O*H*). ^{13}C{^{1}H} NMR ($CDCl_3$, 101 MHz, HSQC): δ 137.7, 132.9 (2 × C_q), 132.4, 129.3, 128.6 (3 × 2CH_{arom}), 128.11, 128.08 (2 × CH_{arom}), 127.9 (2CH_{arom}), 94.3 (d, ^{1}J = 184.8 Hz, C-3), 86.8 (d, ^{3}J = 8.0 Hz, C-1), 73.8 (*C*H_2 Bn), 70.7 (d, ^{3}J = 7.2 Hz, C-5), 70.3 (d, ^{2}J = 17.9 Hz, C-4), 69.0 (d, ^{4}J = 1.3 Hz, C-6), 62.1 (d, ^{2}J = 17.0 Hz, C-2). ^{19}F NMR ($CDCl_3$, 376 MHz): −194.35 (dddd, ^{2}J = 52.8 Hz, ^{3}J = 14.2, 11.1 Hz, ^{4}J = 3.3 Hz). HRMS-APCI (*m/z*): $[M - N_2 + H]^+$ calcd for $C_{19}H_{21}FNO_3S$, 362.1219; found, 362.1215.

Data for β-**35:** R_f 0.26 (EtOAc/PE 1:3). ^{1}H NMR ($CDCl_3$, 400 MHz, ^{1}H {^{19}F}, H-H COSY): δ 7.59–7.56 (m, 2H, CH_{arom}), 7.37–7.28 (m, 8H, CH_{arom}), 4.63, 4.57 (2 × d, 2 × 1H, *J* = 11.9 Hz, C*H*H Bn), 4.42 (d, 1H, *J* = 10.2 Hz, H-1), 4.35 (dt, 1H, *J* = 51.8, 8.9 Hz, H-3), 3.84–3.76 (m, 3H, H-4, 2H-6), 3.48–3.42 (m, 1H, H-5), from ^{1}H {^{19}F} 3.43 (dd, 1H, *J* = 10.2, 9.0 Hz, H-2), 2.84 (br s, 1H, O*H*). ^{13}C{^{1}H} NMR ($CDCl_3$, 101 MHz, HSQC): δ 137.7 (C_q), 133.8 (2CH_{arom}), 130.9 (C_q), 129.3 (2CH_{arom}), 128.8 (CH_{arom}), 128.7 (2CH_{arom}), 128.1 (CH_{arom}), 127.9 (2CH_{arom}), 96.6 (d, ^{1}J = 187.4 Hz, C-3), 85.7 (d, ^{3}J = 7.2 Hz, C-1), 77.3 (d, ^{3}J = 7.7 Hz, C-5), 73.9 (*C*H_2 Bn), 70.1 (d, ^{2}J = 17.9 Hz, C-4), 69.7 (d, ^{4}J = 1.6 Hz, C-6), 63.1 (d, ^{2}J = 17.6 Hz, C-2). ^{19}F NMR ($CDCl_3$, 376 MHz): −189.43 (dt, ^{2}J = 51.8 Hz, ^{3}J = 13.0 Hz).

Phenyl 2-azido-6-*O*-benzyl-2,3,4-trideoxy-3,4-difluoro-1-thio-α/β-D-galactopyranoside (36)

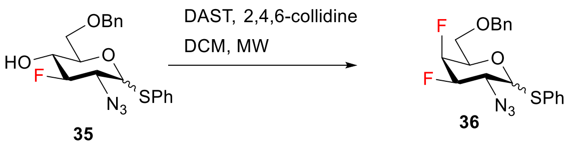

Thioglycoside **35** (400 mg, 1.03 mmol) was subjected to reaction with diethylaminosulfur trifluoride (170 µL, 1.29 mmol) and 2,4,6-collidine (340 µL, 2.57 mmol) in dichloromethane (8 mL) according to the general procedure. Chromatography of the crude product in EtOAc/PE 1:7 first afforded the phenyl 2-azido-6-*O*-benzyl-2,3,4-trideoxy-3,4-difluoro-1-thio-α-D-galactopyranoside (α-**36**) (276 mg, 69%) in ca. 90% purity (by ^{19}F NMR) as a colorless syrup followed by the phenyl 2-azido-6-*O*-benzyl-2,3,4-trideoxy-3,4-difluoro-1-thio-β-D-galactopyranoside β-**36** (63 mg, 16%) in ca 95% purity (by ^{19}F NMR) as a colorless syrup. The impurities in anomers were removed in the next step.

Data for α-**36**: R_f 0.78 (EtOAc/PE 1:3). ^{1}H NMR ($CDCl_3$, 400 MHz, ^{1}H {^{19}F}, H-H COSY): δ 7.51–7.49 (m, 2H, CH_{arom}), 7.37–7.26 (m, 8H, CH_{arom}), 5.64 (dd, 1H, *J* = 5.6, 4.3 Hz, H-1), 5.08 (ddd, 1H, *J* = 50.8, 7.6, 2.7 Hz, H-4), 4.75 (dddd, 1H, *J* = 47.0, 25.2, 10.6, 2.7 Hz, H-3), 4.63–4.52 (m, 1H, H-5), 4.54 (s, 2H, C*H*$_2$ Bn), 4.41 (dddd, 1H, *J* = 10.6, 10.0, 5.6, 0.9 Hz, H-2), 3.76 (dd, 1H, *J* = 9.7, 7.0 Hz, H-6), 3.64 (ddt, 1H, *J* = 9.7, 6.3, 1.4 Hz, H-6´). ^{13}C{^{1}H} NMR ($CDCl_3$, 101 MHz, HSQC): δ 137.7 (C_q), 132.9 (2CH_{arom}), 132.3 (C_q), 129.3, 128.6 (2 × 2CH_{arom}), 128.4, 128.0 (2 × CH_{arom}), 127.8 (2CH_{arom}), 88.7 (dd, ^1J = 192.9 Hz, ^2J = 17.7 Hz, C-3), 86.8 (d, ^3J = 7.3 Hz, C-1), 86.4 (dd, ^1J = 186.3 Hz, ^2J = 16.1 Hz, C-4), 73.7 (*C*H$_2$ Bn), 68.9 (dd, ^2J = 18.2 Hz, ^3J = 5.2 Hz, C-5), 67.4 (dd, ^3J = 5.3, 2.3 Hz, C-6), 59.2 (dd, ^2J = 18.0 Hz, ^3J = 1.8 Hz, C-2). ^{19}F NMR ($CDCl_3$, 376 MHz): −199.30 (m, F-3), −219.17 (dddd, ^2J = 50.8 Hz, ^3J = 29.0, 25.2, 15.1 Hz, F-4). HRMS-APCI (*m/z*): $[M-N_2+H]^+$ calcd for $C_{19}H_{20}F_2NO_2S$, 364.1177; found, 364.1176.

Data for β-**36:** R_f 0.74 (EtOAc/PE 1:3). ^{1}H NMR ($CDCl_3$, 400 MHz, ^{1}H {^{19}F}, H-H COSY): δ 7.59–7.57 (m, 2H, CH_{arom}), 7.36–7.31 (m, 8H, CH_{arom}), 4.99 (ddd, 1H, *J* = 50.4, 7.3, 2.7 Hz, H-4), 4.57 (s, 2H, C*H*$_2$ Bn), 4.40 (dt, 1H, *J* = 10.1, 0.9 Hz, H-1), 4.39 (dddd, 1H, *J* = 46.3, 26.7, 9.7, 2.7 Hz, H-3), 3.82–3.68 (m, 3H, H-2, 2H-6), 3.62 (dtd, 1H, *J* = 25.6, 6.7, 2.0 Hz, H-5). ^{13}C{^{1}H} NMR ($CDCl_3$, 101 MHz, HSQC): δ 137.6 (C_q), 133.7 (2CH_{arom}), 130.8 (C_q), 129.3 (2CH_{arom}), 128.9 (CH_{arom}), 128.7 (2CH_{arom}), 128.1 (CH_{arom}), 127.9 (2CH_{arom}), 91.3 (dd, ^1J = 195.2 Hz, ^2J = 18.2 Hz, C-3), 86.0 (d, ^3J = 6.5 Hz, C-1), 85.2 (dd, ^1J = 185.8 Hz, ^2J = 16.4 Hz, C-4), 75.4 (dd, ^2J = 18.3 Hz, ^3J = 5.5 Hz, C-5), 73.9 (*C*H$_2$ Bn), 67.2 (dd, ^3J = 4.9, 2.4 Hz, C-6), 60.4 (dd, ^2J = 18.4 Hz, ^3J = 0.7 Hz, C-2). ^{19}F NMR ($CDCl_3$, 376 MHz): −192.91 (dddd, ^2J = 46.3 Hz, ^3J = 16.5, 9.7, 7.3 Hz, F-3), −220.27 (dddd, ^2J = 50.4 Hz, ^3J = 26.7, 25.6, 16.5 Hz, F-4). HRMS-APCI (*m/z*): $[M-N_2+H]^+$ calcd for $C_{19}H_{20}F_2NO_2S$, 364.1177; found, 364.1176.

2-Azido-4-*O*-benzyl-2,3,6-trideoxy-3,6-difluoro-D-glucopyranose (37)

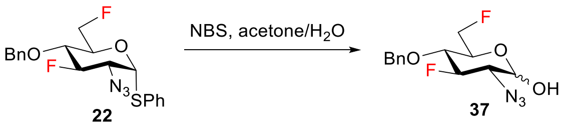

Thioglycoside **22** (200 mg, 0.51 mmol) was hydrolyzed according to the general procedure. Chromatography in EtOAc/PE 1:4 afforded **37** (129 mg, 85%) as a colorless gel, R_f 0.43 (EtOAc/PE 1:3). NMR data for the α-anomer: ^{1}H NMR ($CDCl_3$, 400 MHz, ^{1}H {^{19}F}, H-H COSY): δ 7.39–7.30 (m, 5H, CH_{arom}), 5.35 (q, 1H, J = 3.7 Hz, H-1), 5.05 (dddd, 1H, J = 53.3, 9.6, 8.3, 0.9 Hz, H-3), 4.92 (dd, 1H, J = 11.2, 1.4 Hz, C*H*H Bn), 4.66 (ddd, 1H, J = 47.3, 10.3, 3.2 Hz, H-6´), 4.65 (d, 1H, J = 11.2 Hz, C*H*H Bn), 4.57 (dddd, 1H, J = 48.1, 10.3, 1.9, 1.5 Hz, H-6), 3.99 (ddt, 1H, J = 28.3, 10.0, 1.5 Hz, H-5), 3.76 (ddd, 1H, J = 13.4, 10.0, 8.3 Hz, H-4), 3.54–7.46 (m, 1H, H-2), 2.99 (dd, 1H, J = 3.7, 1.3 Hz, O*H*). ^{13}C{^{1}H} NMR ($CDCl_3$, 101 MHz, HSQC, HMBC): δ 137.4 (C_q), 128.7 (2CH_{arom}), 128.4 (CH_{arom}), 128.3 (2CH_{arom}), 94.1 (dd, ^1J = 184.4 Hz, ^4J = 1.0 Hz, C-3), 92.5 (d, ^3J = 9.7 Hz, C-1), 81.6 (dd, ^1J = 173.7, ^4J = 1.2 Hz, C-6), 74.91 (d, ^4J = 2.9 Hz, CH_2 Bn), 74.85 (dd, ^2J = 17.1 Hz, ^3J = 6.9 Hz, C-4), 69.7 (dd, ^2J = 18.0 Hz, ^3J = 8.8 Hz, C-5), 62.2 (d, ^2J = 16.9 Hz, C-2). ^{19}F NMR ($CDCl_3$, 376 MHz): −194.27 (dddd, ^2J = 53.3 Hz, ^3J = 13.4, 10.6 Hz, ^4J = 3.7 Hz, F-3), −236.44 (dddd, ^2J = 48.1, 47.3 Hz, ^3J = 28.3 Hz, J = 3.2 Hz, F-6). NMR data for the β-anomer: ^{1}H NMR ($CDCl_3$, 400 MHz, ^{1}H {^{19}F}, H-H COSY): δ 7.39–7.30 (m, 5H, CH_{arom}), 4.89 (dd, 1H, J = 11.2, 0.7 Hz, CH*H* Bn), 4.70–7.53 (m, 2H, H-6), 6.62 (d, 1H, J = 8.5 Hz, H-1), 4.63 (d, 1H, J = 11.2 Hz, C*H*H Bn), 4.50 (dddd, 1H, J = 51.1, 9.9, 8.4, 0.8 Hz, H-3), 3.72 (ddd, 1H, J = 12.9, 10.0, 8.4 Hz, H-4), 3.54–3.46 (m, 2H, H-2, H-5), 2.99 (dd, 1H, J = 3.7, 1.3 Hz, O*H*). ^{13}C{^{1}H} NMR ($CDCl_3$, 101 MHz, HSQC, HMBC): δ 137.2 (C_q), 128.7 (2CH_{arom}), 128.4 (CH_{arom}), 128.3 (2CH_{arom}), 96.0 (dd, ^1J = 187.8 Hz, ^4J = 1.1 Hz, C-3), 95.7 (d, ^3J = 10.9 Hz, C-1), 81.4 (dd, ^1J = 174.3, ^4J = 1.8 Hz, C-6), 74.8 (d, ^4J = 2.7 Hz, CH_2 Bn), 74.3 (dd, ^2J = 17.2 Hz, ^3J = 7.0 Hz, C-4), 73.2 (dd, ^2J = 18.7 Hz, ^3J = 9.9 Hz, C-5), 65.6 (d, ^2J = 17.2 Hz, C-2). ^{19}F NMR ($CDCl_3$, 376 MHz): −188.75 (ddd, ^2J = 51.1 Hz, ^3J = 13.3, 12.9 Hz, F-3), −235.25 (ddd, ^2J = 47.6, 47.4 Hz, ^3J = 25.4 Hz, F-6). HRMS-APCI (*m/z*): $[M - N_2 + H]^+$ calcd for $C_{13}H_{16}F_2NO_3$, 272.1092; found, 272.1091.

2-Azido-3-*O*-benzyl-2,4,6-trideoxy-4,6-difluoro-D-glucopyranose (38)

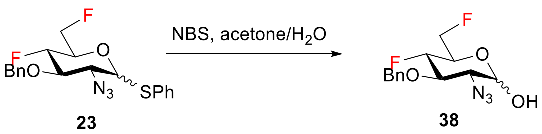

Thioglycoside **23** (350 mg, 0.89 mmol) was hydrolyzed according to the general procedure. Chromatography in EtOAc/PE 1:4 afforded **38** (201 mg, 75%) as a white crystalline solid, mp 97–102 °C (EtOAc/heptane). R_f 0.38 (EtOAc/PE 1:3). NMR data for the α-anomer: ^{1}H NMR ($CDCl_3$, 400 MHz, ^{1}H {^{19}F}, H-H COSY): δ 7.43–7.31 (m, 5H, CH_{arom}), 5.31 (ddd, 1H, J = 3.6, 3.4, 3.2 Hz, H-1), 4.92 (dd, 1H, J = 10.9, 1.2 Hz, C*H*H Bn), 4.80 (d, 1H, J = 10.9 Hz, C*H*H Bn), 4.65 (dddd, 1H, J = 47.0, 10.5, 3.5, 1.7 Hz, H-6´), 4.61 (ddt, 1H, J = 47.3, 10.5, 1.9 Hz, H-6), 4.59

(ddd, 1H, J = 50.5, 10.1, 8.4 Hz, H-4), 4.18 (ddddd, 1H, J = 26.3, 10.1, 4.9, 3.5, 1.9 Hz, H-5), 4.11 (ddd, 1H, J = 14.1, 10.1, 8.4 Hz, H-3), 3.43 (ddd, 1H, J = 10.1, 3.4, 1.3 Hz, H-2), 3.00 (dd, 1H, J = 3.6, 1.3 Hz, OH). ^{13}C{^{1}H} NMR ($CDCl_3$, 101 MHz, HSQC): δ 137.5 (C_q), 128.7, 128.4 (2 × 2CH_{arom}), 128.3 (CH_{arom}), 92.0 (d, ^{4}J = 1.5 Hz, C-1), 89.6 (dd, ^{1}J = 184.6 Hz, ^{3}J = 7.0 Hz, C-4), 81.1 (d, ^{1}J = 174.6 Hz, C-6), 77.3 (dd, ^{2}J = 17.8 Hz, ^{4}J = 0.8 Hz, C-3), 75.1 (d, ^{4}J = 2.9 Hz, CH$_2$ Bn), 68.6 (dd, ^{2}J = 24.6, 18.3 Hz, C-5), 62.7 (d, ^{3}J = 8.4 Hz, C-2). ^{19}F NMR ($CDCl_3$, 376 MHz): –196.09 (dddd, ^{2}J = 50.5 Hz, ^{3}J = 14.1, 4.9 Hz, ^{5}J = 3.2 Hz, F-4), –237.14 (ddd, ^{2}J = 47.3, 47.0 Hz, ^{3}J = 26.3 Hz, F-6). HRMS-APCI (*m/z*): $[M - N_2 + H]^+$ calcd for $C_{13}H_{16}F_2NO_3$, 272.1092; found, 272.1091. NMR signals of the β-anomer could not be observed in intensity sufficient for characterization within 12 h after sample dissolution.

2-Azido-4-*O*-benzyl-2,3,6-trideoxy-3,6-difluoro-D-galactopyranose (39)

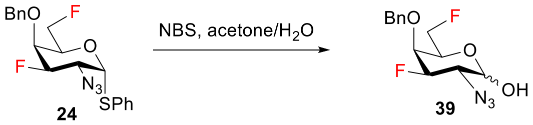

Thioglycoside **24** (184 mg, 0.47 mmol) was hydrolyzed according to the general procedure. Chromatography in EtOAc/PE 1:4 afforded **39** (126 mg, 90%) as a white crystalline solid, mp 115–119 °C (Et_2O/heptane). R_f 0.40 (EtOAc/PE 1:3). NMR data for the α-anomer: ^{1}H NMR ($CDCl_3$, 400 MHz, ^{1}H {^{19}F}, H-H COSY): δ 7.40–7.31 (m, 5H, CH_{arom}), 5.39 (q, 1H, J = 3.8 Hz, H-1), 5.04 (ddd, 1H, J = 48.9, 10.4, 3.1 Hz, H-3), 4.91, 4.58 (2 × d, 2 × 1H, J = 11.4 Hz, CHH Bn), 4.60–4.44 (m, 1H, H-6), from ^{1}H {^{19}F-6} 4.66 (dd, 1H, J = 13.3, 5.6 Hz, H-6´), from ^{1}H {^{19}F-6} 4.28 (dddd, 1H, J = 7.1, 5.6, 2.0, 1.4 Hz, H-5), 4.08 (ddd, 1H, J = 6.7, 3.1, 1.4 Hz, H-4), 4.00 (ddd, 1H, J = 10.4, 10.1, 3.8 Hz, H-2), 2.96 (m, 1H, OH). ^{13}C{^{1}H} NMR ($CDCl_3$, 101 MHz, HSQC, HMBC): δ 137.4 (C_q), 128.7 (2CH_{arom}), 128.61 (CH_{arom}), 128.55 (2CH_{arom}), 92.9 (d, ^{3}J = 9.5 Hz, C-1), 90.4 (dd, ^{1}J = 188.3 Hz, ^{4}J = 1.4 Hz, C-3), 81.9 (dd, ^{1}J = 168.5, ^{4}J = 2.8 Hz, C-6), 75.3 (d, ^{4}J = 4.2 Hz, CH$_2$ Bn), 73.8 (dd, ^{2}J = 15.6 Hz, ^{3}J = 5.7 Hz, C-4), 68.8 (dd, ^{2}J = 23.8 Hz, ^{3}J = 7.0 Hz, C-5), 59.4 (d, ^{2}J = 17.2 Hz, C-2). ^{19}F NMR ($CDCl_3$, 376 MHz): −201.03 (dddd, ^{2}J = 48.9 Hz, ^{3}J = 10.1, 6.7 Hz, ^{4}J = 3.8 Hz, F-3), −231.69 (m, F-6). NMR data for the β-anomer: ^{1}H NMR ($CDCl_3$, 400 MHz, ^{1}H {^{19}F}, H-H COSY): δ 7.40–7.31 (m, 5H, CH_{arom}), 4.91, 4.59 (2 × d, 2 × 1H, J = 11.4, CHH Bn), 4.64–4.47 (m, 2H, H-6), 4.57 (dd, 1H, J = 8.0, 1.1 Hz, H-1), 4.43 (ddd, 1H, J = 47.5, 10.2, 3.3 Hz, H-3), from ^{1}H {^{19}F-3} 3.96 (dd, 1H, J = 3.1, 1.6 Hz, H-4), 3.89 (ddd, 1H, J = 11.2, 10.2, 8.0 Hz, H-2), 3.69 (dtt, 1H, J = 12.2, 6.1, 1.6 Hz, H-5), 3.46 (br s, 1H, OH). ^{13}C{^{1}H} NMR ($CDCl_3$, 101 MHz, HSQC, HMBC): δ 137.2 (C_q), 128.7 (2CH_{arom}), 128.41 (CH_{arom}), 128.37 (2CH_{arom}), 96.2 (d, ^{3}J = 10.3 Hz, C-1), 92.8 (dd, ^{1}J = 191.7 Hz, ^{4}J = 1.3 Hz, C-3), 81.3 (dd, ^{1}J = 169.2, ^{4}J = 3.2 Hz, C-6), 75.1 (d, ^{4}J = 5.0 Hz, CH$_2$ Bn), 72.4 (dd, ^{2}J = 15.6 Hz, ^{3}J = 5.4 Hz, C-4), 72.1 (dd, ^{2}J = 24.0 Hz, ^{3}J = 8.1 Hz, C-5), 63.6 (d, ^{2}J = 17.2 Hz, C-2). ^{19}F NMR ($CDCl_3$, 376 MHz): −195.67 (m, F-3), −231.69 (m, F-6). HRMS-APCI (*m/z*): $[M - N_2 + H]^+$ calcd for $C_{13}H_{16}F_2NO_3$, 272.1092; found, 272.1091.

2-Azido-3-*O*-benzyl-2,4,6-trideoxy-4,6-difluoro-D-galactopyranose (40)

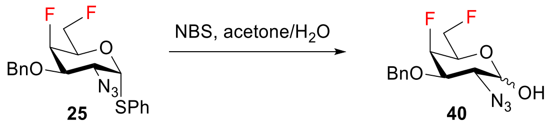

Thioglycoside **25** (175 mg, 0.45 mmol) was hydrolyzed according to the general procedure. Chromatography in EtOAc/PE 1:3 afforded **40** (90 mg, 67%) as a white crystalline solid, mp 89–92 °C (MTBE/heptane). R_f 0.36 (EtOAc/PE 1:3). NMR data for the α-anomer: ^{1}H NMR ($CDCl_3$, 400 MHz, ^{1}H {^{19}F}, H-H COSY): δ 7.43–7.31 (m, 5H, CH_{arom}), 5.37 (d, 1H, J = 3.4 Hz, H-1), 4.87 (dd, 1H, J = 50.2, 2.5 Hz, H-4), 4.78, 4.74 (2 × d, 2 × 1H, J = 11.7 Hz, C*H*H Bn), 4.58 (ddd, 1H, J = 46.1, 9.5, 6.1 Hz, H-6´), 4.53 (dddd, 1H, J = 46.5, 9.5, 6.5, 1.0 Hz, H-6), 4.28 (dddd, 1H, J = 29.6, 12.4, 6.5, 6.1 Hz, H-5), 3.96 (ddd, 1H, J = 26.7, 10.5, 2.5 Hz, H-3), 3.83 (ddd, 1H, J = 10.5, 3.4, 1.0 Hz, H-2). ^{13}C{^{1}H} NMR ($CDCl_3$, 101 MHz, HSQC): δ 137.1 (C_q), 128.8 (2CH_{arom}), 128.4 (CH_{arom}), 128.1 (2CH_{arom}), 92.4 (C-1), 85.2 (dd, ^1J = 185.6 Hz, ^3J = 5.1 Hz, C-4), 81.1 (dd, ^1J = 169.4 Hz, ^3J = 6.5 Hz, C-6), 74.1 (dd, ^2J = 18.0 Hz, ^4J = 0.9 Hz, C-3), 72.0 (CH_2 Bn), 68.0 (dd, ^2J = 23.9, 18.0 Hz, C-5), 59.6 (d, ^3J = 2.3 Hz, C-2). ^{19}F NMR ($CDCl_3$, 376 MHz): –220.98 (ddd, ^2J = 50.2 Hz, ^3J = 29.6, 26.7 Hz, F-4), –232.58 (ddd, ^2J = 46.5, 46.1 Hz, ^3J = 12.4 Hz, F-6). NMR data for the β-anomer: ^{1}H NMR ($CDCl_3$, 400 MHz, ^{1}H {^{19}F}, H-H COSY): δ 7.43–7.31 (m, 5H, CH_{arom}), 4.78 (d, 1H, J = 11.9 Hz, C*H*H Bn), 4.75 (dd, 1H, J = 49.7, 2.6 Hz, H-4), 4.73 (d, 1H, J = 11.9 Hz, C*H*H Bn), 4.58 (dd, 1H, J = 8.1, 1.4 Hz, H-1), overlapped with α-anomer (2H, H-6), 3.72 (ddt, 1H, J = 26.7, 10.6, 6.3 Hz, H-5), 3.68 (dd, 1H, J = 10.3, 8.1 Hz, H-2), 3.38 (ddd, 1H, J = 27.3, 10.3, 2.6 Hz, H-3). ^{13}C{^{1}H} NMR ($CDCl_3$, 101 MHz, HSQC): δ 137.1 (C_q), 128.8 (2CH_{arom}), 128.4 (CH_{arom}), 128.1 (2CH_{arom}), 96.3 (C-1), 84.0 (dd, ^1J = 185.5 Hz, ^3J = 3.5 Hz, C-4), 80.5 (dd, ^1J = 170.1 Hz, ^3J = 6.0 Hz, C-6), overlapped with $CDCl_3$ (C-3), 72.2 (CH_2 Bn), 72.0 (dd, ^2J = 23.0, 18.5 Hz, C-5), 63.8 (d, ^3J = 0.8 Hz, C-2). ^{19}F NMR ($CDCl_3$, 376 MHz): –218.59 (ddd, ^2J = 49.7 Hz, ^3J = 27.3, 26.7 Hz, F-4), –232.46 (td, ^2J = 46.5 Hz, ^3J = 10.6 Hz, F-6). HRMS-APCI (*m/z*): $[M - N_2 + H]^+$ calcd for $C_{13}H_{16}F_2NO_3$, 272.1092; found, 272.1094.

2-Azido-2,3,4,6-tetradeoxy-3,4,6-trifluoro-D-glucopyranose (41)

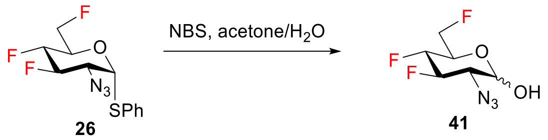

Thioglycoside **26** (330 mg, 1.09 mmol) was hydrolyzed according to the general procedure. Chromatography in EtOAc/PE 1:4 afforded **41** (209 mg, 91%) as a colorless gel. R_f 0.32 (EtOAc/PE 1:3). NMR data for the α-anomer: ^{1}H NMR ($CDCl_3$, 400 MHz, ^{1}H {^{19}F}, H-H COSY): δ 5.37 (dt, 1H, J = 3.6, 3.4 Hz, H-1), 5.05 (dddd, 1H, J = 53.4, 15.4, 10.0, 8.2 Hz, H-3), 4.77–4.53 (m, 3H, H-4, 2H-6), 4.19 (ddddd, 1H, J = 26.6, 10.1, 5.4, 2.7, 2.3 Hz, H-5), from ^{1}H {^{19}F-3} 3.52 (dd, 1H, J = 10.0, 3.6 Hz, H-2), 3.16 (br s, 1H, O*H*). ^{13}C{^{1}H} NMR ($CDCl_3$, 101 MHz, HSQC, HMBC): δ 92.3 (dd, ^3J = 9.3 Hz, ^4J = 1.4 Hz, C-1), 90.4 (ddd, ^1J = 187.0 Hz, ^2J = 19.4 Hz, ^4J = 0.6 Hz, C-3), 86.1

(ddd, ^{1}J = 187.1 Hz, ^{2}J = 18.6 Hz, ^{3}J = 7.7 Hz, C-4), 80.7 (dt, ^{1}J = 175.2 Hz, $^{3,4}J$ = 0.9 Hz, C-6), 68.1 (ddd, ^{2}J = 23.9, 18.2 Hz, ^{3}J = 7.0 Hz, C-5), 61.7 (dd, ^{2}J = 16.9 Hz, ^{3}J = 6.6 Hz, C-2). ^{19}F NMR ($CDCl_3$, 376 MHz): –198.62 (ddddd, ^{2}J = 53.4 Hz, ^{3}J = 13.7, 13.1, 11.0 Hz, ^{4}J = 3.4 Hz, F-3), –199.95 (ddddd, ^{2}J = 51.1 Hz, ^{3}J = 15.4, 13.1, 2.7 Hz, ^{5}J = 3.4 Hz, F-4), –237.44 (td, ^{2}J = 47.2 Hz, ^{3}J = 26.7 Hz, F-6). Resolved signals for the β-anomer: ^{1}H NMR ($CDCl_3$, 400 MHz, ^{1}H {^{19}F}, H-H COSY): δ 4.77–4.53 (m, 4H, H-1, H-4, 2H-6), 4.52 (ddddd, 1H, *J* = 51.5, 15.9, 9.7, 8.3, 0.8 Hz, H-3), 3.69 (br s, 1H, O*H*), from ^{1}H {^{19}F-4} 3.64 (dddd, 1H, *J* = 23.5, 9.9, 4.7, 2.3 Hz, H-5), from ^{1}H {^{19}F-3} 3.54 (dd, 1H, *J* = 9.7, 8.0 Hz, H-2). ^{13}C{^{1}H} NMR ($CDCl_3$, 101 MHz, HSQC, HMBC): δ 95.8 (dd, ^{3}J = 10.2 Hz, ^{4}J = 1.4 Hz, C-1), 92.0 (ddd, ^{1}J = 190.3 Hz, ^{2}J = 19.9 Hz, ^{4}J = 0.8 Hz, C-3), 85.7 (ddd, ^{1}J = 187.0 Hz, ^{2}J = 19.1 Hz, ^{3}J = 7.7 Hz, C-4), 80.6 (ddd, ^{1}J = 175.8 Hz, ^{3}J = 1.9 Hz, ^{4}J = 0.4 Hz, C-6), 71.6 (ddd, ^{2}J = 23.9, 18.8 Hz, ^{3}J = 7.7 Hz, C-5), 65.1 (dd, ^{2}J = 17.4 Hz, ^{3}J = 7.7 Hz, C-2). ^{19}F NMR ($CDCl_3$, 376 MHz): –192.87 (ddt, ^{2}J = 51.5 Hz, ^{3}J = 13.6, 13.4 Hz, F-3), from ^{19}F {^{1}H} –201.89 (d, ^{3}J = 13.6 Hz, F-4), –236.13 (ddd, ^{2}J = 47.0, 47.3 Hz, ^{3}J = 23.5 Hz, F-6). HRMS-APCI (*m/z*): $[M - N_2 + H]^+$ calcd for $C_6H_9F_3NO_2$, 184.0579; found, 184.0576.

2-Azido-2,3,4,6-tetradeoxy-3,4,6-trifluoro-D-galactopyranose (42)

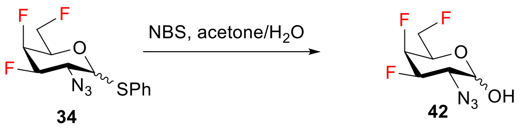

Thioglycoside **34** (250 mg, 0.82 mmol) was hydrolyzed according to the general procedure. Chromatography in EtOAc/PE 1:3 afforded **42** (140 mg, 80%) as a colorless gel. R_f 0.29 (EtOAc/PE 1:3). NMR data for the α-anomer: ^{1}H NMR ($CDCl_3$, 400 MHz, ^{1}H {^{19}F}, H-H COSY): δ 5.43 (dt, 1H, *J* = 4.8, 3.5 Hz, H-1), 5.04 (ddd, 1H, *J* = 51.9, 7.6, 2.7 Hz, H-4), 4.97 (dddd, 1H, *J* = 47.4, 25.8, 10.3, 2.7 Hz, H-3), from ^{1}H {^{19}F-6} 4.62 (dd, 1H, *J* = 9.5, 6.1 Hz, H-6´), from ^{1}H {^{19}F-6} 4.57 (ddd, 1H, *J* = 9.5, 6.5, 0.9 Hz, H-6), from ^{1}H {^{19}F-6} 4.35 (dd, 1H, *J* = 6.5, 6.1 Hz, H-5), 3.90 (dddd, 1H, *J* = 10.8, 10.3, 3.5, 1.3 Hz, H-2), 3.31 (dd, 1H, *J* = 3.5, 1.3 Hz, O*H*). ^{13}C{^{1}H} NMR ($CDCl_3$, 101 MHz, HSQC, HMBC): δ 92.6 (d, ^{3}J = 9.1 Hz, H-1), 87.1 (ddd, ^{1}J = 191.0 Hz, ^{2}J = 17.9 Hz, ^{4}J = 1.1 Hz, C-3), 86.1 (ddd, ^{1}J = 186.0 Hz, ^{2}J = 16.7 Hz, ^{3}J = 5.7 Hz, C-4), 80.7 (ddd, ^{1}J = 170.0 Hz, ^{3}J = 6.3 Hz, ^{4}J = 2.6 Hz, C-6), 67.7 (ddd, ^{2}J = 23.8, 18.0 Hz, ^{3}J = 5.5 Hz, C-5), 58.8 (dd, ^{2}J = 17.6 Hz, ^{3}J = 2.2 Hz, C-2). ^{19}F NMR ($CDCl_3$, 376 MHz): –204.30 (dddddd, ^{2}J = 47.4 Hz, ^{3}J = 14.5, 10.8, 7.6 Hz, ^{4}J = 4.8 Hz, ^{5}J = 1.5 Hz, F-3), –221.55 (dddd, ^{2}J = 51.9 Hz, ^{3}J = 28.8, 25.8, 14.5 Hz, F-4), –232.70 (tdd, ^{2}J = 46.4 Hz, ^{3}J = 12.0 Hz, ^{5}J = 1.5 Hz, F-6). Resolved signals for the β-anomer: ^{1}H NMR ($CDCl_3$, 400 MHz, ^{1}H {^{19}F}, H-H COSY) : δ 4.65 (ddd, 1H, *J* = 7.9, 5.1, 1.0 Hz, H-1), 4.38 (dddd, 1H, *J* = 46.0, 26.2, 10.3, 2.9 Hz, H-3), from ^{1}H {^{19}F-3} 3.84 (dd, 1H, *J* = 10.3, 7.9 Hz, H-2), 3.86–3.74 (m, 1H, H-5). ^{13}C{^{1}H} NMR ($CDCl_3$, 101 MHz, HSQC, HMBC): δ 96.0 (d, ^{3}J = 10.2 Hz, H-1), 89.3 (ddd, ^{1}J = 194.4 Hz, ^{2}J = 17.9 Hz, ^{4}J = 0.9 Hz, C-3), 84.7 (ddd, ^{1}J = 187.0 Hz, ^{2}J = 16.7 Hz, ^{3}J = 5.3 Hz, C-4), 80.3 (ddd, ^{1}J = 170.9 Hz, ^{3}J = 5.8 Hz, ^{4}J = 2.9 Hz, C-6), 71.0 (ddd, ^{2}J = 24.3, 18.1 Hz, ^{3}J = 6.3 Hz, C-5), 62.8 (dd, ^{2}J = 18.1 Hz, ^{3}J = 0.9 Hz, C-2). ^{19}F NMR ($CDCl_3$, 376 MHz): –198.76 (ddddd, ^{2}J = 46.0 Hz, ^{3}J = 15.3, 11.2, 6.5 Hz, ^{5}J = 1.3 Hz, F-3), –219.09 (dtd,

^2J = 51.5 Hz, ^3J = 26.2, 15.3 Hz, F-4), –232.64 (tdd, ^2J = 46.4 Hz, ^3J = 11.2 Hz, ^5J = 1.3 Hz, F-6). HRMS-APCI (*m/z*):[M − N_2 + H]$^+$ calcd for $C_6H_9F_3NO_2$, 184.0579; found, 184.0574.

2-Azido-4,6-di-*O*-benzyl-2,3-dideoxy-3-fluoro-D-glucopyranose (43)

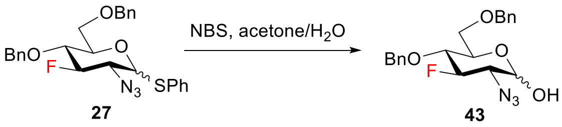

Thioglycoside **27** (350 mg, 0.73 mmol), was hydrolyzed according to the general procedure. Chromatography in EtOAc/PE 1:3 afforded **43** (258 mg, 91%) as a colorless crystalline material, mp 63–65 °C (MTBE/heptane), R_f 0.2 (EtOAc/PE 1:3). NMR data for the α-anomer: ^{1}H NMR ($CDCl_3$, 400 MHz, ^{1}H{^{19}F}, H-H COSY): δ 7.37–7.29 (m, 10H, CH_{arom}), 5.33 (q, 1H, *J* = 3.6 Hz, H-1), 5.02 (ddd, 1H, *J* = 53.5, 10.0, 8.4 Hz, H-3), 4.84 (dd, 1H, *J* = 11.0, 1.0 Hz, CH*H* O-4Bn), 4.59 (d, 1H, *J* = 12.0 Hz, CH*H* O-6Bn), 4.52 (d, 1H, *J* = 11.0 Hz, CH*H* O-4Bn), 4.50 (d, 1H, *J* = 12.0 Hz, CH*H* O-6Bn), 4.05 (ddd, 1H, *J* = 10.0, 4.2, 2.4 Hz, H-5), from ^{1}H{^{19}F} 3.73 (dd, 1H, *J* = 10.0, 8.4 Hz, H-4), 3.73–3.60 (m, 2H, H-6), 3.52–3.43 (m, 1H, H-2), 3.15 (dd, 1H, *J* = 3.6, 1.4 Hz, O*H*). ^{13}C{^{1}H} NMR ($CDCl_3$, 101 MHz, HSQC): δ 137.73, 137.66 (2 × C_q), 128.62, 128.58, 128.24, 128.17 (4 × 2CH_{arom}), 128.12, 128.06 (2 × CH_{arom}), 94.3 (d, ^1J = 183.9 Hz, C-3), 92.4 (d, ^3J = 9.7 Hz, C-1), 76.0 (d, ^2J = 16.5 Hz, C-4), 74.7 (d, ^4J = 2.8 Hz, *C*H_2 O-4Bn), 73.7 (*C*H_2 O-6Bn), 69.9 (d, ^3J = 8.3 Hz, C-5), 68.4 (d, ^4J = 0.9 Hz, C-6), 62.2 (d, ^2J = 16.7 Hz, C-2). ^{19}F NMR ($CDCl_3$ 376 MHz): δ −192.87 (dt, ^2J = 53.8 Hz, ^3J = 13.3 Hz Hz). Resolved signals for the β-anomer: ^{1}H NMR ($CDCl_3$, 400 MHz, ^{1}H{^{19}F}, H-H COSY, HSQC): δ 7.37–7.29 (m, 10H, CH_{arom}), 4.80 (dd, 1H, *J* = 11.0, 1.0 Hz, CH*H* O-4Bn), 4.55 (dd, 1H, *J* = 7.8, 4.5 Hz, H-1), 4.59 (d, 1H, *J* = 12.0 Hz, CH*H* O-6Bn), 4.43 (ddd, 1H, *J* = 51.4, 9.7, 8.4 Hz, H-3), 3.77–3.60 (m, 4H, O*H*, H-4, 2H-6), 3.52–3.43 (m, 2H, H-2, H-5). ^{13}C{^{1}H} NMR ($CDCl_3$, 101 MHz, HSQC): δ 137.6, 137.5 (2 × C_q), 128.63, 128.60, 128.27 (3 × 2CH_{arom}), 128.20 (CH_{arom}), 128.18 (2CH_{arom}), 128.1 (CH_{arom}), 96.2 (d, ^1J = 186.8 Hz, C-3), 95.7 (d, ^3J = 10.8 Hz, C-1), 75.5 (d, ^2J = 16.9 Hz, C-4), 74.6 (d, ^4J = 2.8 Hz, *C*H_2 O-4Bn), 73.8 (*C*H_2 O-6Bn), 68.5 (d, ^4J = 1.8 Hz, C-6), 65.7 (d, ^2J = 16.7 Hz, C-2). ^{19}F NMR ($CDCl_3$ 376 MHz): δ −187.45 (dt, ^2J = 51.4 Hz, ^3J = 13.3 Hz Hz). HRMS-APCI (*m/z*): [M − N_2 + H]$^+$ calcd for $C_{20}H_{23}FNO_4$, 360.1606; found, 360.1609.

2-Azido-3,6-di-*O*-benzyl-2,4-dideoxy-4-fluoro-D-glucopyranose (44)

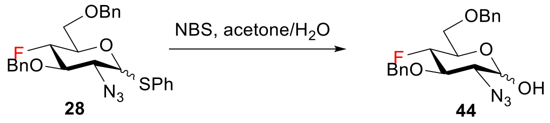

Thioglycoside **28** (240 mg, 0.50 mmol) was hydrolyzed according to the general procedure. Chromatography in EtOAc/PE 1:4 afforded **44** (176 mg, 91%) as a colorless syrup. R_f 0.57 (EtOAc/PE 1:3). NMR data for the α-anomer: ^{1}H NMR ($CDCl_3$, 400 MHz, ^{1}H {^{19}F}, H-H COSY): δ 7.43–7.29 (m, 10H, CH_{arom}), 5.28 (td, 1H, *J* = 3.6, 3.2 Hz, H-

1), 4.90 (dd, 1H, J = 11.0, 1.1 Hz, C*H*H O3-Bn), 4.78 (d, 1H, J = 11.0 Hz, C*H*H O3-Bn), 4.62, 4.58 (2 × d, 2 × 1H, J = 12.2 Hz, C*H*H O6-Bn), 4.53 (ddd, 1H, J = 50.7, 10.0, 8.4 Hz, H-4), from ^{1}H {^{19}F} 4.19 (ddd, 1H, J = 10.0, 5.3, 2.1 Hz, H-5), 4.07 (ddd, 1H, J = 14.2, 10.4, 8.4 Hz, H-3), from ^{1}H {^{19}F} 3.72 (dd, 1H, J = 10.9, 2.1 Hz, H-6´), from ^{1}H {^{19}F} 3.66 (dd, 1H, J = 10.9, 5.3 Hz, H-6), 3.39 (ddd, 1H, J = 10.4, 3.6, 1.2 Hz, H-2), 3.37 (d, 1H, J = 3.6 Hz, O*H*). ^{13}C{^{1}H} NMR ($CDCl_3$, 101 MHz, HSQC): δ 137.7, 137.6 (2 × C_q), 128.6 (4CH_{arom}), 128.4 (2CH_{arom}), 128.2, 128.00 (2 × CH_{arom}), 127.97 (2CH_{arom}), 92.0 (d, ^4J = 1.5 Hz, C-1), 90.9 (d, ^1J = 184.0 Hz, C-4), 77.5 (d, ^2J = 19.4 Hz, C-3), 75.1 (d, ^4J = 2.9 Hz, *C*H_2 O-3Bn), 73.8 (*C*H_2 O-6Bn), 68.8 (d, ^2J = 24.4 Hz, C-5), 68.4 (C-6), 62.8 (d, ^3J = 8.4 Hz, C-2). ^{19}F NMR ($CDCl_3$, 376 MHz): δ −195.45 (dd, ^2J = 50.7 Hz, ^3J = 14.2 Hz). NMR data for the β-anomer: ^{1}H NMR ($CDCl_3$, 400 MHz, ^{1}H {^{19}F}, H-H COSY): δ 7.43–7.29 (m, 10H, CH_{arom}), 4.85 (dd, 1H, J = 11.1, 1.0 Hz, C*H*H O3-Bn), 4.76 (d, 1H, J = 11.1 Hz, C*H*H O3-Bn), 4.59 (2 × d, 2 × 1H, J = 12.2 Hz, C*H*H O6-Bn), 4.56 (d, 1H, J = 8.2 Hz, H-1), 4.47 (ddd, 1H, J = 50.3, 9.7, 8.4 Hz, H-4), 3.83 (br s, 1H, O*H*), from ^{1}H {^{19}F} 3.75 (dd, 1H, J = 10.3, 1.7 Hz, H-6´), from ^{1}H {^{19}F} 3.64 (dd, 1H, J = 10.3, 6.0 Hz, H-6), from ^{1}H {^{19}F} 3.59 (ddd, 1H, J = 9.7, 6.0, 1.7 Hz, H-5), 3.50 (ddd, 1H, J = 14.9, 9.9, 8.4 Hz, H-3), 3.35 (ddd, 1H, J = 9.9, 8.2, 0.8 Hz, H-2). ^{13}C{^{1}H} NMR ($CDCl_3$, 101 MHz, HSQC): δ 137.6, 137.5 (2 × C_q), 128.63, 128.59, 128.3 (3 × 2CH_{arom}), 128.2, 128.1 (2 × CH_{arom}), 128.0 (2CH_{arom}), 96.1 (d, ^4J = 1.6 Hz, C-1), 90.2 (d, ^1J = 183.9 Hz, C-4), 80.3 (d, ^2J = 18.2 Hz, C-3), 74.9 (d, ^4J = 2.6 Hz, *C*H_2 O-3Bn), 73.9 (*C*H_2 O-6Bn), 73.1 (d, ^2J = 24.2 Hz, C-5), 68.5 (C-6), 66.3 (d, ^3J = 9.2 Hz, C-2). ^{19}F NMR ($CDCl_3$, 376 MHz): δ −197.68 (ddd, ^2J = 50.3 Hz, ^3J = 14.9, 3.0 Hz). HRMS-APCI (*m/z*): $[M - N_2 + H]^+$ calcd for $C_{20}H_{23}FNO_4$, 360.1605; found, 360.1608.

2-Azido-4,6-di-*O*-benzyl-2,3-dideoxy-3-fluoro-D-galactopyranose (45)

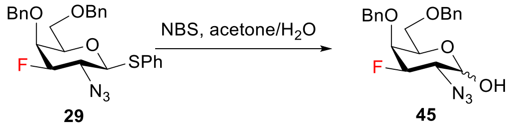

29 45

Thioglycoside **29** (375 mg, 0.78 mmol) was hydrolyzed according to the general procedure. Chromatography in EtOAc/PE 1:4 afforded **45** (238 mg, 79%) as a colorless syrup. R_f 0.50 (EtOAc/PE 1:3). NMR data for the α-anomer: ^{1}H NMR ($CDCl_3$, 400 MHz, ^{1}H {^{19}F}, H-H COSY): δ 7.36–7.25 (m, 10H, CH_{arom}), 5.33 (dt, 1H, J = 5.0, 3.5 Hz, H-1), 4.97 (ddd, 1H, J = 49.1, 10.4, 3.1 Hz, H-3), 4.86 (dd, 1H, J = 11.4, 1.8 Hz, C*H*H O-4Bn), 4.51 (d, 1H, J = 11.4, C*H*H O-4Bn), 4.48, 4.41 (2 × d, 2 × 1H, J = 11.9, C*H*H O-6Bn), from ^{1}H {^{19}F} 4.19 (td, 1H, J = 5.4, 1.3 Hz, H-5), 4.00 (ddd, 1H, J = 6.9, 3.1, 1.3 Hz, H-4), from ^{1}H {^{19}F} 3.94 (ddd, 1H, J = 10.4, 3.5, 1.3 Hz, H-2), 3.76 (dd, 1H, J = 3.5, 1.3 Hz, O*H*), 3.59 (ddd, 1H, J = 9.6, 5.4, 1.1 Hz, H-6), 3.40 (dd, 1H, J = 9.6, 5.4 Hz, H-6´). ^{13}C{^{1}H} NMR ($CDCl_3$, 101 MHz, HSQC, HMBC): δ 137.7, 137.5 (2 × C_q), 128.60, 128.55, 128.5, 128.17 (4 × 2CH_{arom}), 128.15, 128.1 (2 × CH_{arom}), 92.8 (d, ^3J = 9.2 Hz, C-1), 90.6 (d, ^1J = 187.8 Hz, C-3), 75.0 (d, ^4J = 4.1 Hz, *C*H_2 O-4Bn), 74.5 (d, ^2J = 15.3 Hz, C-4), 73.7 (*C*H_2 O-6Bn), 69.1 (d, ^3J = 7.0 Hz, C-5), 69.0 (d, ^4J = 2.8 Hz, C-6), 59.5 (d, ^2J = 17.2 Hz, C-2). ^{19}F NMR ($CDCl_3$, 376 MHz): −200.65 (dddd, ^2J = 49.1 Hz, ^3J = 10.4, 6.9 Hz, ^4J = 5.0 Hz). Resolved signals for the β-anomer: ^{1}H NMR ($CDCl_3$, 400 MHz, ^{1}H {^{19}F}, H-H COSY): δ 7.36–7.25 (m, 10H, CH_{arom}), 4.34 (ddd, 1H,

J = 47.7, 10.1, 3.2 Hz, H-3), from ^{1}H {^{19}F} 3.92 (dd, 1H, J = 3.2, 1.3 Hz, H-4), 3.88 (ddd, 1H, J = 11.1, 10.1, 7.9 Hz, H-2), 3.61 (ddd, 1H, J = 8.8, 6.0, 1.2 Hz, H-6), 3.54 (dddd, 1H, J = 6.0, 5.9, 1.7, 1.3 Hz, H-5), 3.47 (dd, 1H, J = 8.8, 5.9 Hz, H-6´). ^{13}C{^{1}H} NMR ($CDCl_3$, 101 MHz, HSQC, HMBC): δ 137.6, 137.5 (2 × C_q), 96.1 (d, ^{3}J = 10.6 Hz, C-1), 93.1 (d, ^{1}J = 191.0 Hz, C-3), 75.0 (d, ^{4}J = 4.0 Hz, CH$_2$ O-4Bn), 73.7 (CH$_2$ O-6Bn), 73.1 (d, ^{2}J = 15.1 Hz, C-4), 72.6 (d, ^{3}J = 7.8 Hz, C-5), 68.4 (d, ^{4}J = 2.9 Hz, C-6), 63.6 (d, ^{2}J = 17.0 Hz, C-2). ^{19}F NMR ($CDCl_3$, 376 MHz): −195.39 (ddd, ^{2}J = 47.7 Hz, ^{3}J = 11.1, 5.8 Hz). HRMS-APCI (*m/z*): [M − N_2 + H]$^+$ calcd for $C_{20}H_{23}FNO_4$, 360.1605; found, 360.1605.

2-Azido-3,6-di-*O*-benzyl-2,4-dideoxy-4-fluoro-D-galactopyranose (46)

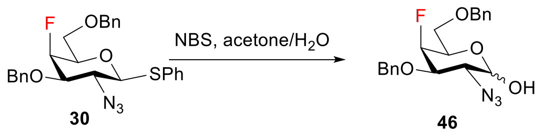

Thioglycoside **30** (330 mg, 0.69 mmol) was hydrolyzed according to the general procedure. Chromatography in EtOAc/PE 1:4 afforded **46** (201 mg, 75%) as a colorless syrup. R_f 0.48 (EtOAc/PE 1:3). NMR data for the α-anomer: ^{1}H NMR ($CDCl_3$, 400 MHz, ^{1}H {^{19}F}, H-H COSY): δ 7.42–7.29 (m, 10H, CH_{arom}), 5.32 (t, 1H, J = 3.4 Hz, H-1), 4.83 (dd, 1H, J = 50.0, 2.5 Hz, H-4), 4.76, 4.70 (2 × d, 2 × 1H, J = 11.7 Hz, CHH Bn), 4.59, 4.53 (2 × d, 2 × 1H, J = 12.0 Hz, CHH Bn), 4.19 (dt, 1H, J = 29.5, 6.5 Hz, H-5), 3.92 (ddd, 1H, J = 26.8, 10.6, 2.5 Hz, H-3), 3.79 (ddd, 1H, J = 10.6, 3.4, 1.3 Hz, H-2), 3.68–3.61 (m, 2H, H-6), 3.59 (dd, 1H, J = 3.4, 1.3 Hz, OH). ^{13}C{^{1}H} NMR ($CDCl_3$, 101 MHz, HSQC): δ 137.5, 137.3 (2 × C_q), 128.7, 128.6 (2 × 2CH_{arom}), 128.2, 128.1 (2 × CH_{arom}), 128.10, 128.07 (2 × 2CH_{arom}), 92.3 (C-1), 85.9 (d, ^{1}J = 184.7 Hz, C-4), 74.4 (d, ^{2}J = 18.0 Hz, C-3), 73.8, 71.9 (2 × CH$_2$ Bn), 68.40 (d, ^{2}J = 18.0 Hz, C-5), 68.38 (d, ^{3}J = 5.4 Hz, C-6), 59.7 (d, ^{3}J = 2.2 Hz, C-2). ^{19}F NMR ($CDCl_3$, 376 MHz): δ −220.71 (ddd, ^{2}J = 50.0 Hz, ^{3}J = 29.5, 26.8 Hz). NMR data for the β-anomer: ^{1}H NMR ($CDCl_3$, 400 MHz, ^{1}H {^{19}F}, H-H COSY): δ 7.42–7.29 (m, 10H, CH_{arom}), 4.76 (dd, 1H, J = 49.7, 2.6 Hz, H-4), 4.76, 4.69 (2 × d, 2 × 1H, J = 12.0 Hz, CHH Bn), 4.58, 4.53 (2 × d, 2 × 1H, J = 11.9 Hz, CHH Bn), 4.48 (ddd, 1H, J = 8.1, 5.6, 1.1 Hz, H-1), 3.98 (d, 1H, J = 5.6 Hz, OH), 3.68–3.61 (m, 3H, H-2, 2H-6), 3.57 (dt, 1H, J = 26.7, 6.5 Hz, H-5), 3.31 (ddd, 1H, J = 27.5, 10.3, 2.6 Hz, H-3). ^{13}C{^{1}H} NMR ($CDCl_3$, 101 MHz, HSQC): δ 137.5, 137.1 (2 × C_q), 128.72, 128.66 (2 × 2CH_{arom}), 128.3, 128.2 (2 × CH_{arom}), 128.1 (4CH_{arom}), 96.2 (C-1), 84.6 (d, ^{1}J = 185.5 Hz, C-4), 77.7 (d, ^{2}J = 18.1 Hz, C-3), 73.9 (CH$_2$ Bn), 72.6 (d, ^{2}J = 18.3 Hz, C-5), 72.1 (CH$_2$ Bn), 67.9 (d, ^{3}J = 5.2 Hz, C-6), 63.9 (d, ^{3}J = 0.8 Hz, C-2). ^{19}F NMR ($CDCl_3$, 376 MHz): δ −218.40 (ddd, ^{2}J = 49.7 Hz, ^{3}J = 27.5, 26.7 Hz). HRMS-APCI (*m/z*): [M − N_2 + H]$^+$ calcd for $C_{20}H_{23}FNO_4$, 360.1605; found, 360.1606.

2-Azido-6-*O*-benzyl-2,3,4-trideoxy-3,4-difluoro-D-glucopyranose (47)

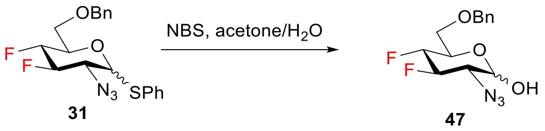

Thioglycoside **31** (268 mg, 0.69 mmol) was hydrolyzed according to the general procedure. Chromatography in EtOAc/PE 1:5 afforded **47** (156 mg, 76%) as a colorless syrup. R_f 0.41 (EtOAc/PE 1:3). NMR data for the α-anomer: ^{1}H NMR ($CDCl_3$, 400 MHz, ^{1}H {^{19}F}, H-H COSY): δ 7.38–7.31 (m, 5H, CH_{arom}), 5.32 (tt, 1H, *J* = 3.6, 3.0 Hz, H-1), 5.00 (dddd, 1H, *J* = 54.0, 15.4, 10.0, 8.2 Hz, H-3), 5.62, 4.58 (2 × d, 2 × 1H, *J* = 12.2, C*H*H Bn), 4.54 (dddd, 1H, *J* = 51.2, 18.0, 10.0, 8.2 Hz, H-4), 4.18 (dddd, 1H, *J* = 10.0, 5.4, 4.9, 2.2 Hz, H-5), 3.88 (d, 1H, *J* = 3.0 Hz, O*H*), 3.73 (ddt, 1H, *J* = 10.9, 2.2, 2.0 Hz, H-6), from ^{1}H {^{19}F} 3.68 (dd, 1H, *J* = 10.9, 5.4 Hz, H-6´), 3.45 (ddd, 1H, *J* = 11.0, 10.0, 3.6 Hz, H-2). ^{13}C{^{1}H} NMR ($CDCl_3$, 101 MHz, HSQC): δ 137.2 (C_q), 128.6 ($2CH_{arom}$), 128.1 (CH_{arom}), 128.0 ($2CH_{arom}$), 92.1 (dd, ^3J = 9.3 Hz, ^4J = 1.3 Hz, C-1), 90.4 (dd, ^1J = 186.3 Hz, ^2J = 19.3 Hz, C-3), 87.4 (dd, ^1J = 186.5 Hz, ^2J = 18.1 Hz, C-4), 73.8 (*C*H_2 Bn), 68.1 (dd, ^2J = 23.5 Hz, ^3J = 6.6 Hz, C-5), 68.0 (t, *J* = 1.1 Hz, C-6), 61.7 (dd, ^2J = 16.6 Hz, ^3J = 6.6 Hz, C-2). ^{19}F NMR ($CDCl_3$, 376 MHz): –198.48 (ddddd, ^2J = 54.0 Hz, ^3J = 18.0, 13.2, 11.0 Hz, ^4J = 3.0 Hz, F-3), –199.25 (ddd, ^2J = 51.2 Hz, ^3J = 15.4, 13.2 Hz, F-4). Resolved signals for the β-anomer: ^{1}H NMR ($CDCl_3$, 400 MHz, ^{1}H {^{19}F}, H-H COSY): δ 4.52 (d, 1H, *J* = 9.3 Hz, H-1), from ^{1}H {^{19}F} 3.76 (dd, 1H, *J* = 10.7, 2.1 Hz, H-6), from ^{1}H {^{19}F} 3.66 (dd, 1H, *J* = 10.7, 5.8 Hz, H-6´), 3.56 (dddd, 1H, *J* = 9.7, 5.4, 2.1, 1.1 Hz, H-5), 3.55 (br s, 1H, O*H*). ^{13}C{^{1}H} NMR ($CDCl_3$, 101 MHz, HSQC): δ 137.1 (C_q), 128.6 ($2CH_{arom}$), 128.2 (CH_{arom}), 128.0 ($2CH_{arom}$), 95.6 (dd, ^3J = 10.2 Hz, ^4J = 1.4 Hz, C-1), 92.1 (dd, ^1J = 189.3 Hz, ^2J = 20.1 Hz, C-3), 86.9 (dd, ^1J = 186.3 Hz, ^2J = 18.6 Hz, C-4), 73.9 (*C*H_2 Bn), 71.9 (dd, ^2J = 23.5 Hz, ^3J = 7.6 Hz, C-5), 65.0 (dd, ^2J = 17.2 Hz, ^3J = 7.7 Hz, C-2). ^{19}F NMR ($CDCl_3$, 376 MHz): –192.68 (dq, ^2J = 51.7 Hz, ^3J = 13.7 Hz, F-3), –201.14 (dddd, ^2J = 50.5 Hz, ^3J = 16.6, 13.7, 2.9 Hz, F-4). HRMS-APCI (*m/z*): $[M - N_2 + H]^+$ calcd for $C_{13}H_{16}F_2NO_3$, 272.1092; found, 272.1089.

2-Azido-6-*O*-benzyl-2,3,4-trideoxy-3,4-difluoro-D-galactopyranose (48)

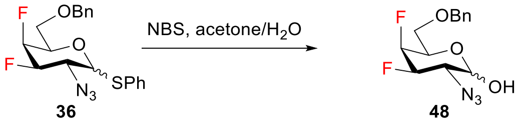

Thioglycoside **36** (285 mg, 0.73 mmol) was hydrolyzed according to the general procedure. Chromatography in EtOAc/PE 1:4 afforded **48** (184 mg, 84%) as a thick colorless gel, R_f 0.33 (EtOAc/PE 1:3). NMR data for the α-anomer: ^{1}H NMR ($CDCl_3$, 400 MHz, ^{1}H {^{19}F}, H-H COSY): δ 7.38–7.30 (m, 5H, CH_{arom}), 5.37 (tt, 1H, *J* = 3.5, 2.8 Hz, H-1), 4.95 (ddd, 1H, *J* = 51.4, 7.6, 2.8 Hz, H-4), 4.89 (dddd, 1H, *J* = 48.0, 25.6, 10.3, 2.8 Hz, H-3), 4.60, 4.54 (2 × d, 2 × 1H, *J* = 11.8 Hz, C*H*H Bn), 4.22 (dddd, 1H, *J* = 29.6, 6.0, 5.8, 2.2 Hz, H-5), 3.84 (ddd, 1H, *J* = 10.3, 3.5,1.2 Hz, H-2), 3.71–3.65 (m, 2H, H-6), 3.60 (dd, 1H, *J* = 3.5, 1.2 Hz, O*H*). ^{13}C{^{1}H} NMR ($CDCl_3$, 101 MHz, HSQC): δ 137.3 (C_q), 128.7 ($2CH_{arom}$), 128.3 (CH_{arom}), 128.1 ($2CH_{arom}$), 92.6 (d, ^3J = 9.1 Hz, C-1), 87.3 (dd, ^1J =

190.5 Hz, ^{2}J = 18.0 Hz, C-3), 86.9 (dd, ^{1}J = 186.1 Hz, ^{2}J = 16.5 Hz, C-4), 73.9 (CH_2 Bn), 68.1 (dd, ^{2}J = 22.0 Hz, ^{3}J = 5.4 Hz, C-5), 68.0 (dd, ^{3}J = 5.5 Hz, ^{4}J = 1.5 Hz, C-6), 58.9 (dd, ^{2}J = 17.3 Hz, ^{3}J = 2.2 Hz, C-2). ^{19}F NMR ($CDCl_3$, 376 MHz): from ^{19}F {^{1}H} −203.96 (d, ^{3}J = 15.0 Hz, F-3), −221.28 (dddd, ^{2}J = 51.4 Hz, ^{3}J = 29.6, 25.6, 15.0 Hz, F-4). Resolved signals for the β-anomer:^{1}H NMR ($CDCl_3$, 400 MHz, ^{1}H {^{19}F}, H-H COSY): δ 7.38–7.30 (m, 5H, CH_{arom}), 4.59, 4.54 (2 × d, 2 × 1H, *J* = 10.9 Hz, C*H*H Bn), 4.50 (dd, 1H, *J* = 7.4, 4.8 Hz, H-1), 4.24 (dddd, 1H, *J* = 46.4, 26.6, 10.2, 2.8 Hz, H-3), 3.99 (d, 1H, *J* = 4.8 Hz, O*H*). 3.79–3.64 (m, 3H, H-2, 2H-6), 3.63 (dtd, 1H, *J* = 25.5, 6.5, 1.9 Hz, H-5). ^{13}C{^{1}H} NMR ($CDCl_3$, 101 MHz, HSQC): δ 137.3 (C_q), 128.7, 128.1 (2 × $2CH_{arom}$), 95.9 (d, ^{3}J = 10.3 Hz, C-1), 89.6 (dd, ^{1}J = 193.6 Hz, ^{2}J = 18.0 Hz, C-3), 85.4 (dd, ^{1}J = 186.4 Hz, ^{2}J = 16.4 Hz, C-4), 73.9 (CH_2 Bn), 71.6 (dd, ^{2}J = 18.2 Hz, ^{3}J = 6.0 Hz, C-5), 67.5 (dd, ^{3}J = 5.1 Hz, ^{4}J =2.9 Hz, C-6), 62.9 (dd, ^{2}J = 17.5 Hz, ^{3}J = 0.7 Hz, C-2). ^{19}F NMR ($CDCl_3$, 376 MHz): −198.49 (dddd, ^{2}J = 46.4 Hz, ^{3}J = 15.6, 12.6, 6.6 Hz, F-3), −218.98 (dddd, ^{2}J = 51.3 Hz, ^{3}J = 26.6, 25.5, 15.6 Hz, F-4). HRMS-APCI (*m/z*): $[M - N_2 + H]^+$ calcd for $C_{13}H_{16}F_2NO_3$, 272.1092; found, 272.1095.

2-Acetamido-4,6-di-*O*-benzyl-2,3-dideoxy-3-fluoro-D-glucopyranose (49)

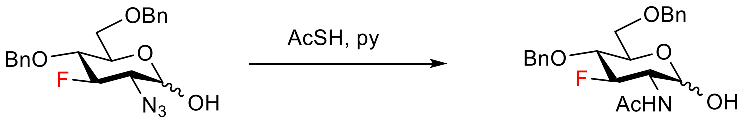

43 **49**

Compound **49** was prepared from compound **43** (258 mg, 0.67 mmol) according to the general procedure for azide/acetamide conversion except for the reaction time (6.5 h). Chromatography in EtOAc gave **49** (149 mg, 55%) as white crystalline material, mp 199–201 °C (EtOAc), R_f 0.24 (EtOAc). NMR data for the α-anomer: ^{1}H NMR (MeOH-d_4, 400 MHz, ^{1}H{^{19}F}, H-H COSY): δ 7.34–7.23 (m, 10H, CH_{arom}), 5.12 (dd, 1H, *J* = 3.9, 3.6 Hz, H-1), 4.79 (dd, 1H, *J* = 11.2, 1.1 Hz, CH*H* O-4Bn), 4.77 (ddd, 1H, *J* = 52.4, 10.4, 8.4 Hz, H-3), 4.56 (d, 1H, *J* = 12.0 Hz, CH*H* O-6Bn), 4.54 (d, 1H, *J* = 11.2 Hz, CH*H* O-4Bn), 4.47 (d, 1H, *J* = 12.0 Hz, CH*H* O-6Bn), 4.13 (ddd, 1H, *J* = 11.0, 10.4, 3.6 Hz, H-2), 3.99 (ddd, 1H, *J* = 10.0, 4.4, 1.9 Hz, H-5), 3.73 (dd, 1H, *J* = 10.8, 4.4 Hz, H-6′), from ^{1}H{^{19}F} 3.71 (dd, 1H, *J* = 10.0, 8.4 Hz, H-4), 3.65 (dt, 1H, *J* = 10.8, 1.9 Hz, H-6), 2.00 (s, 3H, *Me*). ^{13}C{^{1}H} NMR (MeOH-d_4, 101 MHz, HSQC, HMBC): δ 173.5 (CO), 139.5, 139.4 (2 × C_q), 129.4, 129.3, 129.09, 129.06 (4 × $2CH_{arom}$), 128.8, 128.7 (2 × CH_{arom}), 95.2 (d, ^{1}J = 183.1 Hz, C-3), 92.8 (d, ^{3}J = 9.8 Hz, C-1), 78.0 (d, ^{2}J = 16.3 Hz, C-4), 75.5 (d, ^{4}J = 2.8 Hz, CH_2 O-4Bn), 74.4 (CH_2 O-6Bn), 70.7 (d, ^{3}J = 8.3 Hz, C-5), 69.9 (d, ^{4}J = 1.2 Hz, C-6), 54.4 (d, ^{2}J = 16.5 Hz, C-2), 22.5 (Me). ^{19}F NMR (MeOH-d_4, 376 MHz): δ −197.43 (dddd, ^{2}J = 52.4 Hz, ^{3}J = 13.2, 11.0 Hz, ^{4}J = 3.9 Hz). Resolved signals for the β-anomer: ^{1}H NMR (MeOH-d_4, 400 MHz, ^{1}H{^{19}F}, H-H COSY, HSQC): δ 4.63 (d, 1H, *J* = 8.4 Hz, H-1), 3.84 (ddd, 1H, *J* = 12.1, 11.4, 8.4 Hz, H-2). ^{19}F NMR (MeOH-d_4, 376 MHz): δ −192.50 (dt, ^{2}J = 51.9 Hz, ^{3}J = 12.1 Hz). HRMS-APCI (*m/z*): $[M + H]^+$ calcd for $C_{22}H_{27}FNO_5$, 404.1867; found, 404.1868.

2-Acetamido-4-*O*-benzyl-2,3,6-trideoxy-3,6-difluoro-D-glucopyranose (50)

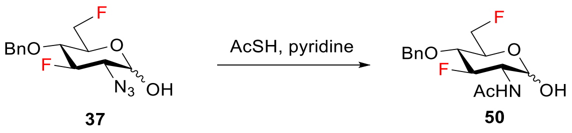

Compound **50** was prepared from **37** (109 mg, 0.36 mmol) according to the general procedure for azide/acetamide conversion. Chromatography in EtOAc/PE 1:1 → EtOAc afforded **50** (83 mg, 72%) as a white crystalline solid, mp 170–180 °C (dec, EtOAc/MeOH). R_f 0.33 (EtOAc). NMR data for the α-anomer: ^{1}H NMR (MeOH-d_4, 400 MHz, ^{1}H {^{19}F}, H-H COSY): δ 7.35–7.27 (m, 5H, CH_{arom}), 5.11 (dd, 1H, J = 3.8, 3.6 Hz, H-1), 4.85 (d, 1H, J = 11.2 Hz, C*H*H Bn), 4.80 (ddd, 1H, J = 53.4, 10.4, 8.3 Hz, H-3), 4.64 (d, 1H, J = 11.2 Hz, CH*H* Bn), 4.62 (ddd, 1H, J = 47.4, 10.3, 3.5 Hz, H-6´), 4.52 (dddd, 1H, J = 48.7, 10.3, 1.9, 1.4 Hz, H-6), 4.12 (ddd, 1H, J = 11.0, 10.4, 3.6 Hz, H-2), 3.99 (dddd, 1H, J = 28.3, 10.1, 3.5, 1.4 Hz, H-5), 3.68 (ddd, 1H, J = 13.7, 10.1, 8.3 Hz, H-4), 2.00 (s, 3H, *Me*). ^{13}C{^{1}H} NMR (MeOH-d_4, 101 MHz, HSQC, HMBC): δ 173.6 (CO), 139.4 (C_q), 129.4, 129.1 (2 × 2CH_{arom}), 128.9 (CH_{arom}), 95.1 (dd, ^1J = 183.0 Hz, ^4J = 0.7 Hz, C-3), 92.8 (d, ^3J = 9.7 Hz, C-1), 83.1 (dd, ^1J = 172.3, ^4J = 1.3 Hz, C-6), 77.0 (dd, ^2J = 16.7 Hz, ^3J = 7.2 Hz, C-4), 75.6 (d, ^4J = 2.9 Hz, *C*H_2 Bn), 70.4 (dd, ^2J = 18.3 Hz, ^3J = 8.7 Hz, C-5), 54.3 (d, ^2J = 16.7 Hz, C-2), 22.5 (Me). ^{19}F NMR (MeOH-d_4, 376 MHz): –197.68 (dddd, ^2J = 53.4 Hz, ^3J = 13.7, 11.0 Hz, ^4J = 3.6 Hz, F-3), –237.04 (ddd, ^2J = 48.7, 47.4 Hz, ^3J = 28.3 Hz, F-6). Resolved signals for the β-anomer: ^{19}F NMR (MeOH-d_4, 376 MHz): –192.51 (dt, ^2J = 51.7 Hz, ^3J = 12.3 Hz, F-3), –236.49 (td, ^2J = 47.8 Hz, ^3J = 25.8 Hz, F-6). HRMS-APCI (*m/z*): [M + H]$^+$ calcd for $C_{15}H_{20}F_2NO_4$, 316.1355; found, 316.1350.

2-Acetamido-3,6-di-*O*-benzyl-2,4-dideoxy-4-fluoro-D-glucopyranose (51)

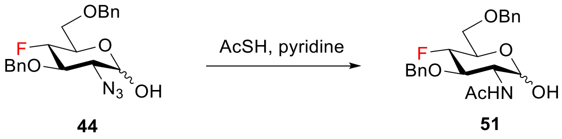

Compound **51** was prepared from **44** (162 mg, 0.42 mmol) according to the general procedure for azide/acetamide conversion. Chromatography in EtOAc/PE 3:1 → EtOAc afforded **51** (114 mg, 68%) as a white crystalline solid, mp 182–185 °C (dec, EtOAc), R_f 0.29 (EtOAc). NMR data for the α-anomer: ^{1}H NMR (MeOH-d_4, 400 MHz, ^{1}H {^{19}F}, H-H COSY): δ 7.35–7.24 (m, 10H, CH_{arom}), 5.06 (dd, J = 3.5, 3.3 Hz, H-1), 4.82, 4.64 (2 × d, 2 × 1H, J = 11.5 Hz, C*H*H O3-Bn), 4.61, 4.56 (2 × d, 2 × 1H, J = 12.0 Hz, C*H*H O6-Bn), 4.51 (ddd, 1H, J = 50.9, 10.0, 8.4 Hz, H-4), 4.17 (ddt, 1H, J = 10.0, 4.5, 3.5 Hz, H-5), 4.06 (ddd, 1H, J = 10.7, 3.5, 1.0 Hz, H-2), 3.93 (ddd, 1H, J = 14.6, 10.7, 8.4 Hz, H-3), 3.93–3.66 (m, 2H, H-6), 1.94 (s, 3H, *Me*). ^{13}C{^{1}H} NMR (MeOH-d_4, 101 MHz, HSQC, HMBC): δ 173.3 (CO), 139.9, 139.4 (2 × C_q), 129.3, 129.2, 128.8 (3 × 2CH_{arom}), 128.7 (CH_{arom}), 128.61 (2CH_{arom}), 128.55 (CH_{arom}), 92.6 (d, ^4J = 1.6 Hz, C-1), 92.2 (d, ^1J = 181.6 Hz, C-4), 79.2 (d, ^2J = 17.4 Hz, C-3), 75.5 (d, ^4J = 2.3 Hz, *C*H_2 O-3Bn), 74.5 (*C*H_2 O-6Bn), 69.8 (C-6), 69.7 (d, ^2J = 23.7 Hz, C-5), 54.3 (d, ^3J = 9.0 Hz, C-2), 22.6 (Me). ^{19}F NMR (MeOH-d_4, 376 MHz): δ −196.54 (dddd, ^2J = 50.9 Hz, ^3J = 14.6, 4.5 Hz, ^5J = 3.3 Hz). Resolved signals for the β-anomer: ^{1}H

NMR (MeOH-d_4, 400 MHz, ^{1}H {^{19}F}, H-H COSY): δ 4.70 (d, 1H, *J* = 8.3 Hz, H-1), 1.891 (s, 3H, *Me*). ^{13}C{^{1}H} NMR ($CDCl_3$, 101 MHz, HSQC, HMBC): δ 23.0 (Me). ^{19}F NMR ($CDCl_3$, 376 MHz): δ −198.67 (dd, ^2J = 50.8 Hz, ^3J = 15.0 Hz). HRMS-APCI (*m/z*): [M + H]$^+$ calcd for $C_{22}H_{27}FNO_5$, 404.1867; found, 404.1880.

2-Acetamido-3-*O*-benzyl-2,4,6-trideoxy-4,6-difluoro-D-glucopyranose (52)

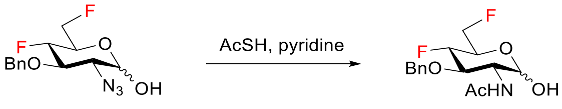

Compound **52** was prepared from **38** (140 mg, 0.47 mmol) according to the general procedure for azide/acetamide conversion. Chromatography in EtOAc/PE 1:1 → EtOAc afforded **52** (125 mg, 85%) as a white amorphous solid, R_f 0.45 (EtOAc). NMR data for the α-anomer: ^{1}H NMR (MeOH-d_4, 400 MHz, ^{1}H {^{19}F}, H-H COSY): δ 7.32–7.30 (m, 5H, CH_{arom}), 5.06 (t, 1H, *J* = 3.4 Hz, H-1), 4.83 (dd, 1H, *J* = 11.5, 1.1 Hz, C*H*H Bn), 4.65 (d, 1H, *J* = 11.5 Hz, CH*H* Bn), 4.60 (dddd, 1H, *J* = 47.2, 10.5, 3.9, 1.7 Hz, H-6´), 4.53 (ddt, 1H, *J* = 48.1, 10.5, 1.9 Hz, H-6), 4.49 (ddd, 1H, *J* = 51.1, 10.1, 8.3 Hz, H-4), 4.17 (ddddd, 1H, *J* = 26.5, 10.1, 4.1, 3.9, 1.9 Hz, H-5), 4.05 (dd, 1H, *J* = 10.8, 3.4 Hz, H-2), 3.96 (ddd, 1H, *J* = 14.5, 10.8, 8.2 Hz, H-3), 1.95 (s, 3H, *Me*). ^{13}C{^{1}H} NMR (MeOH-d_4, 101 MHz, HSQC): δ 173.4 (CO), 139.9 (C_q), 129.3, 128.62 (2 × 2CH_{arom}), 128.59 (CH_{arom}), 92.6 (d, ^4J = 1.5 Hz, C-1), 91.2 (dd, ^1J = 182.0 Hz, ^3J = 7.4 Hz, C-4), 82.6 (d, ^1J = 172.6 Hz, C-6), 79.1 (d, ^2J = 17.2 Hz, C-3), 75.3 (d, ^4J = 2.2 Hz, *C*H_2 Bn), 69.3 (dd, ^2J = 23.8, 18.3 Hz, C-5), 54.2 (d, ^3J = 8.8 Hz, C-2), 22.6 (Me). ^{19}F NMR (MeOH-d_4, 376 MHz): –197.18 (dddd, ^2J = 51.1 Hz, ^3J = 14.5, 4.1 Hz, ^5J = 3.4 Hz, F-4), – 238.37 (ddd, ^2J = 48.1, 47.2 Hz, ^3J = 26.5 Hz, F-6). Resolved signals for the β-anomer:^{1}H NMR (MeOH-d_4, 400 MHz, ^{1}H {^{19}F}, H-H COSY): δ 7.30–7.24 (m, 5H, CH_{arom}), 4.75 (d, 1H, *J* = 8.4 Hz, H-1), 3.84 (ddd, 1H, *J* = 15.3, 10.4, 8.7 Hz, H-3), 3.88–3.65 (m, 1H, H-5), 3.67 (ddd, 1H, *J* = 10.4, 8.4, 0.9 Hz, H-2), 1.91 (s, 3H, *Me*). ^{13}C{^{1}H} NMR (MeOH-d_4, 101 MHz, HSQC): δ 173.6 (CO), 139.7 (C_q), 129.3, 128.8 (2 × 2CH_{arom}), 128.7 (CH_{arom}), 96.6 (d, ^4J = 1.6 Hz, C-1), 82.4 (d, ^1J = 173.1 Hz, C-6), 81.0 (d, ^2J = 17.2 Hz, C-3), 75.6 (d, ^4J = 2.2 Hz, *C*H_2 Bn), 57.5 (d, ^3J = 9.6 Hz, C-2), 23.0 (Me). ^{19}F NMR (MeOH-d_4, 376 MHz): –199.40 (dd, ^2J = 50.8 Hz, ^3J = 15.3 Hz, F-4), –237.77 (td, ^2J = 47.5 Hz, ^3J = 24.5 Hz, F-6). HRMS-APCI (*m/z*): [M + H]$^+$ calcd for $C_{15}H_{20}F_2NO_4$, 316.1355; found, 316.1354.

2-Acetamido-6-*O*-benzyl-2,3,4-trideoxy-3,4-difluoro-D-glucopyranose (53)

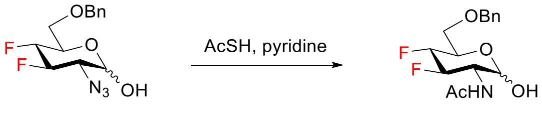

Compound **53** was prepared from **47** (120 mg, 0.40 mmol) according to the general procedure for azide/acetamide conversion. Chromatography in EtOAc/PE 3:1 → EtOAc afforded **53** (105 mg, 83%) as a white crystalline solid, mp 192–195 °C (dec, EtOAc). R_f 0.37 (EtOAc). NMR data for the α-anomer: ^{1}H NMR (MeOH-d_4, 400 MHz, ^{1}H {^{19}F},

H-H COSY): δ 7.36–7.26 (m, 5H, CH_{arom}), 5.12 (q, 1H, *J* = 3.4 Hz, H-1), 4.81 (dddd, 1H, *J* = 54.2, 16.1, 10.4, 8.2 Hz, H-3), 5.61 (d, 1H, *J* = 12.0 Hz, C*H*H Bn), 4.58 (dddd, 1H, *J* = 51.7, 15.1, 10.0, 8.2 Hz, H-4), 4.56 (d, 1H, *J* = 12.0 Hz, C*H*H Bn), 4.17–4.10 (m, 2H, H-2, H-5), 3.77–3.70 (m, 2H, H-6), 1.99 (s, 3H, *Me*). $^{13}C\{^1H\}$ NMR (MeOH-d_4, 101 MHz, HSQC, HMBC): δ 173.5 (CO), 139.4 (C_q), 129.4, 128.8 (2 × $2CH_{arom}$), 128.7 (CH_{arom}), 92.7 (dd, ^3J = 9.4 Hz, ^4J = 1.2 Hz, C-1), 90.4 (dd, ^1J = 186.3 Hz, ^2J = 19.3 Hz, C-3), 89.3 (dd, ^1J = 183.2 Hz, ^2J = 17.9 Hz, C-4), 74.5 (*C*H_2 Bn), 69.5 (d, *J* = 1.3 Hz, C-6), 69.2 (dd, ^2J = 23.2 Hz, ^3J = 6.6 Hz, C-5), 53.9 (dd, ^2J = 16.7 Hz, ^3J = 7.4 Hz, C-2), 22.5 (Me). ^{19}F NMR MeOH-d_4, 376 MHz): – 200.23 (ddddt, ^2J = 51.7 Hz, ^3J = 16.1, 13.8, 5.4 Hz, ^5J = 3.4 Hz, F-4), –201.68 (ddddd, ^2J = 54.2 Hz, ^3J = 15.1, 13.8, 10.7 Hz, ^4J = 3.4 Hz, F-3). Resolved signals for the β-anomer: ^1H NMR (MeOH-d_4, 400 MHz, ^1H {^{19}F}, H-H COSY): δ 4.72 (d, 1H, *J* = 8.4 Hz, H-1), 3.86–3.78 (m, 1H, H-2), 3.69–3.64 (m, 1H, H-5), 2.01 (s, 3H, *Me*). $^{13}C\{^1H\}$ NMR (MeOH-d_4, 101 MHz, HSQC, HMBC): δ 173.8 (CO), 139.2 (C_q), 96.1 (dd, ^3J = 10.2 Hz, ^4J = 1.3 Hz, C-1), 93.0 (dd, ^1J = 186.6 Hz, ^2J = 19.0 Hz, C-3), 89.0 (dd, ^1J = 183.9 Hz, ^2J = 18.3 Hz, C-4), 74.6 (*C*H_2 Bn), 73.1 (dd, ^2J = 23.5 Hz, ^3J = 7.8 Hz, C-5), 57.0 (dd, ^2J = 16.9 Hz, ^3J = 7.8 Hz, C-2), 22.8 (Me). ^{19}F NMR (MeOH-d_4, 376 MHz): –196.48 (ddt, ^2J = 52.3 Hz, ^3J = 13.6, 12.8 Hz, F-3), –202.19 (dddd, ^2J = 51.4 Hz, ^3J = 15.3, 13.6, 1.7 Hz, F-4). HRMS-APCI (*m/z*): $[M + H]^+$ calcd for $C_{15}H_{20}F_2NO_4$, 316.1355; found, 316.1348.

2-Acetamido-4,6-di-*O*-benzyl-2,3-dideoxy-3-fluoro-D-galactopyranose (54)

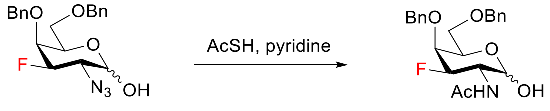

45 54

Compound **54** was prepared from **45** (225 mg, 0.58 mmol) according to the general procedure for azide/acetamide conversion. Chromatography in EtOAc/PE 2:1 → EtOAc afforded **54** (178 mg, 76%) as a white crystalline solid, mp 181–183 °C (dec, EtOAc/MeOH). R_f 0.44 (EtOAc). NMR data for the α-anomer: ^1H NMR (MeOH-d_4, 400 MHz, ^1H {^{19}F}, H-H COSY): δ 7.33–7.25 (m, 10H, CH_{arom}), 5.13 (dd, 1H, *J* = 5.0, 3.6 Hz, H-1), 4.87 (ddd, 1H, *J* = 48.9, 10.8, 3.1 Hz, H-3), 4.82 (d, 1H, *J* = 11.3 Hz, C*H*H O-4Bn), 4.59 (ddd, 1H, *J* = 10.8, 9.0, 3.6 Hz, H-2), 4.51 (d, 1H, *J* = 11.3, C*H*H O-4Bn), 4.49, 4.43 (2 × d, 2 × 1H, *J* = 11.8, C*H*H O-6Bn), 4.22 (dddd, 1H, *J* = 6.8, 6.2, 2.2, 1.4 Hz, H-5), 4.09 (ddd, 1H, *J* = 7.0, 3.1, 1.4 Hz, H-4), 3.60 (dd, 1H, *J* = 9.4, 6.8 Hz, H-6), 3.53 (ddd, 1H, *J* = 9.4, 6.2, 1.4 Hz, H-6´), 1.99 (s, 3H, *Me*). $^{13}C\{^1H\}$ NMR (MeOH-d_4, 101 MHz, HSQC): δ 173.7 (CO), 139.8, 139.4 (2 × C_q), 129.4, 129.3, 129.2, 129.0 (4 × $2CH_{arom}$), 128.8, 128.7 (2 × CH_{arom}), 93.3 (d, ^3J = 9.5 Hz, C-1), 91.5 (d, ^1J = 187.0 Hz, C-3), 76.2 (d, ^2J = 15.2 Hz, C-4), 75.9 (d, ^4J = 4.0 Hz, *C*H_2 O-4Bn), 74.4 (*C*H_2 O-6Bn), 70.0 (d, ^4J = 2.5 Hz, C-6), 69.6 (d, ^3J = 7.1 Hz, C-5), 51.0 (d, ^2J = 17.2 Hz, C-2), 22.6 (Me). ^{19}F NMR (MeOH-d_4, 376 MHz): –202.91 (dddd, ^2J = 48.9 Hz, ^3J = 9.0, 7.0 Hz, ^4J = 5.0 Hz). HRMS-APCI (*m/z*): $[M + H]^+$ calcd for $C_{22}H_{27}FNO_5$, 404.1867; found, 404.1861. NMR signals of the β-anomer could not be observed in intensity sufficient for characterization within 12 h after sample preparation.

2-Acetamido-4-*O*-benzyl-2,3,6-trideoxy-3,6-difluoro-D-galactopyranose (55)

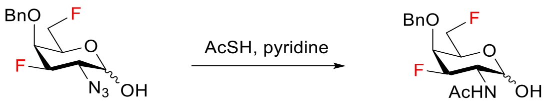

Compound **55** was prepared from **39** (98 mg, 0.33 mmol) according to the general procedure for azide/acetamide conversion. Chromatography in EtOAc/PE 2:1 → EtOAc afforded **55** (73 mg, 71%) as a white crystalline solid, mp 182–183 °C (dec, EtOAc). R_f 0.35 (EtOAc). NMR data for the α-anomer: ^{1}H NMR (MeOH-d_4, 400 MHz, ^{1}H {^{19}F}, H-H COSY): δ 7.37–7.28 (m, 5H, CH_{arom}), 5.16 (dd, 1H, J = 4.9, 3.6 Hz, H-1), 4.89 (ddd, 1H, J = 50.6, 10.9, 3.1 Hz, H-3), 4.85 (d, 1H, J = 11.3 Hz, C*H*H Bn), 4.60 (ddd, 1H, J = 10.9, 8.9, 3.6 Hz, H-2), 4.54 (d, 1H, J = 11.3 Hz, C*H*H Bn), 4.51–4.37 (m, 2H, H-6), 4.29 (ddddd, 1H, J = 13.0, 6.8, 5.2, 2.1, 1.4 Hz, H-5), 4.12 (ddd, 1H, J = 6.8, 3.1, 1.4 Hz, H-4), 2.00 (s, 3H, *Me*). ^{13}C{^{1}H} NMR (MeOH-d_4, 101 MHz, HSQC): δ 173.7 (CO), 139.6 (C_q), 129.4, 129.3 (2 × $2CH_{arom}$), 128.9 (CH_{arom}), 93.4 (d, ^{3}J = 9.4 Hz, C-1), 91.3 (dd, ^{1}J = 187.0 Hz, ^{4}J = 1.4 Hz, C-3), 83.5 (dd, ^{1}J = 167.3, ^{4}J = 3.1 Hz, C-6), 75.9 (d, ^{4}J = 4.3 Hz, CH_2 Bn), 75.8 (dd, ^{2}J = 15.3 Hz, ^{3}J = 6.5 Hz, C-4), 69.6 (dd, ^{2}J = 23.3 Hz, ^{3}J = 7.4 Hz, C-5), 50.8 (d, ^{2}J = 17.1 Hz, C-2), 22.6 (Me). ^{19}F NMR (MeOH-d_4, 376 MHz): –203.45 (ddddd, ^{2}J = 50.6 Hz, ^{3}J = 8.9, 6.8 Hz, ^{4}J = 4.9, 2.1 Hz, F-3), −233.00 (tdd, ^{2}J = 47.3 Hz, ^{3}J = 13.0 Hz, ^{4}J = 2.3 Hz, F-6). Resolved signals for the β-anomer: ^{1}H NMR (MeOH-d_4, 400 MHz, ^{1}H {^{19}F}, H-H COSY): δ from ^{1}H {^{19}F} 4.72 (dd, 1H, J = 10.7, 3.1 Hz, H-3), 4.64 (d, 1H, J = 7.4 Hz, H-1), 4.06 (ddd, 1H, J = 5.2, 3.1, 2.7 Hz, H-4). ^{13}C{^{1}H} NMR (MeOH-d_4, 101 MHz, HSQC): δ 96.6 (d, ^{3}J = 10.4 Hz, C-1), 54.5 (d, ^{2}J = 17.5 Hz, C-2), 22.9 (Me). ^{19}F NMR (MeOH-d_4, 376 MHz): –199.00 (m, F-3), –232.74 (m, F-6). HRMS-APCI (*m/z*): [M + H]$^{+}$ calcd for $C_{15}H_{20}F_2NO_4$, 316.1355; found, 316.1353.

2-Acetamido-3,6-di-*O*-benzyl-2,4-dideoxy-4-fluoro-D-galactopyranose (56)

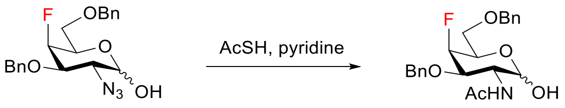

Compound **56** was prepared from **46** (185 mg, 0.48 mmol) according to the general procedure for azide/acetamide conversion. Chromatography in EtOAc/PE 2:1 → EtOAc afforded **56** (162 mg, 84%) as a white crystalline solid, mp 215–217 °C (dec, EtOAc/MeOH). R_f 0.34 (EtOAc). NMR data for the α-anomer: ^{1}H NMR (MeOH-d_4, 400 MHz, ^{1}H {^{19}F}, H-H COSY): δ 7.36–7.25 (m, 10H, CH_{arom}), 5.12 (d, 1H, J = 3.5 Hz, H-1), 4.95 (dd, 1H, J = 50.8, 2.5 Hz, H-4), 4.72, 4.58 (2 × d, 2 × 1H, J = 11.8 Hz, C*H*H Bn), 4.55 (s, 2H, CH_2 Bn), 4.35 (dd, 1H, J = 11.1, 3.5 Hz, H-2), 4.21 (ddd, 1H, J = 29.7, 6.7, 6.5 Hz, H-5), 3.86 (ddd, 1H, J = 26.2, 11.1, 2.5 Hz, H-3), 3.69 (dd, 1H, J = 9.7, 6.7 Hz, H-6´), 3.60 (ddd, 1H, J = 9.7, 6.5, 1.4 Hz, H-6), 1.96 (s, 3H, *Me*). ^{13}C{^{1}H} NMR (MeOH-d_4, 101 MHz, HSQC, HMBC): δ 173.5 (CO), 139.7, 139.4 (2 × C_q), 129.4, 129.3, 128.9 (3 × $2CH_{arom}$), 128.8 (CH_{arom}), 128.74 ($2CH_{arom}$), 128.70 (CH_{arom}), 93.0 (C-1), 87.4 (d, ^{1}J = 182.3 Hz, C-4), 75.6 (d, ^{2}J = 18.4 Hz, C-3), 74.4 (CH_2 O-6Bn), 72.5 (CH_2 O-3Bn),

69.6 (d, ^3J = 5.6 Hz, C-6), 68.9 (d, ^2J = 18.0 Hz, C-5), 50.8 (d, ^3J = 2.7 Hz, C-2), 22.7 (Me). ^{19}F NMR (MeOH-d_4, 376 MHz): δ −223.37 (ddd, ^2J = 50.8 Hz, ^3J = 29.7, 26.2 Hz). Resolved signals for the β-anomer: ^{1}H NMR (MeOH-d_4, 400 MHz, ^{1}H {^{19}F}, H-H COSY): δ from ^{1}H {^{19}F} 4.91 (d, 1H, *J* = 2.6 Hz, H-4), 4.71 (d, 1H, *J* = 11.8 Hz, C*H*H Bn), 4.55 (s, 2H, CH_2 Bn), 4.69 (dd, 1H, *J* = 7.9, 1.0 Hz, H-1), 3.88 (dd, 1H, *J* = 11.0, 7.9 Hz, H-2), 3.77–3.65 (m, 2H, H-3, H-5), 1.94 (s, 3H, *Me*). ^{13}C{^{1}H} NMR (MeOH-d_4, 101 MHz, HSQC, HMBC): δ 173.8 (CO), 96.8 (C-1), 86.3 (d, ^1J = 182.9 Hz, C-4), 78.4 (d, ^2J = 17.8 Hz, C-3), 74.5 (CH_2 O-6Bn), 73.4 (d, ^2J = 17.9 Hz, C-5), 72.5 (CH_2 O-3Bn), 69.4 (d, ^3J = 5.1 Hz, C-6), 54.8 (d, ^3J = 1.6 Hz, C-2), 23.0 (Me). ^{19}F NMR (MeOH-d_4, 376 MHz): δ −221.09 (dt, ^2J = 52.0 Hz, ^3J = 26.5 Hz). HRMS-APCI (*m/z*): $[M + H]^+$ calcd for $C_{22}H_{27}FNO_5$, 404.1867; found, 404.1862.

2-Acetamido-3-*O*-benzyl-2,4,6-trideoxy-4,6-difluoro-D-galactopyranose (57)

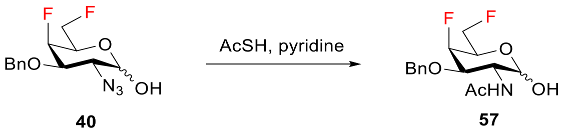

Compound **57** was prepared from **40** (74 mg, 0.25 mmol) according to the general procedure for azide/acetamide conversion. Chromatography in EtOAc/PE 2:1 → EtOAc afforded **57** (62 mg, 80%) as a white crystalline solid, mp 205–206 °C (dec, EtOAc/EtOH), R_f 0.52 (EtOAc). NMR data for the α-anomer: ^{1}H NMR (MeOH-d_4, 400 MHz, ^{1}H {^{19}F}, H-H COSY): δ 7.36–7.24 (m, 5H, CH_{arom}), 5.15 (d, 1H, *J* = 3.4 Hz, H-1), 4.99 (dd, 1H, *J* = 51.2, 2.5 Hz, H-4), 4.73 (d, 1H, *J* = 11.6 Hz, C*H*H Bn), 4.59 (ddd, 1H, *J* = 46.3, 9.4, 5.6 Hz, H-6′), 4.58 (d, 1H, *J* = 11.6 Hz, C*H*H Bn), 4.49 (dddd, 1H, *J* = 47.1, 9.4, 6.7, 1.2 Hz, H-6), 4.37 (dd, 1H, *J* = 11.1, 3.4 Hz, H-2), 4.31 (dddd, 1H, *J* = 30.1, 12.7, 6.7, 5.6 Hz, H-5), 3.89 (ddd, 1H, *J* = 27.9, 11.1, 2.5 Hz, H-3), 1.96 (s, 3H, *Me*). ^{13}C{^{1}H} NMR (MeOH-d_4, 101 MHz, HSQC): δ 173.5 (CO), 139.5 (C_q), 129.3 ($2CH_{arom}$), 128.69 (CH_{arom}), 128.68 ($2CH_{arom}$), 93.0 (C-1), 87.0 (dd, ^1J = 181.9 Hz, ^3J = 5.9 Hz, C-4), 82.7 (dd, ^1J = 168.3 Hz, ^3J = 6.3 Hz, C-6), 75.3 (dd, ^2J = 18.1 Hz, ^4J = 0.9 Hz, C-3), 72.4 (CH_2 Bn), 68.8 (dd, ^2J = 23.2, 17.8 Hz, C-5), 50.6 (d, ^3J = 2.7 Hz, C-2), 22.7 (Me). ^{19}F NMR (MeOH-d_4, 376 MHz): –223.13 (ddd, ^2J = 51.2 Hz, ^3J = 30.1, 27.9 Hz, F-4), –234.00 (ddd, ^2J = 47.1, 46.3 Hz, ^3J = 12.7 Hz, F-6). Resolved signals for the β-anomer: ^{1}H NMR (MeOH-d_4, 400 MHz, ^{1}H {^{19}F}, H-H COSY): δ 7.36–7.24 (m, 5H, CH_{arom}), 4.94 (dd, 1H, *J* = 50.8, 2.5 Hz, H-4), 3.95–3.83 (m, 2H, H-2, H-5), 3.76 (ddd, 1H, *J* = 27.8, 11.0, 2.5 Hz, H-3), 1.94 (s, 3H, *Me*). ^{13}C{^{1}H} NMR (MeOH-d_4, 101 MHz, HSQC): δ 173.8 (CO), 139.4 (C_q), 129.4 ($2CH_{arom}$), 128.8 (CH_{arom}), 128.7 ($2CH_{arom}$), 96.7 (C-1), 85.9 (dd, ^1J = 182.8 Hz, ^3J = 5.9 Hz, C-4), 82.4 (dd, ^1J = 169.2 Hz, ^3J = 6.2 Hz, C-6), 78.1 (d, ^2J = 18.0 Hz, C-3), 73.1 (dd, ^2J = 22.8, 17.7 Hz, C-5), 72.6 (CH_2 Bn), 54.7 (d, ^3J = 1.7 Hz, C-2), 23.0 (Me). ^{19}F NMR (MeOH-d_4, 376 MHz): –220.96 (dt, ^2J = 50.8 Hz, ^3J = 27.8 Hz, F-4), –233.70 (td, ^2J = 46.9 Hz, ^3J = 11.7 Hz, F-6). HRMS-APCI (*m/z*): $[M + H]^+$ calcd for $C_{15}H_{20}F_2NO_4$, 316.1355; found, 316.1354.

2-Acetamido-6-*O*-benzyl-2,3,4-trideoxy-3,4-difluoro-D-galactopyranose (58)

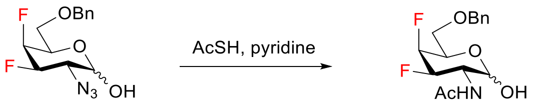

48 **58**

Compound **58** was prepared from **48** (160 mg, 0.53 mmol) according to the general procedure for azide/acetamide conversion. Chromatography in EtOAc/PE 2:1 → EtOAc afforded **58** (105 mg, 62%) as a white crystalline solid, mp 207–208 °C (dec, EtOAc). R_f 0.29 (EtOAc). NMR data for the α-anomer: ^{1}H NMR (MeOH-d_4, 400 MHz, ^{1}H {^{19}F}, H-H COSY): δ 7.35–7.25 (m, 5H, CH$_{arom}$), 5.16 (dd, 1H, *J* = 5.3, 3.5 Hz, H-1), 5.00 (ddd, 1H, *J* = 51.7, 8.1, 2.7 Hz, H-4), 4.81 (dddd, 1H, *J* = 47.5, 26.8, 10.8, 2.7 Hz, H-3), 4.58, 4.54 (2 × d, 2 × 1H, *J* = 12.2 Hz, C*H*H Bn), 4.43 (ddd, 1H, *J* = 10.8, 9.1, 3.5 Hz, H-2), 4.25 (dddd, 1H, *J* = 29.0, 6.5, 1.8, 0.9 Hz, H-5), 3.71 (dd, 1H, *J* = 9.7, 6.8 Hz, H-6), 3.71 (dddd, 1H, *J* = 9.7, 6.5, 1.8, 0.9 Hz, H-6´), 1.99 (s, 3H, *Me*). ^{13}C{^{1}H} NMR (MeOH-d_4, 101 MHz, HSQC, HMBC): δ 173.6 (CO), 139.3 (C$_q$), 129.4, 128.9 (2 × 2CH$_{arom}$), 128.8 (CH$_{arom}$), 93.2 (d, ^3J = 9.2 Hz, C-1), 88.17 (dd, ^1J = 183.3 Hz, ^2J = 20.0 Hz, C-3/4), 88.16 (dd, ^1J = 188.8 Hz, ^2J = 18.3 Hz, C-3/4), 74.4 (*C*H$_2$ Bn), 69.1 (dd, ^3J = 5.6 Hz, ^3J = 2.4 Hz, C-6), 68.6 (dd, ^2J = 18.0 Hz, ^3J = 5.5 Hz, C-5), 50.5 (dd, ^2J = 17.4 Hz, ^3J = 2.6 Hz, C-2), 22.5 (Me). ^{19}F NMR (MeOH-d_4, 376 MHz): from ^{19}F {^{1}H} −206.55 (d, ^3J = 15.0 Hz, F-3), –224.59 (dddd, ^2J = 51.7 Hz, ^3J = 29.0, 26.7, 15.0 Hz, F-4). Resolved signals for the β-anomer: ^{1}H NMR (MeOH-d_4, 400 MHz, ^{1}H {^{19}F}, H-H COSY): δ 4.69 (dd, 1H, *J* = 8.3, 1.2 Hz, H-1). ^{13}C{^{1}H} NMR (MeOH-d_4, 101 MHz, HSQC, HMBC): δ 96.3 (d, ^3J = 10.7 Hz, C-1), 22.9 (Me). ^{19}F {^{1}H} NMR (MeOH-d_4, 376 MHz): −200.80 (d, ^3J = 15.1 Hz, F-3), –220.84 (d, ^3J = 15.1 Hz, F-4). HRMS-APCI (*m/z*): [M + H]$^+$ calcd for $C_{15}H_{20}F_2NO_4$, 316.1355; found, 316.1349.

2-Acetamido-2,3,4,6-tetradeoxy-3,4,6-trifluoro-D-glucopyranose (59)

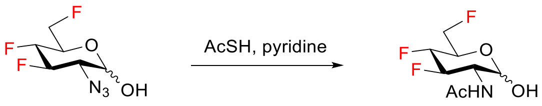

41 **59**

Compound **59** was prepared from **41** (180 mg, 0.85 mmol) according to the general procedure for azide/acetamide conversion. Chromatography in EtOAc/PE 1:1 → EtOAc afforded **59** (144 mg, 74%) as a white crystalline solid, mp 175–177 °C (dec, sub >160 °C, EtOAc/MTBE), R_f 0.40 (EtOAc). NMR data for the α-anomer: ^{1}H NMR (MeOH-d_4, 400 MHz, ^{1}H {^{19}F}, H-H COSY): δ 5.12 (q, 1H, *J* = 3.6 Hz, H-1), 4.84 (dddd, 1H, *J* = 54.0, 16.4, 10.4, 8.3 Hz, H-3), 4.72–4.50 (m, 2H, H-6), from ^{1}H {^{19}F} 4.58 (dd, 1H, *J* = 10.0, 8.3 Hz, H-4), 4.21–4.10 (m, 1H, H-5), 4.13 (ddd, 1H, *J* = 11.1, 10.4, 3.6 Hz, H-2), 2.00 (s, 3H, *Me*). ^{13}C{^{1}H} NMR (MeOH-d_4, 101 MHz, HSQC, HMBC): δ 173.6 (CO), 92.7 (dd, ^3J = 9.5 Hz, ^4J = 1.5 Hz, C-1), 91.5 (ddd, ^1J = 185.0 Hz, ^2J = 18.7 Hz, ^4J = 0.9 Hz, C-3), 88.3 (ddd, ^1J = 183.7 Hz, ^2J = 18.3 Hz, ^3J = 8.1 Hz, C-4), 82.4 (d, ^1J = 173.4 Hz, C-6), 68.8 (ddd, ^2J = 23.2, 18.5 Hz, ^3J = 7.0 Hz, C-5), 53.9 (dd, ^2J = 16.6 Hz, ^3J = 7.3 Hz, C-2), 22.4 (Me). ^{19}F NMR (MeOH-d_4, 376 MHz): –200.72 (ddddd, ^2J = 52.6 Hz, ^3J = 16.8, 13.3, 3.6 Hz, ^5J = 3.6 Hz, F-4), –201.81 (ddddd, ^2J = 54.0 Hz, ^3J = 14.2, 13.3, 11.1 Hz, ^4J = 3.6

Hz, F-3), –238.52 (td, ^2J = 47.7 Hz, ^3J = 26.7 Hz, F-6). HRMS-APCI (*m/z*): $[M + H]^+$ calcd for $C_8H_{13}F_3NO_3$, 228.0842; found, 228.0846. NMR signals of the β-anomer could not be observed in intensity sufficient for characterization within 12 h after sample preparation.

2-Acetamido-2,3,4,6-tetradeoxy-3,4,6-trifluoro-D-galactopyranose (60)

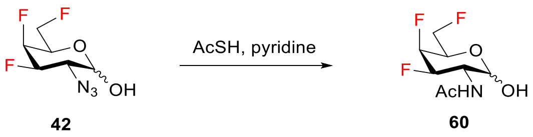

Compound **60** was prepared from **42** (110 mg, 0.52 mmol) according to the general procedure for azide/acetamide conversion. Chromatography in EtOAc/PE 2:1 → EtOAc afforded **60** (101 mg, 85%) as a white crystalline solid, mp 180–205 °C (dec, EtOAc/EtOH), R_f 0.42 (EtOAc). NMR data for the α-anomer: ^{1}H NMR (MeOH-d_4, 400 MHz, ^{1}H {^{19}F}, H-H COSY): δ 5.19 (dd, 1H, *J* = 3.5, 5.3 Hz, H-1), 5.03 (ddd, 1H, *J* = 52.5, 8.2, 2.8 Hz, H-4), 4.84 (dddd, 1H, *J* = 47.3, 26.7, 11.0, 2.8 Hz, H-3), 4.61 (ddd, 1H, *J* = 46.2, 9.4, 5.6 Hz, H-6´), 4.61 (ddddd, 1H, *J* = 47.1, 9.4, 6.6, 1.4, 1.1 Hz, H-6), from ^{1}H {^{19}F} 4.44 (dd, 1H, *J* = 11.0, 3.5 Hz, H-2), 4.35 (ddddd, 1H, *J* = 29.3, 13.0, 6.6, 5.6, 1.6 Hz, H-5), 2.00 (s, 3H, *Me*). ^{13}C{^{1}H} NMR (MeOH-d_4, 101 MHz, HSQC, HMBC): δ 173.7 (CO), 93.2 (d, ^3J = 9.3 Hz, C-1), 87.9 (ddd, ^1J = 189.9 Hz, ^2J = 18.2 Hz, ^4J = 1.2 Hz, C-3), 87.87 (ddd, ^1J = 182.0 Hz, ^2J = 16.5 Hz, ^3J = 6.6 Hz, C-4), 82.4 (ddd, ^1J = 168.5 Hz, ^3J = 6.3 Hz, ^4J = 2.8 Hz, C-6), 68.4 (ddd, ^2J = 23.5 Hz, 17.6 Hz, ^3J = 5.7 Hz, C-5), 50.4 (dd, ^2J = 17.3 Hz, ^3J = 2.6 Hz, C-2), 22.5 (Me). ^{19}F NMR (MeOH-d_4, 376 MHz): –207.09 (dddddd, ^2J = 47.3 Hz, ^3J = 14.4, 10.0, 8.2 Hz, ^4J = 5.3 Hz, ^5J = 1.8 Hz, F-3), – 224.30 (dddd, ^2J = 52.5 Hz, ^3J = 29.3, 26.7, 14.4 Hz, F-4), –234.38 (dddd, ^2J = 47.1, 46.2 Hz, ^3J = 13.0 Hz, ^5J = 1.8 Hz, F-6). Resolved signals for the β-anomer: ^{1}H NMR (MeOH-d_4, 400 MHz, ^{1}H {^{19}F}, H-H COSY): δ 4.73 (dd, 1H, *J* = 8.6, 1.0 Hz, H-1), 4.05 (ddd, 1H, *J* = 11.2, 11.0, 8.6 Hz, H-2), 3.93 (ddtd, 1H, *J* = 25.5, 11.8, 6.4, 2.0 Hz, H-5). ^{13}C{^{1}H} NMR (MeOH-d_4, 101 MHz, HSQC, HMBC): δ 174.0 (CO), 96.3 (d, ^3J = 10.4 Hz, C-1), 82.1 (ddd, ^1J = 169.4 Hz, ^3J = 5.8 Hz, ^4J = 2.3 Hz, C-6), 72.0 (ddd, ^2J = 23.2, 17.5 Hz, ^3J = 6.0 Hz, C-5), 54.2 (dd, ^2J = 17.4 Hz, ^3J = 1.9 Hz, C-2), 22.9 (Me). ^{19}F NMR (MeOH-d_4, 376 MHz): –202.55 (dddt, ^2J = 46.3 Hz, ^3J = 14.5, 8.6 Hz, ^5J = 1.8 Hz, F-3), – 221.98 (dddd, ^2J = 52.6 Hz, ^3J = 28.1, 25.5, 14.5 Hz, F-4), –234.15 (tdd, ^2J = 46.5 Hz, ^3J = 11.8 Hz, ^5J = 1.8 Hz, F-6). HRMS-APCI (*m/z*): $[M + H]^+$ calcd for $C_8H_{13}F_3NO_3$, 228.0842; found, 228.0846.

2-Acetamido-2,3-dideoxy-3-fluoro-D-glucopyranose (61)

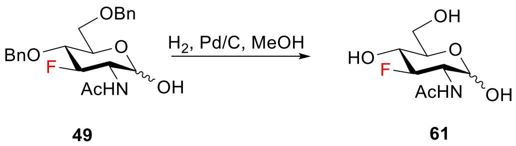

Compound **49** (134 mg, 0.33 mmol) was hydrogenated overnight according to the general procedure for debenzylation using palladium on carbon (70 mg) in methanol (9 mL) to afford white crystalline material that was

purified by recrystallization to give **61** [4] (50 mg, 68%), mp 178–180 °C (EtOH/MTBE), R_f 0.35 (EtOAc/MeOH 5:1). NMR data for the α-anomer: ^{1}H NMR (MeOH-d_4, 400 MHz, ^{1}H{^{19}F}, H-H COSY): δ 5.12 (dd, 1H, J = 3.8, 3.6 Hz, H-1), 4.57 (ddd, 1H, J = 53.8, 10.4, 8.5 Hz, H-3), 4.06 (ddd, 1H, J = 10.5, 10.4, 3.6 Hz, H-2), 3.84–3.78 (m, 2H, H-5, H-6´), 3.74 (dd, 1H, J = 12.0, 5.1 Hz, H-6), 3.65 (ddd, 1H, J = 15.0, 9.8, 8.5 Hz, H-4), 1.99 (s, 3H, *Me*). ^{13}C{^{1}H} NMR (MeOH-d_4, 101 MHz, HSQC): δ 173.5 (CO), 94.2 (d, ^1J = 182.3 Hz, C-3), 92.9 (d, ^3J = 9.8 Hz, C-1), 72.6 (d, ^3J = 7.1 Hz, C-5), 70.3 (d, ^2J = 17.5 Hz, C-4), 62.3 (d, ^4J = 1.4 Hz, C-6), 54.3 (d, ^2J = 16.4 Hz, C-2), 22.5 (Me). ^{19}F NMR (MeOH-d_4, 376 MHz): δ −201.21 (ddddd, ^2J = 53.8 Hz, ^3J = 15.0, 10.5 Hz, ^4J = 3.8, 1.1 Hz). Resolved signals for the β-anomer: 4.63 (d, 1H, J = 8.4 Hz, H-1), 4.34 (ddd, 1H, J = 52.1, 10.2, 8.6 Hz, H-3), 3.87 (dt, 1H, J = 12.0, 1.6 Hz, H-6), 3.59 (ddd, 1H, J = 12.9, 9.7, 8.6 Hz, H-4), 3.28 (dddd, 1H, J = 9.7, 5.5, 2.3, 1.2 Hz, H-5). ^{13}C{^{1}H} NMR (MeOH-d_4, 101 MHz, HSQC): δ 173.8 (CO), 96.3 (d, ^3J = 10.6 Hz, C-1), 95.9 (d, ^1J = 184.7 Hz, C-3), 76.8 (d, ^3J = 8.1 Hz, C-5), 70.1 (d, ^2J = 17.6 Hz, C-4), 62.4 (d, ^4J = 2.0 Hz, C-6), 57.1 (d, ^2J = 17.1 Hz, C-2), 22.8 (Me). ^{19}F NMR (MeOH-d_4, 376 MHz): δ −196.02 (dt, ^2J = 52.1 Hz, ^3J = 12.9 Hz Hz). HRMS-APCI (*m/z*): $[M+H]^+$ calcd for $C_8H_{15}FNO_5$, 224.0929; found, 224.0927.

2-Acetamido-2,3,6-trideoxy-3,6-difluoro-D-glucopyranose (62)

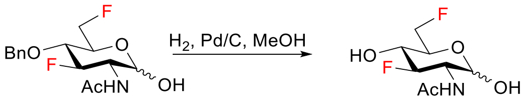

50 **62**

Compound **50** (85 mg, 0.27 mmol) was hydrogenated for 2 days according to the general procedure for debenzylation using palladium on carbon (40 mg) in methanol (5 mL) to afford the product **62** (58 mg, 94%) as a white crystalline solid, mp 208–210 °C (EtOH), R_f 0.17 (EtOAc). NMR signals of the α-anomer: ^{1}H NMR (MeOH-d_4, 400 MHz, ^{1}H {^{19}F}, H-H COSY): δ 5.12 (dd, 1H, J = 3.8, 3.6 Hz, H-1), 4.66 (ddd, 1H, J = 47.5, 10.2, 4.0 Hz, H-6´), from ^{1}H {^{19}F-3} 4.58 (dd, 1H, J = 10.5, 8.5 Hz, H-3), from ^{1}H {^{19}F-6} 4.56 (ddd, 1H, J = 10.2, 1.6, 1.4 Hz, H-6), 4.06 (ddd, 1H, J = 10.6, 10.5, 3.6 Hz, H-2), 3.94 (dddd, 1H, J = 26.7, 10.2, 4.0, 1.6 Hz, H-5), 3.66 (ddd, 1H, J = 14.7, 10.2, 8.5 Hz, H-4), 1.99 (s, 3H, *Me*). ^{13}C{^{1}H} NMR (MeOH-d_4, 101 MHz, HSQC): δ 173.5 (CO), 94.0 (dd, ^1J = 182.6 Hz, ^4J = 0.9 Hz, C-3), 92.9 (d, ^3J = 9.8 Hz, C-1), 83.1 (dd, ^1J = 171.8, ^4J = 1.6 Hz, C-6), 71.4 (dd, ^2J = 18.0 Hz, ^3J = 7.8 Hz, C-5), 69.3 (dd, ^2J = 18.2 Hz, ^3J = 7.6 Hz, C-4), 54.2 (d, ^2J = 16.5 Hz, C-2), 22.5 (Me). ^{19}F NMR (MeOH-d_4, 376 MHz): –201.24 (dddd, ^2J = 53.4 Hz, ^3J = 14.7, 10.6 Hz, ^4J = 3.8 Hz, F-3), –238.19 (ddd, ^2J = 48.6, 47.5 Hz, ^3J = 26.7 Hz, F-6). HRMS-APCI (*m/z*): $[M + H]^+$ calcd for $C_8H_{14}F_2NO_4$, 226.0885; found, 226.0884. NMR signals of the β-anomer could not be observed in intensity sufficient for characterization within 12 h after sample preparation.

2-Acetamido-2,4-dideoxy-4-fluoro-D-glucopyranose (63)

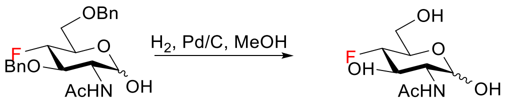

Compound **51** (58 mg, 0.14 mmol) was hydrogenated overnight according to the general procedure for debenzylation using palladium on carbon (60 mg) in methanol (5 mL) to afford the known product **63** [5] (30 mg, 93%) as a yellowish crystalline solid, mp 183–185 °C (dec, EtOH/EtOAc, ref 5 gives 176–180 °C (dec)), R_f 0.23 (EtOAc/MeOH 5:1). NMR data for the α-anomer: ^{1}H NMR (MeOH-d_4, 400 MHz, ^{1}H {^{19}F}, H-H COSY): δ 5.09 (dd, 1H, J = 3.3, 3.1 Hz, H-1), 4.30 (ddd, 1H, J = 50.9, 9.9, 8.2 Hz, H-4), 4.00–3.92 (m, 1H, H-5), from ^{1}H {^{19}F} 3.96 (dd, 1H, J = 10.7, 8.2 Hz, H-3), 3.88 (dd, 1H, J = 10.7, 3.3 Hz, H-2), 3.84–3.67 (m, 2H, H-6), 1.99 (s, 3H, *Me*). ^{13}C{^{1}H} NMR (MeOH-d_4, 101 MHz, HSQC): δ 173.7 (CO), 92.4 (d, ^4J = 1.5 Hz, C-1), 91.5 (d, ^1J = 180.0 Hz, C-4), 70.60 (d, ^2J = 19.0 Hz, C-3), 70.58 (d, ^2J = 23.8 Hz, C-5), 61.8 (C-6), 55.5 (d, ^3J = 8.2 Hz, C-2), 22.6 (Me). ^{19}F NMR (MeOH-d_4, 376 MHz): δ −199.79 (m). Resolved signals for the β-anomer: ^{1}H NMR (MeOH-d_4, 400 MHz, ^{1}H {^{19}F}, H-H COSY): δ 4.65 (d, 1H, J = 8.3 Hz, H-1), 4.27 (ddd, 1H, J = 50.8, 9.9, 8.5 Hz, H-4), 3.84–3.67 (m, 3H, H-3, 2H-6), 3.61 (dd, 1H, J = 10.6, 8.3 Hz, H-2), 3.47 (ddt, 1H, J = 9.9, 5.2, 2.5 Hz, H-5), 1.99 (s, 3H, *Me*). ^{13}C{^{1}H} NMR (MeOH-d_4, 101 MHz, HSQC): δ 174.1 (CO), 96.9 (d, ^4J = 1.4 Hz, C-1), 91.1 (d, ^1J = 180.9 Hz, C-4), 75.4 (d, ^2J = 24.2 Hz, C-5), 73.6 (d, ^2J = 18.6 Hz, C-3), 61.9 (C-6), 58.6 (d, ^3J = 8.6 Hz, C-2), 22.9 (Me). ^{19}F NMR (MeOH-d_4, 376 MHz): δ −201.87 (ddd, ^2J = 50.8 Hz, ^3J = 15.6, 2.5 Hz).

2-Acetamido-2,4,6-trideoxy-4,6-difluoro-D-glucopyranose (64)

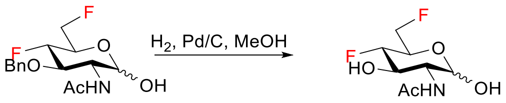

Compound **52** (90 mg, 0.29 mmol) was hydrogenated overnight according to the general procedure for debenzylation using palladium on carbon (80 mg) in methanol (6 mL) to afford the product **64** (64 mg, 100%) as a white crystalline solid, mp 206–209 °C (dec, EtOH/EtOAc). R_f 0.10 (EtOAc). NMR signals of the α-anomer: ^{1}H NMR (MeOH-d_4, 400 MHz, ^{1}H {^{19}F}, H-H COSY): δ 5.09 (t, 1H, J = 3.3 Hz, H-1), 4.61 (dddd, 1H, J = 47.5, 10.4, 3.9, 1.7 Hz, H-6´), 4.55 (dddd, 1H, J = 48.1, 10.4, 1.8, 1.4 Hz, H-6), 4.29 (ddd, 1H, J = 50.6, 10.1, 8.3 Hz, H-4), 4.11 (ddddd, 1H, J = 26.6, 10.1, 4.1, 3.9, 1.8 Hz, H-5), 3.97 (ddd, 1H, J = 14.8, 10.8, 8.3 Hz, H-3), 3.89 (dd, 1H, J = 10.8, 3.3 Hz, H-2), 1.99 (s, 3H, *Me*). ^{13}C{^{1}H} NMR (MeOH-d_4, 101 MHz, HSQC): δ 173.7 (CO), 92.5 (d, ^4J = 1.5 Hz, C-1), 90.6 (dd, ^1J = 181.2 Hz, ^3J = 7.4 Hz, C-4), 82.7 (d, ^1J = 172.5 Hz, C-6), 70.5 (d, ^2J = 18.5 Hz, C-3), 69.2 (dd, ^2J = 23.7, 18.2 Hz, C-5), 55.3 (d, ^3J = 8.0 Hz, C-2), 22.6 (Me). ^{19}F NMR (MeOH-d_4, 376 MHz): –199.99 (dddd, ^2J = 50.6 Hz, ^3J = 14.8, 4.1 Hz, ^5J = 3.3 Hz, F-4), –238.52 (ddd, ^2J = 48.1, 47.5 Hz, ^3J = 26.6 Hz, F-6). HRMS-APCI (*m/z*):

$[M + H]^+$ calcd for $C_8H_{14}F_2NO_4$, 226.0885; found, 226.0884. NMR signals of the β-anomer could not be observed in intensity sufficient for characterization within 12 h after sample preparation.

2-Acetamido-2,3,4-trideoxy-3,4-difluoro-D-glucopyranose (65)

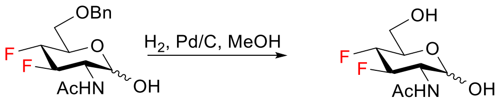

53 **65**

Compound **53** (85 mg, 0.27 mmol) was hydrogenated for 4 days according to general procedure for debenzylation using palladium on carbon (added in 3 portions, 180 mg in total) in methanol (6 mL) to afford the product **65** (55 mg, 90%) as a white crystalline solid, mp 196–199 °C (dec, EtOH/EtOAc) R_f 0.07 (EtOAc). NMR data for the α-anomer: ^{1}H NMR (MeOH-d_4, 400 MHz, ^{1}H {^{19}F}, H-H COSY): δ 5.12 (dt, 1H, J = 3.6, 3.5 Hz, H-1), 4.82 (dddd, 1H, J = 54.3, 16.1, 10.5, 8.2 Hz, H-3), 4.59 (dddd, 1H, J = 51.7, 15.3, 9.9, 8.2 Hz, H-4), 4.12 (ddd, 1H, J = 10.9, 10.5, 3.6 Hz, H-2), 3.99 (dddd, 1H, J = 9.9, 4.8, 4.0, 2.4 Hz, H-5), from ^{1}H {^{19}F} 3.79 (dd, 1H, J = 12.4, 2.4 Hz, H-6), from ^{1}H {^{19}F} 3.74 (dd, 1H, J = 12.4, 4.1 Hz, H-6´), 2.00 (s, 3H, *Me*). ^{13}C{^{1}H} NMR (MeOH-d_4, 101 MHz, HSQC): δ 173.5 (CO), 92.7 (dd, ^3J = 9.2 Hz, ^4J = 1.3 Hz, C-1), 91.7 (dd, ^1J = 184.8 Hz, ^2J = 18.9 Hz, C-3), 89.0 (dd, ^1J = 182.3 Hz, ^2J = 17.7 Hz, C-4), 70.1 (dd, ^2J = 23.7 Hz, ^3J = 6.2 Hz, C-5), 61.4 (C-6), 54.0 (dd, ^2J = 16.5 Hz, ^3J = 7.4 Hz, C-2), 22.5 (Me). ^{19}F NMR (MeOH-d_4, 376 MHz): –200.68 (ddddd, ^2J = 51.7 Hz, ^3J = 16.1, 13.7, 4.8 Hz, ^5J = 3.5 Hz, F-4), –201.65 (ddddd, ^2J = 54.3 Hz, ^3J = 15.3, 13.7, 10.9 Hz, ^4J = 3.5 Hz, F-3). Resolved signals for the β-anomer: ^{1}H {^{19}F} NMR (MeOH-d_4, 400 MHz, H-H COSY): δ 4.42 (d, 1H, J = 8.5 Hz, H-1), 3.84 (dd, 1H, J = 12.4, 2.2 Hz, H-6), 3.72 (dd, 1H, J = 12.4, 4.8 Hz, H-6´), 3.51 (ddd, 1H, J = 9.8, 4.8, 2.2 Hz, H-5). ^{13}C{^{1}H} NMR (MeOH-d_4, 101 MHz, HSQC): δ 96.1 (d, ^3J = 11.3 Hz, C-1), 74.1 (dd, ^2J = 23.5 Hz, ^3J = 7.2 Hz, C-5), 57.0 (dd, ^2J = 17.1 Hz, ^3J = 7.7 Hz, C-2). 22.8 (Me). ^{19}F NMR (MeOH-d_4, 376 MHz): –196.42 (dt, ^2J = 57.8 Hz, ^3J = 15.0 Hz, F-4), –202.68 (m, F-3). HRMS-APCI (*m/z*): $[M + H]^+$ calcd for $C_8H_{14}F_2NO_4$, 226.0885; found, 226.0888.

2-Acetamido-2,3-dideoxy-3-fluoro-D-galactopyranose (66)

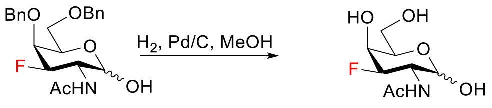

54 **66**

Compound **54** (125 mg, 0.31 mmol) was hydrogenated overnight according to the general procedure for debenzylation using palladium on carbon (70 mg) in methanol (9 mL) to afford the product **66** (65 mg, 94%) as a white foam, which slowly crystallized over a few days into white crystalline material, mp 159–161 °C (dec, EtOH/MTBE). R_f 0.12 (EtOAc/MeOH 10:1). NMR data for the α-anomer: ^{1}H NMR (MeOH-d_4, 400 MHz, ^{1}H {^{19}F}, H-H COSY): δ 5.15 (dd, 1H, J = 4.6, 3.7 Hz, H-1), 4.70 (ddd, 1H, J = 49.1, 10.8, 3.2 Hz, H-3), 4.52 (ddd, 1H, J =

10.8, 9.7, 3.7 Hz, H-2), 4.16 (ddd, 1H, J = 7.7, 3.2, 1.2 Hz, H-4), 4.04 (tdd, 1H, J = 6.2, 2.0, 1.2 Hz, H-5), 3.77–3.71 (m, 2H, H-6), 1.99 (s, 3H, *Me*). $^{13}C\{^{1}H\}$ NMR (MeOH-d_4, 101 MHz, HSQC): δ 173.6 (CO), 93.2 (d, ^{3}J = 9.5 Hz, C-1), 90.6 (d, ^{1}J = 185.0 Hz, C-3), 71.1 (d, ^{3}J = 6.1 Hz, C-5), 68.4 (d, ^{2}J = 16.6 Hz, C-4), 62.3 (d, ^{4}J = 2.9 Hz, C-6), 50.4 (d, ^{2}J = 17.2 Hz, C-2), 22.6 (Me). ^{19}F NMR (MeOH-d_4, 376 MHz): –204.16 (ddddd, ^{2}J = 49.1 Hz, ^{3}J = 9.7, 7.7 Hz, ^{4}J = 4.6, 2.0 Hz). NMR data for the β-anomer: ^{1}H NMR (MeOH-d_4, 400 MHz, ^{1}H {^{19}F}, H-H COSY): δ 4.59 (d, 1H, J = 8.4 Hz, H-1), 4.51 (ddd, 1H, J = 48.3, 10.6, 3.3 Hz, H-3), from ^{1}H {^{19}F} 4.14 (dd, 1H, J = 10.6, 8.4 Hz, H-2), 4.09 (ddd, 1H, J = 6.7, 3.2, 1.9 Hz, H-4), 3.77–3.71 (m, 2H, H-6), 3.50 (tdd, 1H, J = 5.7, 1.9, 1.5 Hz, H-5), 1.99 (*Me*). $^{13}C\{^{1}H\}$ NMR (MeOH-d_4, 101 MHz, HSQC): δ 174.0 (CO), 96.8 (d, ^{3}J = 10.5 Hz, C-1), 92.5 (d, ^{1}J = 187.0 Hz, C-3), 75.5 (d, ^{3}J = 7.0 Hz, C-5), 67.6 (d, ^{2}J = 16.6 Hz, C-4), 62.1 (d, ^{4}J = 3.4 Hz, C-6), 54.1 (d, ^{2}J = 17.5 Hz, C-2), 22.9 (Me). ^{19}F NMR (MeOH-d_4, 376 MHz): –199.96 (dddd, ^{2}J = 48.3 Hz, ^{3}J = 10.0, 6.7 Hz, ^{4}J = 1.9 Hz). HRMS-APCI (*m/z*): $[M + H]^+$ calcd for $C_8H_{15}FNO_5$, 224.0928; found, 224.0927.

2-Acetamido-2,3,6-trideoxy-3,6-difluoro-D-galactopyranose (67)

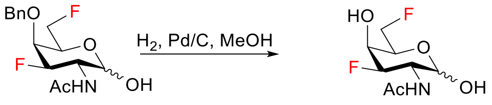

55 **67**

Compound **55** (66 mg, 0.21 mmol) was hydrogenated overnight according to general procedure for debenzylation using palladium on carbon (70 mg) in methanol (9 mL) to afford the product **67** (45 mg, 95%) as a white crystalline solid, mp 169–172 °C (dec, EtOH). R_f 0.11 (EtOAc). NMR signals of the α-anomer: ^{1}H NMR (MeOH-d_4, 400 MHz, ^{1}H {^{19}F}, H-H COSY): δ 5.17 (dd, 1H, J = 3.6, 5.0 Hz, H-1), 4.72 (ddd, 1H, J = 49.4, 10.7, 3.1 Hz, H-3), from ^{1}H {^{19}F-6} 4.60 (dd, 1H, J = 9.5, 5.0 Hz, H-6´), 4.52 (dddd, 1H, J = 47.9, 9.5, 6.9, 1.2 Hz, H-6), 4.52 (ddd, 1H, J = 10.7, 10.3, 3.5 Hz, H-2), 4.28 (dddd, 1H, J = 14.1, 6.9, 5.0, 1.3 Hz, H-5), 4.15 (ddd, 1H, J = 7.7, 3.1, 1.3 Hz, H-4), 1.99 (s, 3H, *Me*). $^{13}C\{^{1}H\}$ NMR (MeOH-d_4, 101 MHz, HSQC): δ 173.6 (CO), 93.2 (d, ^{3}J = 9.4 Hz, C-1), 90.1 (dd, ^{1}J = 185.0 Hz, ^{4}J = 1.3 Hz, C-3), 83.7 (dd, ^{1}J = 167.0, ^{4}J = 3.5 Hz, C-6), 69.6 (dd, ^{2}J = 22.6 Hz, ^{3}J = 6.8 Hz, C-5), 68.0 (dd, ^{2}J = 17.2 Hz, ^{3}J = 6.8 Hz, C-4), 50.2 (d, ^{2}J = 17.3 Hz, C-2), 22.6 (Me). ^{19}F NMR (MeOH-d_4, 376 MHz): –204.93 (ddddd, ^{2}J = 49.4 Hz, ^{3}J = 10.3, 7.7 Hz, ^{4}J = 5.0 Hz, ^{5}J = 2.5 Hz, F-3), –233.55 (dddd, ^{2}J = 47.9, 46.1 Hz, ^{3}J = 14.1 Hz, ^{5}J = 2.5 Hz, F-6). HRMS-APCI (*m/z*): $[M + H]^+$ calcd for $C_8H_{14}F_2NO_4$, 226.0885; found, 226.0880. NMR signals of the β-anomer could not be observed in intensity sufficient for characterization within 12 h after sample preparation.

2-Acetamido-2,4-dideoxy-4-fluoro-D-galactopyranose (68)

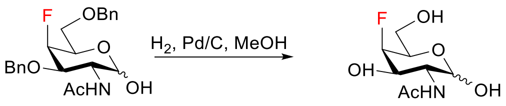

Compound **56** (90 mg, 0.22 mmol) was hydrogenated overnight according to general procedure for debenzylation using palladium on carbon (80 mg) in methanol (6 mL) to afford the known product **68** [5] (48 mg, 96%) as a white crystalline solid, mp 210–213 °C (dec, EtOH, ref 5 gives 205–209 °C). R_f 0.11 (EtOAc/MeOH 10:1). NMR data for the α-anomer: ^{1}H NMR (MeOH-d_4, 400 MHz, ^{1}H {^{19}F}, H-H COSY): δ 5.14 (d, 1H, J = 3.5 Hz, H-1), from ^{1}H {^{19}F} 4.80 (d, 1H, J = 2.6 Hz, H-4), 4.20 (dd, 1H, J = 11.1, 3.5 Hz, H-2), 4.07 (dt, 1H, J = 30.3, 6.8 Hz, H-5), 3.91 (ddd, 1H, J = 28.9, 11.1, 2.6 Hz, H-3), 3.74–3.62 (m, 2H, H-6), 1.99 (s, 3H, *Me*). ^{13}C{^{1}H} NMR (MeOH-d_4, 101 MHz, HSQC): δ 173.9 (CO), 92.3 (C-1), 90.5 (d, ^1J = 180.1 Hz, C-4), 70.6 (d, ^2J = 18.1 Hz, C-5), 68.0 (d, ^2J = 19.1 Hz, C-3), 61.3 (d, ^3J = 6.0 Hz, C-6), 52.2 (d, ^3J = 2.9 Hz, C-2), 22.6 (Me). ^{19}F NMR (MeOH-d_4, 376 MHz): δ −224.66 (ddd, ^2J = 50.5 Hz, ^3J = 30.3, 28.9 Hz). Resolved signals for the β-anomer: ^{1}H NMR (MeOH-d_4, 400 MHz, ^{1}H {^{19}F}, H-H COSY): δ 4.72 (dd, 1H, J = 50.8, 2.5 Hz, H-4), 4.63 (d, 1H, J = 8.1 Hz, H-1). ^{13}C{^{1}H} NMR (MeOH-d_4, 101 MHz, HSQC): δ 97.1 (C-1), 89.5 (d, ^1J = 180.5 Hz, C-4), 56.1 (C-2). ^{19}F NMR (MeOH-d_4, 376 MHz): δ −222.05 (dt, ^2J = 50.8 Hz, ^3J = 28.4 Hz).

2-Acetamido-2,4,6-trideoxy-4,6-difluoro-D-galactopyranose (69)

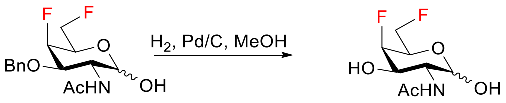

Compound **57** (48 mg, 0.15 mmol) was hydrogenated overnight according to the general procedure for debenzylation using palladium on carbon (70 mg) in methanol (9 mL) to afford the known product **69** [6] (31 mg, 92%) as a white crystalline solid, mp 206–209 °C (dec, EtOH/MTBE, ref 6 gives 205–206 °C (dec)). R_f 0.06 (EtOAc). NMR data for the α-anomer: ^{1}H NMR (MeOH-d_4, 400 MHz, ^{1}H {^{19}F}, H-H COSY): δ 5.16 (d, 1H, J = 3.5 Hz, H-1), 4.77 (dd, 1H, J = 51.0, 2.6 Hz, H-4), 4.59 (ddd, 1H, J = 46.2, 9.4, 5.5 Hz, H-6´), 4.49 (dddd, 1H, J = 47.3, 9.4, 6.8, 1.3 Hz, H-6), 4.33 (dddd, 1H, J = 30.3, 12.7, 6.8, 5.5 Hz, H-5), 4.21 (dd, 1H, J = 11.1, 3.5 Hz, H-2), 3.94 (ddd, 1H, J = 28.9, 11.1, 2.6 Hz, H-3), 2.00 (s, 3H, *Me*). ^{13}C{^{1}H} NMR (MeOH-d_4, 101 MHz, HSQC): δ 173.9 (CO), 92.8 (Hz, C-1), 90.5 (dd, ^1J = 180.1 Hz, ^3J = 5.9 Hz, C-4), 82.7 (dd, ^1J = 168.1 Hz, ^3J = 6.5 Hz, C-6), 68.8 (dd, ^2J = 23.1, 17.7 Hz, C-5), 67.6 (d, ^2J = 18.8 Hz, C-3), 52.0 (d, ^3J = 2.7 Hz, C-2), 22.6 (Me). ^{19}F NMR (MeOH-d_4, 376 MHz): −224.17 (ddd, ^2J = 51.0 Hz, ^3J = 30.3, 28.9 Hz, F-4), −234.16 (ddd, ^2J = 47.3, 46.2 Hz, ^3J = 12.7 Hz, F-6). Resolved signal for the β-anomer: ^{1}H NMR (MeOH-d_4, 400 MHz, ^{1}H {^{19}F}, H-H COSY): δ 4.67 (d, 1H, J = 8.1 Hz, H-1), 3.95–3.71 (m, 3H, H-2, H-3, H-5). ^{13}C{^{1}H} NMR (MeOH-d_4, 101 MHz, HSQC): δ 174.4 (CO), 97.0 (C-1), 89.5 (dd, ^1J = 181.0 Hz, ^3J = 6.1 Hz, C-4), 82.4 (dd, ^1J = 168.9 Hz, ^3J = 6.1 Hz, C-6), 73.1 (dd, ^2J = 22.8, 17.7 Hz, C-5), 71.3 (d, ^2J = 18.7

Hz, C-3), 55.9 (d, ^3J = 1.6 Hz, C-2), 22.9 (Me). ^{19}F NMR (MeOH-d_4, 376 MHz): −221.72 (dt, ^2J = 51.6 Hz, ^3J = 27.9 Hz, F-4), −233.90 (ddd, ^2J = 47.0, 46.0 Hz, ^3J = 12.7 Hz, F-6). HRMS-APCI (*m/z*): [M + H]$^+$ calcd for $C_8H_{14}F_2NO_4$, 226.0885; found, 226.0884.

2-Acetamido-2,3,4-trideoxy-3,4-difluoro-D-galactopyranose (70)

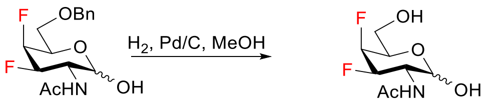

58 **70**

Compound **58** (105 mg, 0.33 mmol) was hydrogenated for 4 days according to general procedure for debenzylation using palladium on carbon (added in 2 portions, 120 mg in total) in methanol (8 mL) to afford the product **70** (75 mg, 100%) as a white crystalline solid, mp 150–151 °C (dec, EtOH/MTBE). R_f 0.07 (EtOAc). NMR data for the α-anomer: ^{1}H NMR (MeOH-d_4, 400 MHz, ^{1}H {^{19}F}, H-H COSY): δ 5.16 (dd, 1H, *J* = 5.3, 3.5 Hz, H-1), 5.01 (ddd, 1H, *J* = 51.8, 8.2, 2.7 Hz, H-4), 4.75 (dddd, 1H, *J* = 47.7, 26.8, 11.0, 2.7 Hz, H-3), 4.53 (ddd, 1H, *J* = 11.0, 9.2, 3.5 Hz, H-2), 4.08 (dddd, 1H, *J* = 29.0, 7.0, 6.7, 1.6 Hz, H-5), 3.73 (dd, 1H, *J* = 11.0, 7.0 Hz, H-6´), 3.66 (dddd, 1H, *J* = 11.0, 6.7, 1.7, 1.2 Hz, H-6), 1.99 (s, 3H, *Me*). ^{13}C{^{1}H} NMR (MeOH-d_4, 101 MHz, HSQC, HMBC): δ 173.7 (CO), 93.1 (d, ^3J = 9.2 Hz, C-1), 88.3 (dd, ^1J = 188.7 Hz, ^2J = 18.3 Hz, C-3), 87.8 (dd, ^1J = 182.8 Hz, ^2J = 16.1 Hz, C-4), 70.2 (dd, ^2J = 18.1 Hz, ^3J = 5.1 Hz, C-5), 60.9 (dd, ^3J = 5.9, ^4J = 2.4 Hz, C-6), 50.6 (dd, ^2J = 17.4 Hz, ^3J = 2.7 Hz, C-2), 22.5 (Me). ^{19}F NMR (MeOH-d_4, 376 MHz): −202.42 (ddddd, ^2J = 47.7 Hz, ^3J = 15.0, 9.2, 8.2 Hz, ^4J = 5.3 Hz, F-3), −220.90 (m, F-4). Resolved signals for the β-anomer: ^{1}H NMR (MeOH-d_4, 400 MHz, ^{1}H {^{19}F}, H-H COSY): 4.95 (ddd, 1H, *J* = 52.3, 7.0, 2.7 Hz, H-4), 4.67 (dddd, 1H, *J* = 46.5, 26.8, 10.9, 2.7 Hz, H-3), 4.68 (d, 1H, *J* = 8.3 Hz, H-1). ^{19}F NMR (MeOH-d_4, 376 MHz): −198.00 (dddd, ^2J = 46.5 Hz, ^3J = 15.0, 11.0, 7.0 Hz, F-3), −218.46 (dddd, ^2J = 52.3 Hz, ^3J = 26.8, 26.5, 15.0 Hz, F-4). HRMS-APCI (*m/z*): [M + H]$^+$ calcd for $C_8H_{14}F_2NO_4$, 226.0885; found, 226.0887.

2-Acetamido-2,6-dideoxy-6-fluoro-D-glucopyranose (71)

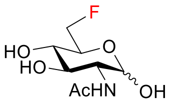

71

Compound **71** was prepared according to ref 7.

2-Acetamido-2,6-dideoxy-6-fluoro-α-D-galactopyranose (72)

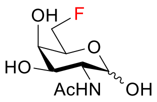

72

Compound **72** was prepared following the published procedure [8], mp 168–171 °C (EtOH), R_f 0.25 (EtOAc/MeOH 20:3). Since NMR data are incomplete in ref 8, we report them here: ^{1}H NMR (MeOH-d_4, 400 MHz, ^{1}H {^{19}F}, H-H COSY): δ 5.14 (d, 1H, *J* = 3.6 Hz, H-1), 4.58 (ddd, 1H, *J* = 46.4, 9.5, 4.8 Hz, H-6), 4.50 (ddd, 1H, *J* = 48.1, 9.5, 7.0 Hz, H-6´), 4.27 (dddd, 1H, *J* = 14.6, 7.0, 4.8, 1.3 Hz, H-5), 4.22 (dd, 1H, *J* = 10.9, 3.6 Hz, H-2), 3.88 (dd, 1H, *J* = 3.3, 1.3 Hz, H-4), 3.83 (dd, 1H, *J* = 10.9, 3.3 Hz, H-3), 1.99 (s, 3H, *Me*). ^{13}C{^{1}H} NMR (MeOH-d_4, 101 MHz, HSQC): δ 174.0 (CO), 92.9 (C-1), 84.1 (d, ^1J = 166.8 Hz, C-6), 70.2 (d, ^2J = 13.0 Hz, C-5), 70.0 (d, ^3J = 2.5 Hz, C-4), 69.3 (d, ^4J = 1.1 Hz, C-3), 51.9 (C-2), 22.7 (*Me*). ^{19}F NMR (MeOH-d_4, 376 MHz): –233.19 (ddd, ^2J = 48.1, 46.4 Hz, ^3J = 14.6 Hz). Resolved signals for β-anomer: ^{19}F NMR (MeOH-d_4, 376 MHz): –232.96 (ddd, ^2J = 48.0, 46.5 Hz, ^3J = 13.6 Hz). HRMS-APCI (*m/z*): [M + H]$^+$ calcd for $C_8H_{15}FNO_5$, 224.0928; found, 224.0928.

Table S1.

The values of $J_{\text{H-F}}$, $J_{\text{C-F}}$ and $J_{\text{F-F}}$ coupling constants extracted from NMR spectra of the target fluoro analogs. (See also refs 9–11 for the discussion of the relationship between the values and the spatial orientation of the coupled nuclei)

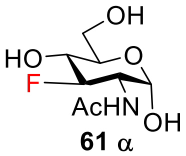<br>**61** α	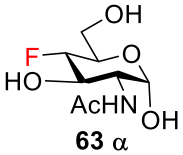<br>**63** α	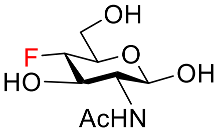<br>**63** β	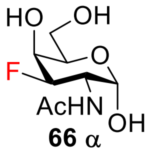<br>**66** α
$^{1}J_{\text{C3-F}}$ = 182.3 Hz<br>$^{2}J_{\text{C2-F}}$ = 16.4 Hz<br>$^{2}J_{\text{C4-F}}$ = 17.5 Hz<br>$^{3}J_{\text{C1-F}}$ = 9.8 Hz<br>$^{3}J_{\text{C5-F}}$ = 7.1 Hz<br>$^{4}J_{\text{C6-F}}$ = 1.4 Hz	$^{1}J_{\text{C4-F}}$ = 180.0 Hz<br>$^{2}J_{\text{C3-F}}$ = 19.0 Hz<br>$^{2}J_{\text{C5-F}}$ = 23.8 Hz<br>$^{3}J_{\text{C2-F}}$ = 8.2 Hz	$^{1}J_{\text{C4-F}}$ = 180.9 Hz<br>$^{2}J_{\text{C3-F}}$ = 18.6 Hz<br>$^{2}J_{\text{C5-F}}$ = 24.2 Hz<br>$^{3}J_{\text{C2-F}}$ = 8.6 Hz	$^{1}J_{\text{C3-F}}$ = 185.0 Hz<br>$^{2}J_{\text{C2-F}}$ = 17.2 Hz<br>$^{2}J_{\text{C4-F}}$ = 16.6 Hz<br>$^{3}J_{\text{C1-F}}$ = 9.5 Hz<br>$^{3}J_{\text{C5-F}}$ = 6.1 Hz<br>$^{4}J_{\text{C6-F}}$ = 2.9 Hz
$^{2}J_{\text{H3-F}}$ = 53.8 Hz<br>$^{3}J_{\text{H4-F}}$ = 15.0 Hz<br>$^{3}J_{\text{H2-F}}$ = 10.5 Hz<br>$^{4}J_{\text{H1-F}}$ = 3.8 Hz<br>$^{4}J_{\text{H5-F}}$ = 1.1 Hz	$^{2}J_{\text{H4-F}}$ = 50.9 Hz	$^{2}J_{\text{H4-F}}$ = 50.8 Hz<br>$^{3}J_{\text{H3-F}}$ = 15.6 Hz<br>$^{3}J_{\text{H5-F}}$ = 2.5 Hz	$^{2}J_{\text{H3-F}}$ = 49.1 Hz<br>$^{3}J_{\text{H2-F}}$ = 9.7 Hz<br>$^{3}J_{\text{H4-F}}$ = 7.7 Hz<br>$^{4}J_{\text{H1-F}}$ = 4.6 Hz<br>$^{4}J_{\text{H5-F}}$ = 2.0 Hz

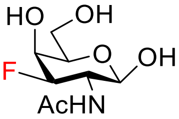<br>**66** β	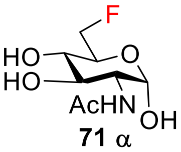<br>**71** α	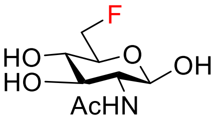<br>**71** β	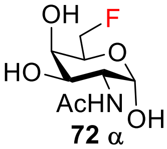<br>**72** α
$^{1}J_{\text{C3-F}}$ = 187.0 Hz<br>$^{2}J_{\text{C2-F}}$ = 17.5 Hz<br>$^{2}J_{\text{C4-F}}$ = 16.6 Hz<br>$^{3}J_{\text{C1-F}}$ = 10.5 Hz<br>$^{3}J_{\text{C5-F}}$ = 7.0 Hz<br>$^{4}J_{\text{C6-F}}$ = 3.4 Hz	$^{1}J_{\text{C6-F6}}$ = 171.3 Hz<br>$^{2}J_{\text{C5-F6}}$ = 17.9 Hz<br>$^{3}J_{\text{C4-F6}}$ = 7.0 Hz<br>$^{4}J_{\text{C3-F6}}$ = 0.7 Hz		$^{1}J_{\text{C6-F6}}$ = 166.8 Hz<br>$^{2}J_{\text{C5-F6}}$ = 13.0 Hz<br>$^{3}J_{\text{C4-F6}}$ = 2.5 Hz<br>$^{4}J_{\text{C3-F6}}$ = 1.1 Hz
$^{2}J_{\text{H3-F}}$ = 48.3 Hz<br>$^{3}J_{\text{H2-F}}$ = 10.0 Hz<br>$^{3}J_{\text{H4-F}}$ = 6.7 Hz<br>$^{4}J_{\text{H5-F}}$ = 1.9 Hz	$^{2}J_{\text{H6-F6}}$ = 48.2 Hz<br>$^{2}J_{\text{H6'-F6}}$ = 48.2 Hz<br>$^{3}J_{\text{H5-F6}}$ = 27.1 Hz	$^{2}J_{\text{H6-F6}}$ = 48.2 Hz<br>$^{2}J_{\text{H6'-F6}}$ = 48.2 Hz<br>$^{3}J_{\text{H5-F6}}$ = 24.6 Hz	$^{2}J_{\text{H6-F6}}$ = 46.4 Hz<br>$^{2}J_{\text{H6'-F6}}$ = 48.1 Hz<br>$^{3}J_{\text{H5-F6}}$ = 14.6 Hz

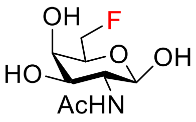<br>**72** β	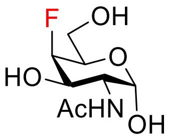<br>**68** α	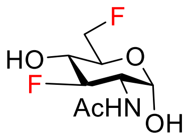<br>**62** α	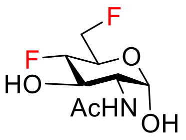<br>**64** α
	$^{1}J_{C4\text{-}F}$ = 180.1 Hz<br>$^{2}J_{C3\text{-}F}$ = 19.1 Hz<br>$^{2}J_{C5\text{-}F}$ = 18.1 Hz<br>$^{3}J_{C6\text{-}F}$ = 6.0 Hz<br>$^{3}J_{C2\text{-}F}$ = 2.9 Hz	$^{1}J_{C3\text{-}F3}$ = 182.6 Hz<br>$^{2}J_{C2\text{-}F3}$ = 16.5 Hz<br>$^{2}J_{C4\text{-}F3}$ = 18.2 Hz<br>$^{3}J_{C1\text{-}F3}$ = 9.8 Hz<br>$^{3}J_{C5\text{-}F3}$ = 7.8 Hz<br>$^{4}J_{C6\text{-}F3}$ = 1.6 Hz<br><br>$^{1}J_{C6\text{-}F6}$= 171.8 Hz<br>$^{2}J_{C5\text{-}F6}$ = 18.0 Hz<br>$^{3}J_{C4\text{-}F6}$ = 7.6 Hz<br>$^{4}J_{C3\text{-}F6}$ = 0.9 Hz	$^{1}J_{C4\text{-}F4}$ = 181.2 Hz<br>$^{2}J_{C3\text{-}F4}$ = 18.5 Hz<br>$^{2}J_{C5\text{-}F4}$ = 23.7 Hz<br>$^{3}J_{C2\text{-}F4}$ = 8.0 Hz<br>$^{4}J_{C1\text{-}F4}$ = 1.5 Hz<br><br>$^{1}J_{C6\text{-}F6}$ = 172.5 Hz<br>$^{2}J_{C5\text{-}F6}$ = 18.2 Hz<br>$^{3}J_{C4\text{-}F6}$ = 7.4 Hz
$^{2}J_{H6\text{ or }H6'\text{-}F6}$ = 48.0 Hz<br>$^{2}J_{H6\text{ or }H6'\text{-}F6}$ = 46.5 Hz<br>$^{3}J_{H5\text{-}F6}$ = 13.6 Hz	$^{2}J_{H4\text{-}F}$ = 50.5 Hz<br>$^{3}J_{H3\text{-}F}$ = 28.9 Hz<br>$^{3}J_{H5\text{-}F}$ = 30.3 Hz	$^{2}J_{H3\text{-}F3}$ = 53.4 Hz<br>$^{3}J_{H4\text{-}F3}$ = 14.7 Hz<br>$^{3}J_{H2\text{-}F3}$ = 10.6 Hz<br>$^{4}J_{H1\text{-}F3}$ = 3.8 Hz<br><br>$^{2}J_{H6\text{-}F6}$ = 48.6 Hz<br>$^{2}J_{H6'\text{-}F6}$ = 47.5 Hz<br>$^{3}J_{H5\text{-}F6}$ = 26.7 Hz	$^{2}J_{H4\text{-}F4}$ = 50.6 Hz<br>$^{3}J_{H3\text{-}F4}$ = 14.8 Hz<br>$^{3}J_{H5\text{-}F4}$ = 4.1 Hz<br>$^{5}J_{H1\text{-}F4}$ = 3.3 Hz<br><br>$^{2}J_{H6\text{-}F6}$ = 48.1 Hz<br>$^{3}J_{H6'\text{-}F6}$ = 47.5 Hz<br>$^{3}J_{H5\text{-}F6}$ = 26.6 Hz

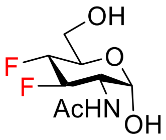<br>**65** α	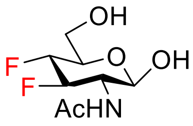<br>**65** β	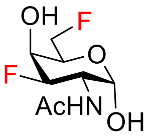<br>**67** α	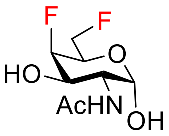<br>**69** α
$^{1}J_{\text{C3-F3}}$ = 184.8 Hz	$^{2}J_{\text{C5-F4}}$ = 23.5 Hz	$^{1}J_{\text{C3-F3}}$ = 185 Hz	$^{1}J_{\text{C4-F4}}$ = 180.1 Hz
$^{2}J_{\text{C4-F3}}$ = 17.7 Hz	$^{3}J_{\text{C2-F4}}$ = 7.7 Hz	$^{2}J_{\text{C2-F3}}$ = 17.3 Hz	$^{2}J_{\text{C3-F4}}$ = 18.8 Hz
$^{2}J_{\text{C2-F3}}$ = 16.5 Hz		$^{2}J_{\text{C4-F3}}$ = 17.2 Hz	$^{2}J_{\text{C5-F4}}$ = 17.7 Hz
$^{3}J_{\text{C5-F3}}$ = 6.2 Hz	$^{2}J_{\text{C2-F3}}$ = 17.1 Hz	$^{3}J_{\text{C1-F3}}$ = 9.4 Hz	$^{3}J_{\text{C2-F4}}$ = 2.7 Hz
$^{3}J_{\text{C1-F3}}$ = 9.2 Hz	$^{3}J_{\text{C1-F3}}$ = 11.3 Hz	$^{3}J_{\text{C5-F3}}$ = 6.8 Hz	$^{3}J_{\text{C6-F4}}$ = 6.5 Hz
	$^{3}J_{\text{C5-F3}}$ = 7.2 Hz	$^{4}J_{\text{C6-F3}}$ = 3.5 Hz	
$^{1}J_{\text{C4-F4}}$= 182.3 Hz			$^{1}J_{\text{C6-F6}}$ = 168.1 Hz
$^{2}J_{\text{C3-F4}}$ = 18.9 Hz		$^{1}J_{\text{C6-F6}}$ = 167 Hz	$^{2}J_{\text{C5-F6}}$ = 23.1 Hz
$^{2}J_{\text{C5-F4}}$ = 23.7 Hz		$^{2}J_{\text{C5-F6}}$ = 22.6 Hz	$^{3}J_{\text{C4-F6}}$ = 5.9 Hz
$^{3}J_{\text{C2-F4}}$ = 7.4 Hz		$^{3}J_{\text{C4-F6}}$ = 6.8 Hz	
$^{4}J_{\text{C1-F4}}$ = 1.3 Hz		$^{4}J_{\text{C3-F6}}$ = 1.3 Hz	
$^{2}J_{\text{H3-F3}}$ = 54.3 Hz	$^{2}J_{\text{H4-F4}}$ = 57.8 Hz	$^{2}J_{\text{H3-F3}}$ = 49.4 Hz	$^{2}J_{\text{H4-F4}}$ = 51.0 Hz
$^{3}J_{\text{H4-F3}}$ = 15.3 Hz	$^{3}J_{\text{H3-F4}}$ = 15.0 Hz	$^{3}J_{\text{H2-F3}}$ = 10.3 Hz	$^{3}J_{\text{H5-F4}}$ = 30.3 Hz
$^{3}J_{\text{H2-F3}}$ = 10.9 Hz		$^{3}J_{\text{H4-F3}}$ = 7.7 Hz	$^{3}J_{\text{H3-F4}}$ = 28.9 Hz
$^{3}J_{\text{F4-F3}}$ = 13.7 Hz		$^{4}J_{\text{H1-F3}}$ = 5.0 Hz	
$^{4}J_{\text{H1-F3}}$ = 3.5 Hz		$^{5}J_{\text{F6-F3}}$ = 2.5 Hz	$^{2}J_{\text{H6-F6}}$ = 47.3 Hz
			$^{2}J_{\text{H6´-F6}}$ = 46.2 Hz
$^{2}J_{\text{H4-F4}}$ = 51.7 Hz		$^{2}J_{\text{H6-F6}}$ = 47.9 Hz	$^{3}J_{\text{H5-F6}}$ = 12.7 Hz
$^{3}J_{\text{H3-F4}}$ = 16.1 Hz		$^{2}J_{\text{H6´-F6}}$ = 46.1 Hz	
$^{3}J_{\text{H5-F4}}$ = 4.8 Hz		$^{3}J_{\text{H5-F6}}$ = 14.1 Hz	
$^{3}J_{\text{F3-F4}}$ = 13.7 Hz		$^{5}J_{\text{F3-F6}}$ = 2.5 Hz	
$^{5}J_{\text{H1-F4}}$ = 3.5 Hz			

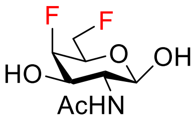 **69** β	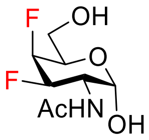 **70** α	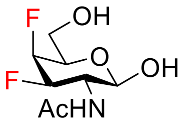 **70** β	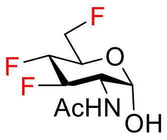 **59** α
$^{1}J_{\mathrm{C4\text{-}F4}}$ = 181.0 Hz	$^{1}J_{\mathrm{C3\text{-}F3}}$ = 188.7 Hz		$^{1}J_{\mathrm{C3\text{-}F3}}$ = 185.0 Hz
$^{2}J_{\mathrm{C3\text{-}F4}}$ = 18.7 Hz	$^{2}J_{\mathrm{C2\text{-}F3}}$ = 17.4 Hz		$^{2}J_{\mathrm{C2\text{-}F3}}$ = 16.6 Hz
$^{2}J_{\mathrm{C5\text{-}F4}}$ = 17.7 Hz	$^{2}J_{\mathrm{C4\text{-}F3}}$ = 16.1 Hz		$^{2}J_{\mathrm{C4\text{-}F}}$ = 18.3 Hz
$^{3}J_{\mathrm{C2\text{-}F4}}$ = 1.6 Hz	$^{3}J_{\mathrm{C1\text{-}F3}}$ = 9.2 Hz		$^{3}J_{\mathrm{C1\text{-}F3}}$ = 9.5 Hz
$^{3}J_{\mathrm{C6\text{-}F4}}$ = 6.1 Hz	$^{3}J_{\mathrm{C5\text{-}F3}}$ = 5.1 Hz		$^{3}J_{\mathrm{C5\text{-}F3}}$ = 7.0 Hz
	$^{4}J_{\mathrm{C6\text{-}F3}}$ = 2.4 Hz		
$^{1}J_{\mathrm{C6\text{-}F6}}$ = 168.9 Hz			$^{1}J_{\mathrm{C4\text{-}F4}}$ = 183.7 Hz
$^{2}J_{\mathrm{C5\text{-}F6}}$ = 22.8 Hz			$^{2}J_{\mathrm{C3\text{-}F4}}$ = 18.7 Hz
$^{3}J_{\mathrm{C4\text{-}F6}}$ = 6.1 Hz	$^{1}J_{\mathrm{C4\text{-}F4}}$ = 182.8 Hz		$^{2}J_{\mathrm{C5\text{-}F4}}$ = 23.2 Hz
	$^{2}J_{\mathrm{C3\text{-}F4}}$ = 18.3 Hz		$^{3}J_{\mathrm{C2\text{-}F4}}$ = 7.3 Hz
	$^{2}J_{\mathrm{C5\text{-}F4}}$ = 18.1 Hz		$^{4}J_{\mathrm{C1\text{-}F4}}$ = 1.5 Hz
	$^{3}J_{\mathrm{C2\text{-}F4}}$ = 2.7 Hz		
	$^{3}J_{\mathrm{C6\text{-}F4}}$ = 5.9 Hz		$^{1}J_{\mathrm{C6\text{-}F6}}$ = 173.4 Hz
			$^{2}J_{\mathrm{C5\text{-}F6}}$ = 18.5 Hz
			$^{3}J_{\mathrm{C4\text{-}F6}}$ = 8.1 Hz
			$^{4}J_{\mathrm{C3\text{-}F6}}$ = 0.9 Hz
$^{2}J_{\mathrm{H4\text{-}F4}}$ = 51.6 Hz	$^{2}J_{\mathrm{H3\text{-}F3}}$ = 47.7 Hz	$^{2}J_{\mathrm{H3\text{-}F3}}$ = 46.5Hz	$^{2}J_{\mathrm{H3\text{-}F3}}$ = 54.0 Hz
$^{3}J_{\mathrm{H5\text{-}F4}}$ = 27.9 Hz	$^{3}J_{\mathrm{H2\text{-}F3}}$ = 9.2 Hz	$^{3}J_{\mathrm{H2\text{-}F3}}$ = 11.0 Hz	$^{3}J_{\mathrm{F4\text{-}F3}}$ = 13.3 Hz
$^{3}J_{\mathrm{H3\text{-}F4}}$ = 27.9 Hz	$^{3}J_{\mathrm{H4\text{-}F3}}$ = 8.2 Hz	$^{3}J_{\mathrm{H4\text{-}F3}}$ = 7.0 Hz	$^{3}J_{\mathrm{H2\text{-}F3}}$ = 11.1 Hz
	$^{3}J_{\mathrm{F4\text{-}F3}}$ = 15.0 Hz	$^{3}J_{\mathrm{F4\text{-}F3}}$ = 15.0 Hz	$^{3}J_{\mathrm{H4\text{-}F3}}$ = 14.2 Hz
$^{2}J_{\mathrm{H6\ or\ H6'\text{-}F6}}$ = 47.0 Hz	$^{4}J_{\mathrm{H1\text{-}F3}}$ = 5.3 Hz		$^{4}J_{\mathrm{H1\text{-}F3}}$ = 3.6 Hz
$^{2}J_{\mathrm{H6\ or\ H6'\text{-}F6}}$ = 46.0 Hz		$^{2}J_{\mathrm{H4\text{-}F4}}$ = 52.3 Hz	
$^{3}J_{\mathrm{H5\text{-}F6}}$ = 12.7 Hz	$^{2}J_{\mathrm{H4\text{-}F4}}$ = 51.8 Hz	$^{3}J_{\mathrm{H3\text{-}F4}}$ = 26.8 Hz	$^{2}J_{\mathrm{H4\text{-}F4}}$ = 52.6 Hz
	$^{3}J_{\mathrm{H3\text{-}F4}}$ = 26.8 Hz	$^{3}J_{\mathrm{H5\text{-}F4}}$ = 26.5 Hz	$^{3}J_{\mathrm{F3\text{-}F4}}$ = 13.3 Hz
	$^{3}J_{\mathrm{H5\text{-}F4}}$ = 29.0 Hz	$^{3}J_{\mathrm{F3\text{-}F4}}$ = 15.0 Hz	$^{3}J_{\mathrm{H3\text{-}F4}}$ = 16.8 Hz
			$^{3}J_{\mathrm{H5\text{-}F4}}$ = 3.6 Hz
			$^{5}J_{\mathrm{H1\text{-}F4}}$ = 3.6 Hz
			$^{2}J_{\mathrm{H6\text{-}F6}}$ = 47.7 Hz
			$^{2}J_{\mathrm{H6'\text{-}F6}}$ = 47.7 Hz
			$^{3}J_{\mathrm{H5\text{-}F6}}$ = 26.7 Hz

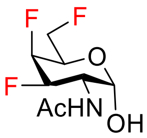 **60** α	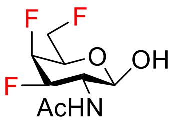 **60** β
$^{1}J_{\text{C3-F3}}$ = 189.9 Hz	$^{2}J_{\text{C2-F3}}$ = 17.4 Hz
$^{2}J_{\text{C2-F3}}$ = 17.3 Hz	$^{3}J_{\text{C1-F3}}$ = 10.4 Hz
$^{2}J_{\text{C4-F3}}$ = 16.5 Hz	$^{4}J_{\text{C6-F3}}$ = 2.3 Hz
$^{3}J_{\text{C1-F3}}$ = 9.3 Hz	$^{3}J_{\text{C5-F3}}$ = 6.0 Hz
$^{3}J_{\text{C5-F3}}$ = 5.7 Hz	
$^{4}J_{\text{C6-F3}}$ = 2.8 Hz	$^{3}J_{\text{C2-F4}}$ = 1.9 Hz
	$^{3}J_{\text{C6-F4}}$ = 5.8 Hz
$^{1}J_{\text{C4-F4}}$ = 182.0 Hz	$^{2}J_{\text{C5-F4}}$ = 17.5 Hz
$^{2}J_{\text{C3-F4}}$ = 18.2 Hz	
$^{2}J_{\text{C5-F4}}$ = 17.6 Hz	$^{1}J_{\text{C6-F6}}$ = 169.4 Hz
$^{3}J_{\text{C2-F4}}$ = 2.6 Hz	$^{2}J_{\text{C5-F6}}$ = 23.2 Hz
$^{3}J_{\text{C6-F4}}$ = 6.3 Hz	
$^{1}J_{\text{C6-F6}}$ = 168.5 Hz	
$^{2}J_{\text{C5-F6}}$ = 23.5 Hz	
$^{3}J_{\text{C4-F6}}$ = 6.6 Hz	
$^{4}J_{\text{C3-F6}}$ = 1.2 Hz	
$^{2}J_{\text{H3-F3}}$ = 47.3 Hz	$^{2}J_{\text{H3-F3}}$ = 46.3 Hz
$^{3}J_{\text{F4-F3}}$ = 14.4 Hz	$^{5}J_{\text{F6-F3}}$ = 1.8 Hz
$^{3}J_{\text{H2-F3}}$ = 10.0 Hz	$^{3}J_{\text{F4-F3}}$ = 14.5 Hz
$^{3}J_{\text{H4-F3}}$ = 8.2 Hz	$^{3}J_{\text{H2-F3}}$ = 8.6 Hz
$^{4}J_{\text{H1-F3}}$ = 5.3 Hz	$^{3}J_{\text{H4-F3}}$ = 8.6 Hz
$^{5}J_{\text{F6-F3}}$ = 1.8 Hz	
	$^{2}J_{\text{H4-F4}}$ = 52.6 Hz
$^{2}J_{\text{H4-F4}}$ = 52.5 Hz	$^{3}J_{\text{H3-F4}}$ = 28.1 Hz
$^{3}J_{\text{F3-F4}}$ = 14.4 Hz	$^{3}J_{\text{H5-F4}}$ = 25.5 Hz
$^{3}J_{\text{H3-F4}}$ = 26.7 Hz	$^{3}J_{\text{F3-F4}}$ = 14.5 Hz
$^{3}J_{\text{H5-F4}}$ = 29.3 Hz	
	$^{2}J_{\text{H6-F6}}$ = 46.5 Hz
$^{2}J_{\text{H6-F6}}$ = 47.1 Hz	$^{2}J_{\text{H6'-F6}}$ = 46.5 Hz
$^{2}J_{\text{H6'-F6}}$ = 46.2 Hz	$^{3}J_{\text{H5-F6}}$ = 11.8 Hz
$^{3}J_{\text{H5-F6}}$ = 13.0 Hz	$^{5}J_{\text{F3-F6}}$ = 1.8 Hz
$^{5}J_{\text{F3-F6}}$ = 1.8 Hz	

References

1. Horník, S.; Červenková Šťastná, L.; Cuřínová, P.; Sýkora, J.; Káňová, K.; Hrstka, R.; Císařová, I.; Dračínský, M.; Karban, J. *Beilstein J. Org. Chem.* **2016,** *12*, 750–759. https://doi.org/10.3762/bjoc.12.75
2. Kurfiřt, M.; Červenková Šťastná, L.; Dračínský, M.; Müllerová, M.; Hamala, V.; Cuřínová, P.; Karban, J. *J. Org. Chem.* **2019,** *84* (10), 6405–6431. https://doi.org/10.1021/acs.joc.9b00705
3. Hamala, V.; Červenková Šťastná, L.; Kurfiřt, M.; Cuřínová, P.; Dračínský, M.; Karban, J. *Org. Biomol. Chem.* **2020,** *18*, 5427–5434. https://doi.org/10.1039/D0OB01065K
4. Sharma, M.; Bernacki, R. J.; Hillman, M. J.; Korytnyk, W. *Carbohydr. Res.* **1993,** *240*, 85–93. https://doi.org/10.1016/0008-6215(93)84174-5
5. Berkin, A.; Szarek, W. A.; Kisilevsky, R. *Carbohydr. Res.* **2000**, *326*, 250–263. https://doi.org/10.1016/S0008-6215(00)00049-5
6. Sharma, M.; Bernacki, R. J.; Paul, B.; Korytnyk, W. *Carbohydr. Res.* **1990,** *198* (2), 205–221. https://doi.org/10.1016/0008-6215(90)84293-4
7. Morrison, Z. A.; Nitz, M. *Carbohydr. Res.* **2020,** *495*, 108071. https://doi.org/10.1016/j.carres.2020.108071
8. Sharma, M.; Potti, G. G.; Simmons, O. D.; Korytnyk, W. *Carbohydr. Res.* **1987,** *162*, 41–51. https://doi.org/10.1016/0008-6215(87)80199-4
9. Wray, V. *J. Chem. Soc., Perkin Trans. 2* **1976**, 1598–1605. https://doi.org/10.1039/P29760001598
10. Phillips, L.; Wray, V. *J. Chem. Soc. B.* **1971**, 1618–1624. https://doi.org/10.1039/J29710001618
11. Michalik, M.; Hein, M.; Frank, M. *Carbohydr. Res.* **2000,** *327*, 185–218. https://doi.org/10.1016/S0008-6215(99)00323-7
