# Supplementary material for: Synthesis of multiply fluorinated N-acetyl-D-glucosamine and D-galactosamine analogs via the corresponding deoxyfluorinated glucosazide and galactosazide phenyl thioglycosides
Source: Beilstein J Org Chem. 2021 May 11;17:1086–95. doi: 10.3762/bjoc.17.85 (PMC8144920; doi:10.3762/bjoc.17.85)

BEILSTEIN JOURNAL OF ORGANIC CHEMISTRY

Supporting Information

for

Synthesis of multiply fluorinated *N*-acetyl-D-glucosamine and D-galactosamine analogs via the corresponding deoxyfluorinated glucosazide and galactosazide phenyl thioglycosides

Vojtěch Hamala, Lucie Červenková Šťastná, Martin Kurfiřt, Petra Cuřínová, Martin Dračínský and Jindřich Karban

Beilstein J. Org. Chem. **2021,** *17,* 1086–1095. doi:10.3762/bjoc.17.85

Copies of ^{1}H, ^{13}C, ^{19}F, and 2D NMR spectra for new compounds

License and Terms: This is a supporting information file under the terms of the Creative Commons Attribution License (https://creativecommons.org/licenses/by/4.0). Please note that the reuse, redistribution and reproduction in particular requires that the author(s) and source are credited and that individual graphics may be subject to special legal provisions.
The license is subject to the *Beilstein Journal of Organic Chemistry* terms and conditions: (https://www.beilstein-journals.org/bjoc/terms)

Table of Contents

NMR COMPOUND **18** S4

NMR COMPOUND α-**19** S9

NMR COMPOUND β-**19** S14

NMR COMPOUND **20** S19

NMR COMPOUND α-**23** S26

NMR COMPOUND β-**23** S31

NMR COMPOUND α-**24** S37

NMR COMPOUND β-**24** S42

NMR COMPOUND **25** S47

NMR COMPOUND **26** S52

NMR COMPOUND **31** S57

NMR COMPOUND β-**25** S63

NMR COMPOUND **32** S68

NMR COMPOUNDS β-**22** and **33** S73

NMR COMPOUND α-**34** S79

NMR COMPOUND β-**34** S84

NMR COMPOUND α-**35** S90

NMR COMPOUND **35** (α/β ca. 1/5) S95

NMR COMPOUND α-**36** S100

NMR COMPOUND β-**36** S105

NMR COMPOUND **37** S110

NMR COMPOUND **38** S116

NMR COMPOUND **39** S121

NMR COMPOUND **40** ... S127

NMR COMPOUND **41** ... S132

NMR COMPOUND **42** ... S138

NMR COMPOUND **43** ... S144

NMR COMPOUND **44** ... S149

NMR COMPOUND **45** ... S154

NMR COMPOUND **46** ... S160

NMR COMPOUND **47** ... S165

NMR COMPOUND **48** ... S170

NMR COMPOUND **49** ... S175

NMR COMPOUND **50** ... S181

NMR COMPOUND **51** ... S187

NMR COMPOUND **52** ... S193

NMR COMPOUND **53** ... S198

NMR COMPOUND **54** ... S204

NMR COMPOUND **55** ... S209

NMR COMPOUND **56** ... S214

NMR COMPOUND **57** ... S220

NMR COMPOUND **58** ... S225

NMR COMPOUND **59** ... S231

NMR COMPOUND **60** ... S237

NMR COMPOUND **61** ... S243

NMR COMPOUND **62** ... S248

NMR COMPOUND **63** ... S253

NMR COMPOUND **64** S258

NMR COMPOUND **65** S263

NMR COMPOUND **66** S268

NMR COMPOUND **67** S273

NMR COMPOUND **68** S278

NMR COMPOUND **69** S283

NMR COMPOUND **70** S288

NMR COMPOUND **71** S294

NMR COMPOUND **72** S297

NMR COMPOUND 18

^{1}H NMR (400 MHz, $CDCl_3$) 18 (α/β ca. 10/3)

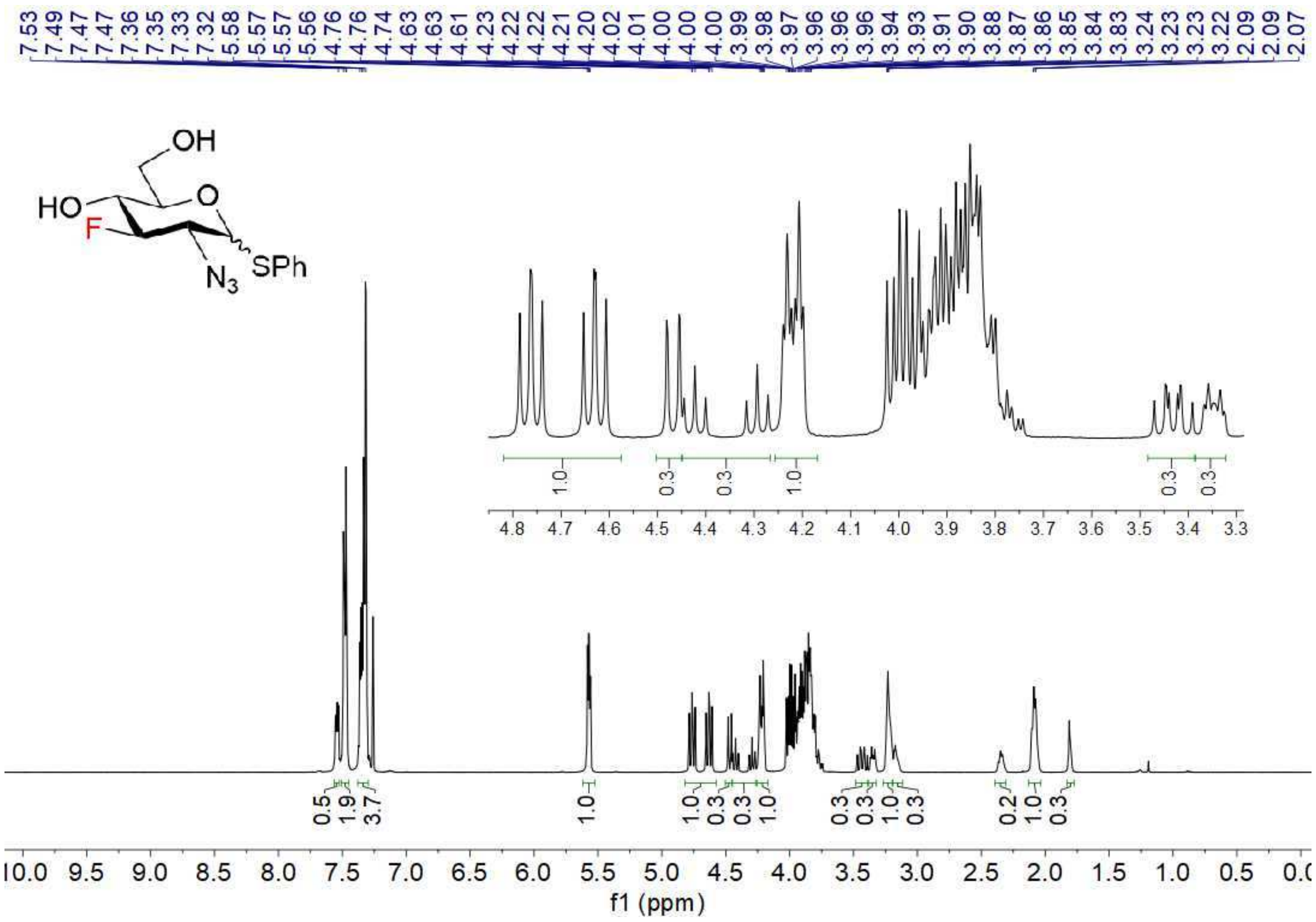

^{13}C NMR (100 MHz, $CDCl_3$) 18

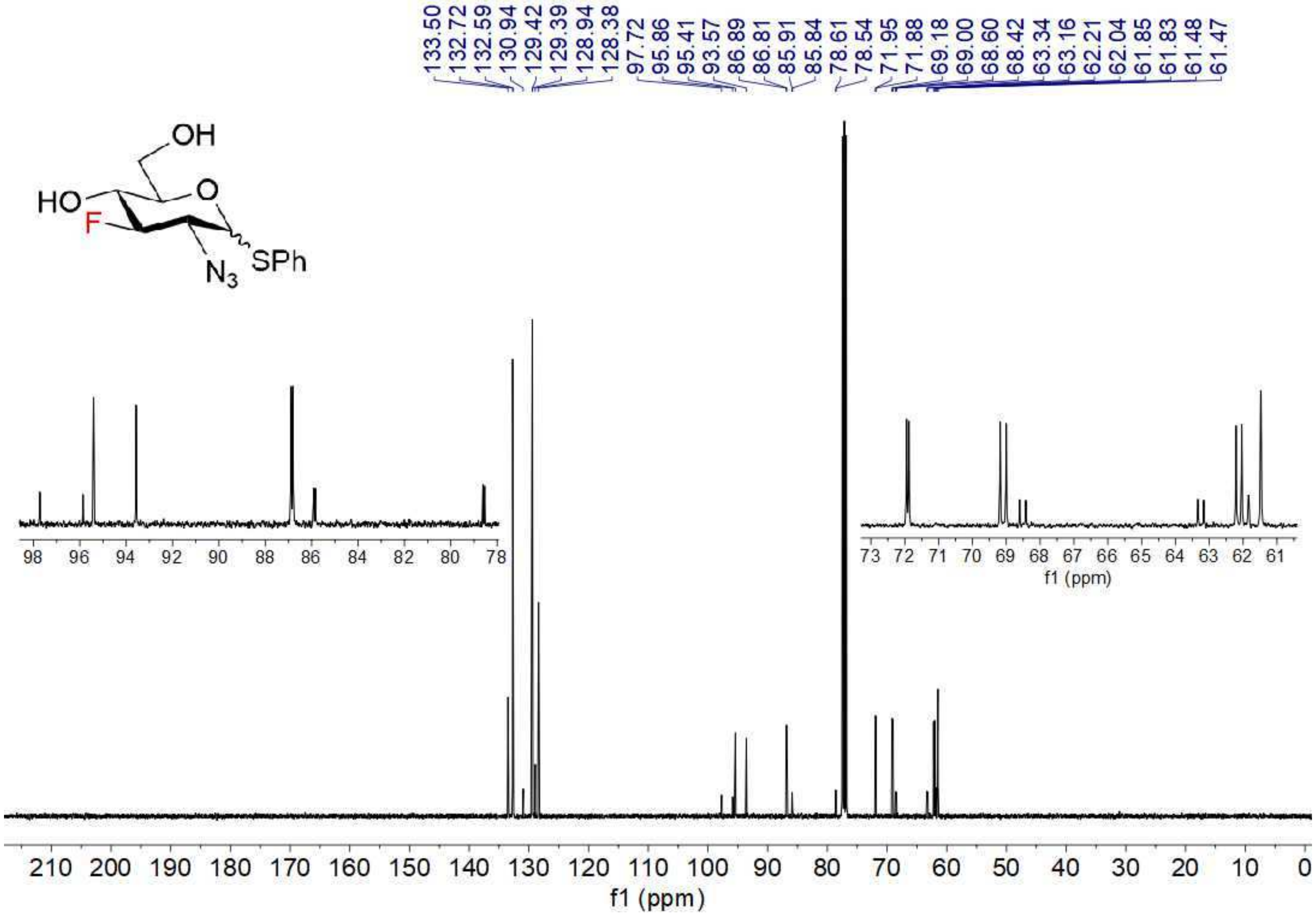

^{19}F NMR (376 MHz, CDCl$_3$) 18 (α/β ca. 10/3)

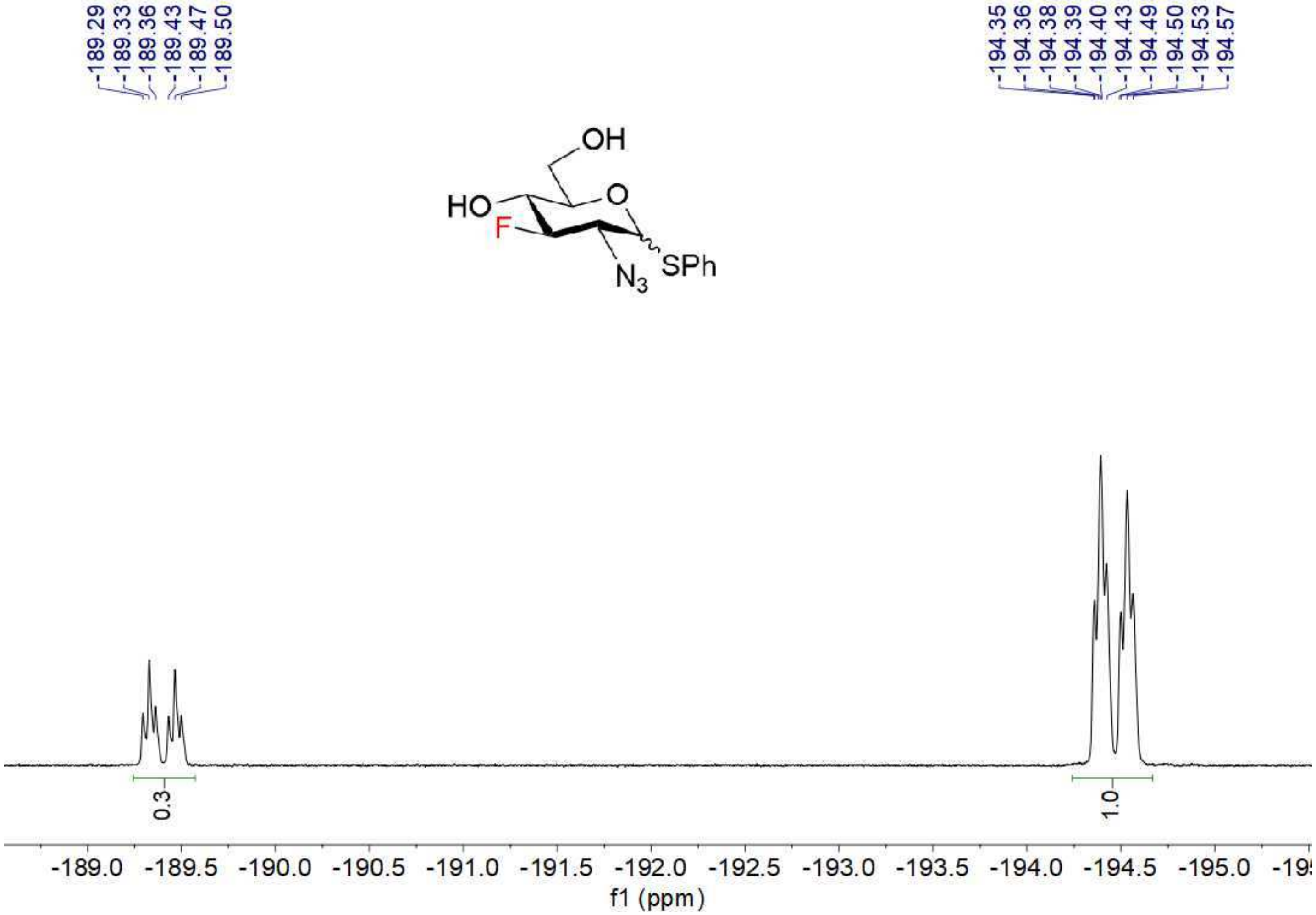

^{1}H-^{1}H COSY 18

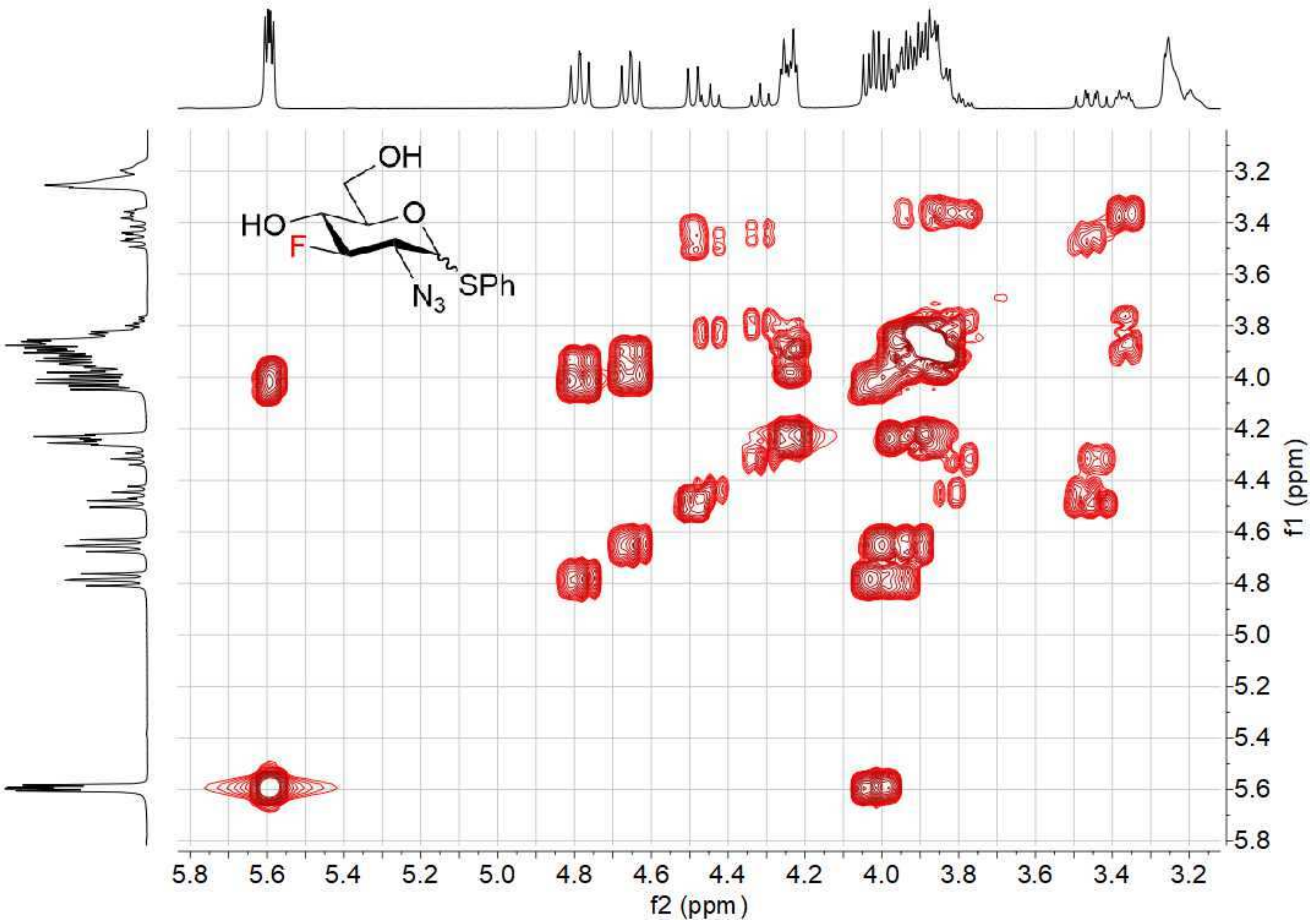

^{1}H-^{13}C HSQC 18

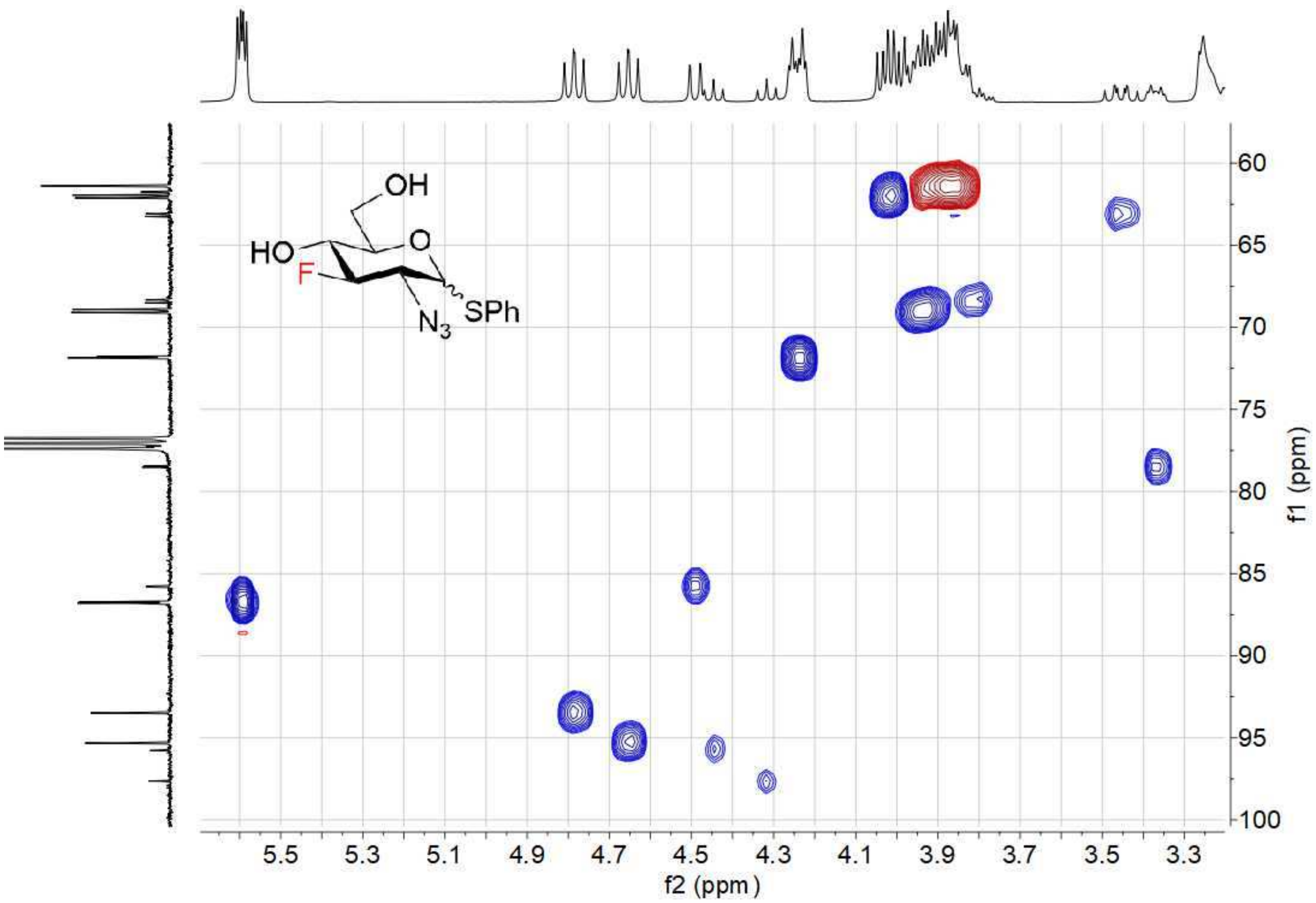

NMR COMPOUND α-19

^{1}H NMR (400 MHz, $CDCl_3$) α-19

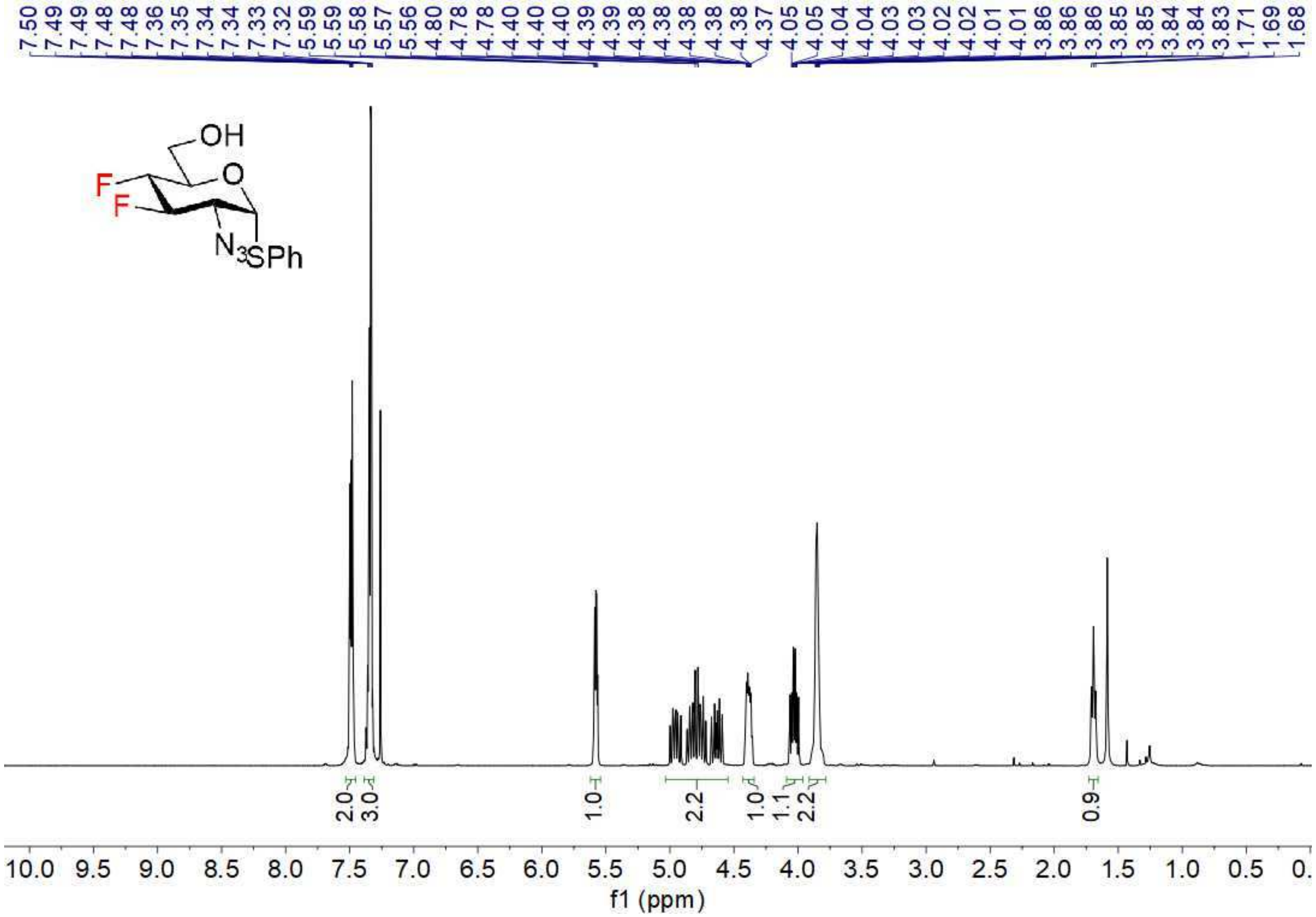

^{13}C NMR (100 MHz, CDCl$_3$) α-19

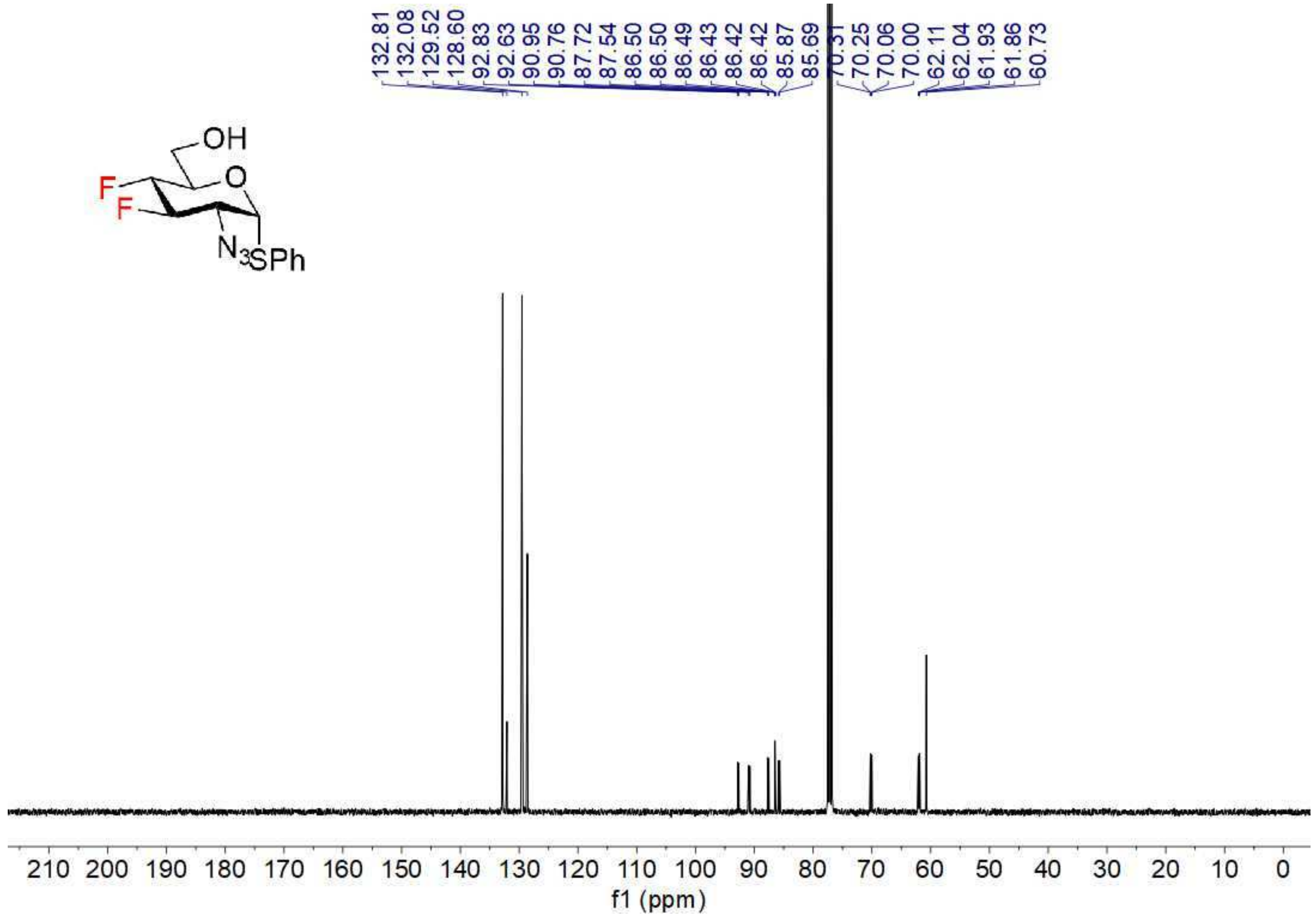

^{19}F NMR (376 MHz, $CDCl_3$) α-19

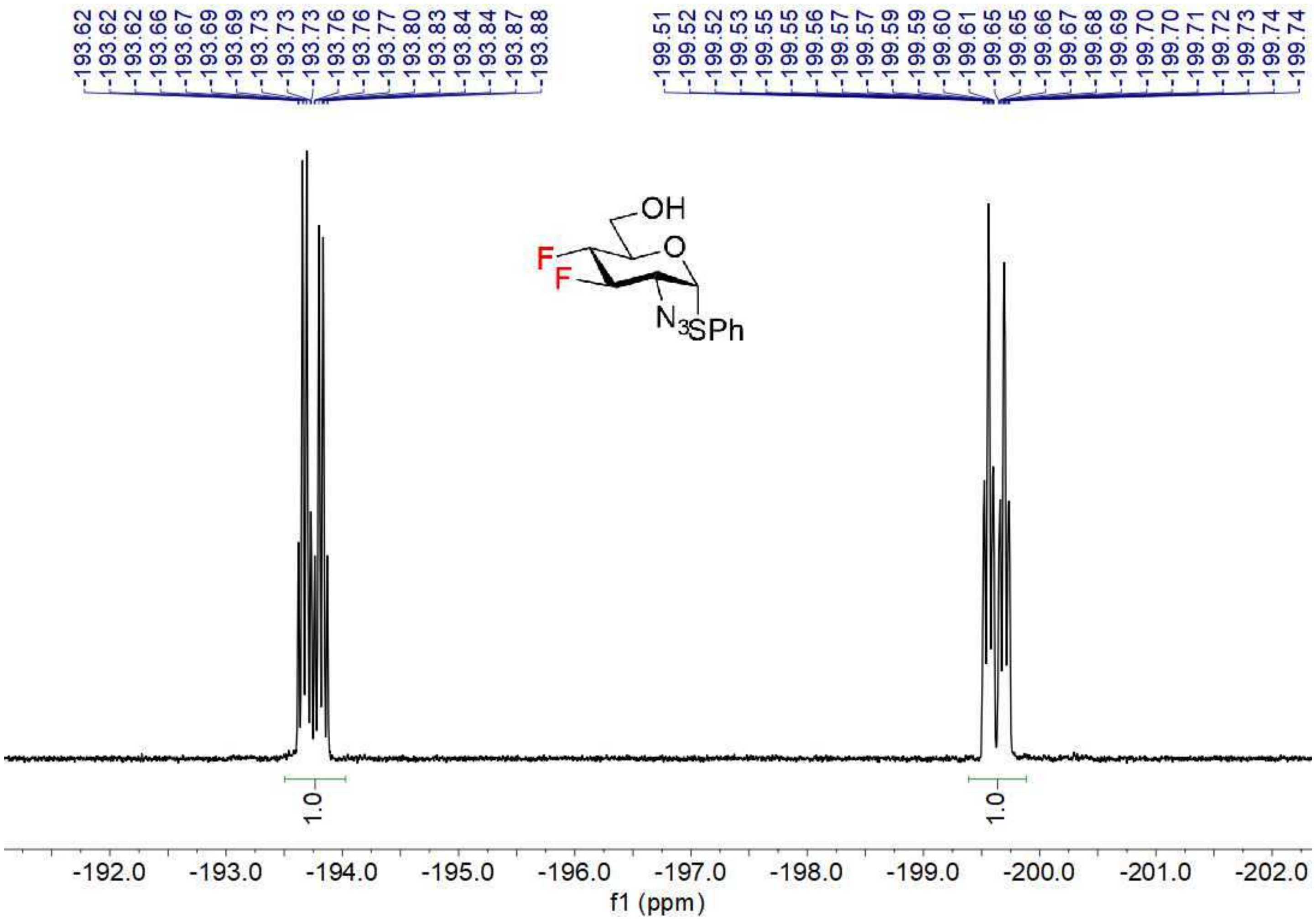

^{1}H-^{1}H COSY α-19

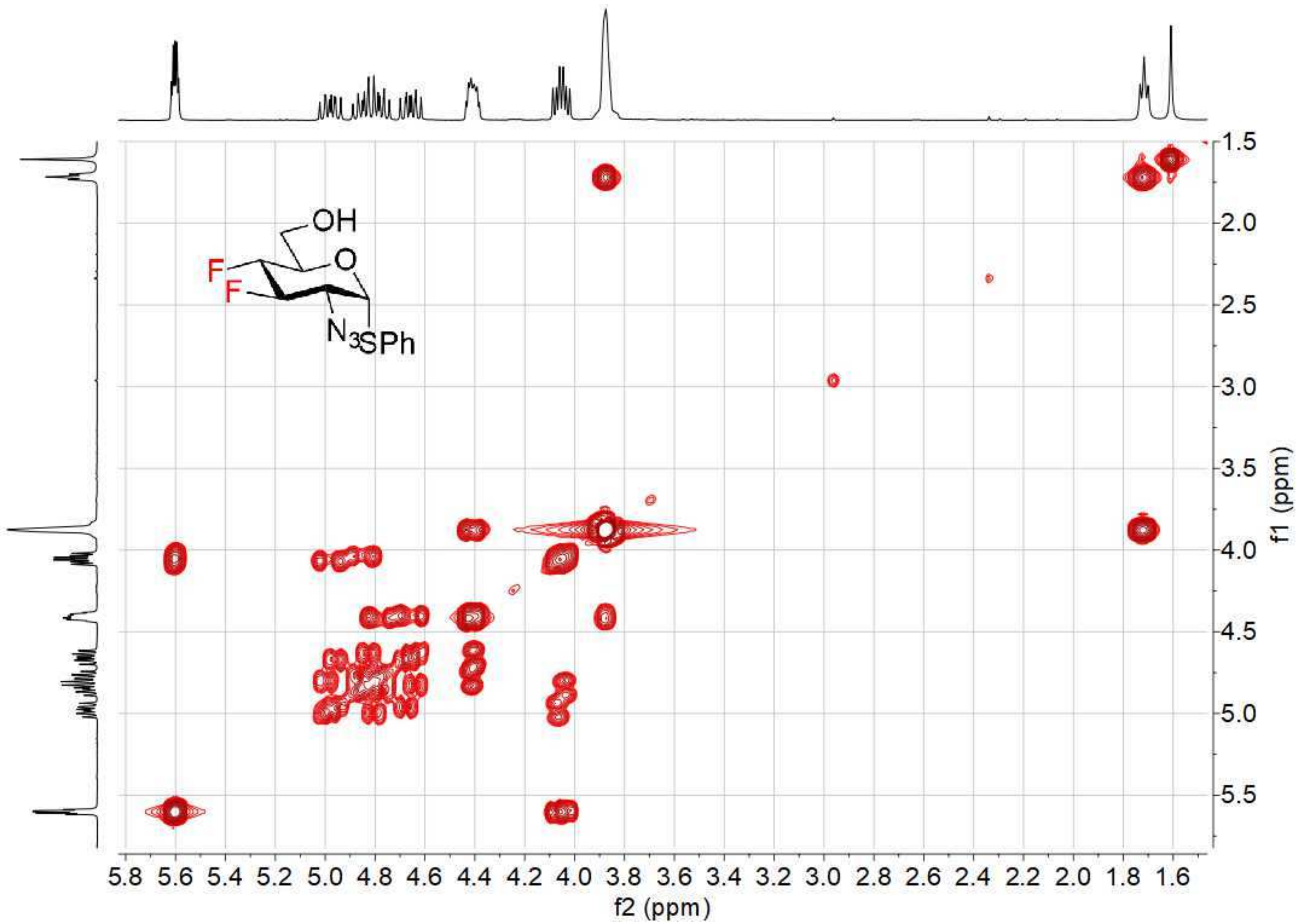

^{1}H-^{13}C HSQC α-19

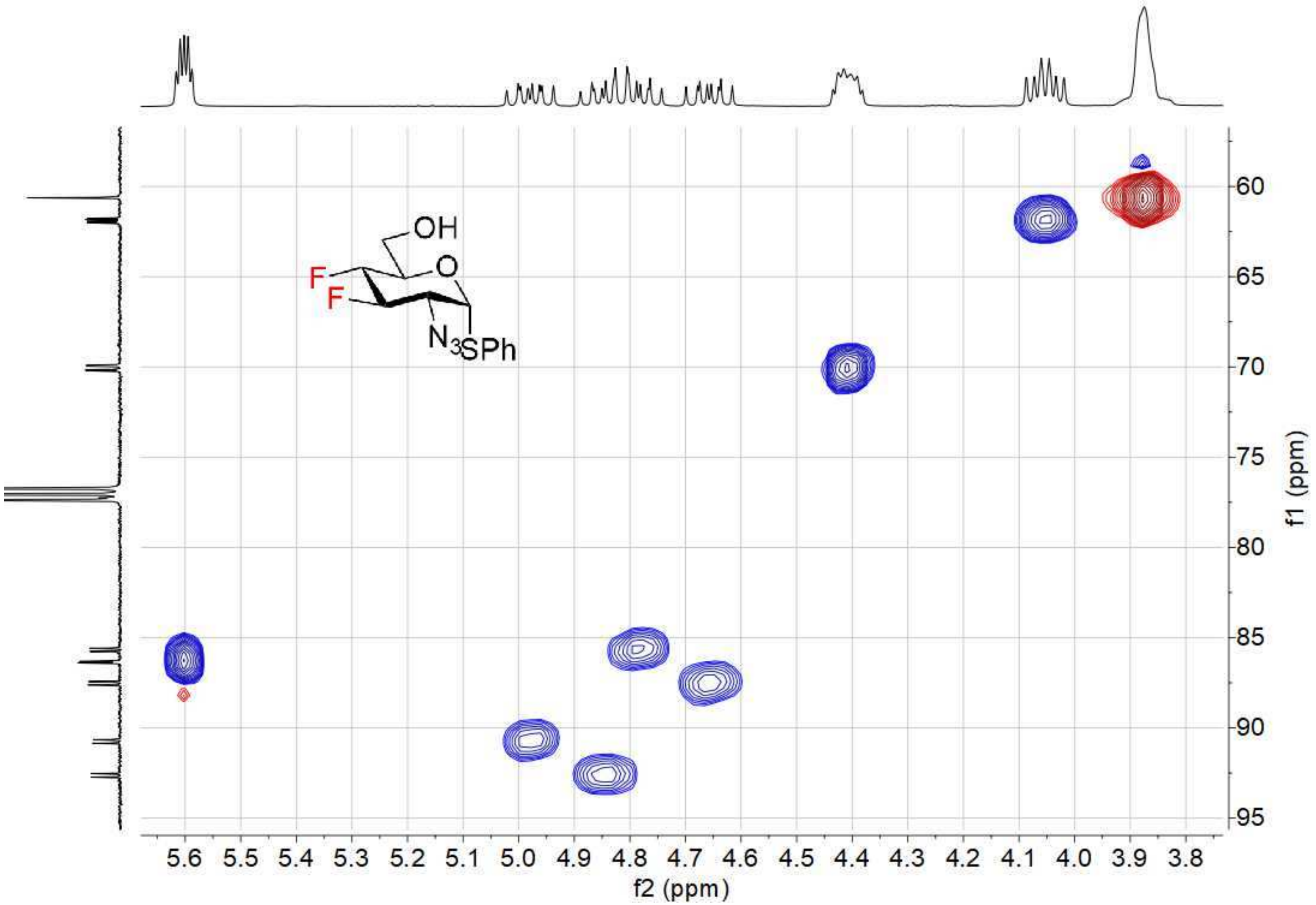

NMR COMPOUND β-19

(ca 16% of 2,3-dideoxy-2,3-difluoro-*C*-α- or β-D-arabinofuranosyl-formaldehyde diphenyl dithioacetal S1)

^{1}H NMR (400 MHz, $CDCl_3$) β-19 (and ca 16% S1)

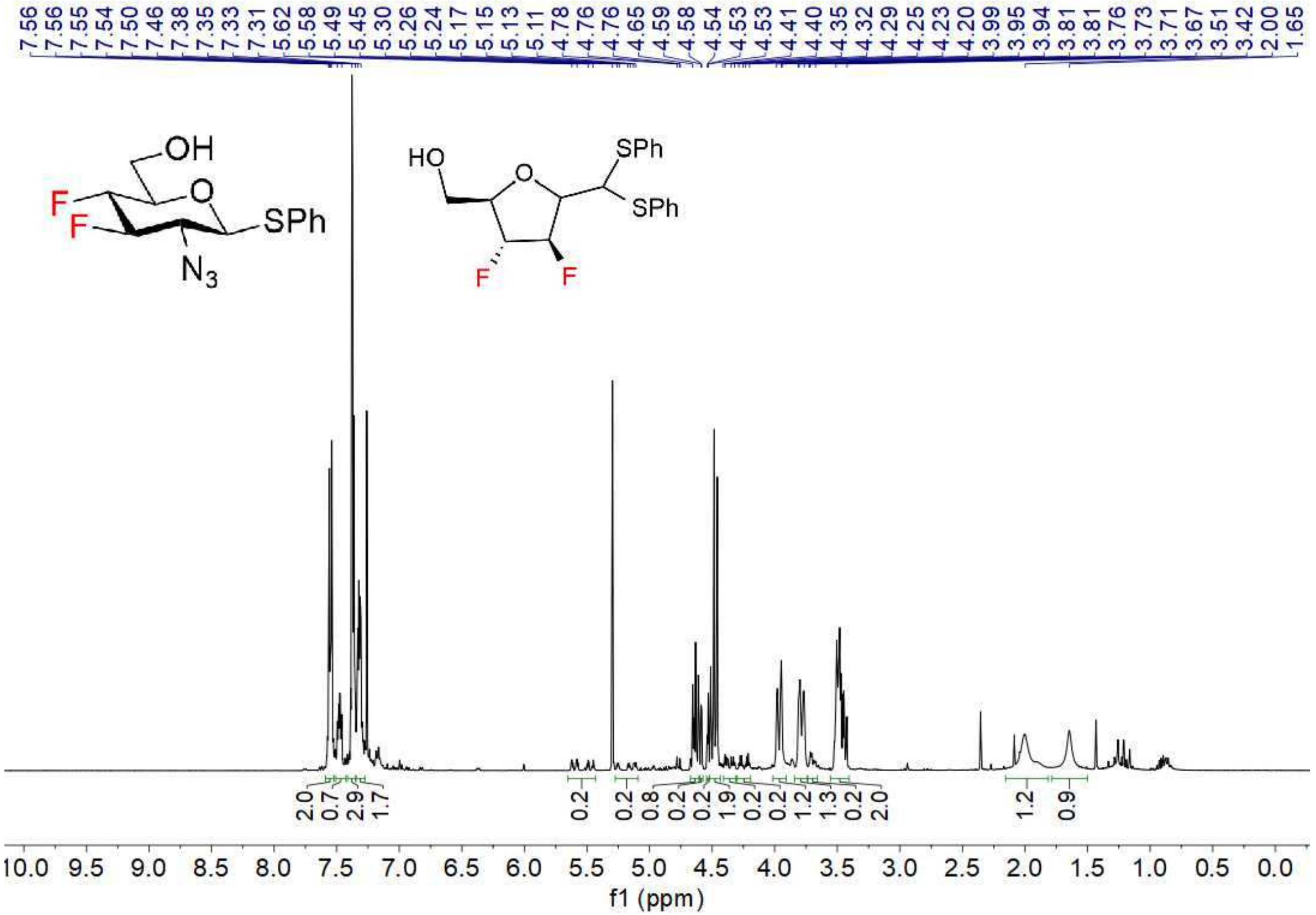

^{13}C NMR (100 MHz, $CDCl_3$) β-19 (and ca 16% S1)

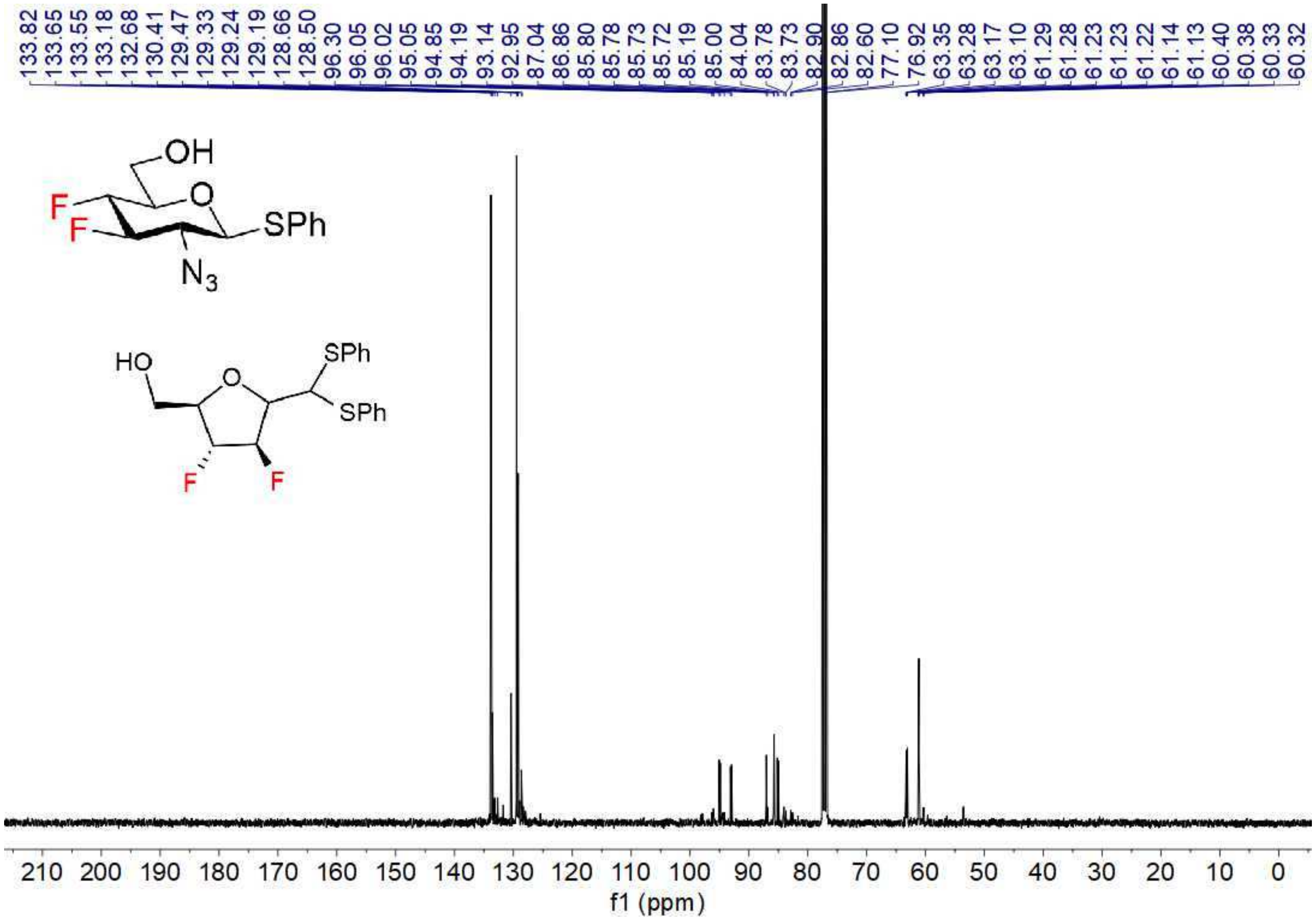

^{19}F NMR (376 MHz, $CDCl_3$) β-19 (and ca 16% S1)

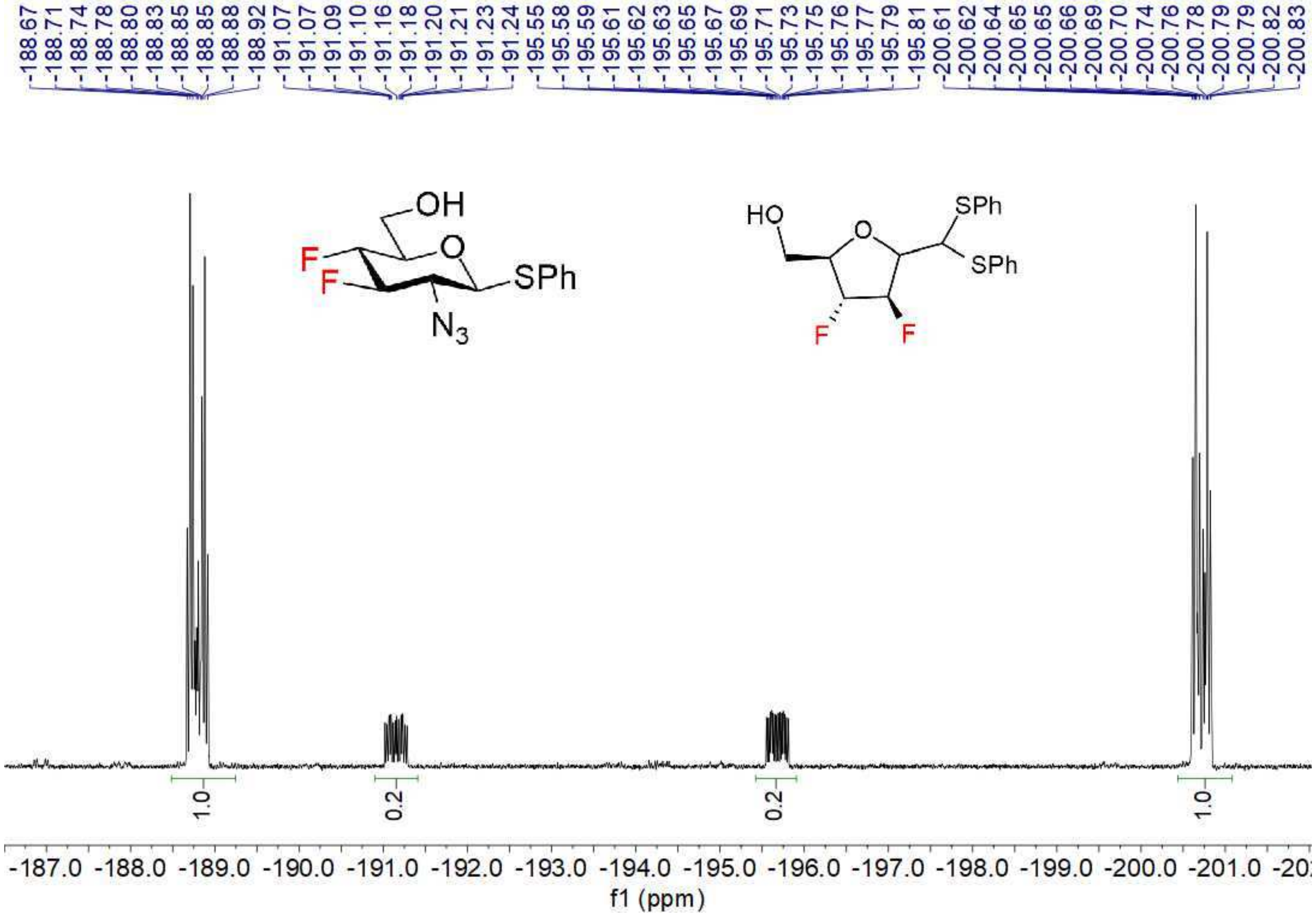

^{1}H-^{1}H COSY β-19

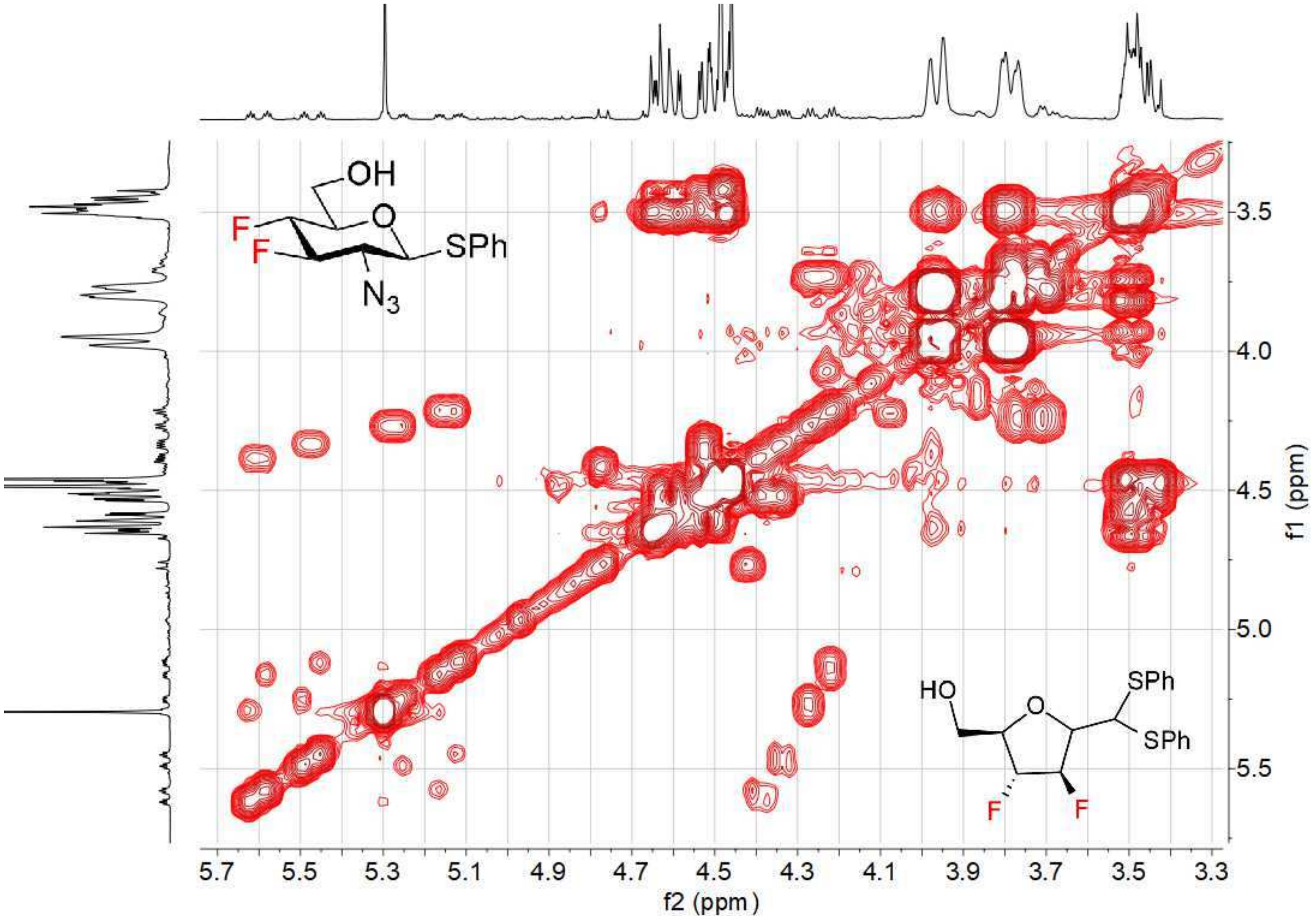

^{1}H-^{13}C HSQC β-19

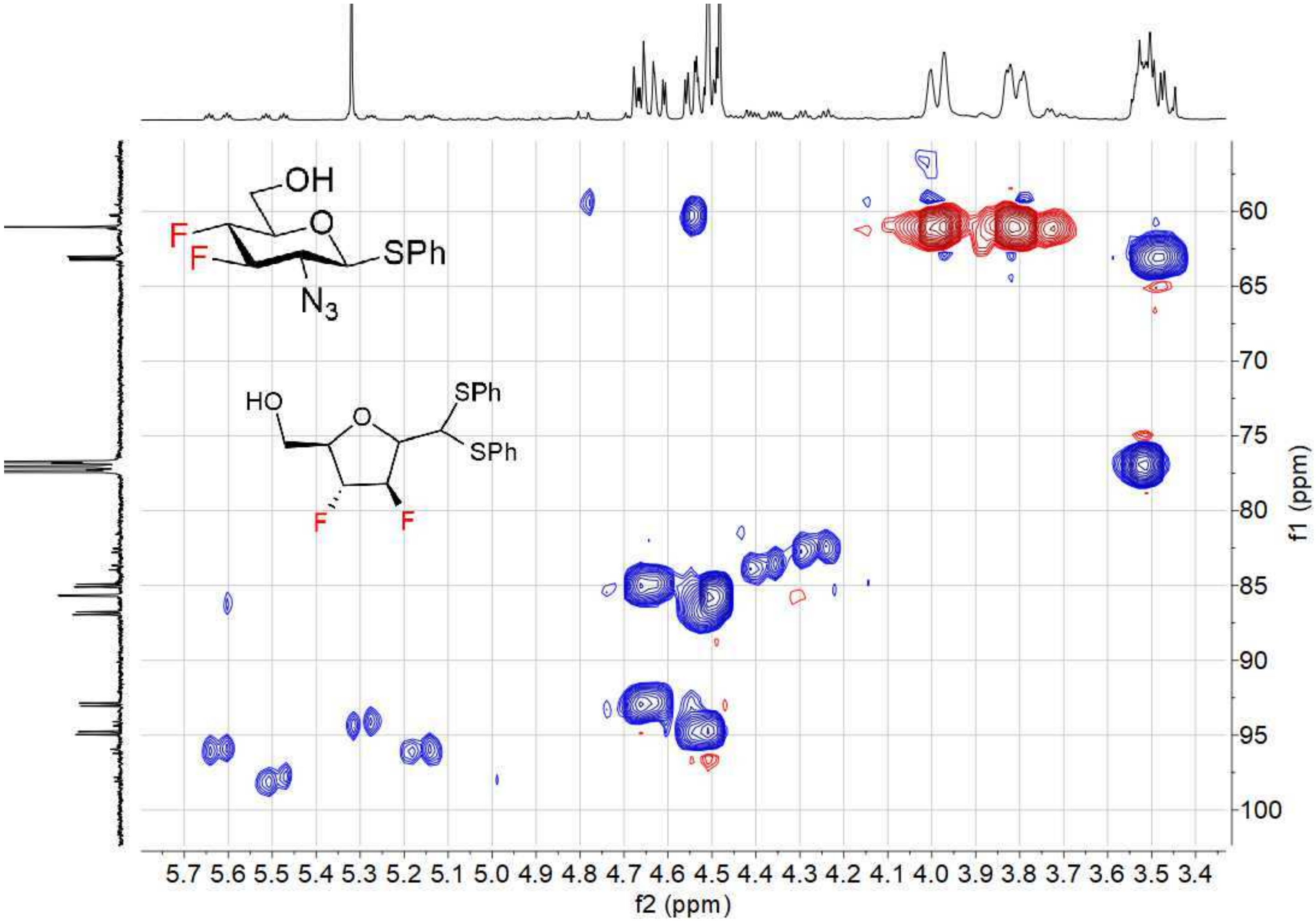

NMR COMPOUND 20

^{1}H NMR (400 MHz, $CDCl_3$) 20

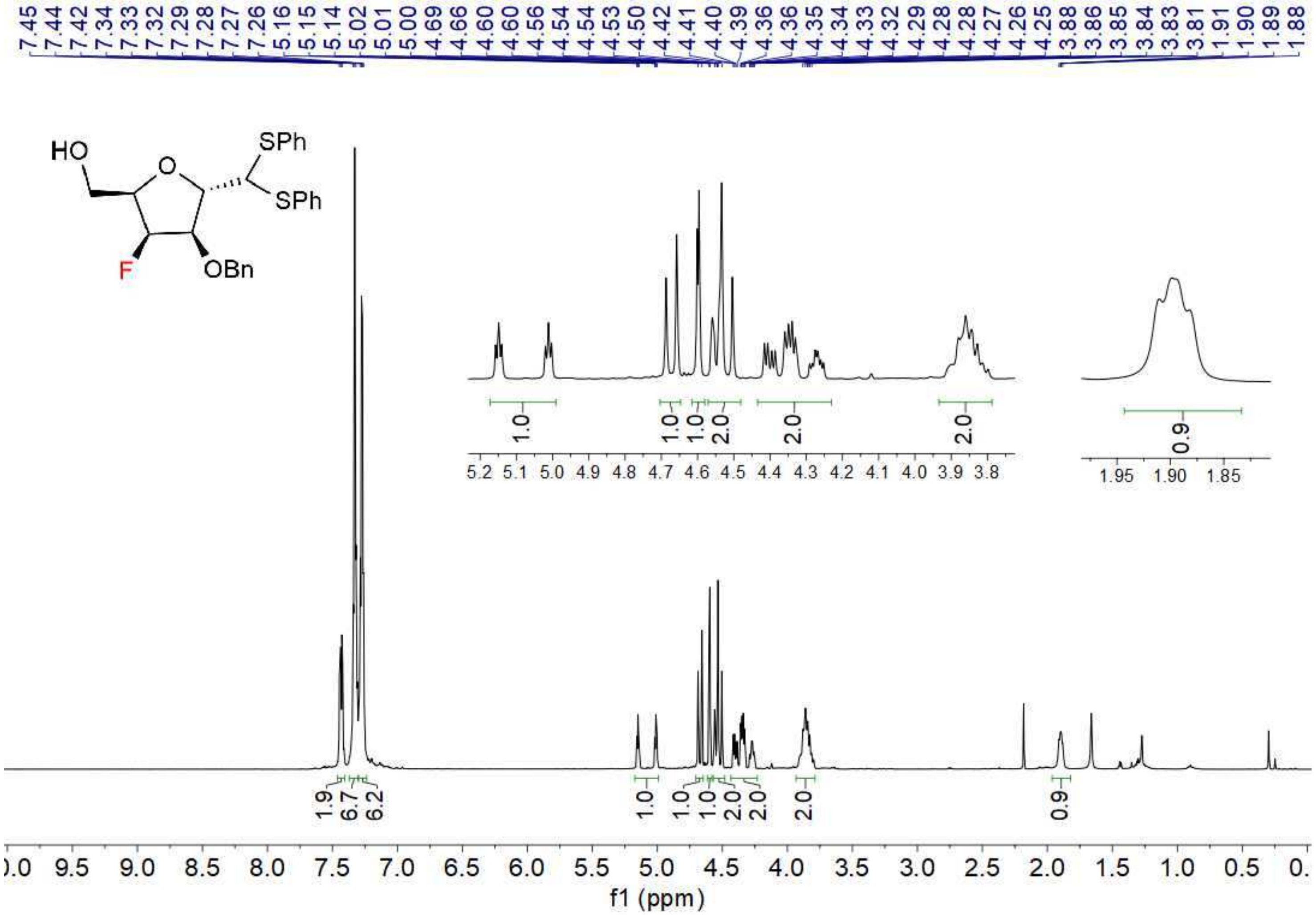

^{13}C NMR (100 MHz, CDCl$_3$) 20

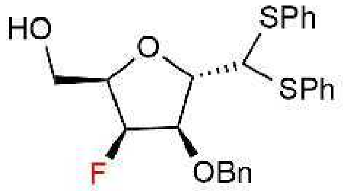

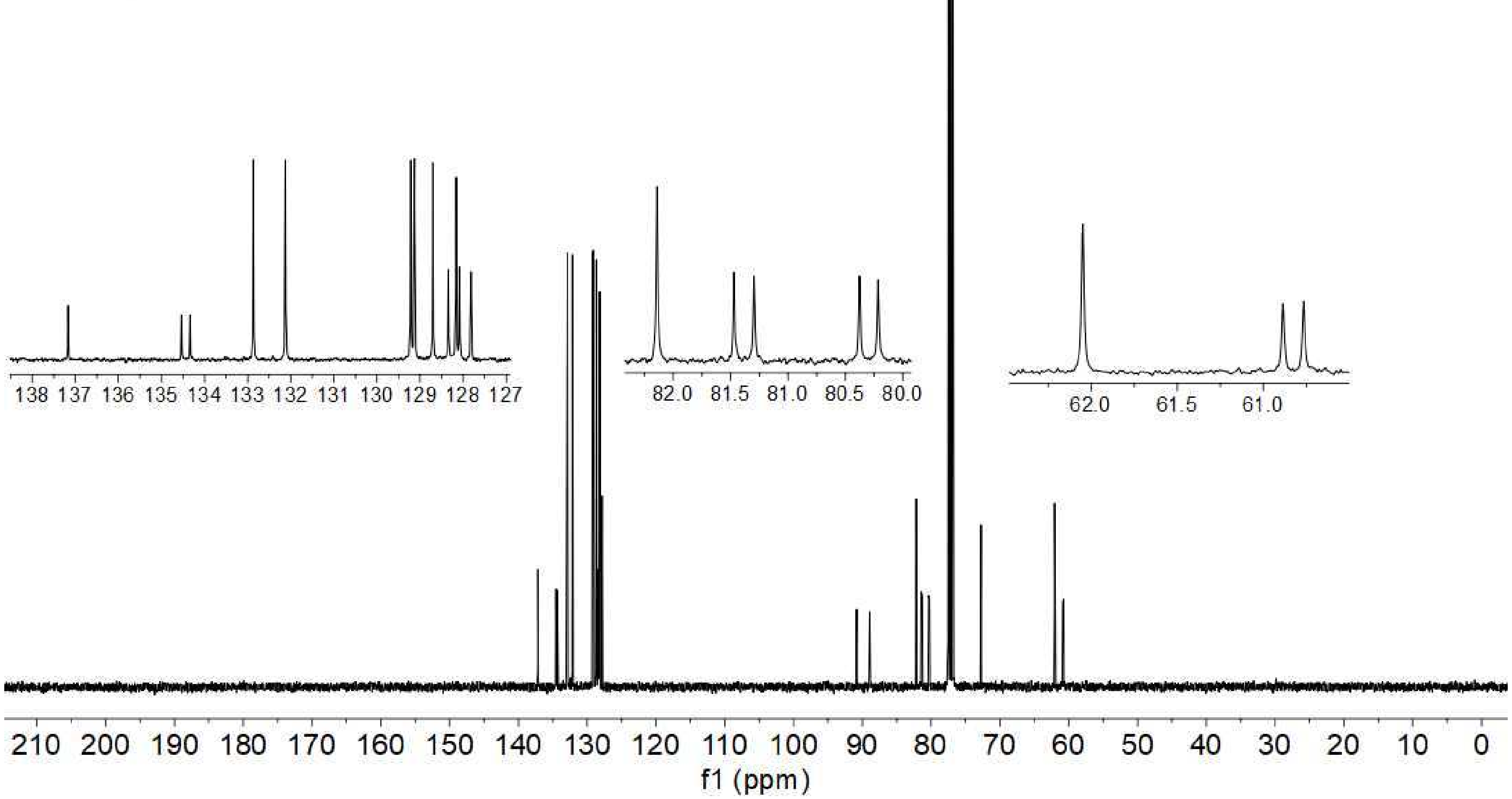

^{19}F NMR (376 MHz, $CDCl_3$) 20

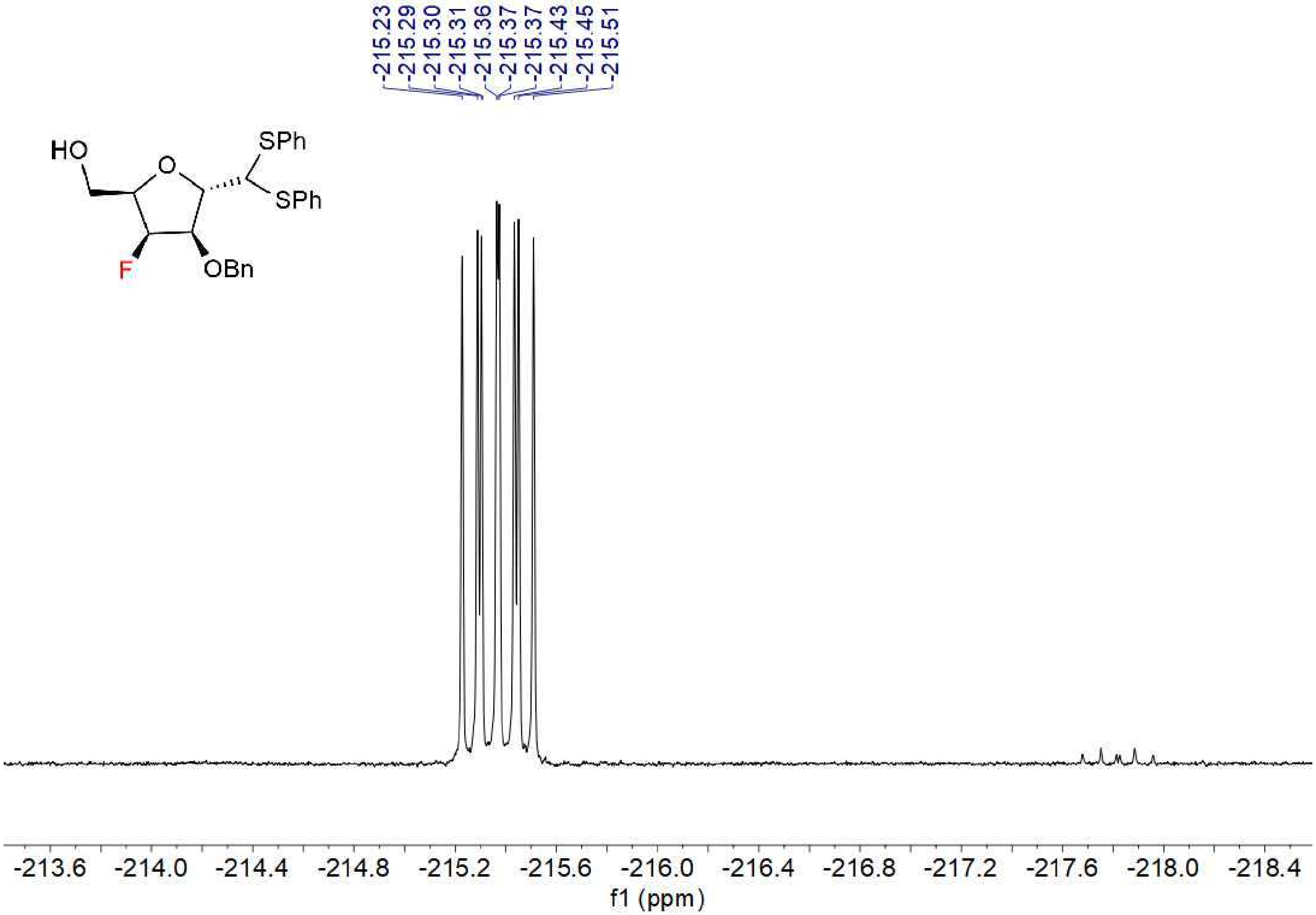

^{1}H-^{1}H COSY 20

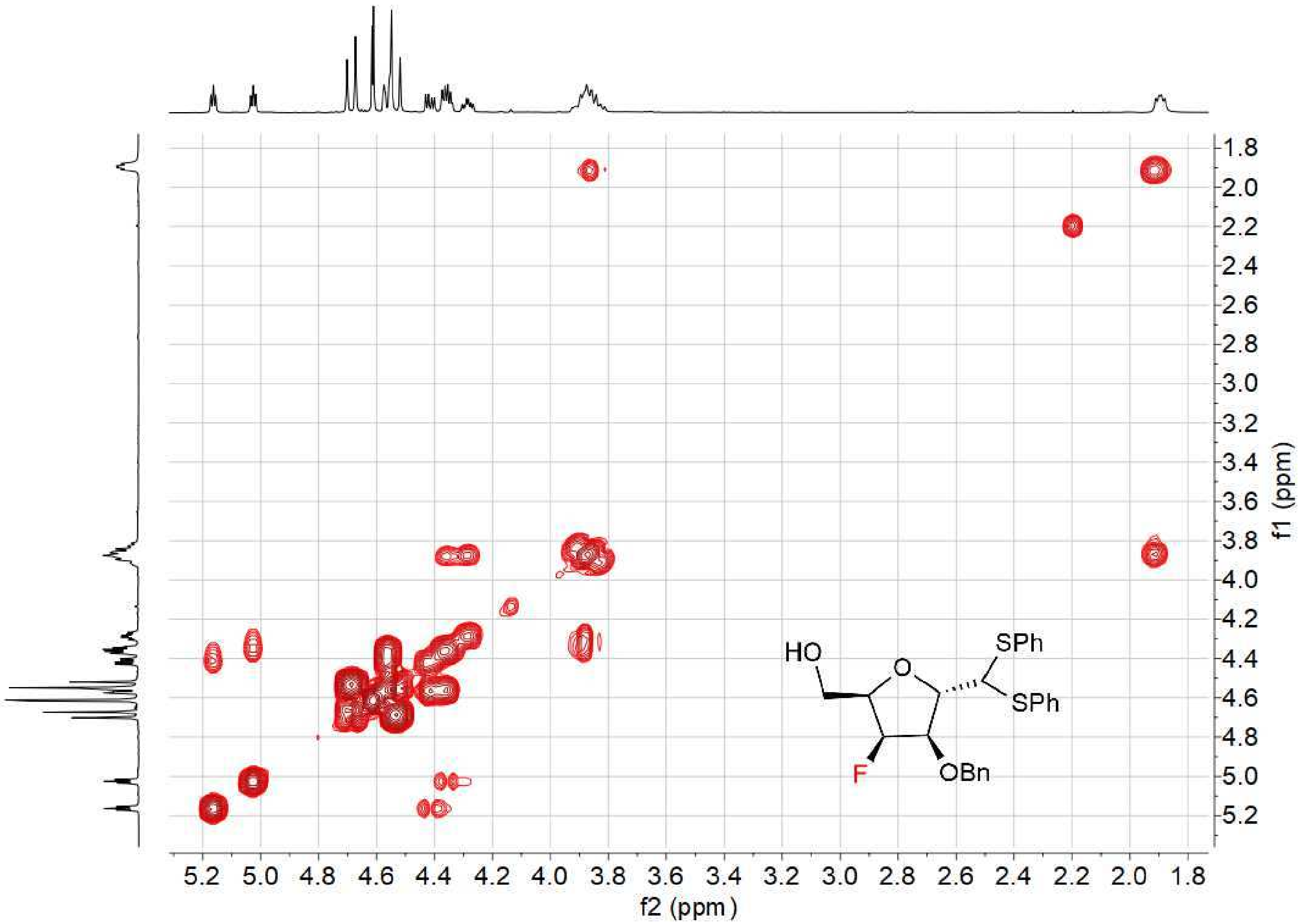

5.2
5.0
4.8
4.6
4.4
4.2
4.0
3.8
3.6
3.4
3.2
3.0
2.8
2.6
2.4
2.2
2.0
1.8
f2 (ppm)
f1 (ppm)
HO
O
SPh
SPh
F
OBn

^{1}H-^{13}C HMBC 20

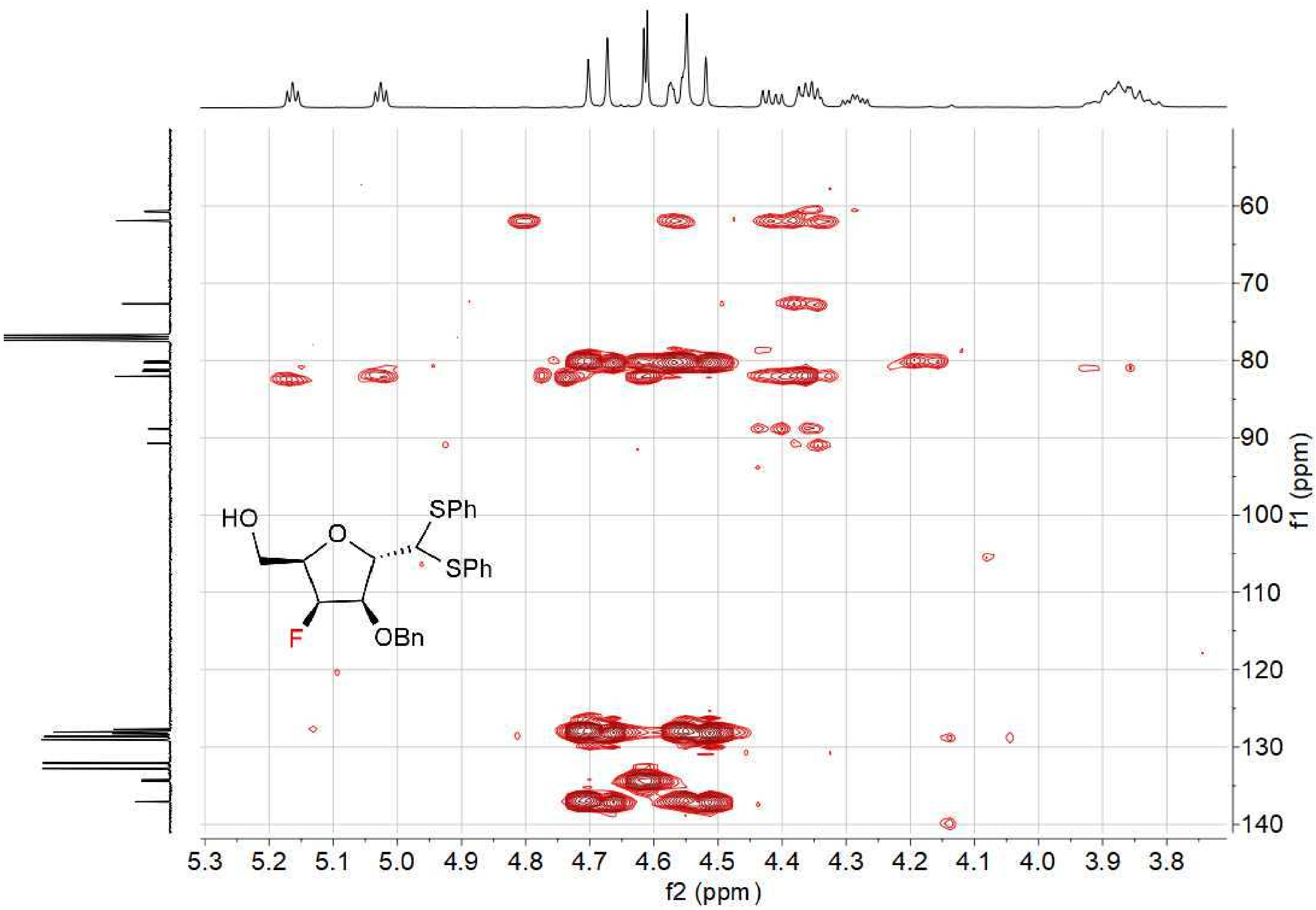

^{1}H-^{13}C HSQC 20

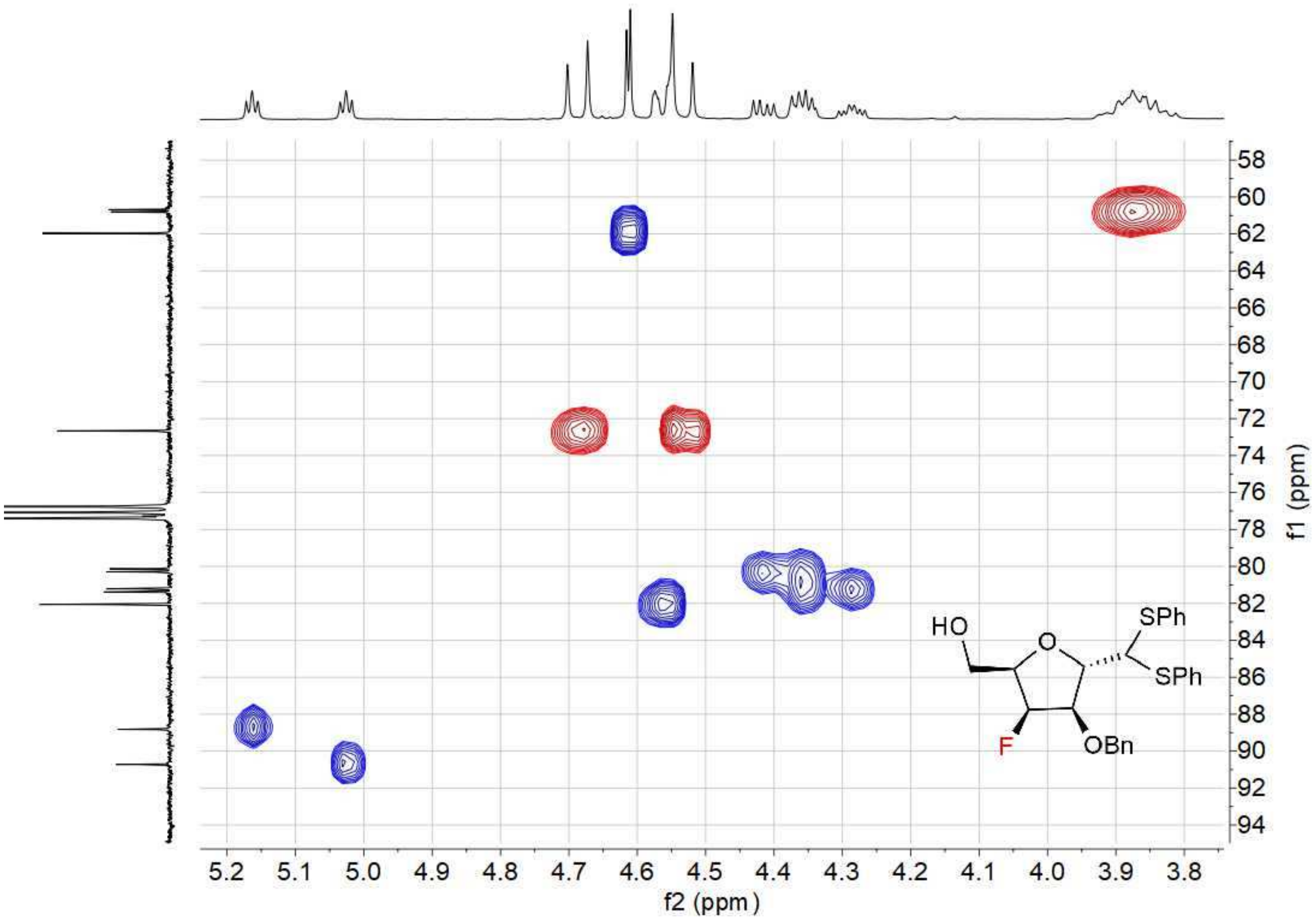

^{1}H-^{3}H ROESY 20

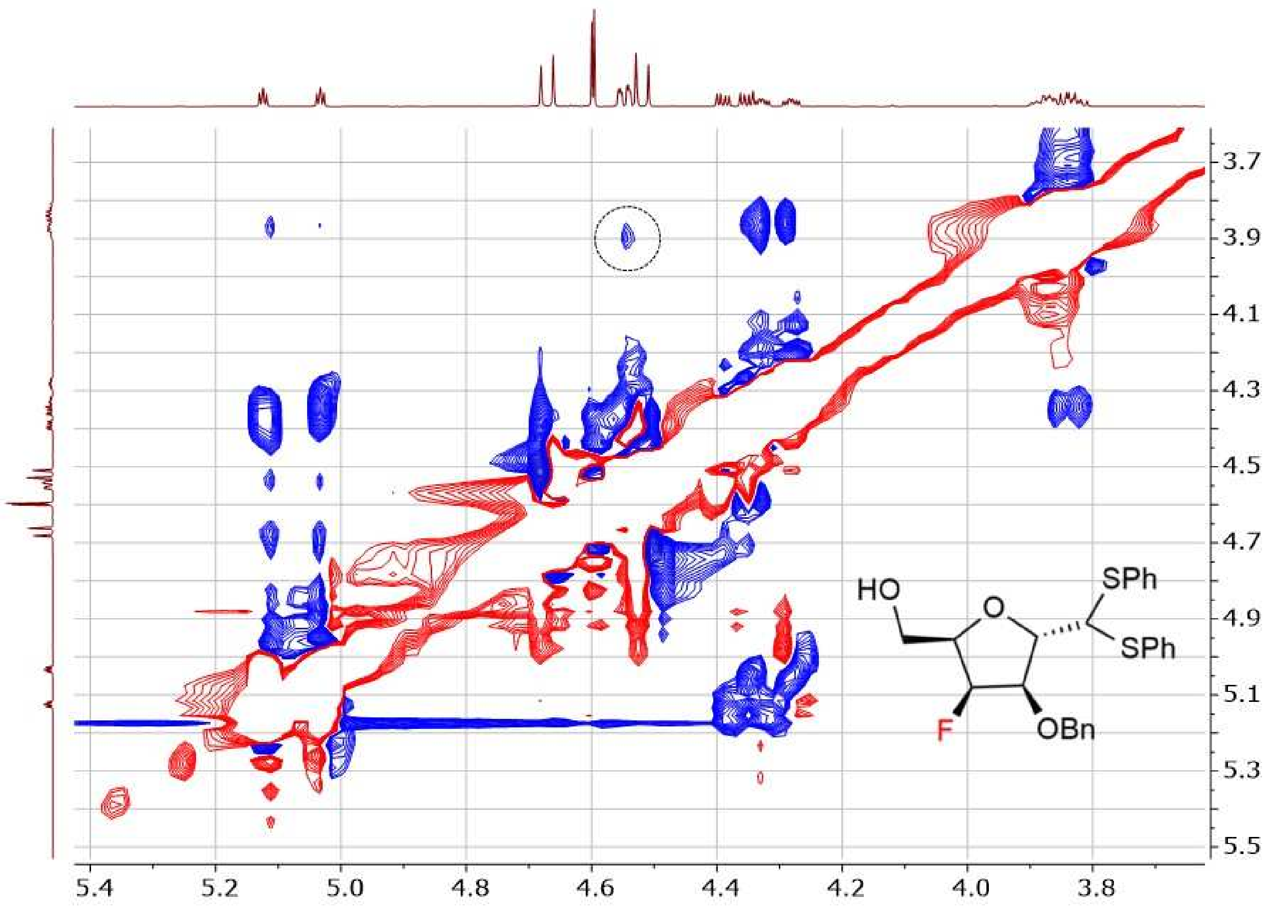

NMR COMPOUND α-23

^{1}H NMR (400 MHz, $CDCl_3$) α-23

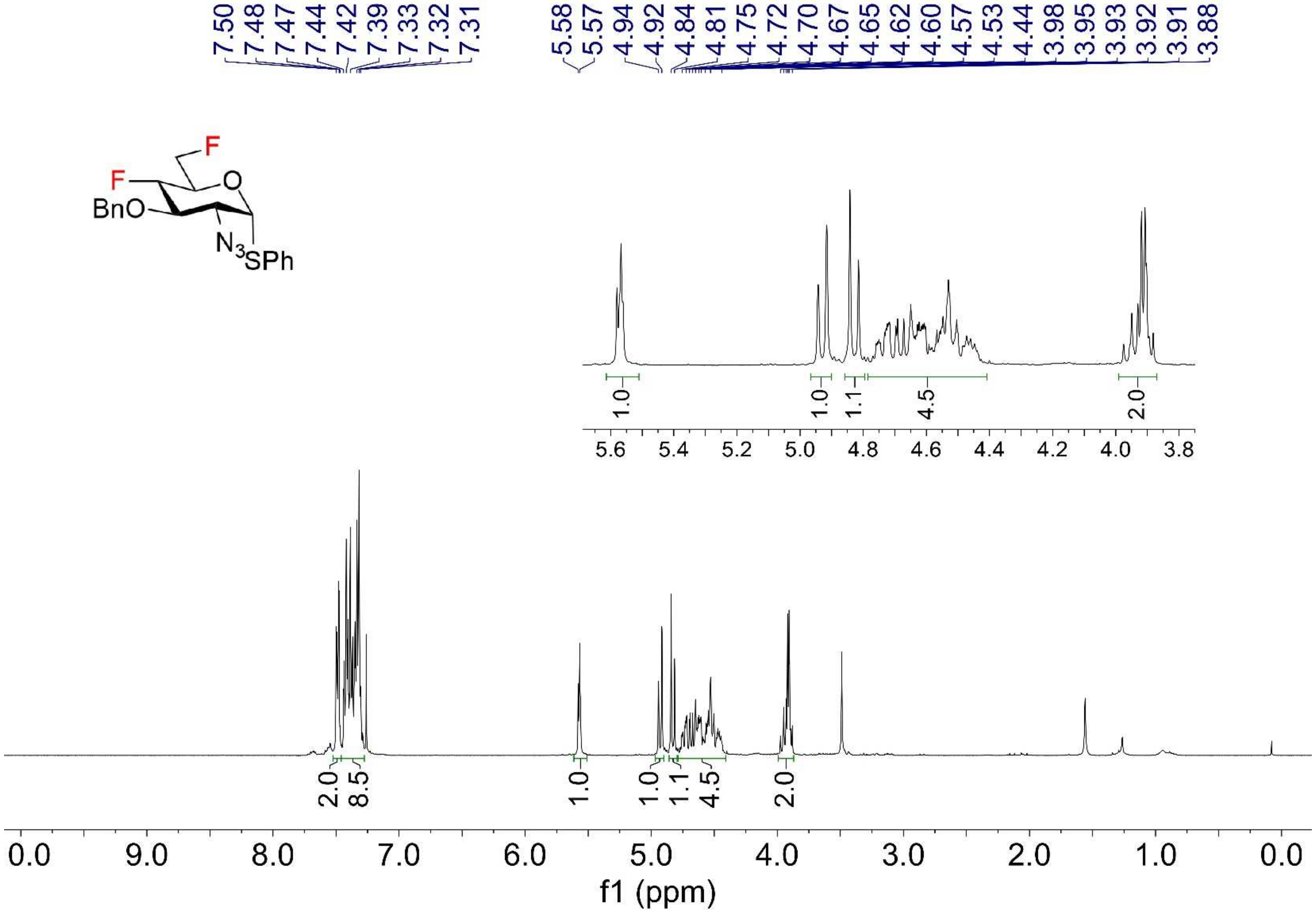

^{13}C NMR (100 MHz, CDCl$_3$) α-23

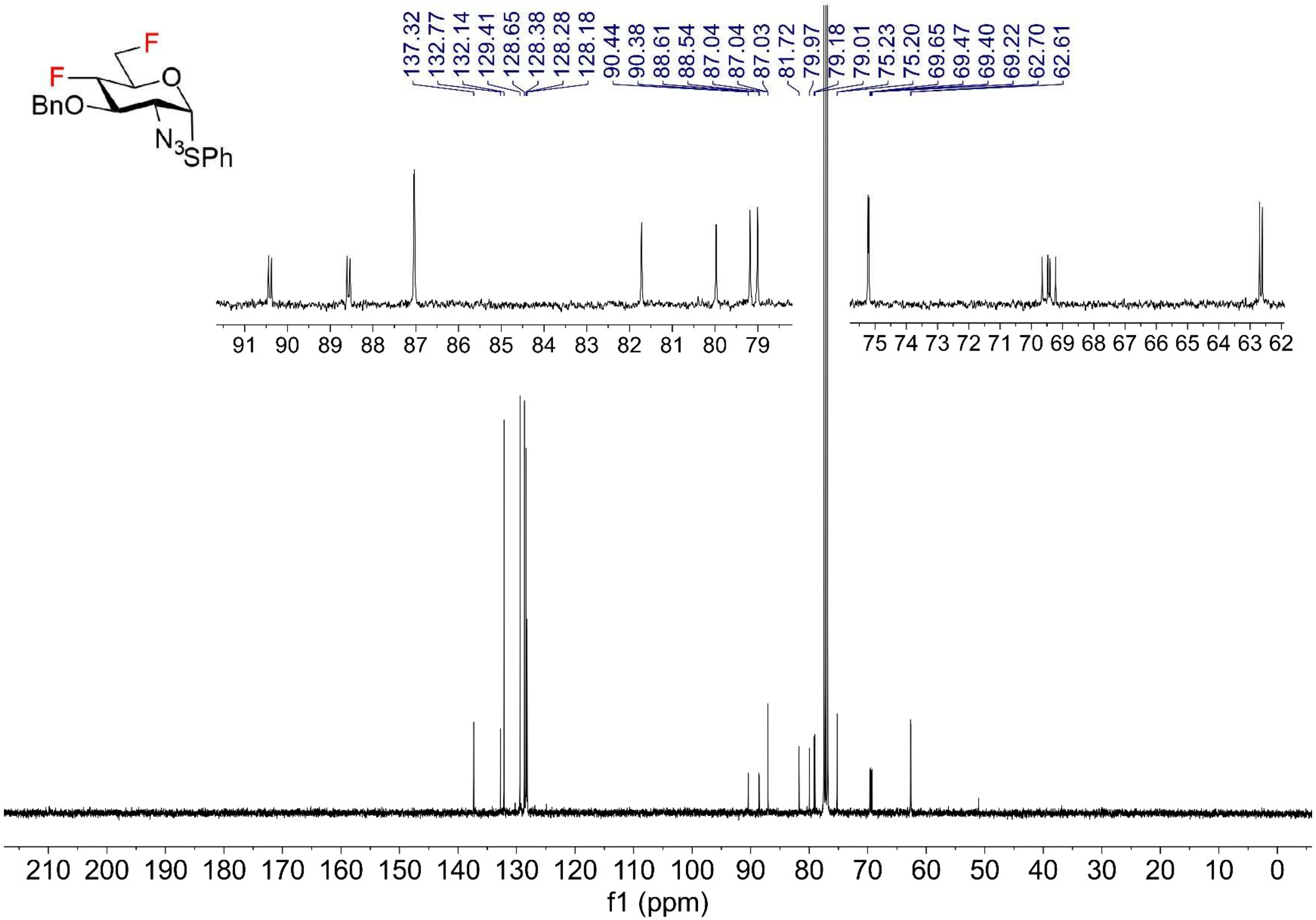

^{19}F NMR (376 MHz, CDCl₃) α-23

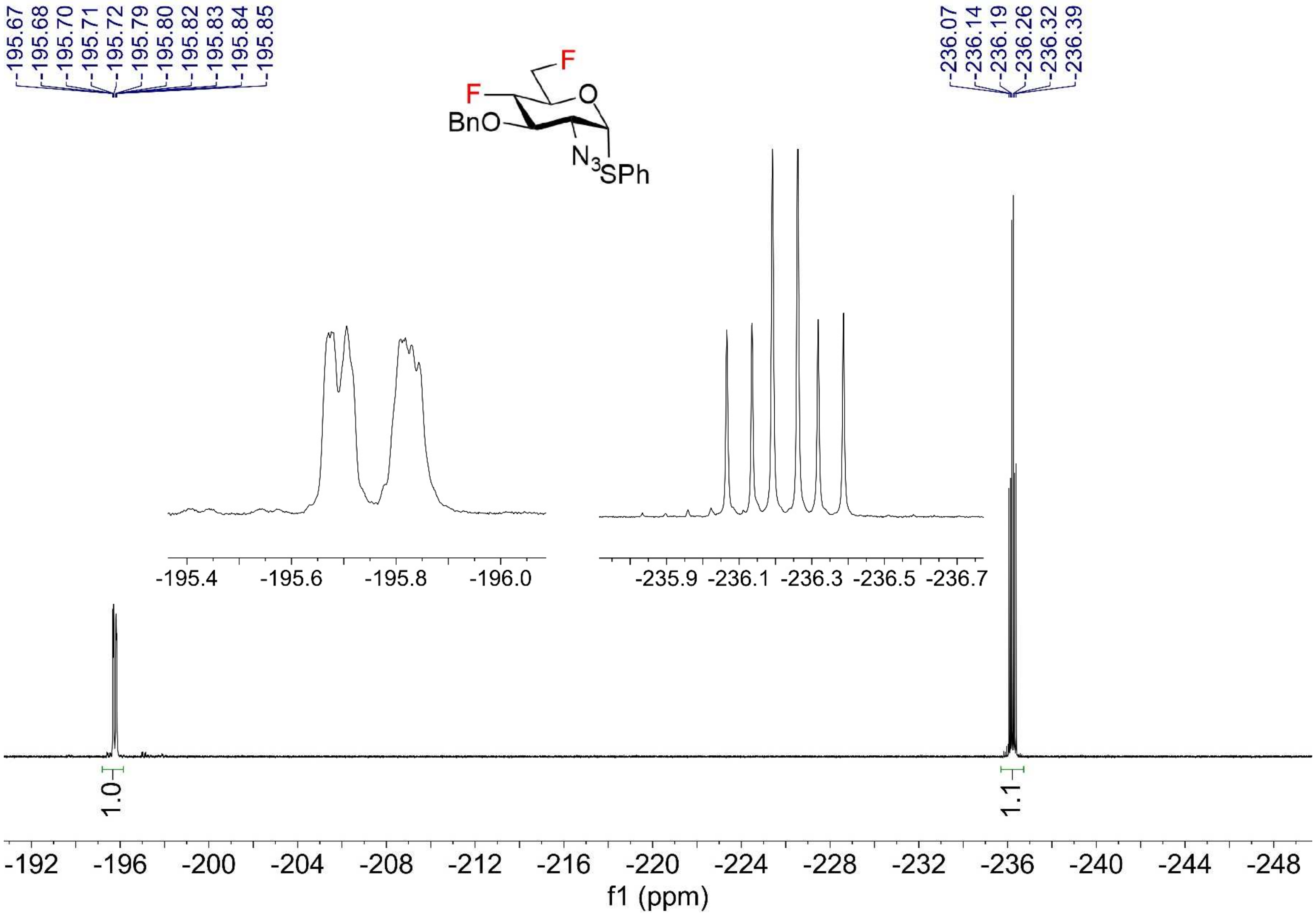

^{1}H-^{1}H COSY α-23

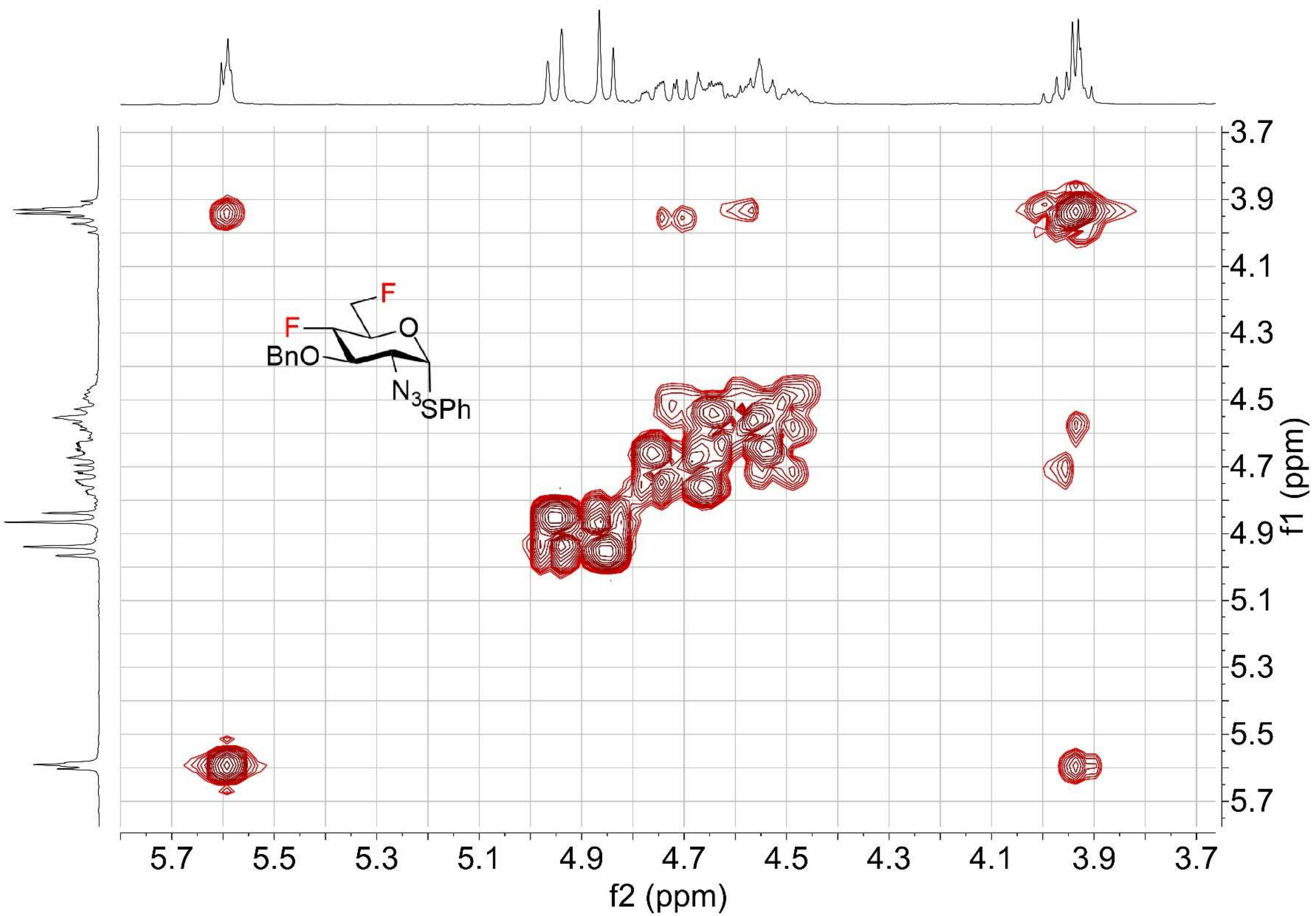

^{1}H-^{13}C HSQC α-23

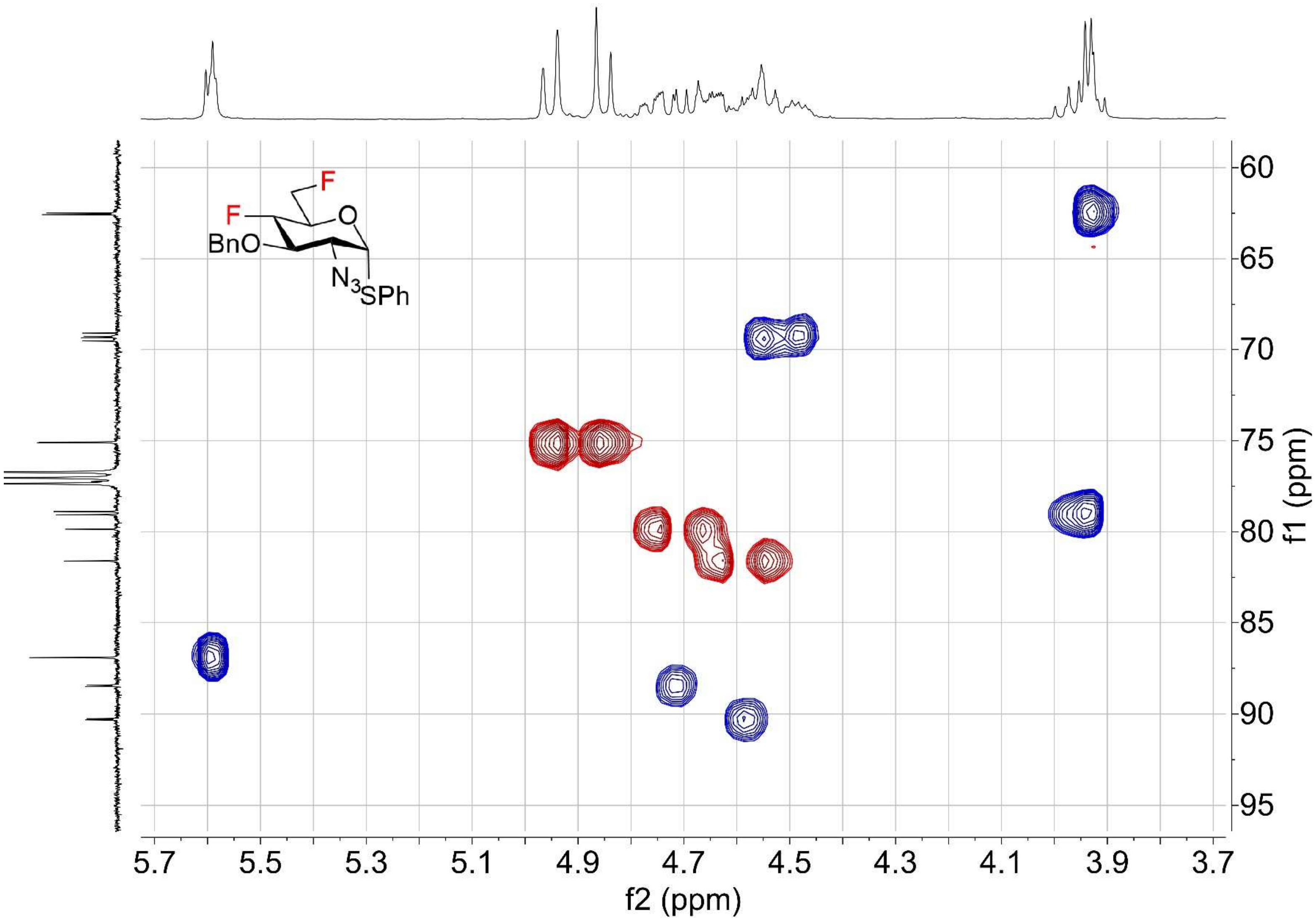

NMR COMPOUND β-23

^{1}H NMR (400 MHz, $CDCl_3$) β-23

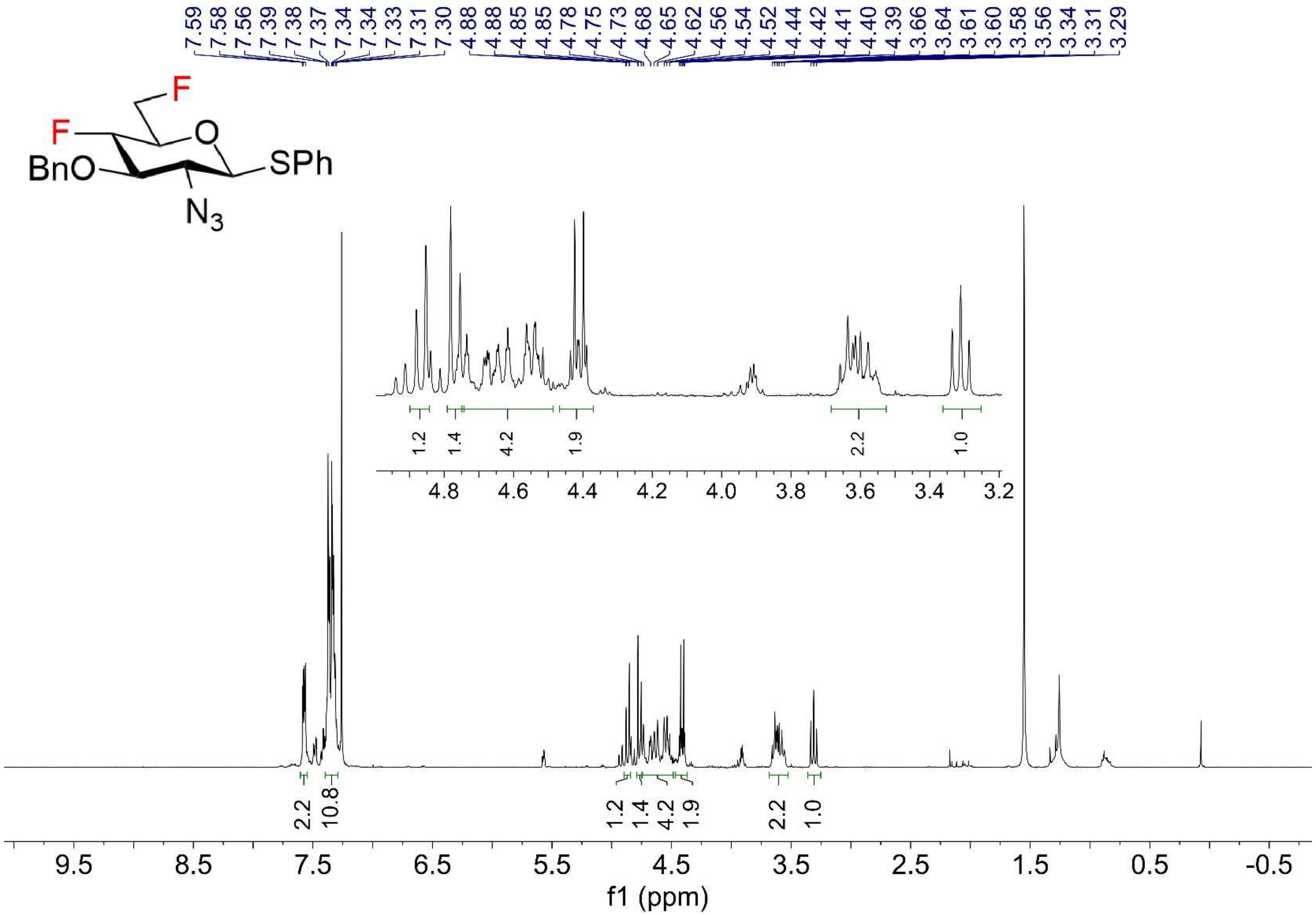

^{13}C NMR (100 MHz, CDCl$_3$) β-23

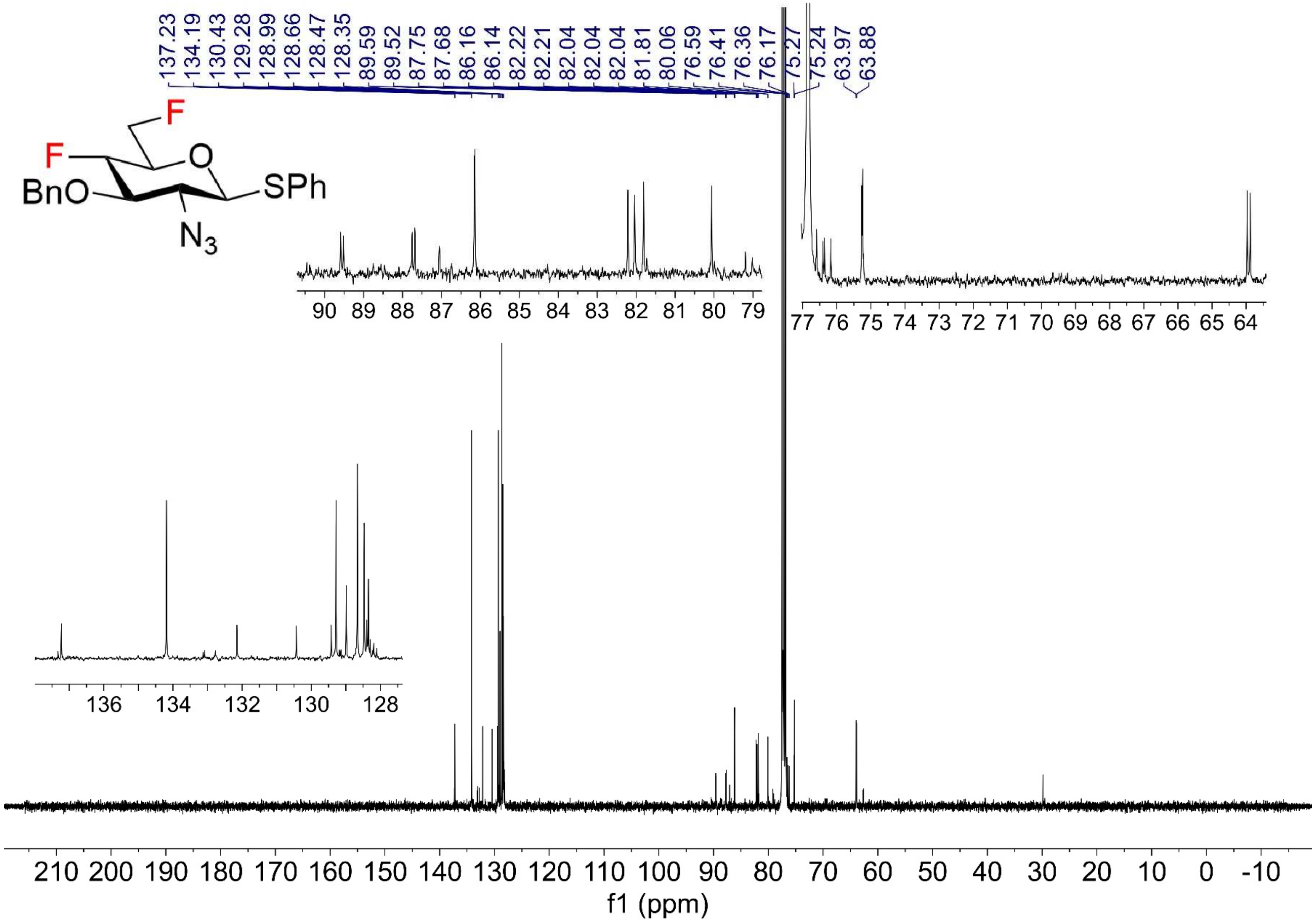

^{19}F NMR (376 MHz, CDCl$_3$) β-23

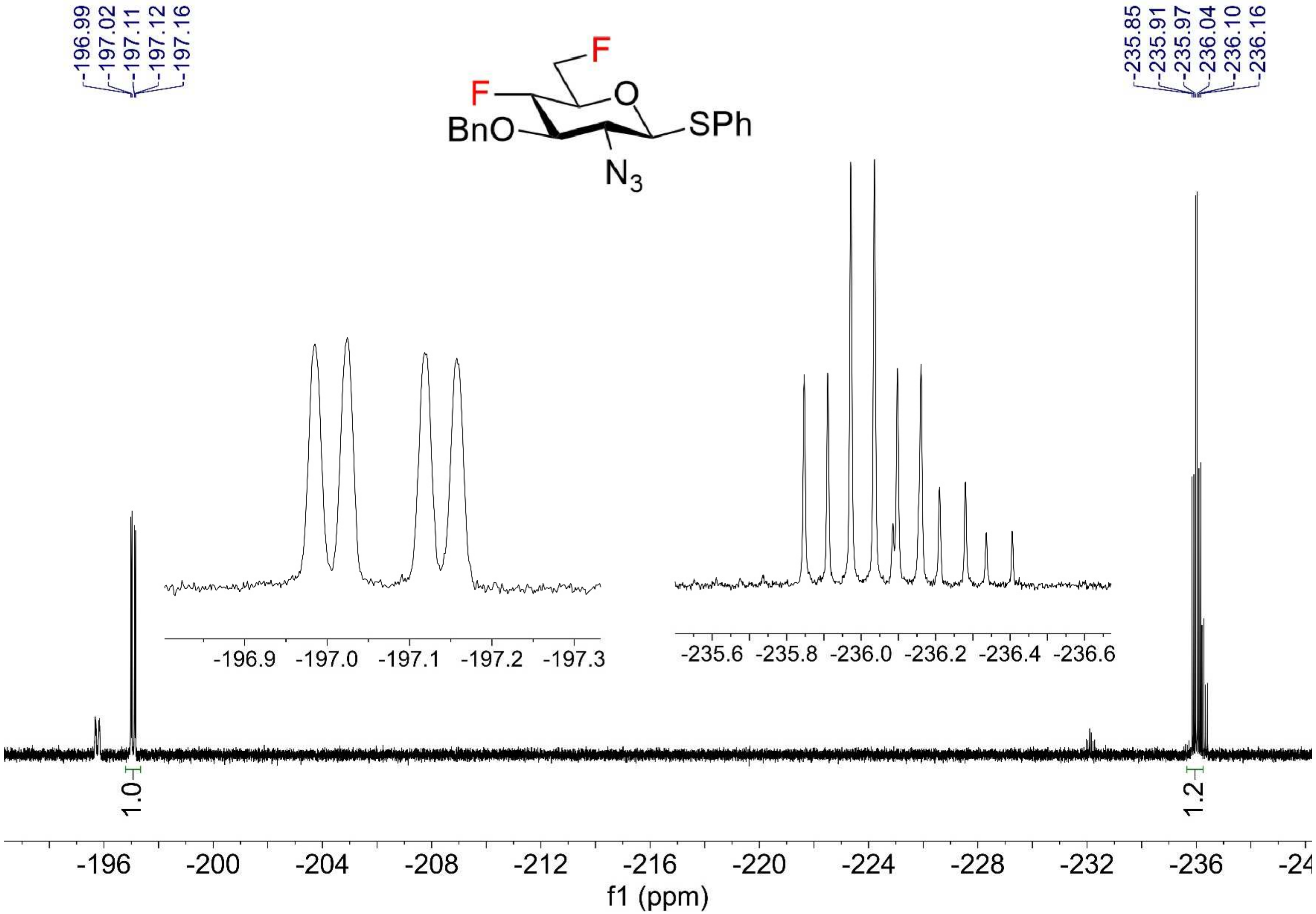

^{1}H-^{1}H COSY β-23

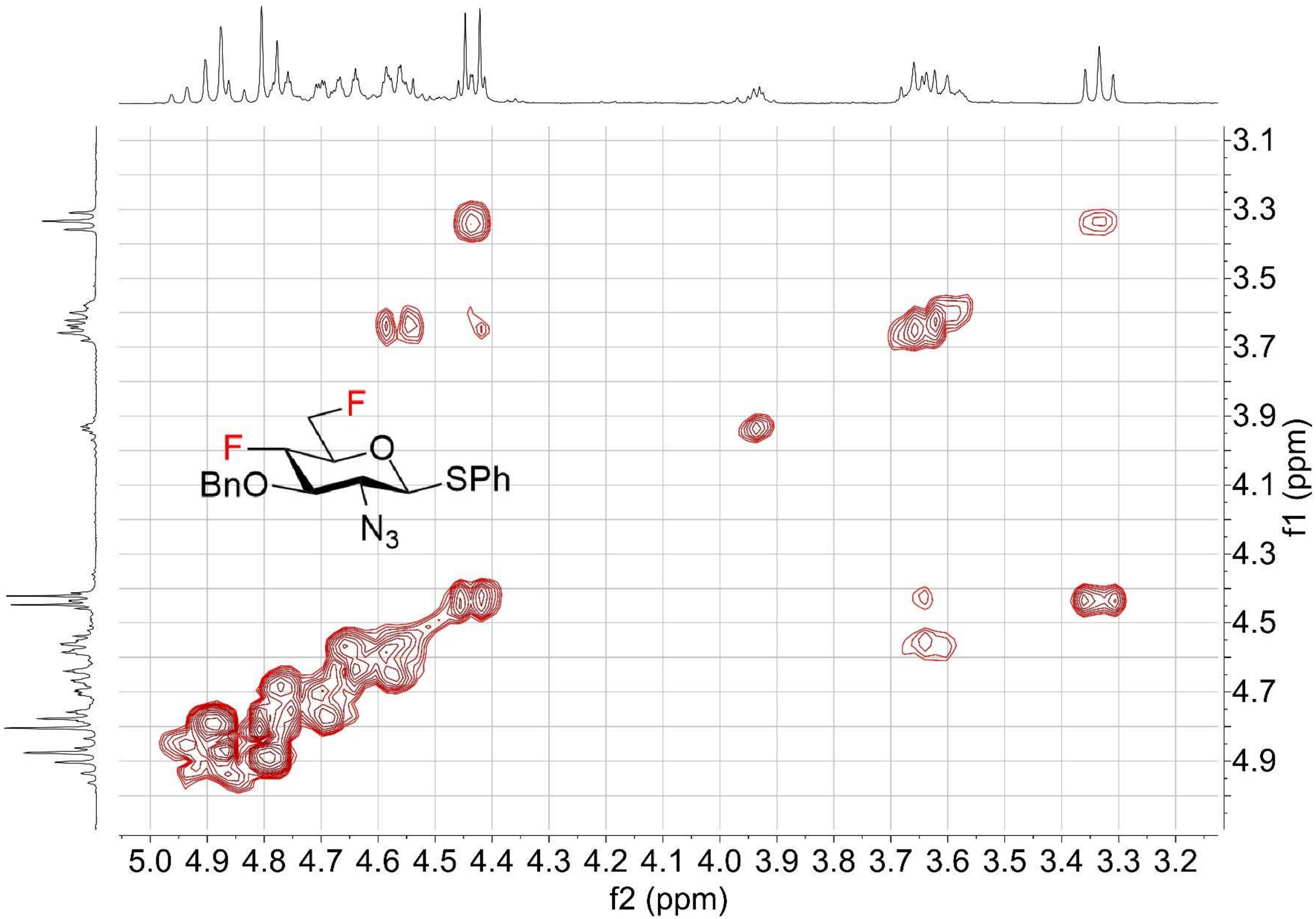

^{1}H-^{13}C HSQC β-23

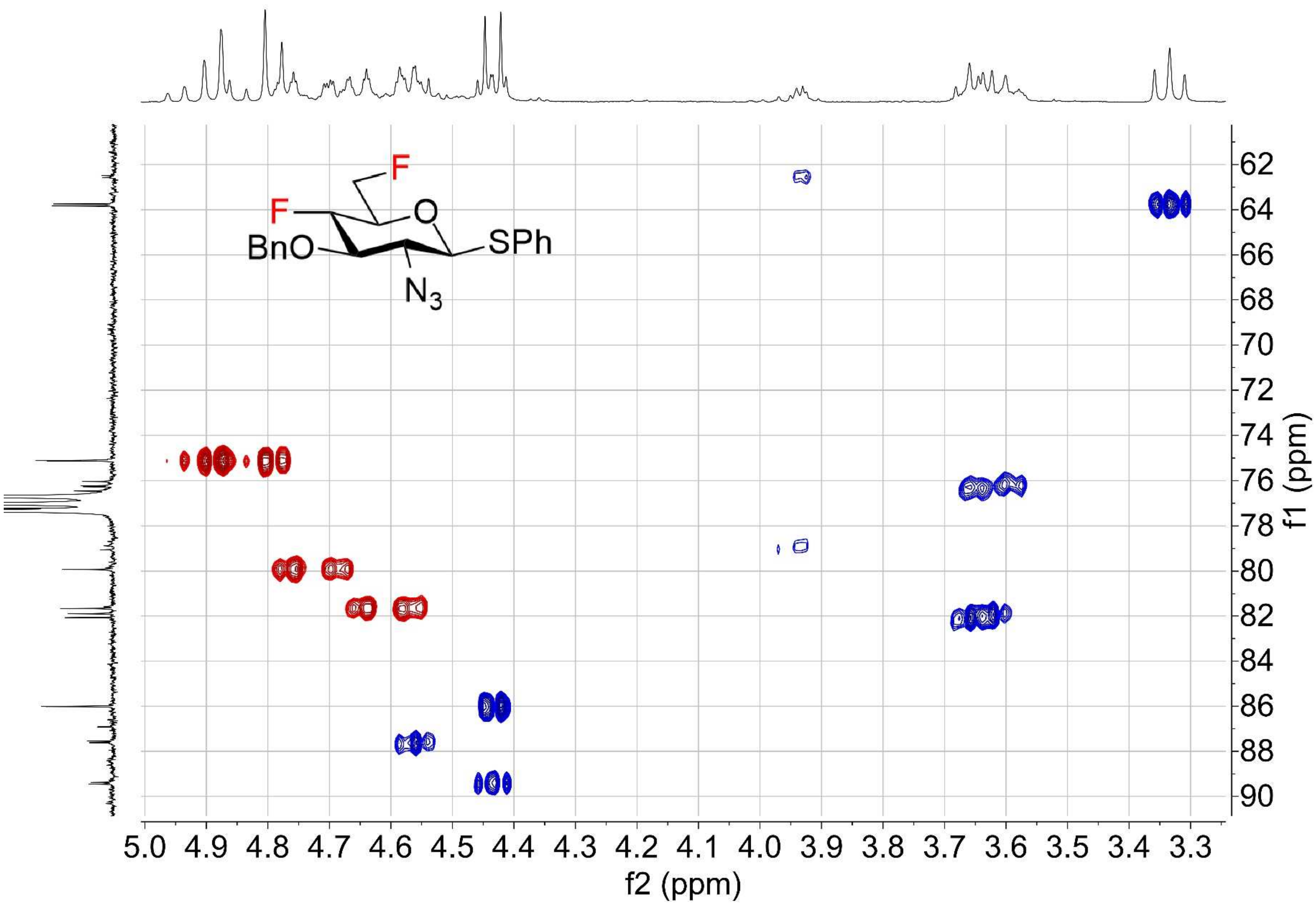

^{1}H-^{13}C HMBC β-23

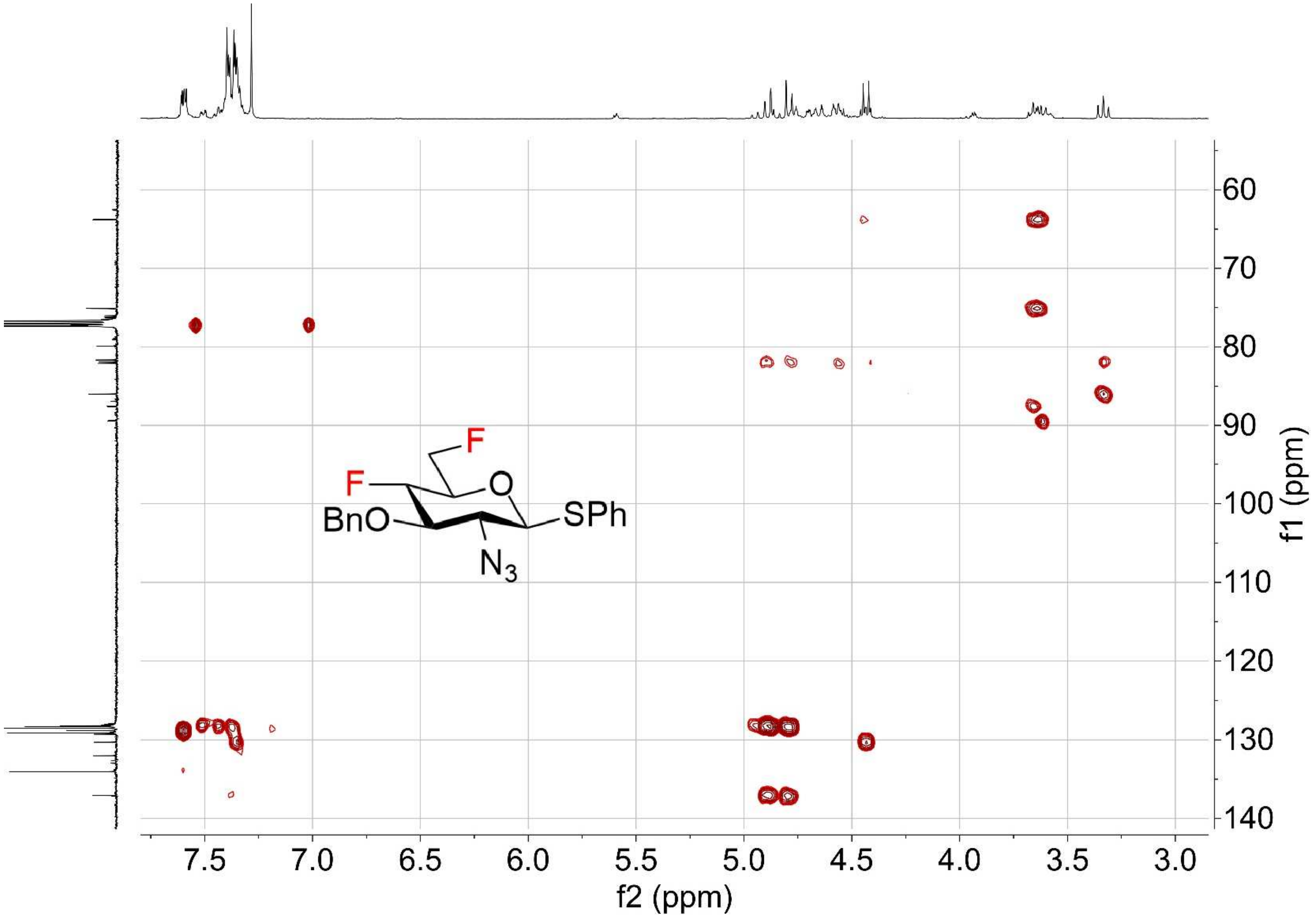

NMR COMPOUND α-24

^{1}H NMR (400 MHz, CDCl$_3$) α-24

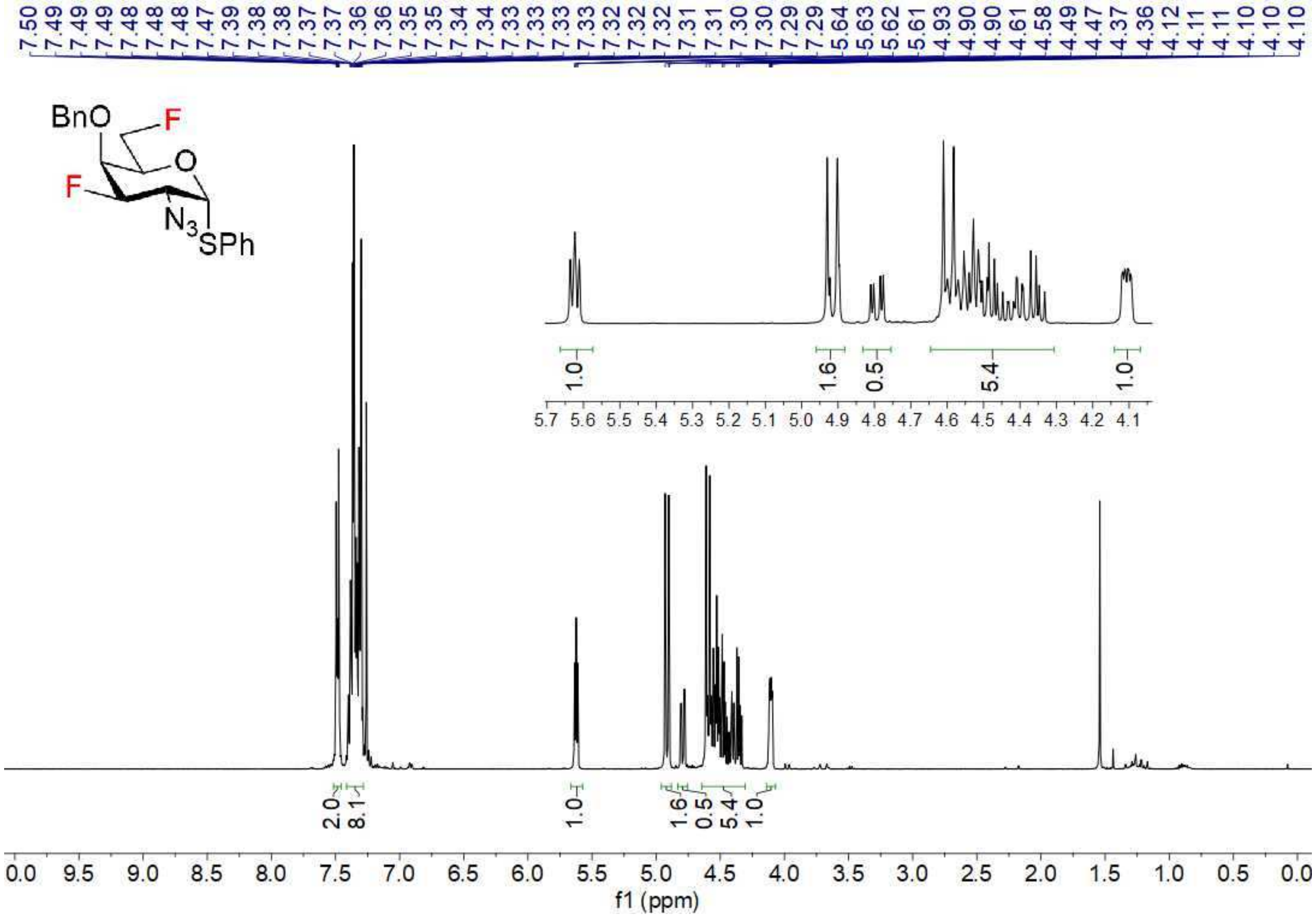

^{13}C NMR (100 MHz, CDCl$_3$) α-24

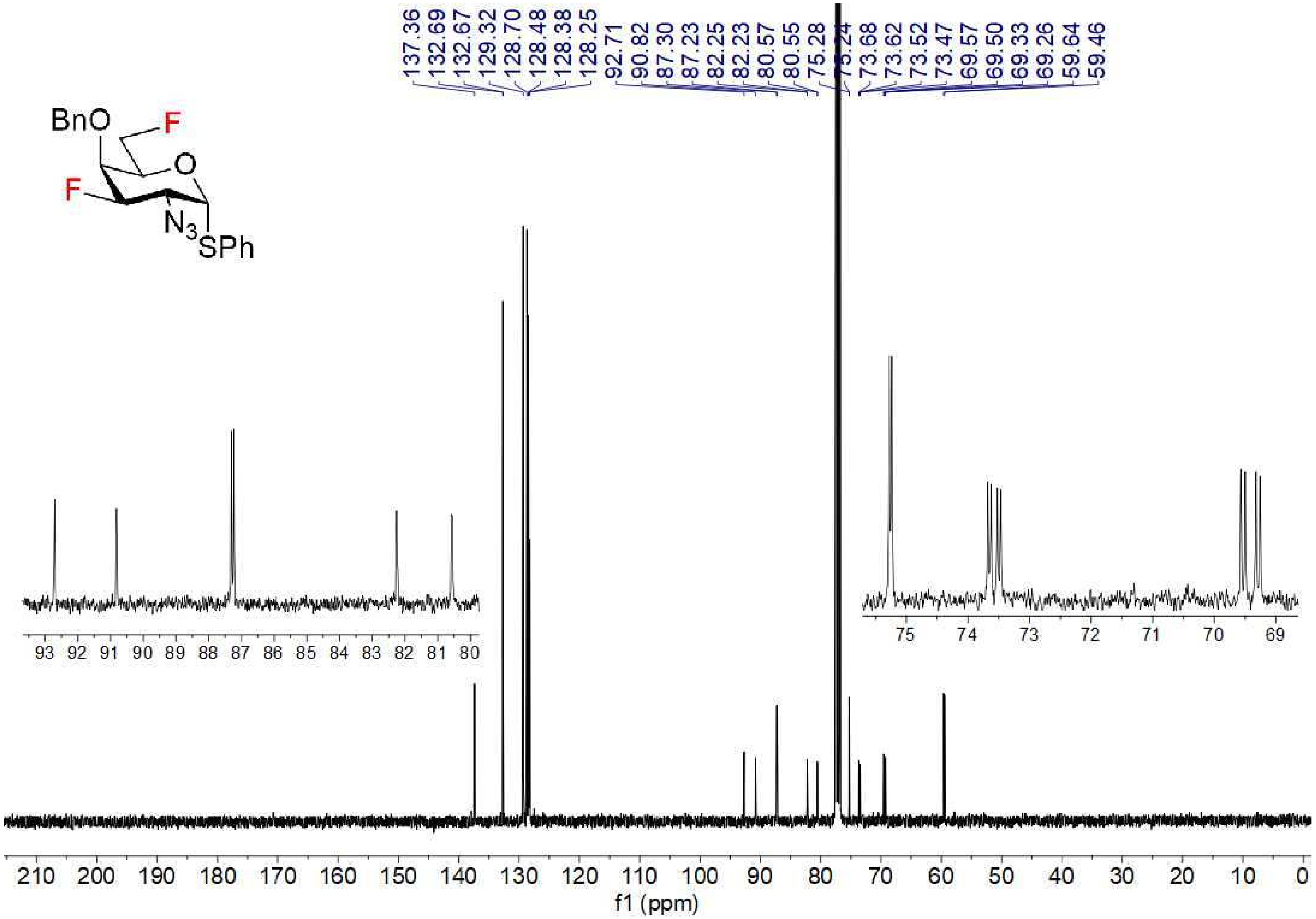

^{19}F NMR (376 MHz, $CDCl_3$) α-24

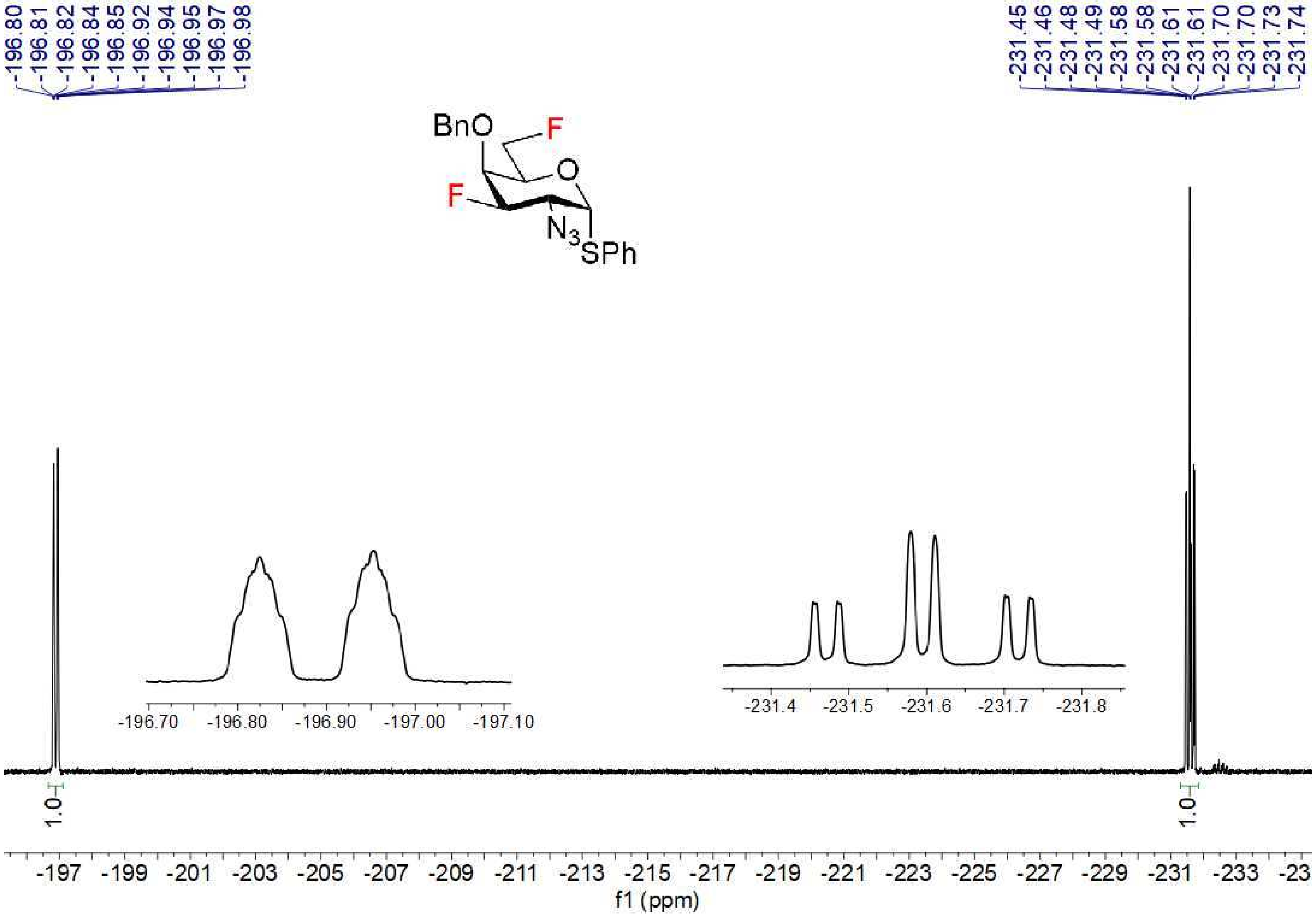

^{1}H-^{1}H COSY α-24

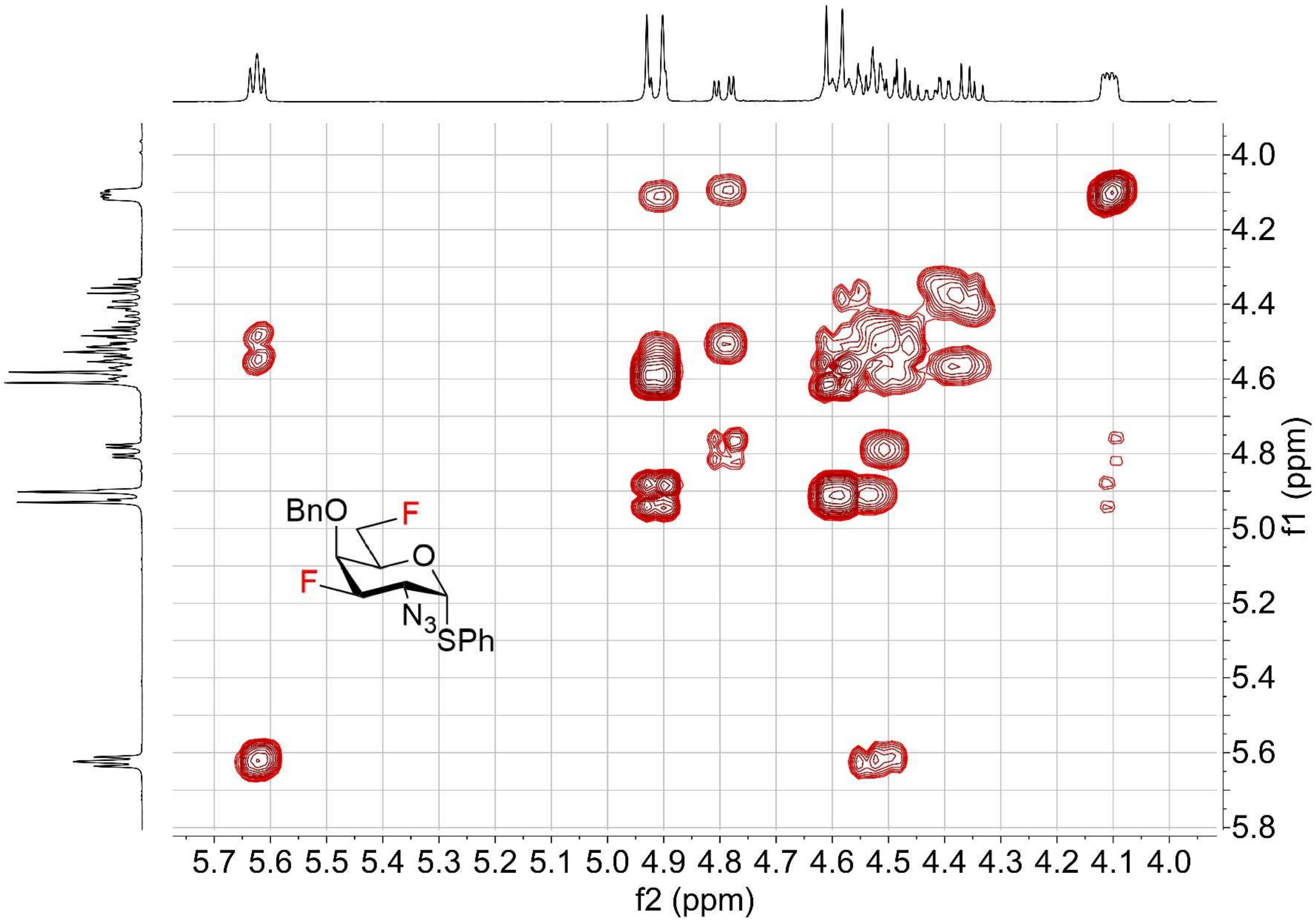

^{1}H-^{13}C HSQC α-24

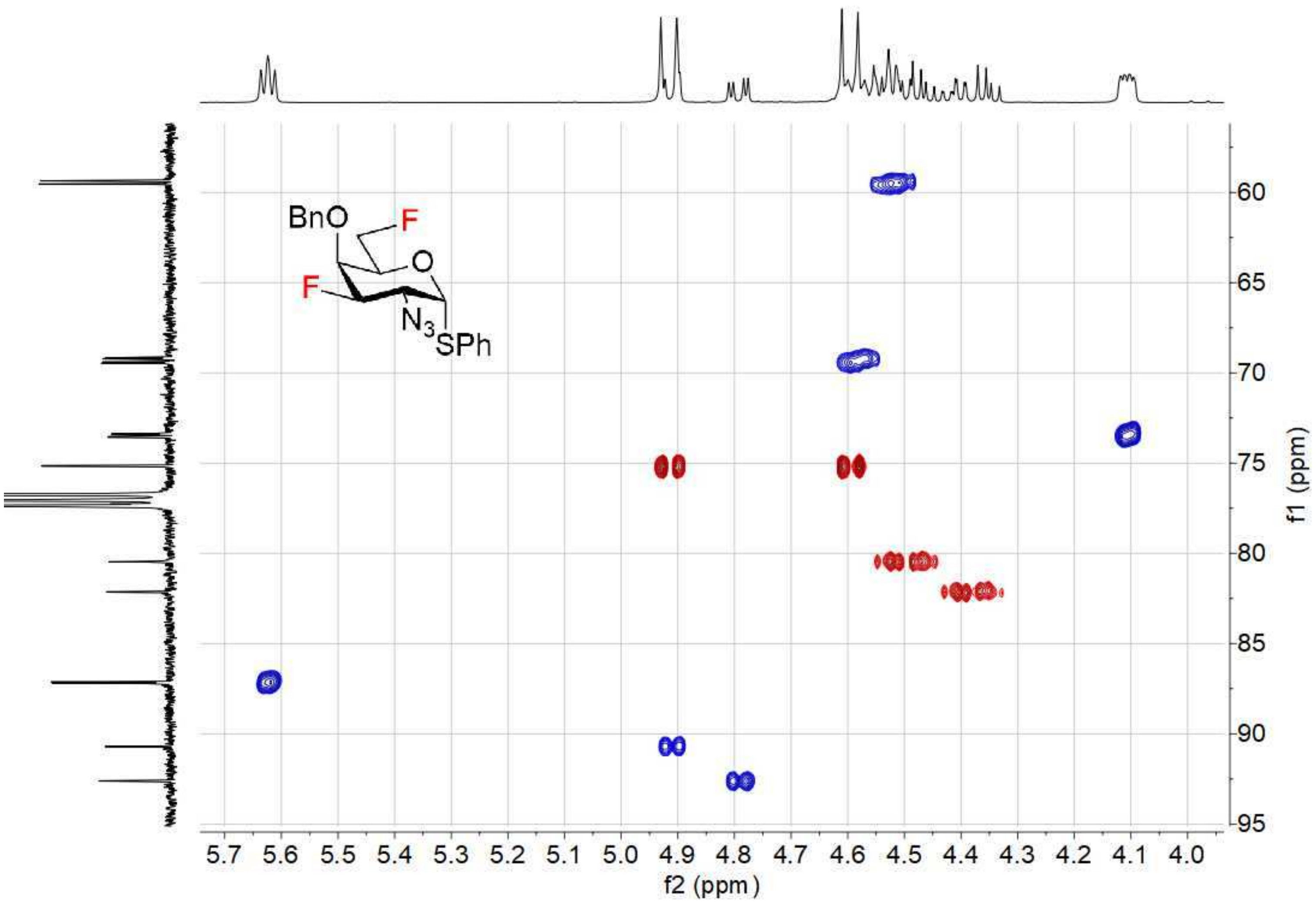

NMR COMPOUND β-24

^{1}H NMR (400 MHz, $CDCl_3$) β-24

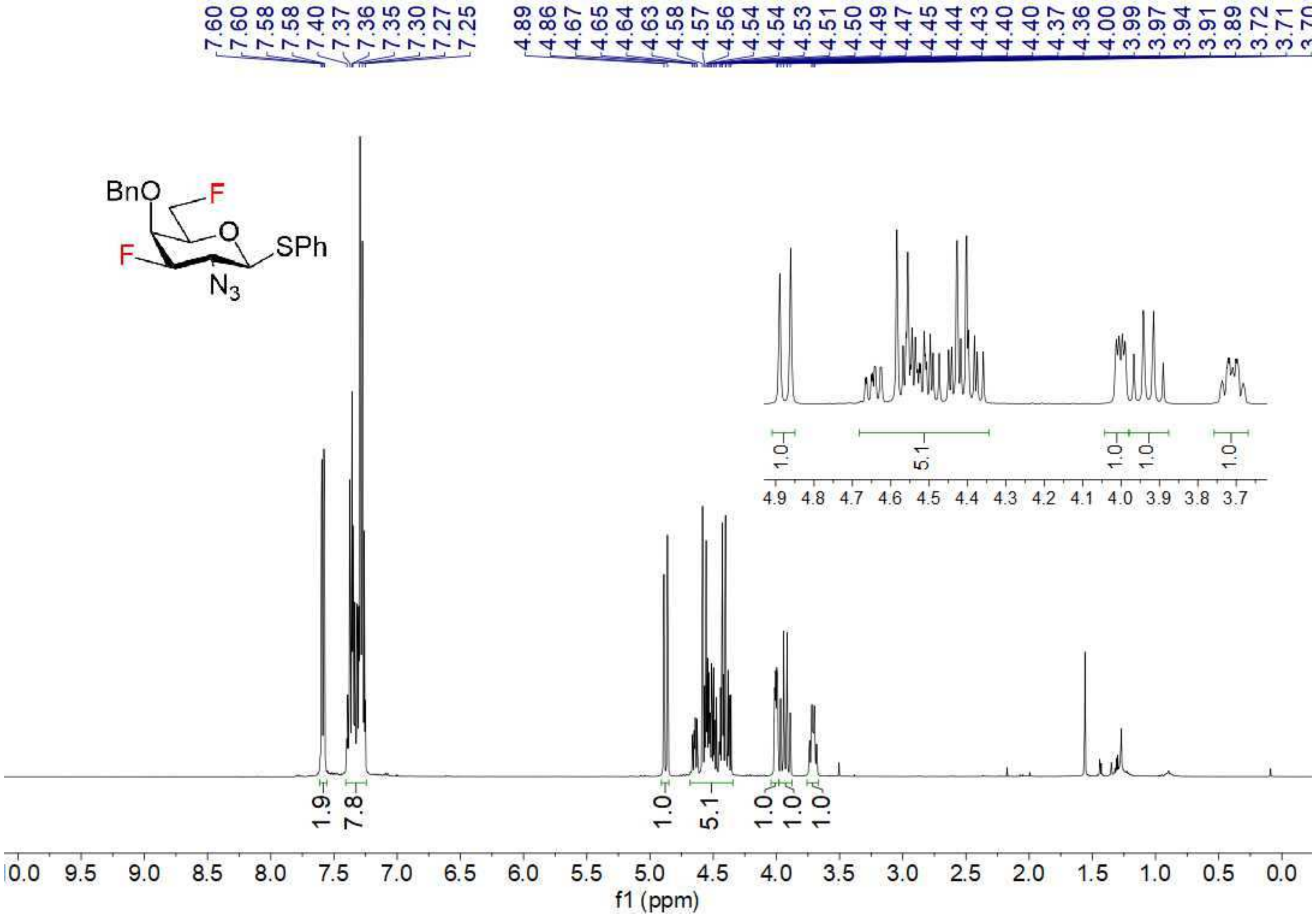

^{13}C NMR (100 MHz, CDCl$_3$) β-24

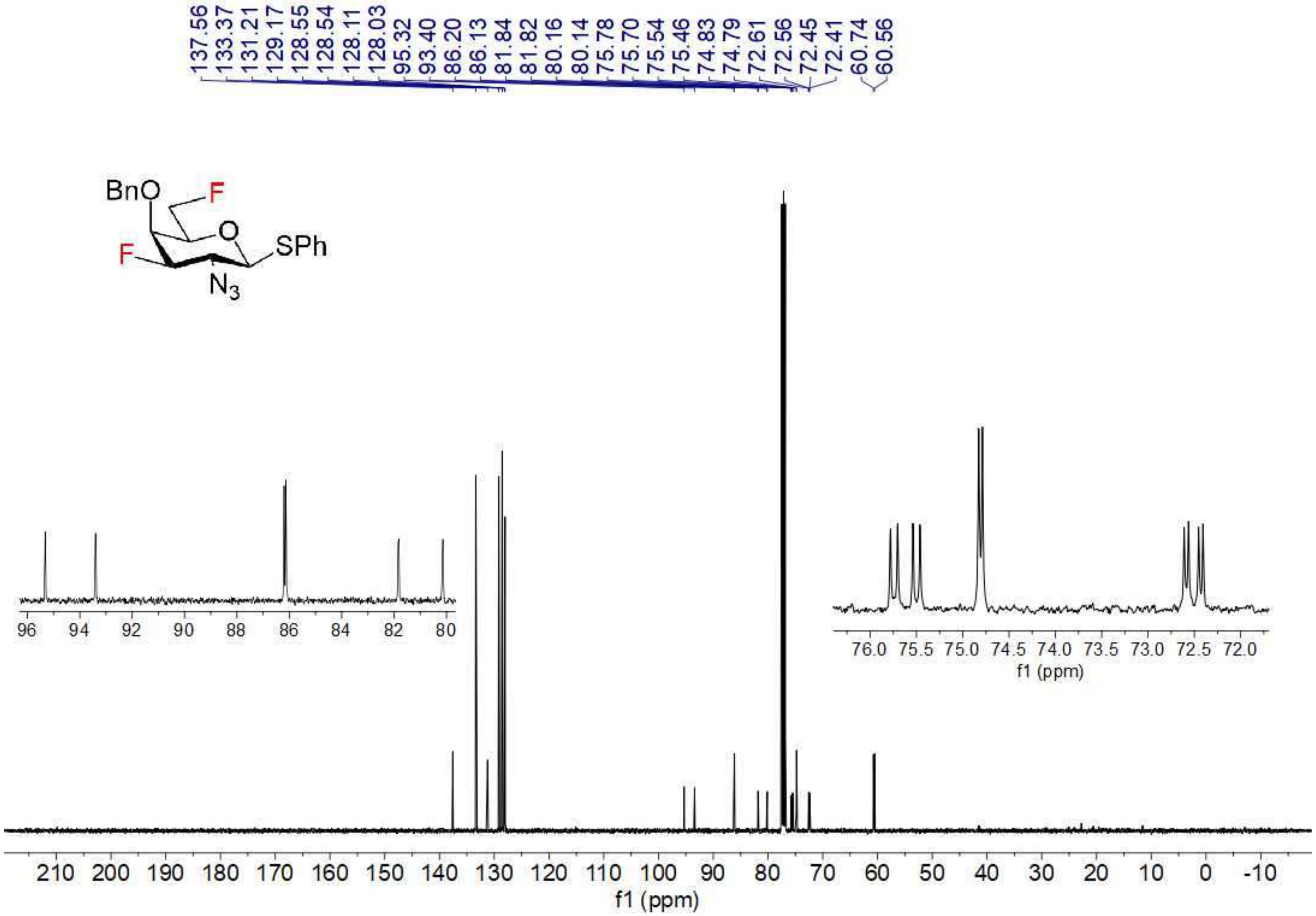

^{19}F NMR (376 MHz, CDCl$_3$) β-24

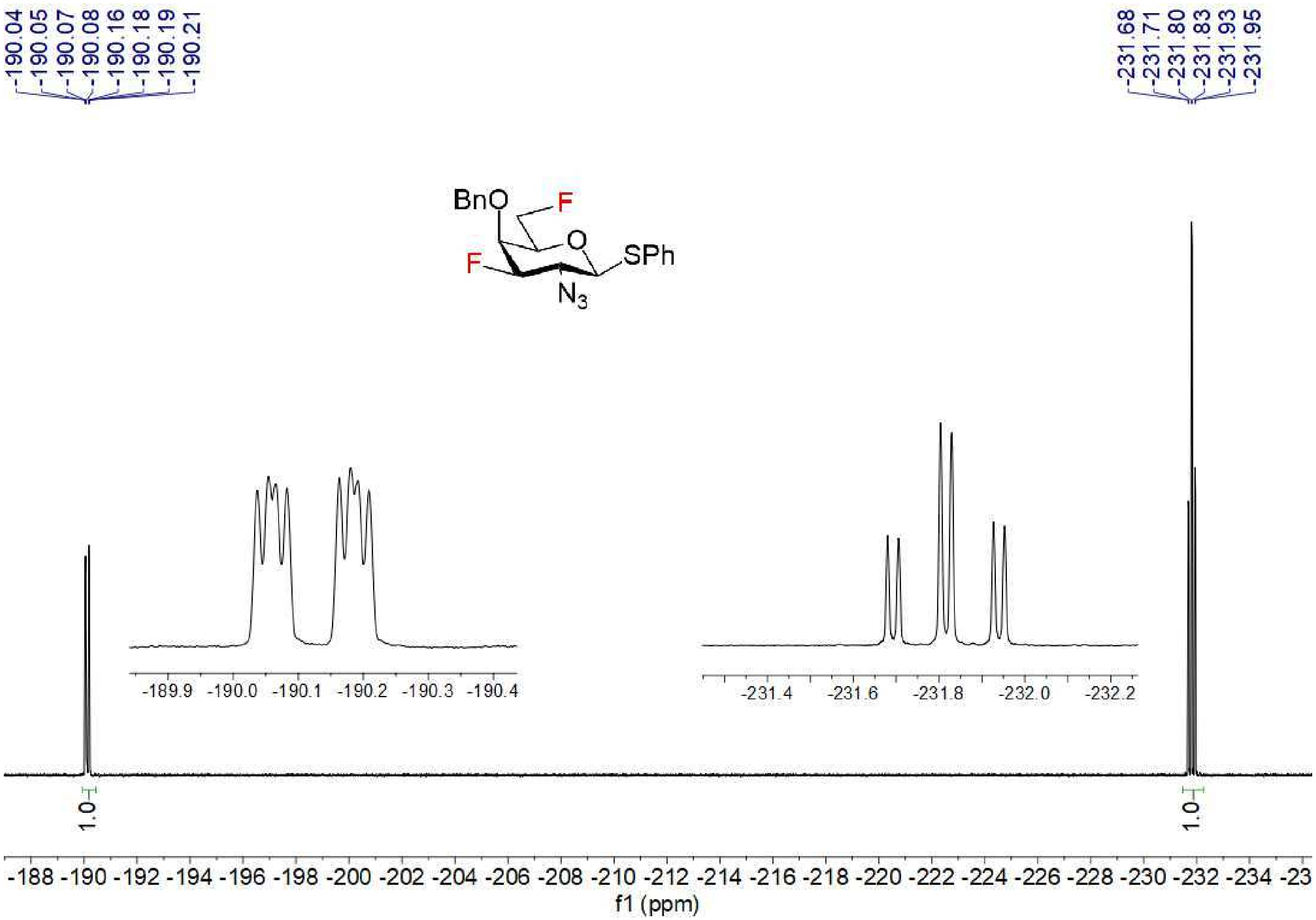

^{1}H-^{1}H COSY β-24

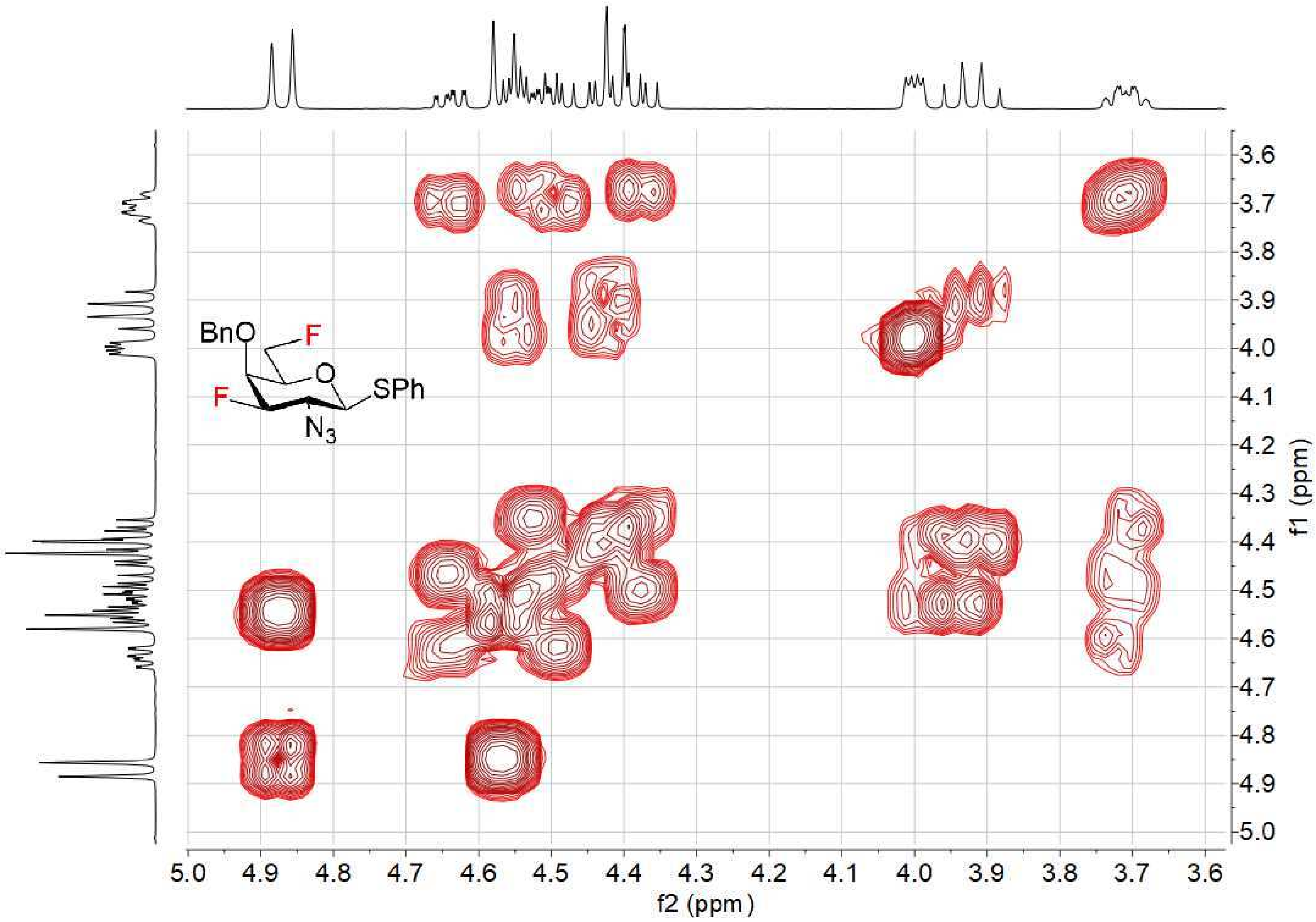

^{1}H-^{13}C HSQC β-24

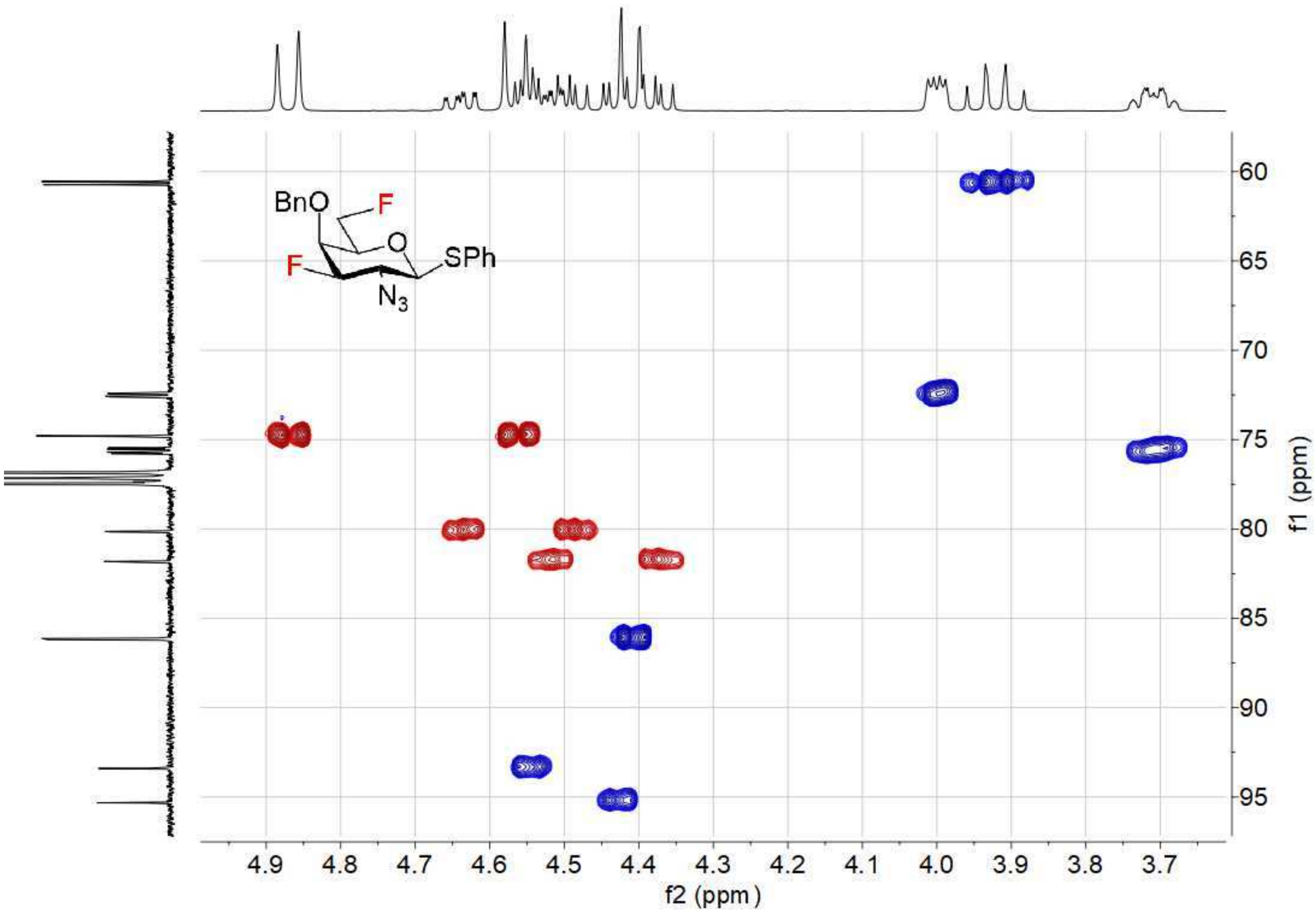

NMR COMPOUND 25

^{1}H NMR (400 MHz, CDCl$_3$) 25

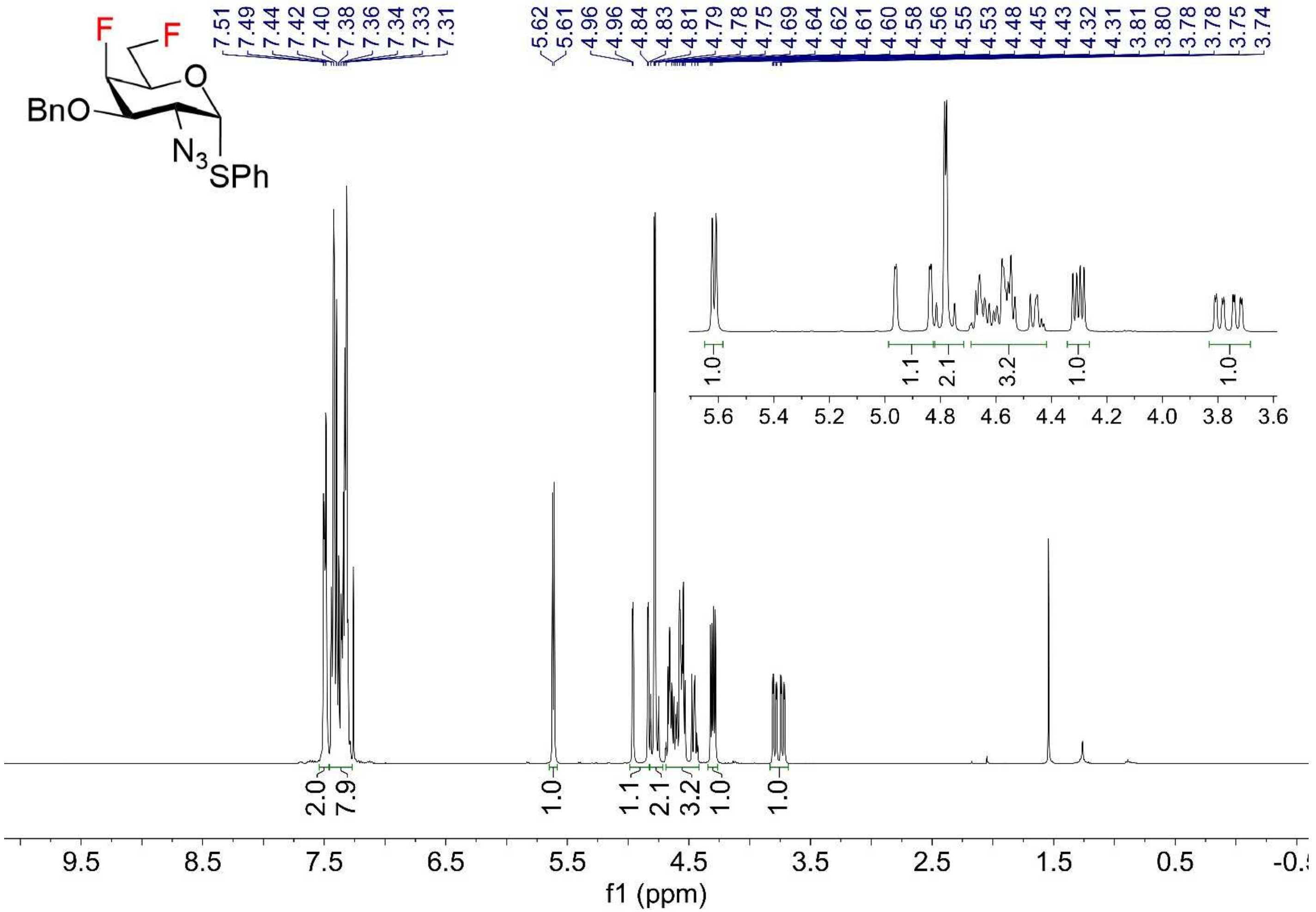

^{13}C NMR (100 MHz, CDCl$_3$) 25

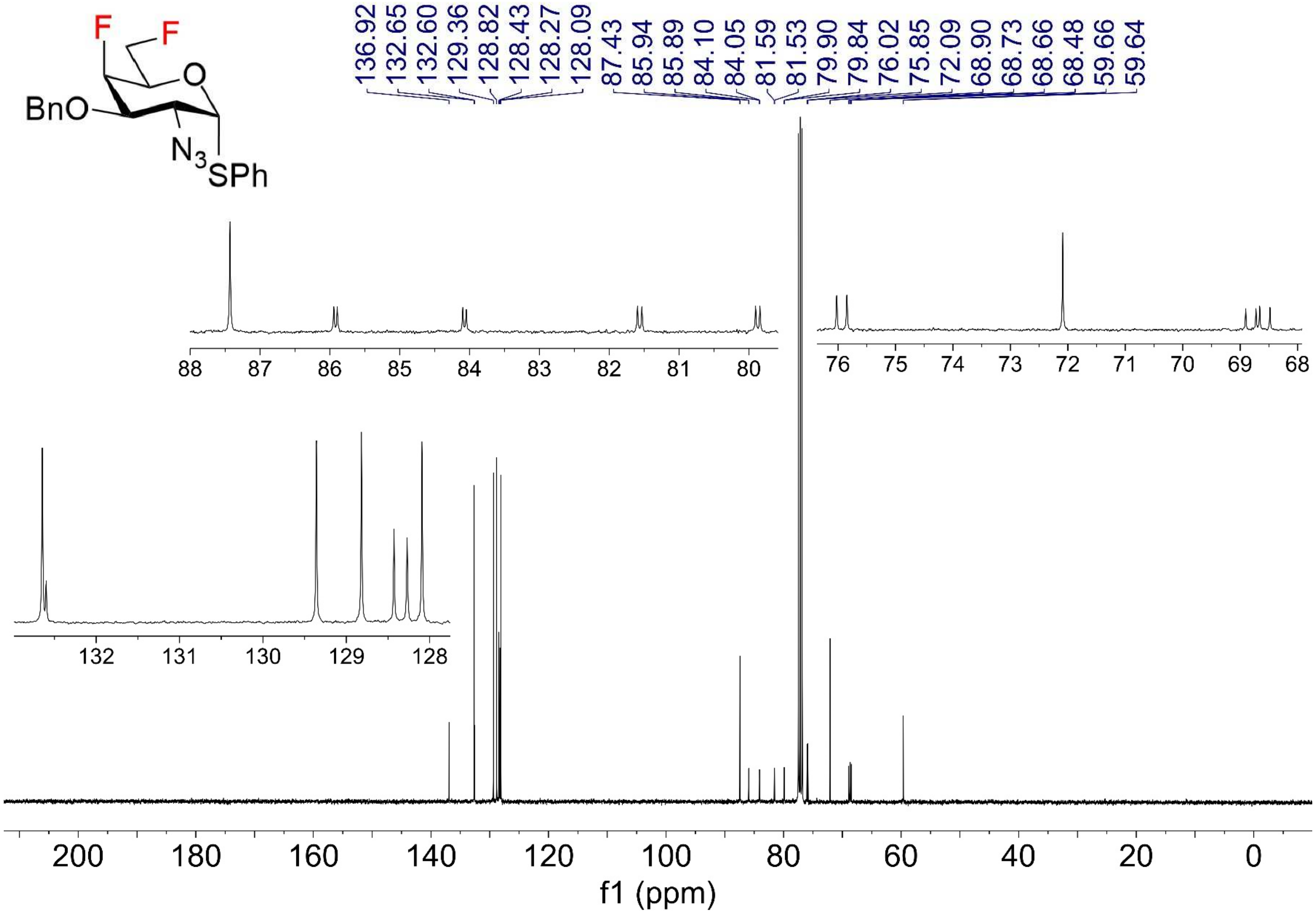

^{19}F NMR (376 MHz, CDCl$_3$) 25

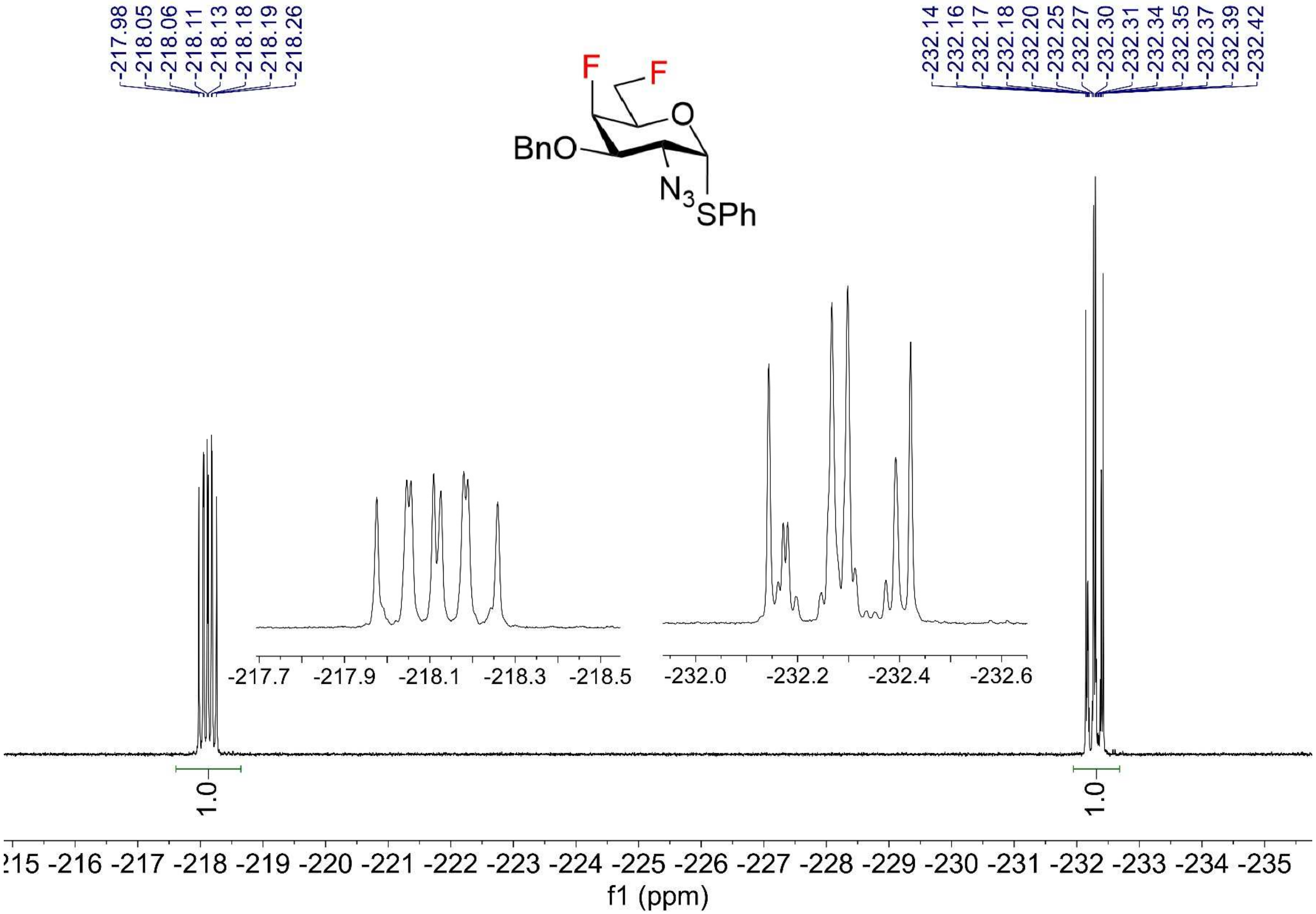

^{1}H-^{1}H COSY 25

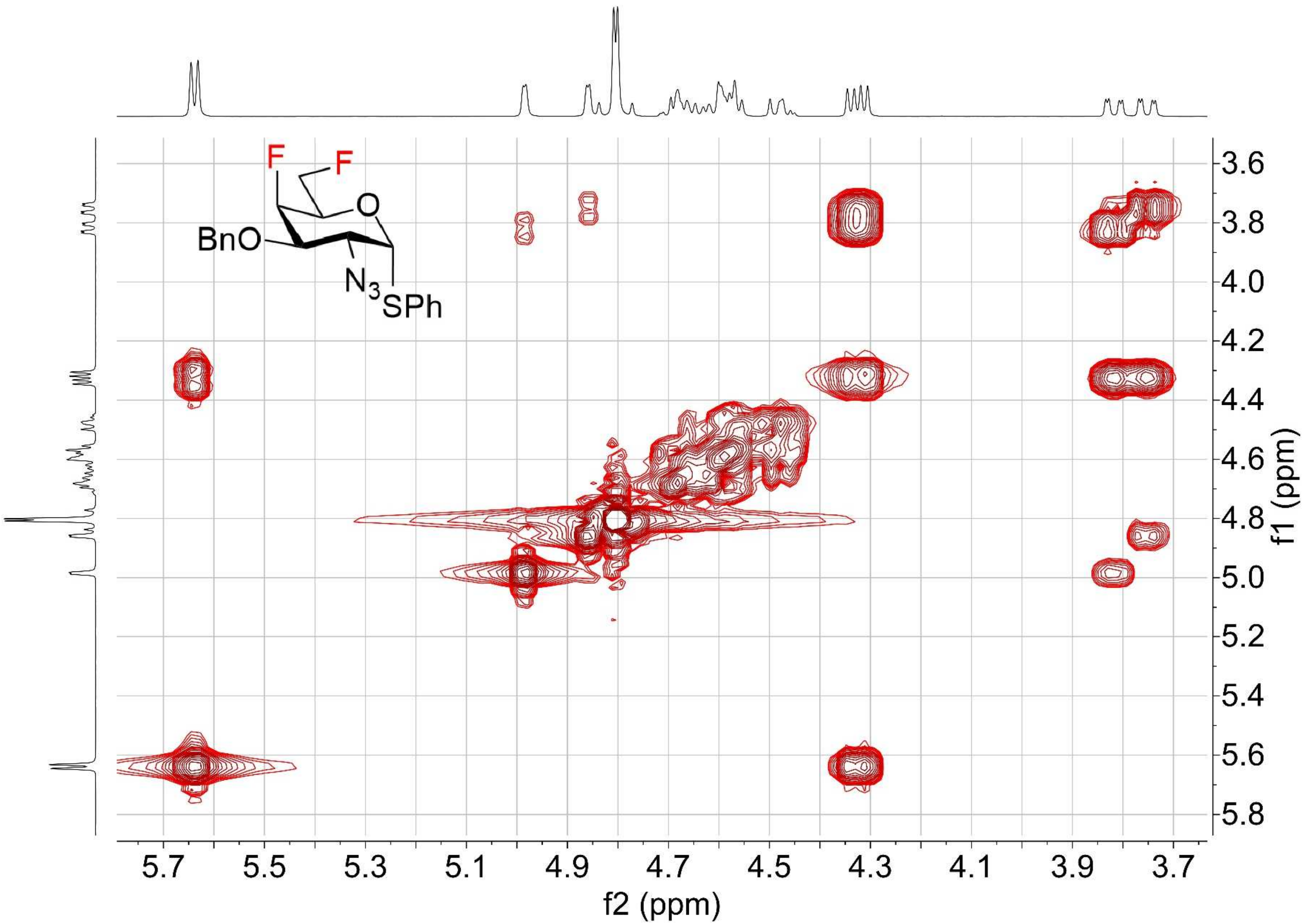
F
F
O
BnO
N3
SPh
3.6
3.8
4.0
4.2
4.4
4.6
4.8
5.0
5.2
5.4
5.6
5.8
f1 (ppm)
5.7
5.5
5.3
5.1
4.9
4.7
4.5
4.3
4.1
3.9
3.7
f2 (ppm)

^{1}H-^{13}C HSQC 25

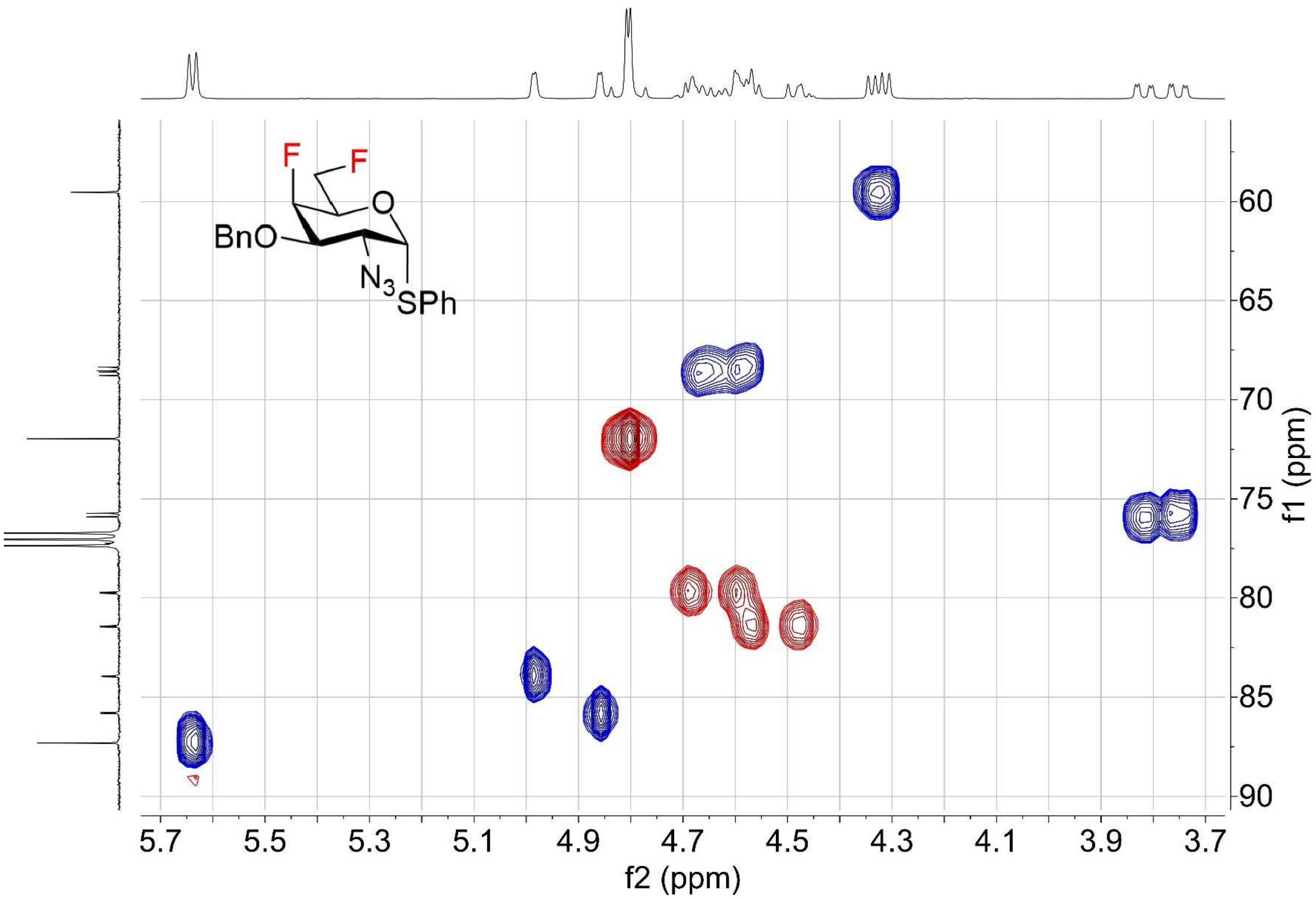

NMR COMPOUND 26

^{1}H NMR (400 MHz, $CDCl_3$) 26

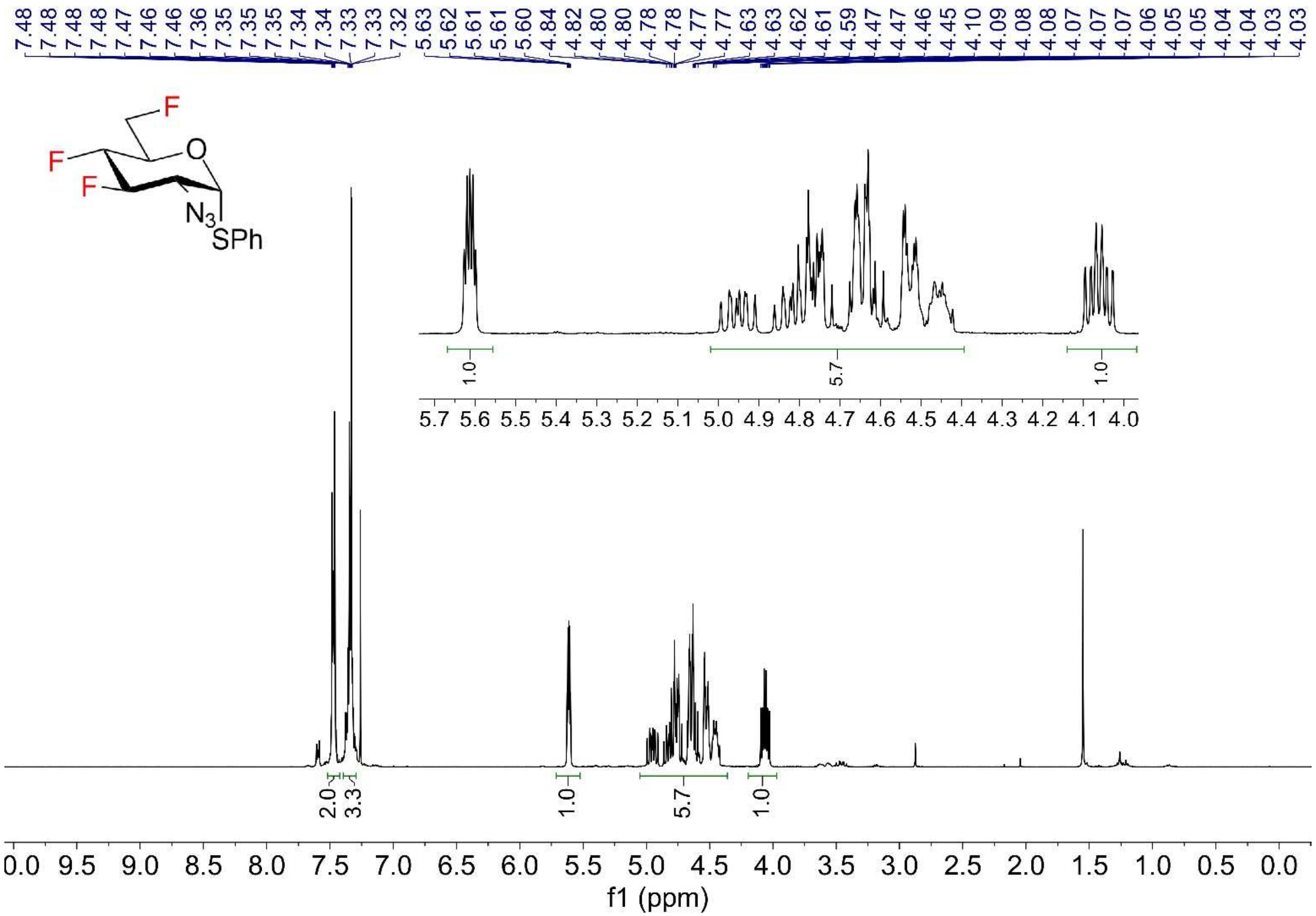

^{13}C NMR (100 MHz, CDCl$_3$) 26

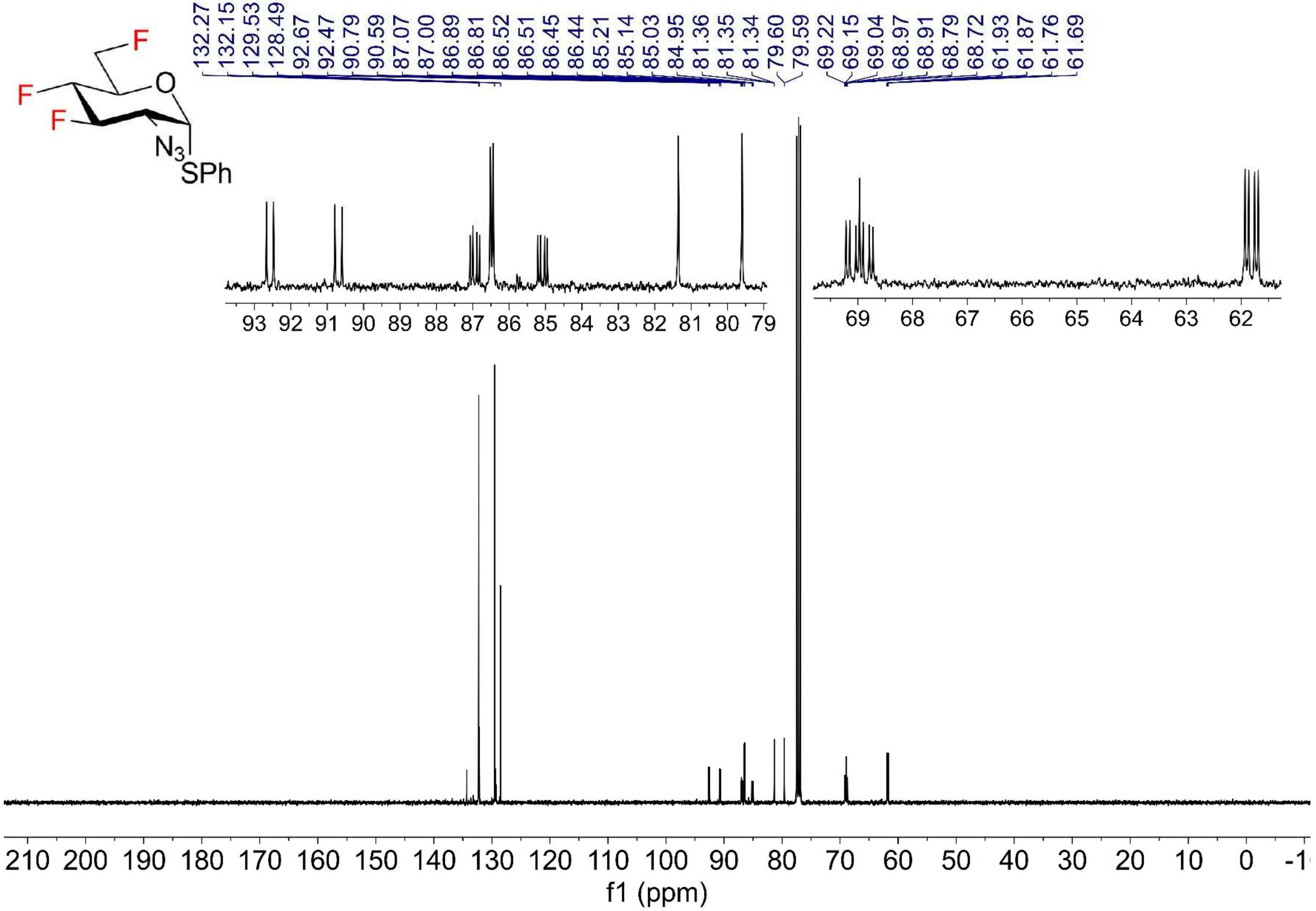

^{19}F NMR (376 MHz, CDCl$_3$) 26

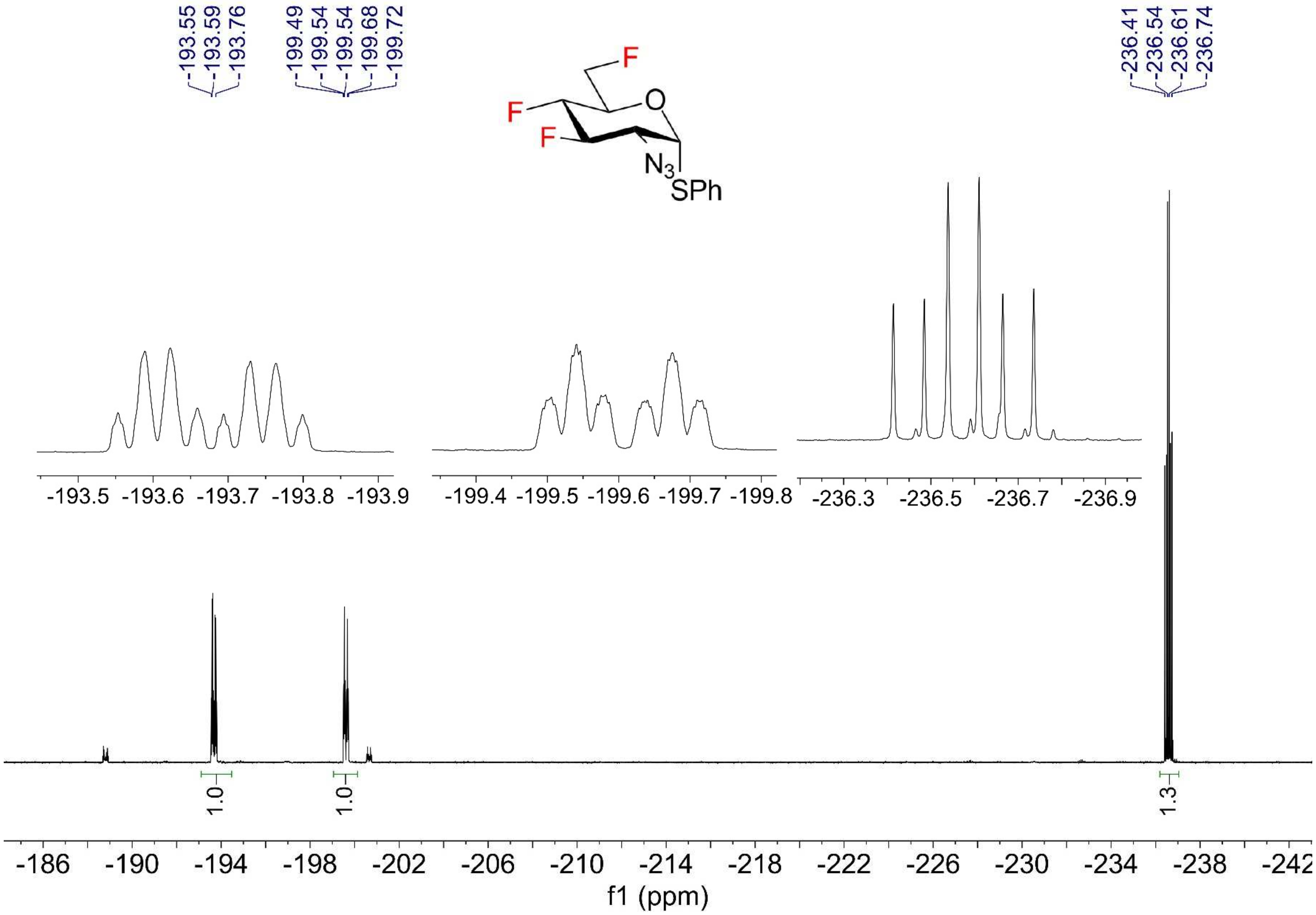

^{1}H-^{1}H COSY 26

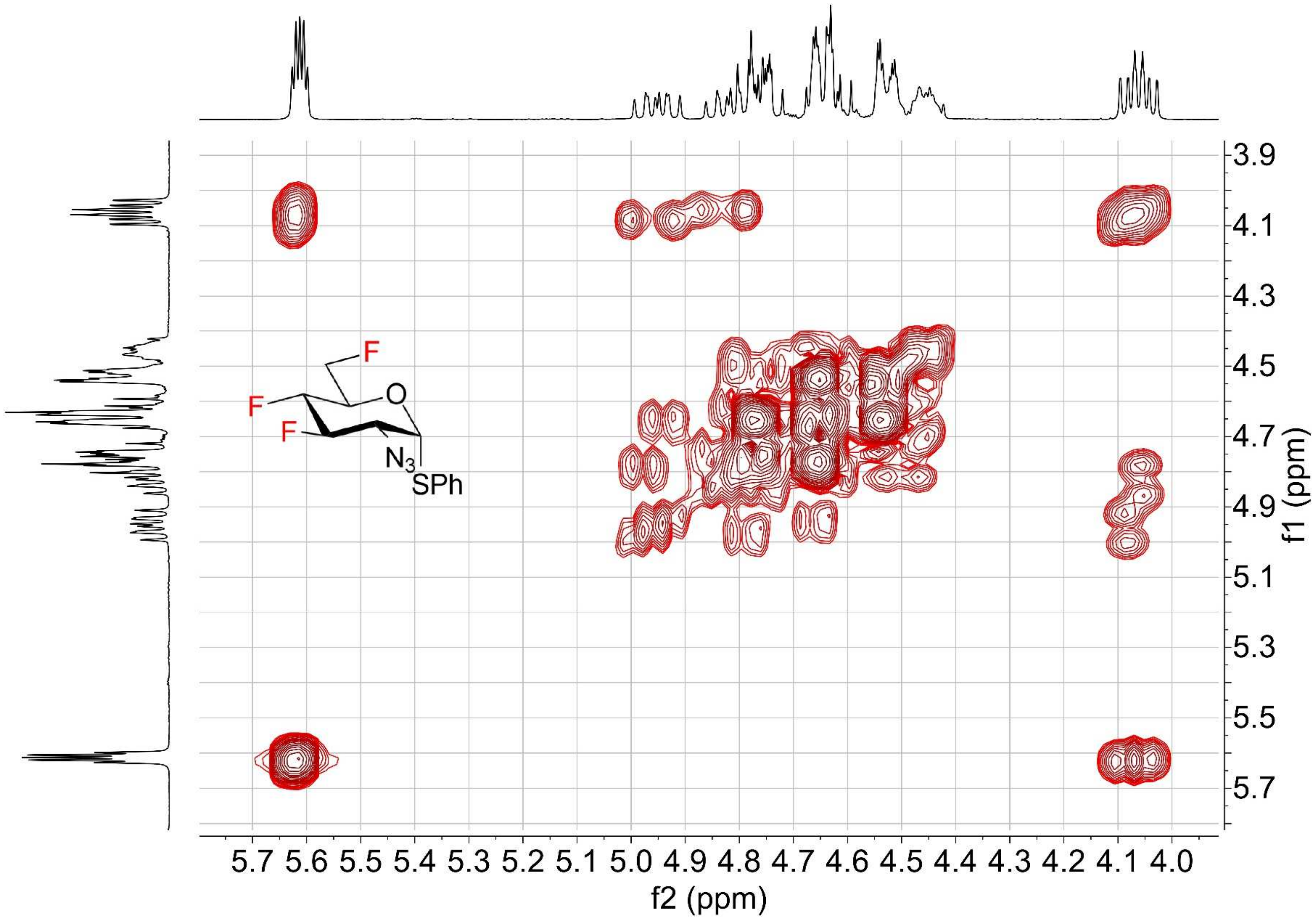
F
F
O
F
N3
SPh
3.9
4.1
4.3
4.5
4.7
4.9
5.1
5.3
5.5
5.7
f1 (ppm)
5.7 5.6 5.5 5.4 5.3 5.2 5.1 5.0 4.9 4.8 4.7 4.6 4.5 4.4 4.3 4.2 4.1 4.0
f2 (ppm)

^{1}H-^{13}C HSQC 26

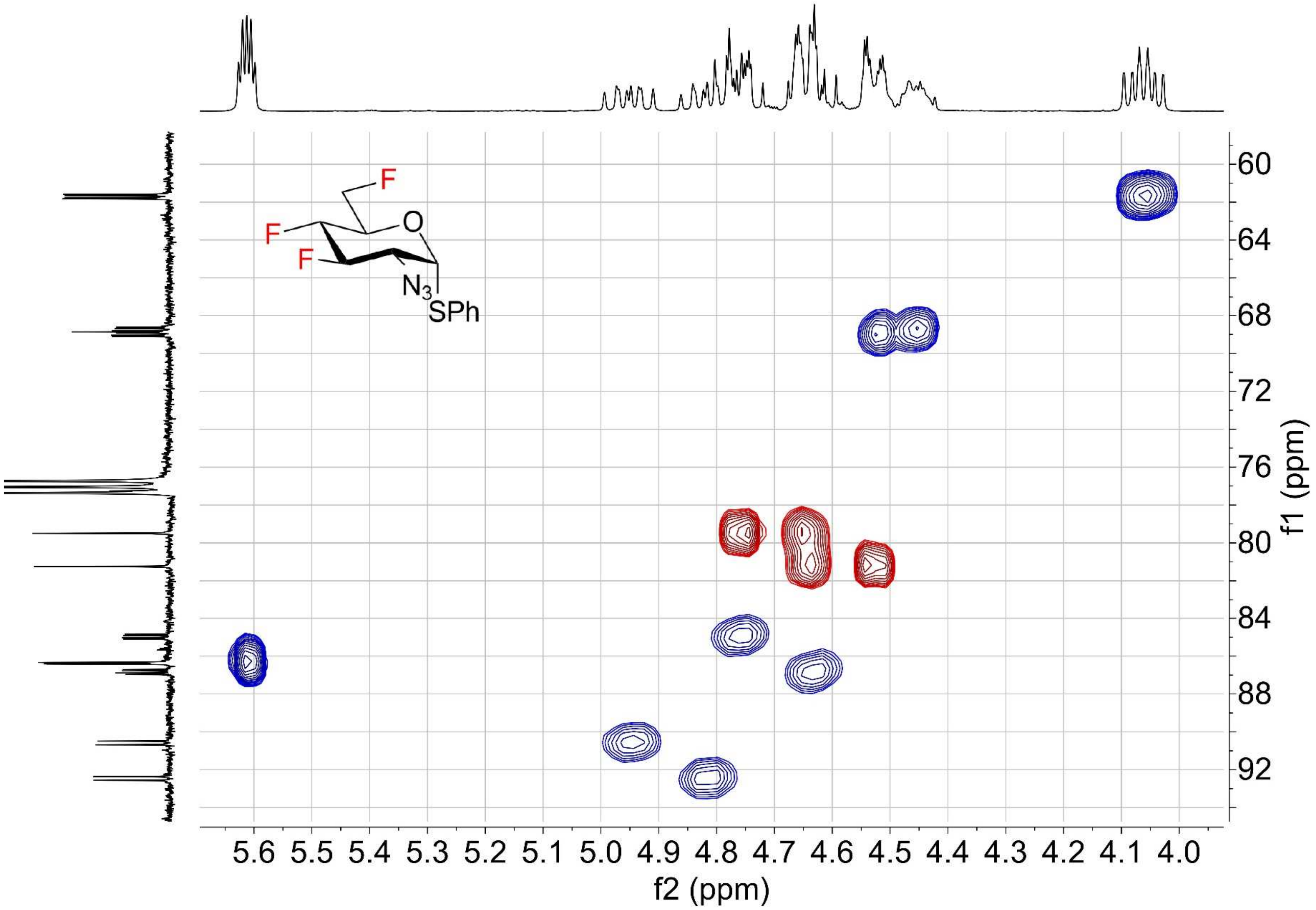

NMR COMPOUND 31

^{1}H NMR (400 MHz, CDCl$_3$) 31

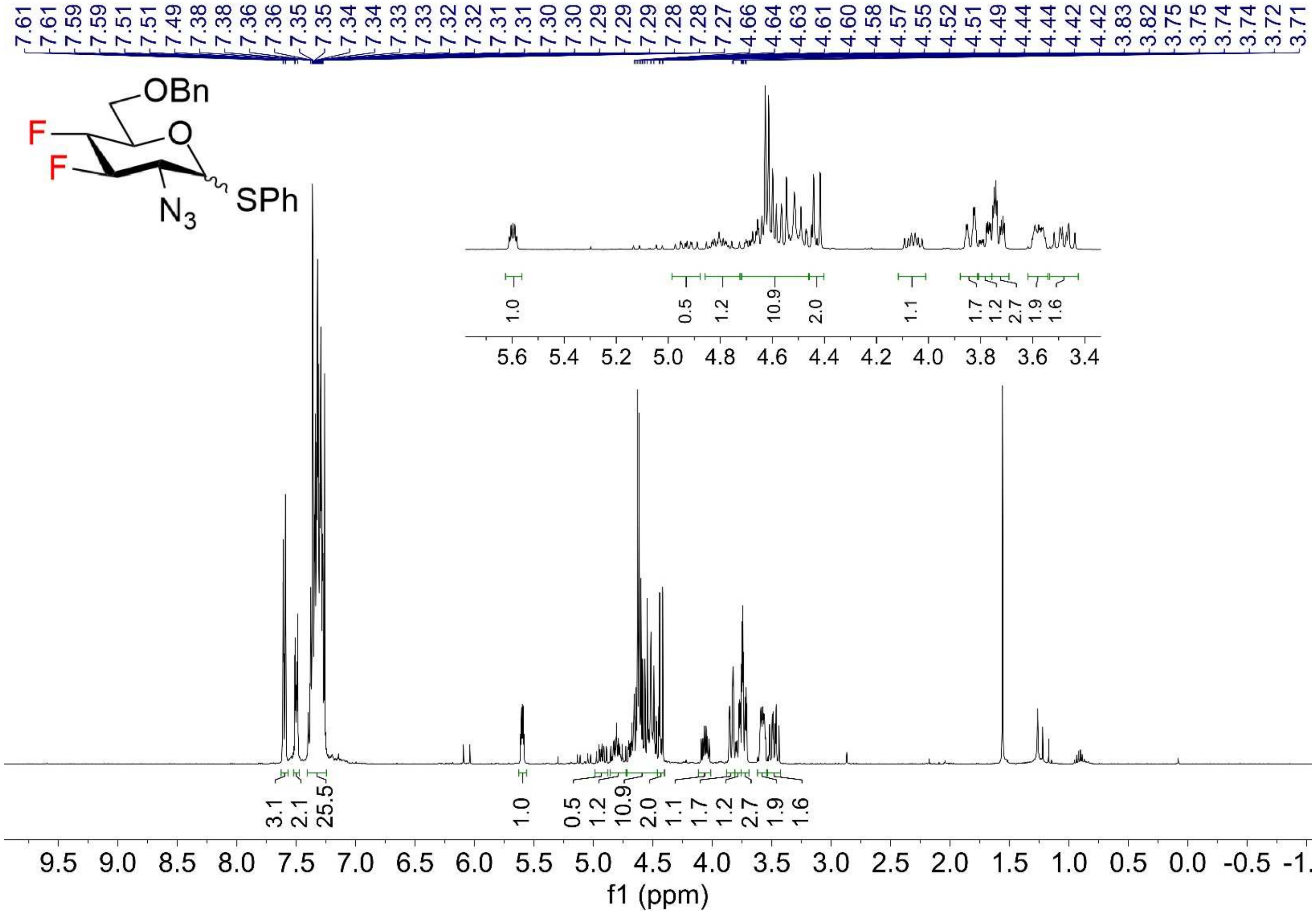

^{13}C NMR (100 MHz, $CDCl_3$) 31

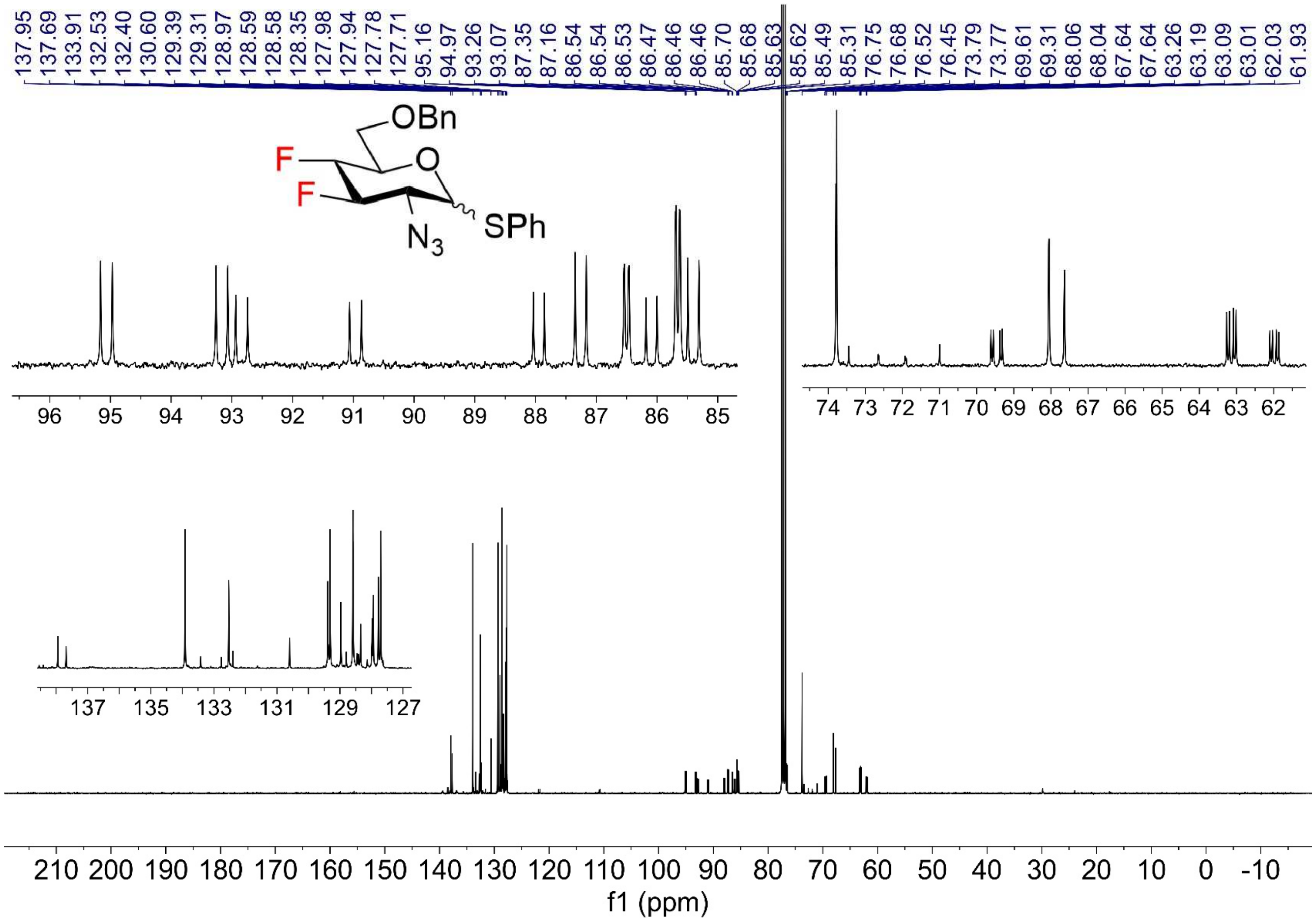

^{19}F NMR (376 MHz, CDCl$_3$) 31

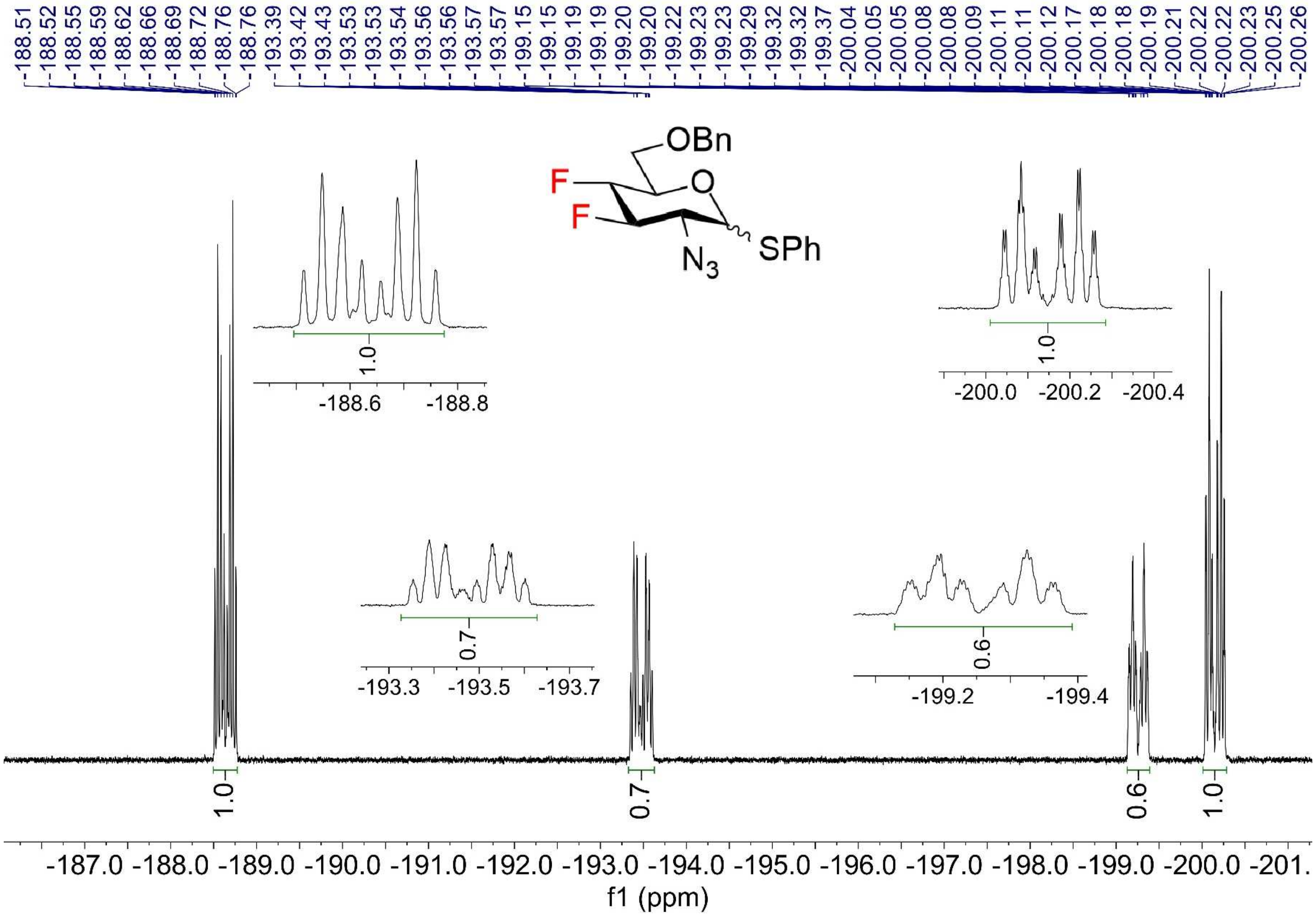

^{1}H-^{1}H COSY 31

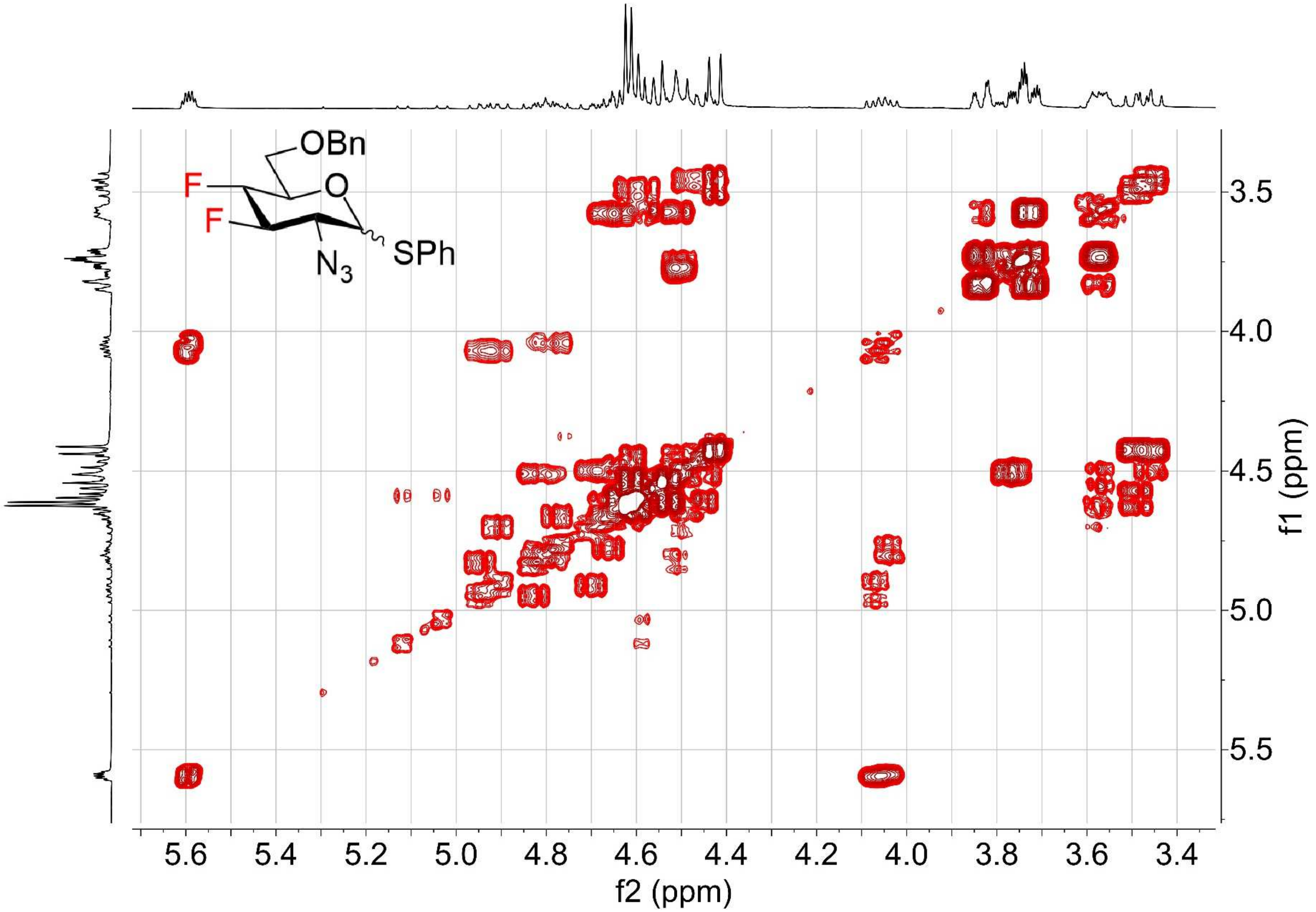

OBn
F
F
O
N3
SPh
5.6
5.4
5.2
5.0
4.8
4.6
4.4
4.2
4.0
3.8
3.6
3.4
f2 (ppm)
3.5
4.0
4.5
5.0
5.5
f1 (ppm)

^{1}H-^{13}C HSQC 31

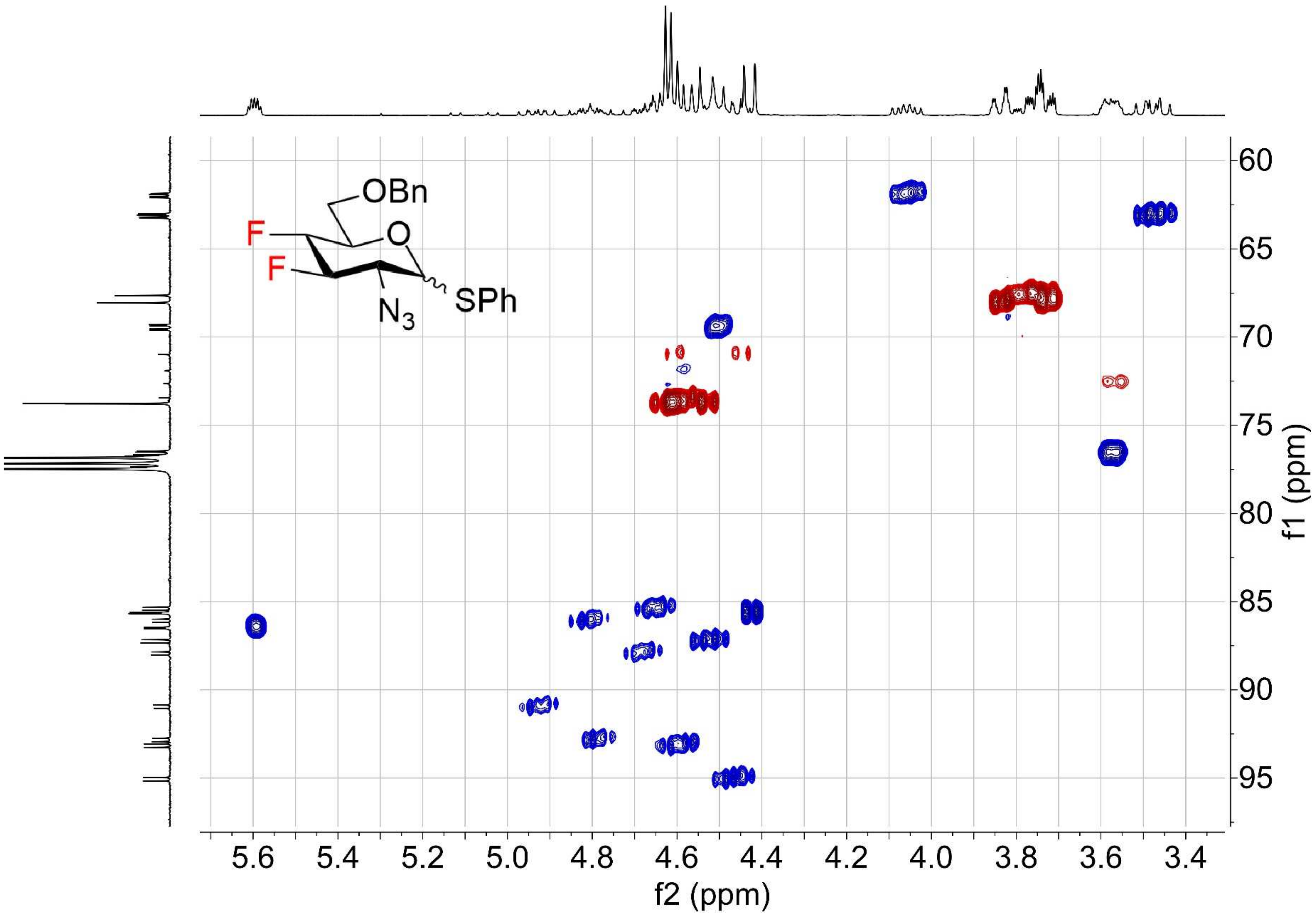

^{1}H-^{13}C HMBC 31

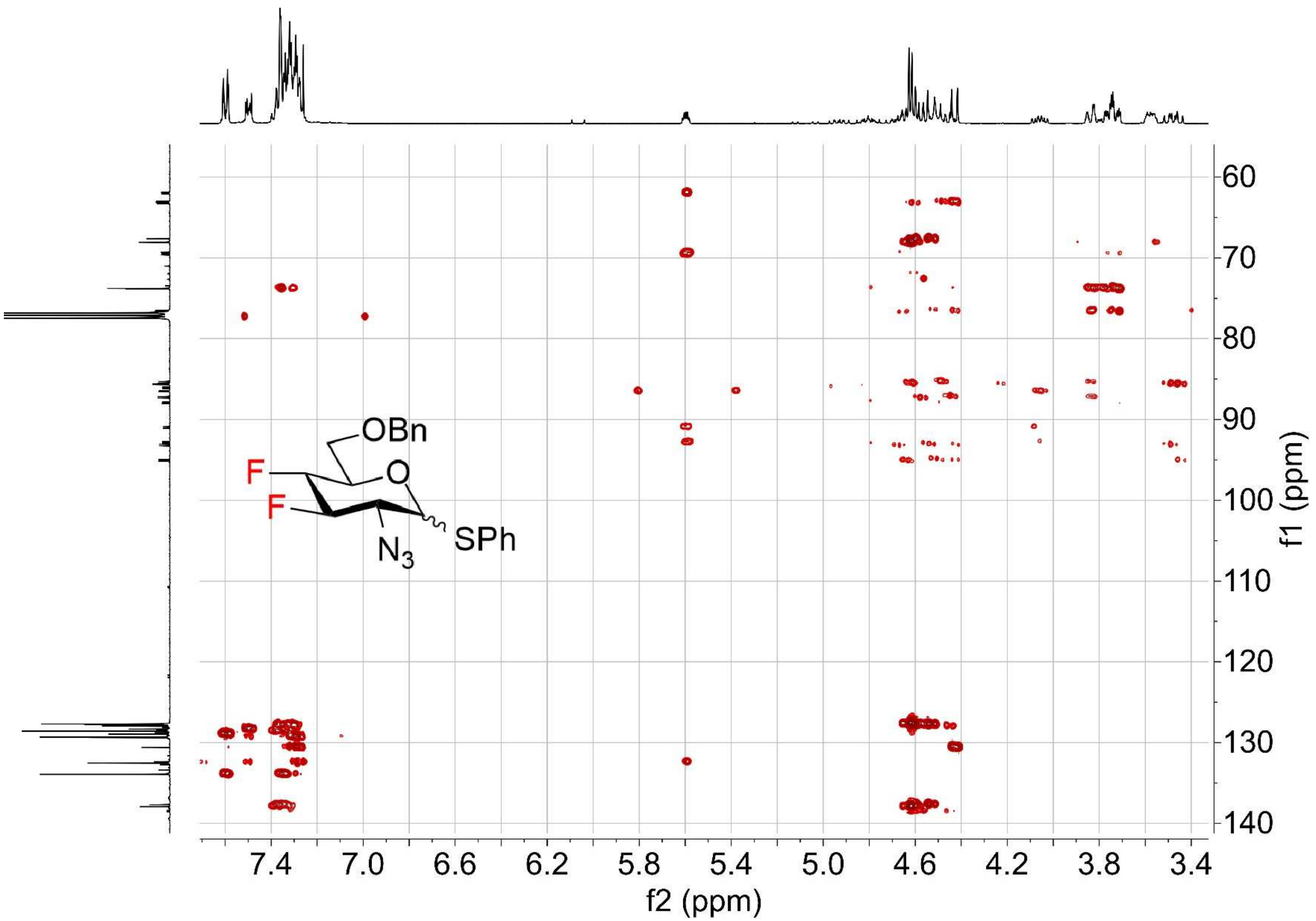

NMR COMPOUND β-25

^{1}H NMR (400 MHz, CDCl$_3$) β-25

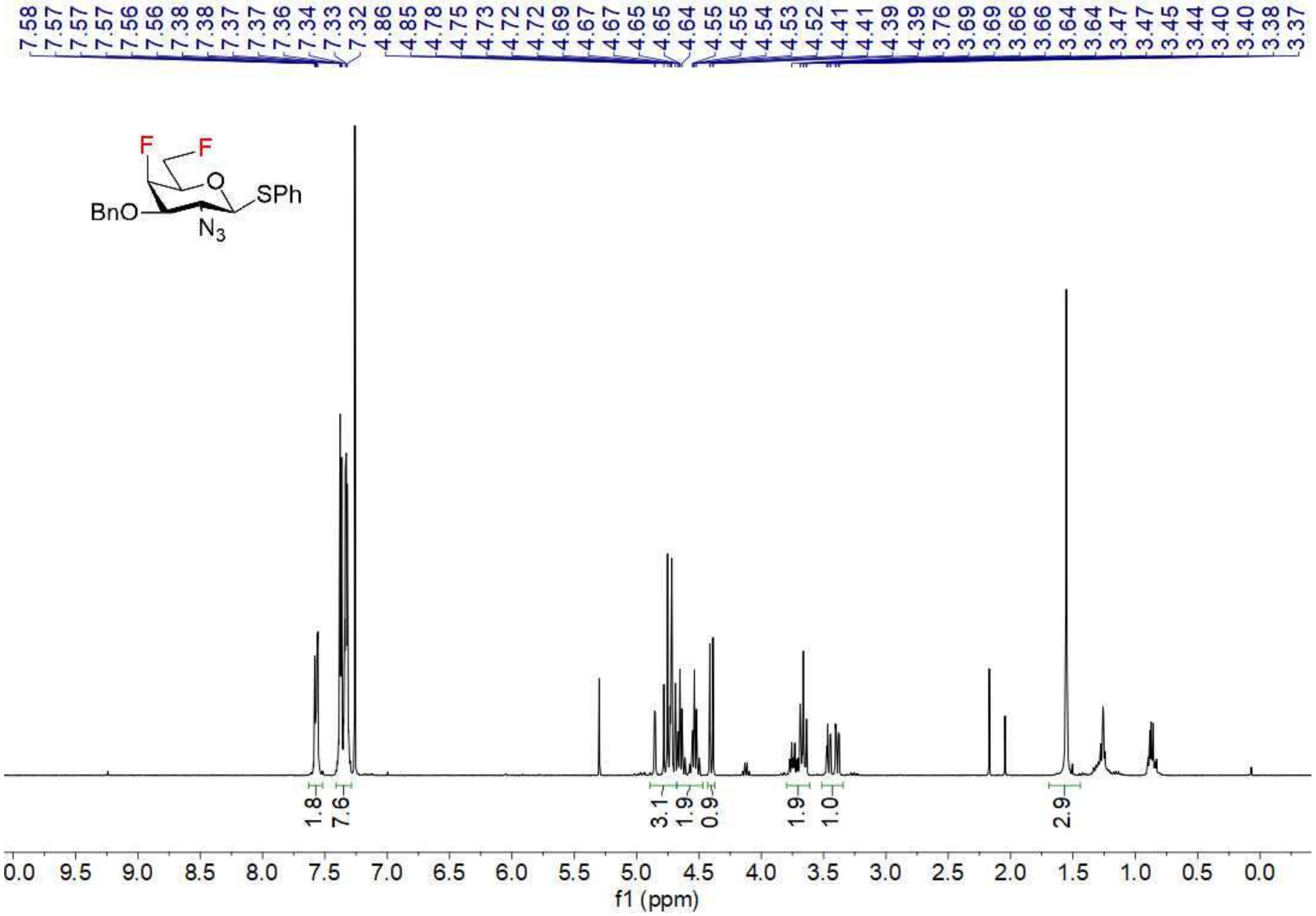

^{13}C NMR (100 MHz, CDCl$_3$) β-**25**

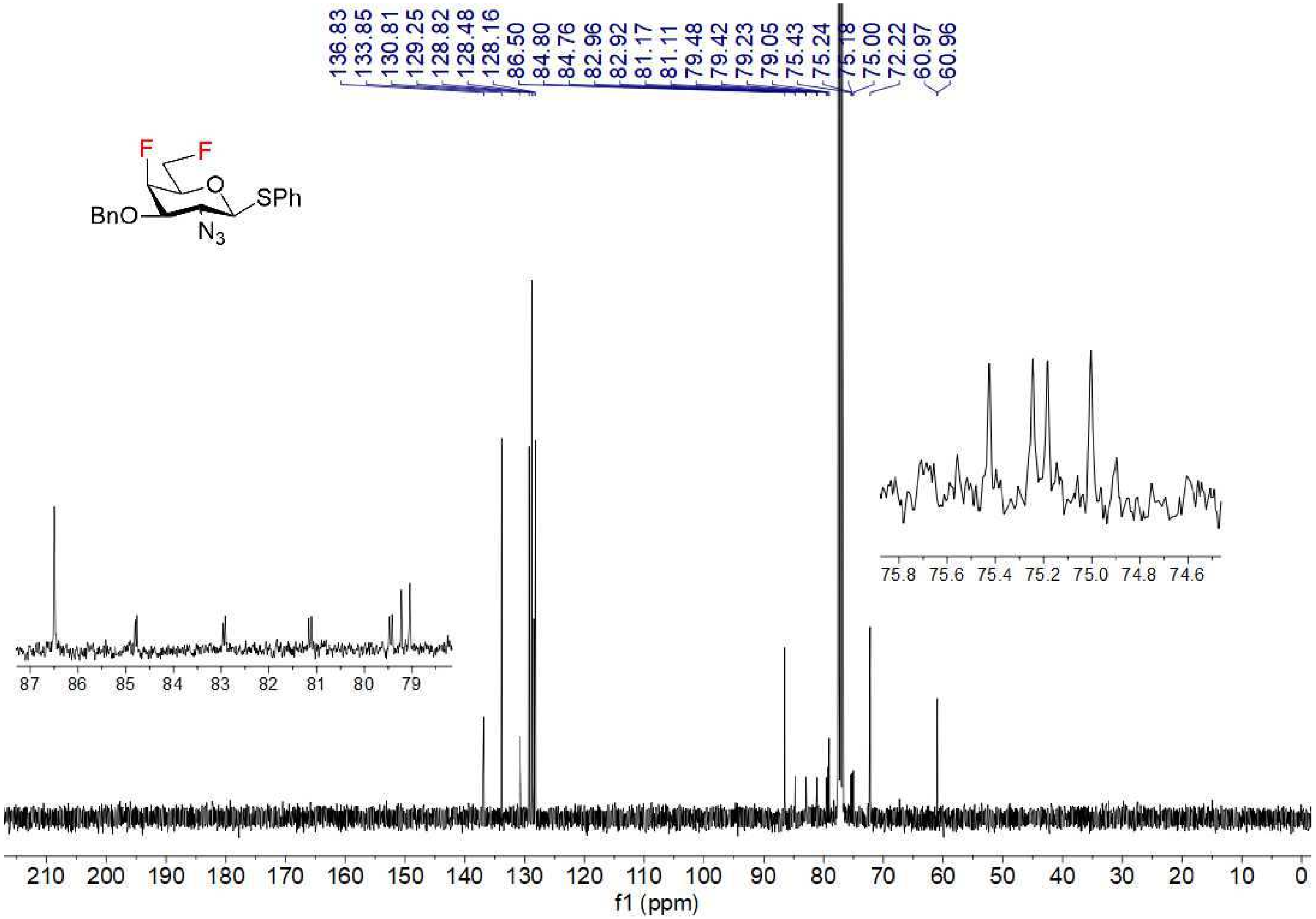

^{19}F NMR (376 MHz, CDCl$_3$) β-25

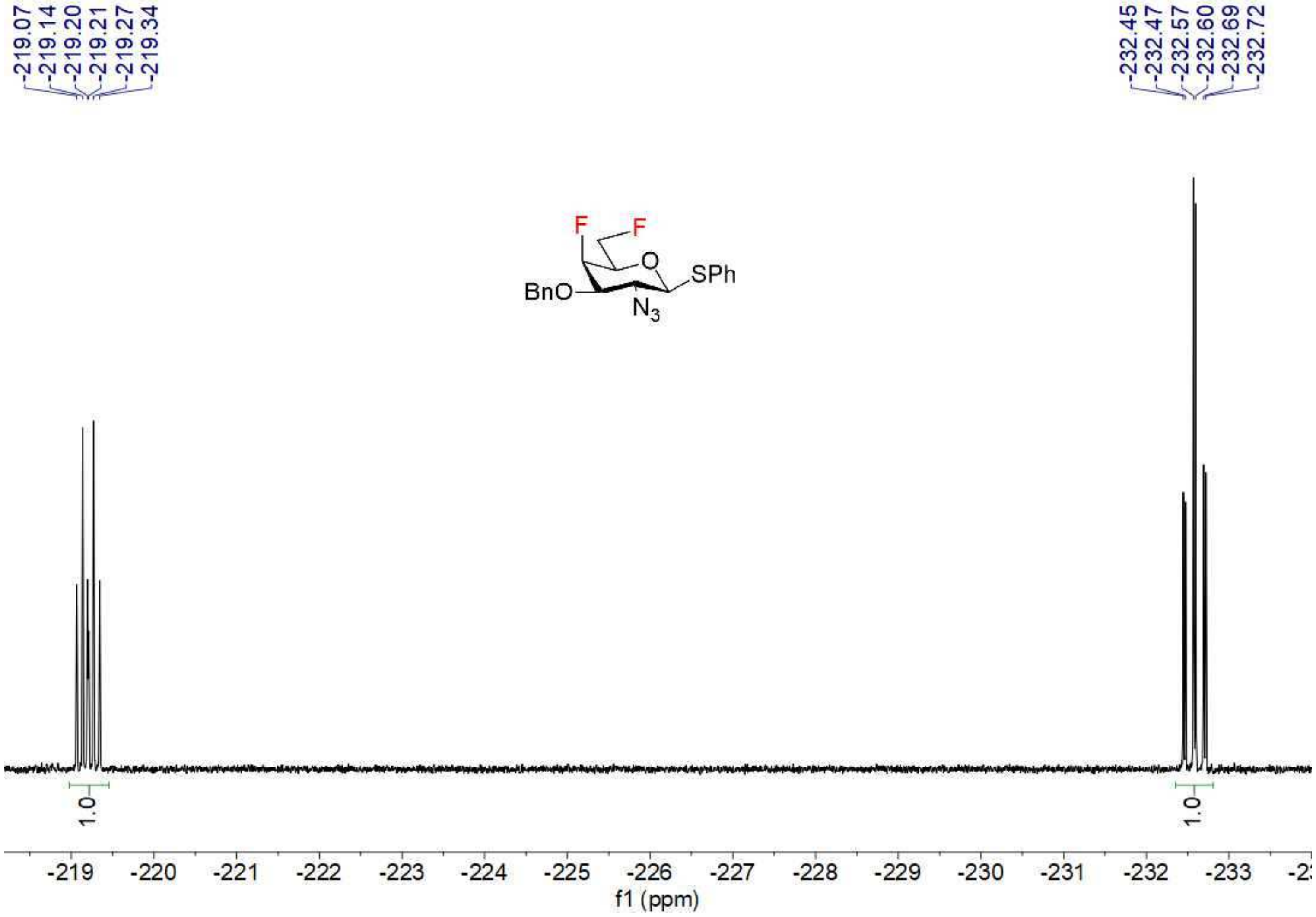

^{1}H-^{1}H COSY β-25

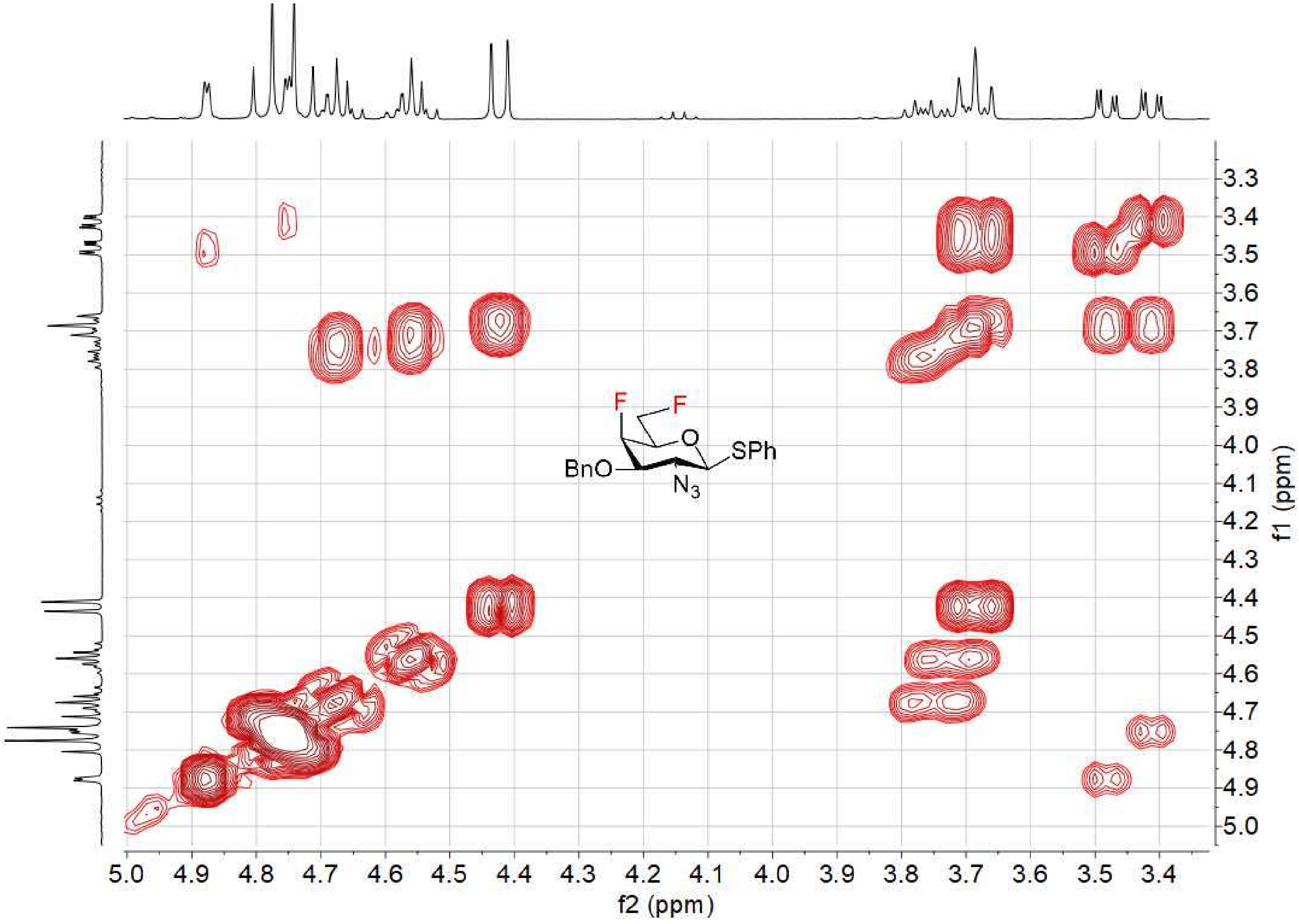

^{1}H-^{13}C HSQC β-25

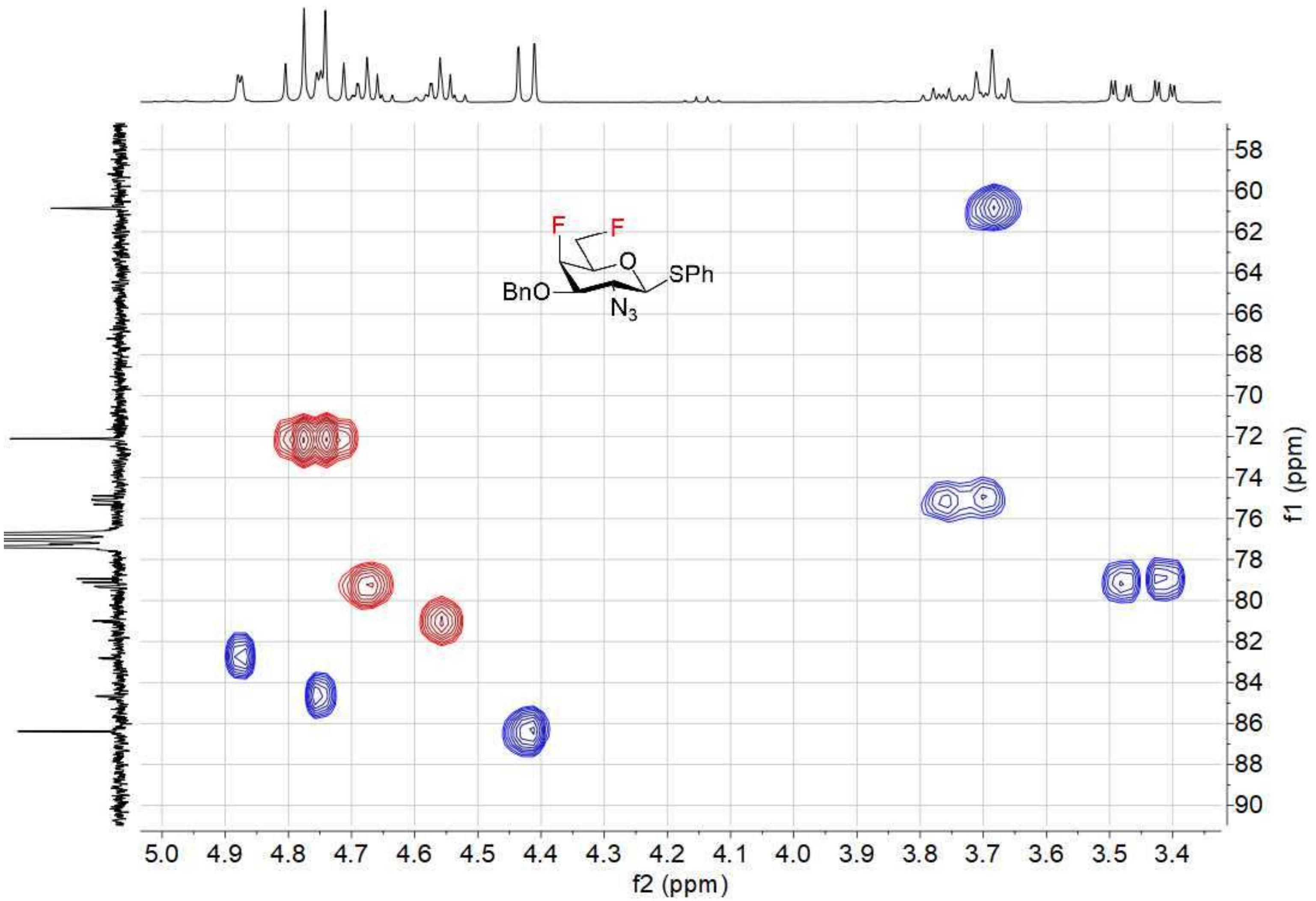

NMR COMPOUND 32

^{1}H NMR (400 MHz, $CDCl_3$) 32

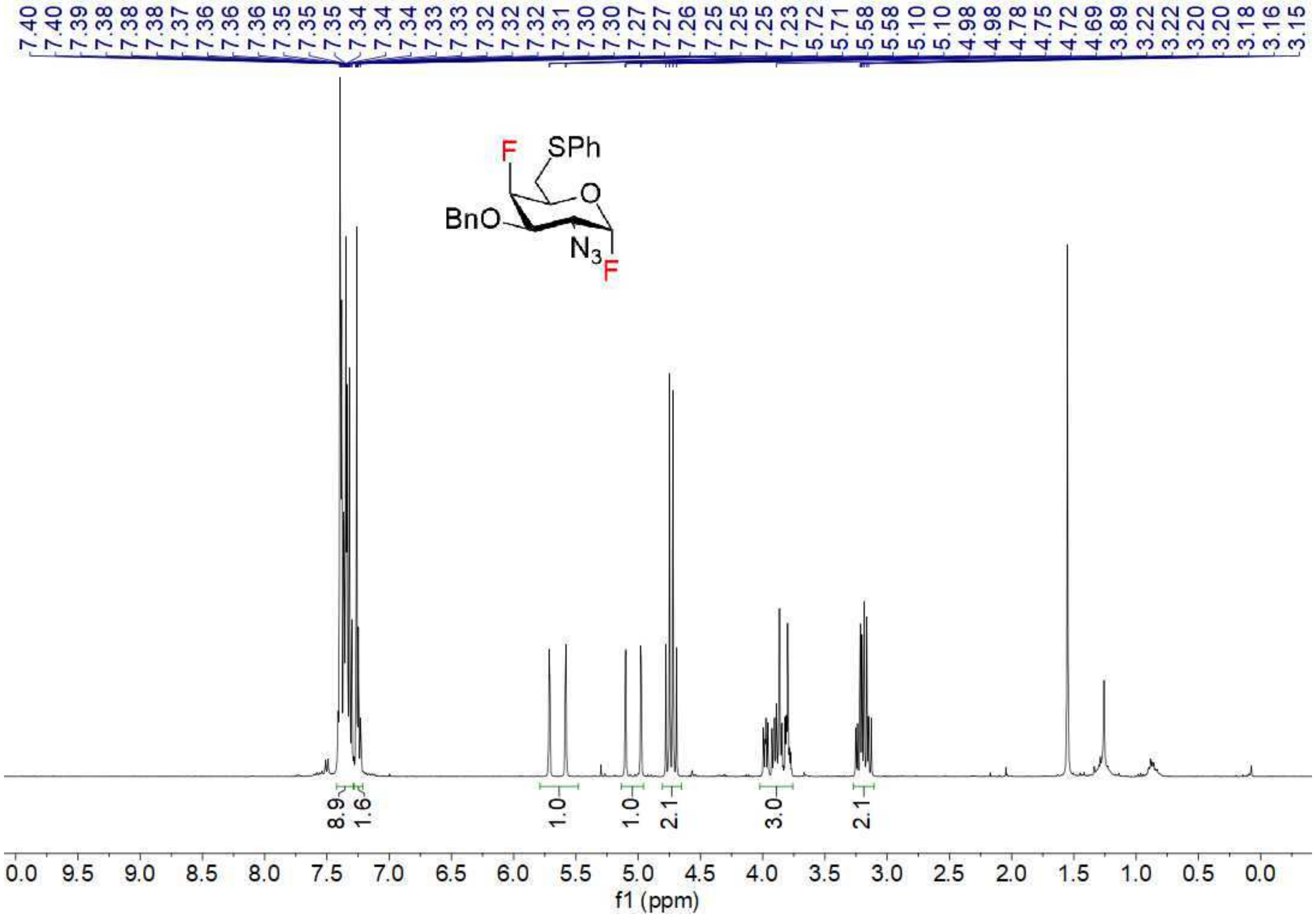

^{13}C NMR (100 MHz, CDCl$_3$) 32

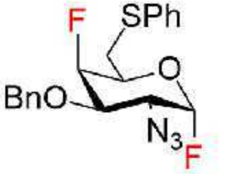

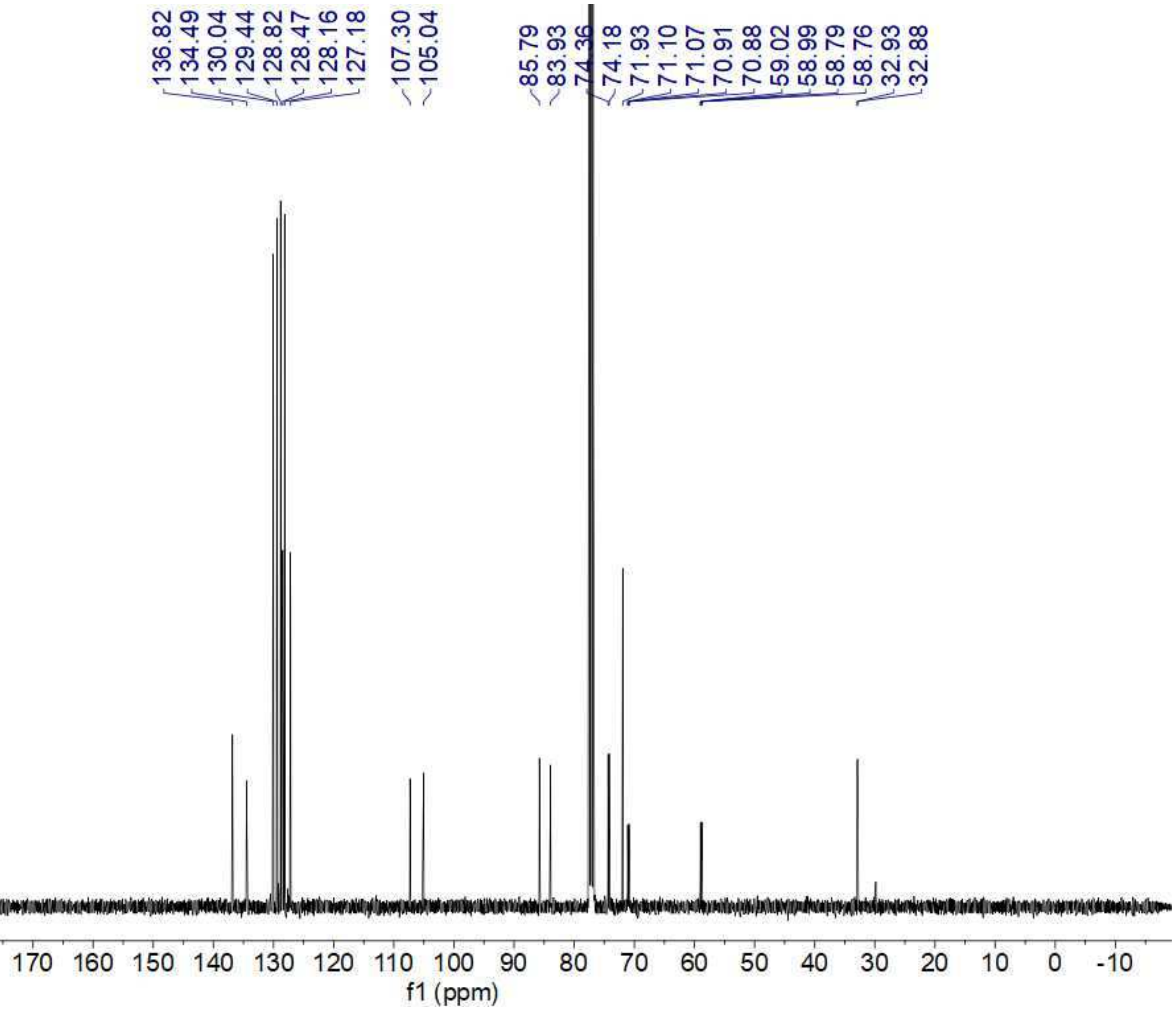

^{19}F NMR (376 MHz, $CDCl_3$) 32

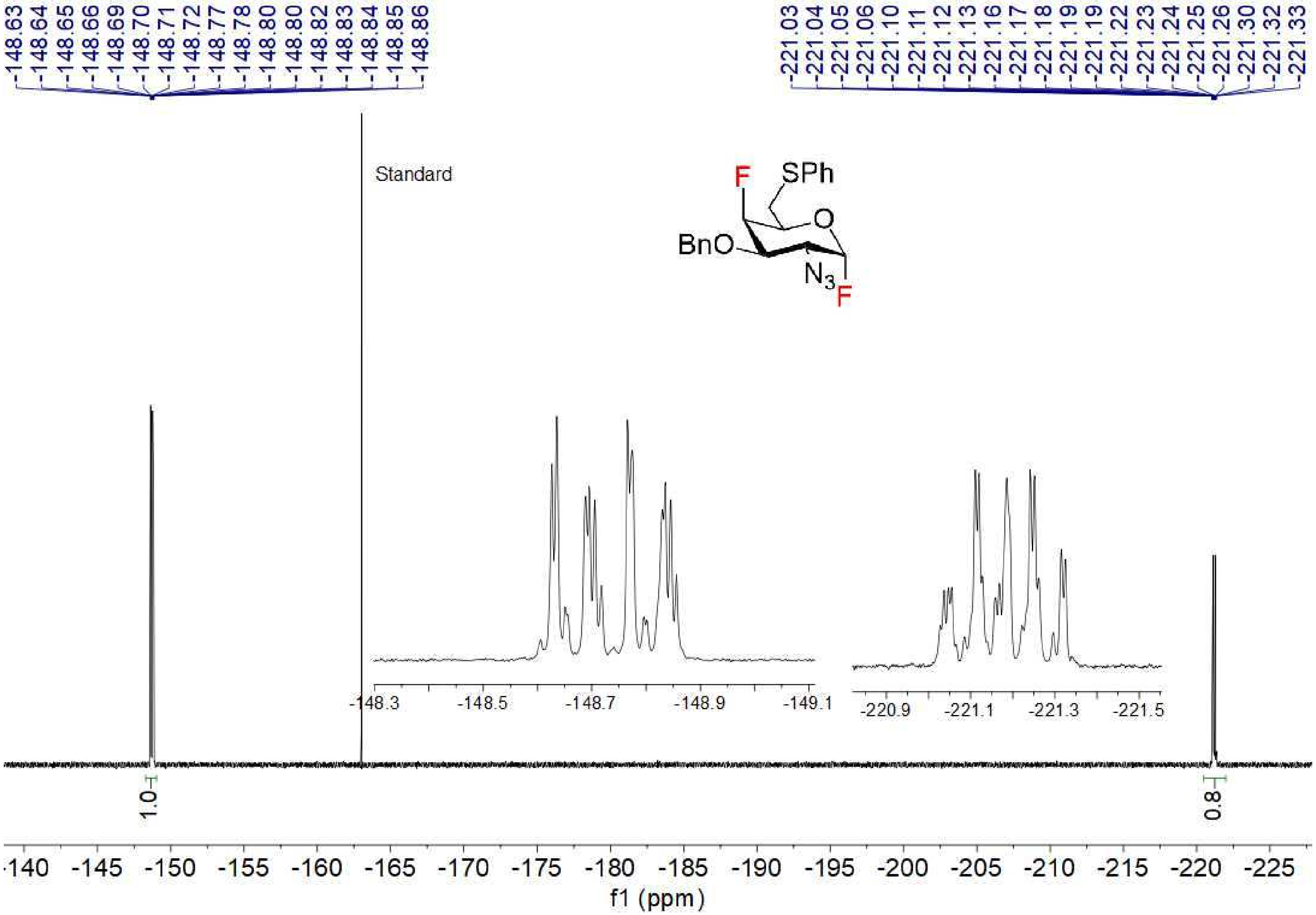

^{1}H-^{1}H COSY 32

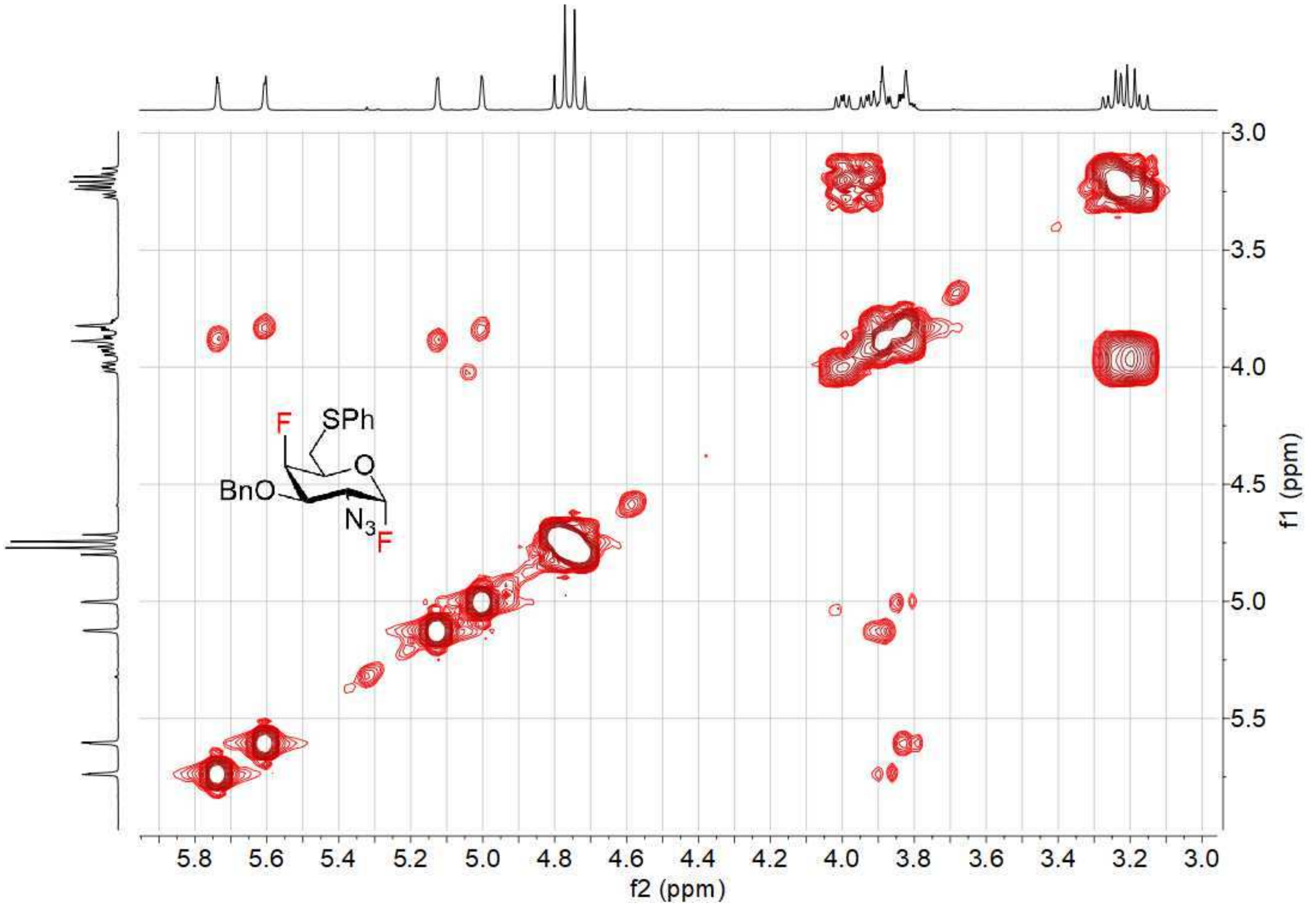

F
SPh
O
BnO
N3
F
5.8
5.6
5.4
5.2
5.0
4.8
4.6
4.4
4.2
4.0
3.8
3.6
3.4
3.2
3.0
f2 (ppm)
3.0
3.5
4.0
4.5
5.0
5.5
f1 (ppm)

^{1}H-^{13}C HSQC 32

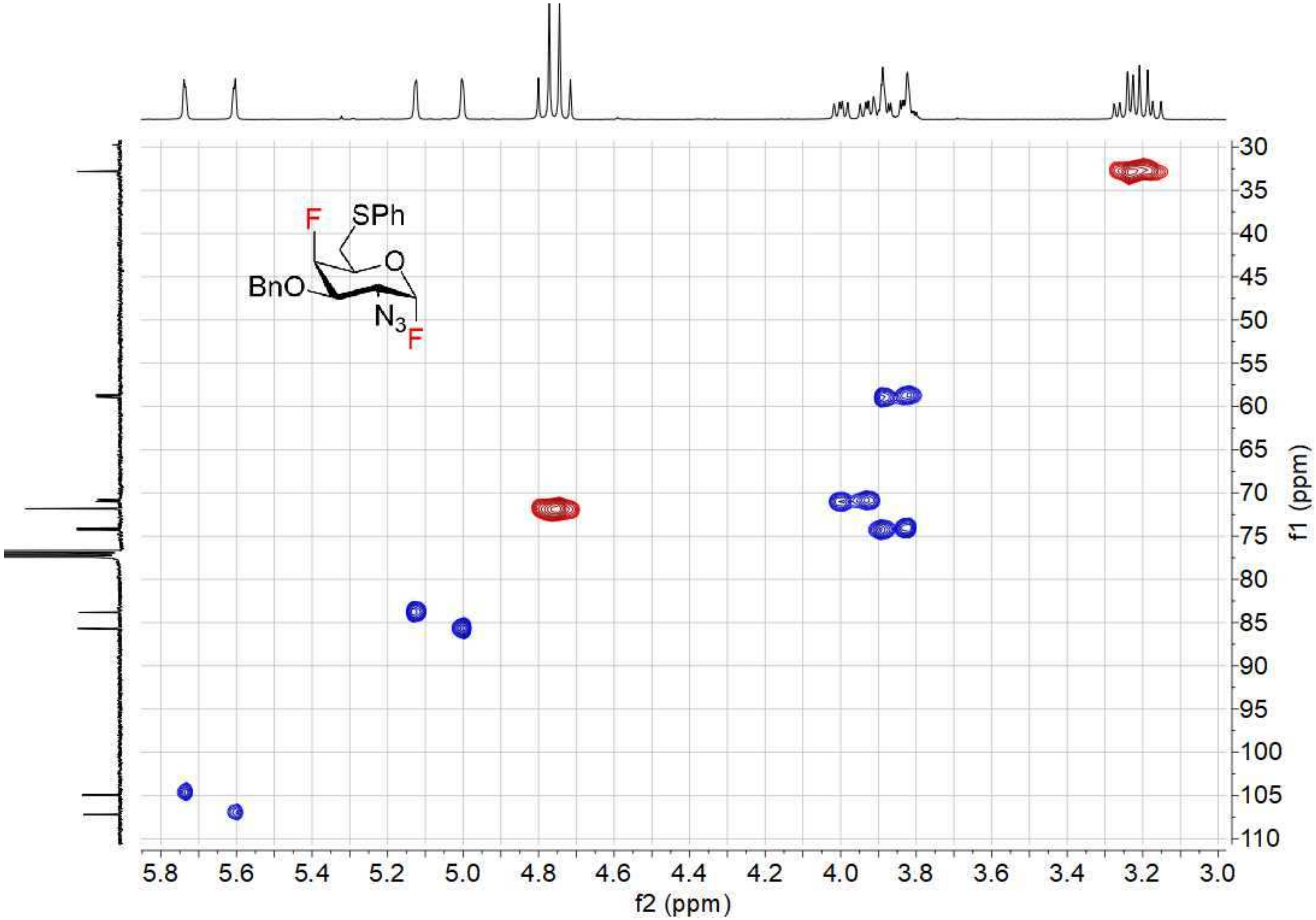

NMR COMPOUNDS β-22 and 33

^{1}H NMR (400 MHz, $CDCl_3$) β-22 (ca. 9% of 33)

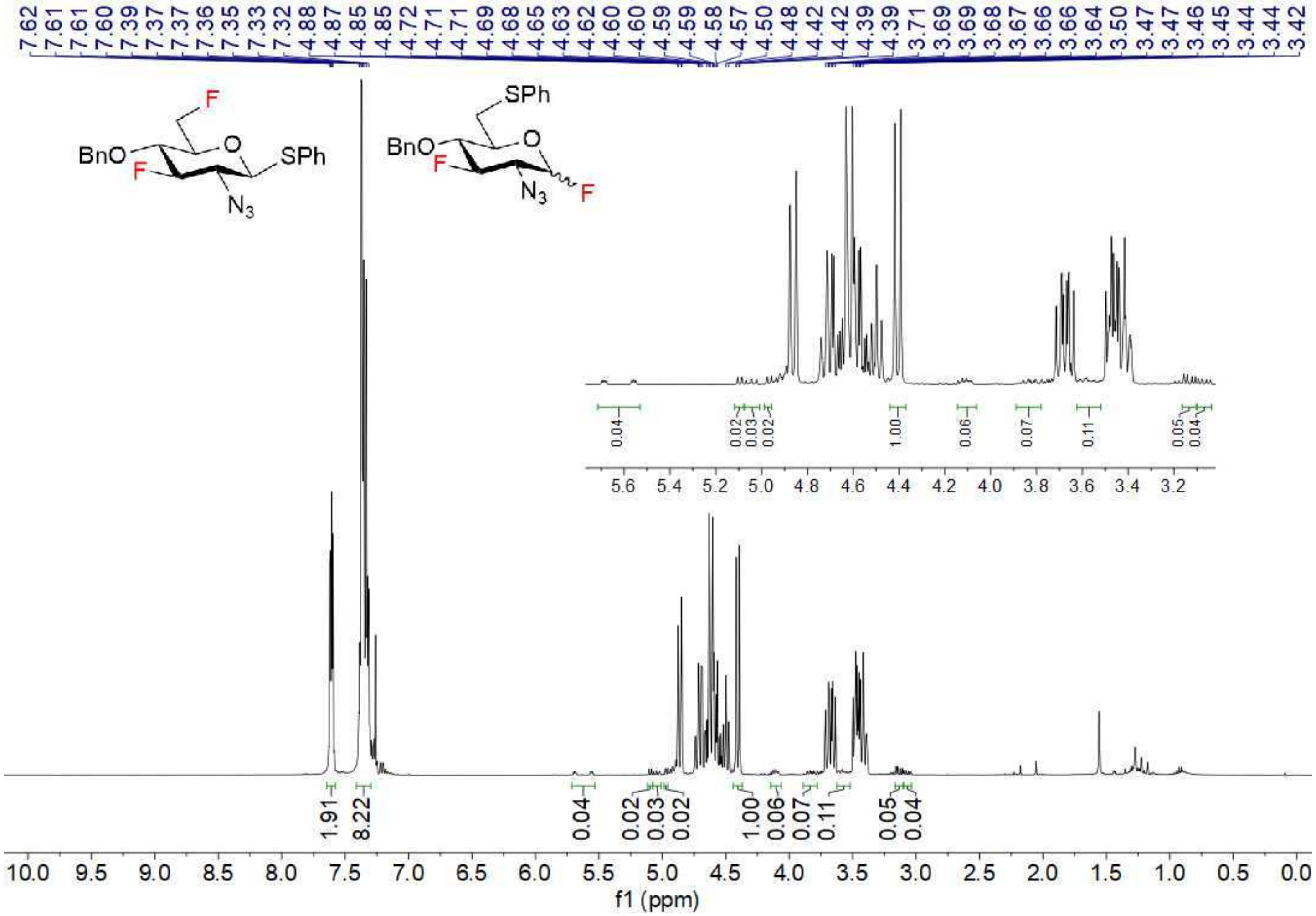

^{13}C NMR (100 MHz, CDCl$_3$) β-22 (ca. 9% of 33)

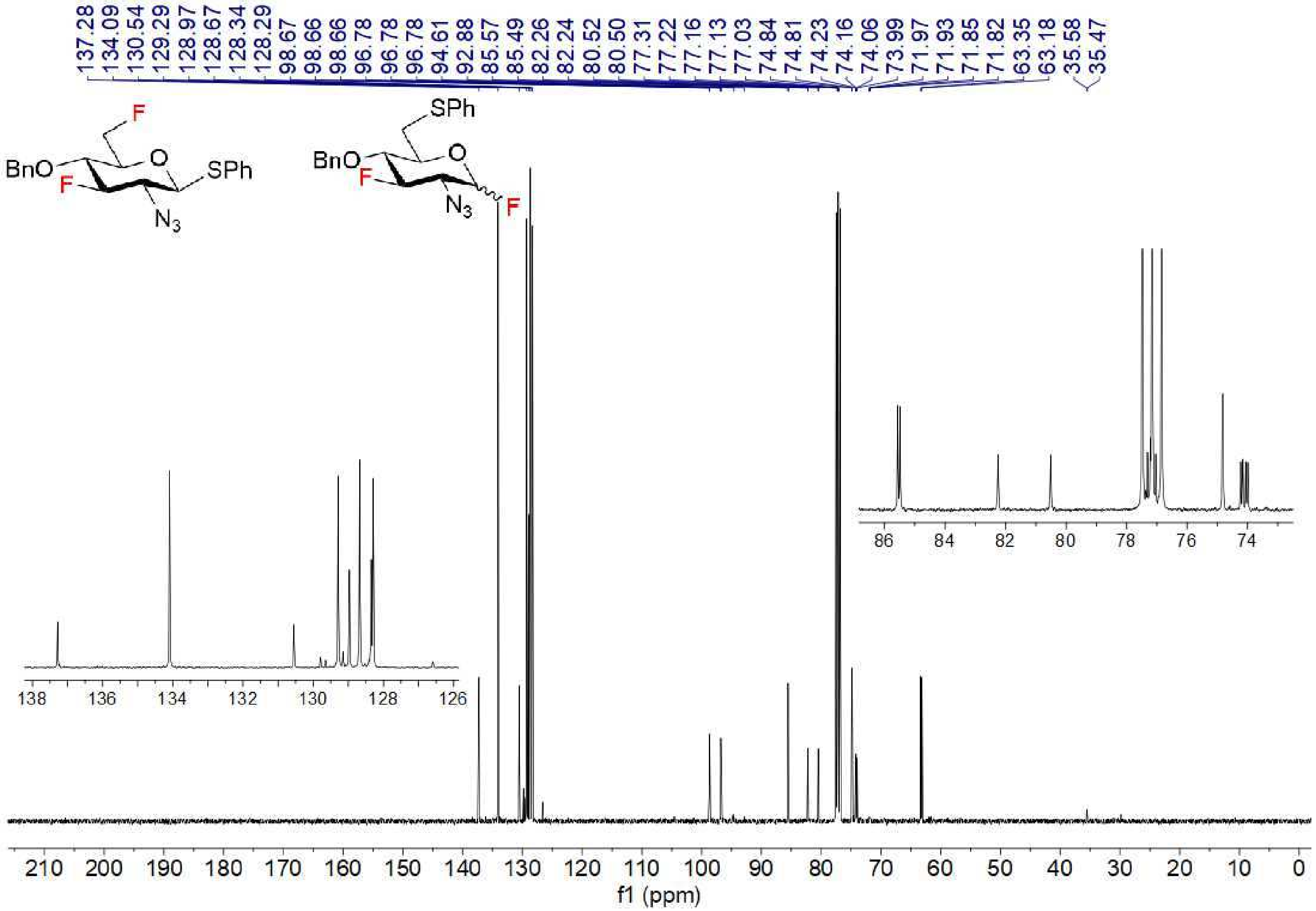

^{19}F NMR (376 MHz, $CDCl_3$) β-22 (ca. 5% of α-33 and 4% of β-33)

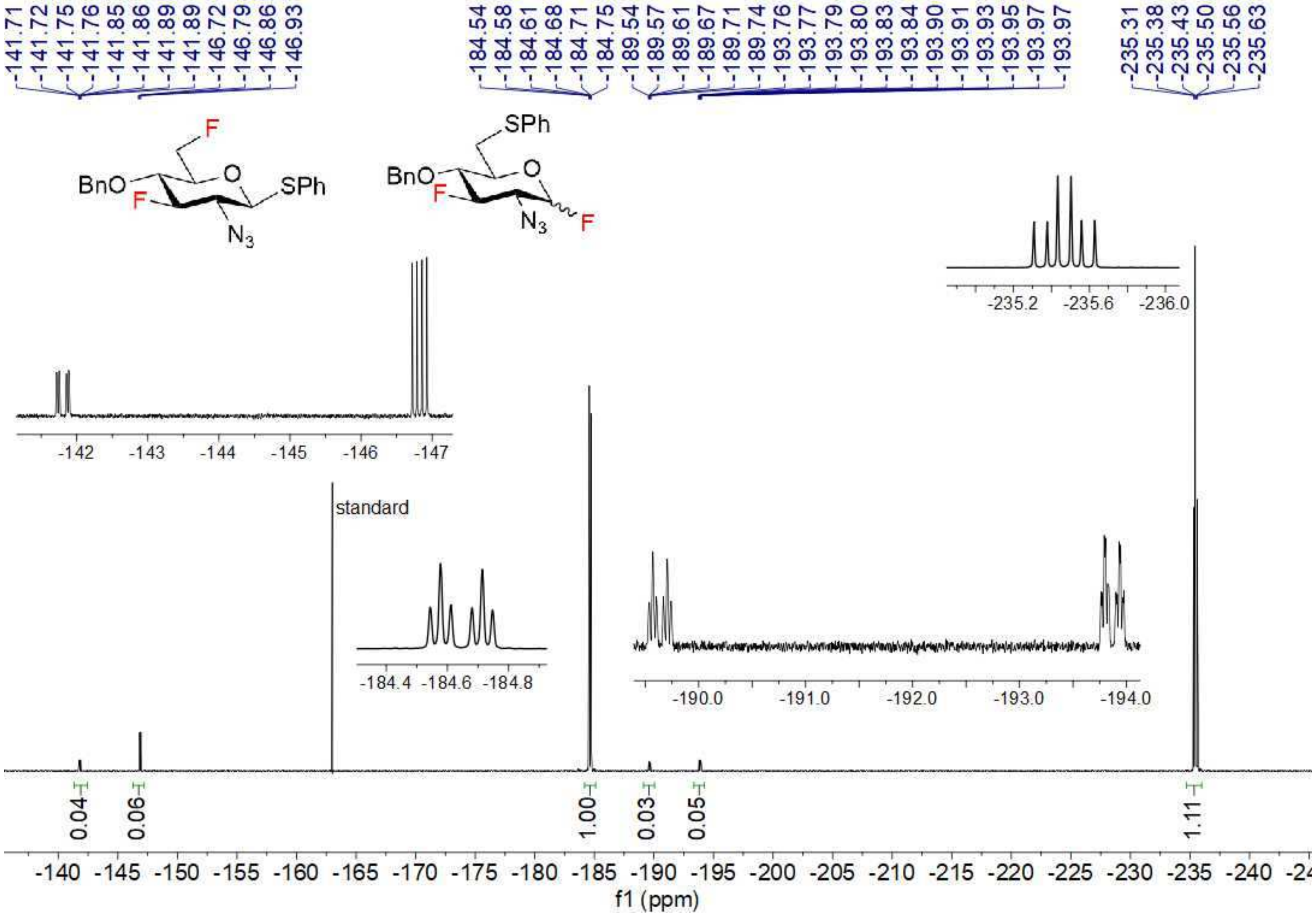

^{1}H-^{1}H COSY β-22 (ca. 9% of 33)

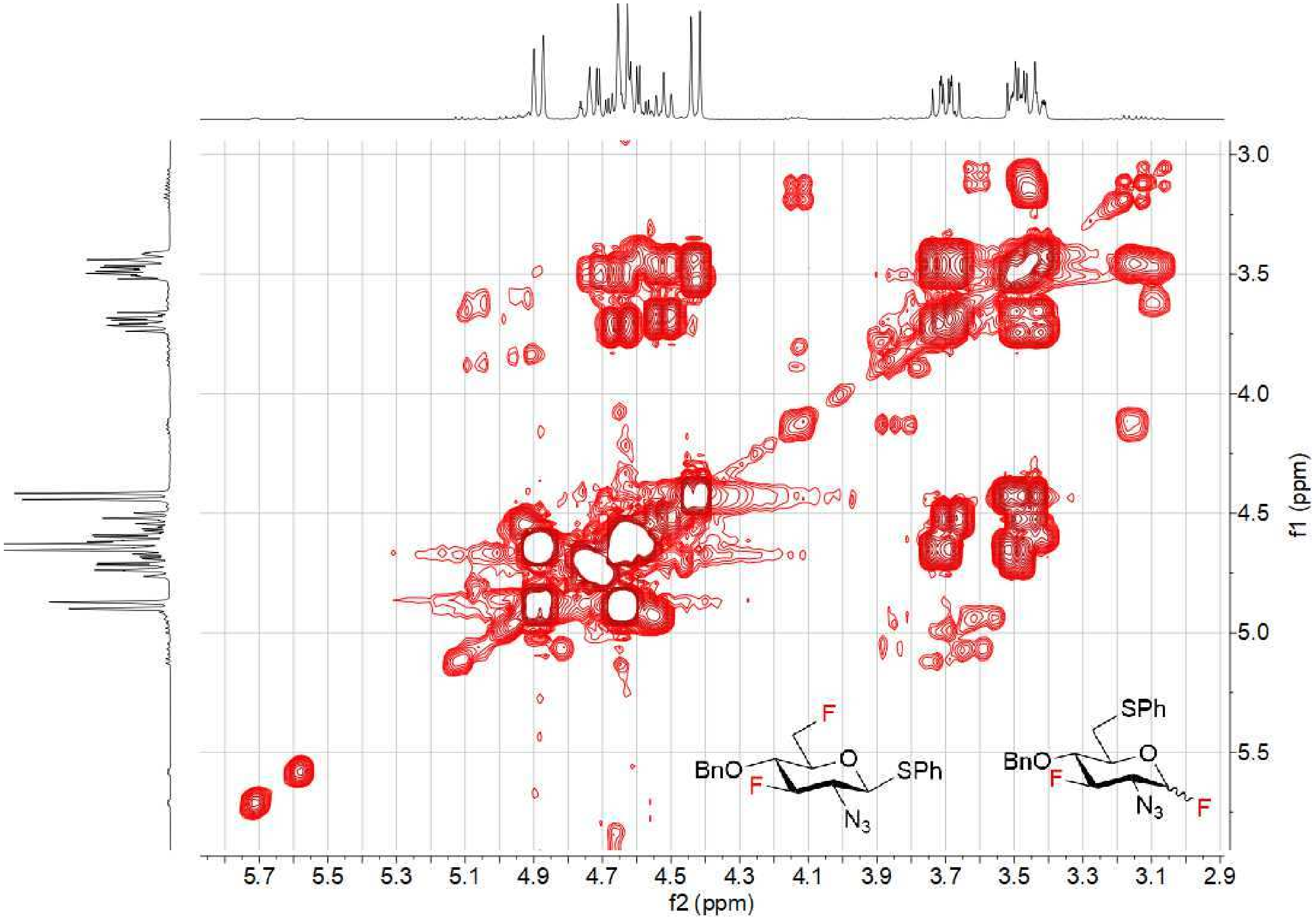

^{1}H-^{13}C HMBC β-22 (ca. 9% of 33)

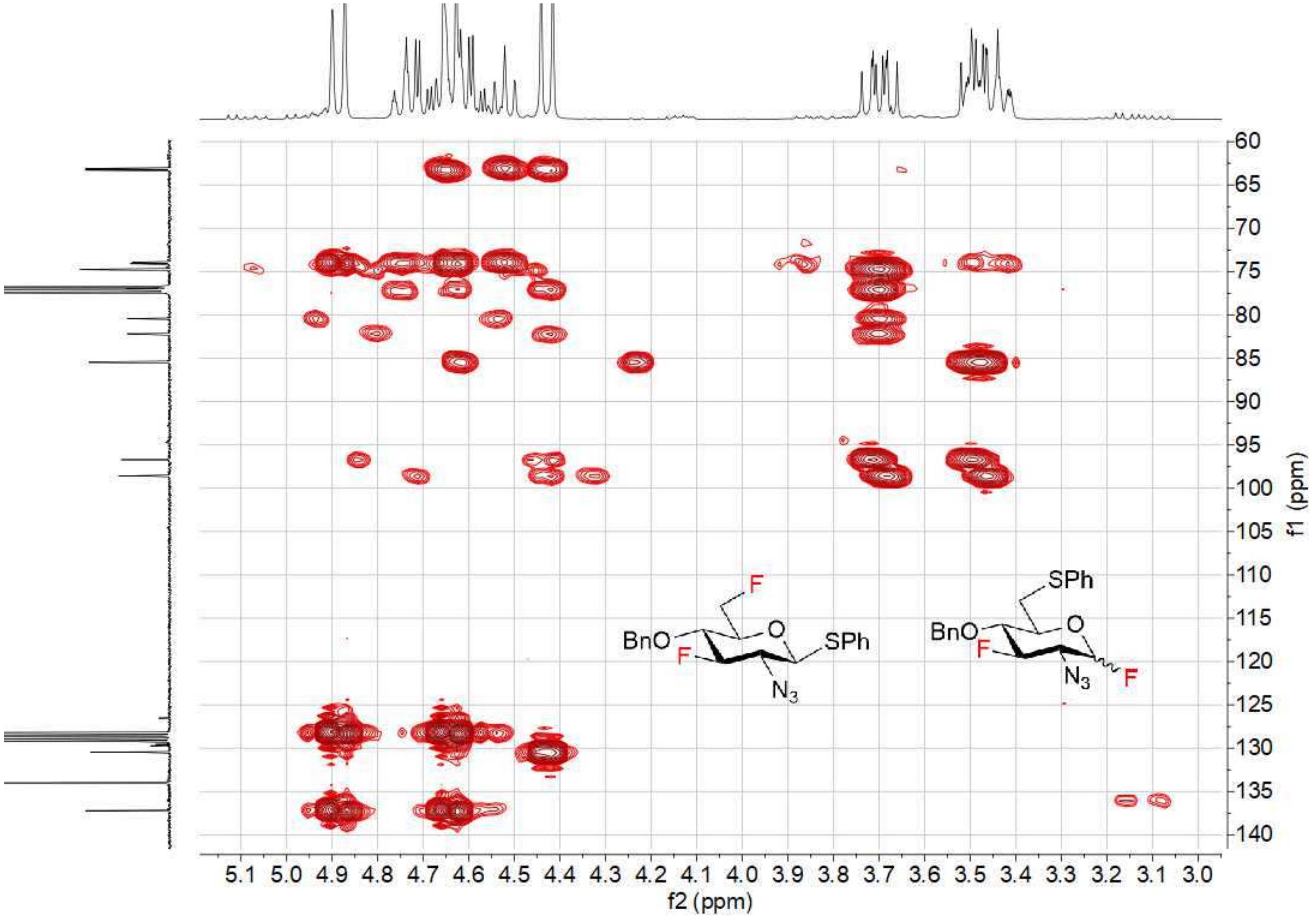

^{1}H-^{13}C HSQC β-22 (ca. 9% of 33)

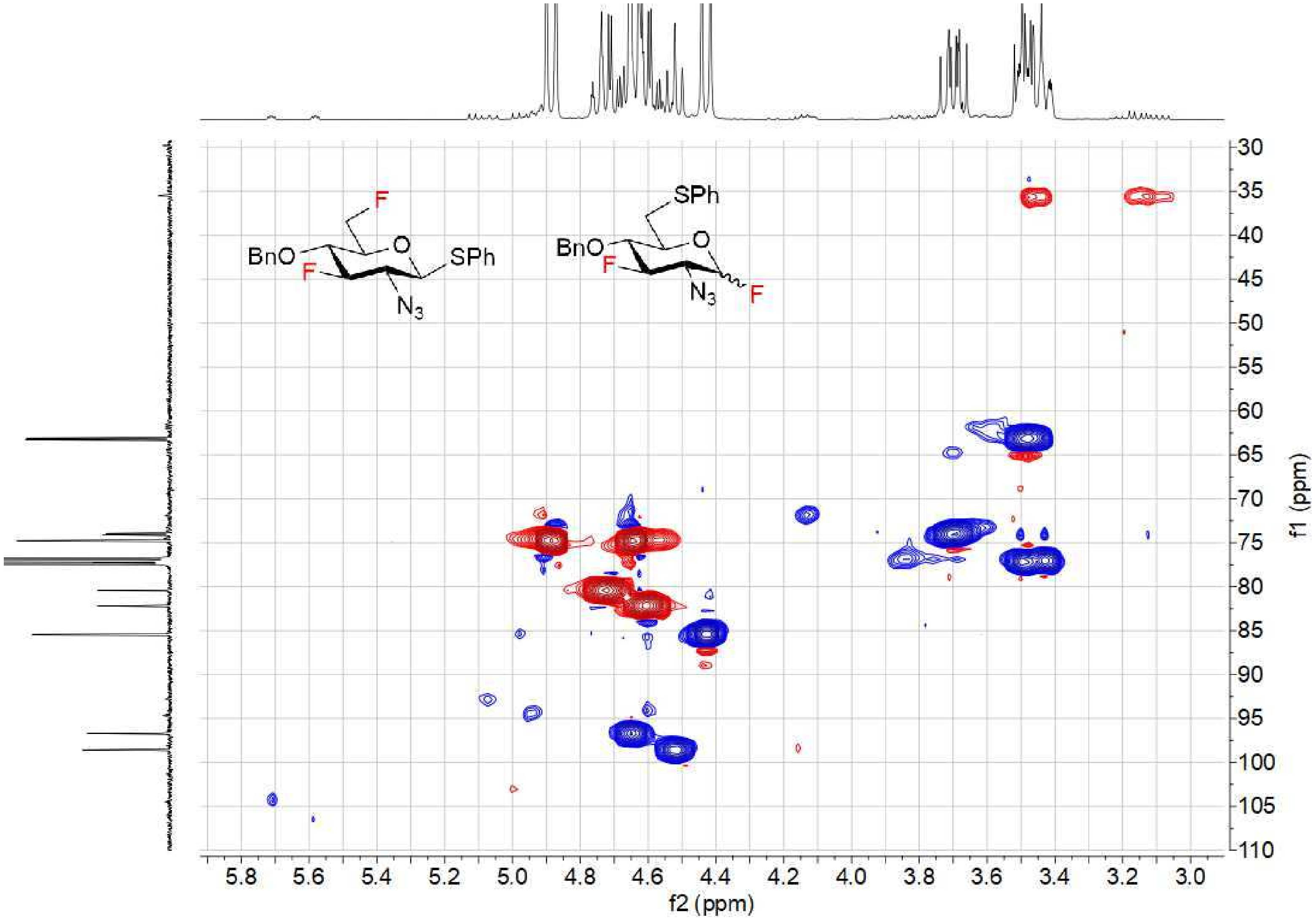

NMR COMPOUND α-34

^{1}H NMR (400 MHz, $CDCl_3$) α-34

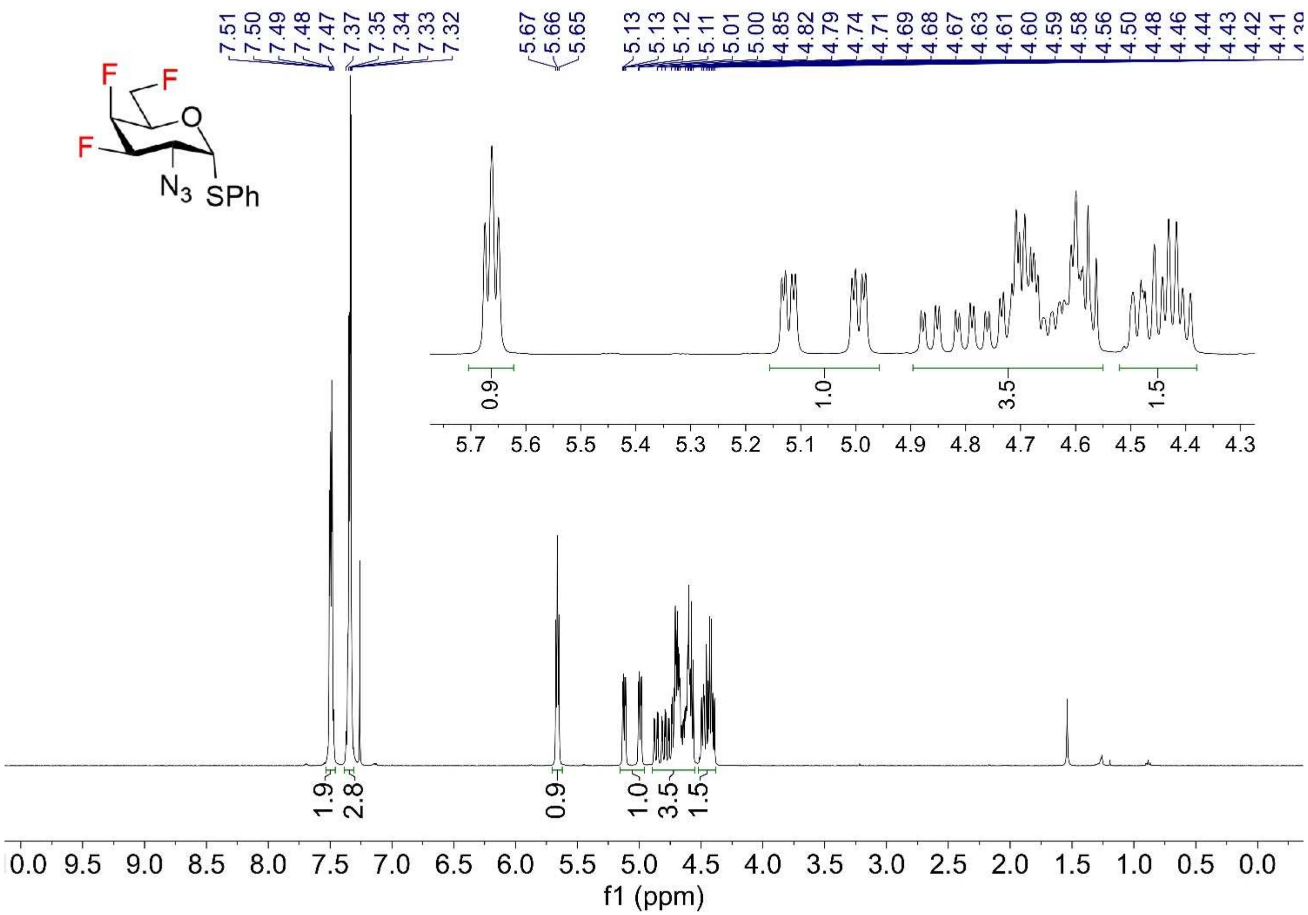

^{13}C NMR (100 MHz, CDCl$_3$) α-34

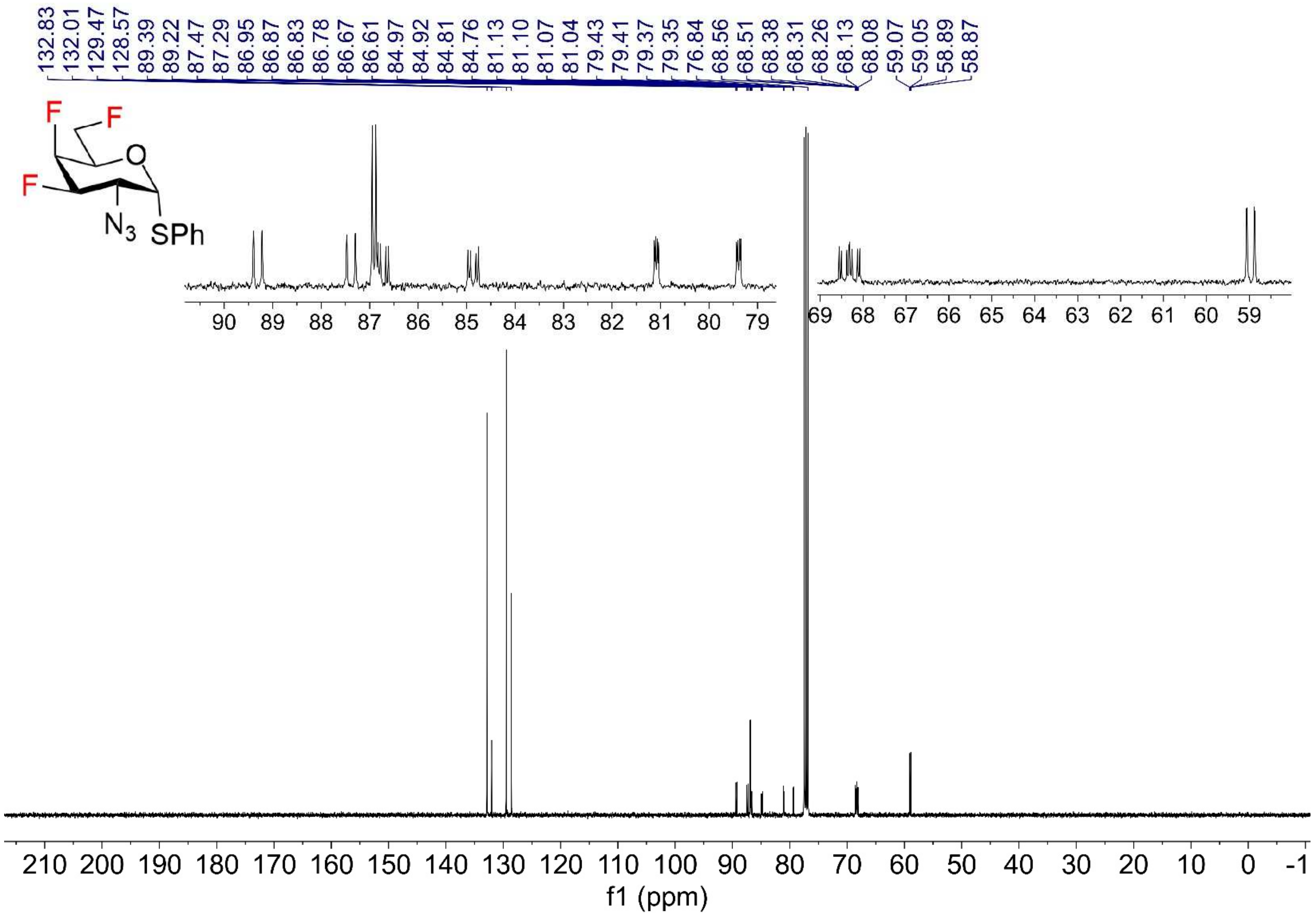

^{19}F NMR (376 MHz, CDCl$_3$) α-34

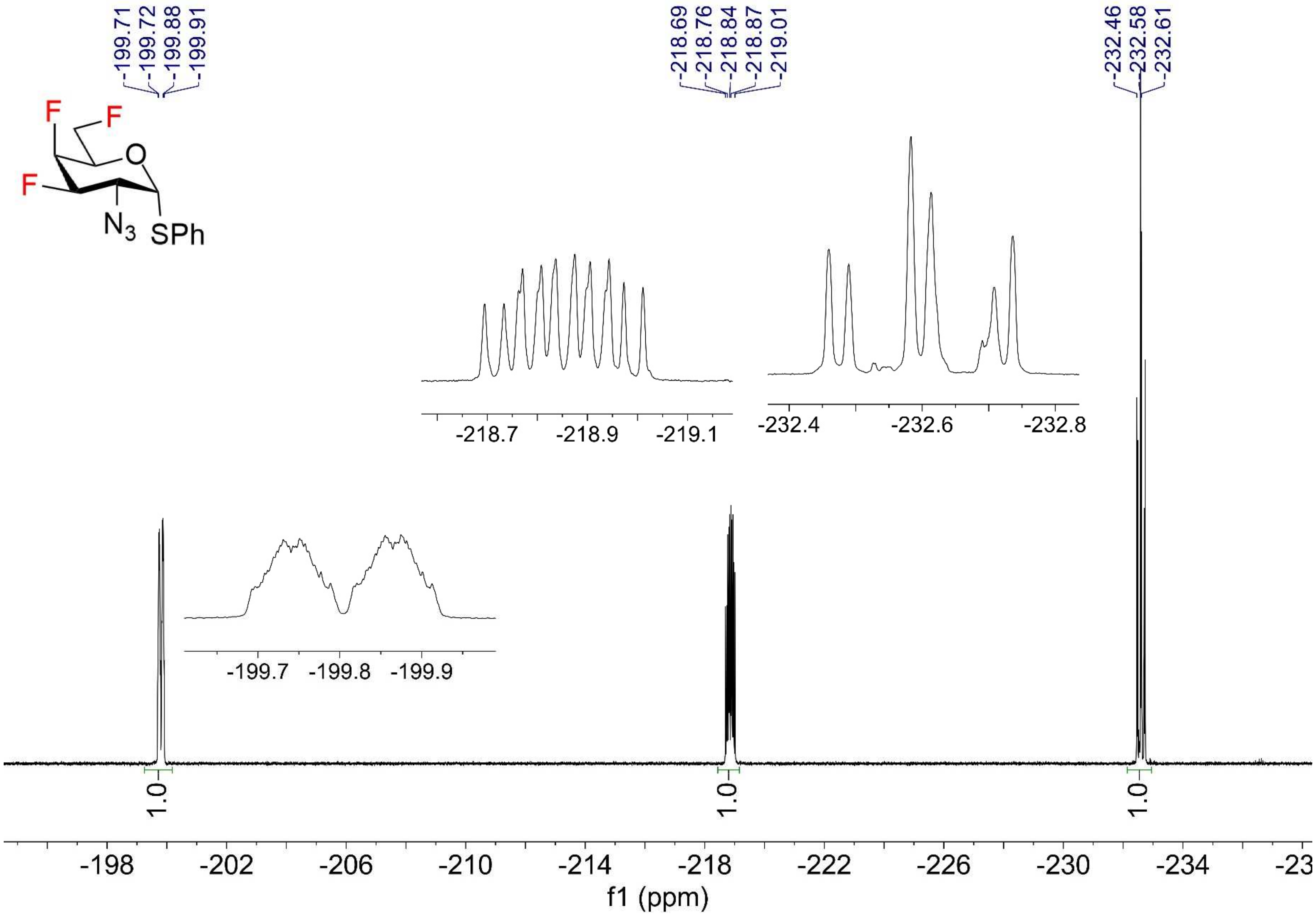

^{1}H-^{1}H COSY α-34

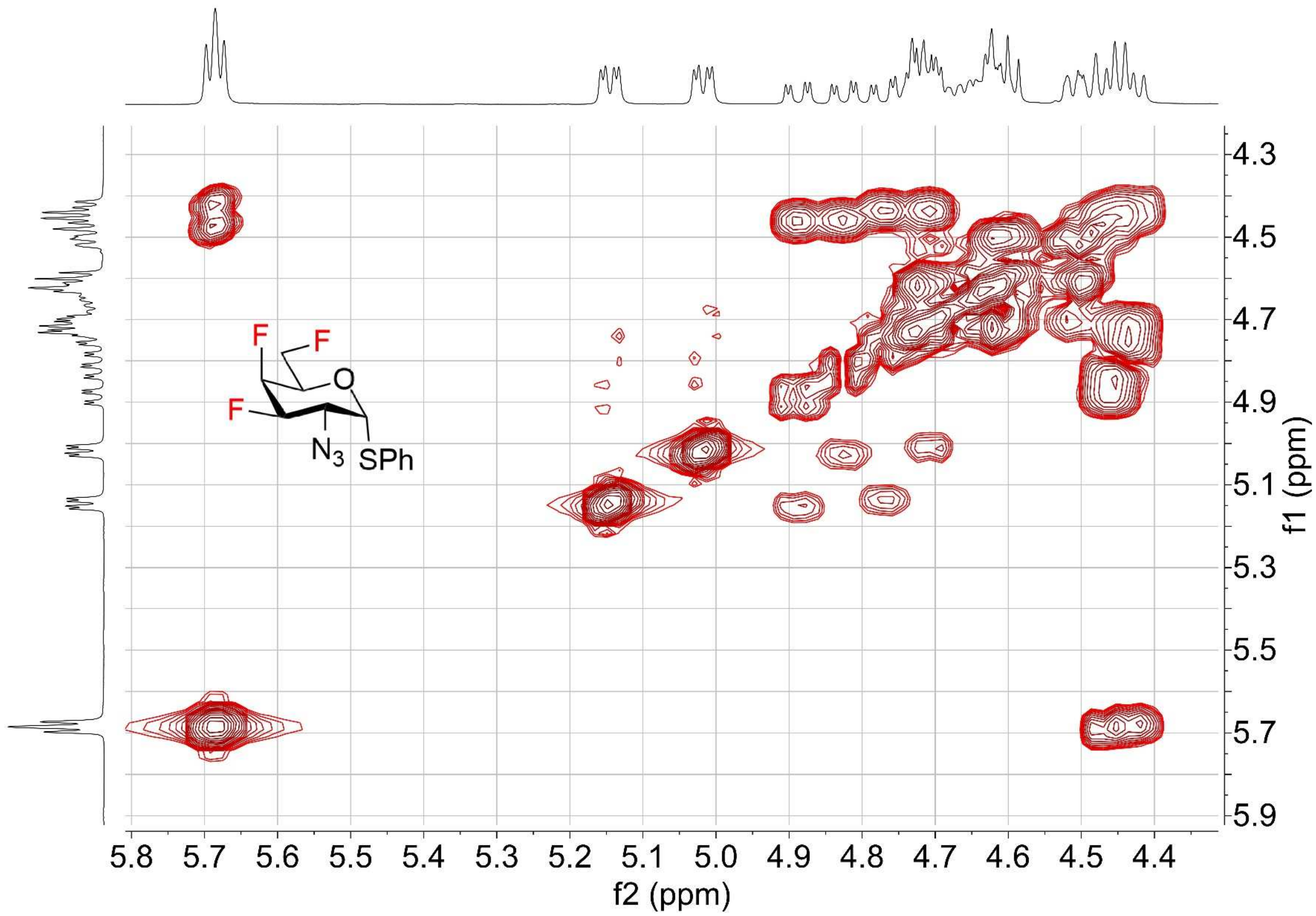

^{1}H-^{13}C HSQC α-34

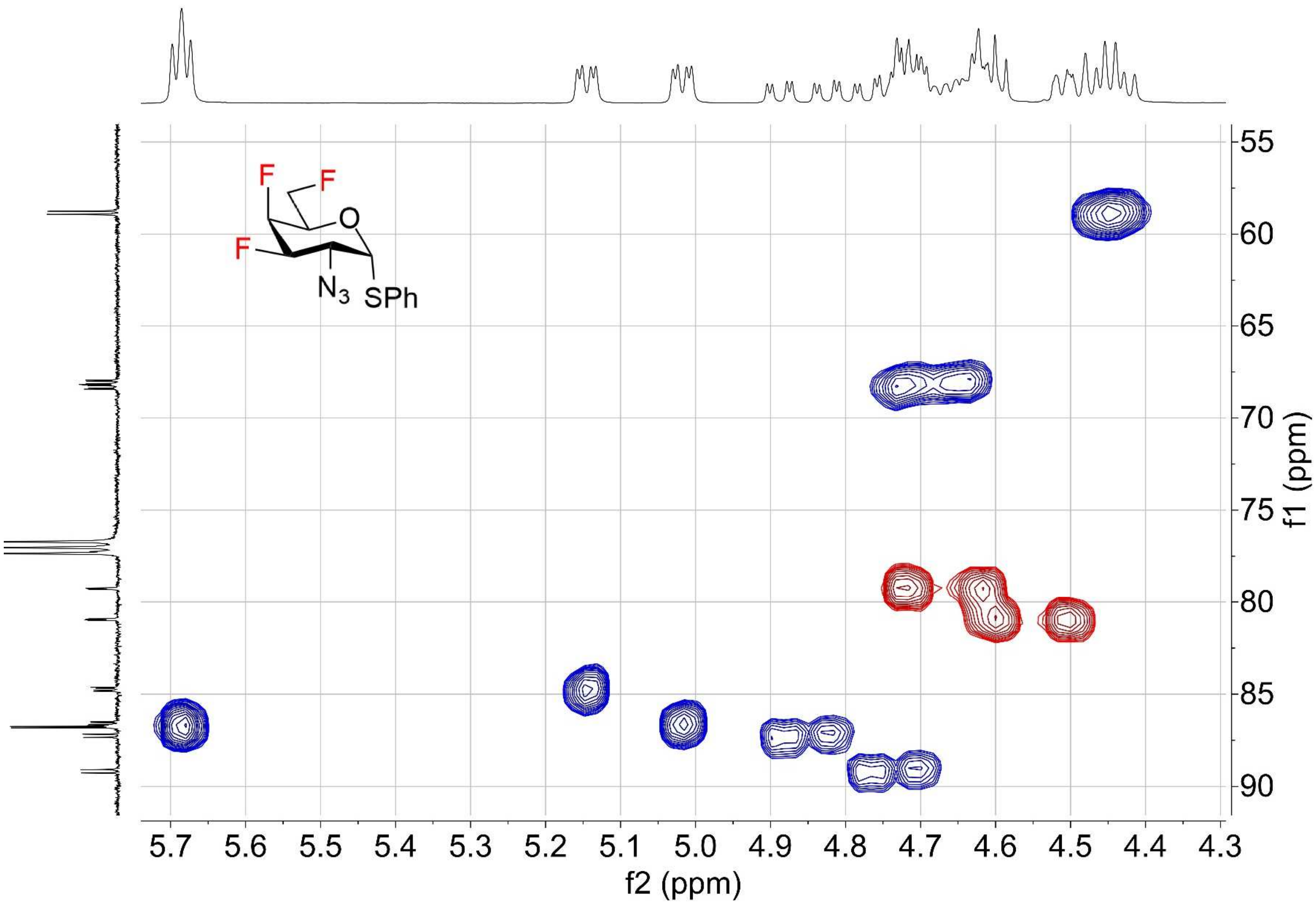

NMR COMPOUND β-34

^{1}H NMR (400 MHz, CDCl$_3$) β-34

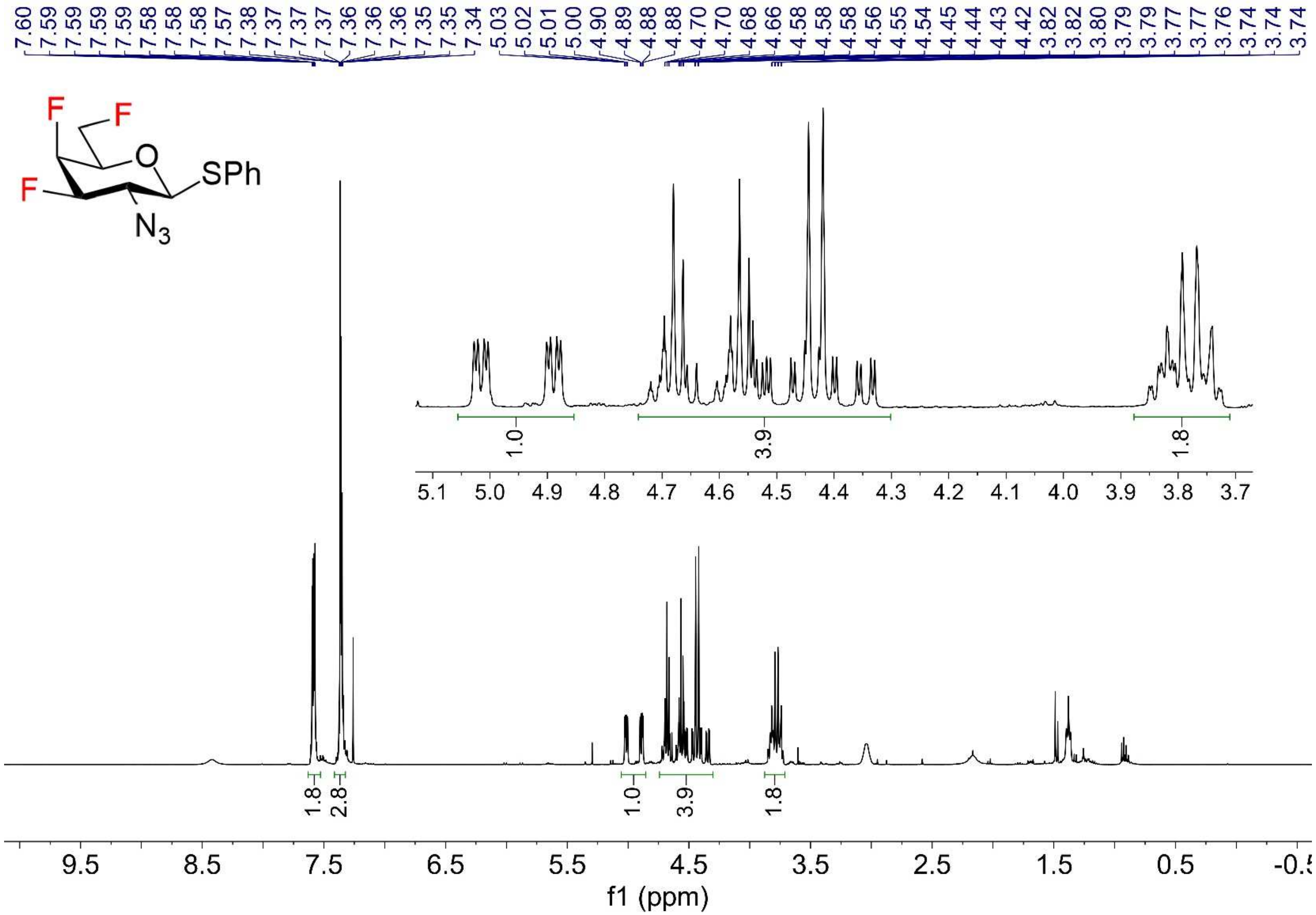

^{13}C NMR (100 MHz, CDCl$_3$) β-34

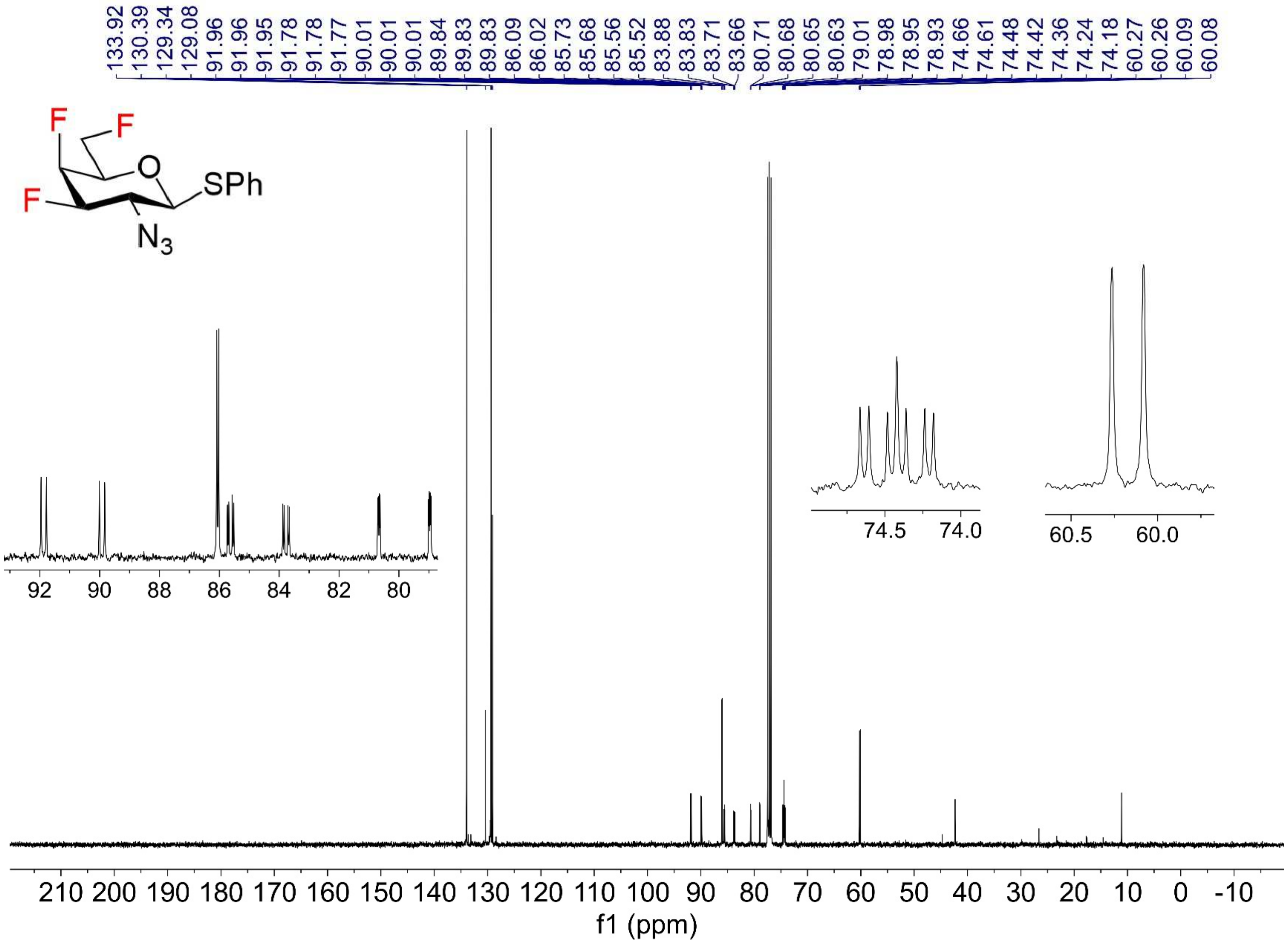

^{19}F NMR (376 MHz, CDCl$_3$) β-34

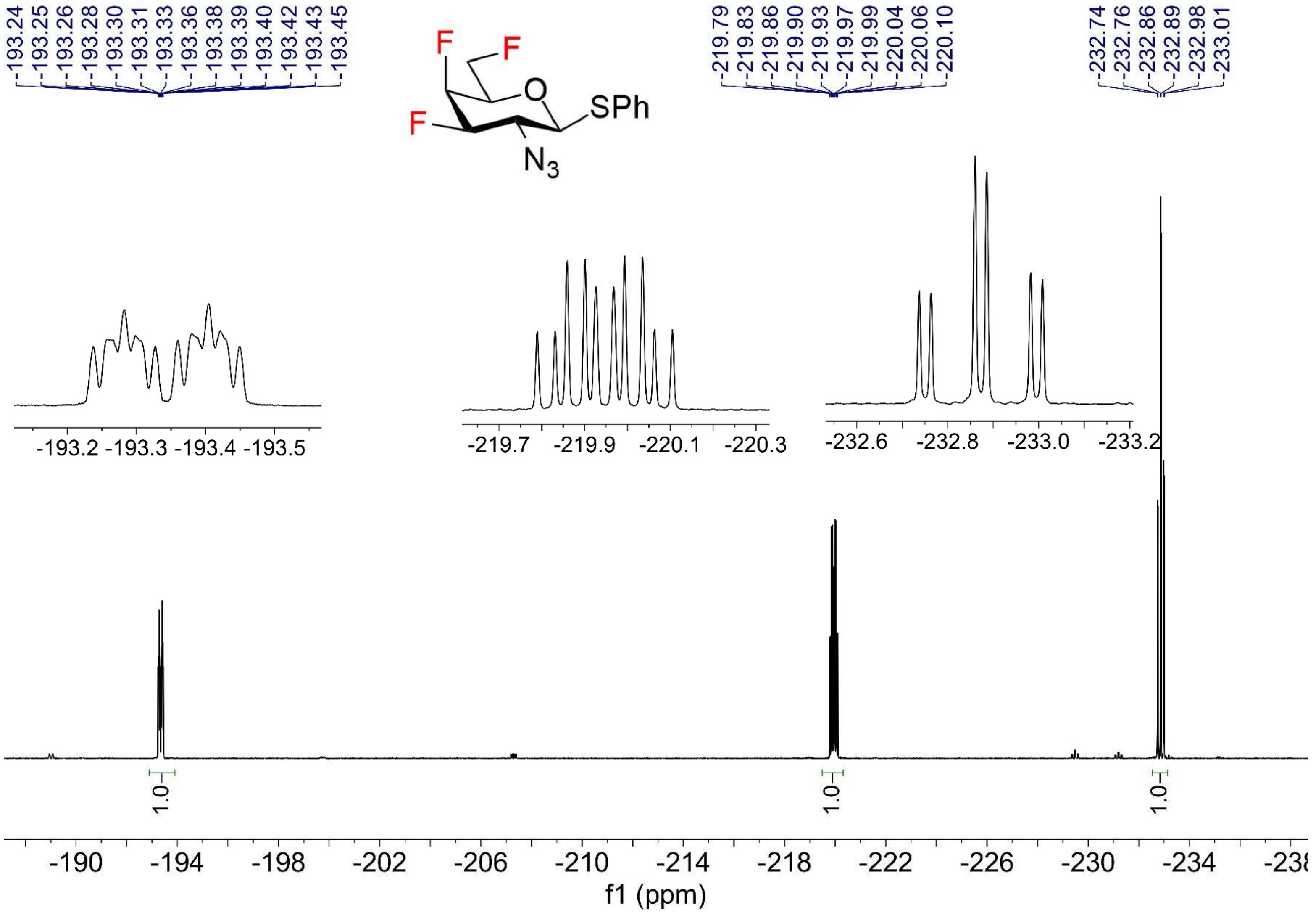

^{1}H-^{1}H COSY β-34

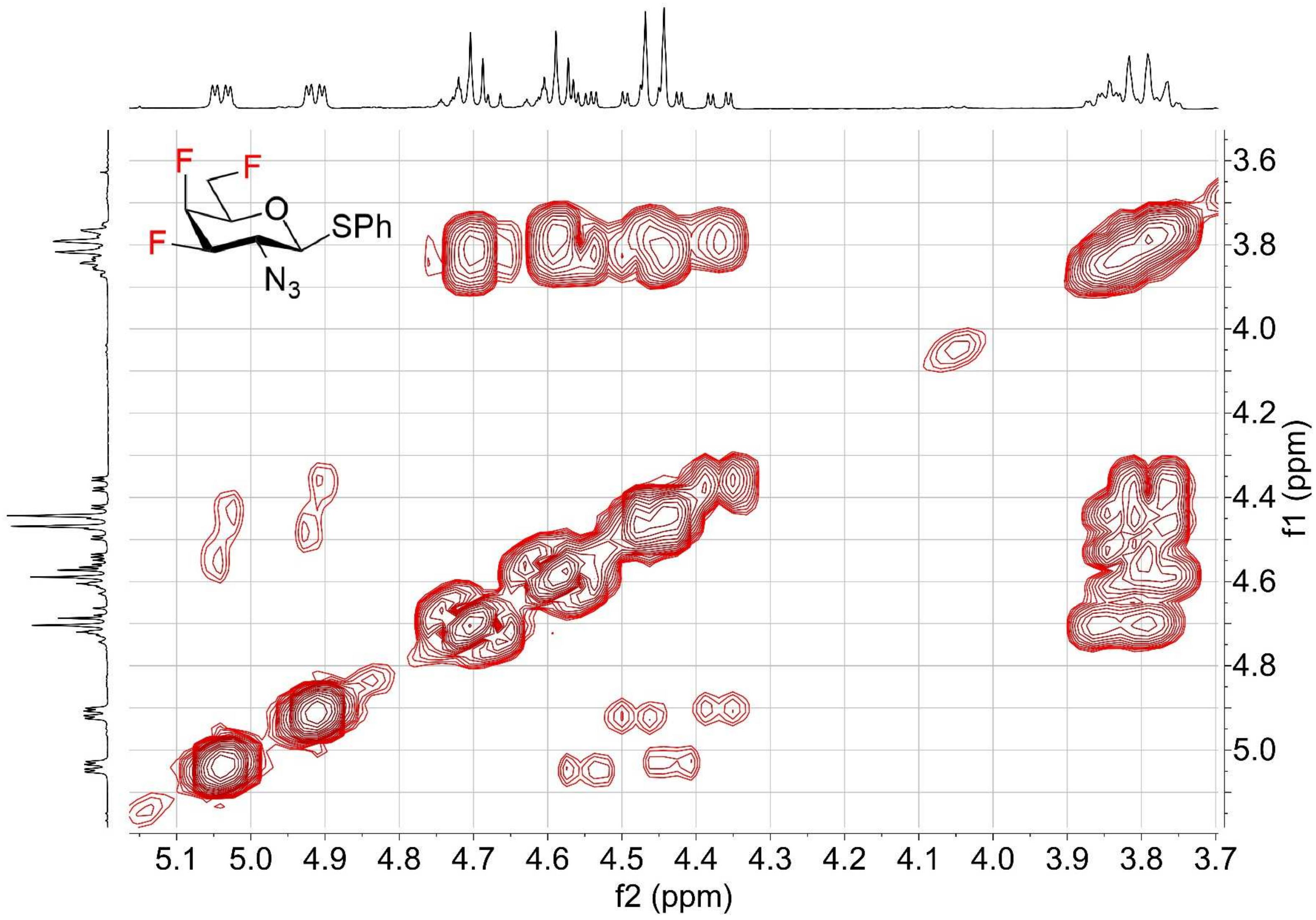

^{1}H-^{13}C HMBC β-34

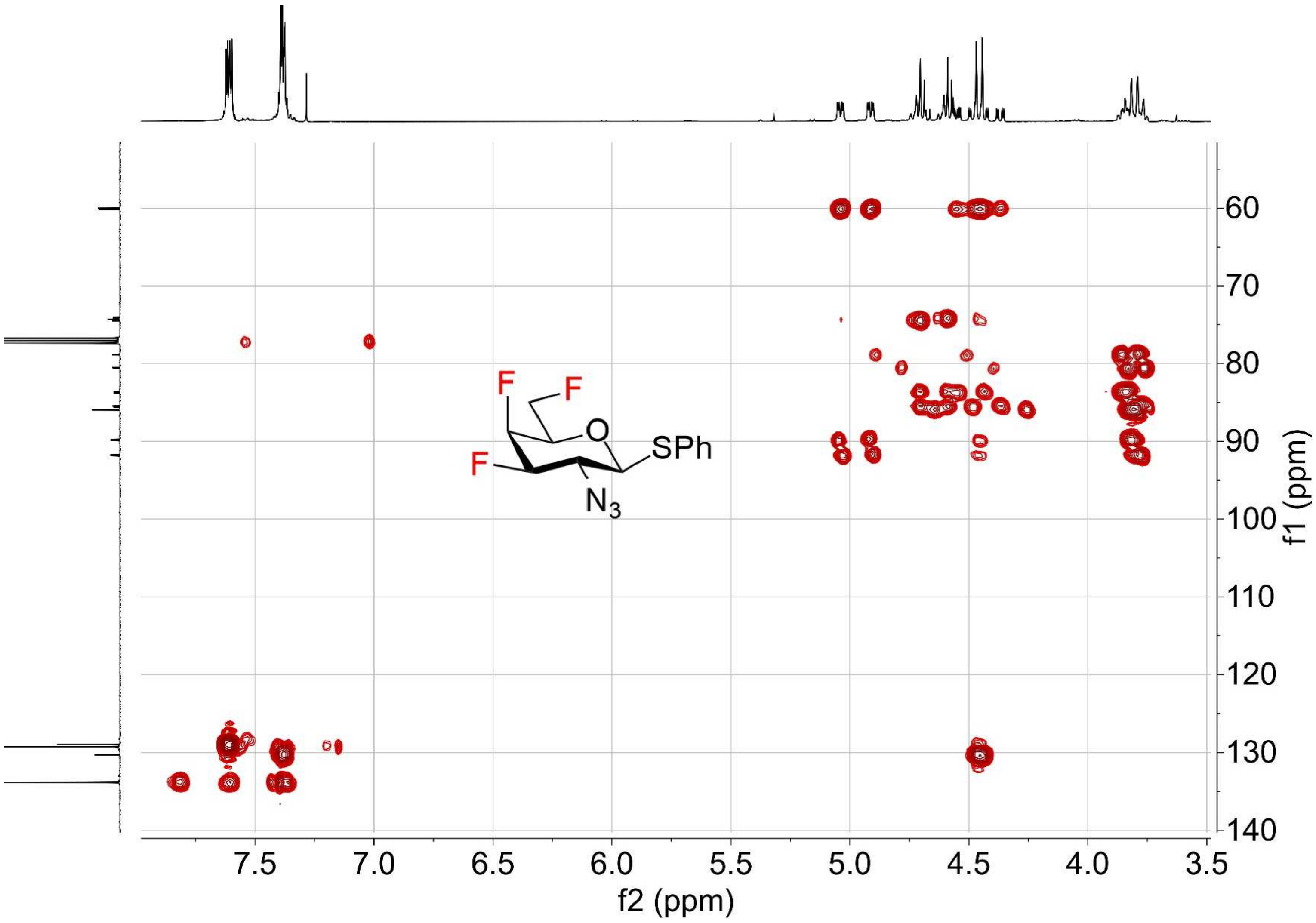

^{1}H-^{13}C HSQC β-34

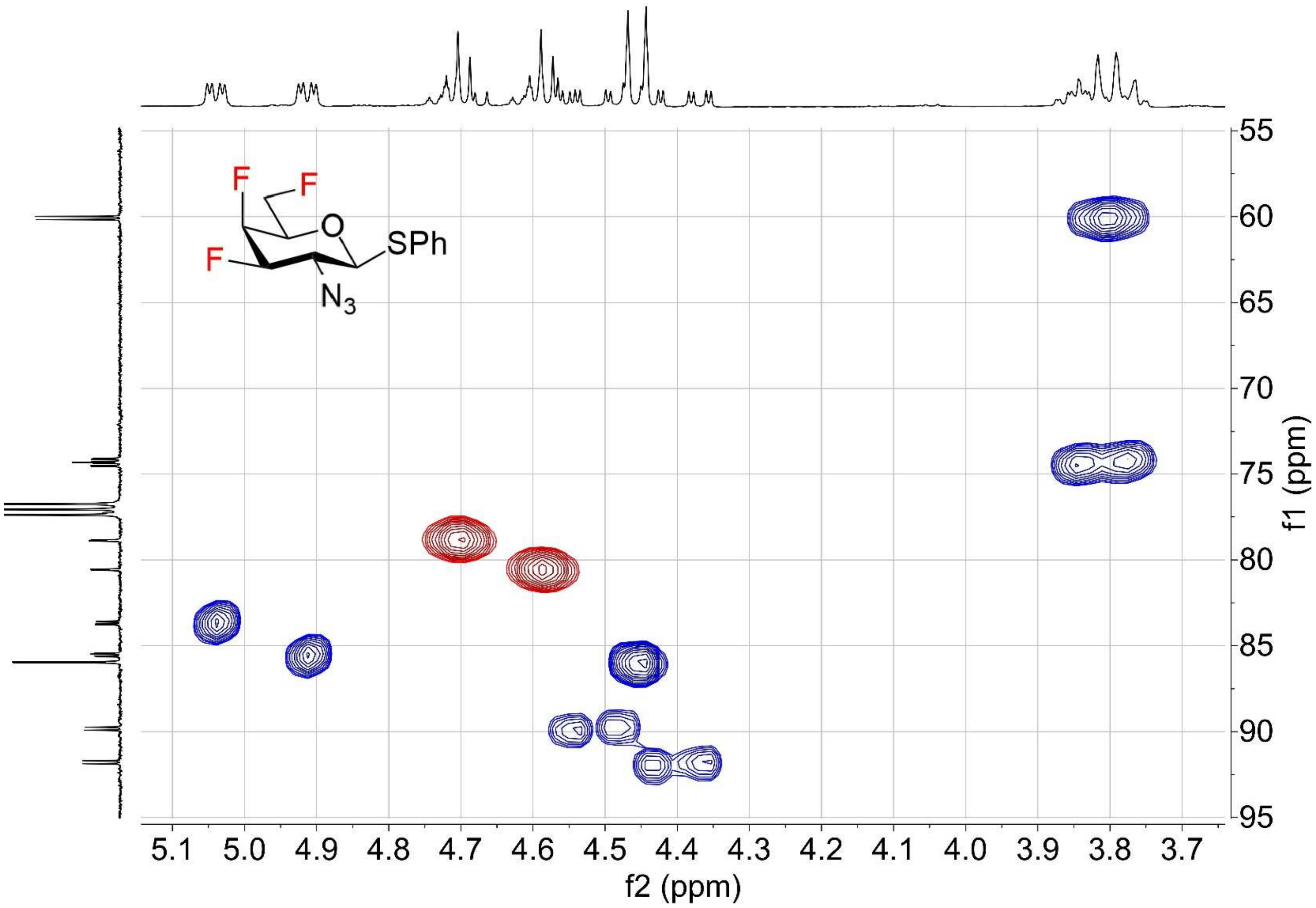

NMR COMPOUND α-35

^{1}H NMR (400 MHz, $CDCl_3$) α-35

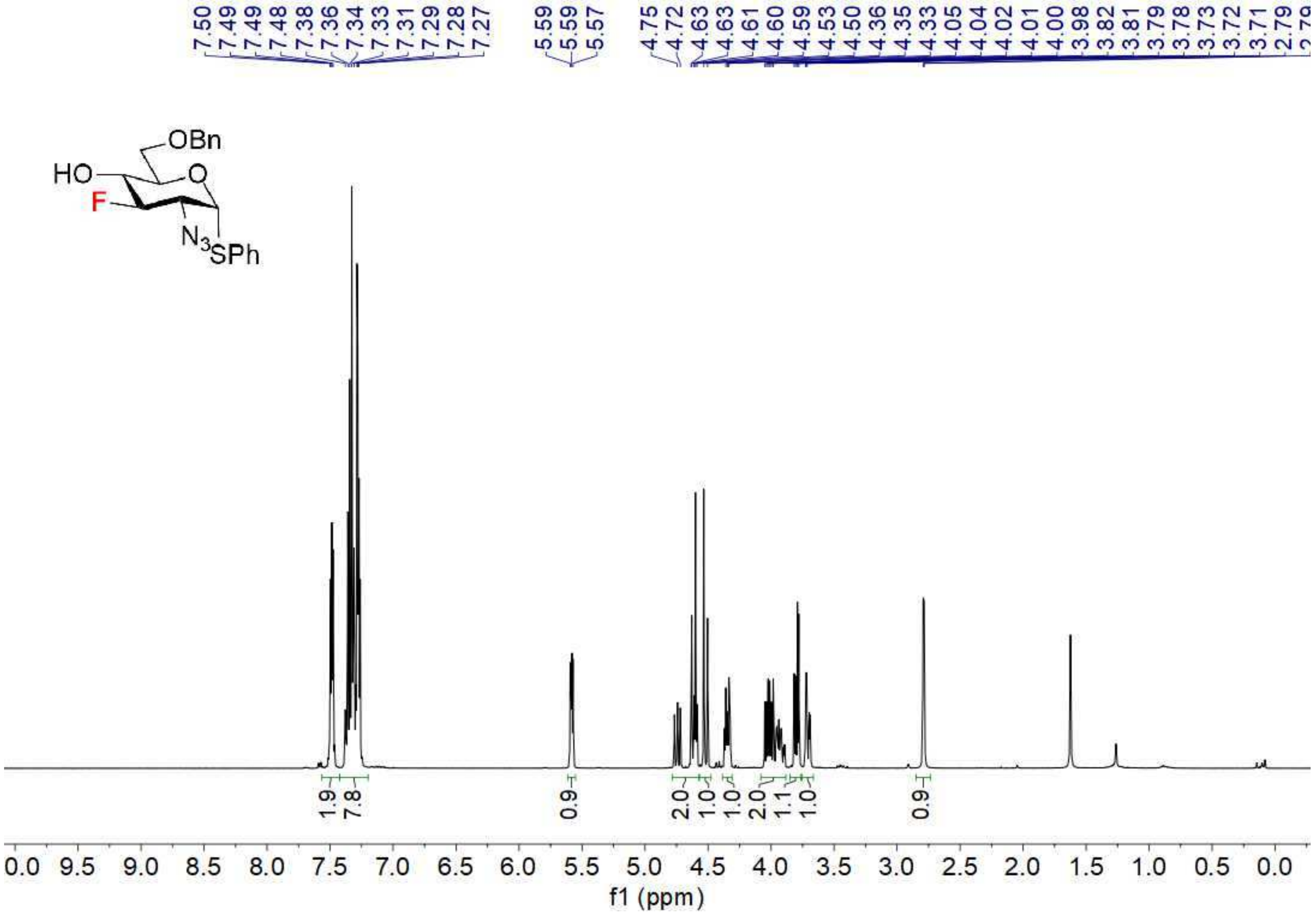

^{13}C NMR (100 MHz, CDCl$_3$) α-35

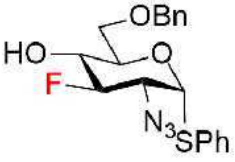

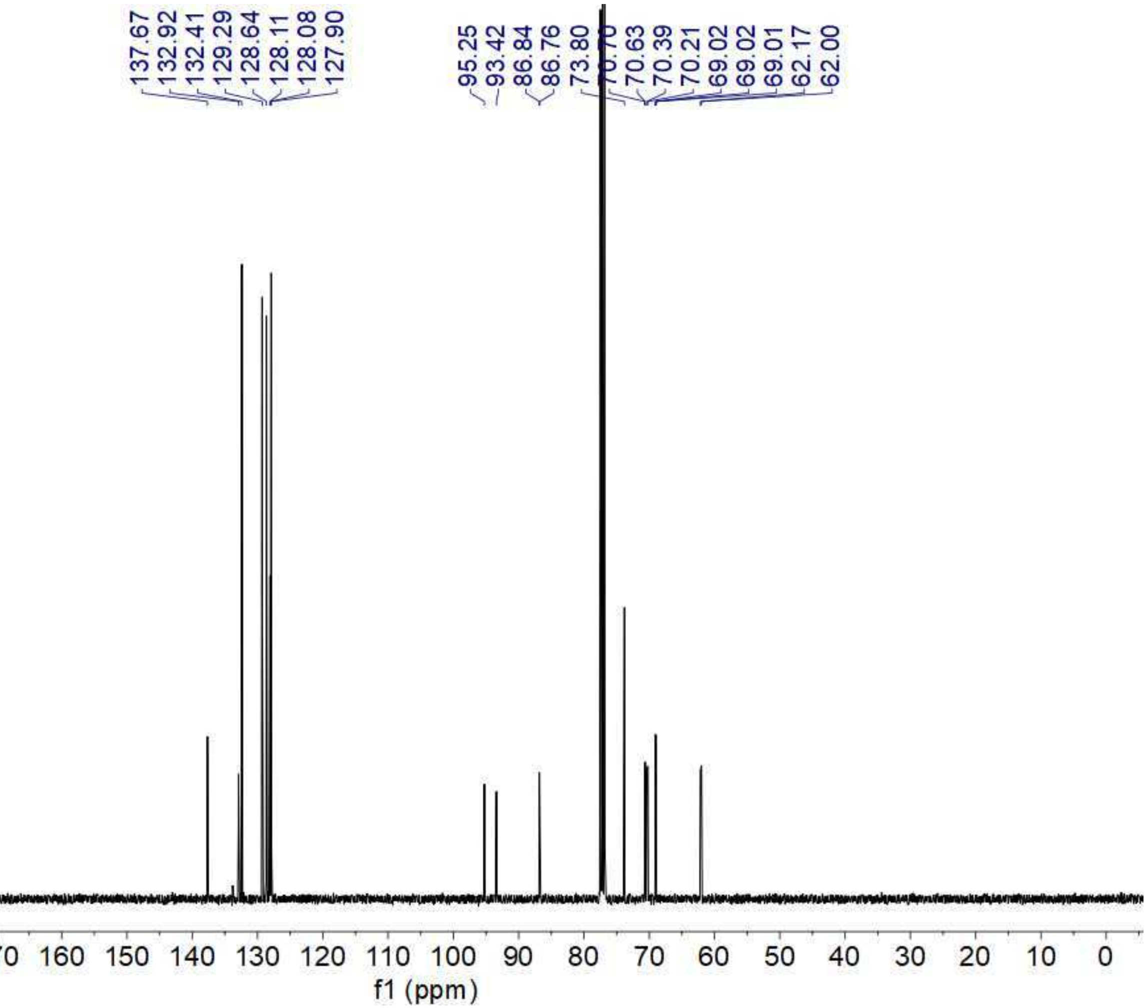

^{19}F NMR (376 MHz, CDCl$_3$) α-35

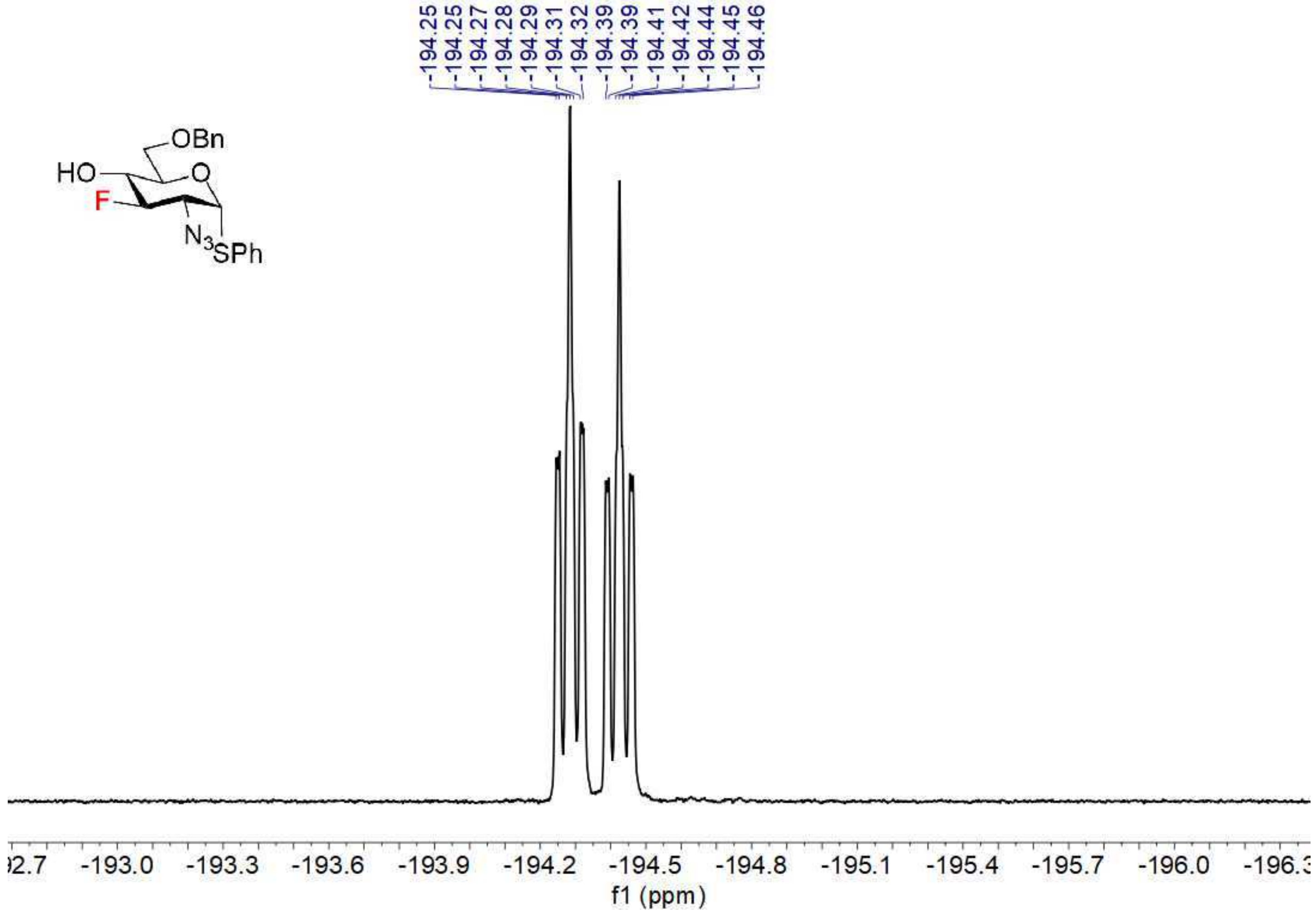

^{1}H-^{1}H COSY α-35

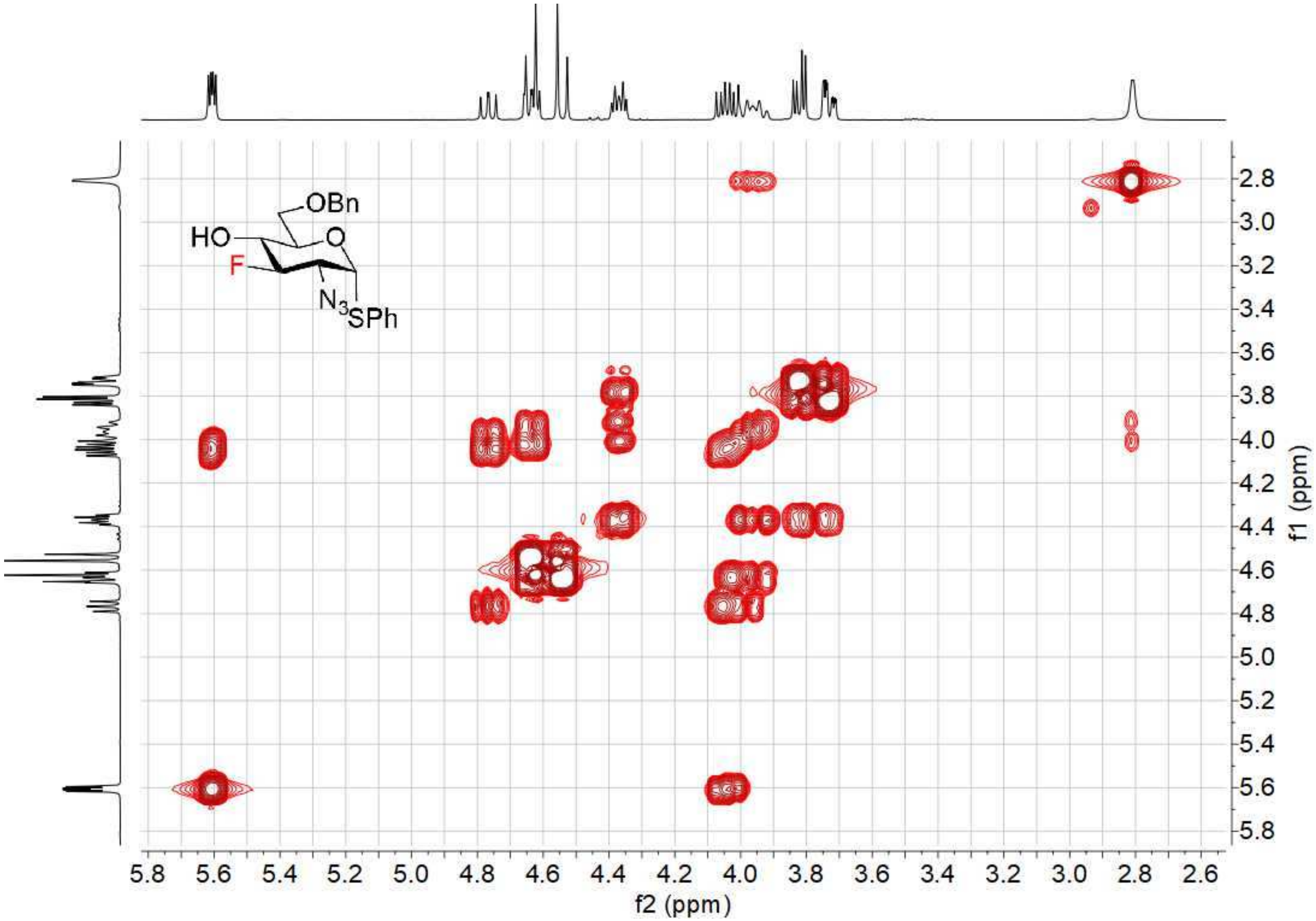

^{1}H-^{13}C HSQC α-35

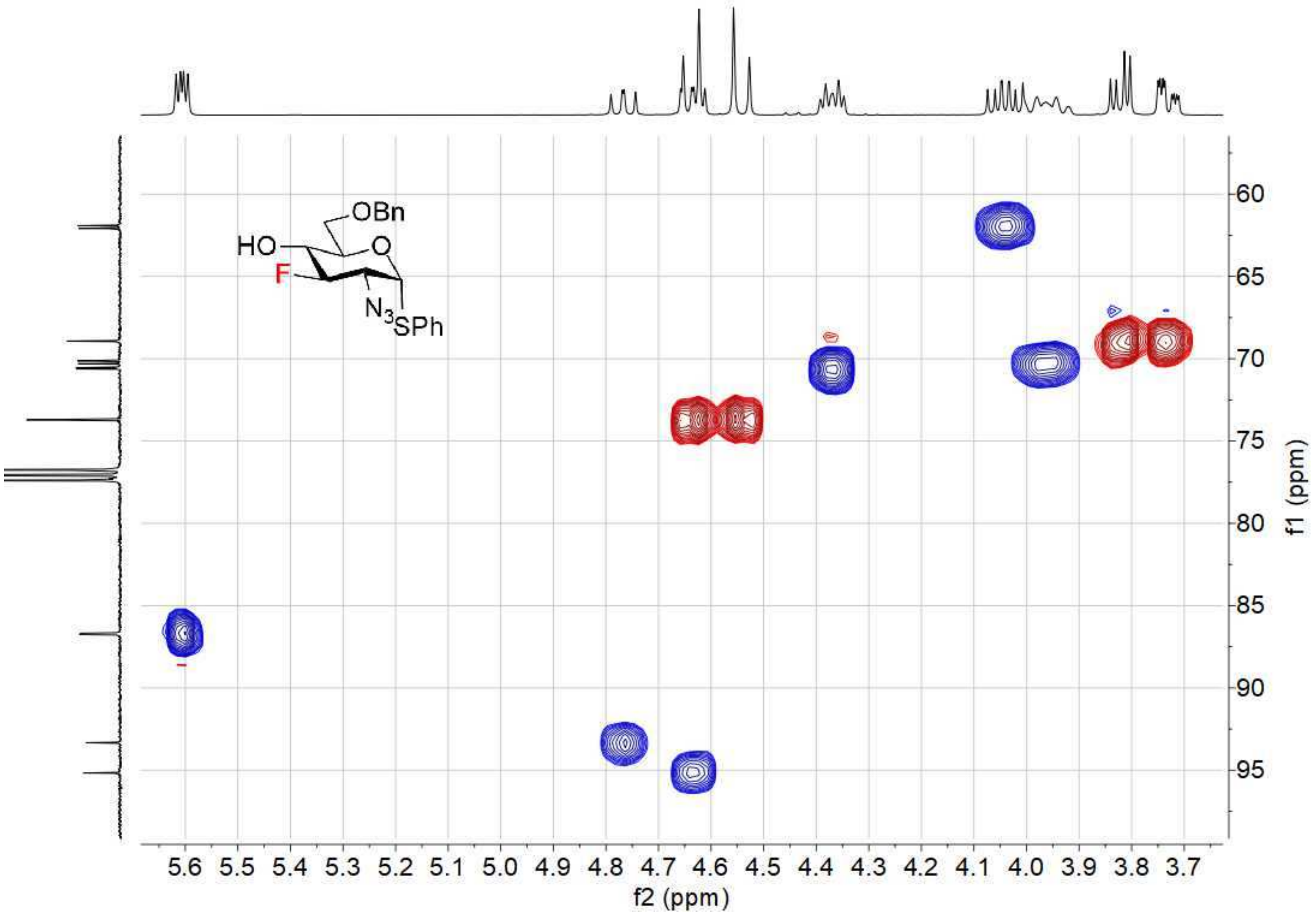

NMR COMPOUND 35 (α/β ca. 1/5)

^{1}H NMR (400 MHz, CDCl$_3$) 35 (α/β ca. 1/5)

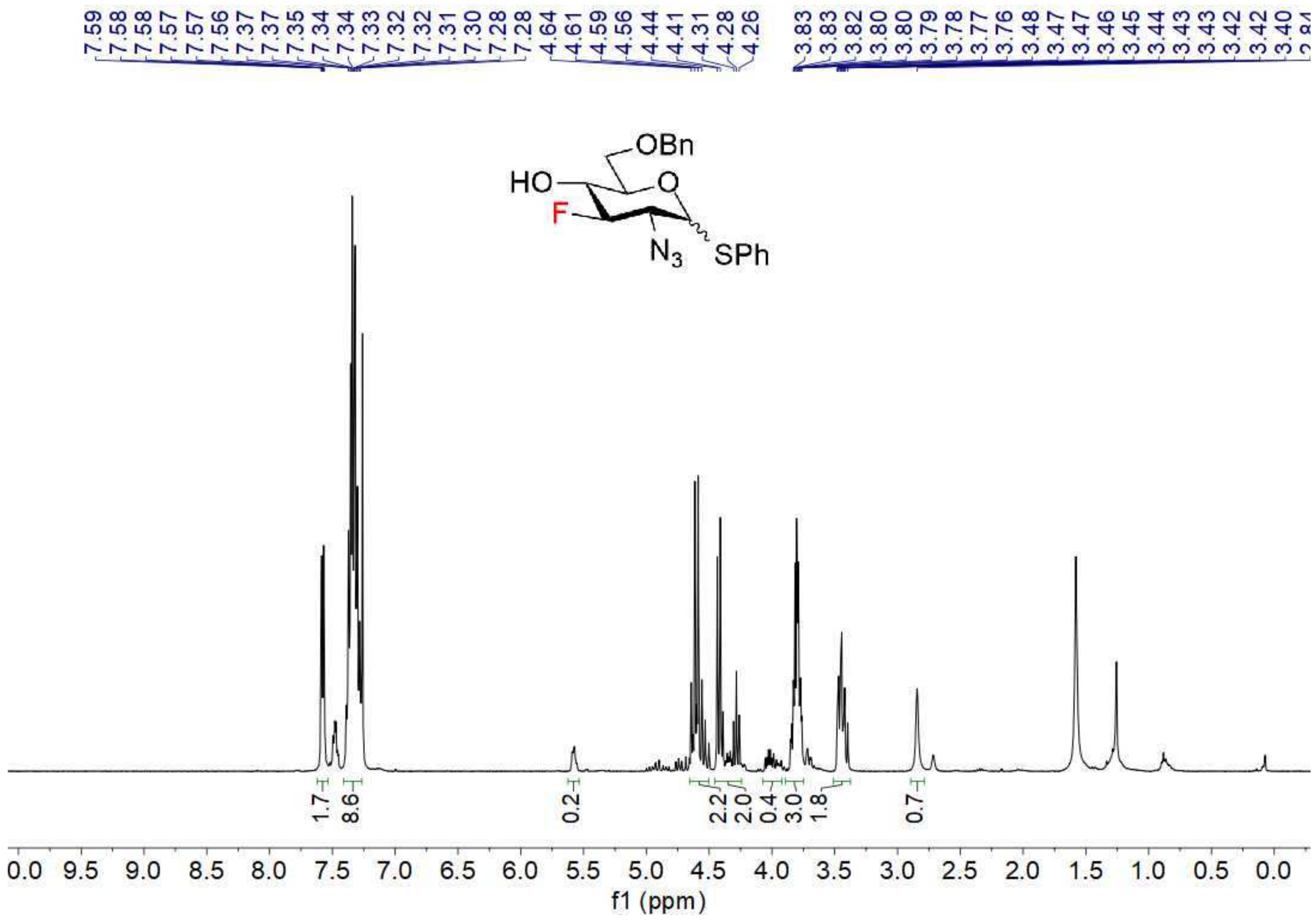

^{13}C NMR (100 MHz, CDCl$_3$) 35 (α/β ca. 1/5)

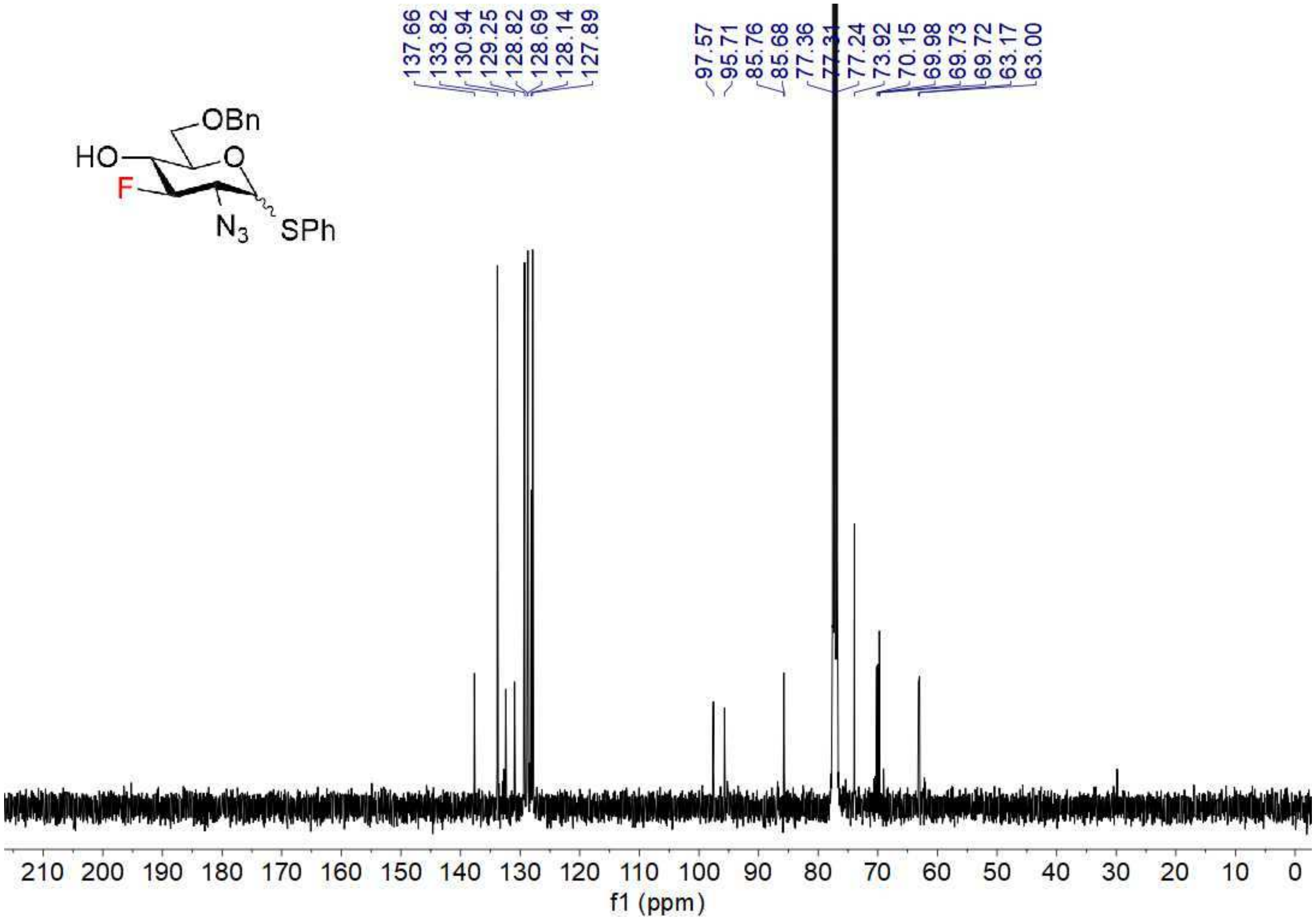

^{19}F NMR (376 MHz, $CDCl_3$) 35 (α/β ca. 1/5)

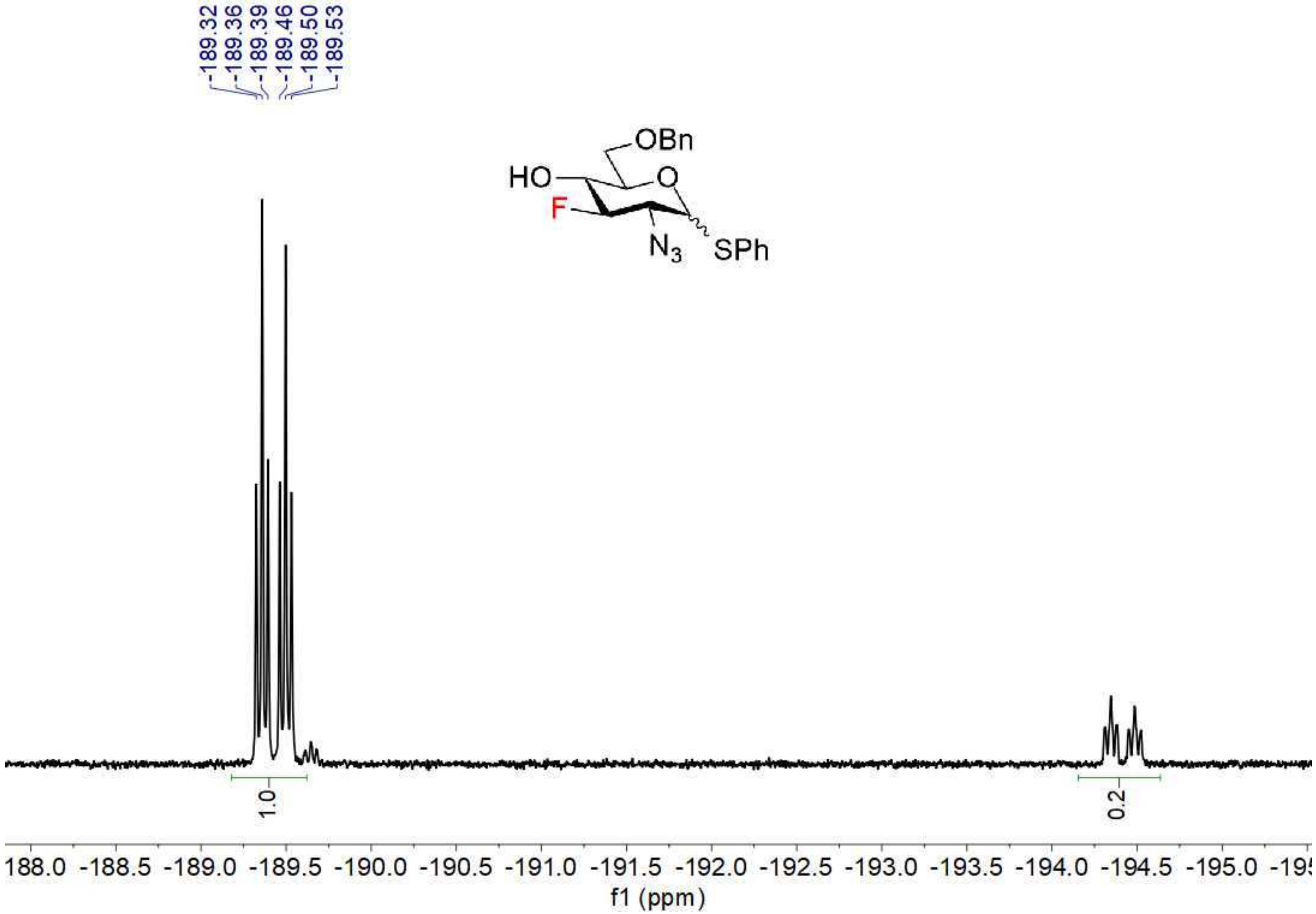

^{1}H-^{1}H COSY 35 (α/β ca. 1/5)

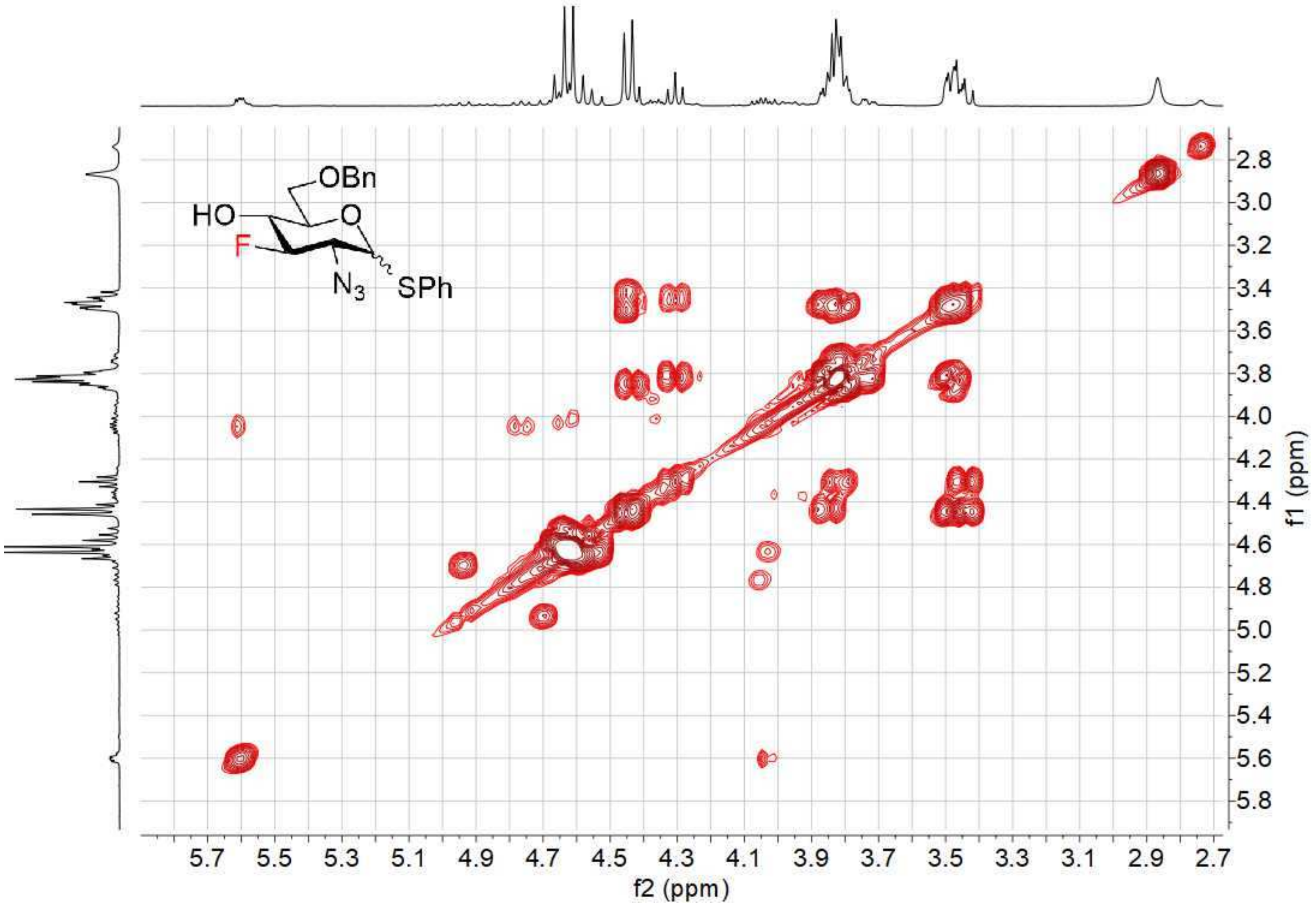

^{1}H-^{13}C HSQC 35 (α/β ca. 1/5)

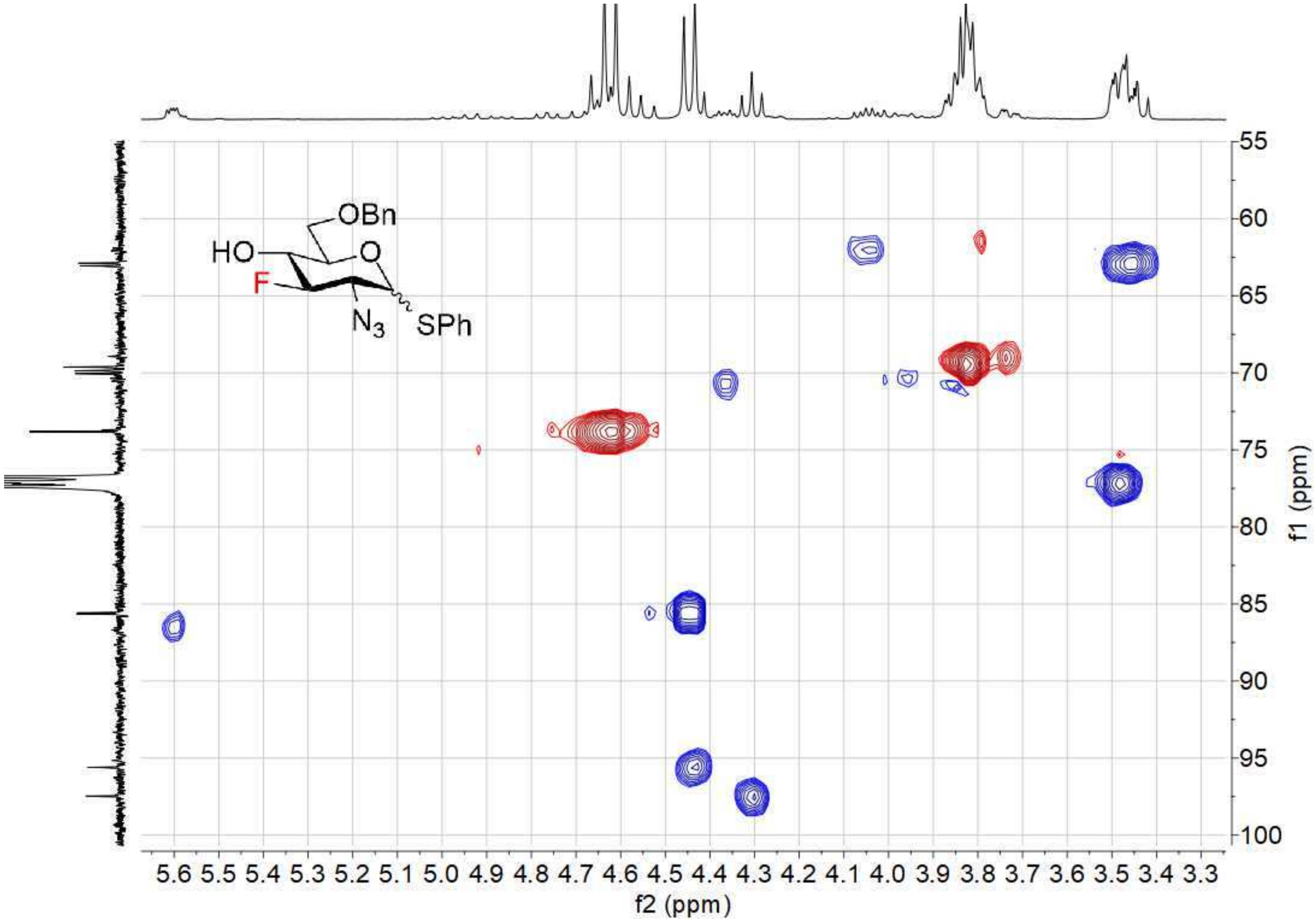

NMR COMPOUND α-36

^{1}H NMR (400 MHz, CDCl$_3$) α-36

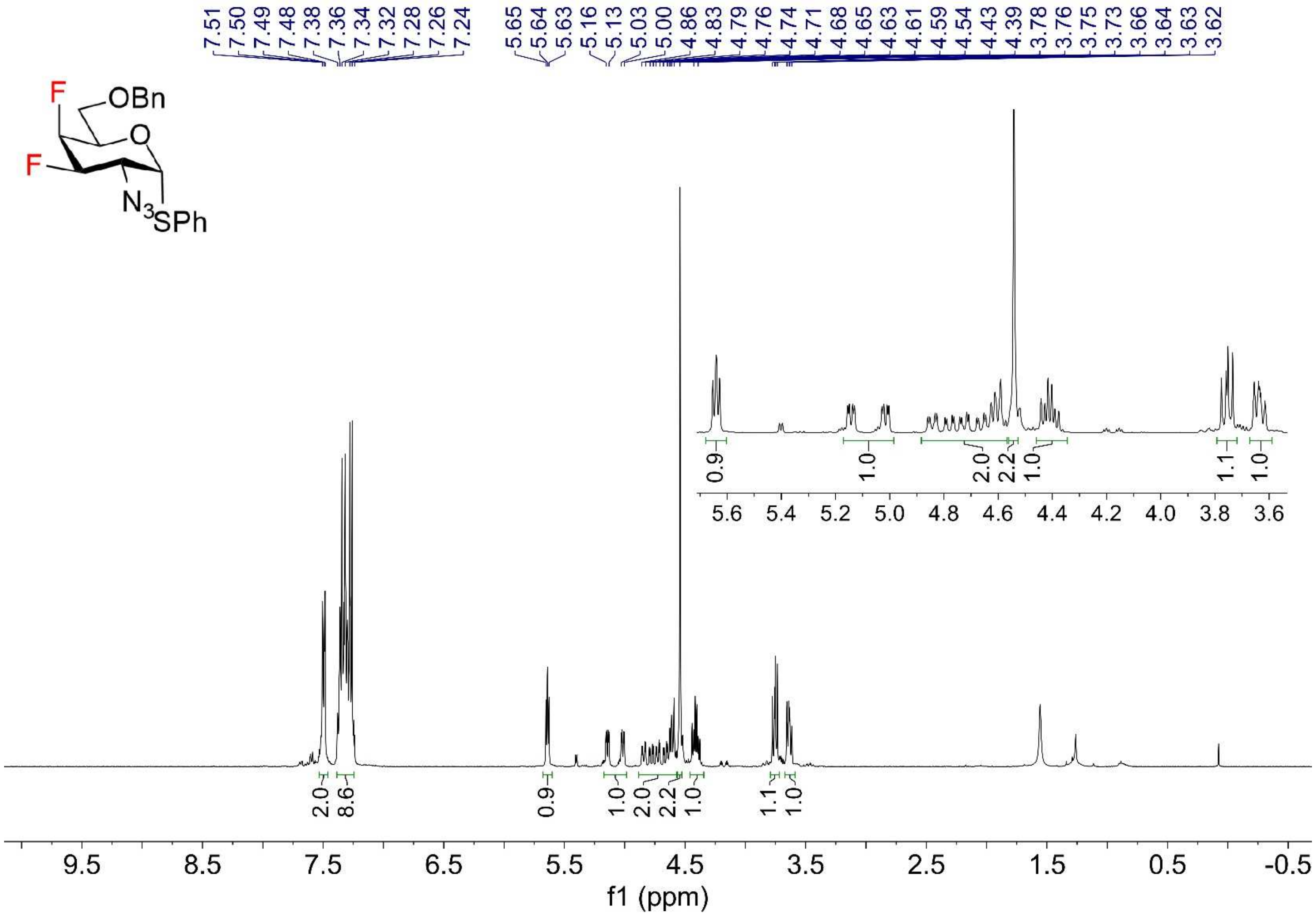

^{13}C NMR (100 MHz, CDCl$_3$) α-36

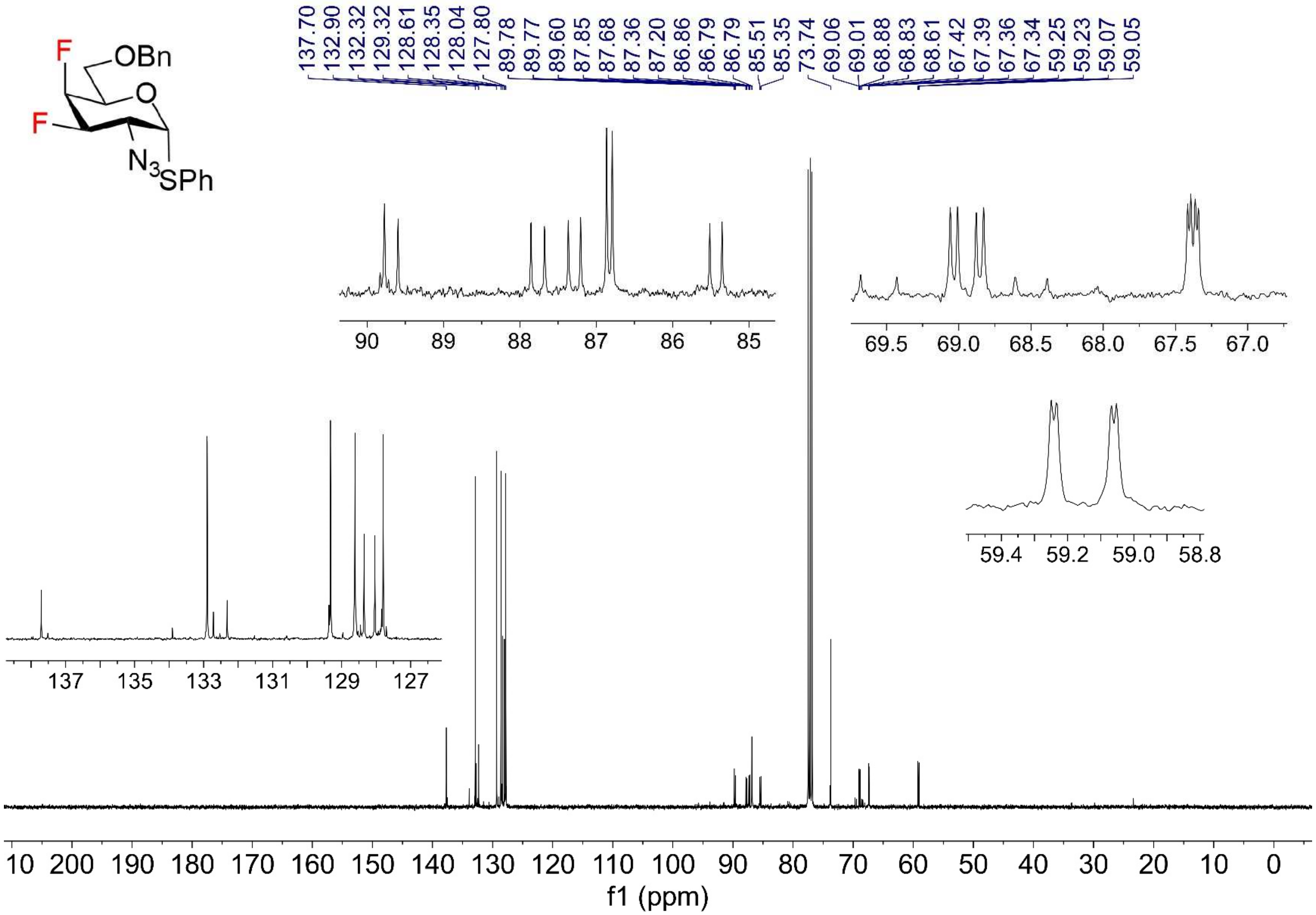

^{19}F NMR (376 MHz, CDCl$_3$) α-36

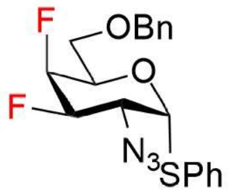

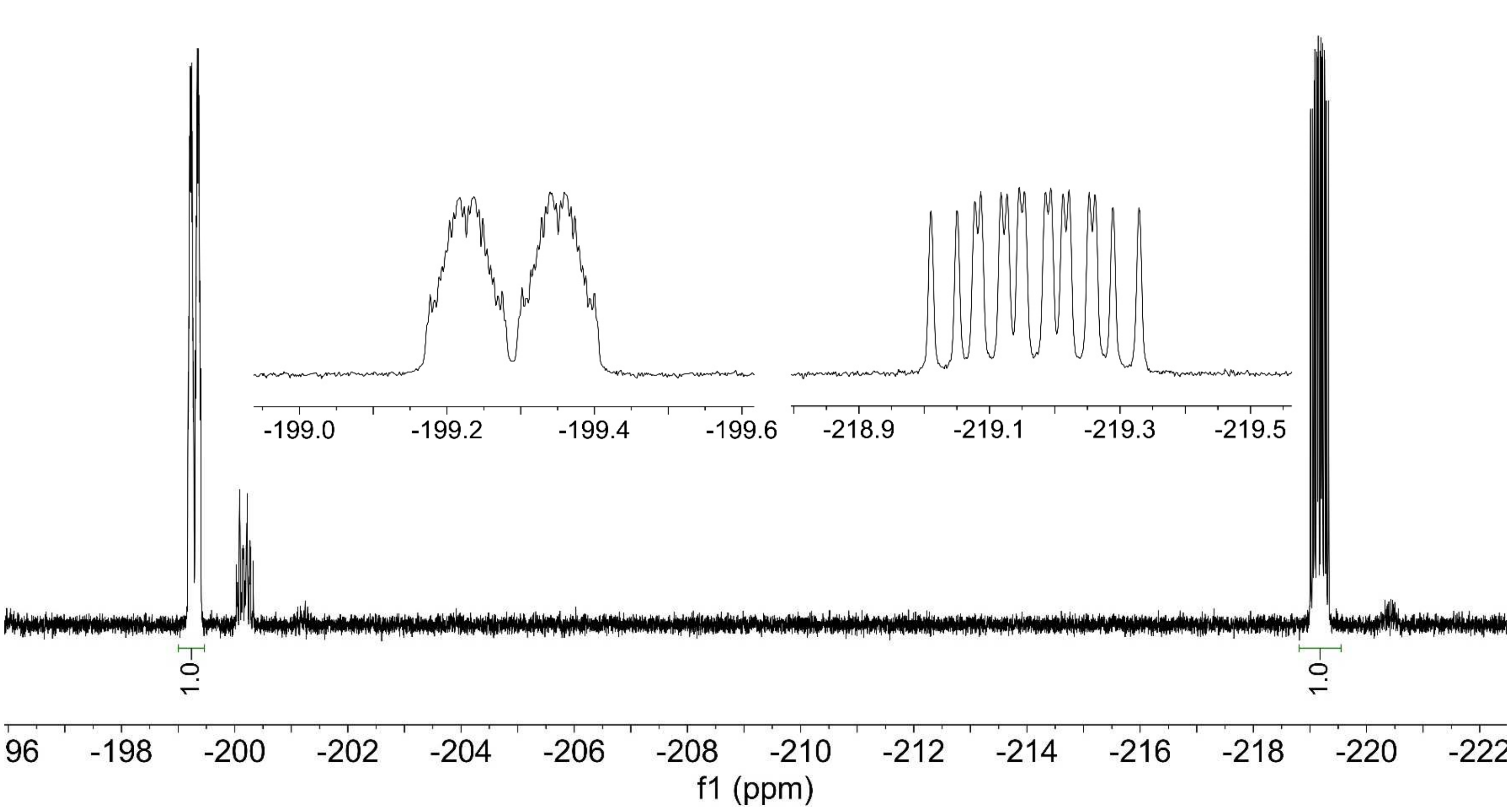

^{1}H-^{1}H COSY α-36

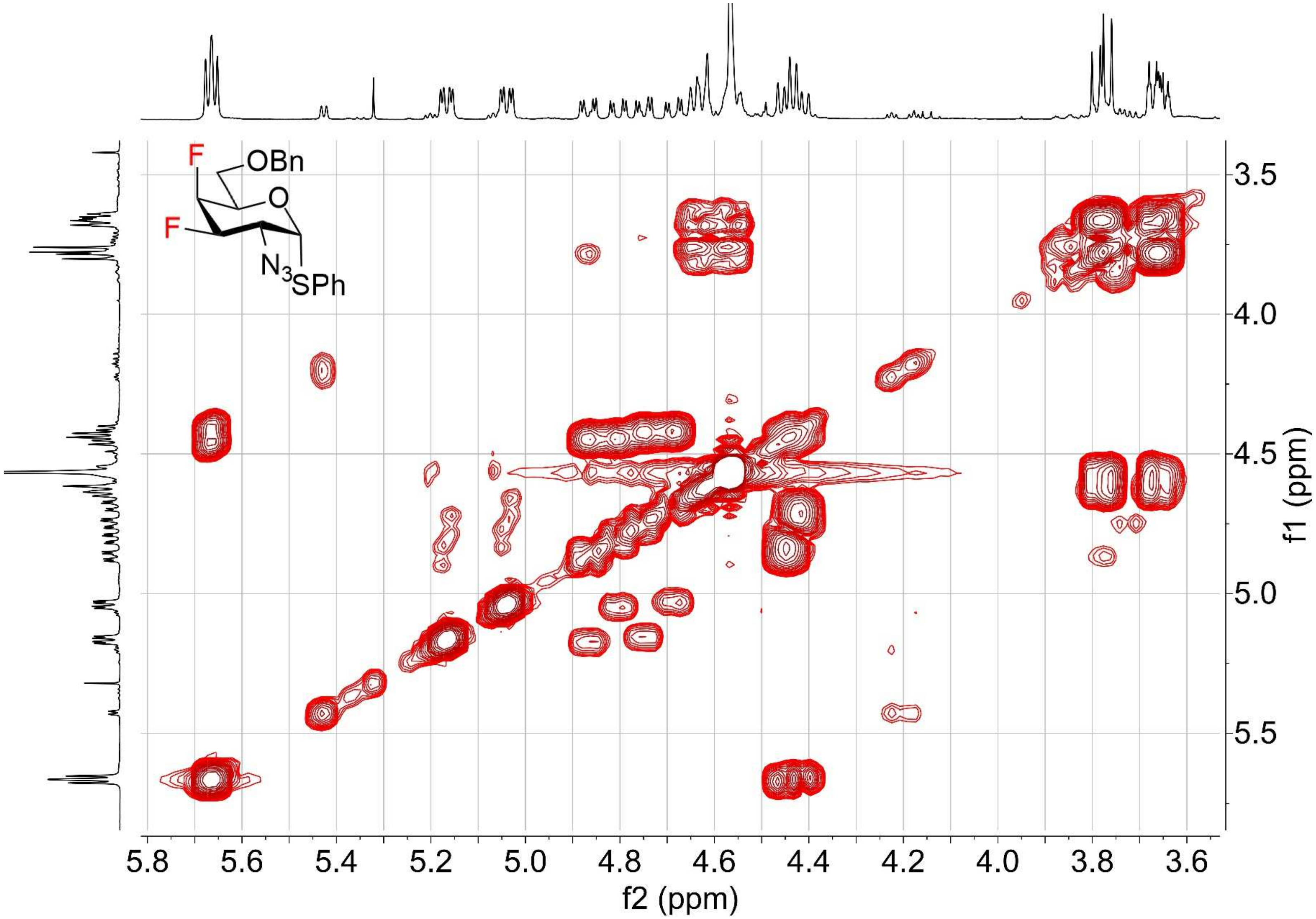

^{1}H-^{13}C HSQC α-36

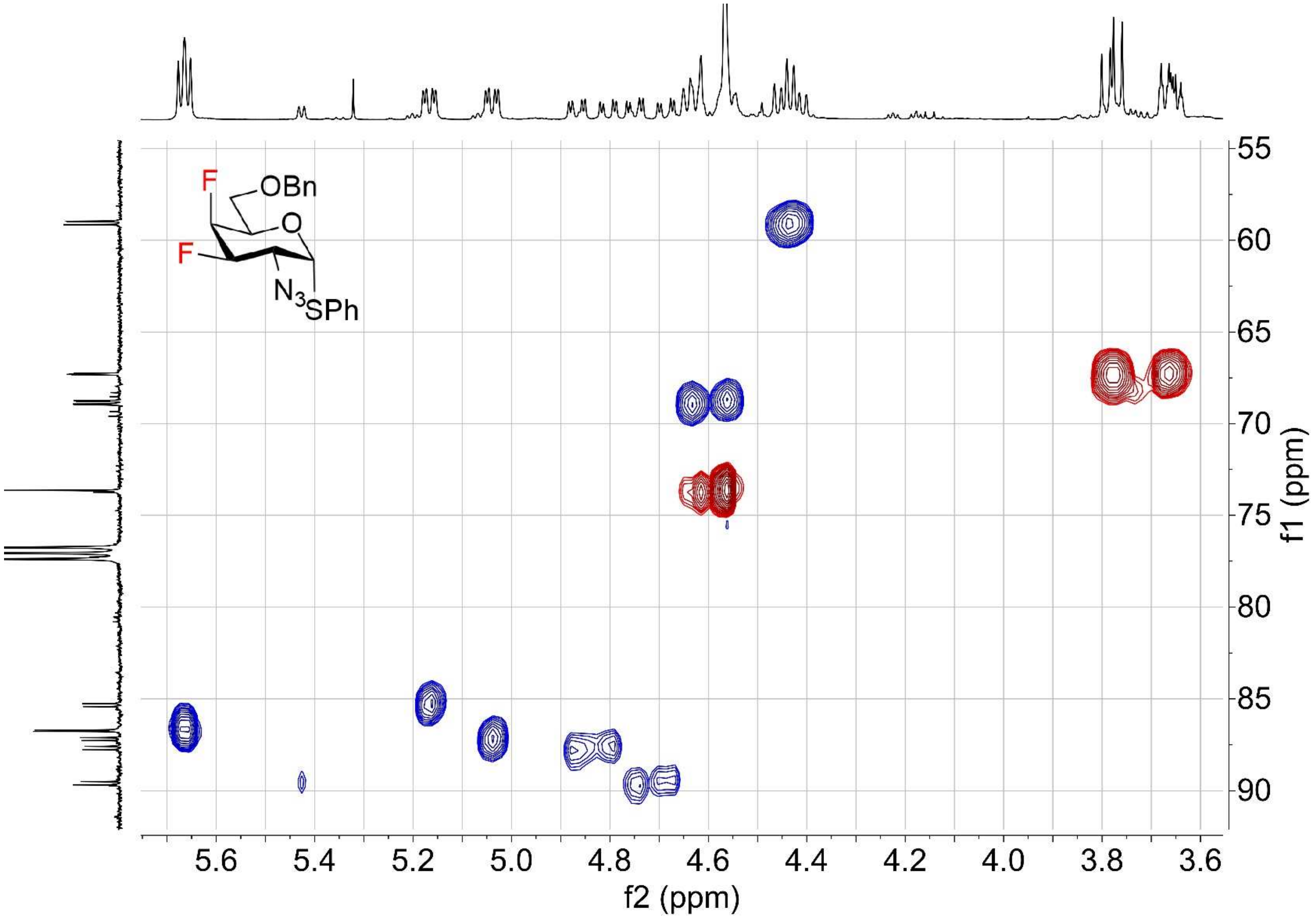

NMR COMPOUND β-36

^{1}H NMR (400 MHz, CDCl₃) β-38

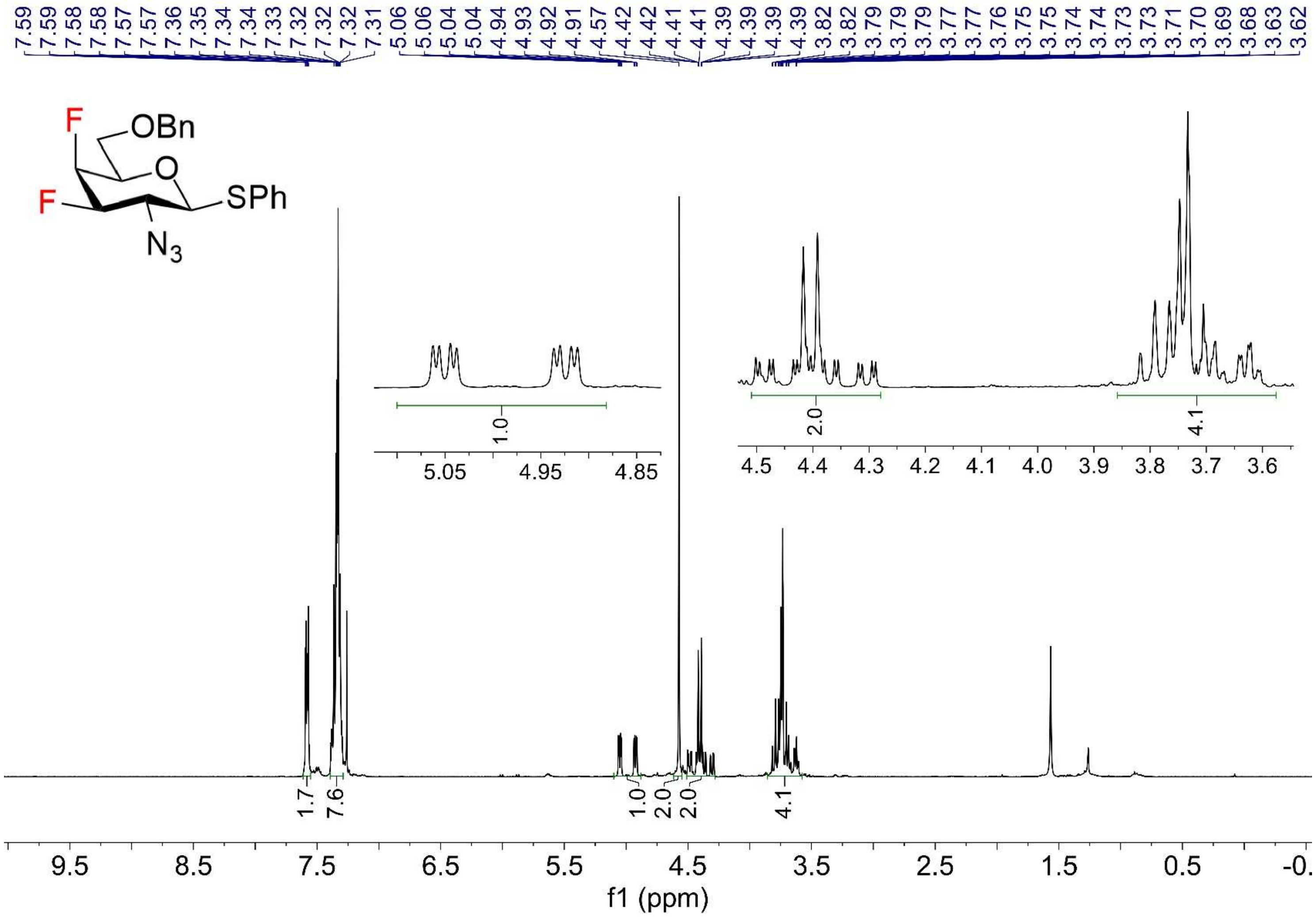

^{13}C NMR (100 MHz, $CDCl_3$) β-36

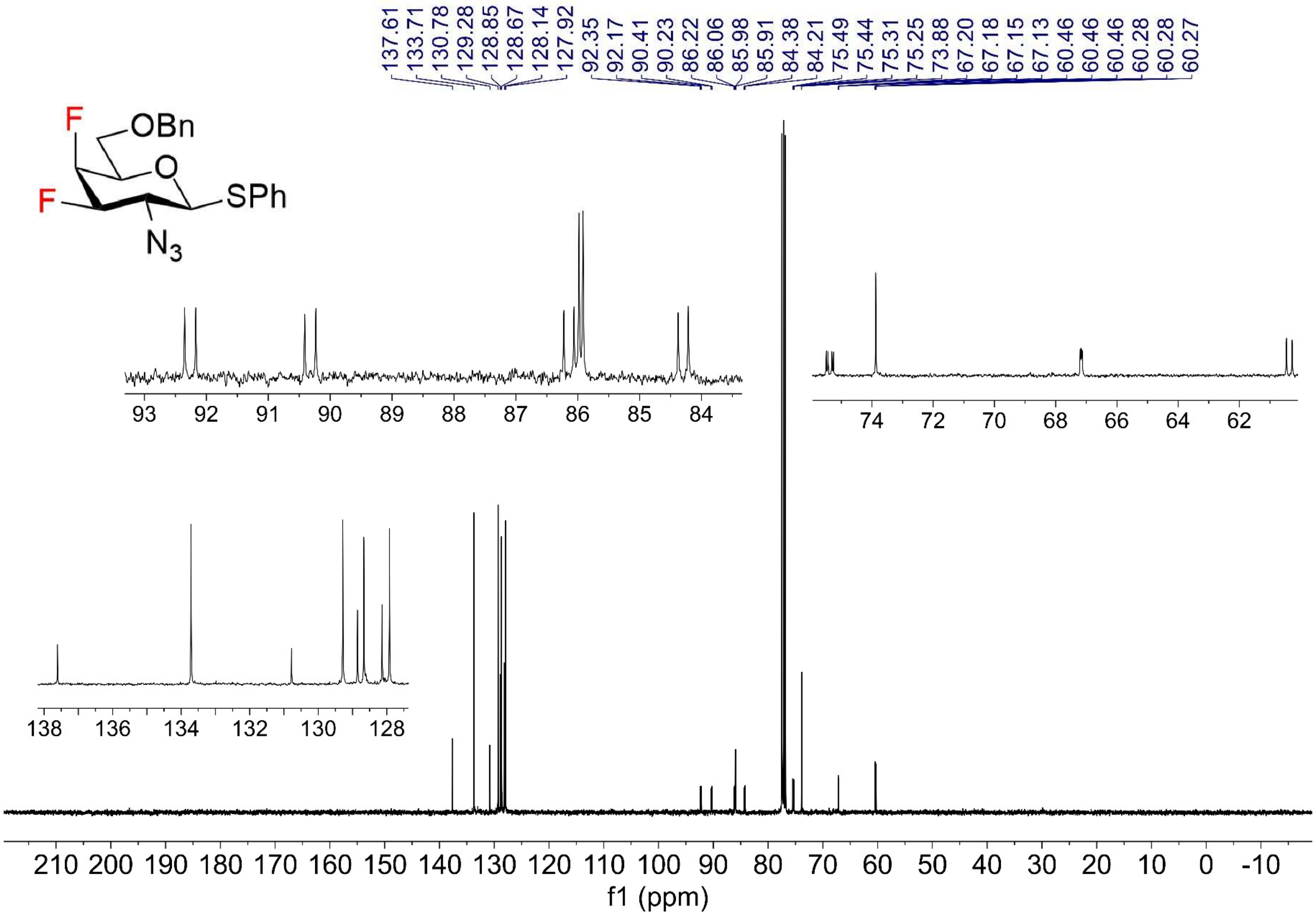

^{19}F NMR (376 MHz, CDCl$_3$) β-36

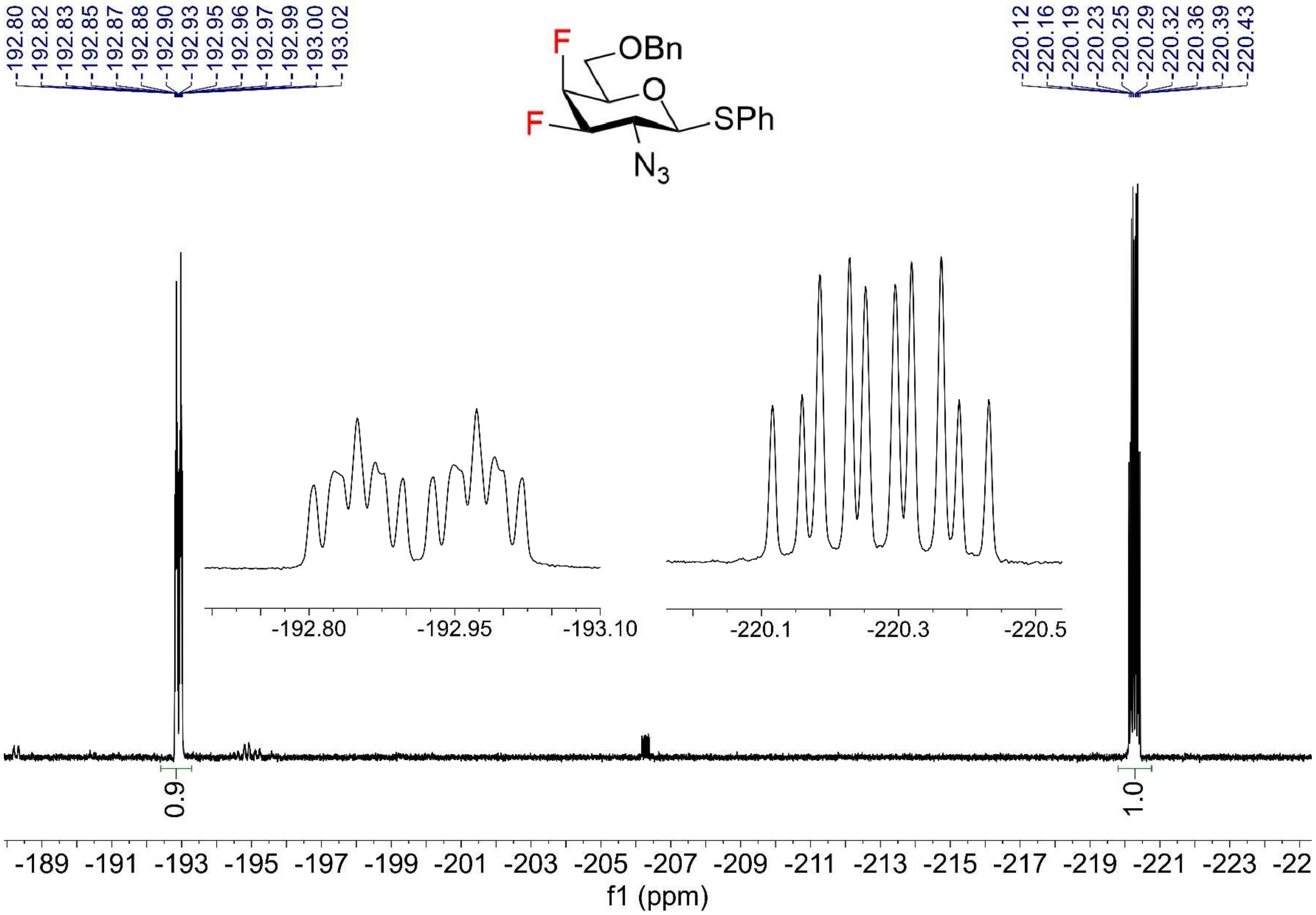

^{1}H-^{1}H COSY β-36

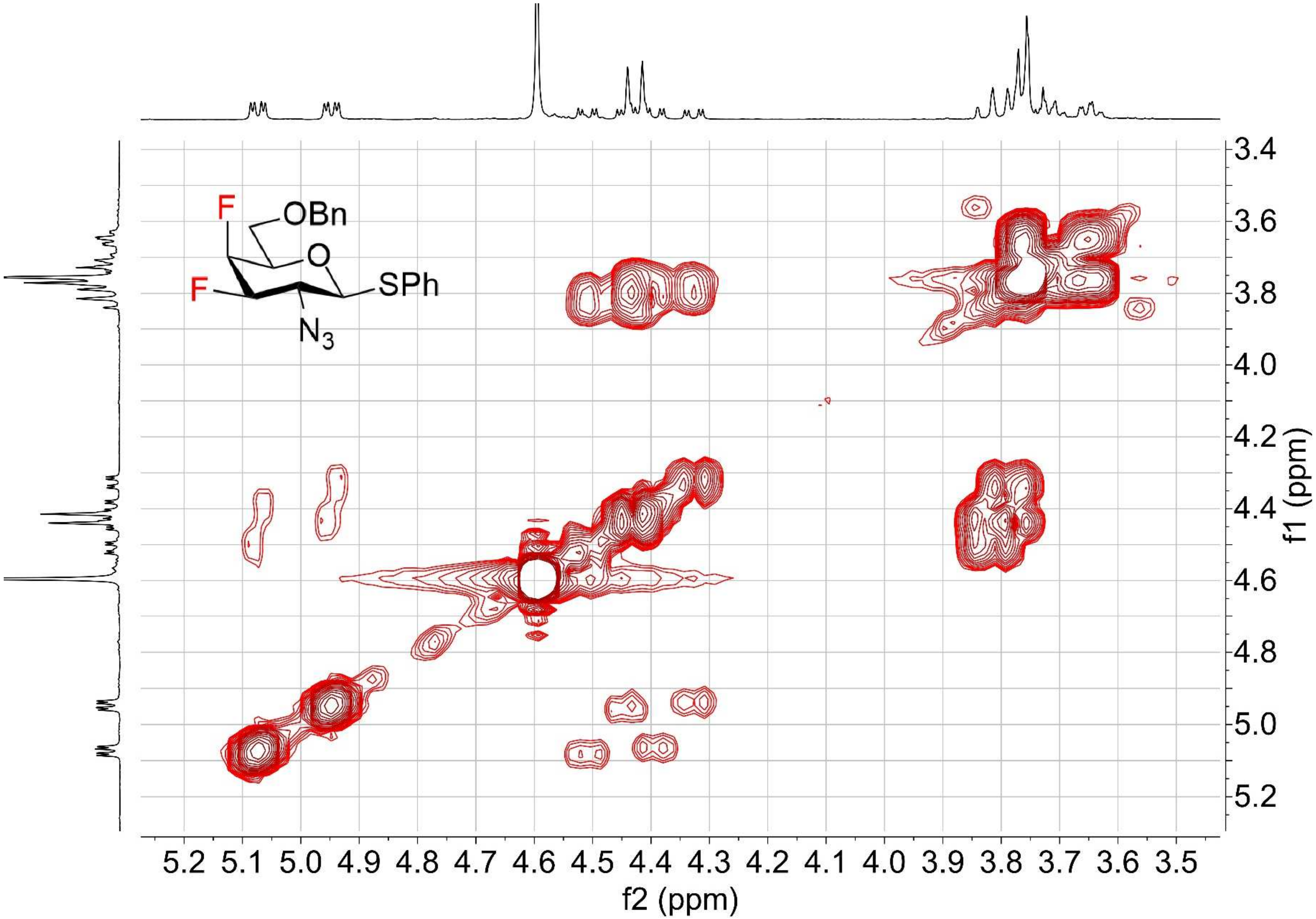

^{1}H-^{13}C HSQC β-36

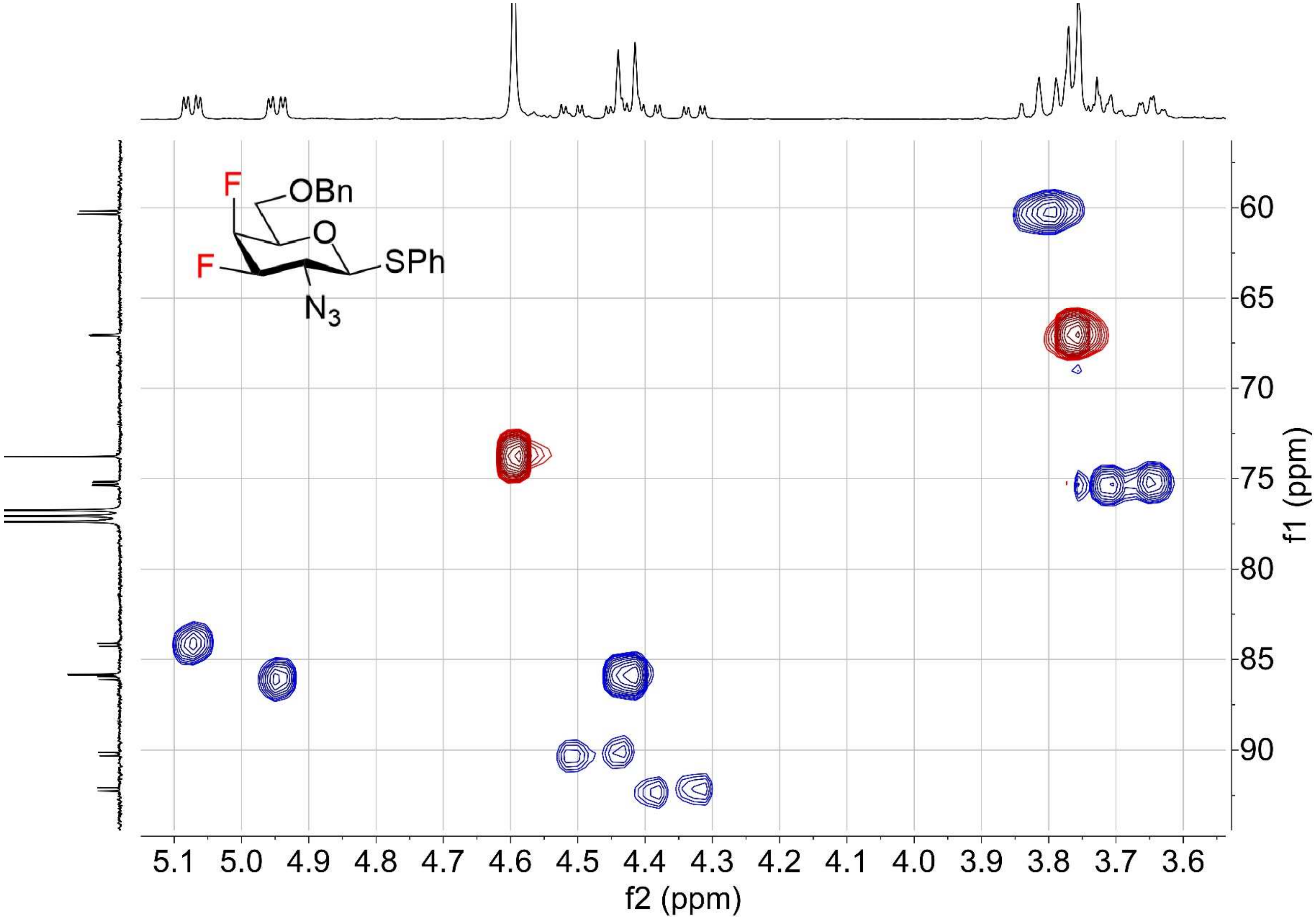

NMR COMPOUND 37

^{1}H NMR (400 MHz, $CDCl_3$) 37 (α/β ca. 5/3)

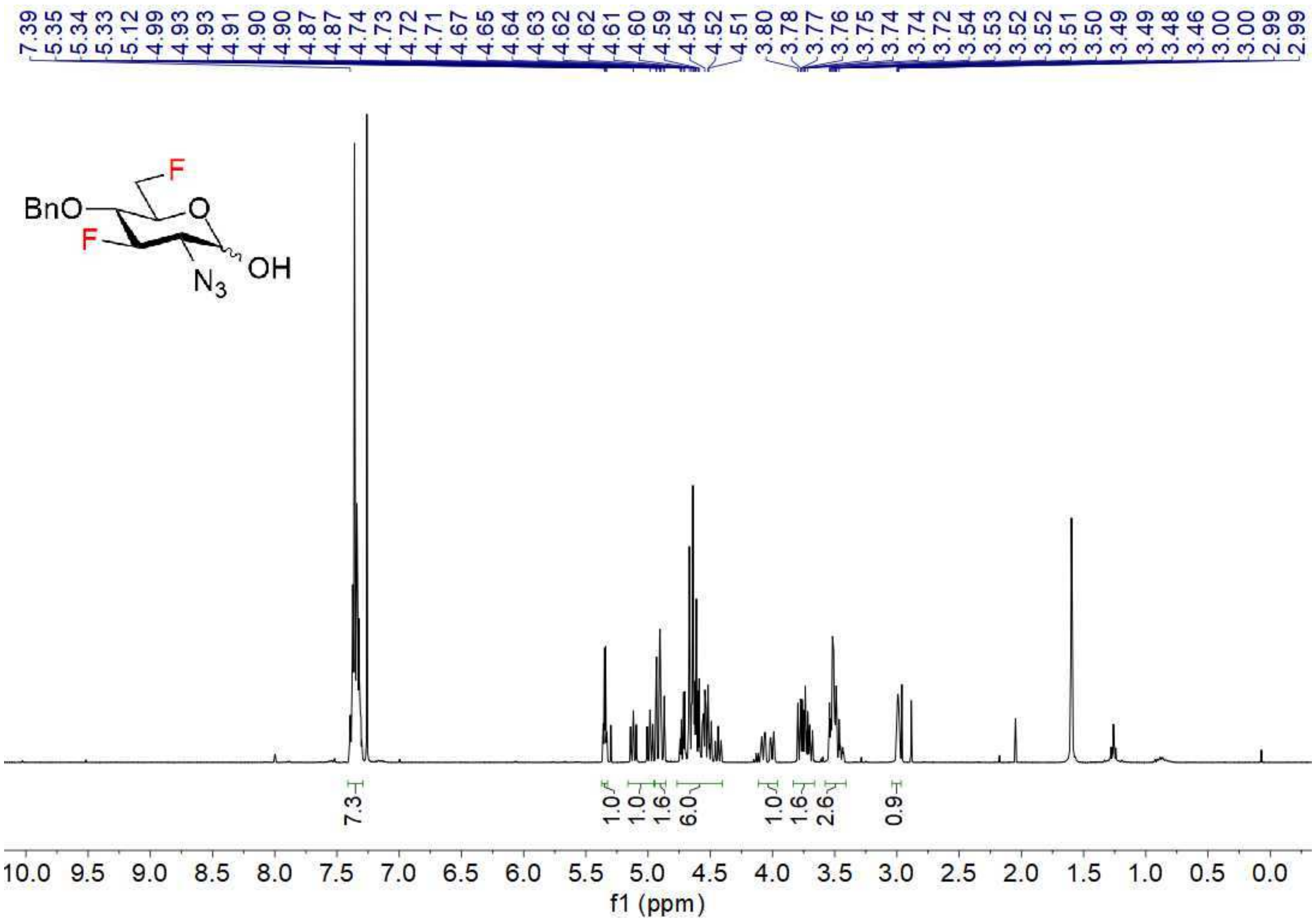

^{13}C NMR (100 MHz, CDCl$_3$) 37

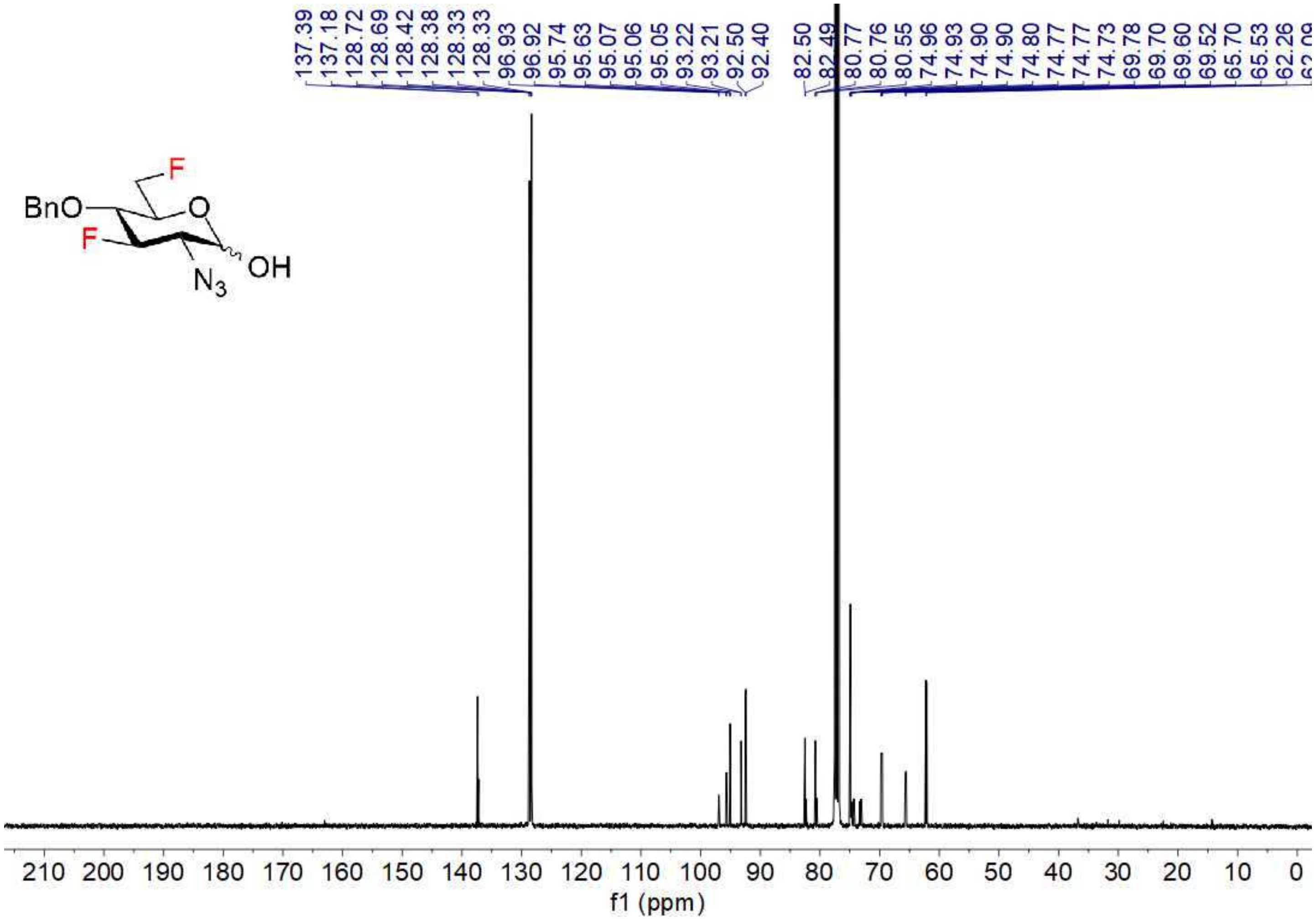

^{19}F NMR (376 MHz, $CDCl_3$) 37 (α/β ca. 5/3)

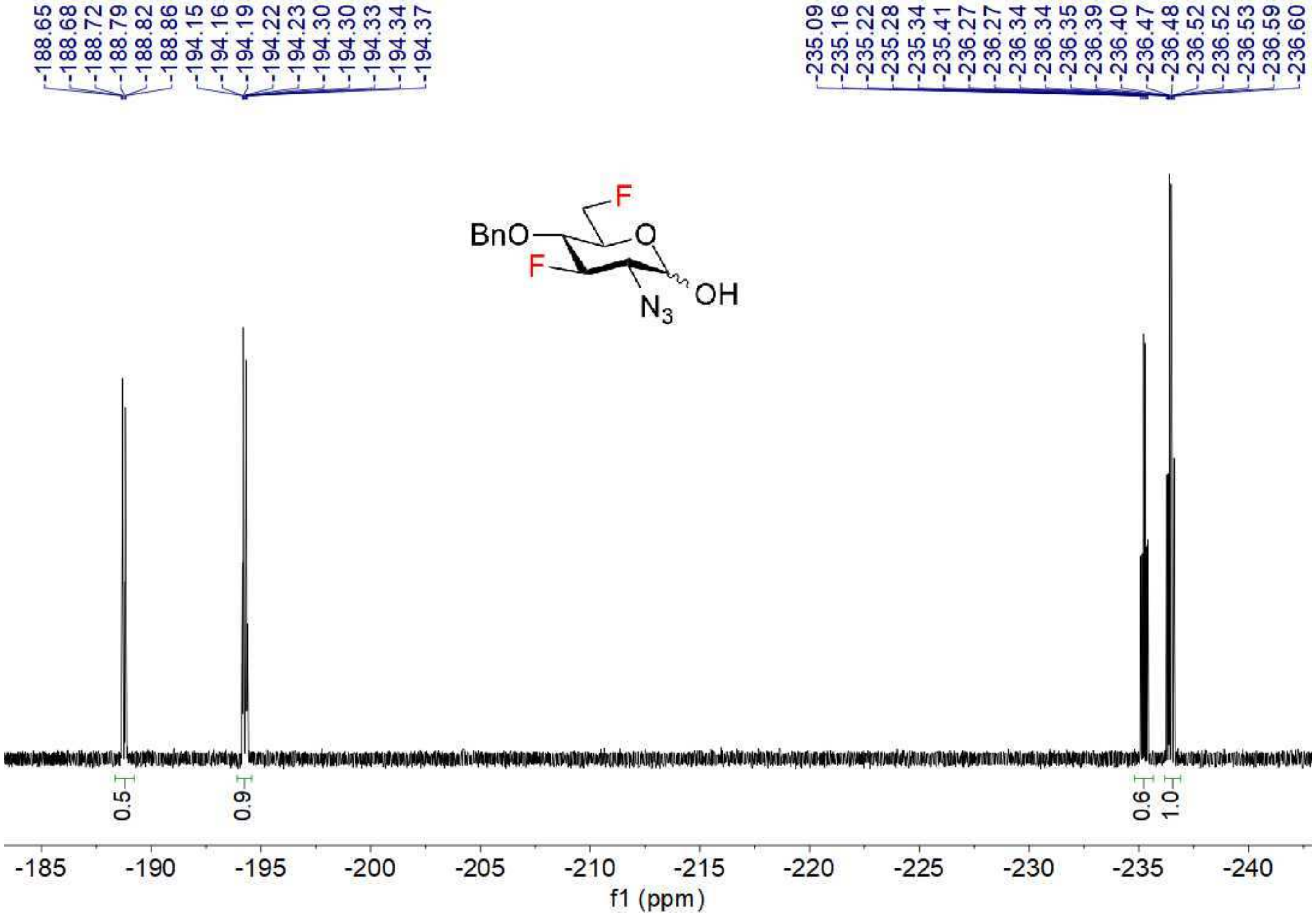

^{1}H-^{1}H COSY 37

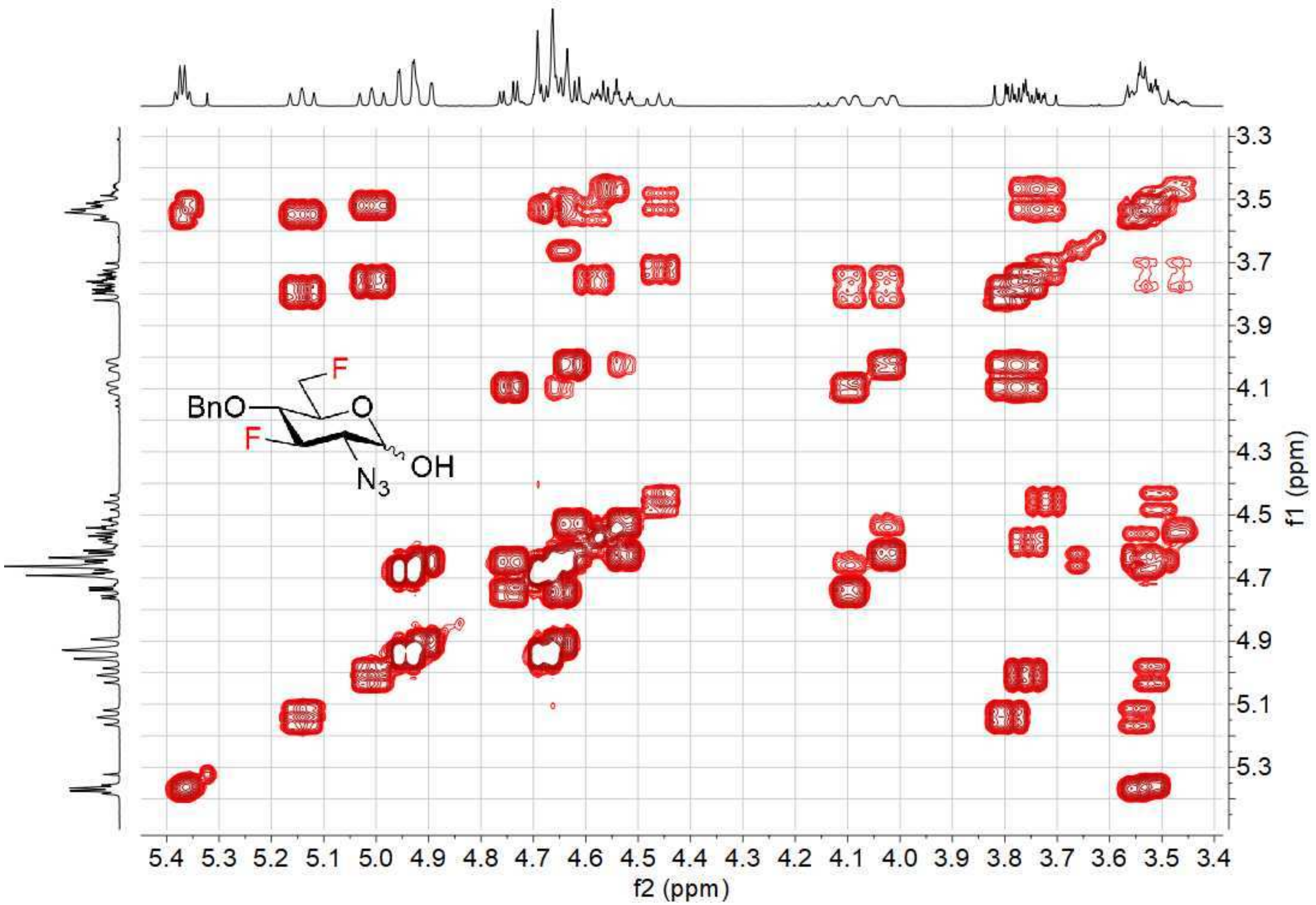
BnO
F
F
O
OH
N3
5.4 5.3 5.2 5.1 5.0 4.9 4.8 4.7 4.6 4.5 4.4 4.3 4.2 4.1 4.0 3.9 3.8 3.7 3.6 3.5 3.4
f2 (ppm)
3.3
3.5
3.7
3.9
4.1
4.3
4.5
4.7
4.9
5.1
5.3
f1 (ppm)

^{1}H-^{13}C HMBC 37

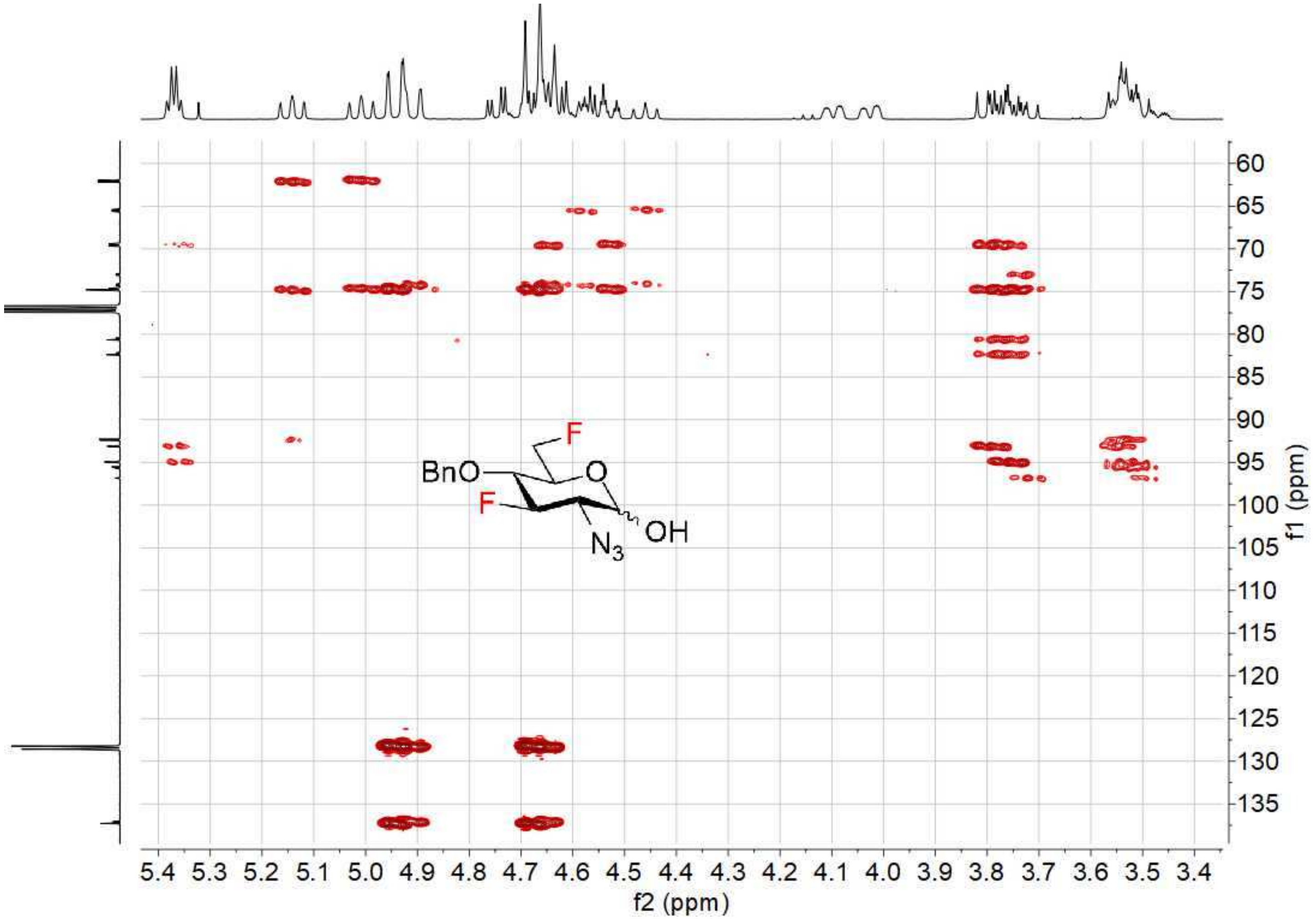

^{1}H-^{13}C HSQC 37

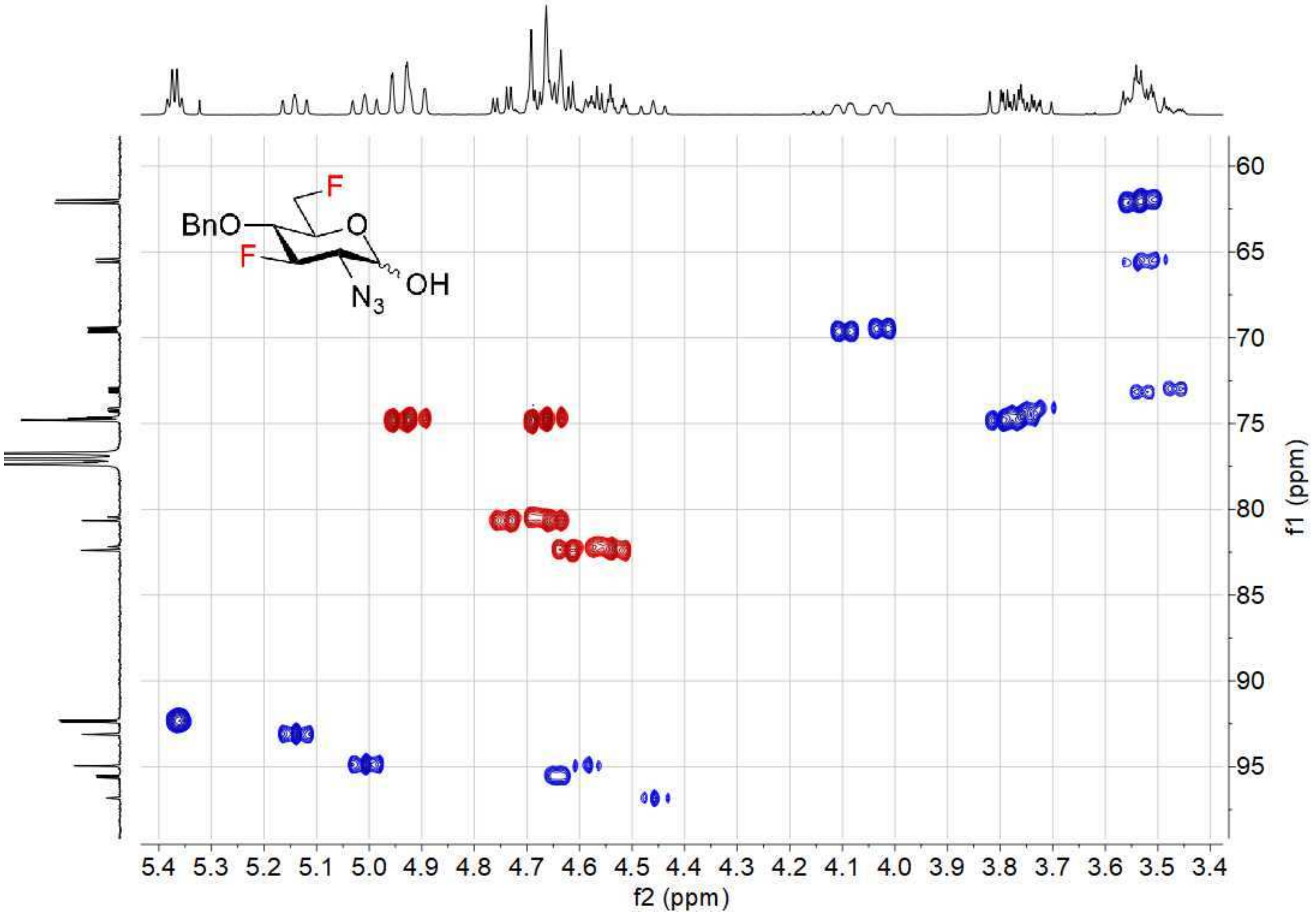

NMR COMPOUND 38

^{1}H NMR (400 MHz, $CDCl_3$) 38

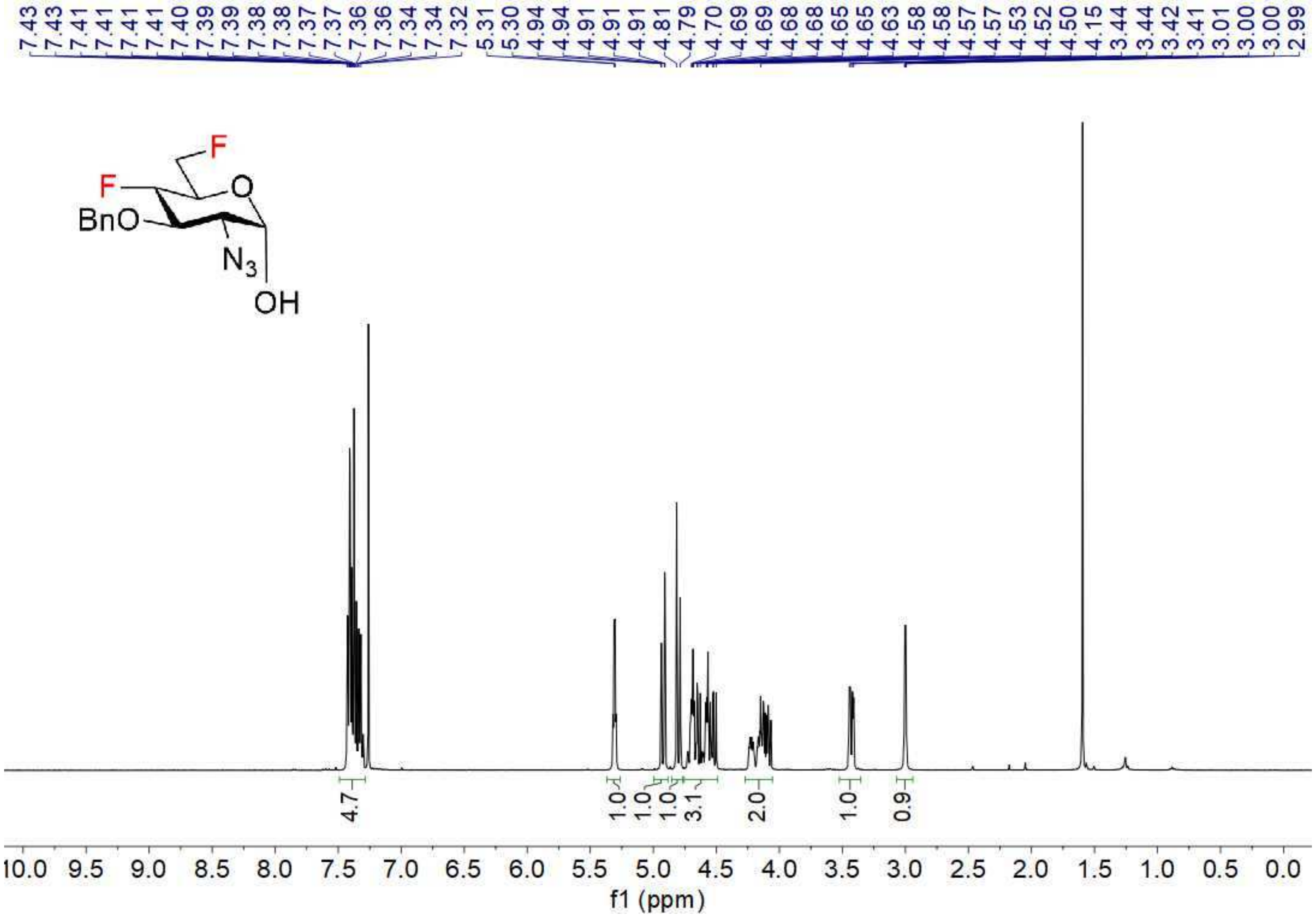

^{13}C NMR (100 MHz, CDCl$_3$) 38

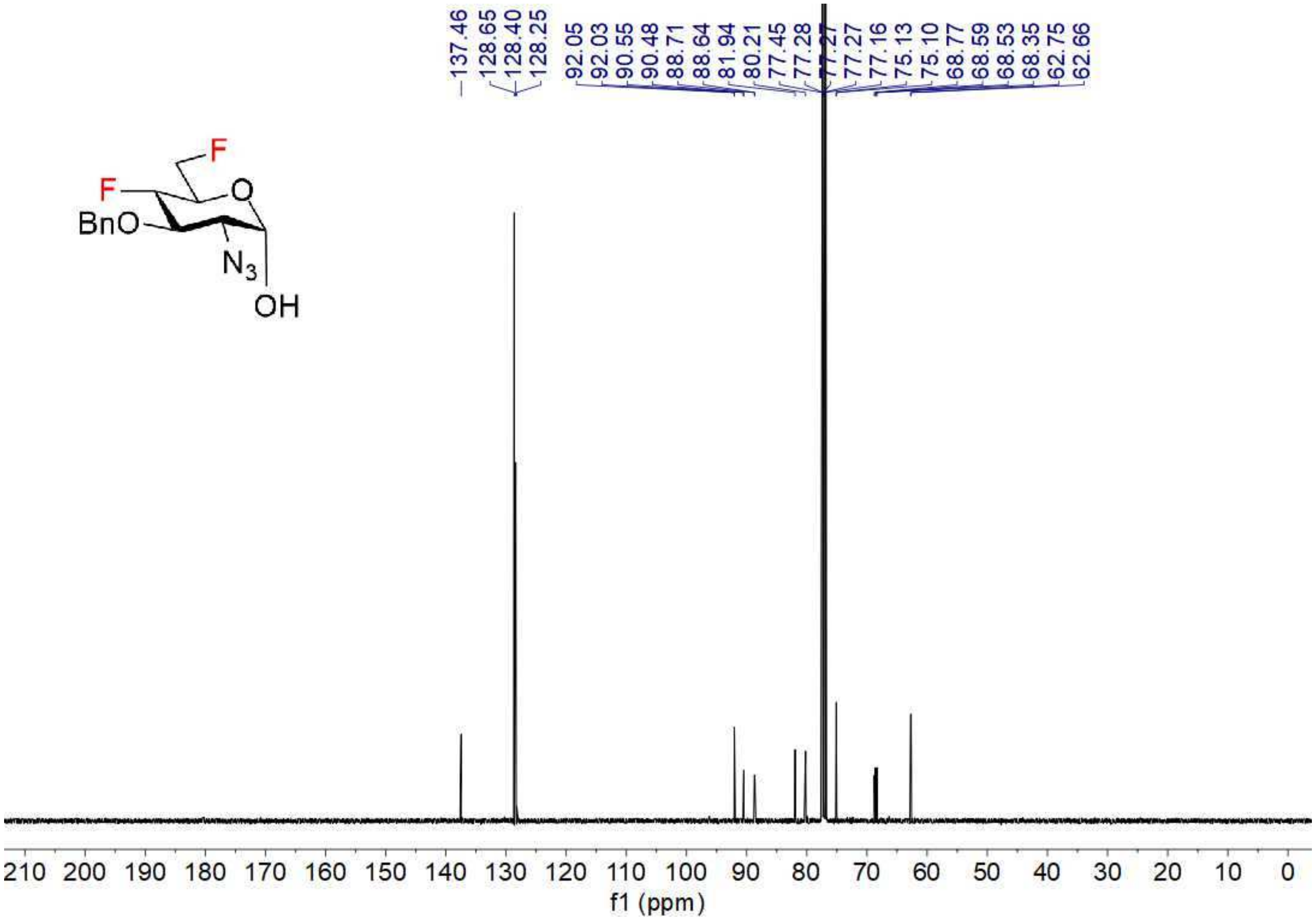

^{19}F NMR (376 MHz, CDCl$_3$) 38

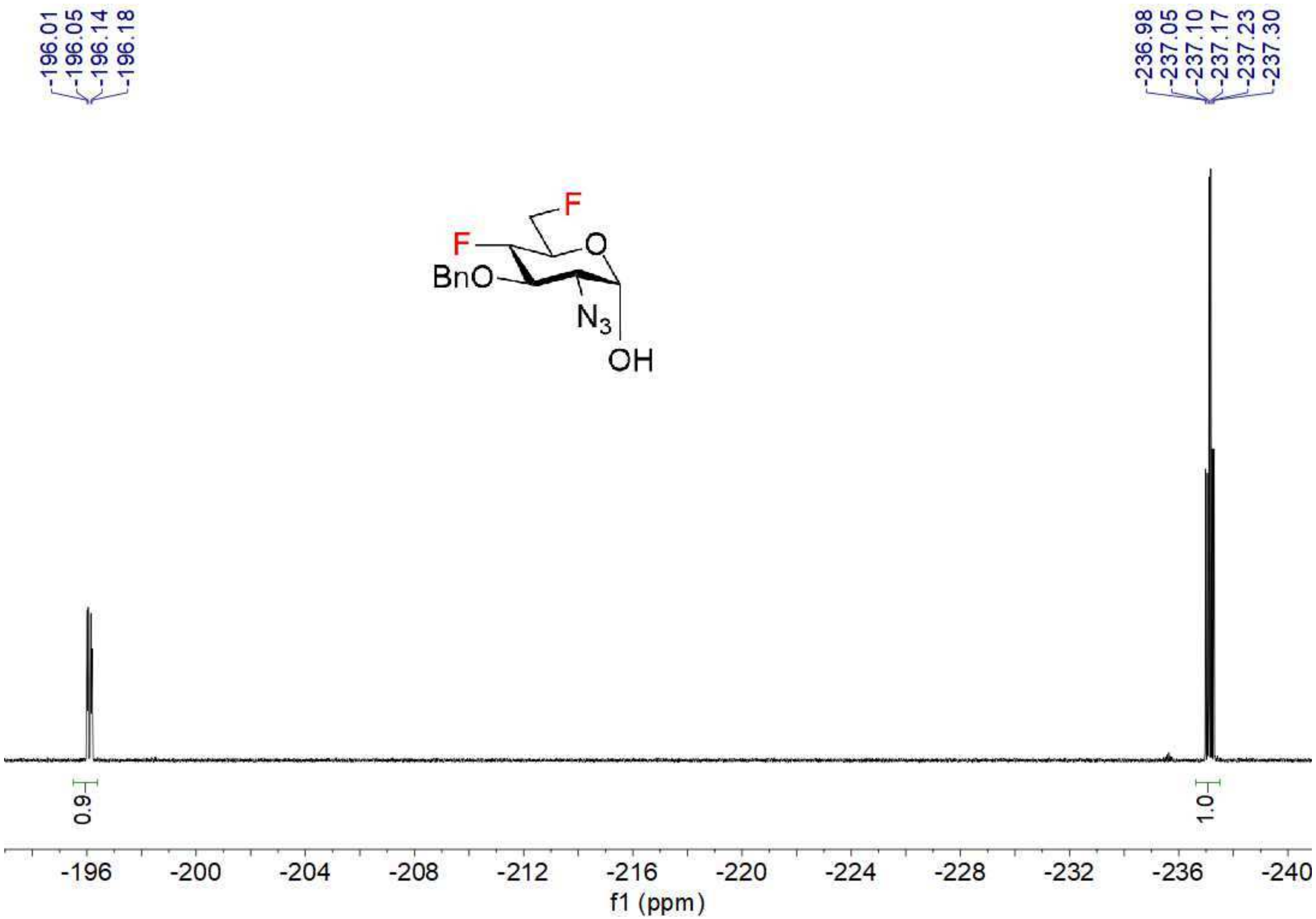

^{1}H-^{1}H COSY 38

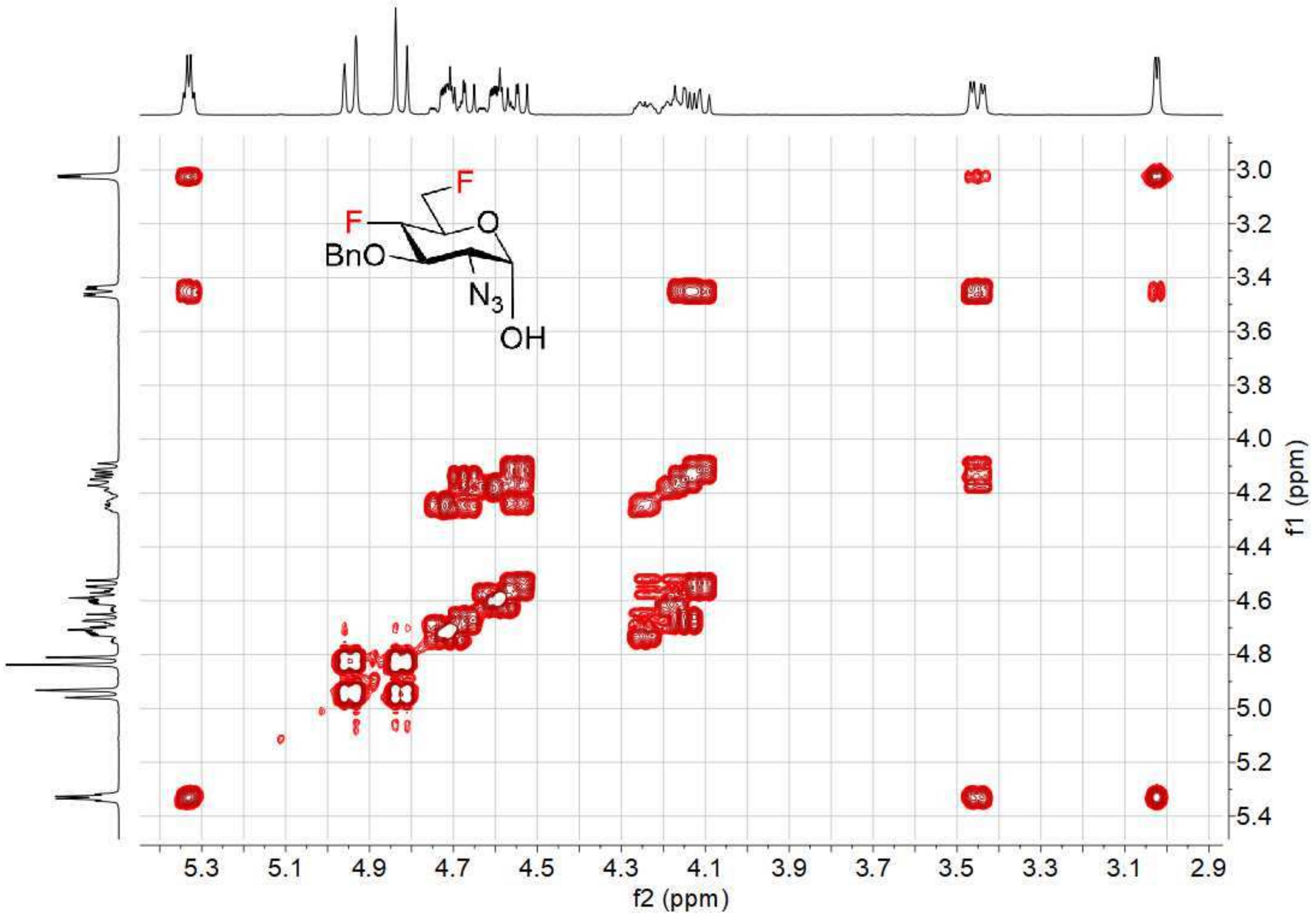
F
F
O
BnO
N3
OH
5.3
5.1
4.9
4.7
4.5
4.3
4.1
3.9
3.7
3.5
3.3
3.1
2.9
f2 (ppm)
3.0
3.2
3.4
3.6
3.8
4.0
4.2
4.4
4.6
4.8
5.0
5.2
5.4
f1 (ppm)

^{1}H-^{13}C HSQC 38

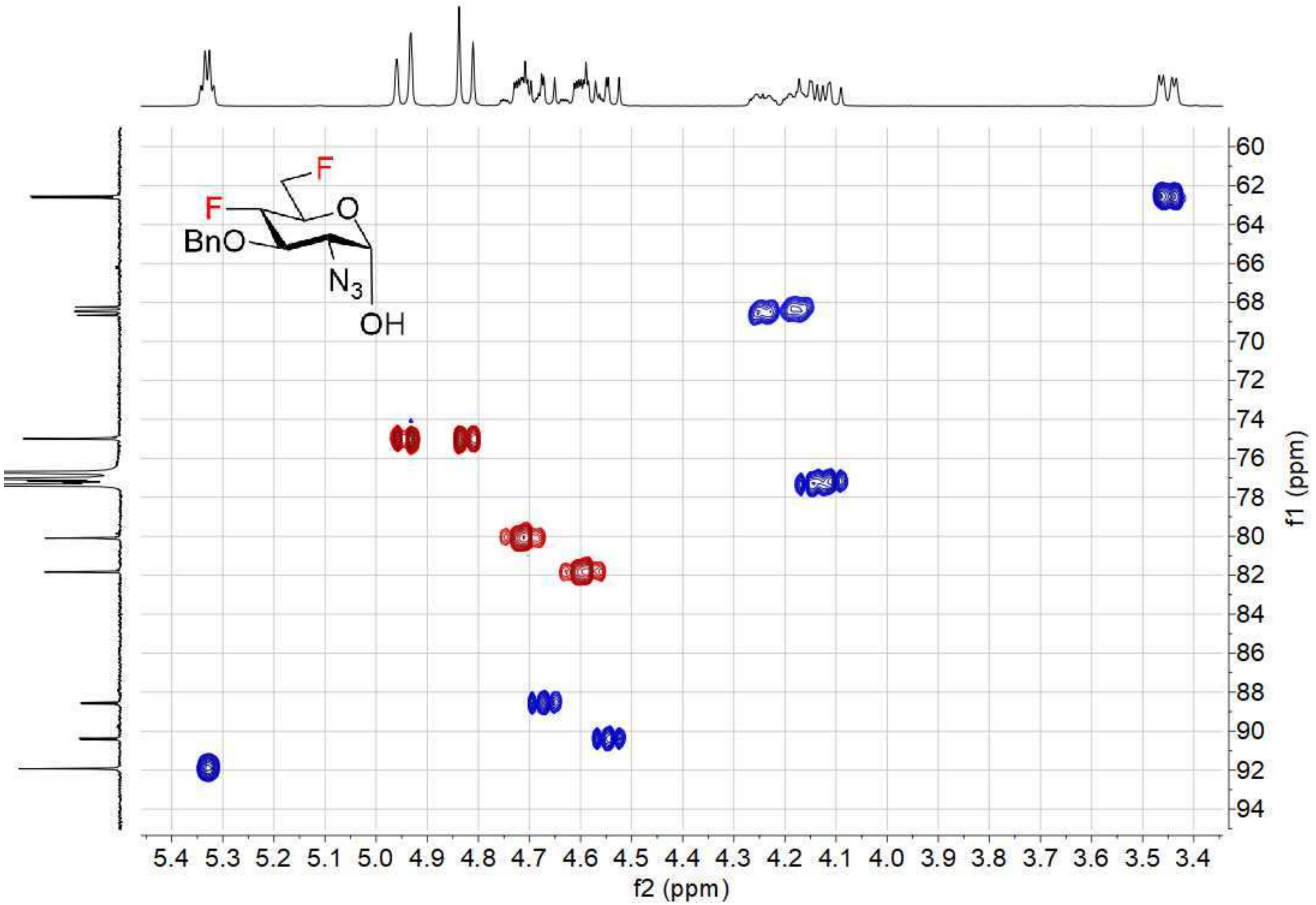

NMR COMPOUND 39

^{1}H NMR (400 MHz, CDCl$_3$) 39 (ca. 40% β-anomer)

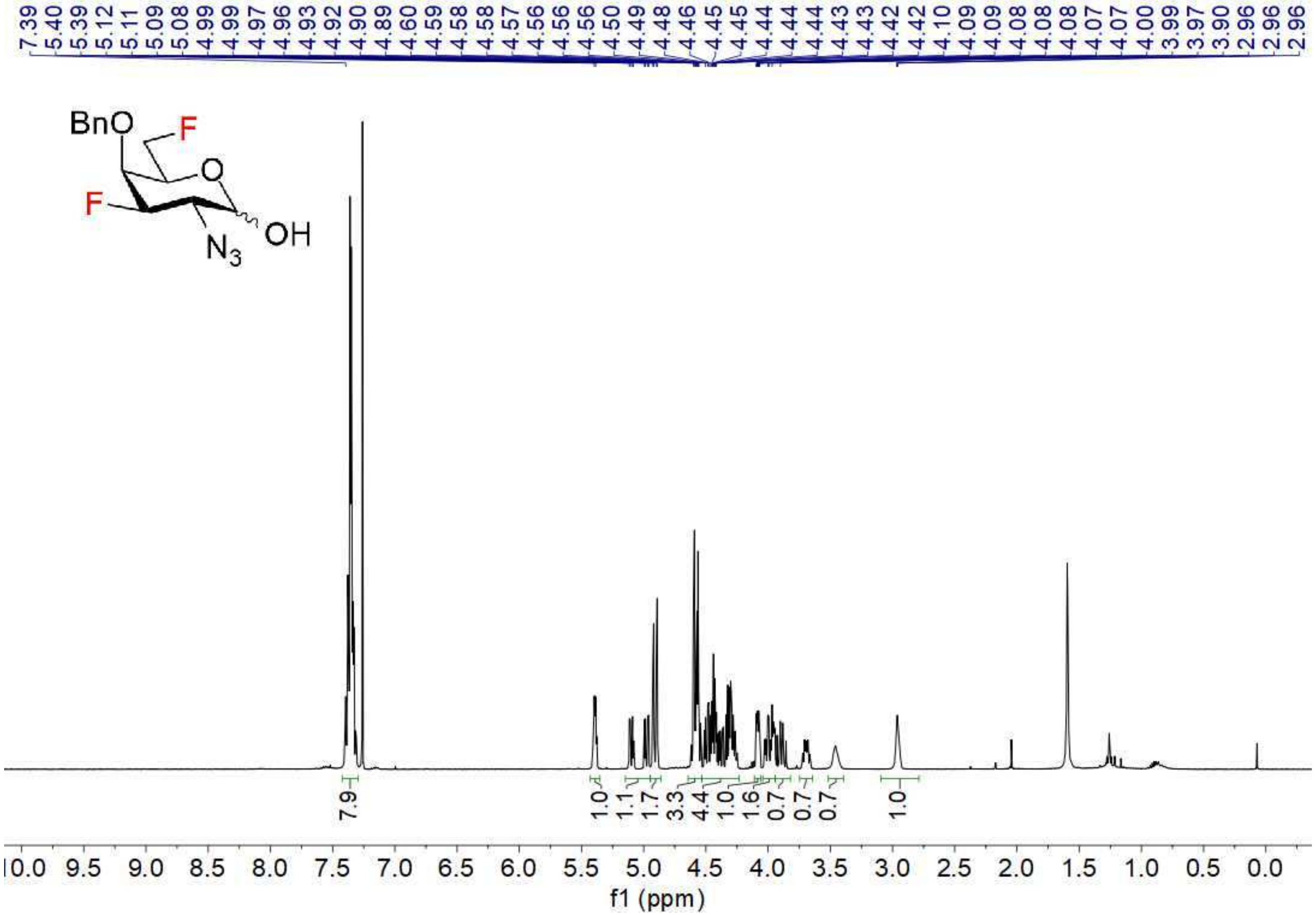

^{13}C NMR (100 MHz, CDCl$_3$) 39

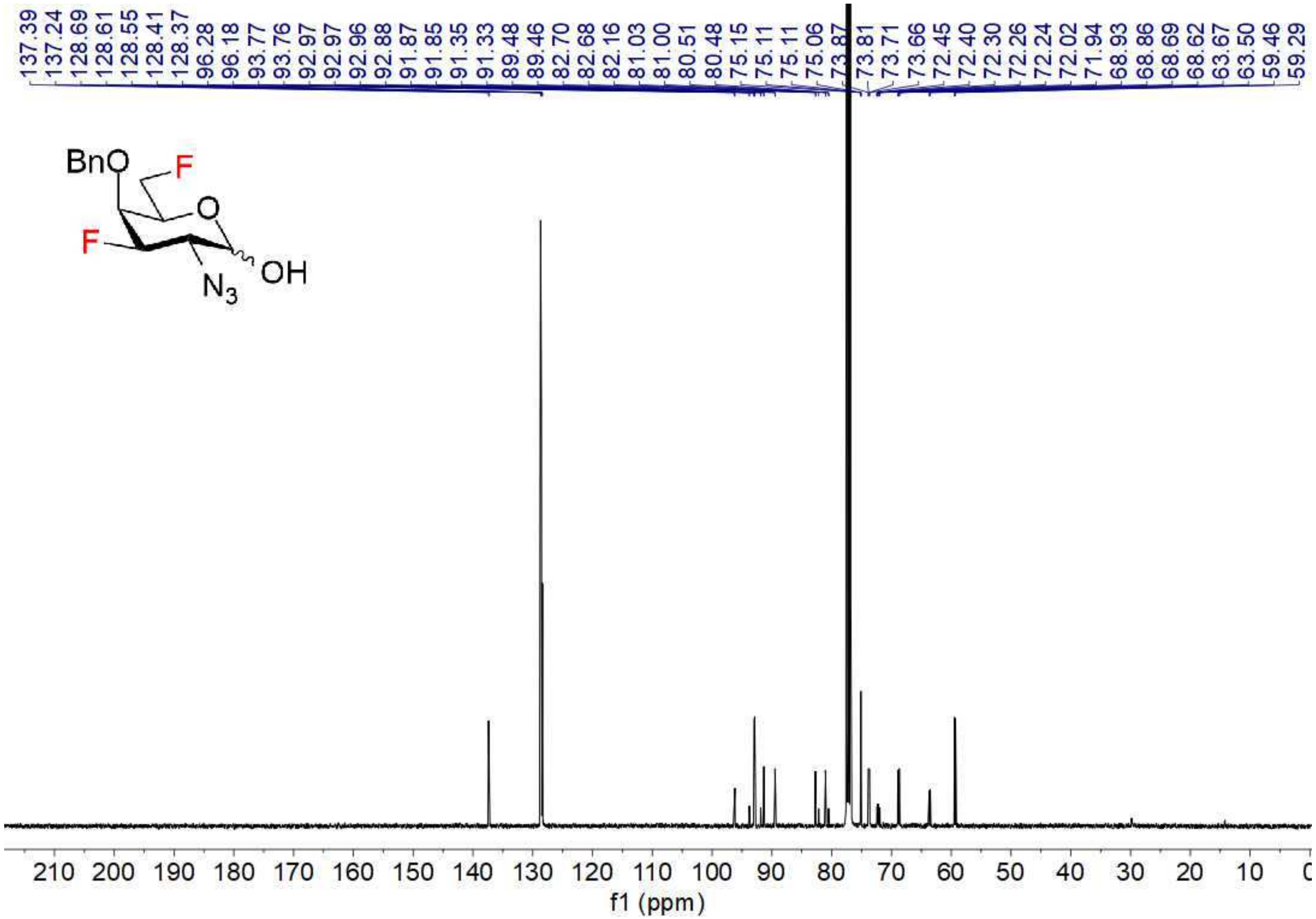

^{19}F NMR (376 MHz, $CDCl_3$) 39 (ca. 40% β-anomer)

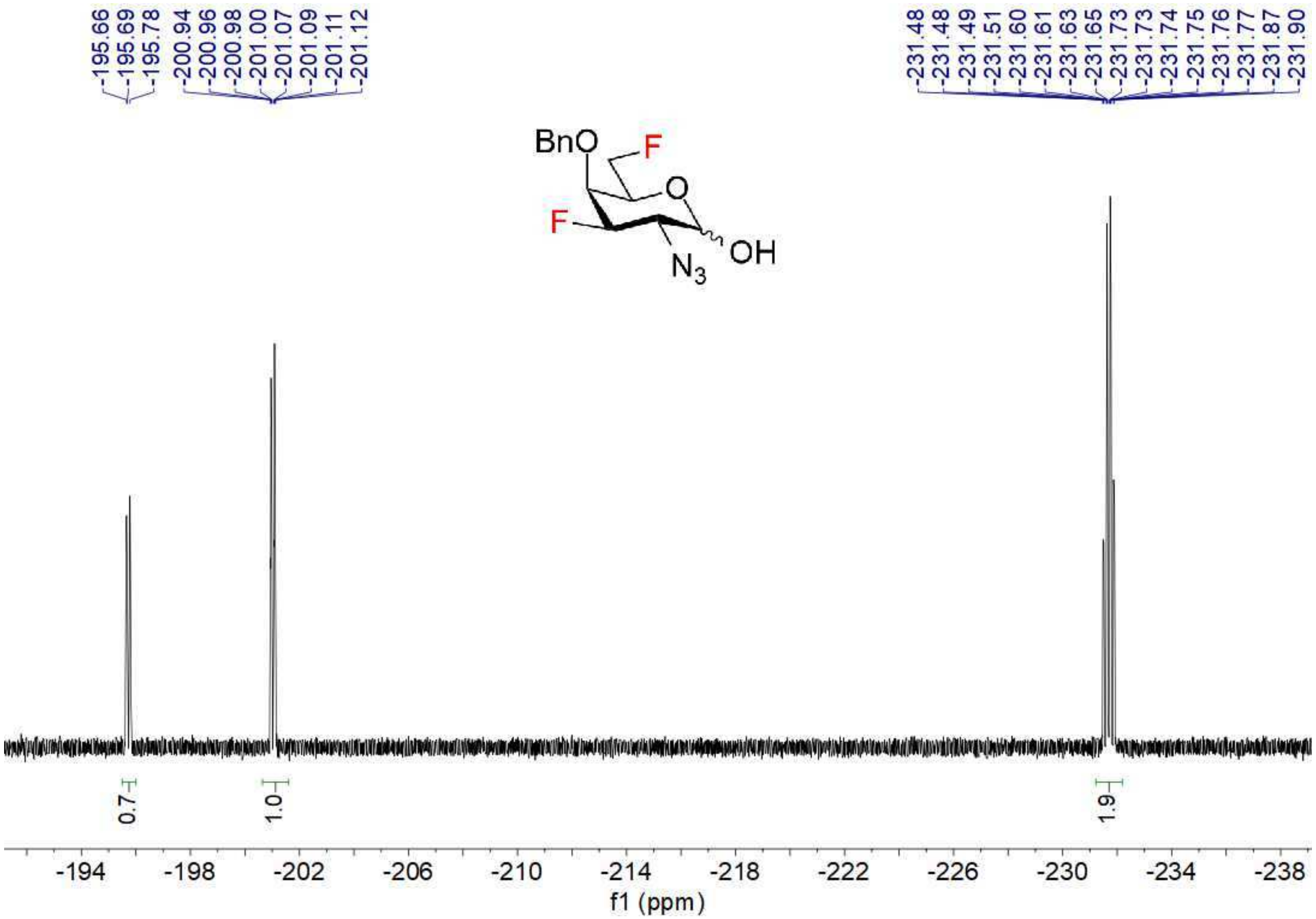

^{1}H-^{1}H COSY 39

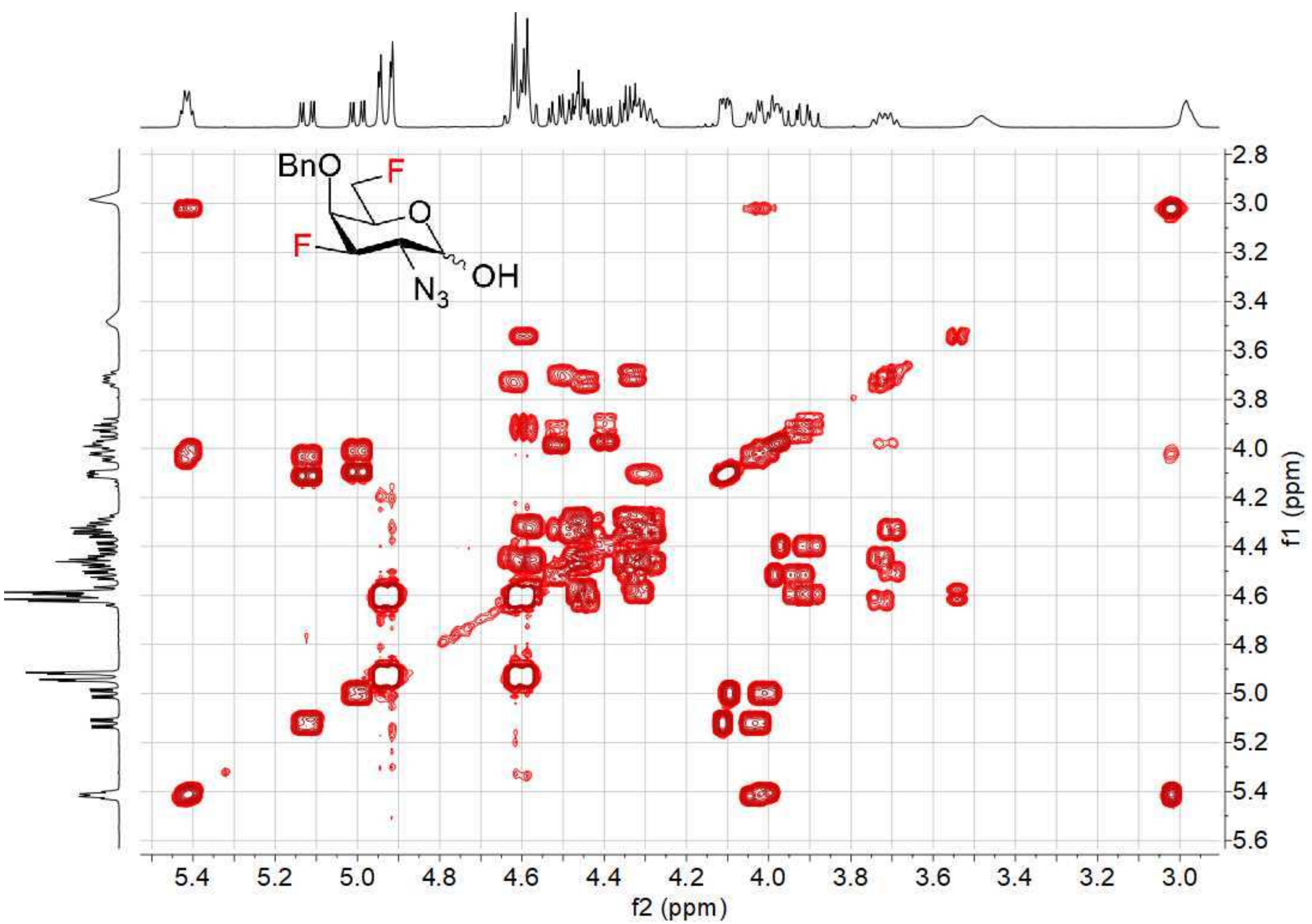

^{1}H-^{13}C HMBC 39

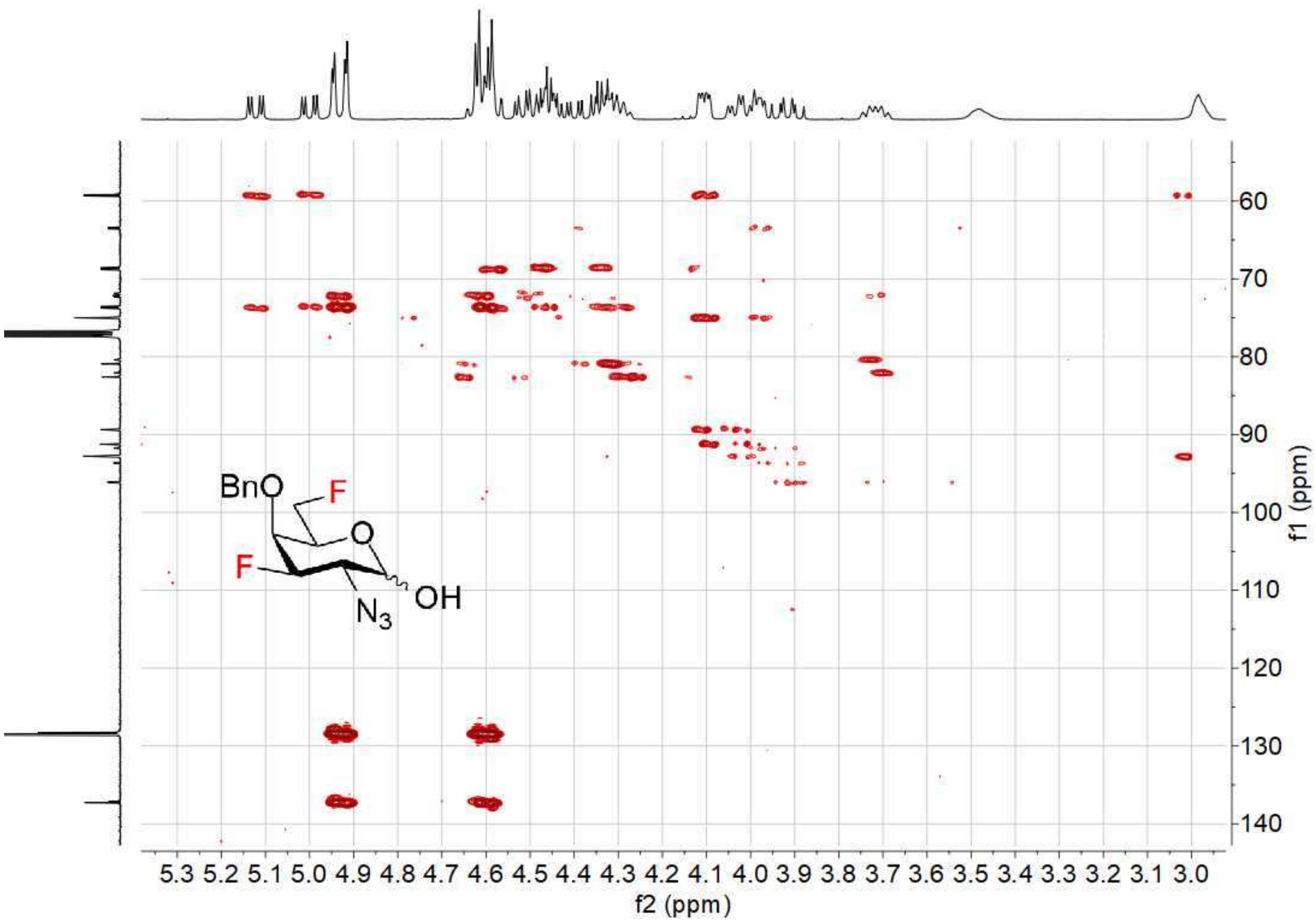

^{1}H-^{13}C HSQC 39

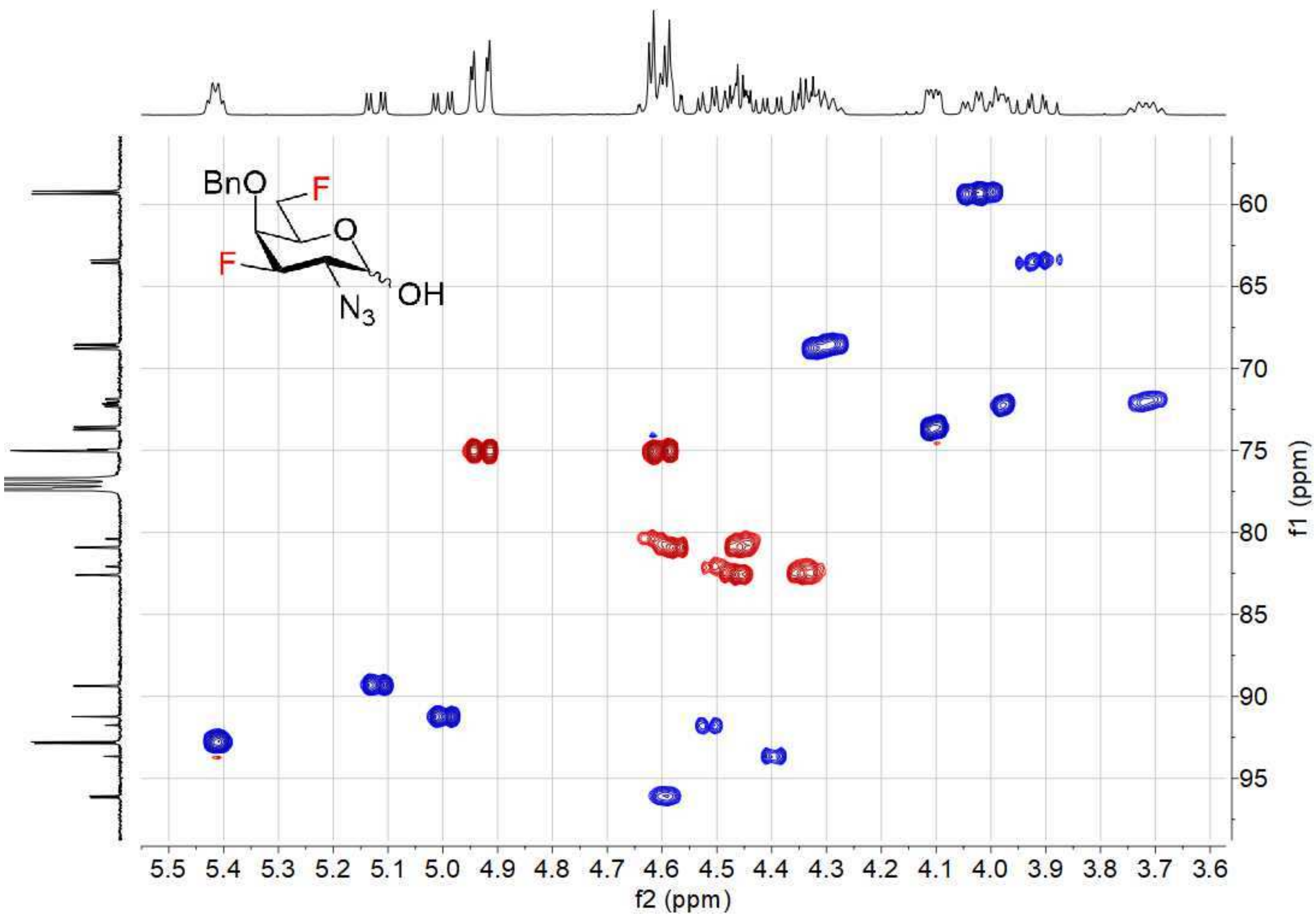

NMR COMPOUND 40

^{1}H NMR (400 MHz, $CDCl_3$) 40 (α/β ca. 10/3)

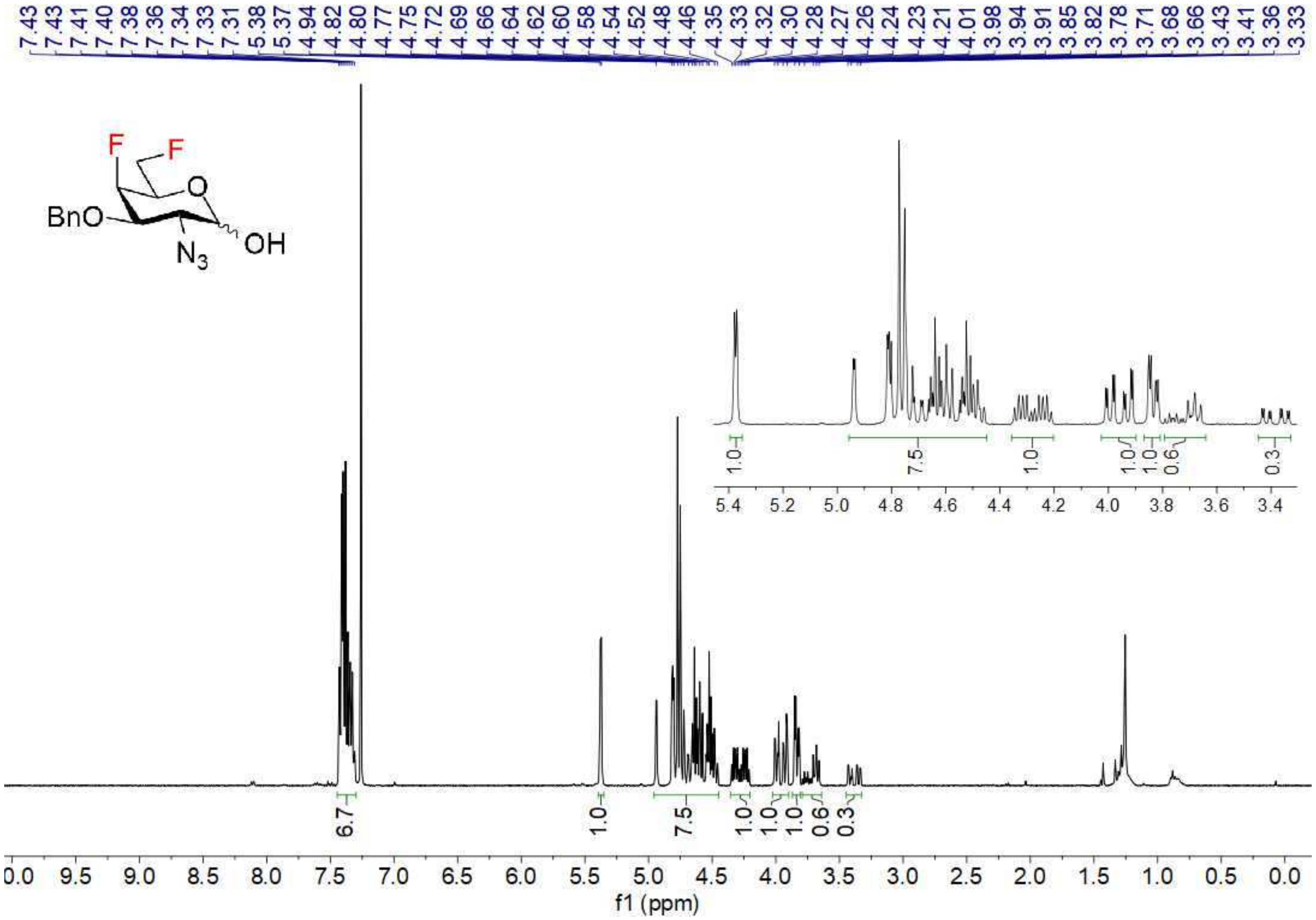

^{13}C NMR (100 MHz, CDCl$_3$) 40

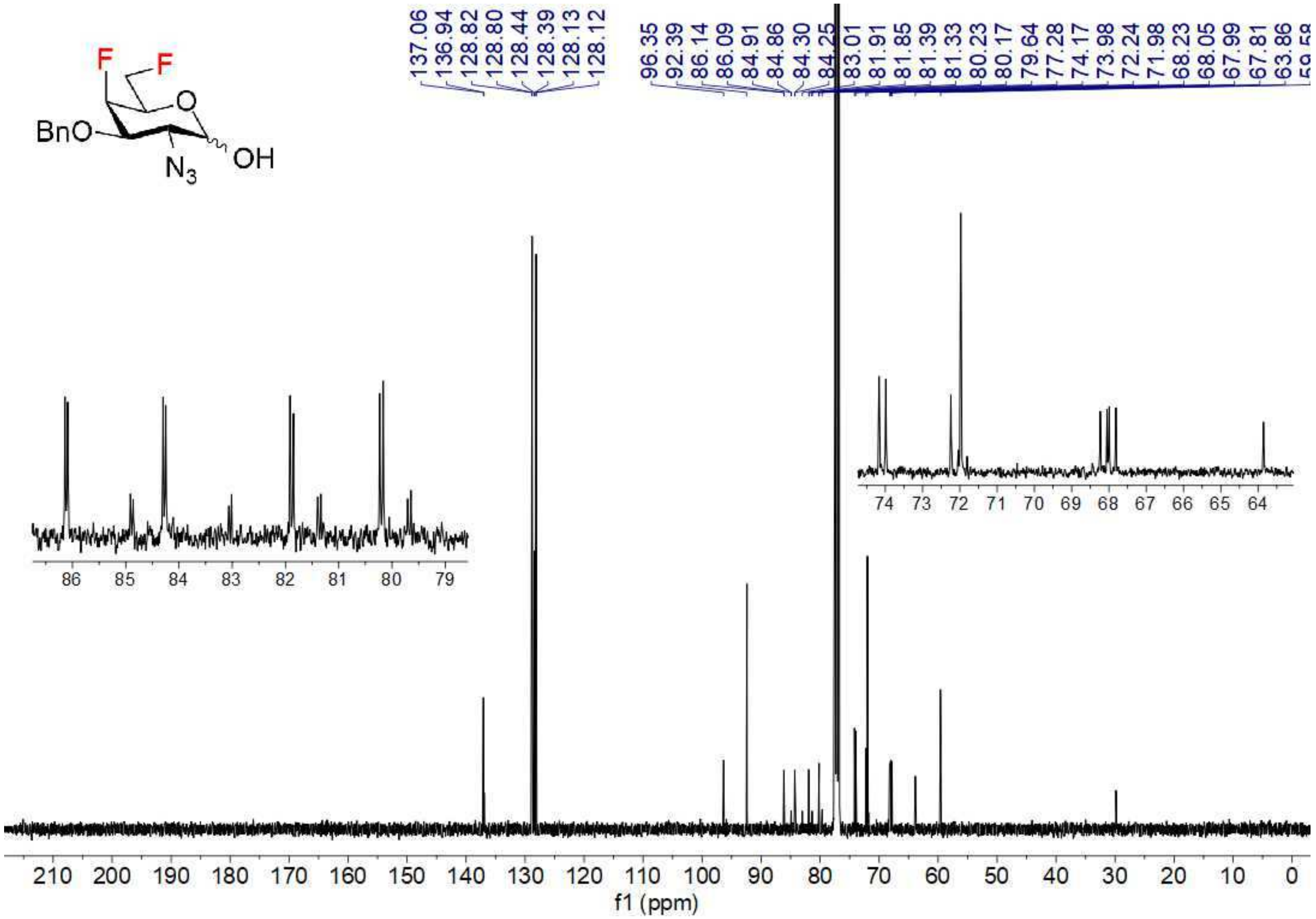

^{19}F NMR (376 MHz, CDCl$_3$) 40 (α/β ca. 10/3)

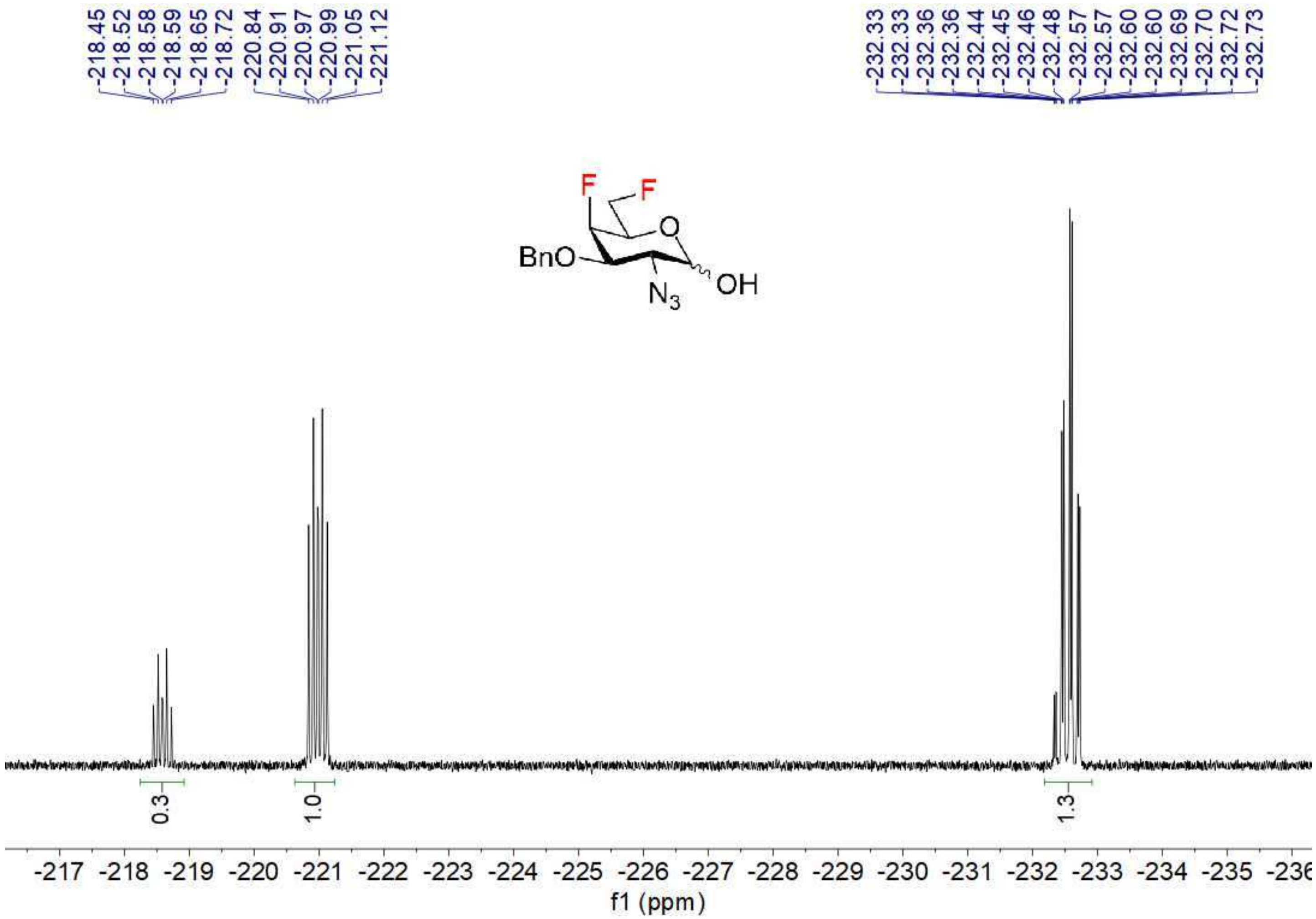

^{1}H-^{1}H COSY 40

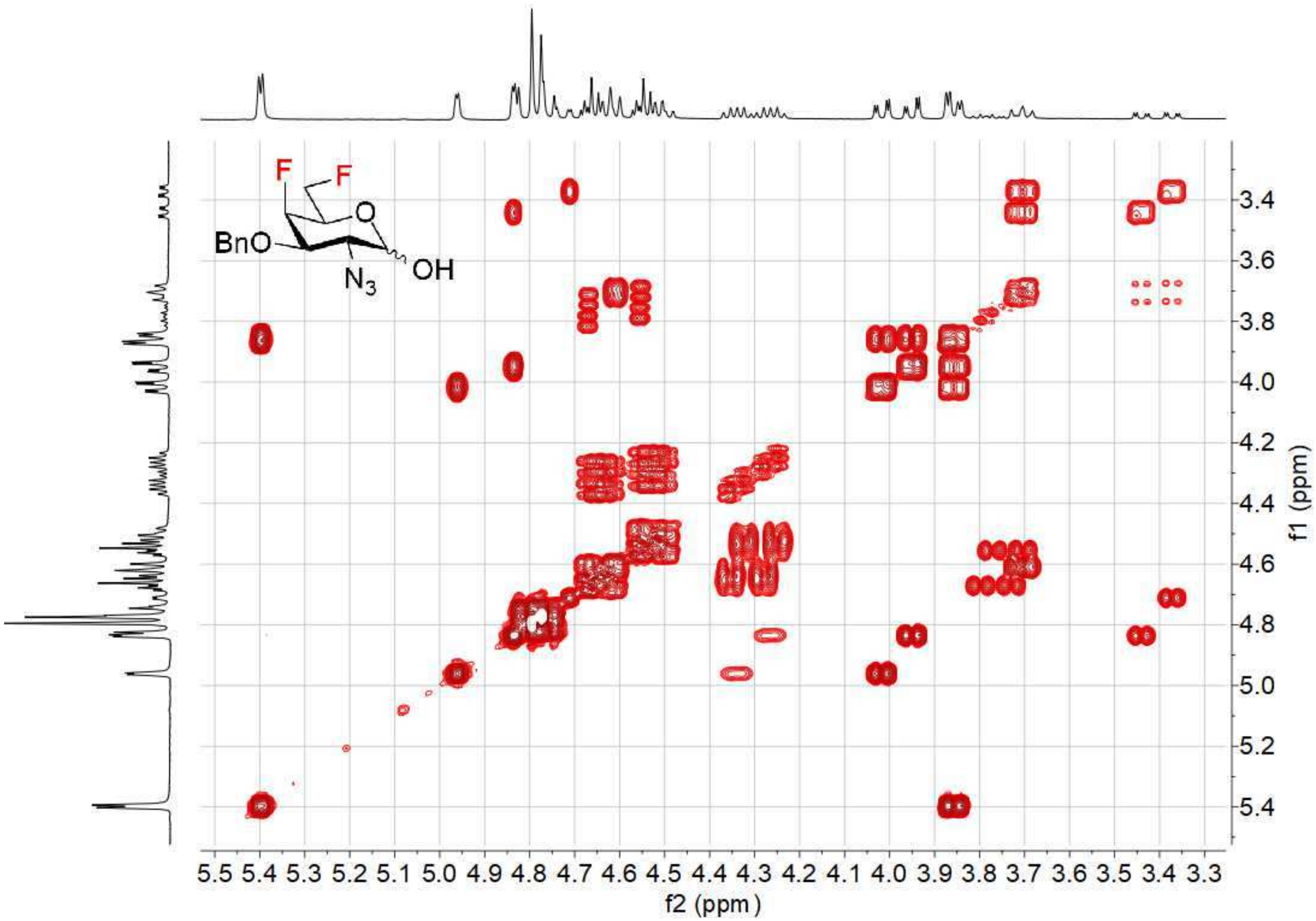
F
F
BnO
O
N3
OH
5.5 5.4 5.3 5.2 5.1 5.0 4.9 4.8 4.7 4.6 4.5 4.4 4.3 4.2 4.1 4.0 3.9 3.8 3.7 3.6 3.5 3.4 3.3
f2 (ppm)
3.4
3.6
3.8
4.0
4.2
4.4
4.6
4.8
5.0
5.2
5.4
f1 (ppm)

^{1}H-^{13}C HSQC 40

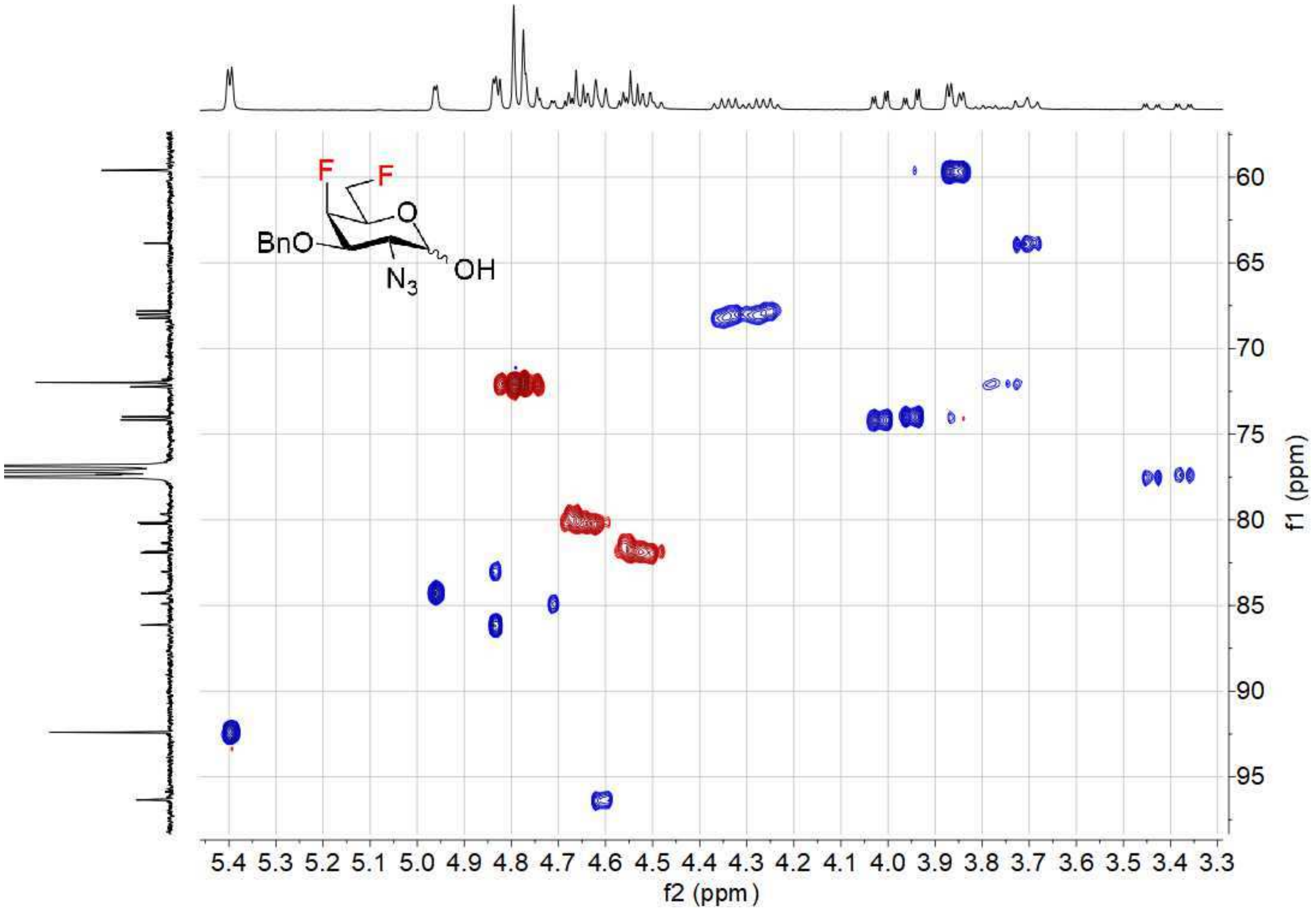

NMR COMPOUND 41

^{1}H NMR (400 MHz, $CDCl_3$) 41 (α/β ca. 2/1)

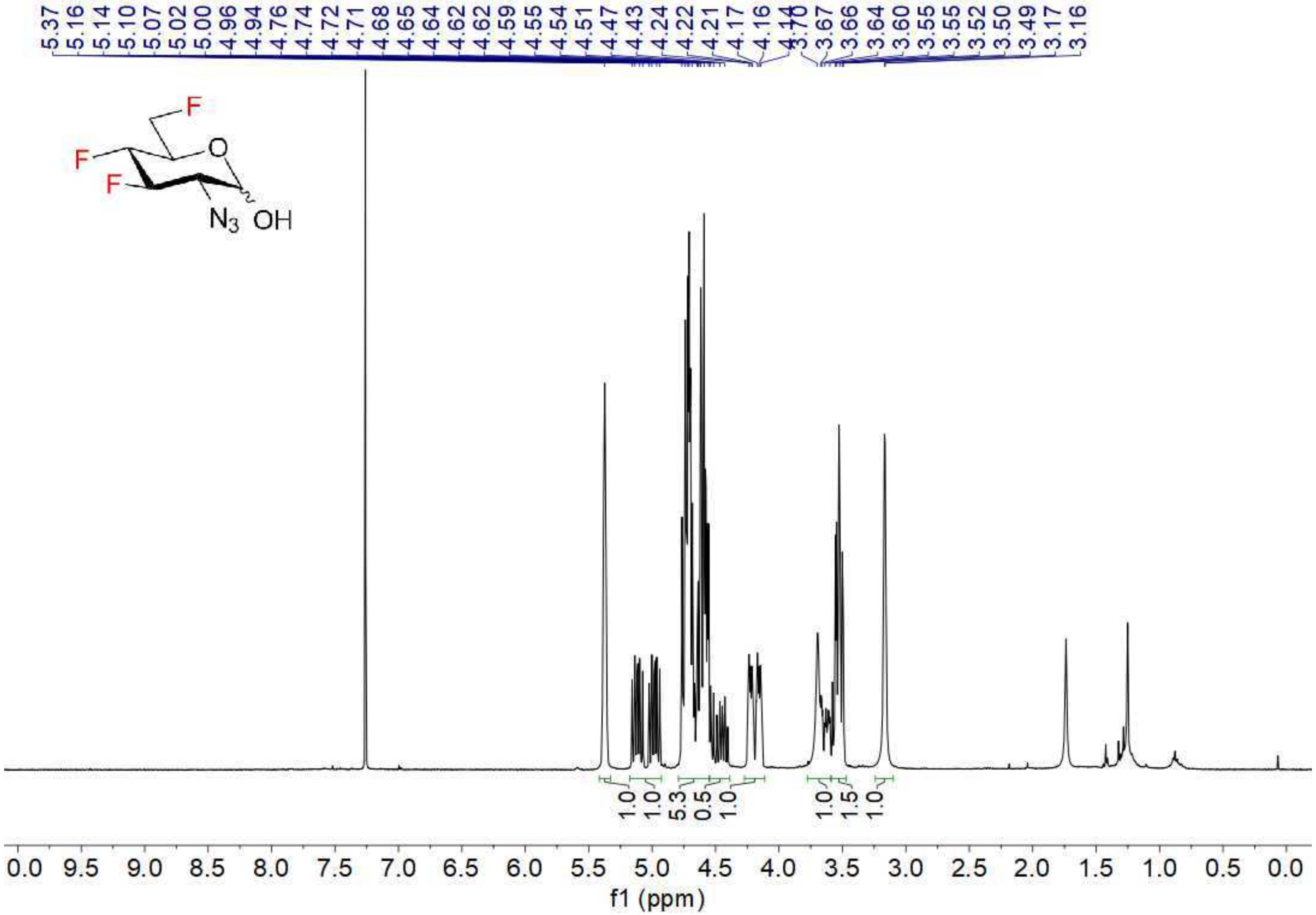

^{13}C NMR (100 MHz, CDCl$_3$) 41

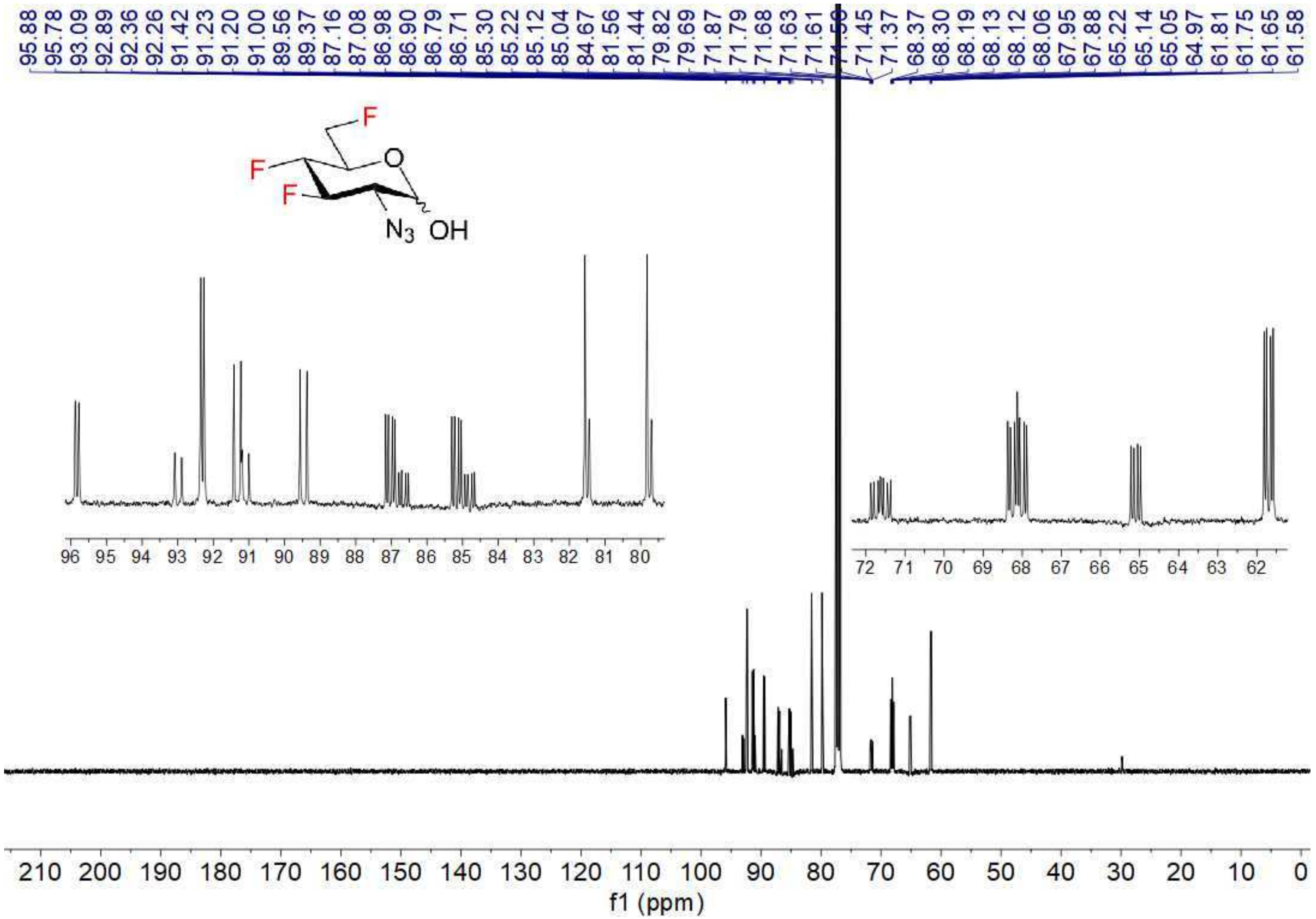

^{19}F NMR (376 MHz, CDCl$_3$) 41 (α/β ca. 2/1)

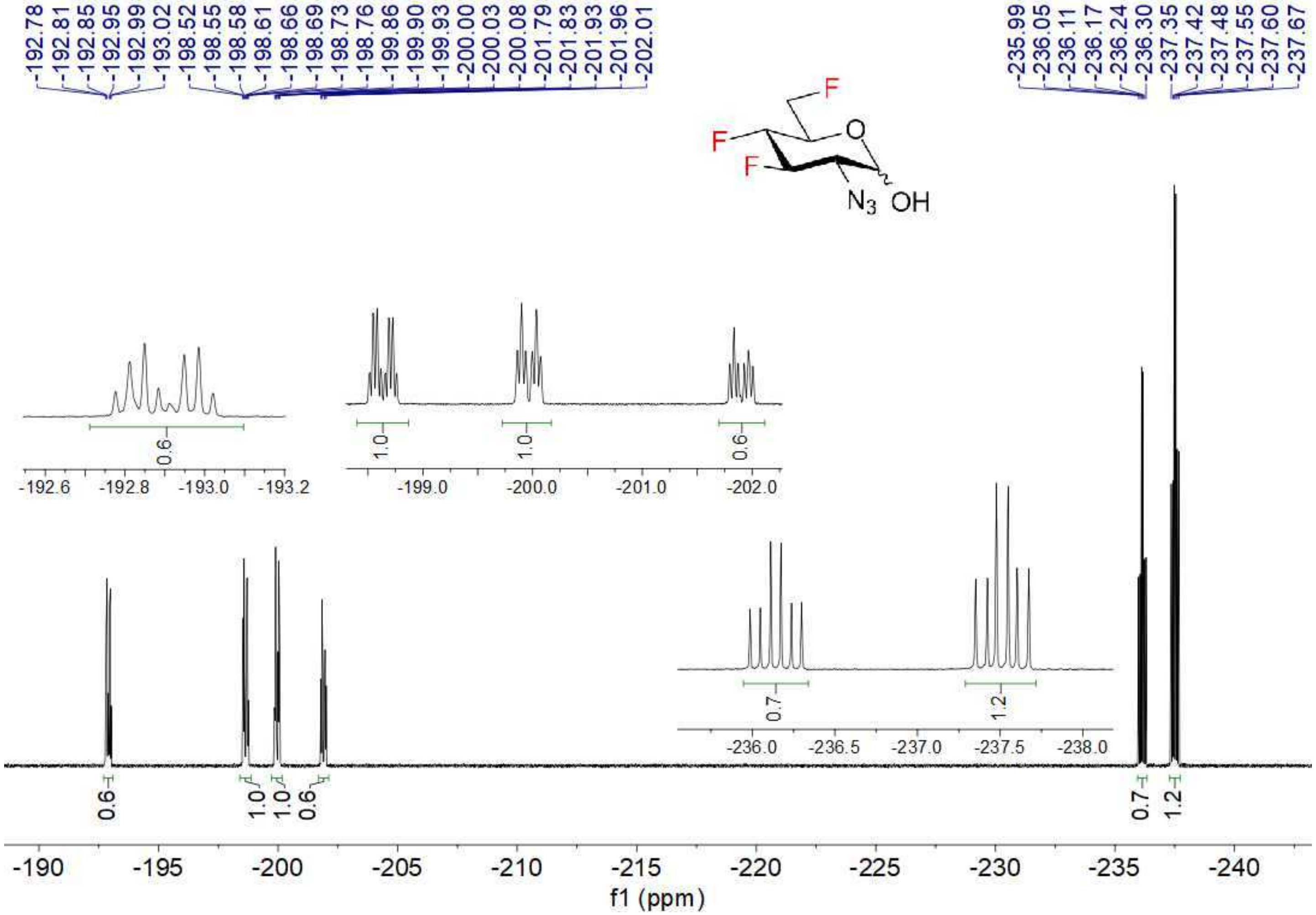

^{1}H-^{1}H COSY 41

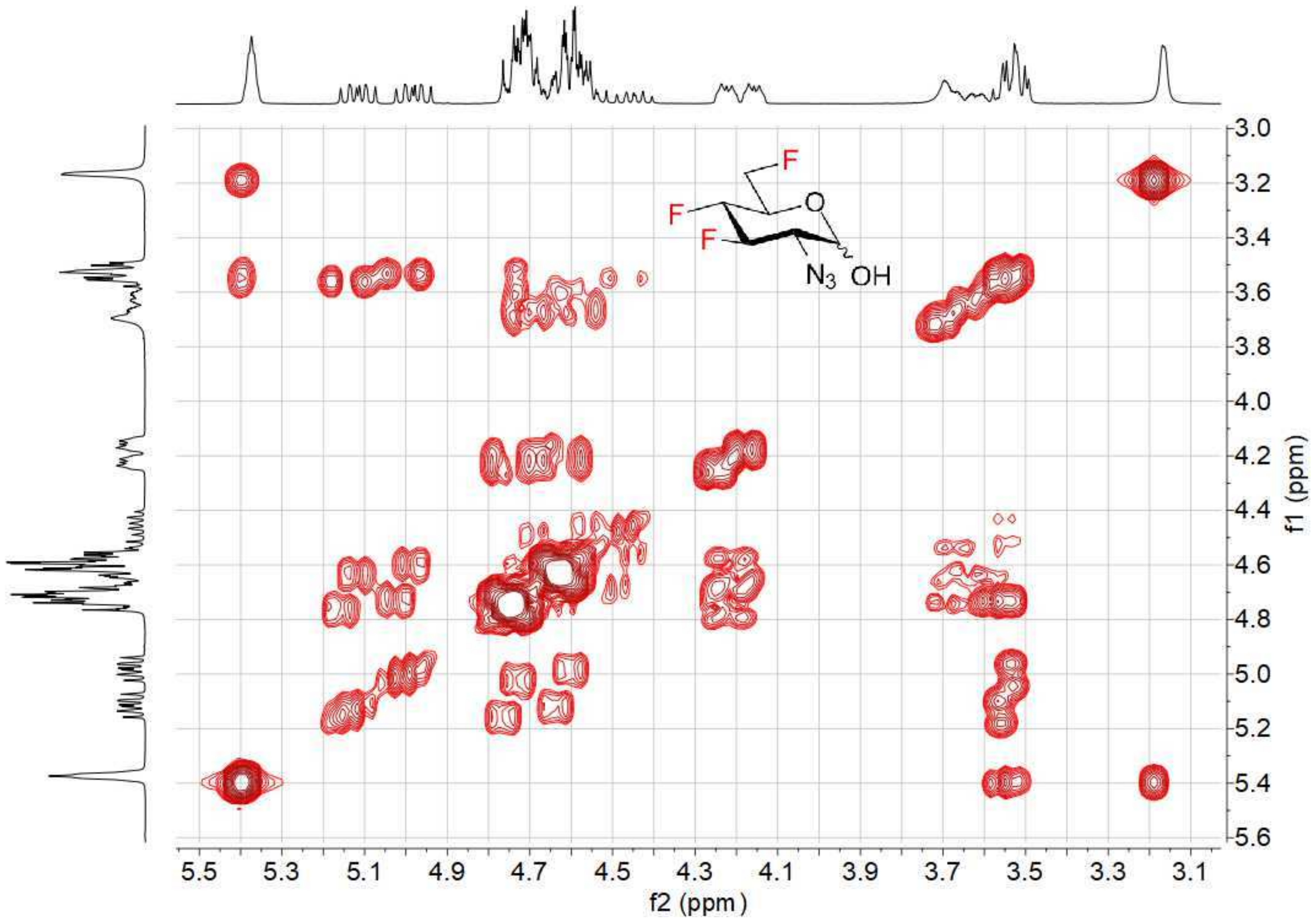
F
F
F
O
N_3 OH
3.0
3.2
3.4
3.6
3.8
4.0
4.2
4.4
4.6
4.8
5.0
5.2
5.4
5.6
f1 (ppm)
5.5 5.3 5.1 4.9 4.7 4.5 4.3 4.1 3.9 3.7 3.5 3.3 3.1
f2 (ppm)

^{1}H-^{13}C HMBC 41

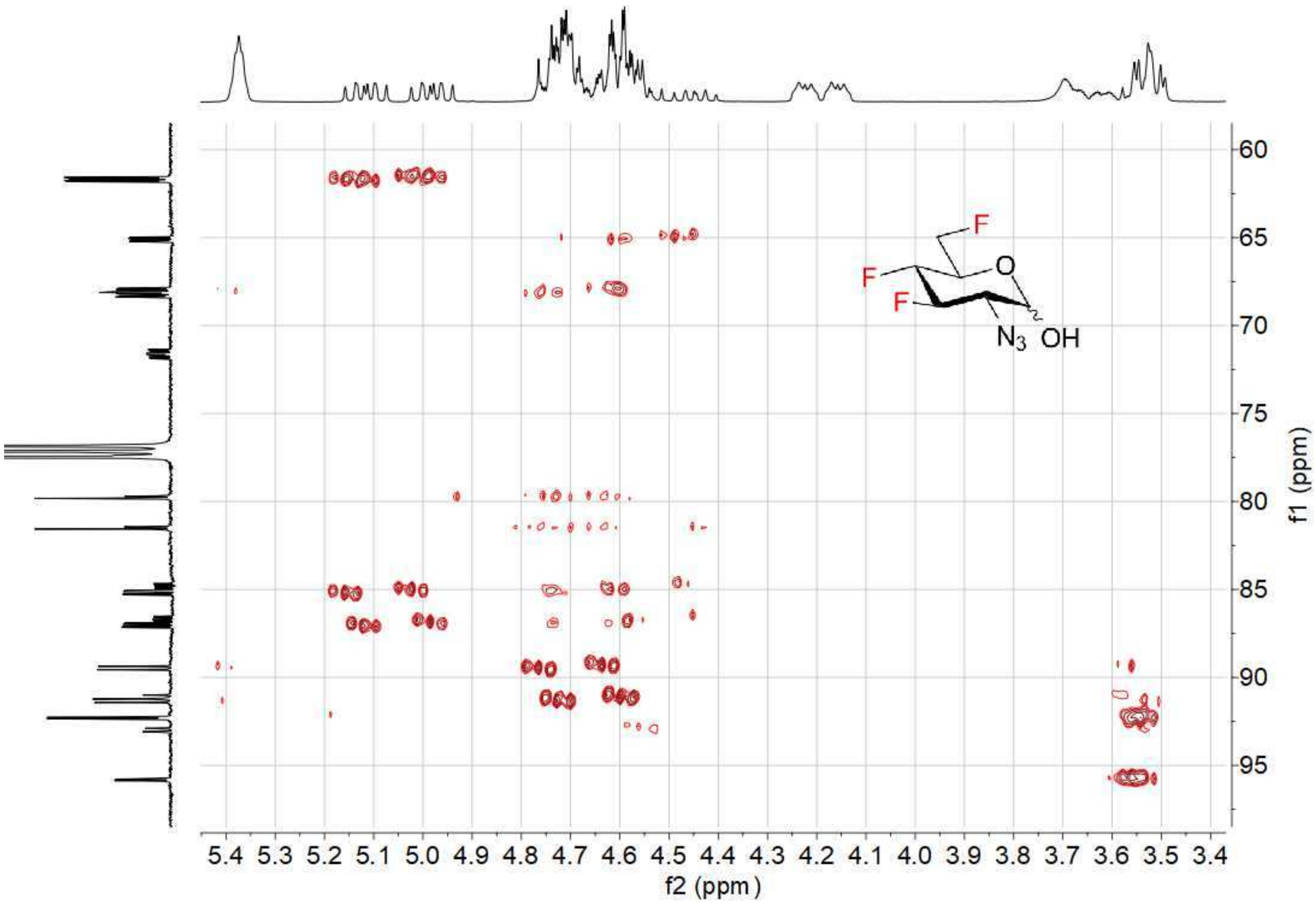

^{1}H-^{13}C HSQC 41

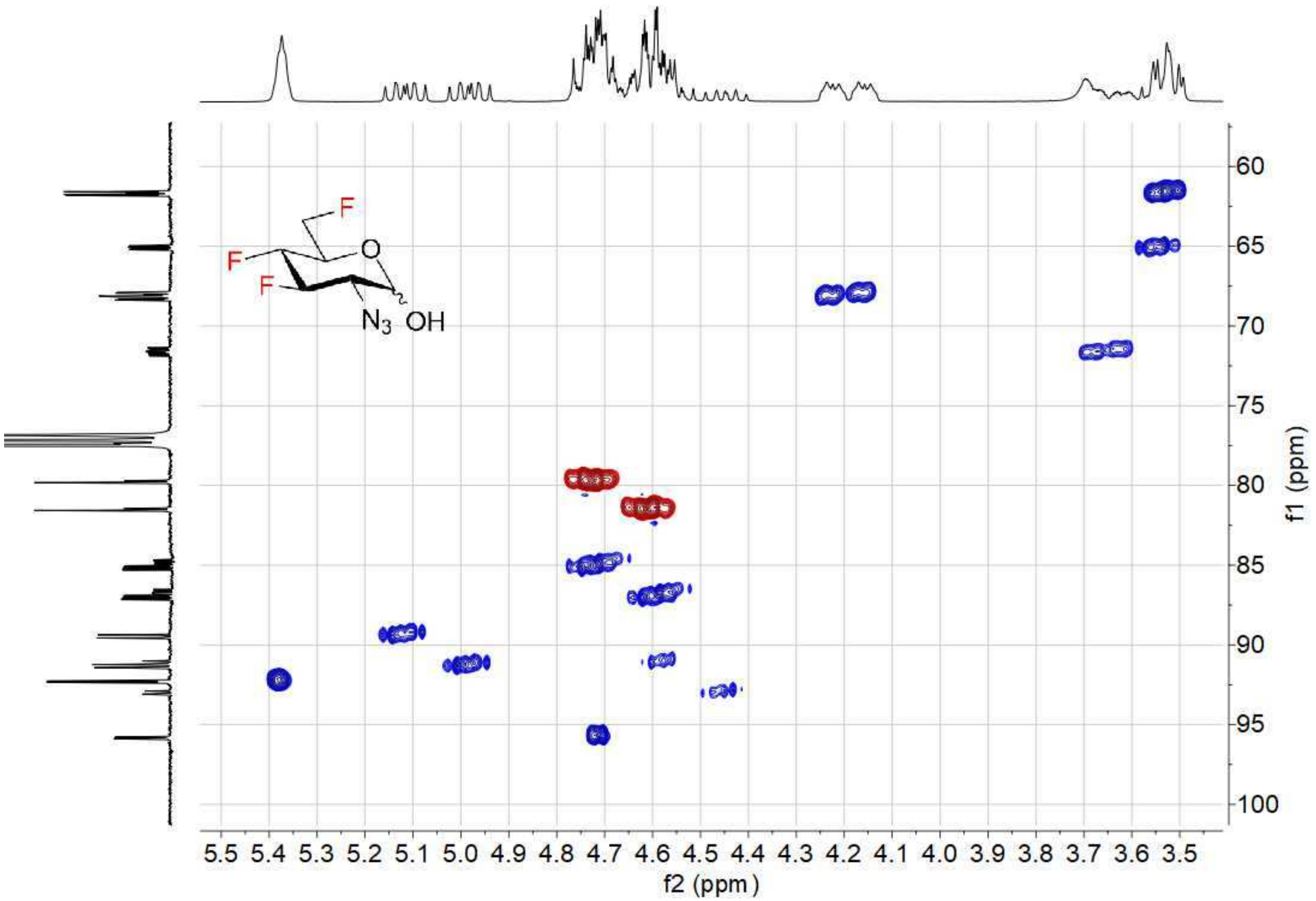

NMR COMPOUND 42

^{1}H NMR (400 MHz, CDCl$_3$) 42 (α/β ca. 10/3)

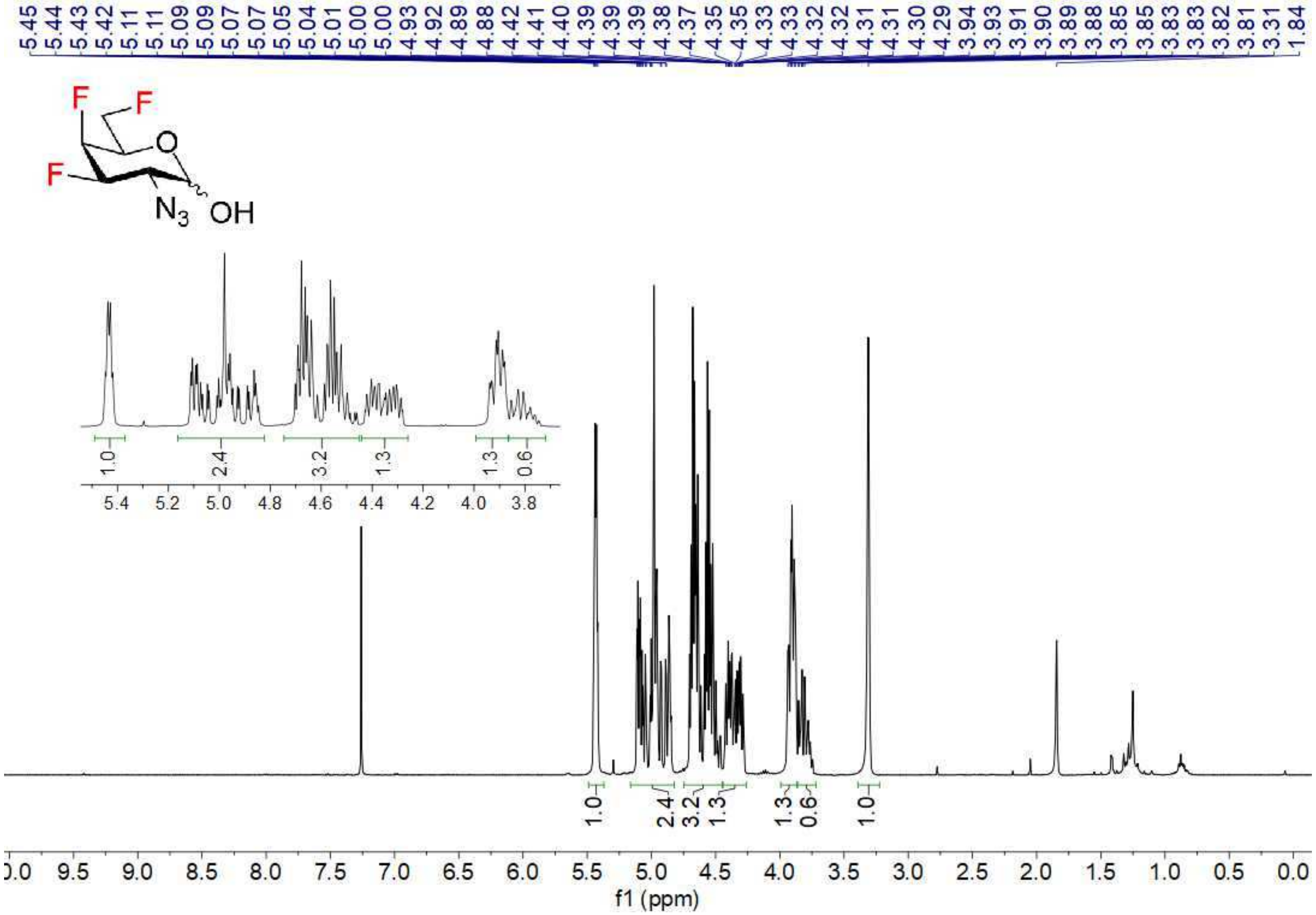

^{13}C NMR (100 MHz, $CDCl_3$) 42

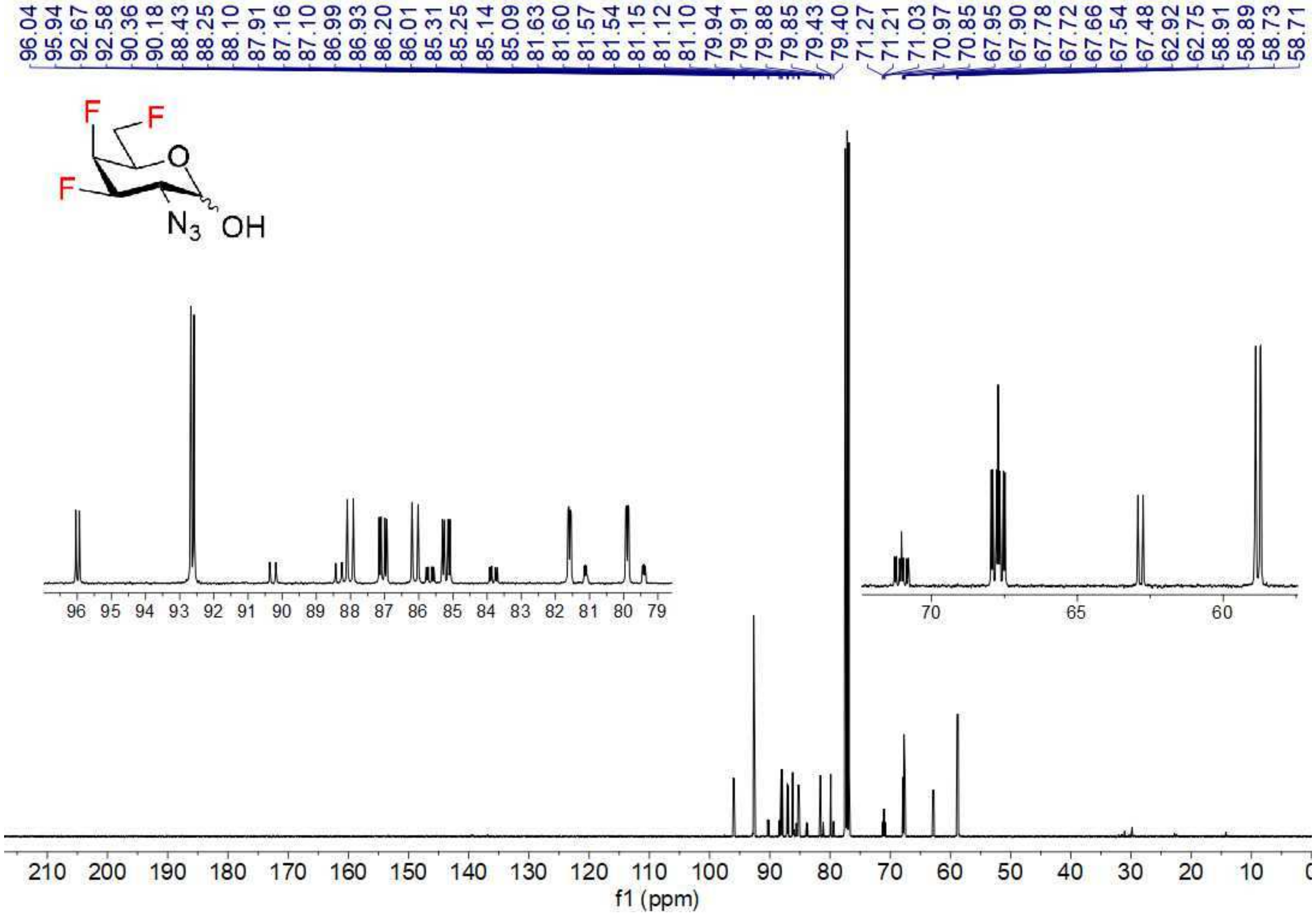

^{19}F NMR (376 MHz, CDCl$_3$) 42 (α/β ca. 10/3)

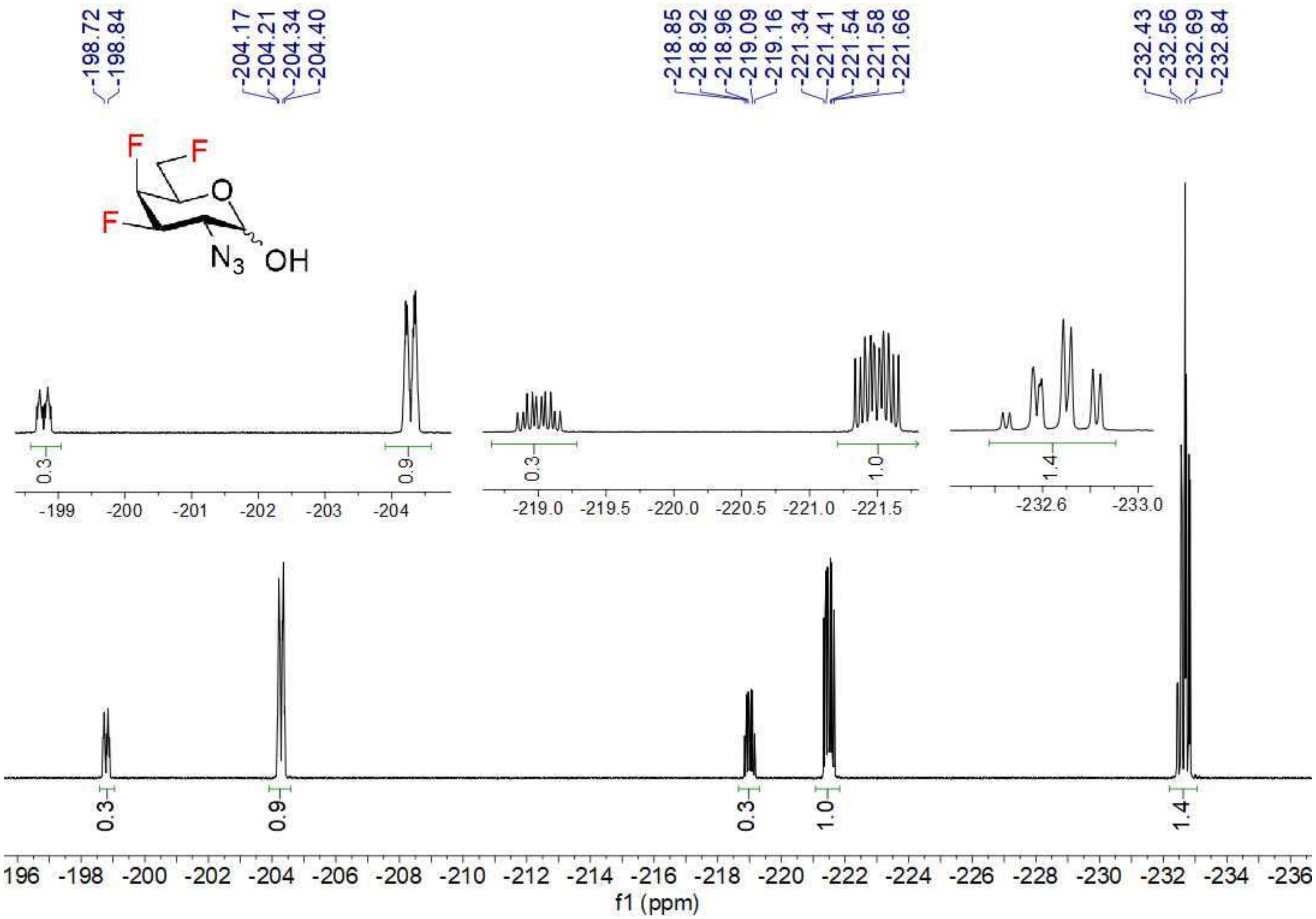

^{1}H-^{1}H COSY 42

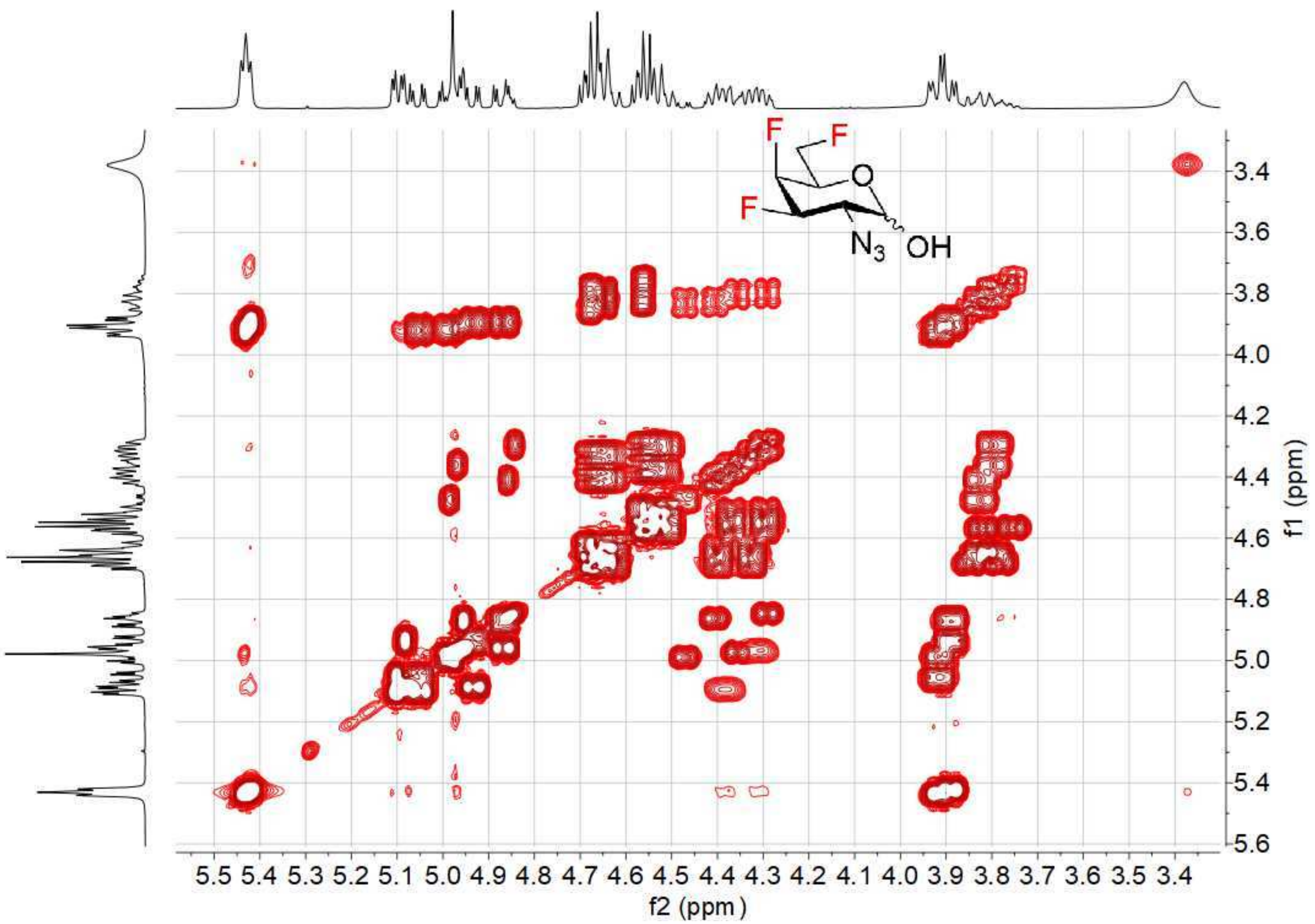
f2 (ppm)
f1 (ppm)
5.5 5.4 5.3 5.2 5.1 5.0 4.9 4.8 4.7 4.6 4.5 4.4 4.3 4.2 4.1 4.0 3.9 3.8 3.7 3.6 3.5 3.4
3.4 3.6 3.8 4.0 4.2 4.4 4.6 4.8 5.0 5.2 5.4 5.6
F
F
F
O
N3
OH

^{1}H-^{13}C HMBC 42

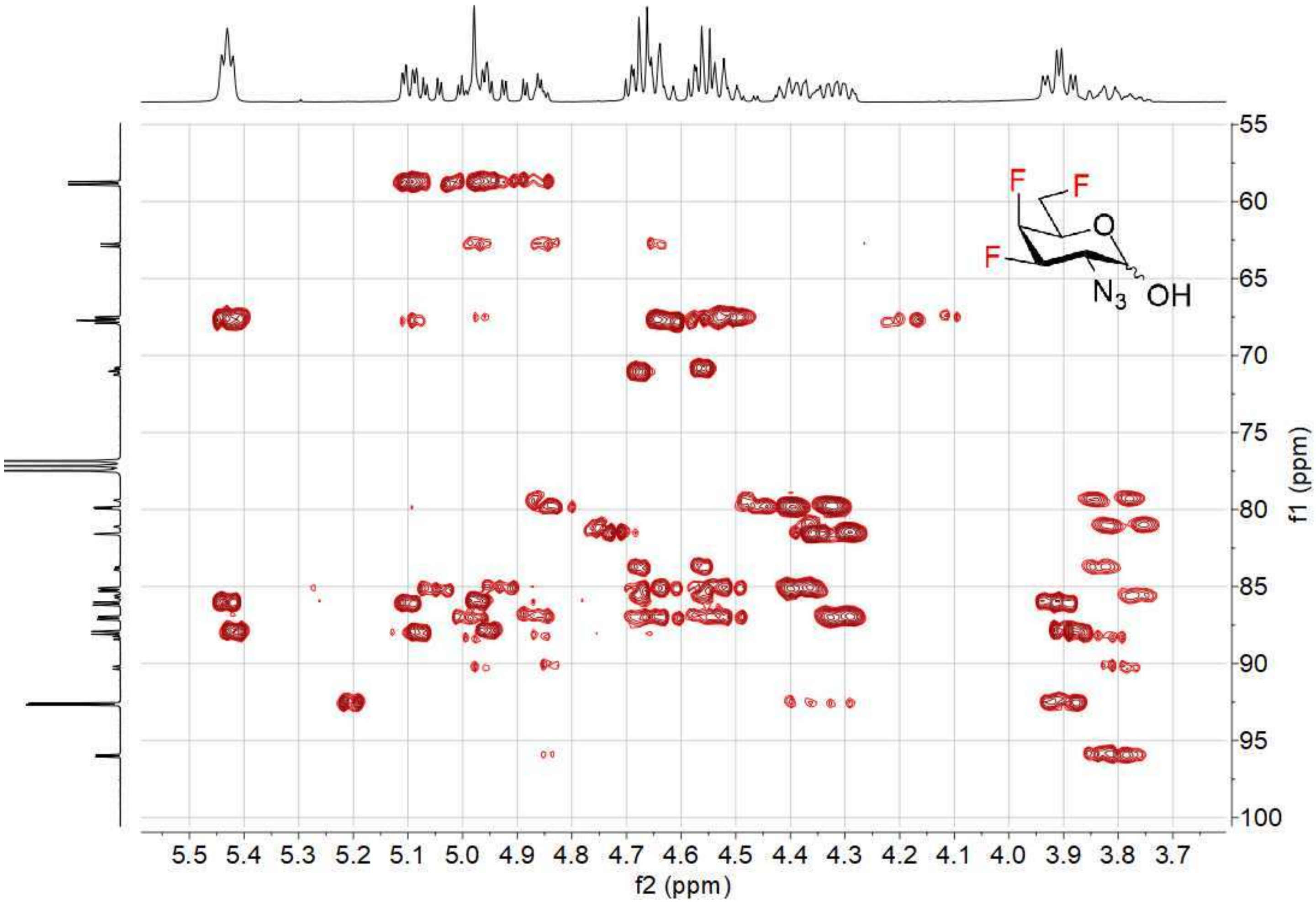

^{1}H-^{13}C HSQC 42

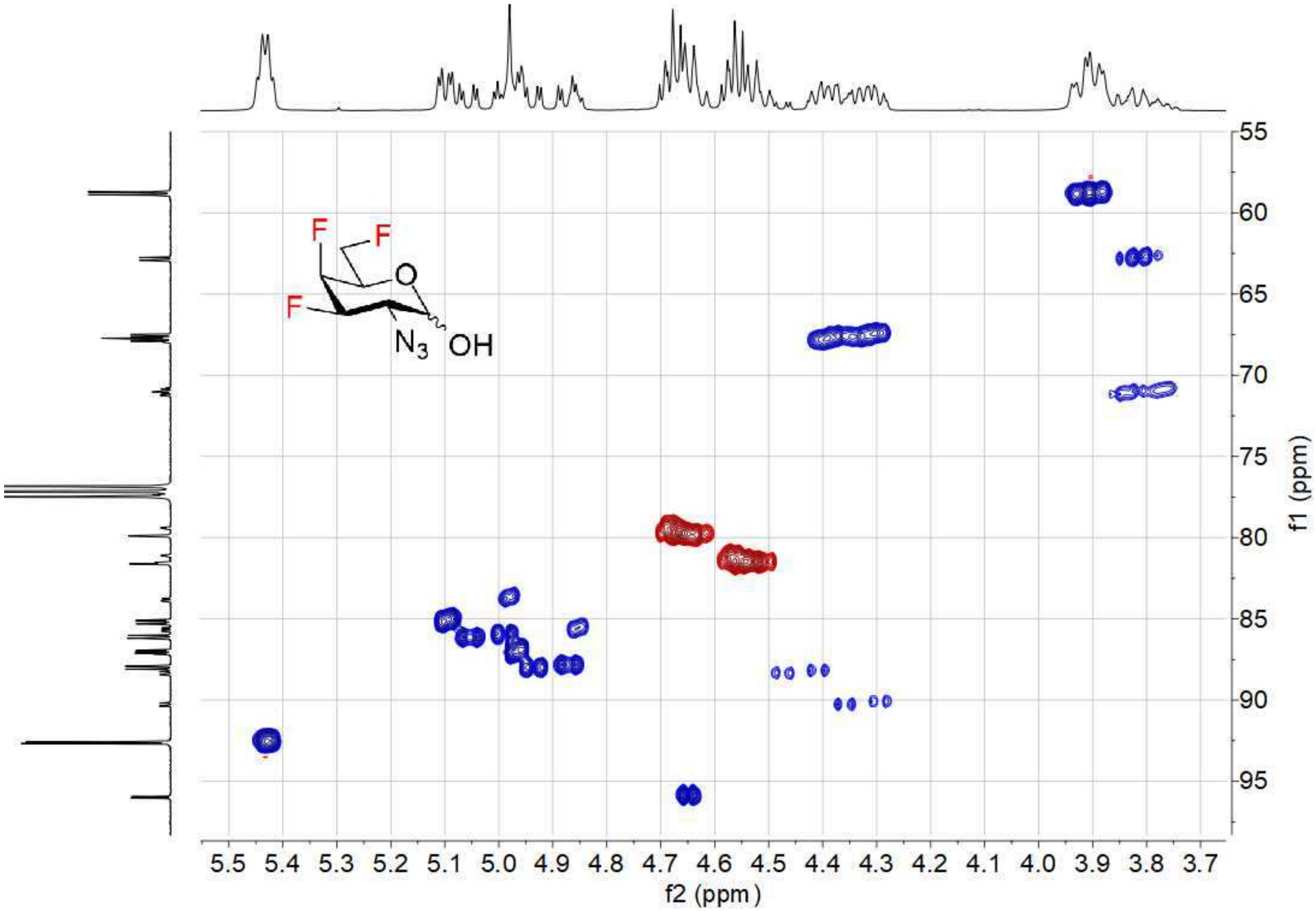

NMR COMPOUND 43

^{1}H NMR (400 MHz, $CDCl_3$) 43 (α/β ca. 2/1)

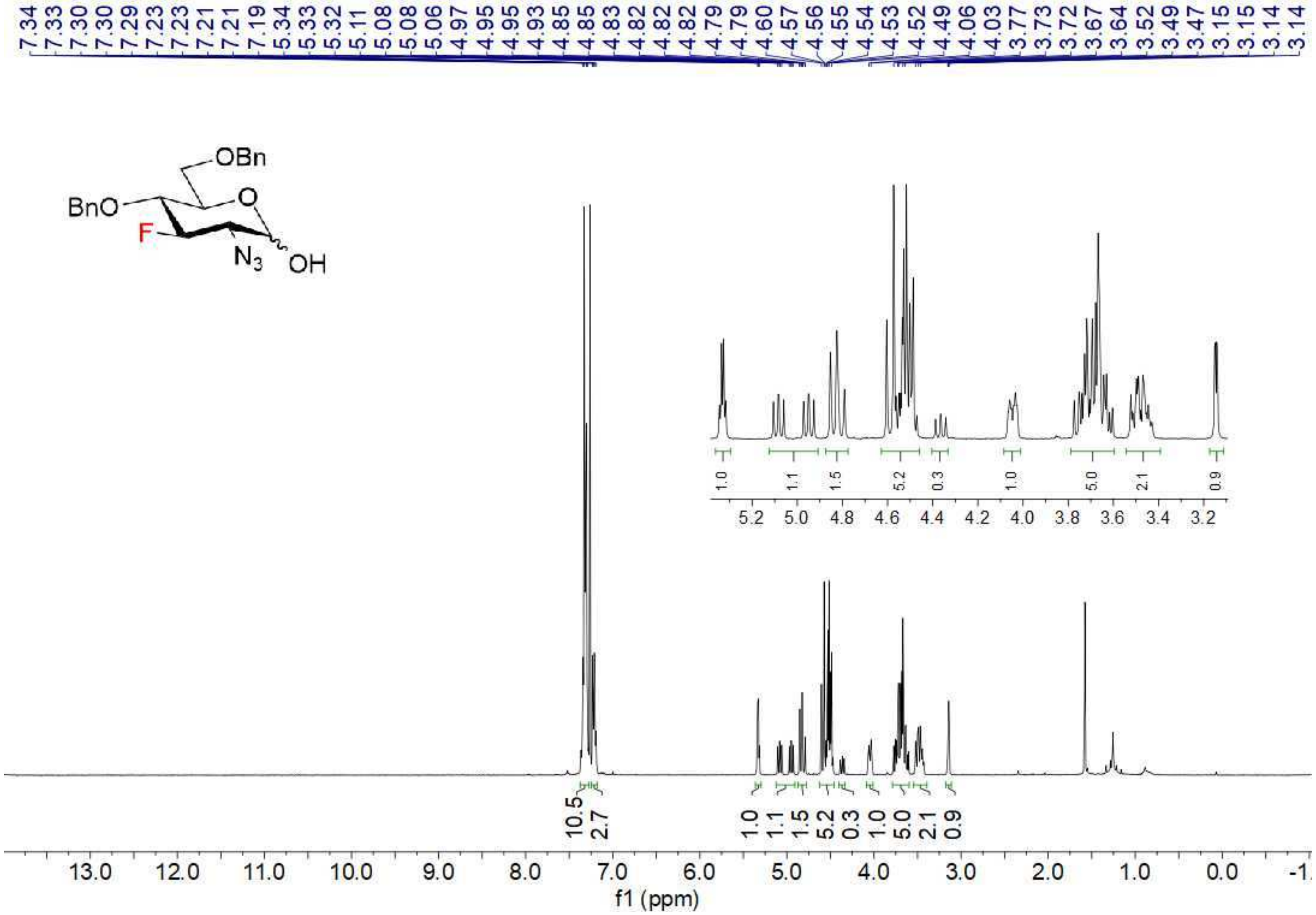

^{13}C APT NMR (100 MHz, $CDCl_3$) 43

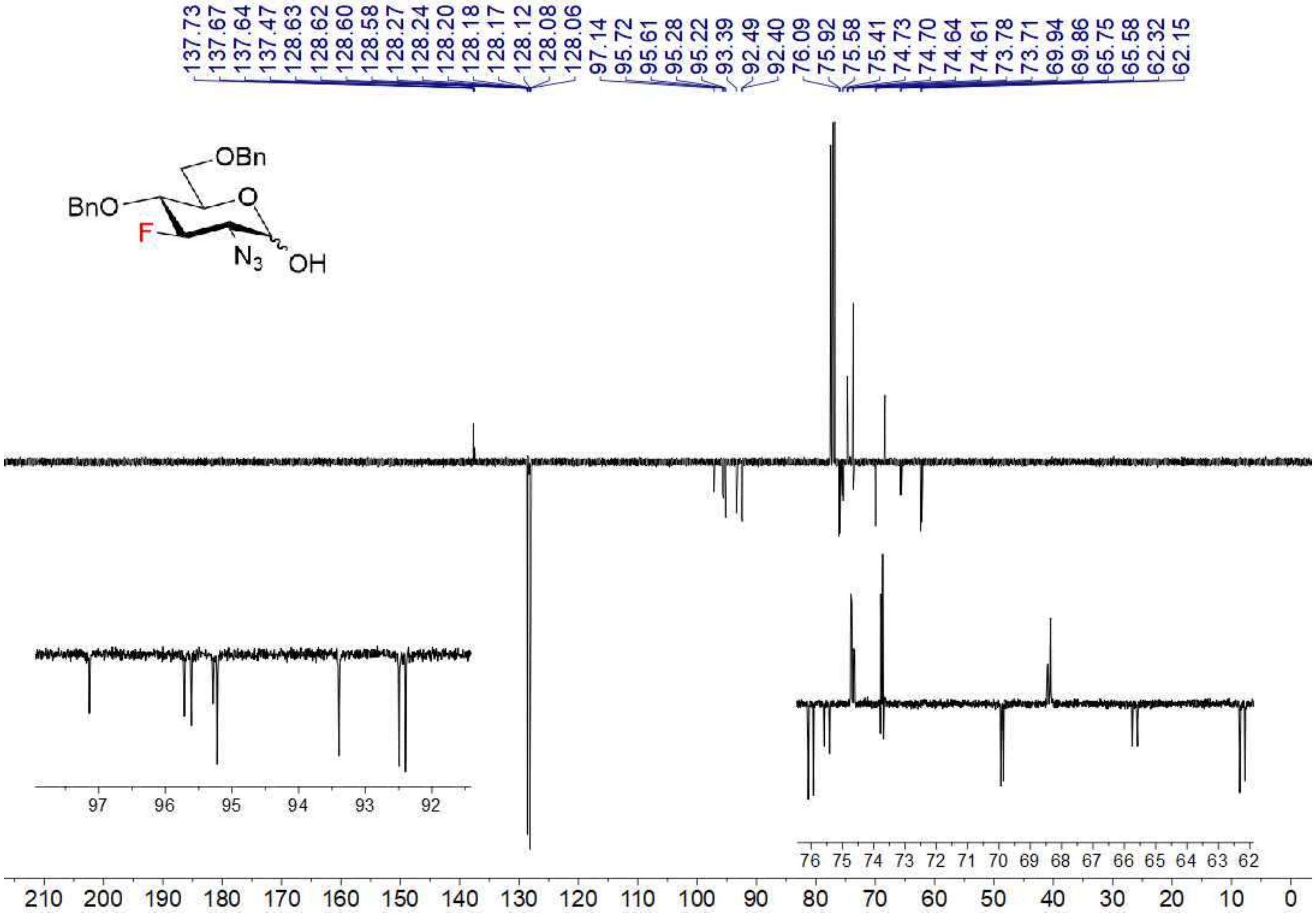

^{19}F NMR (376 MHz, CDCl$_3$) 43 (α/β ca. 2/1)

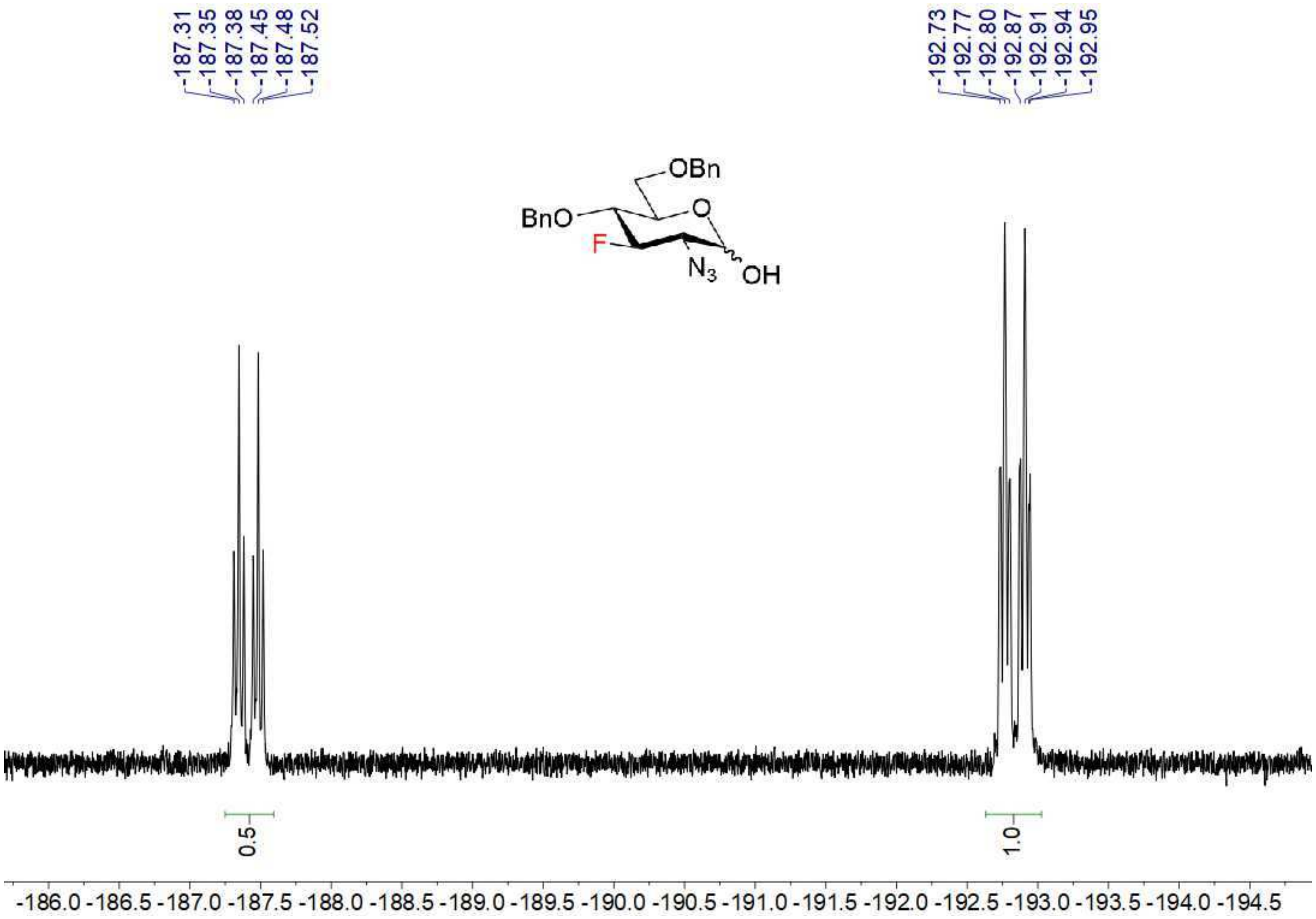

^{1}H-^{1}H COSY 43

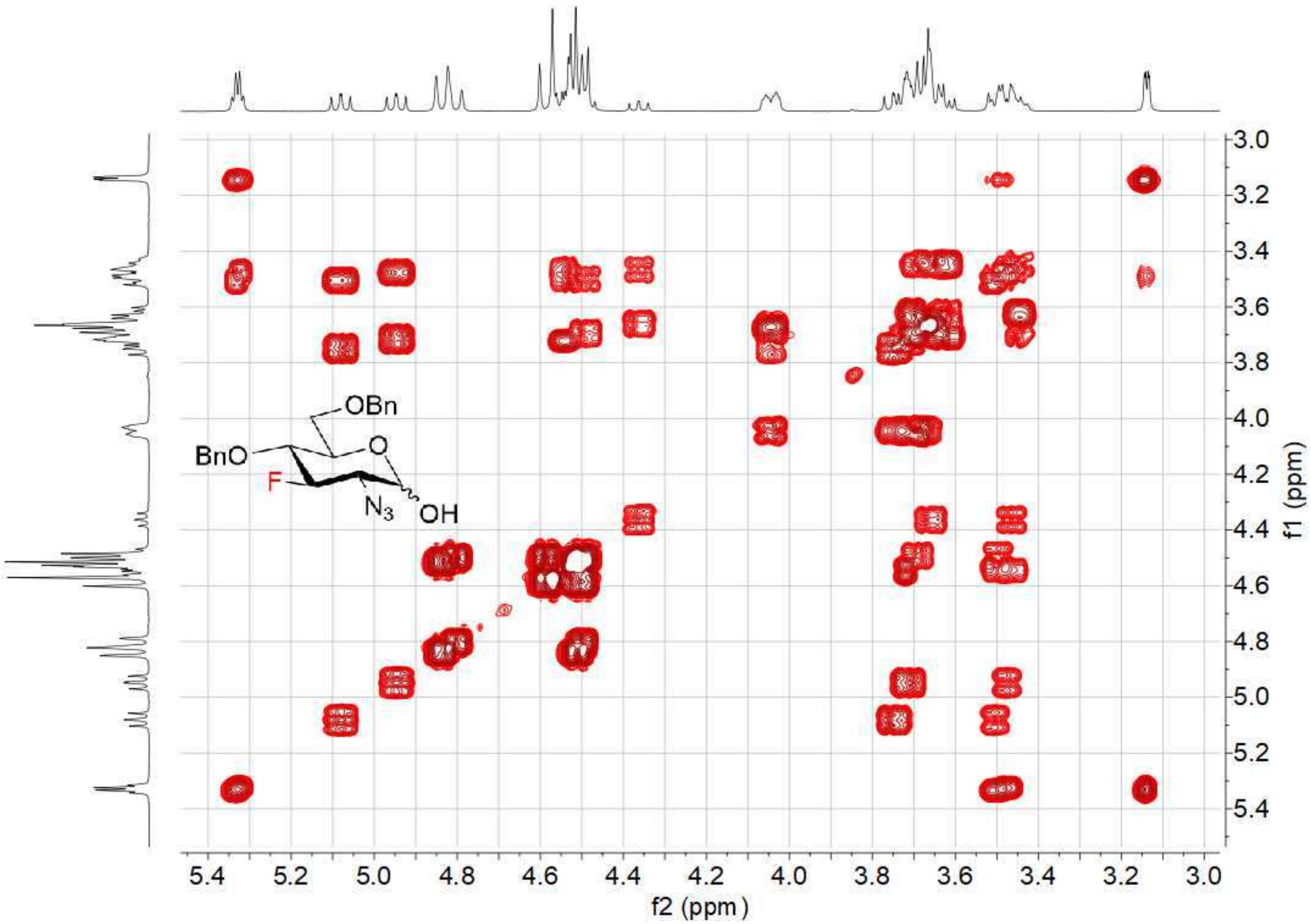

OBn
BnO
O
F
N3
OH
f1 (ppm)
f2 (ppm)
3.0
3.2
3.4
3.6
3.8
4.0
4.2
4.4
4.6
4.8
5.0
5.2
5.4
5.4
5.2
5.0
4.8
4.6
4.4
4.2
4.0
3.8
3.6
3.4
3.2
3.0

^{1}H-^{13}C HSQC 43

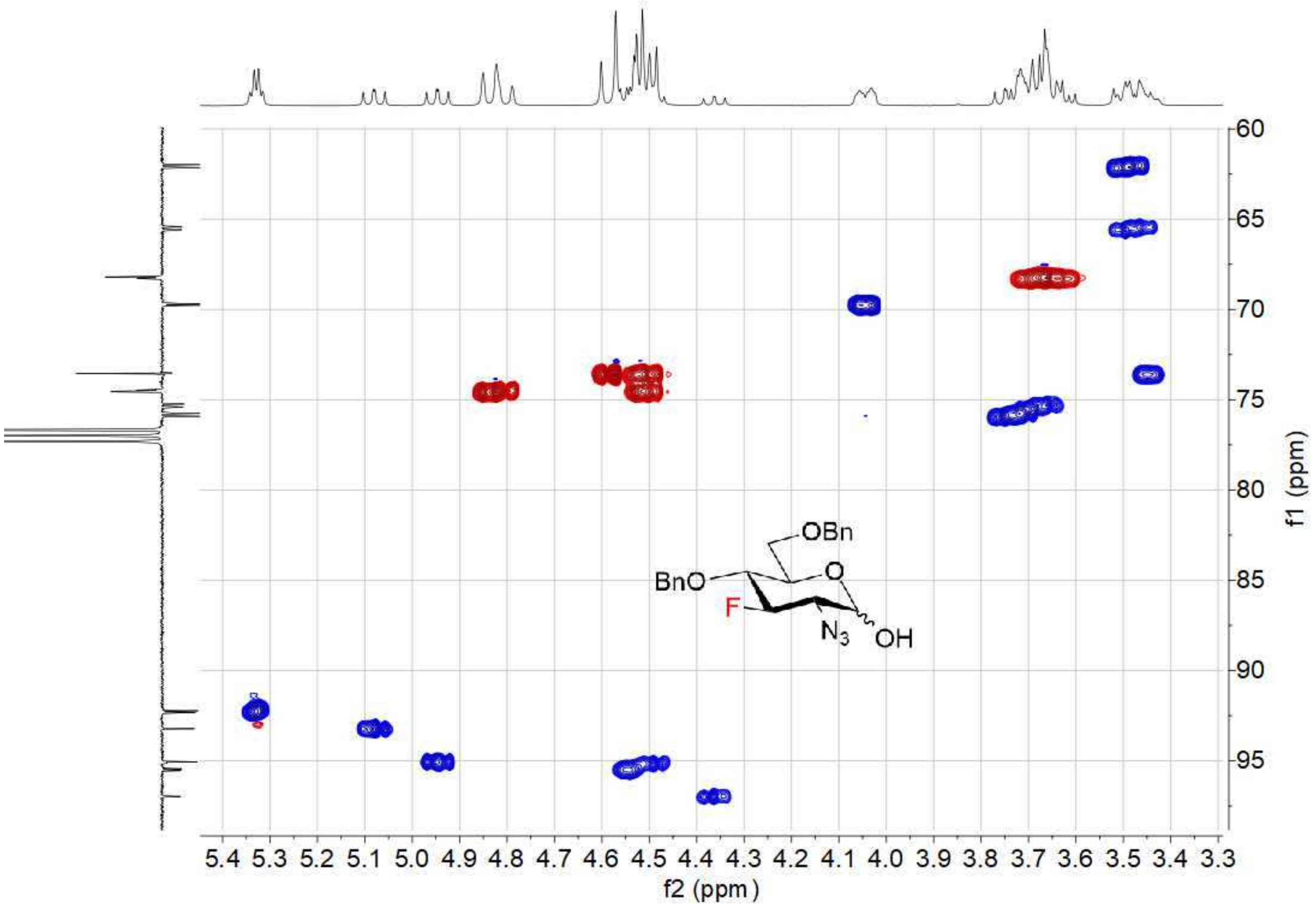

NMR COMPOUND 44

^{1}H NMR (400 MHz, $CDCl_3$) 44 (α/β ca. 2/1)

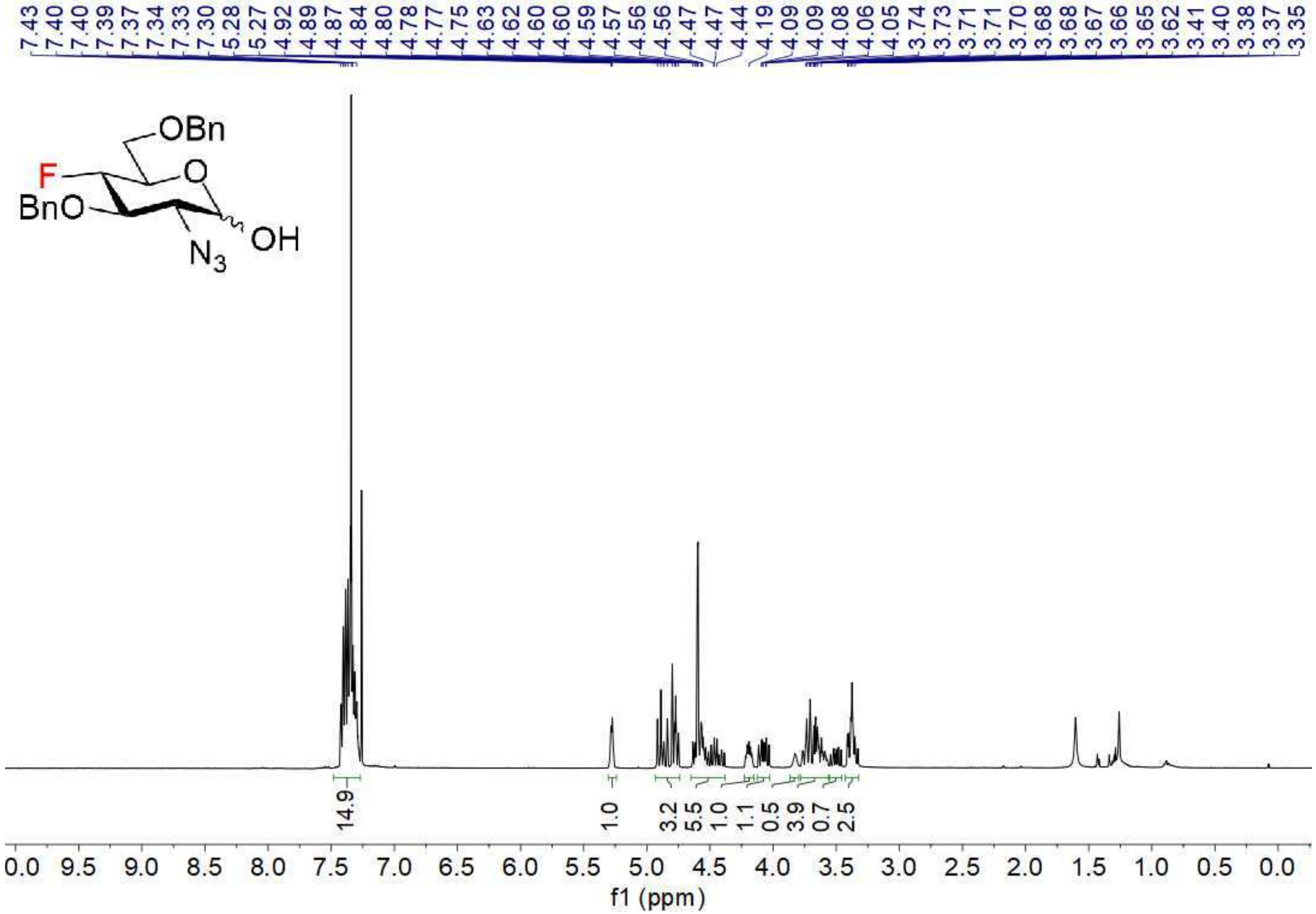

^{13}C NMR (100 MHz, CDCl$_3$) 44

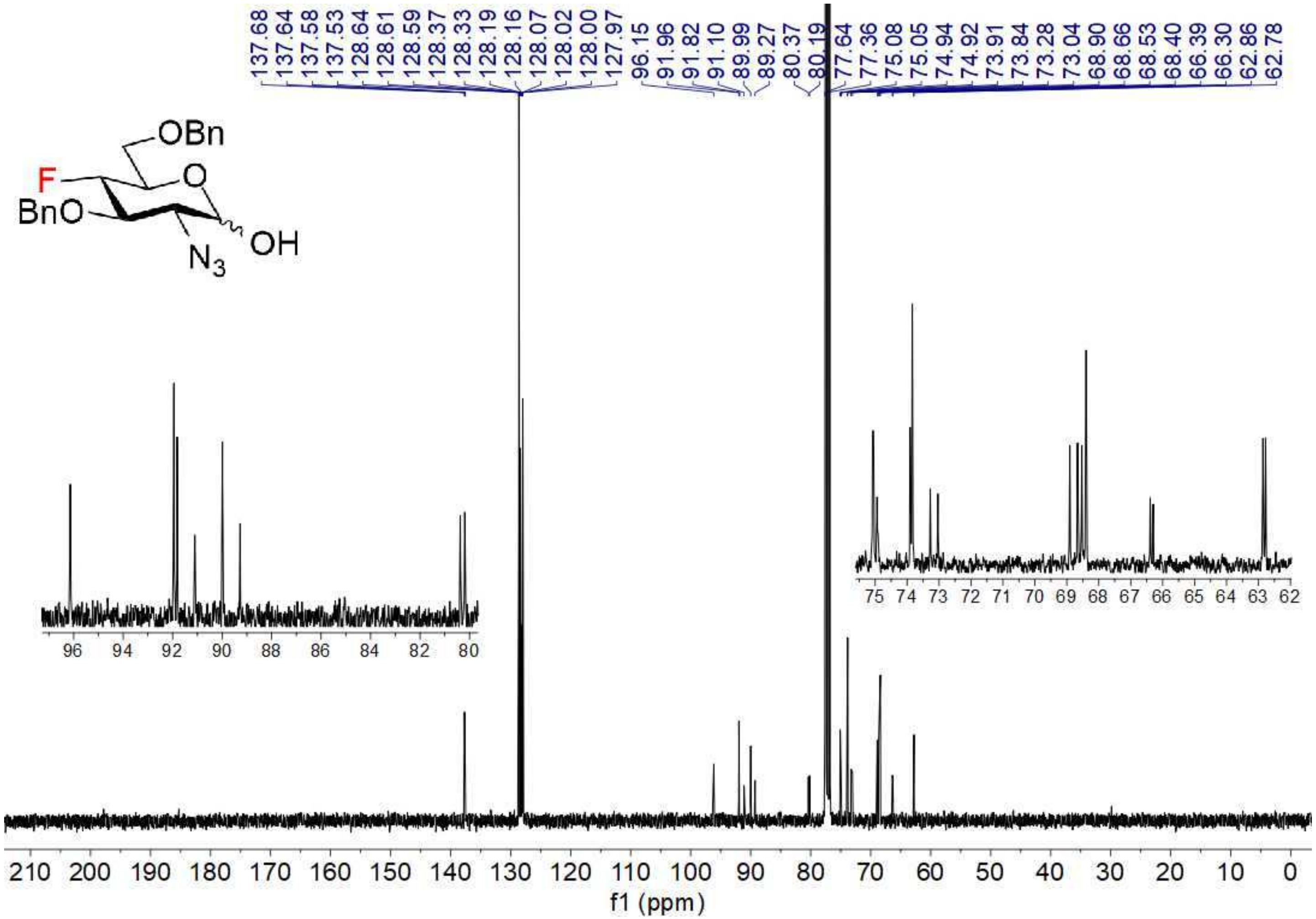

^{19}F NMR (376 MHz, $CDCl_3$) 44 (α/β ca. 2/1)

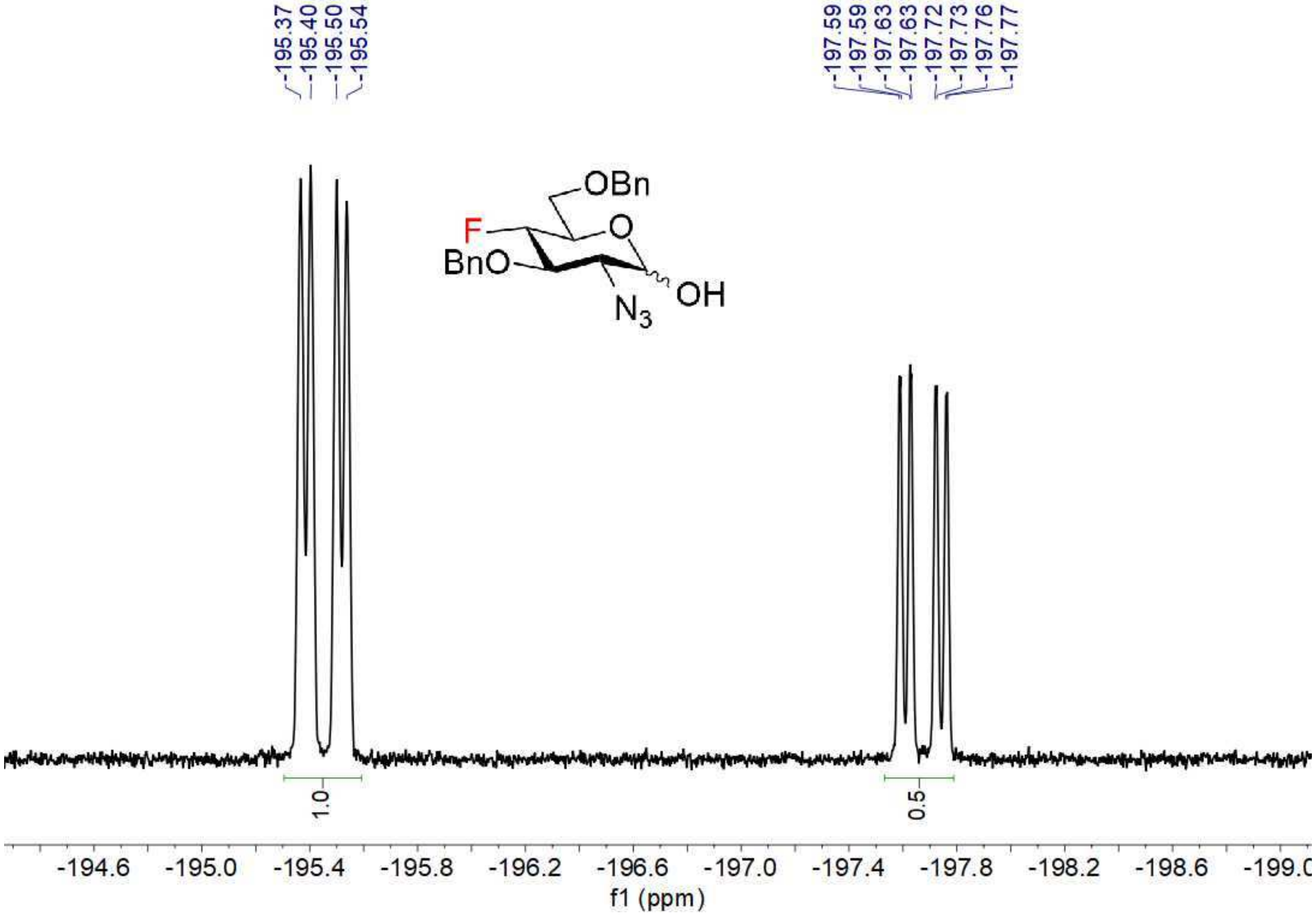

^{1}H-^{1}H COSY 44

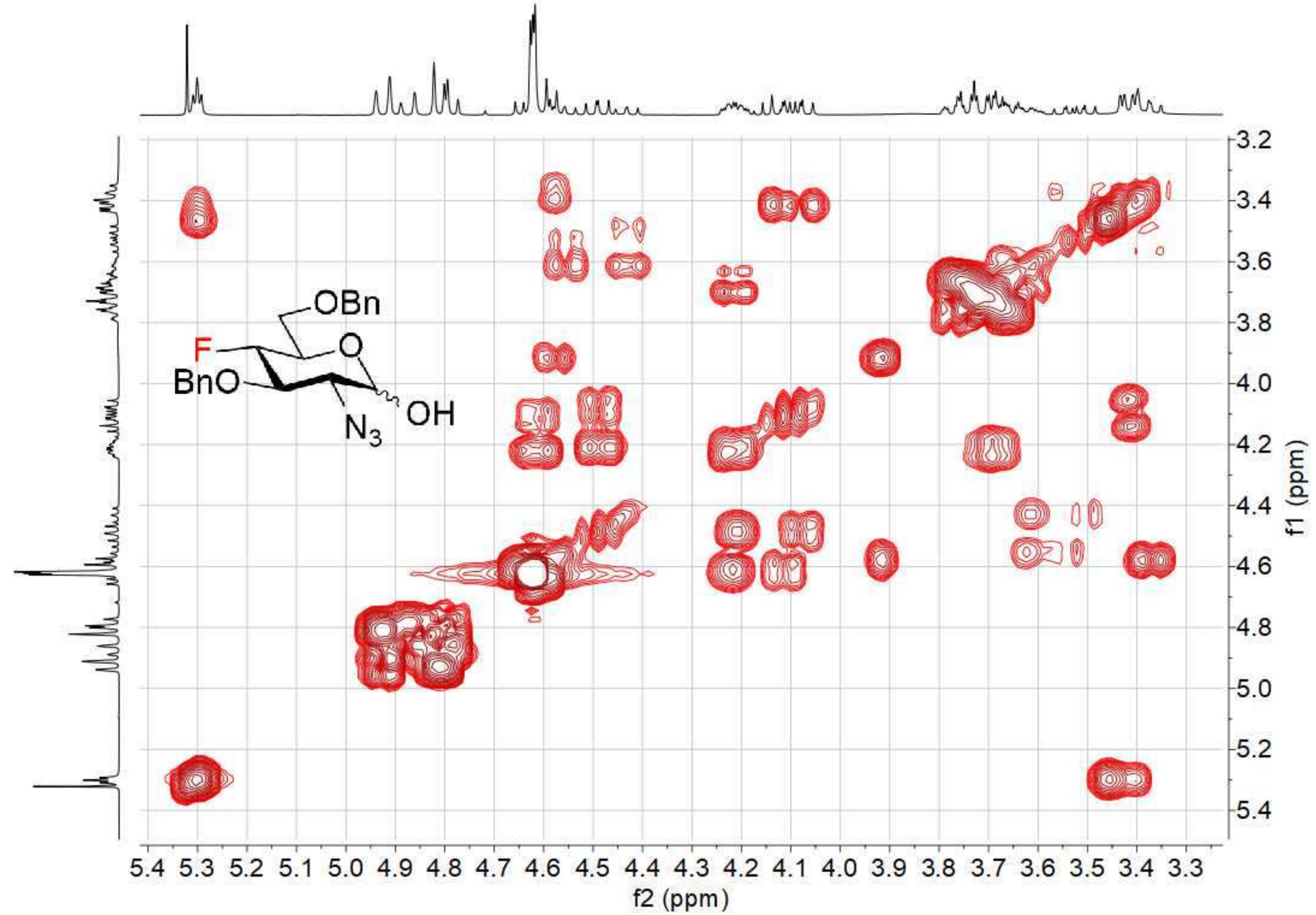

^{1}H-^{13}C HSQC 44

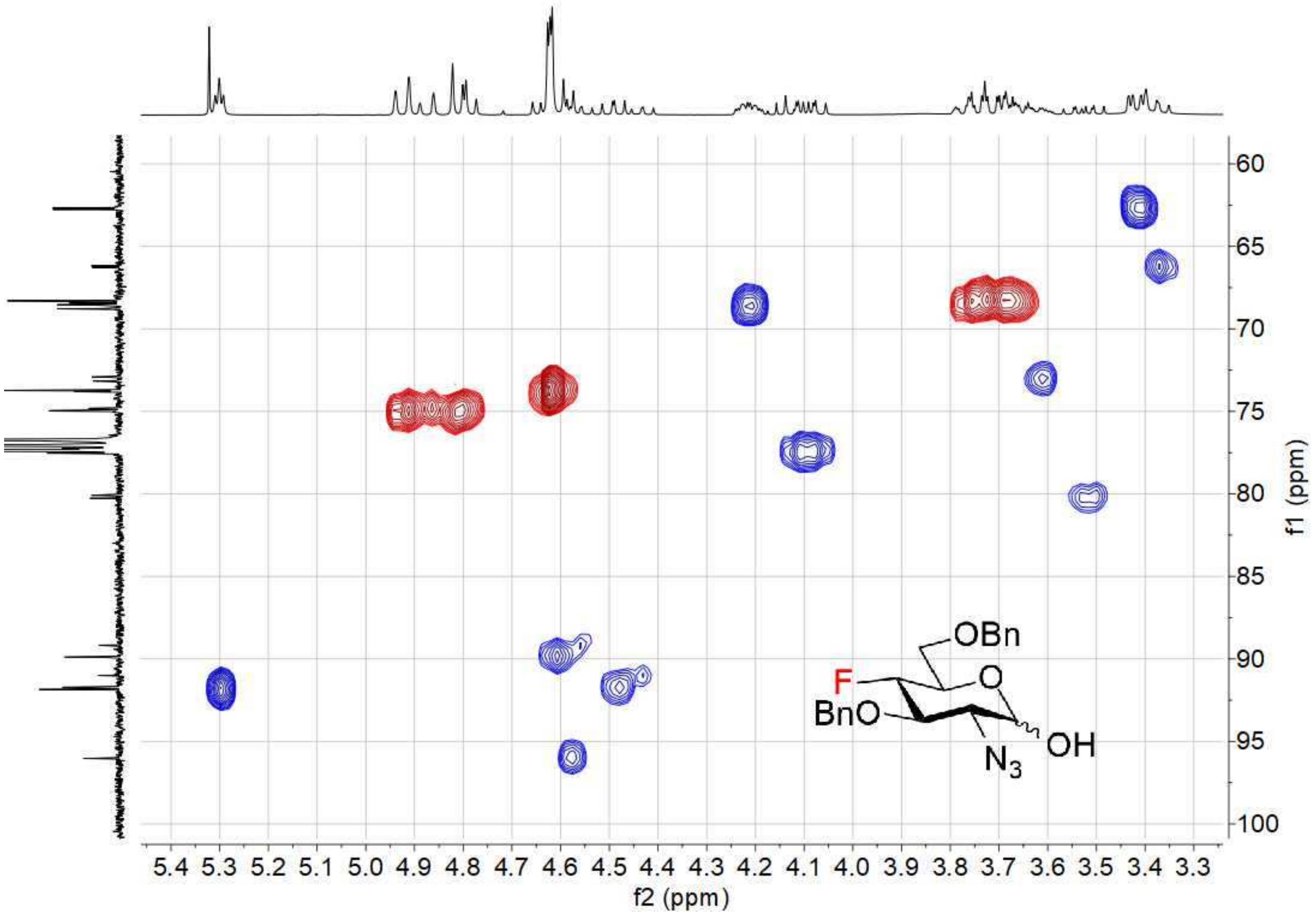

NMR COMPOUND 45

^{1}H NMR (400 MHz, $CDCl_3$) 45 (α/β ca. 5/3)

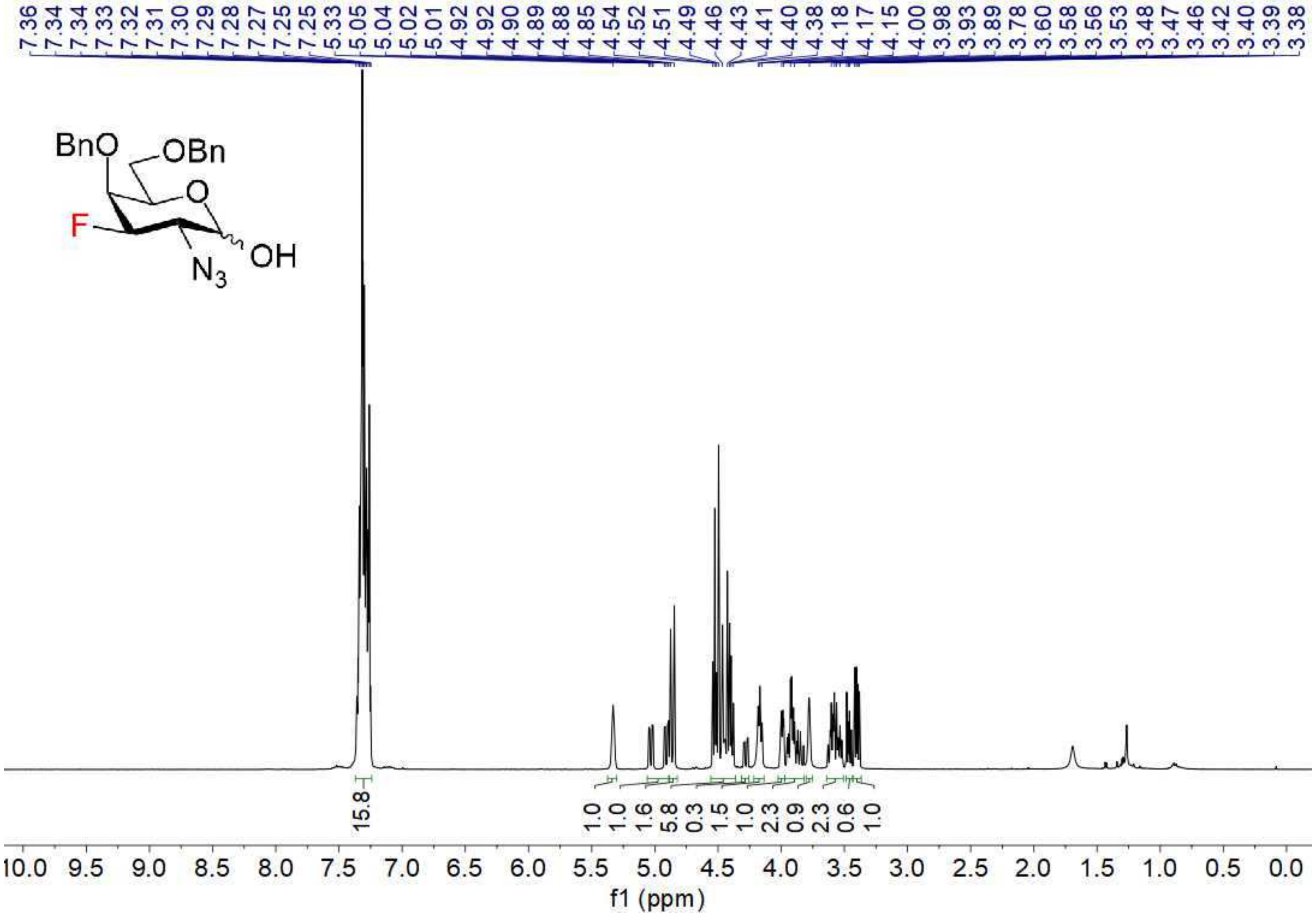

^{13}C NMR (100 MHz, CDCl$_3$) 45

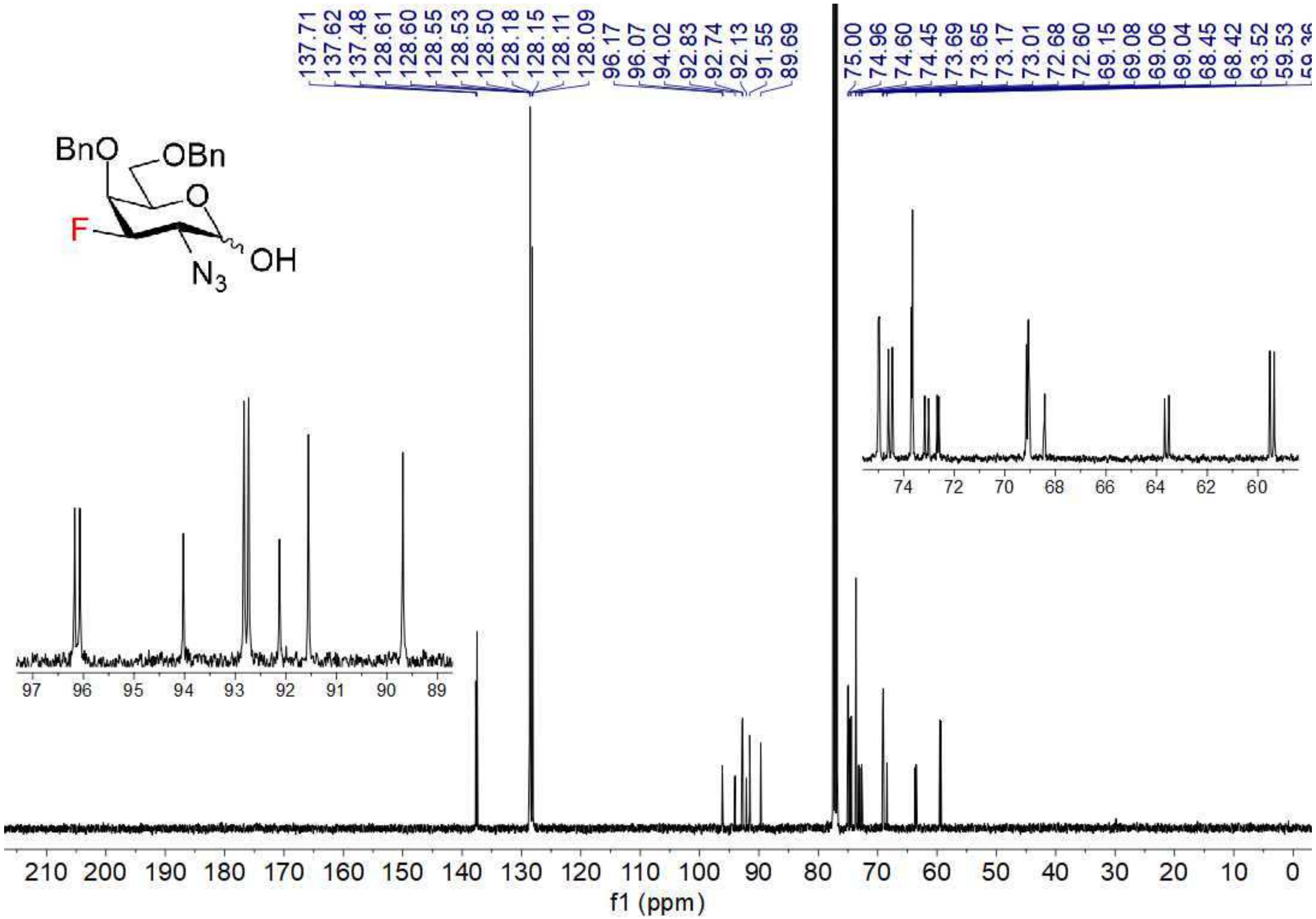

^{19}F NMR (376 MHz, CDCl$_3$) 45 (α/β ca. 5/3)

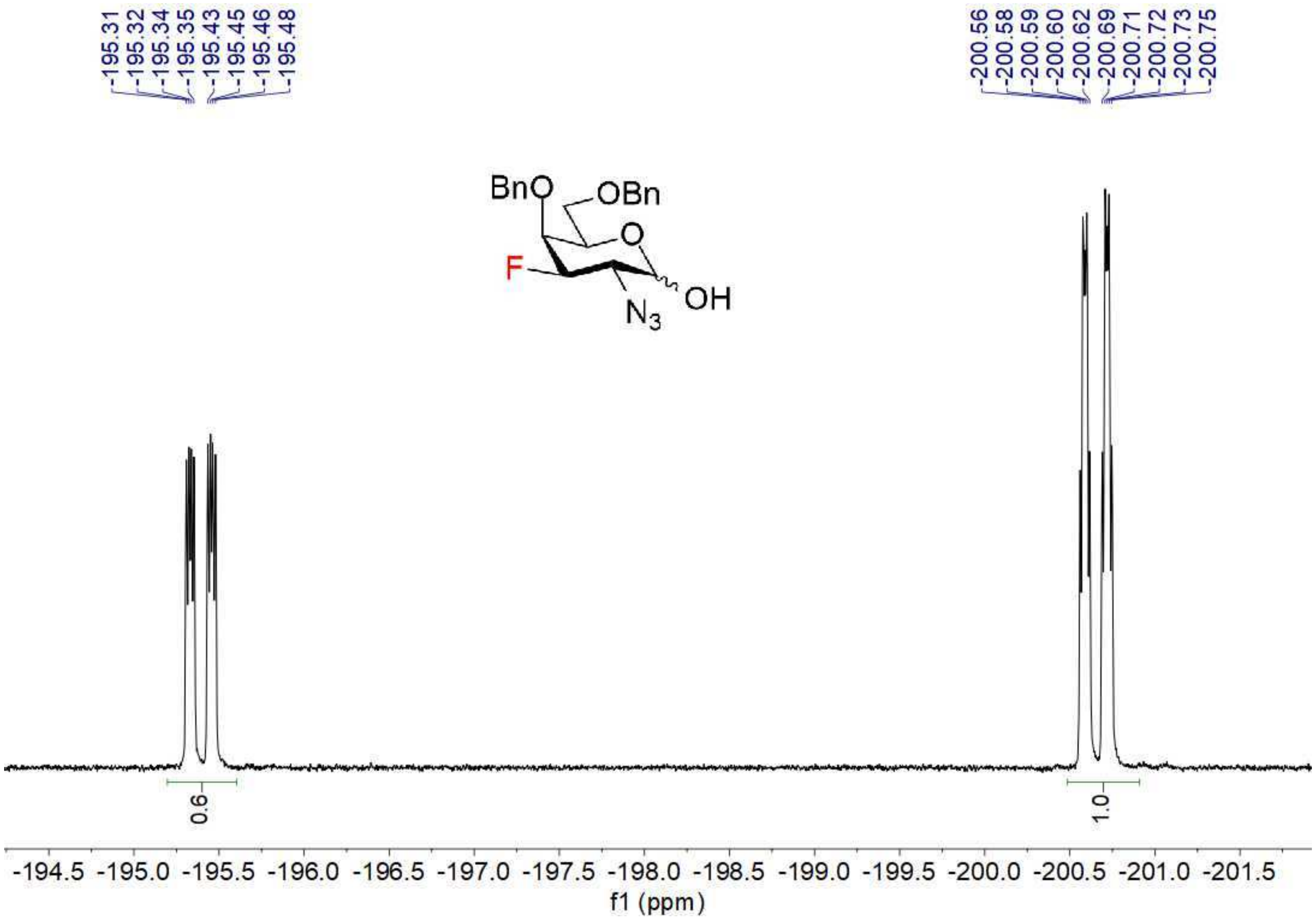

^{1}H-^{1}H COSY 45

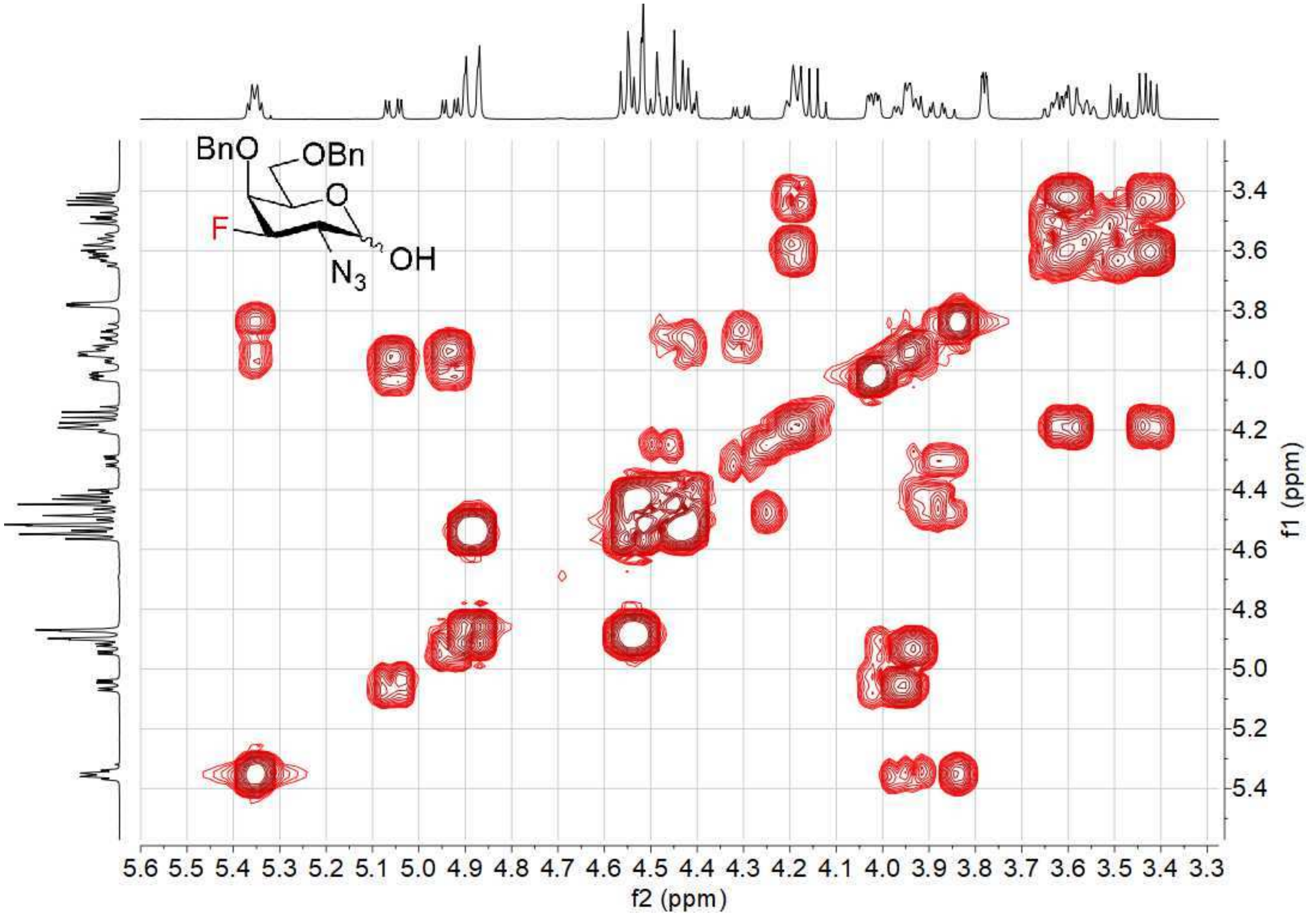

BnO
OBn
O
F
N3
OH
5.6 5.5 5.4 5.3 5.2 5.1 5.0 4.9 4.8 4.7 4.6 4.5 4.4 4.3 4.2 4.1 4.0 3.9 3.8 3.7 3.6 3.5 3.4 3.3
f2 (ppm)
3.4
3.6
3.8
4.0
4.2
4.4
4.6
4.8
5.0
5.2
5.4
f1 (ppm)

^{1}H-^{13}C HMBC 45

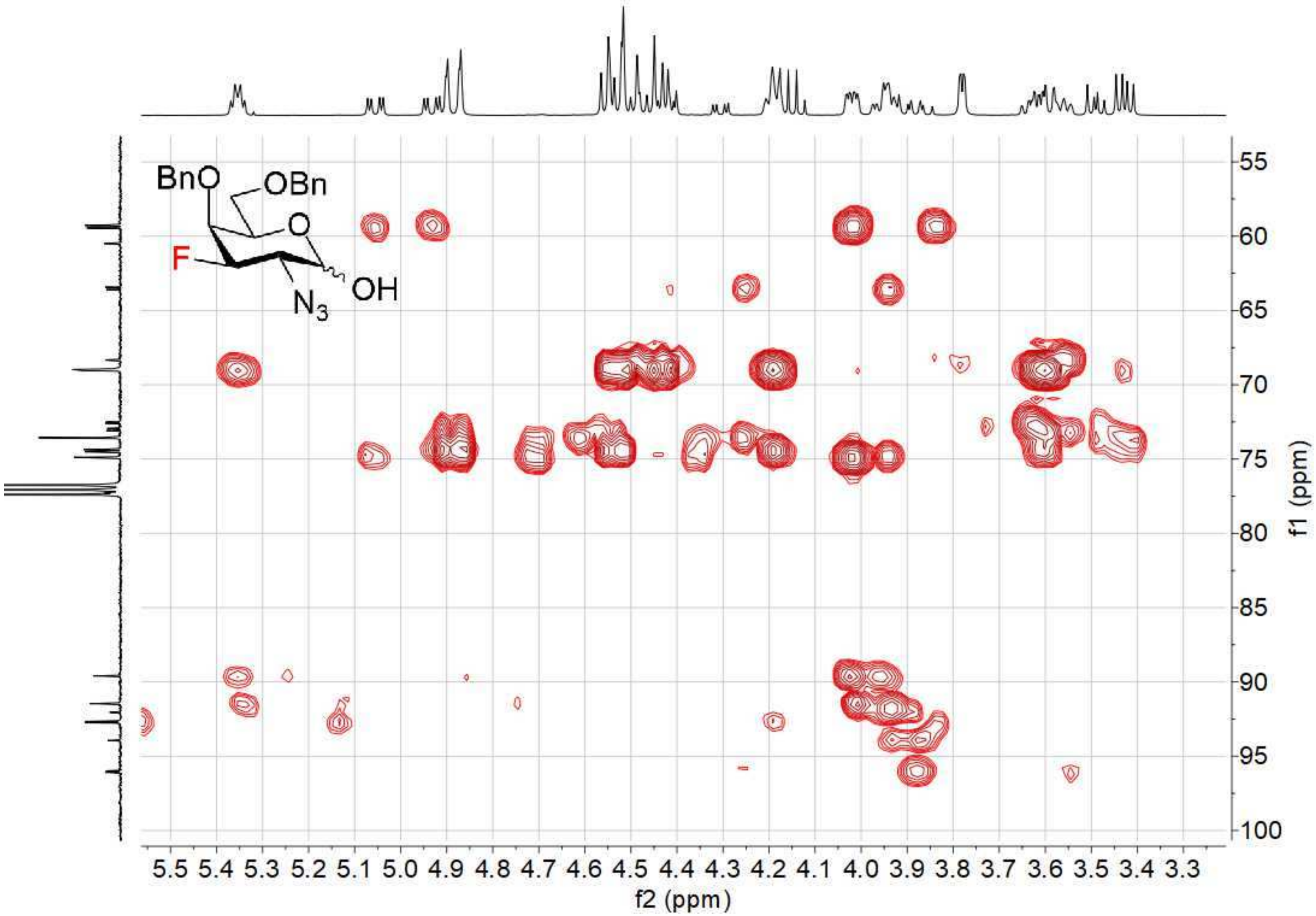

^{1}H-^{13}C HSQC 45

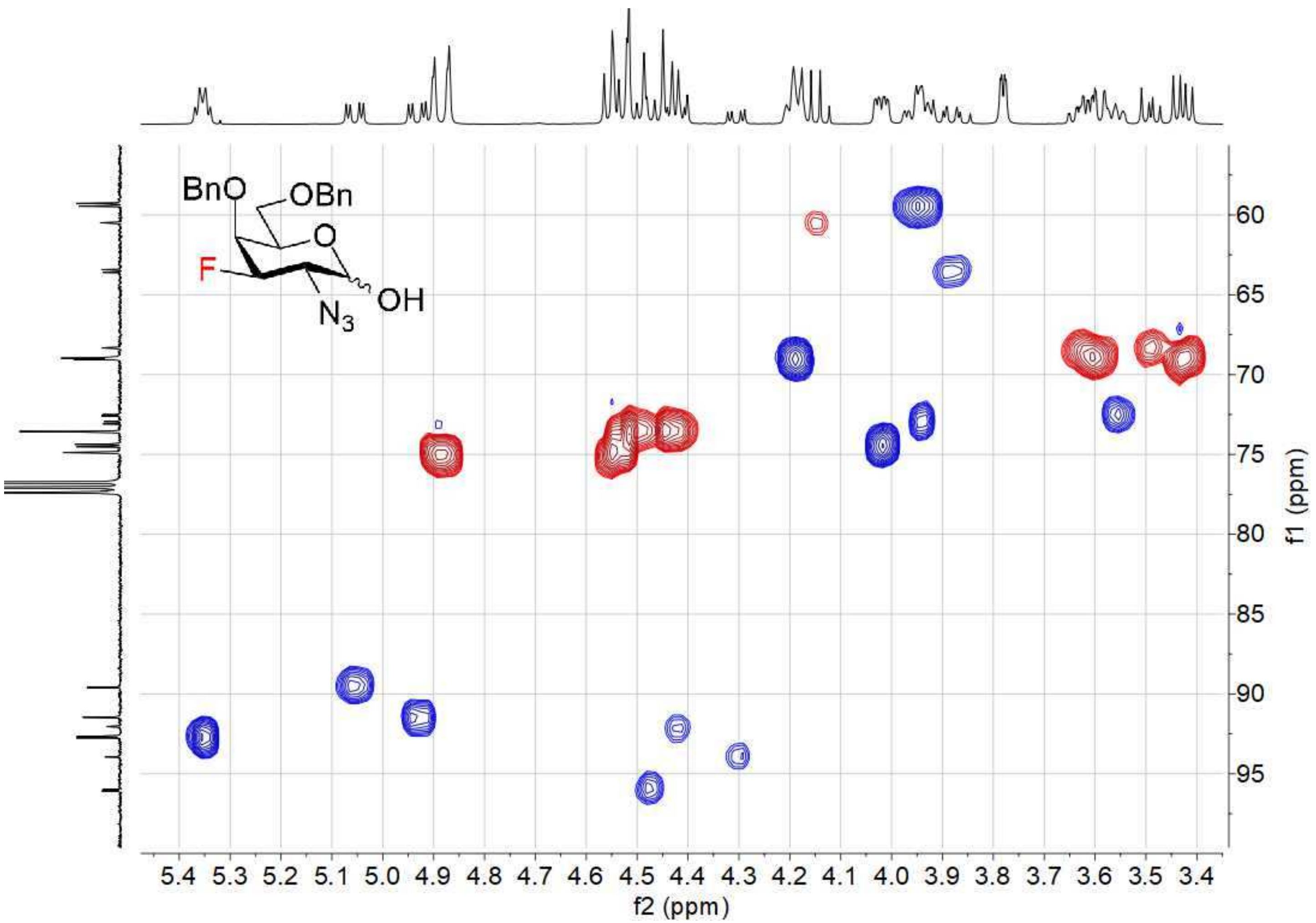

NMR COMPOUND 46

^{1}H NMR (400 MHz, $CDCl_3$) 46 (α/β ca. 5/2)

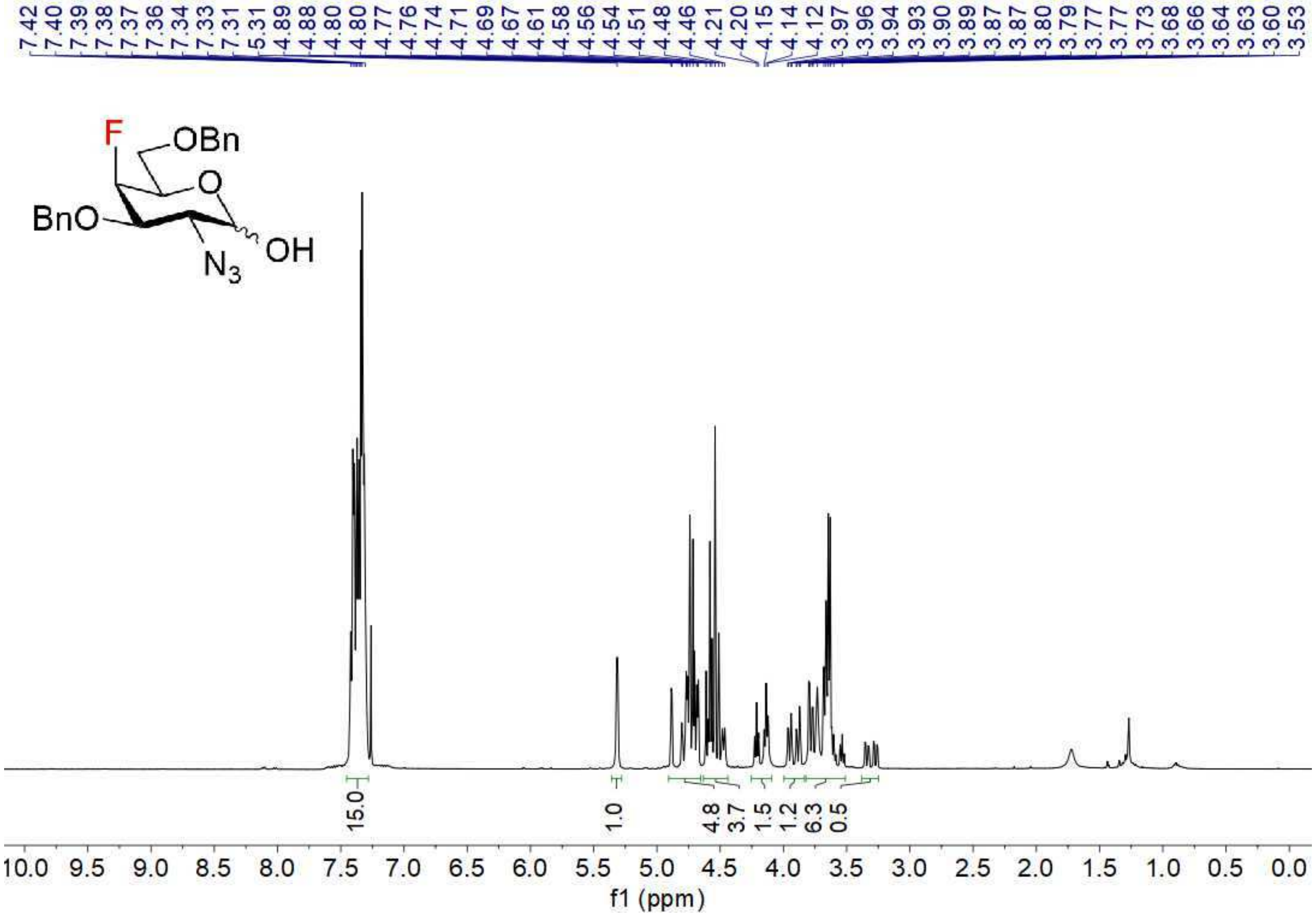

^{13}C NMR (100 MHz, $CDCl_3$) 46

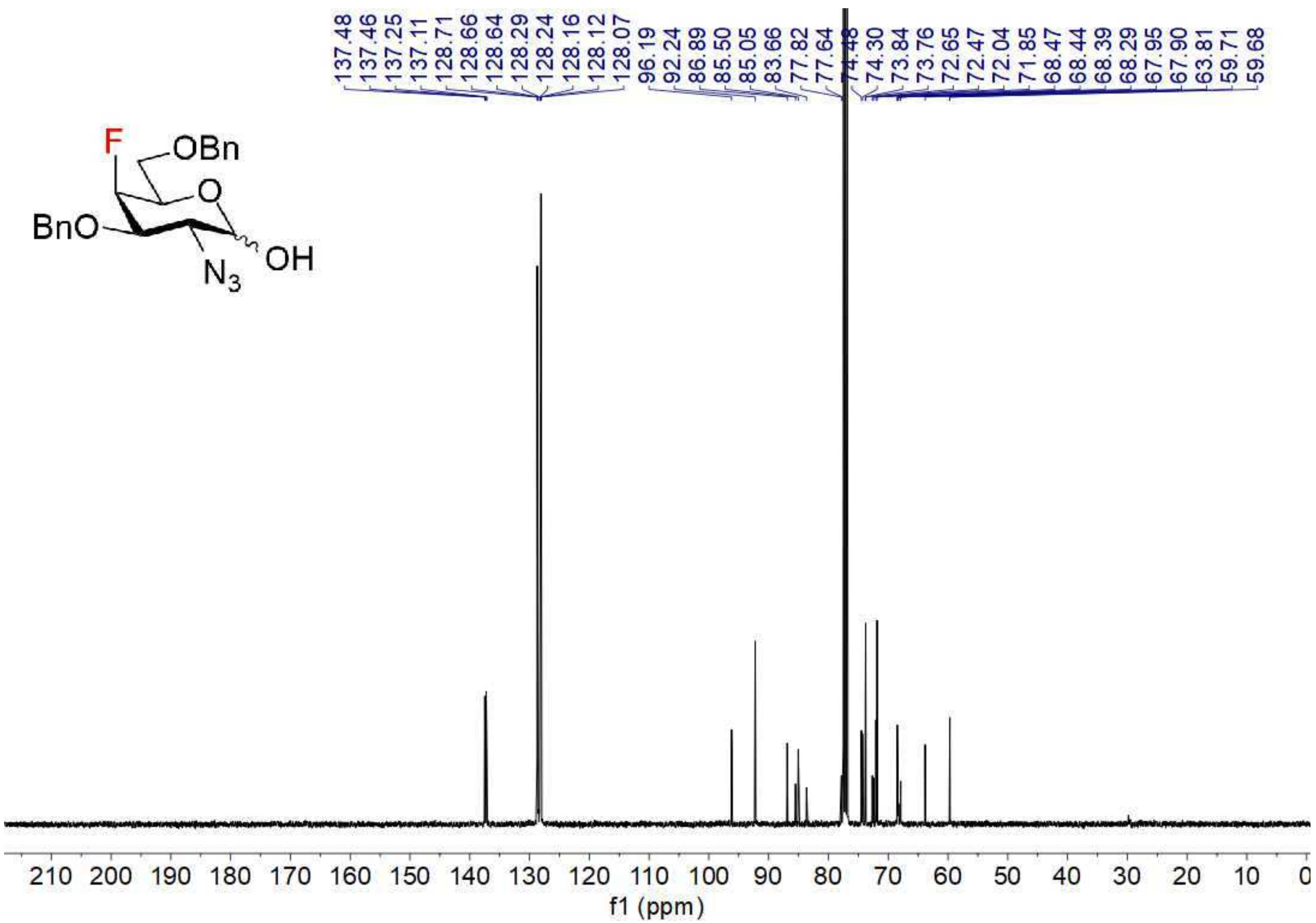

^{19}F NMR (376 MHz, CDCl$_3$) 46 (α/β ca. 5/2)

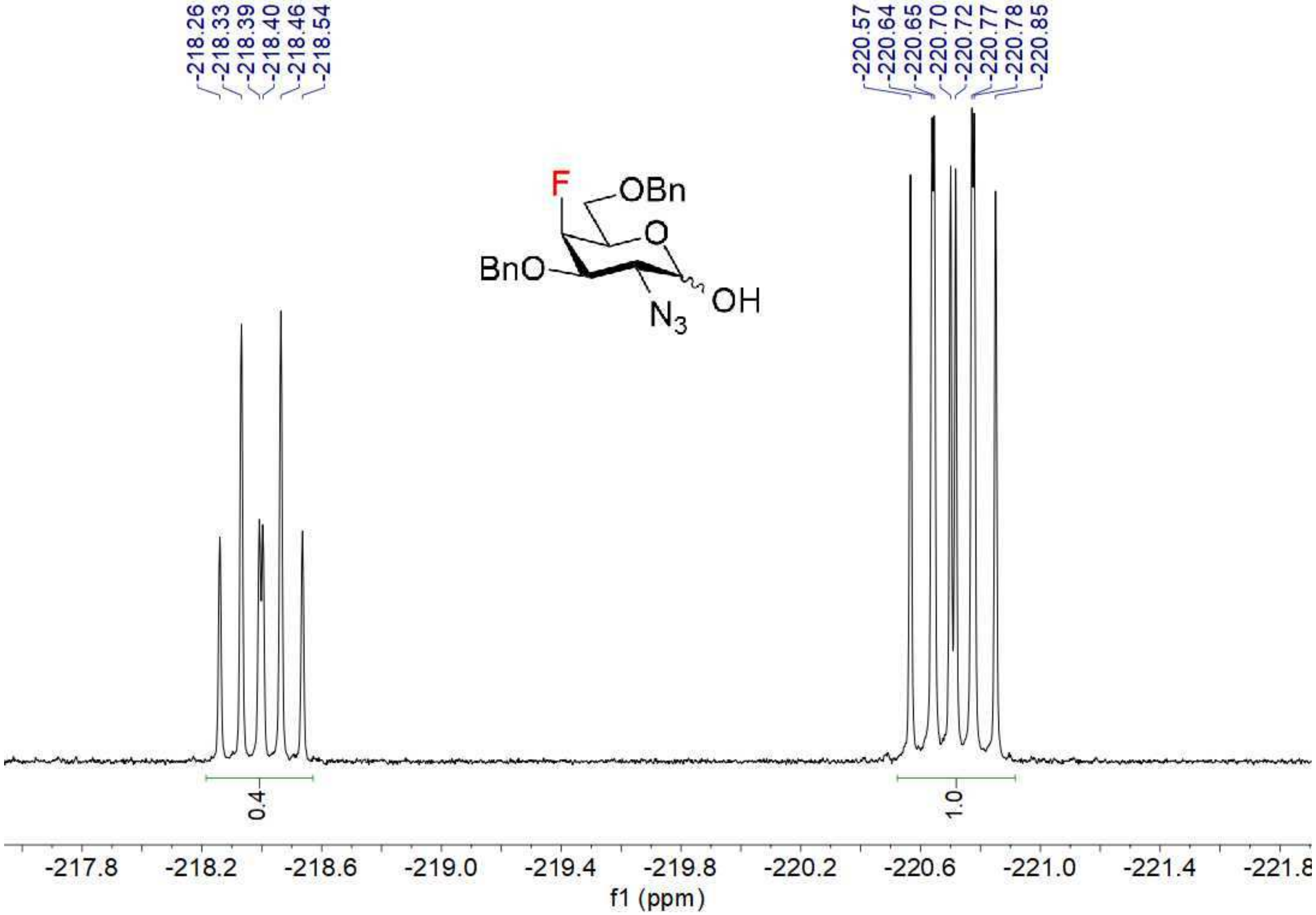

^{1}H-^{1}H COSY 46

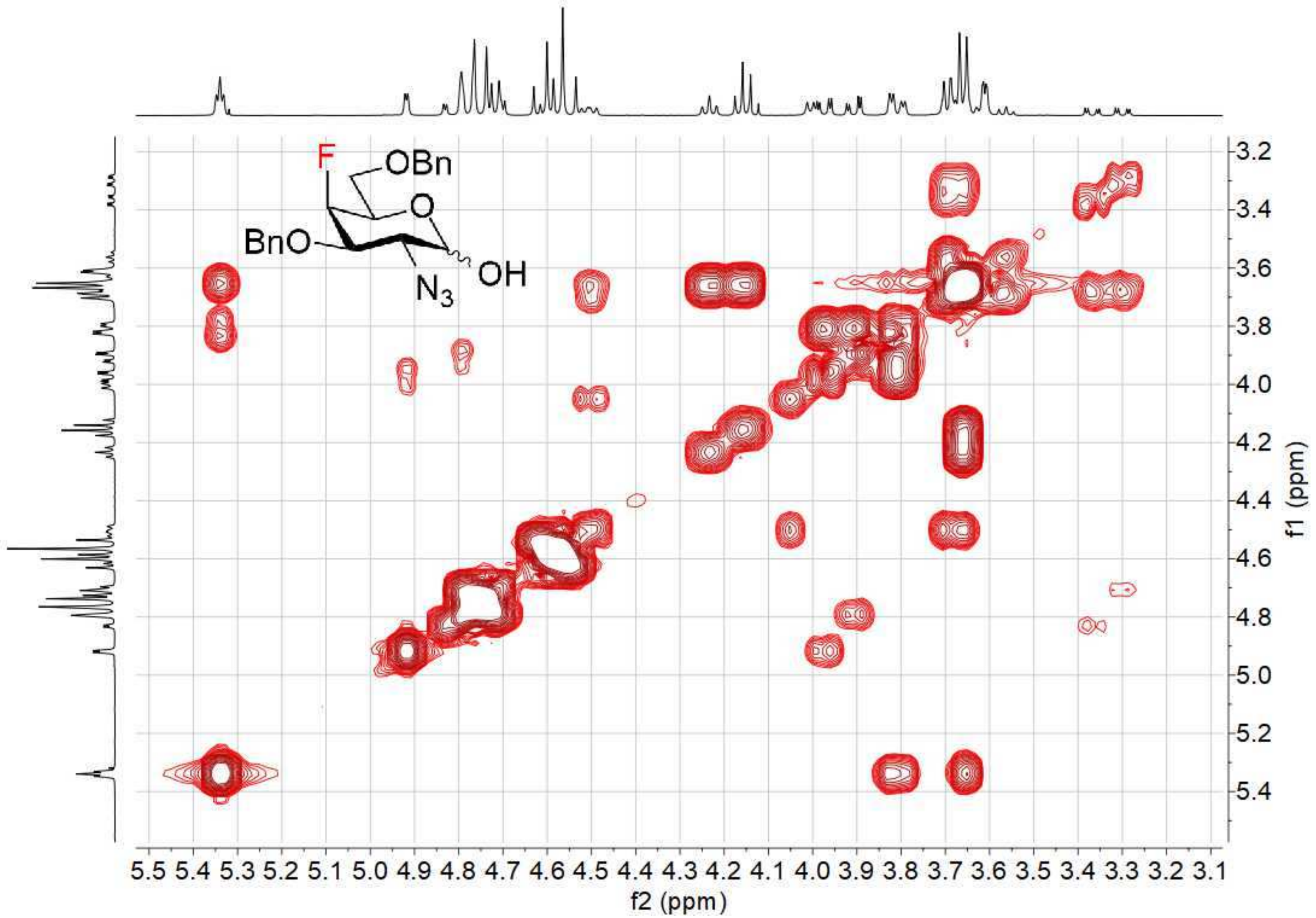

^{1}H-^{13}C HSQC 46

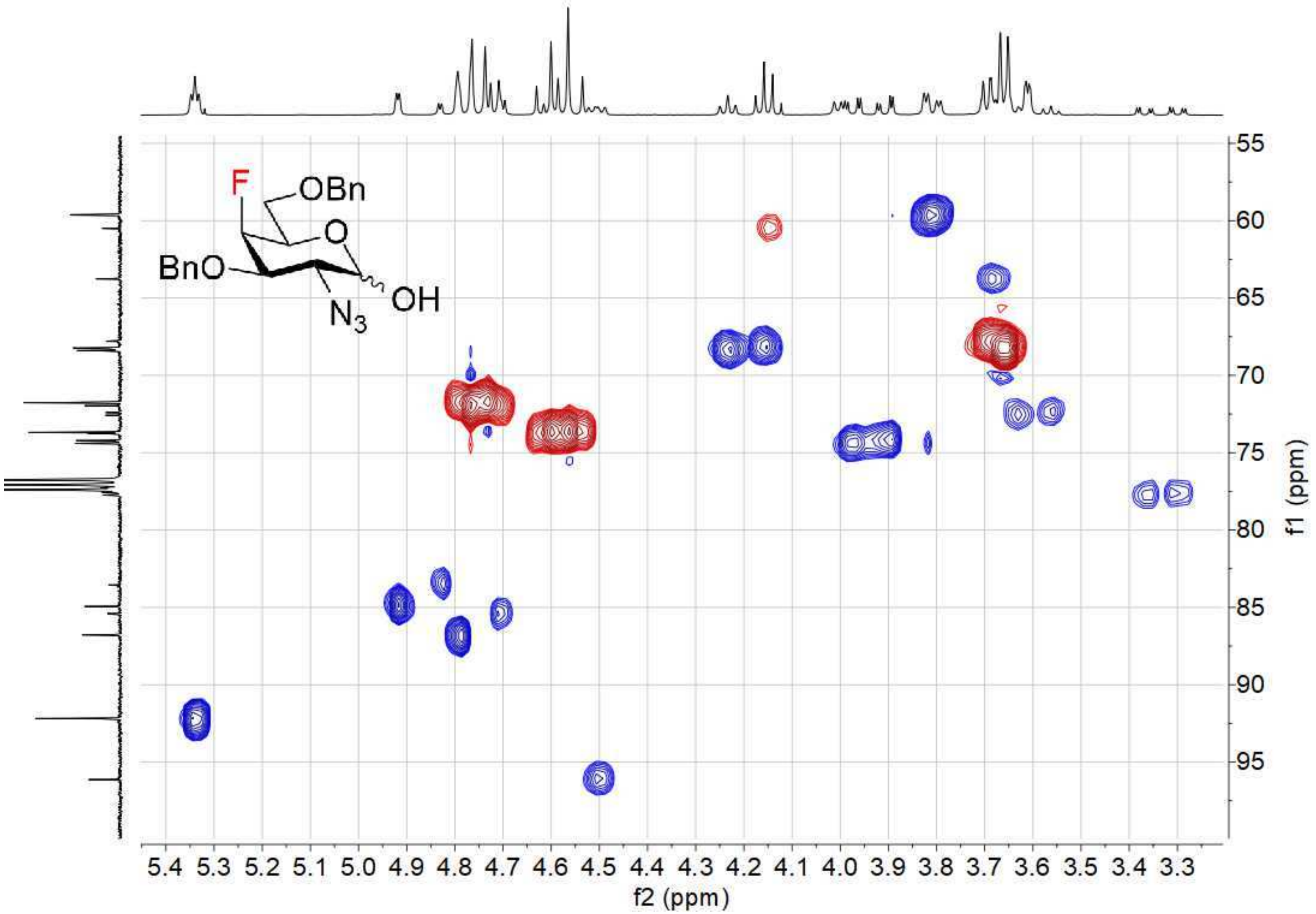

NMR COMPOUND 47

^{1}H NMR (400 MHz, CDCl$_3$) 47 (α/β ca. 2/1)

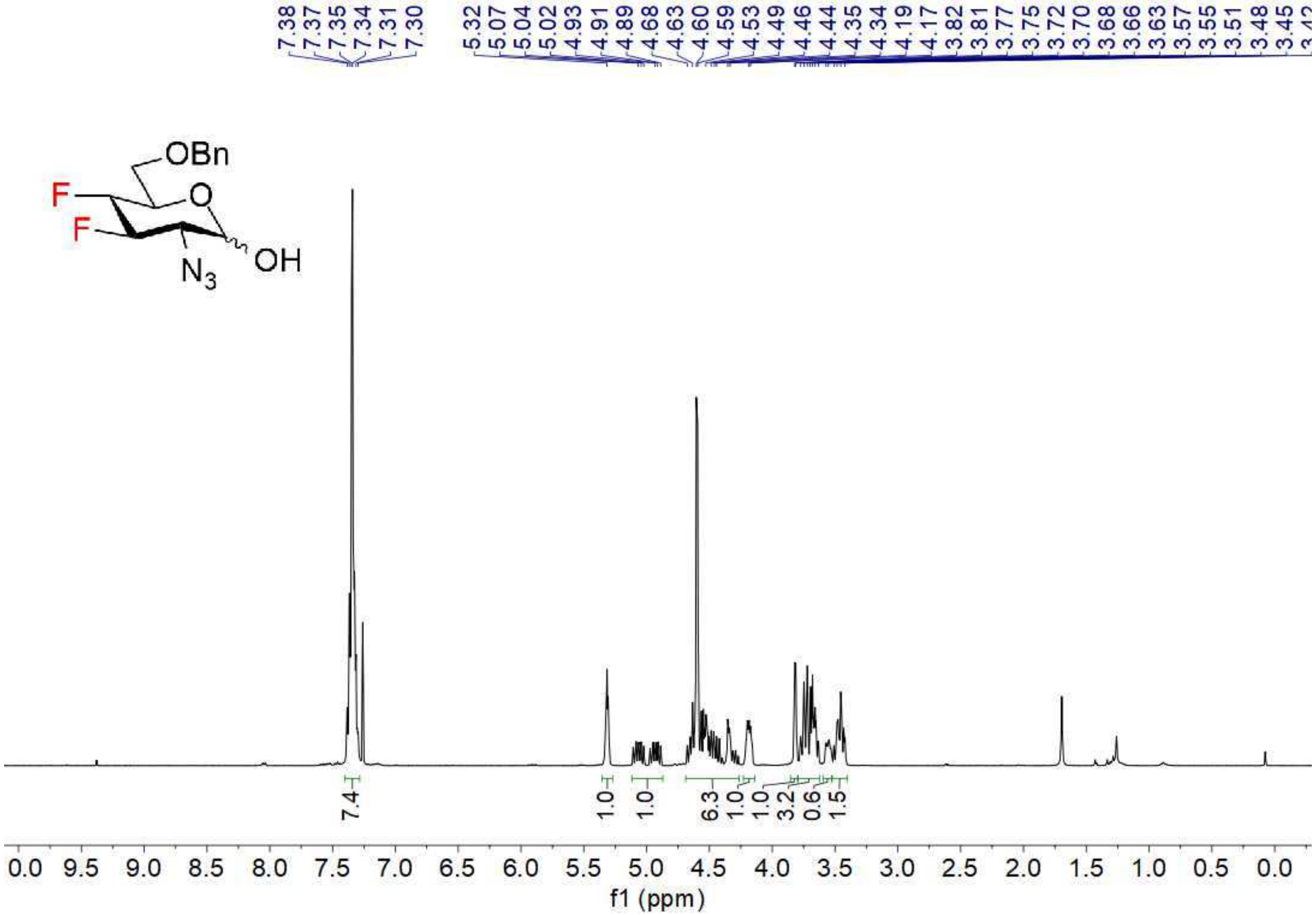

^{13}C NMR (100 MHz, CDCl$_3$) 47

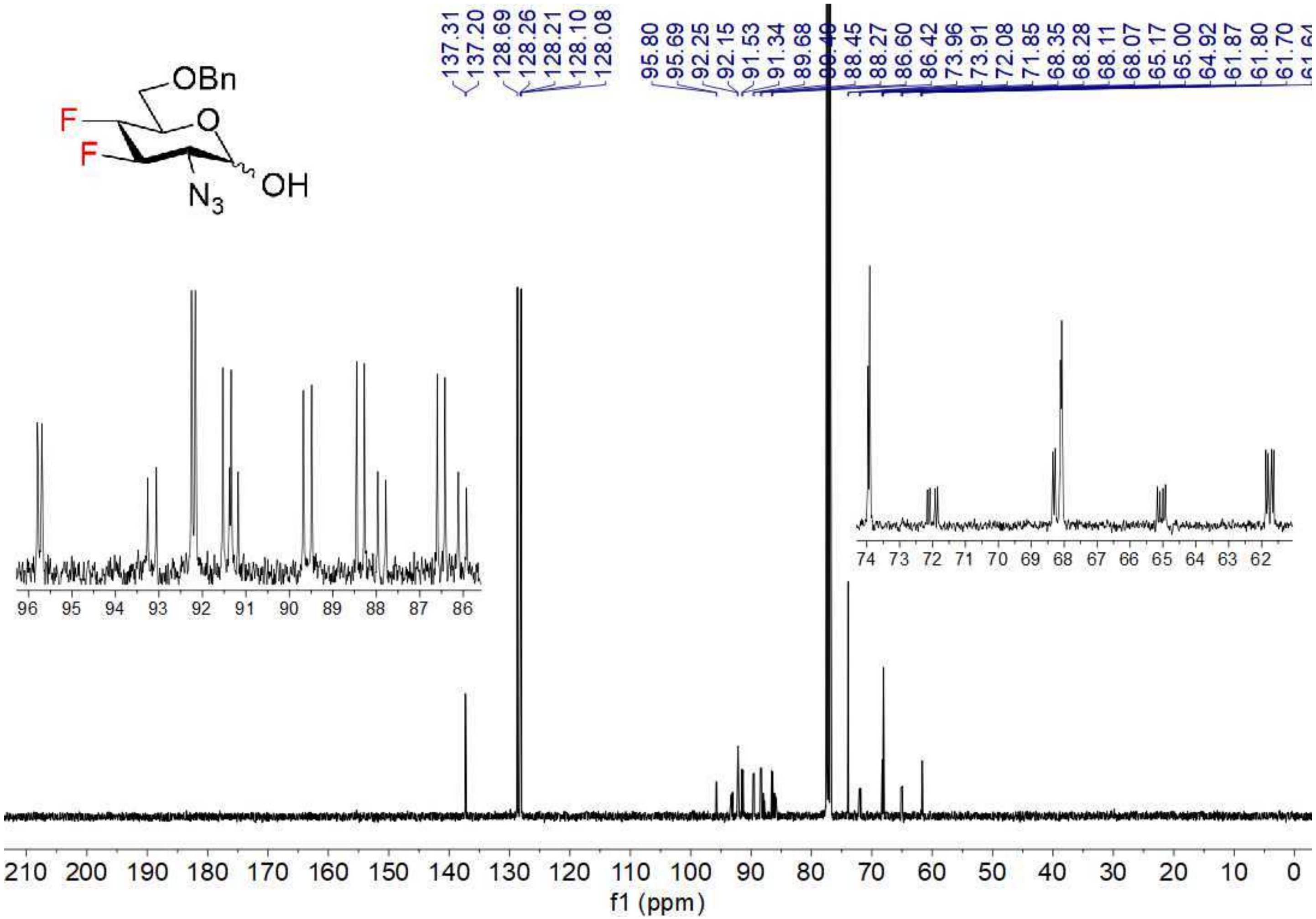

^{19}F NMR (376 MHz, $CDCl_3$) 47 (α/β ca. 2/1)

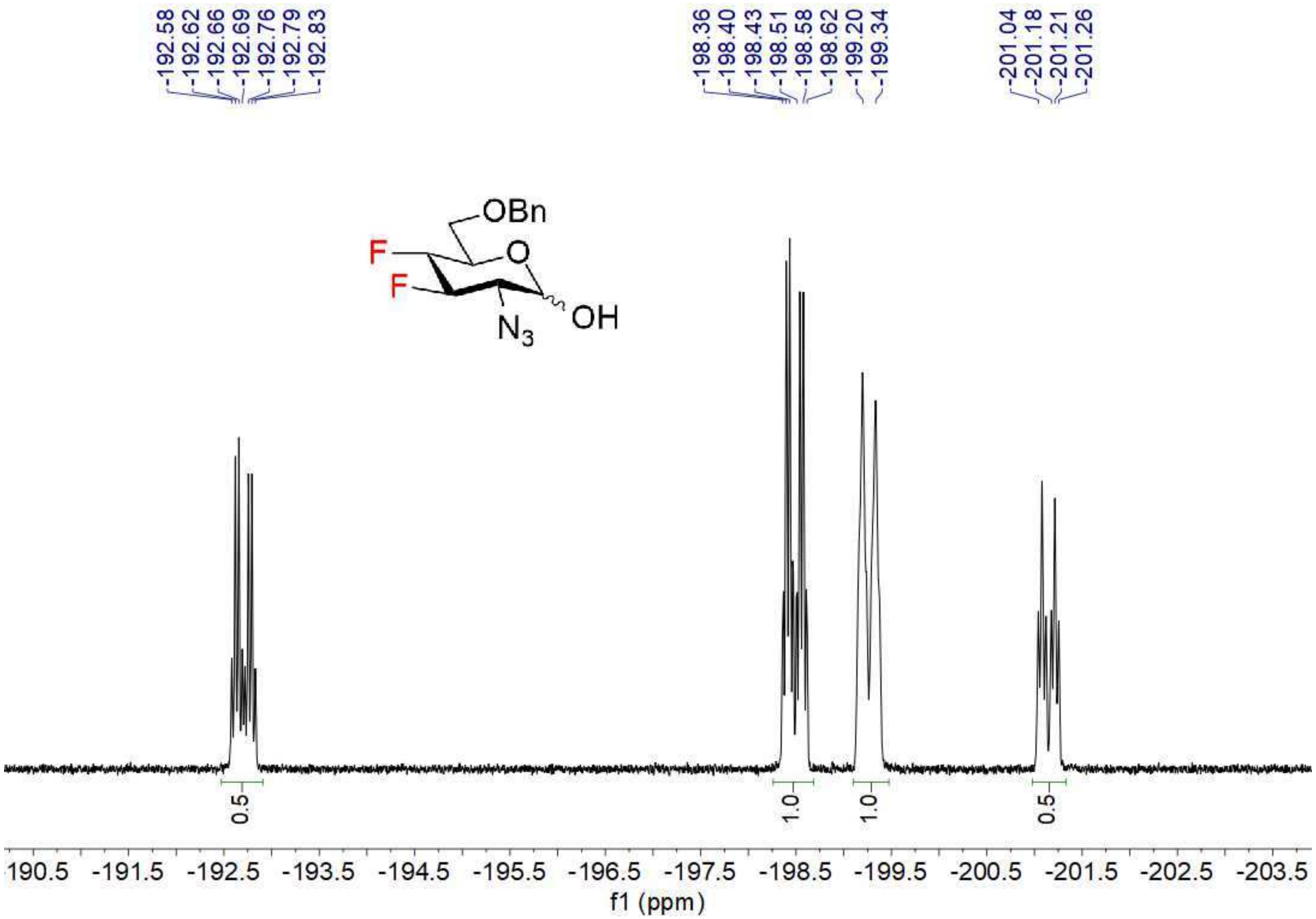

^{1}H-^{1}H COSY 47

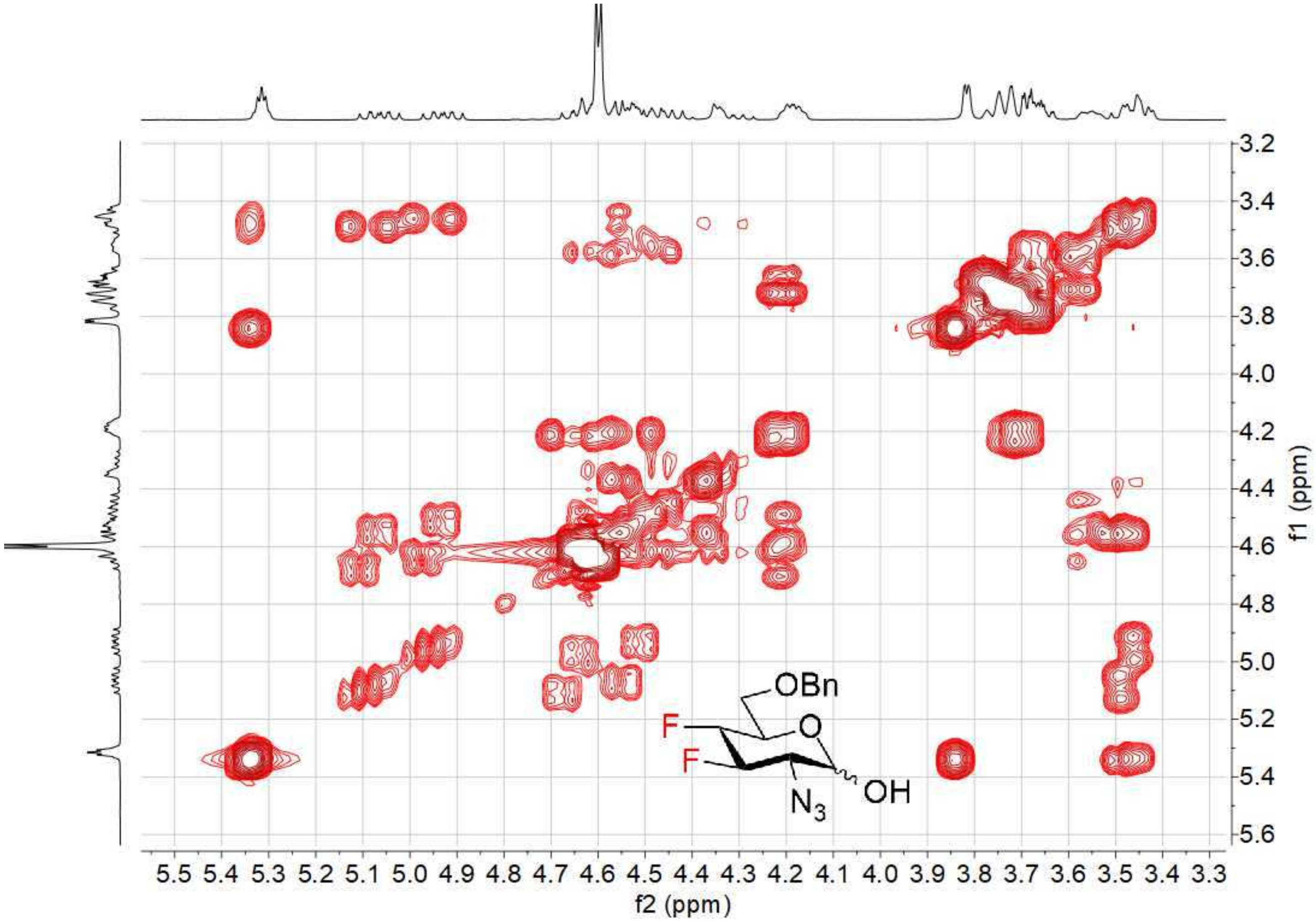

5.5 5.4 5.3 5.2 5.1 5.0 4.9 4.8 4.7 4.6 4.5 4.4 4.3 4.2 4.1 4.0 3.9 3.8 3.7 3.6 3.5 3.4 3.3
f2 (ppm)
3.2 3.4 3.6 3.8 4.0 4.2 4.4 4.6 4.8 5.0 5.2 5.4 5.6
f1 (ppm)
OBn
O
F
F
N3
OH

^{1}H-^{13}C HSQC 47

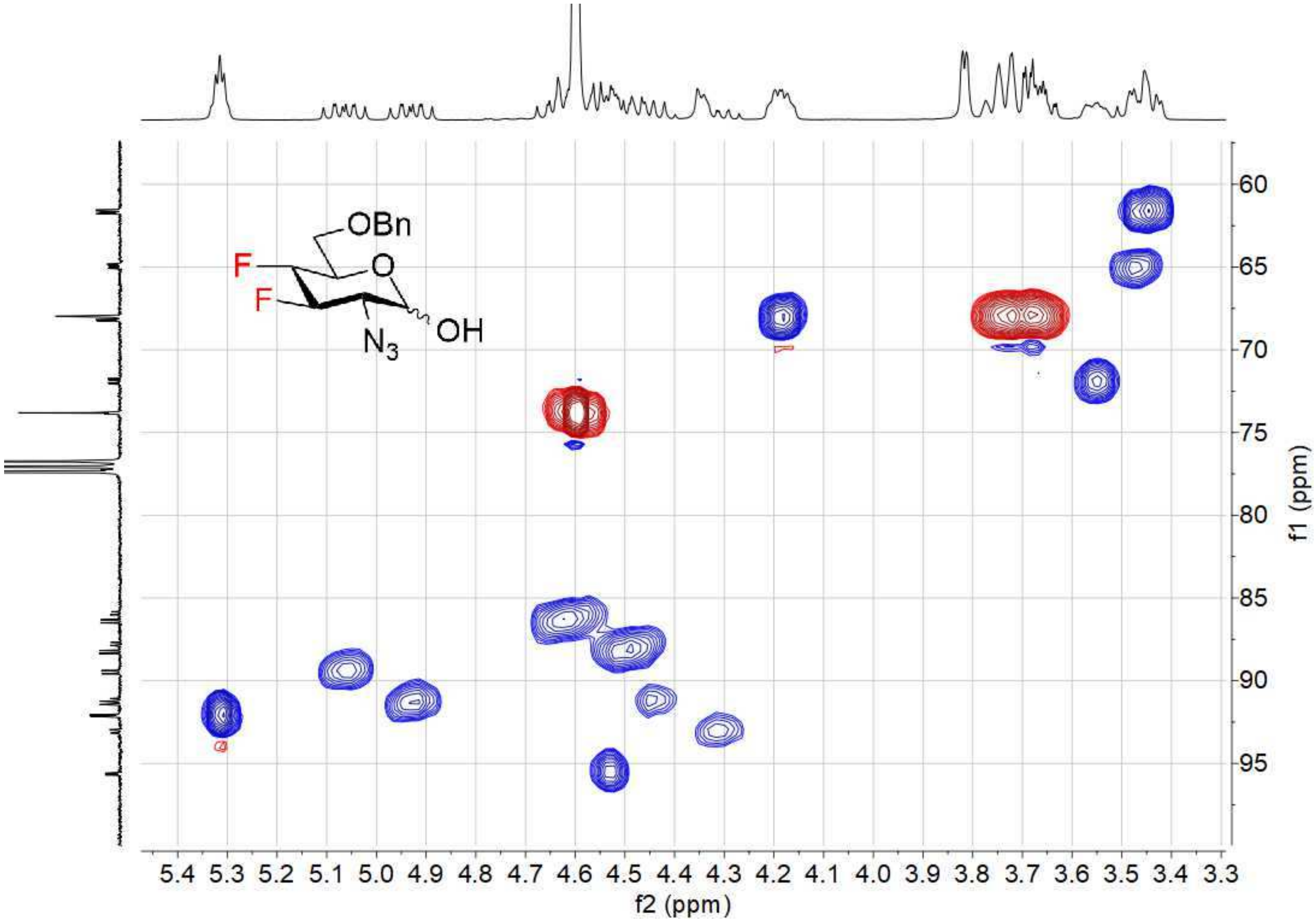

NMR COMPOUND 48

^{1}H NMR (400 MHz, $CDCl_3$) 48 (α/β ca. 2/1)

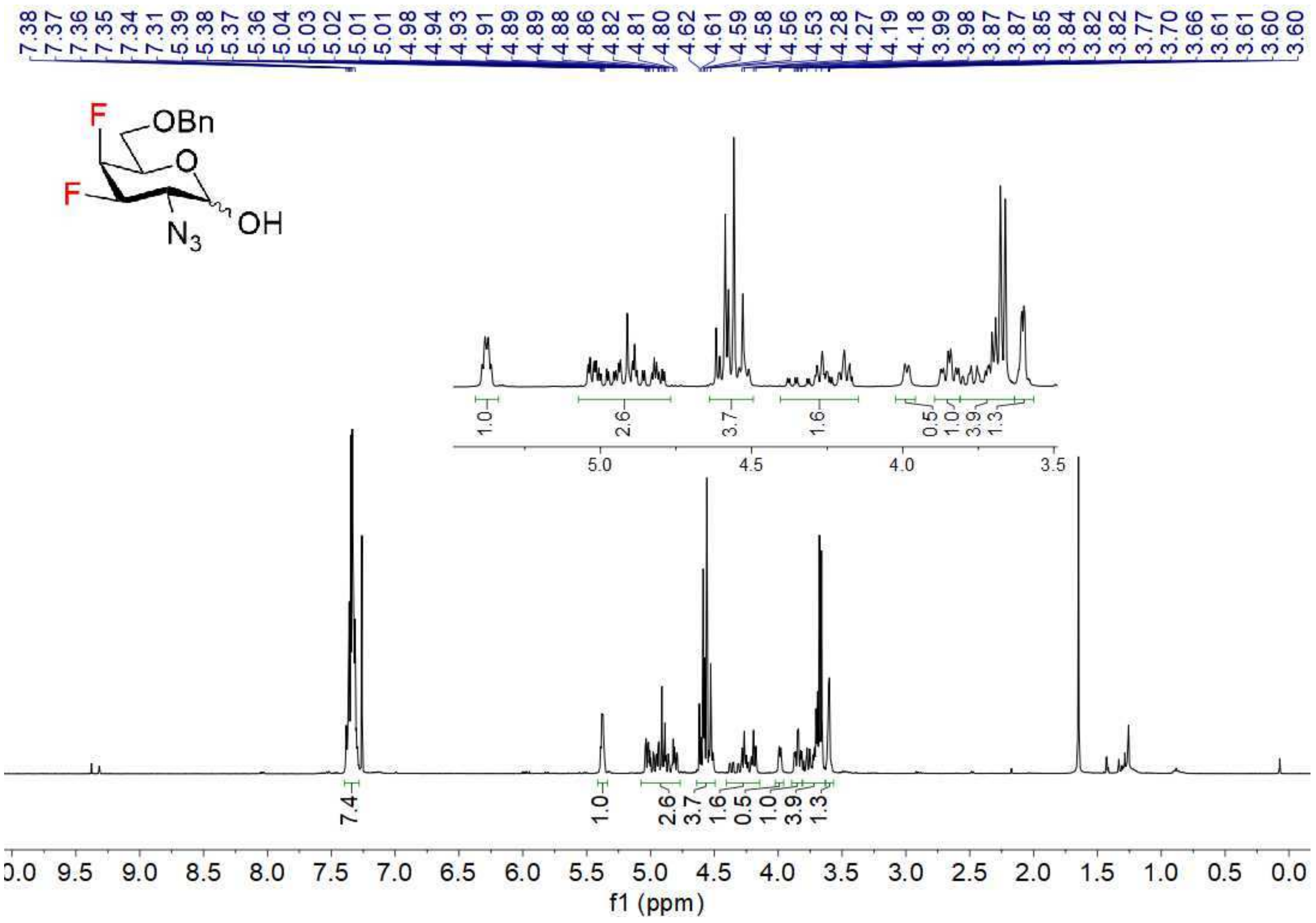

^{13}C NMR (100 MHz, CDCl$_3$) 48

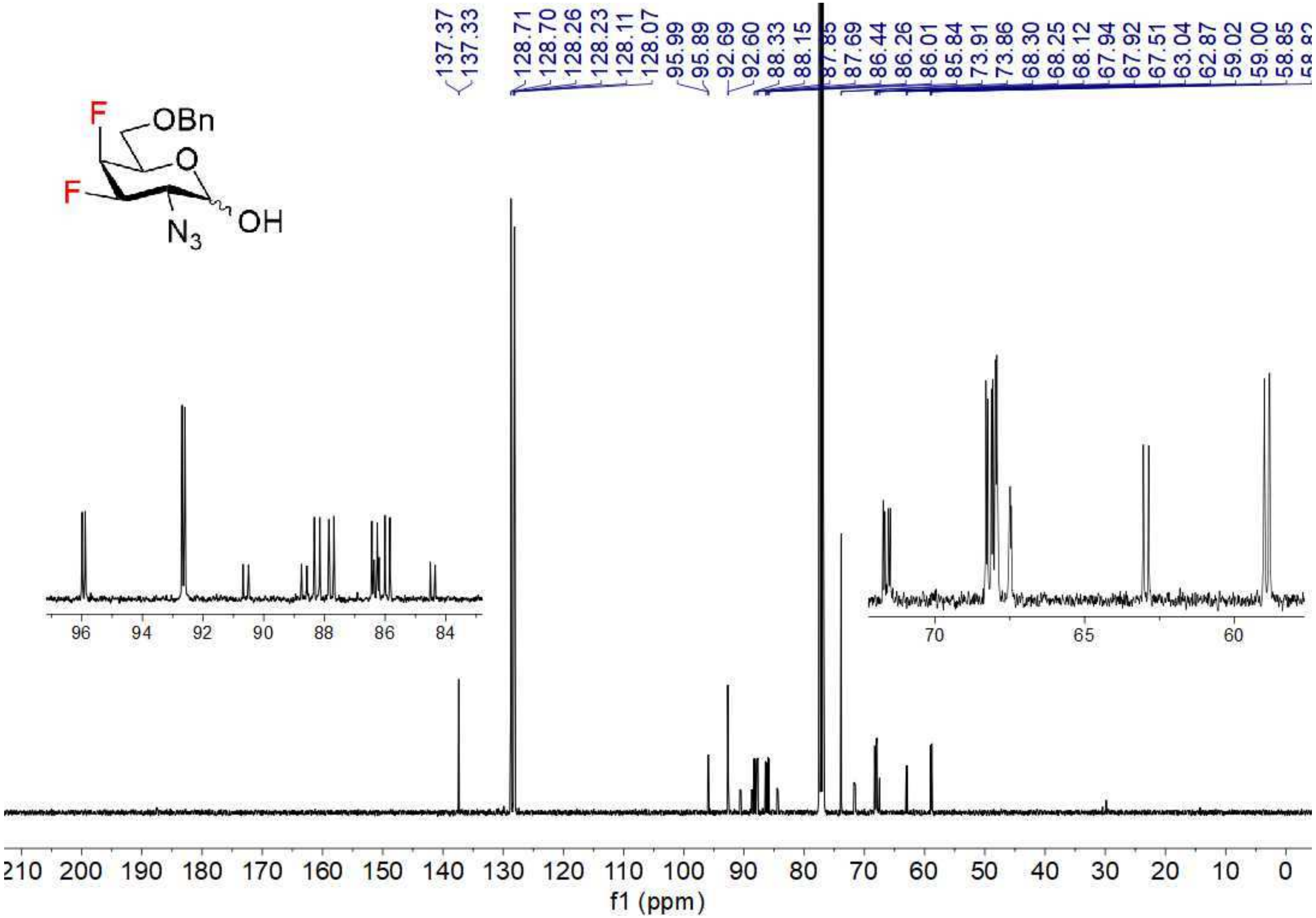

^{19}F NMR (376 MHz, CDCl$_3$) 48 (α/β ca. 2/1)

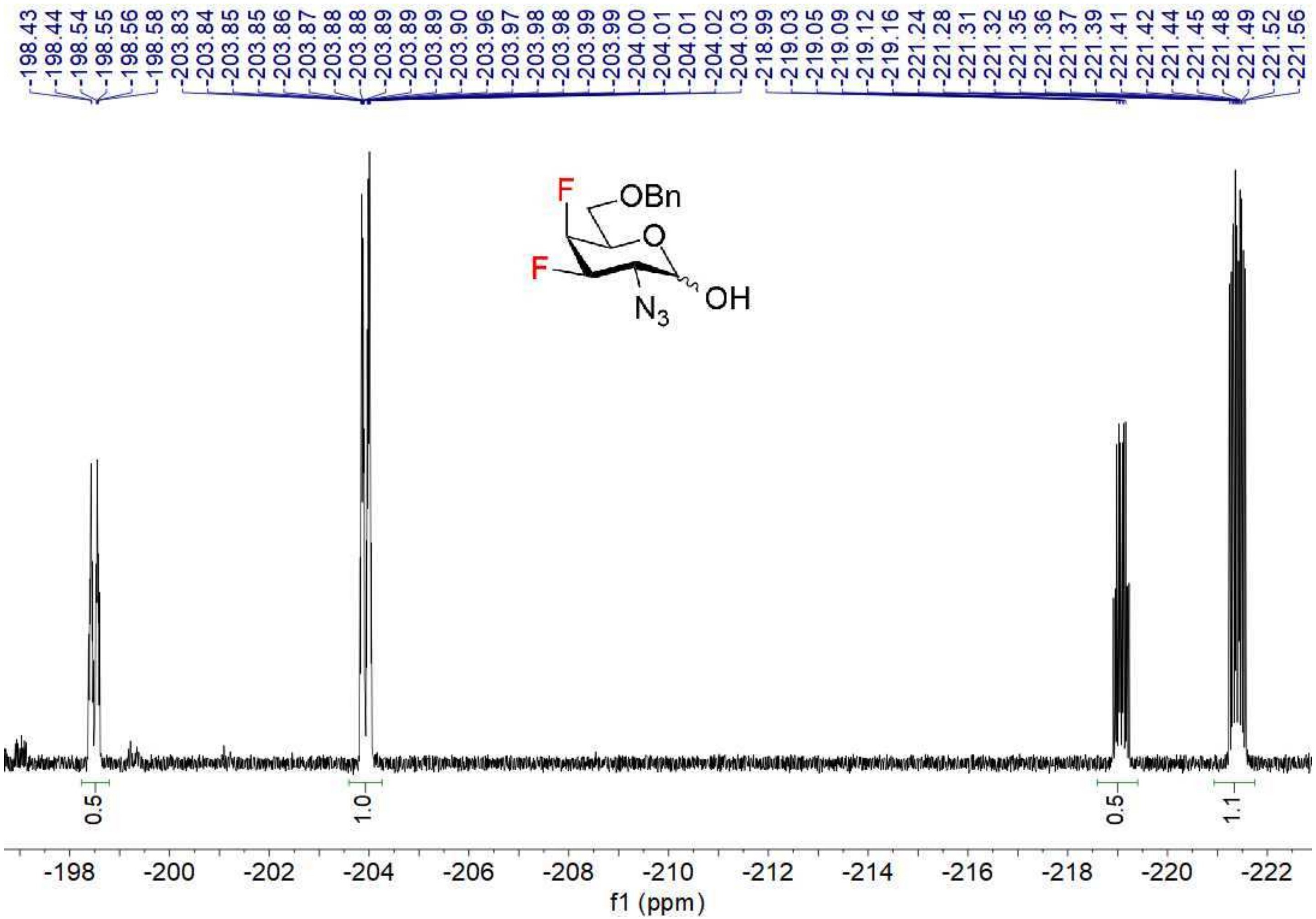

^{1}H-^{1}H COSY 48

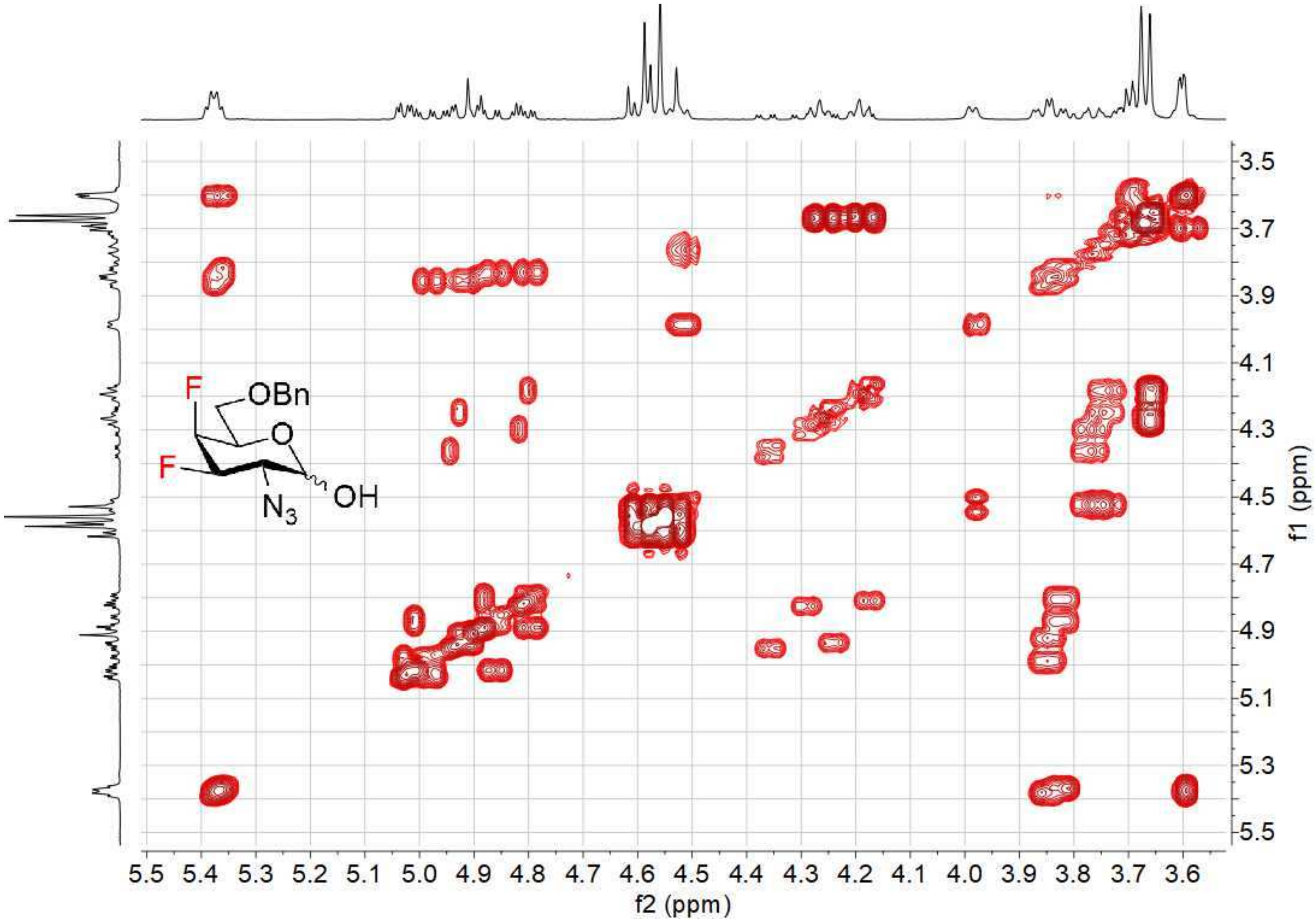

F
OBn
O
F
OH
N_3
3.5
3.7
3.9
4.1
4.3
4.5
4.7
4.9
5.1
5.3
5.5
f1 (ppm)
5.5 5.4 5.3 5.2 5.1 5.0 4.9 4.8 4.7 4.6 4.5 4.4 4.3 4.2 4.1 4.0 3.9 3.8 3.7 3.6
f2 (ppm)

^{1}H-^{13}C HSQC 48

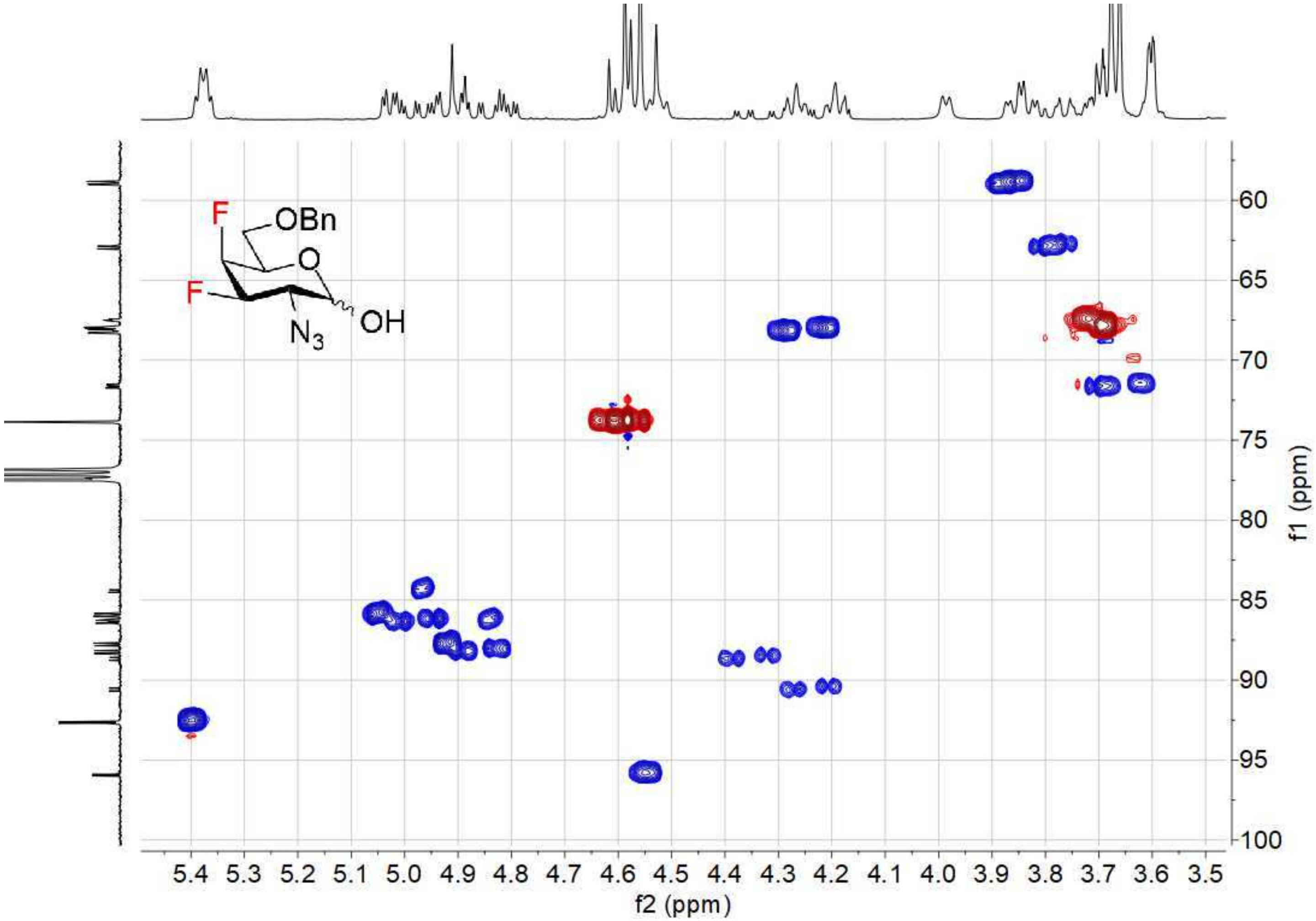

NMR COMPOUND 49

^{1}H NMR (400 MHz, CD_3OD) 49 (ca. 5% β-anomer)

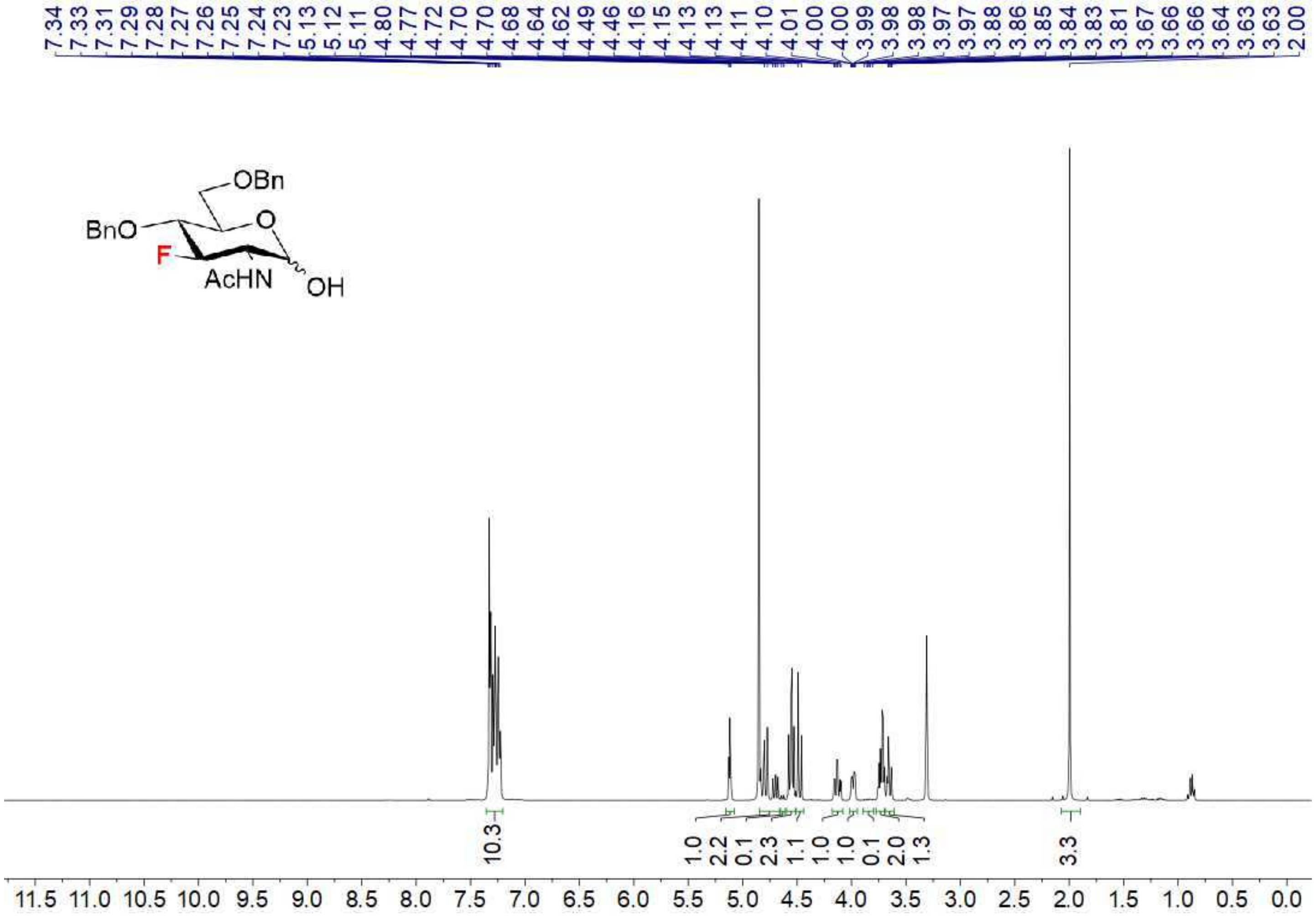

^{13}C NMR (100 MHz, CD_3OD) 49

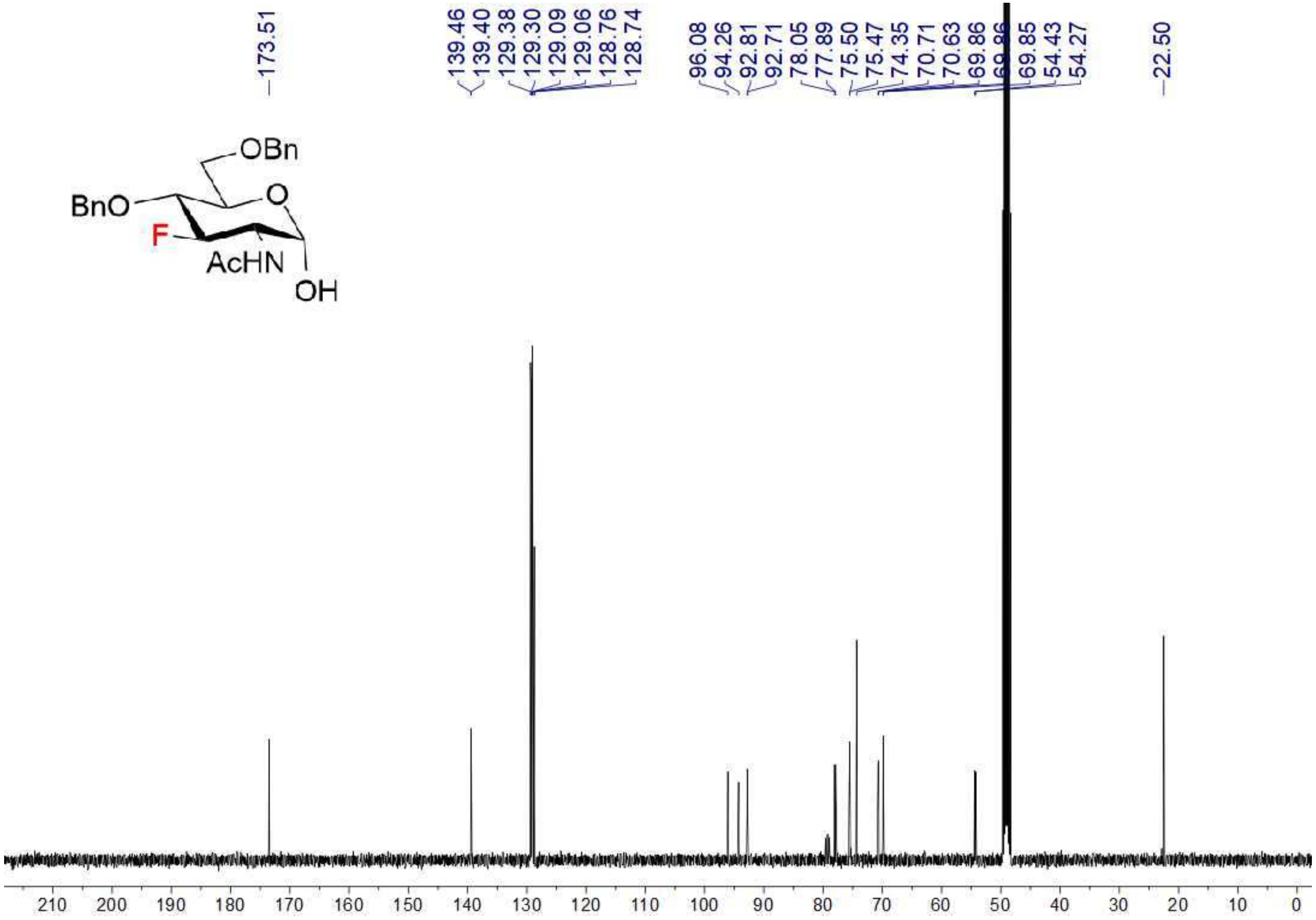

^{19}F NMR (376 MHz, CD_3OD) 49 (ca. 5% β-anomer)

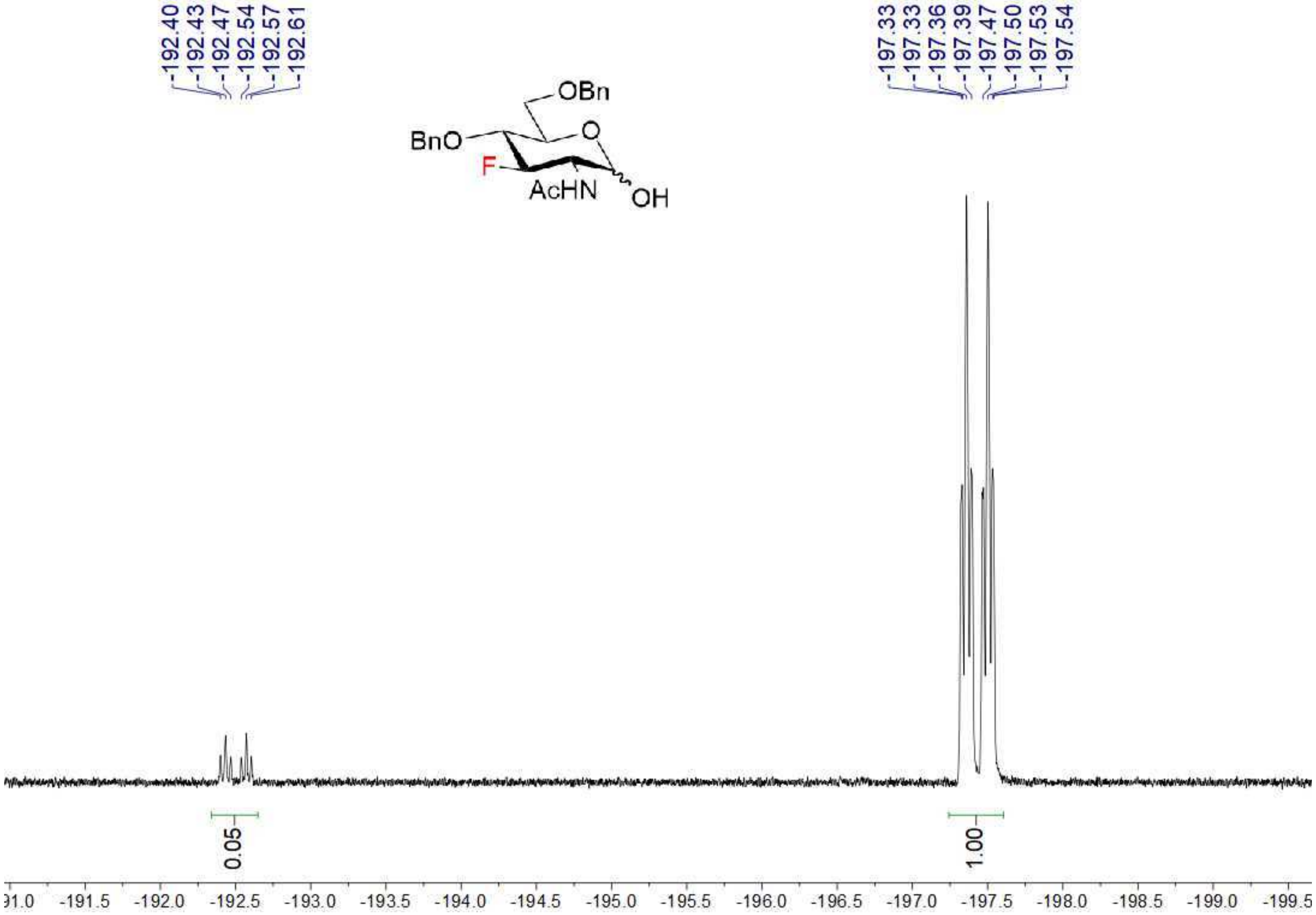

^{1}H-^{1}H COSY 49

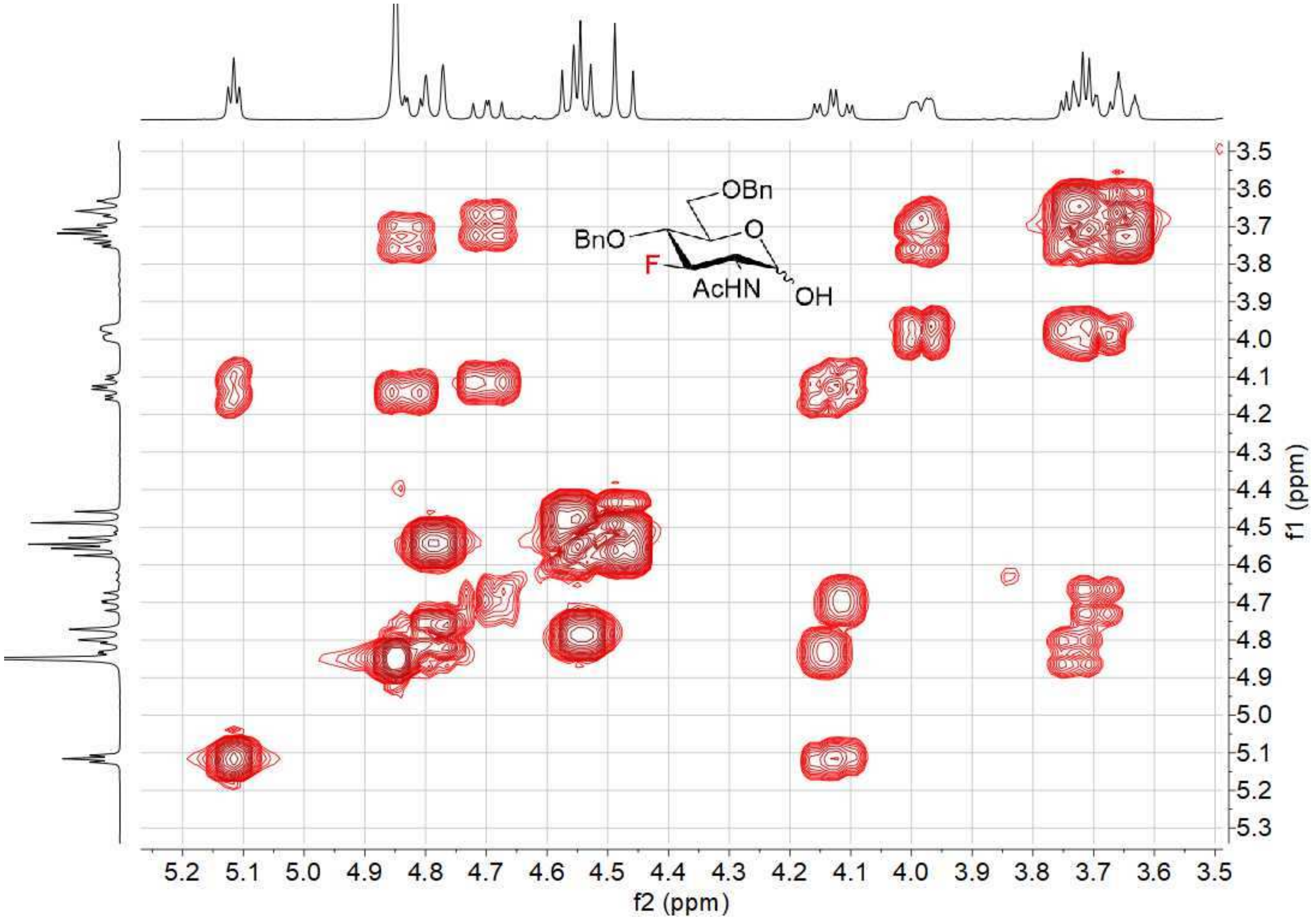

^{1}H-^{13}C HMBC 49

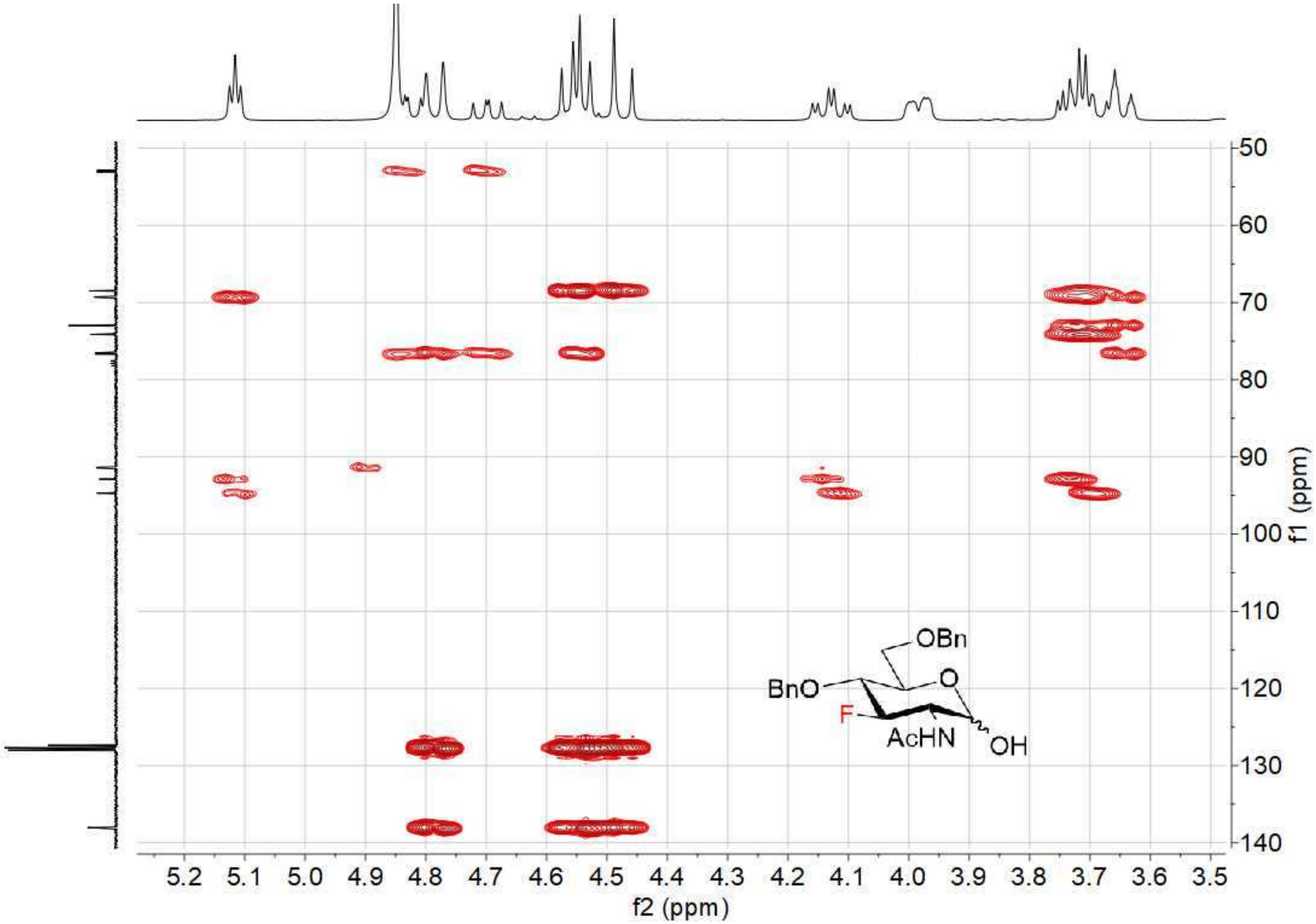

^{1}H-^{13}C HSQC 49

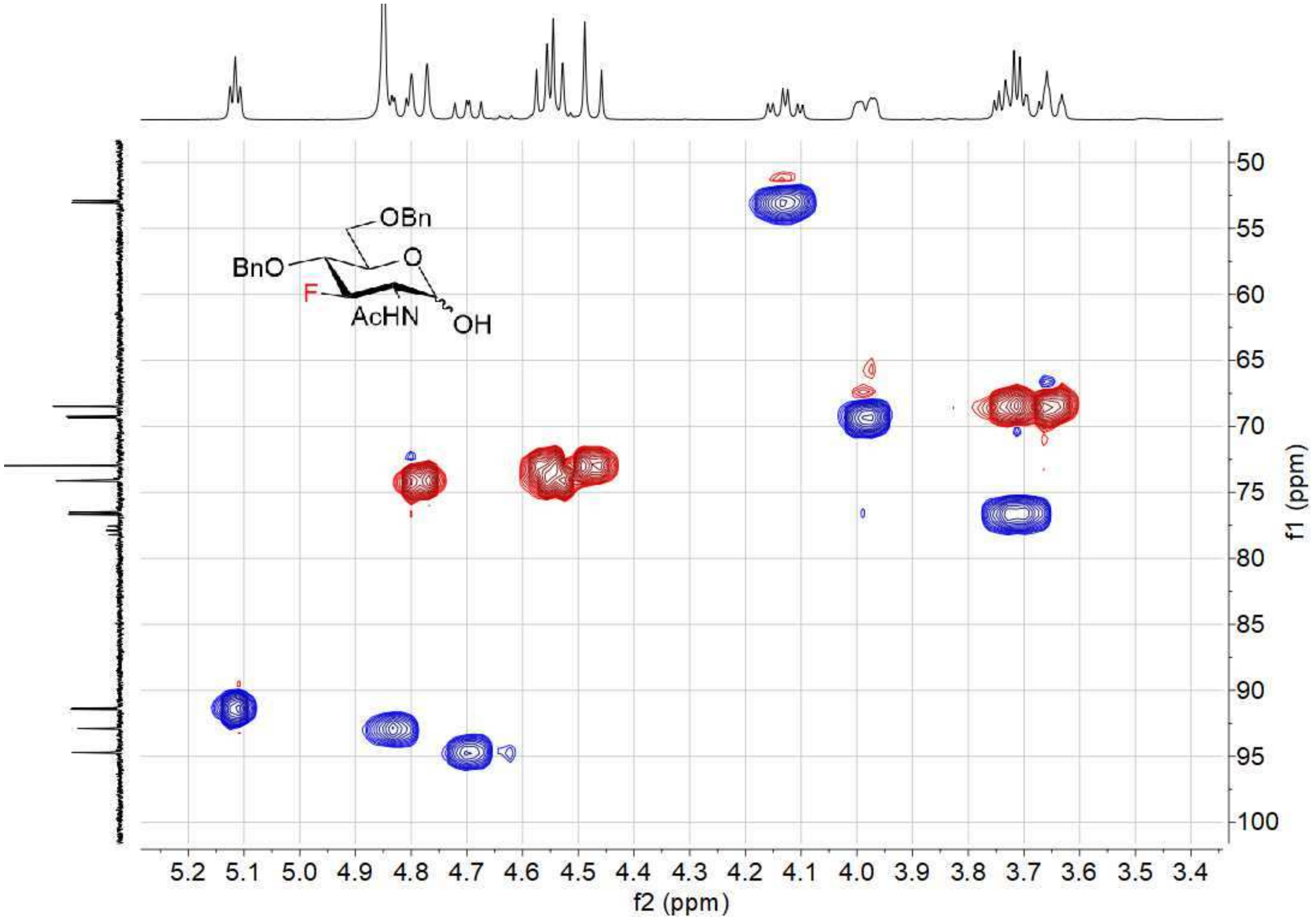

NMR COMPOUND 50

^{1}H NMR (400 MHz, CD_3OD) 50 (α/β ca. 10/1)

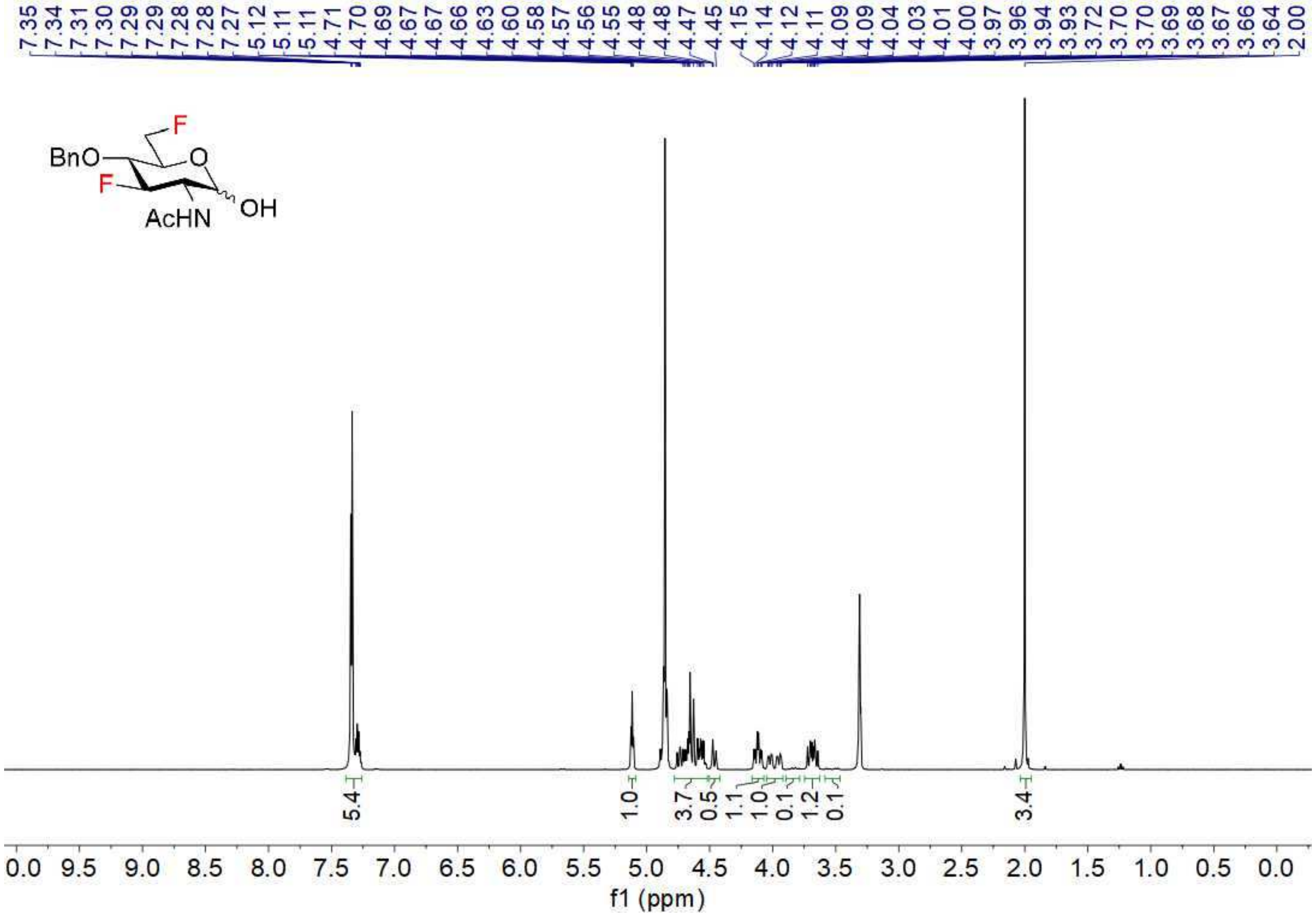

^{13}C NMR (100 MHz, CD$_3$OD) 50

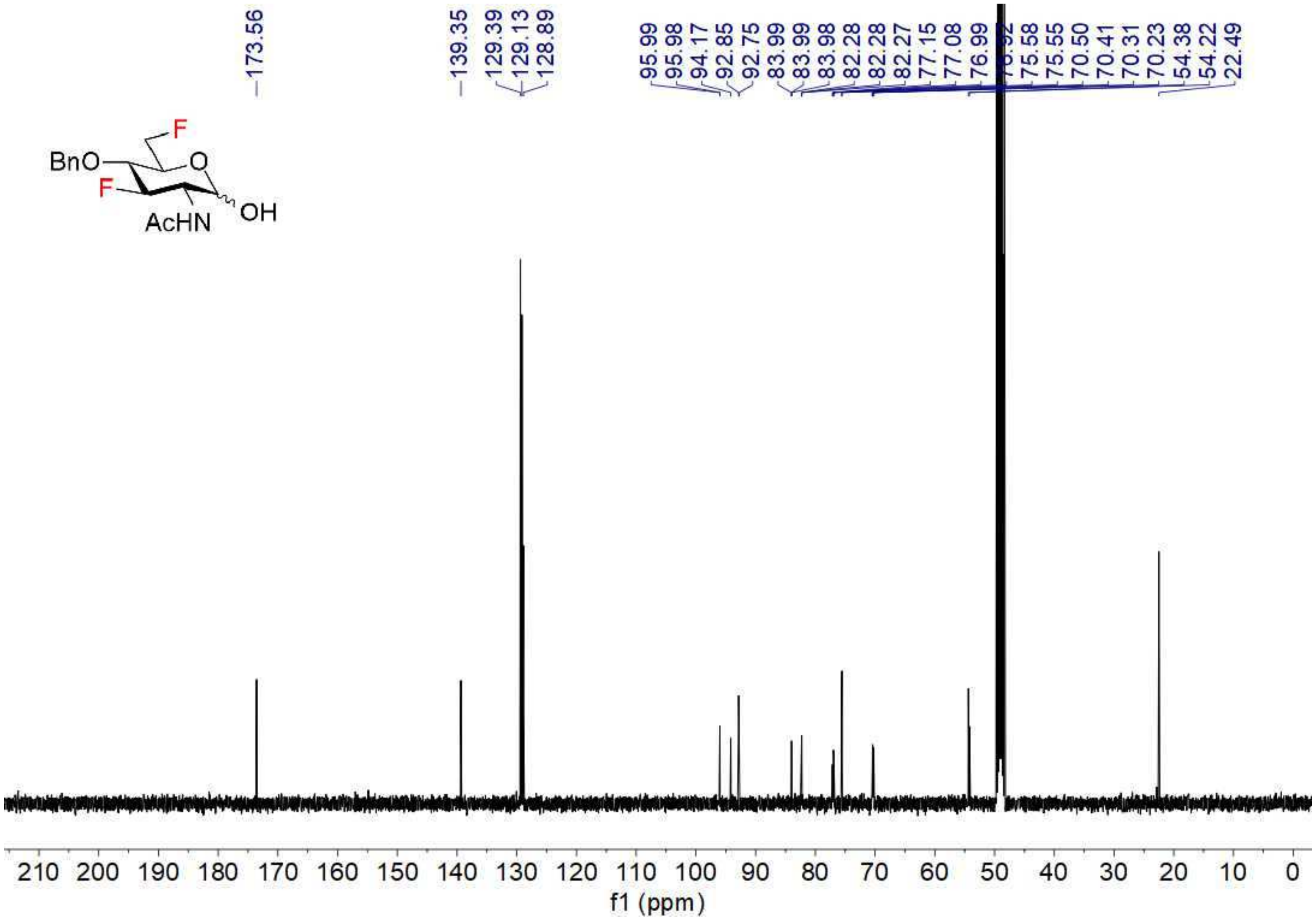

^{19}F NMR (376 MHz, CD$_3$OD) 50 (α/β ca. 10/1)

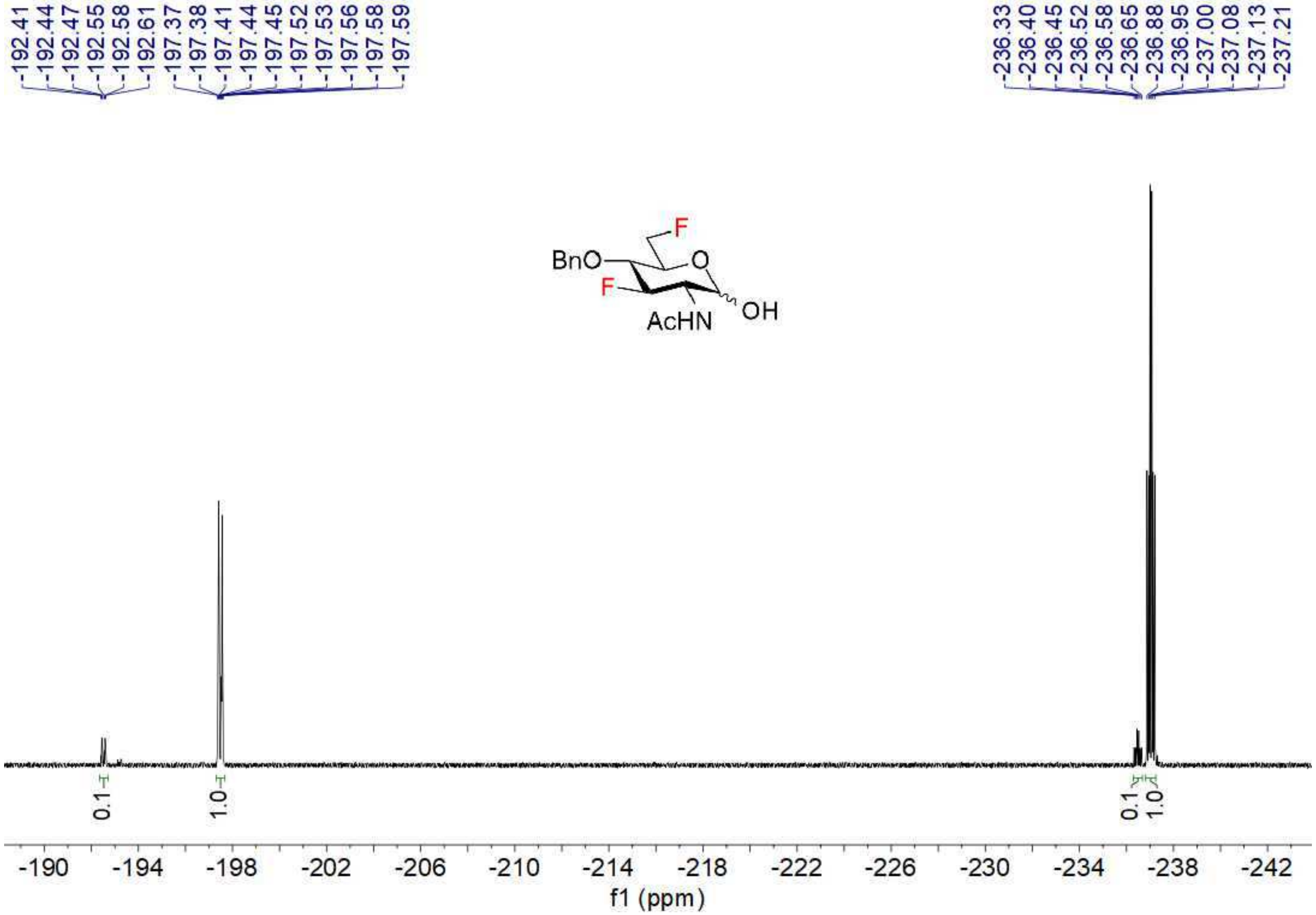

^{1}H-^{1}H COSY 50

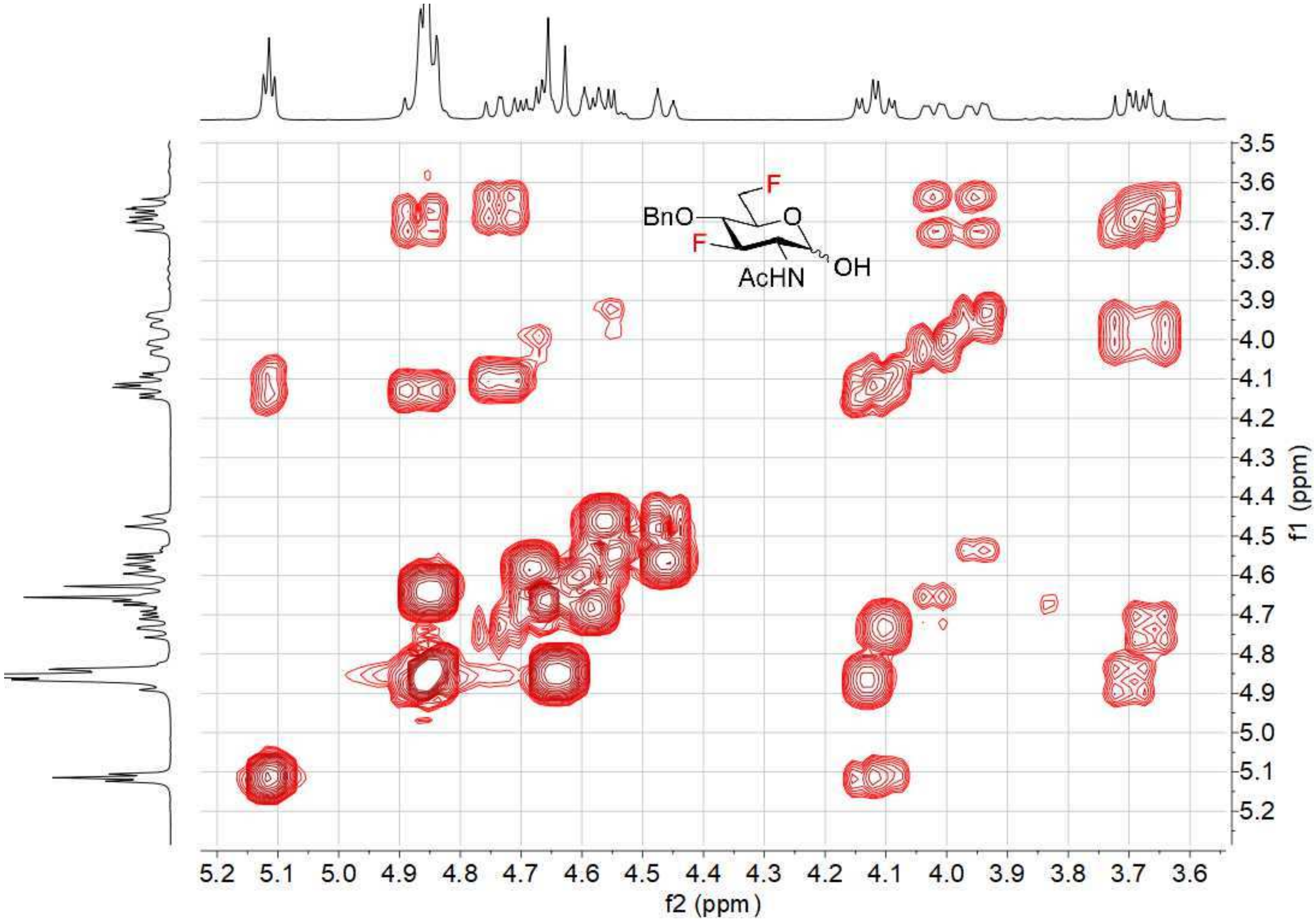

^{1}H-^{13}C HMBC 50

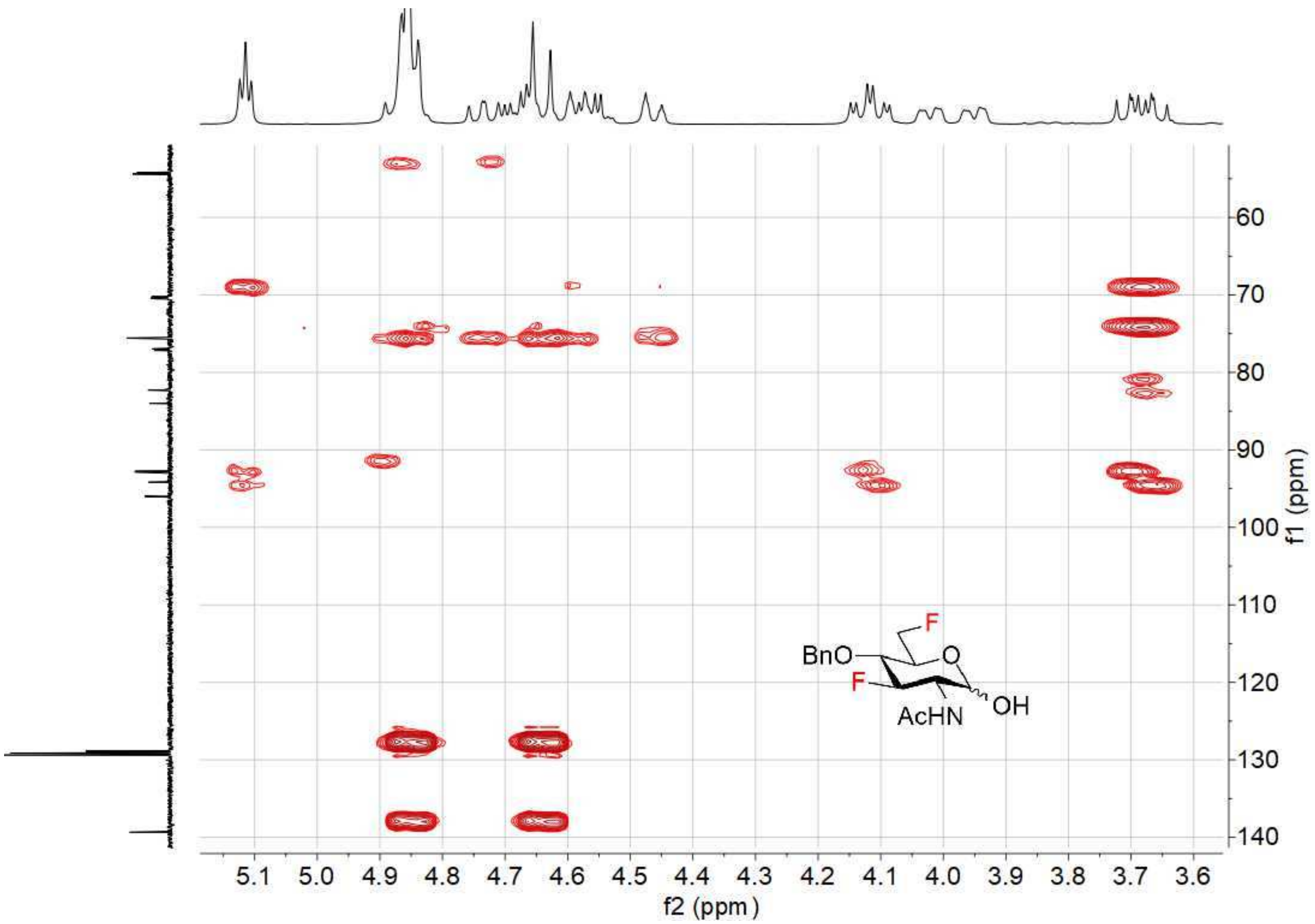

^{1}H-^{13}C HSQC 50

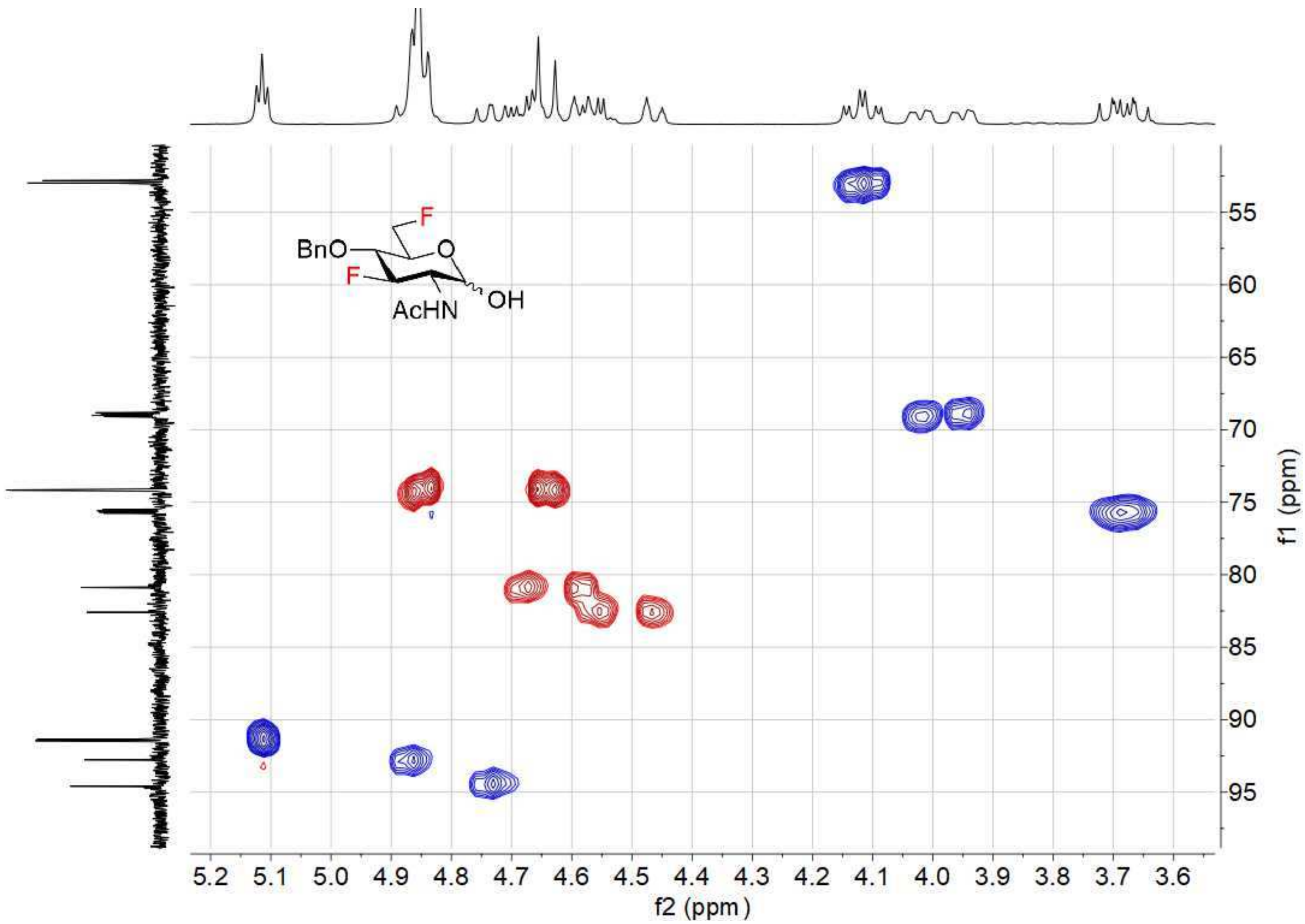

NMR COMPOUND 51

^{1}H NMR (400 MHz, CD_3OD) 51 (α/β ca. 10/1)

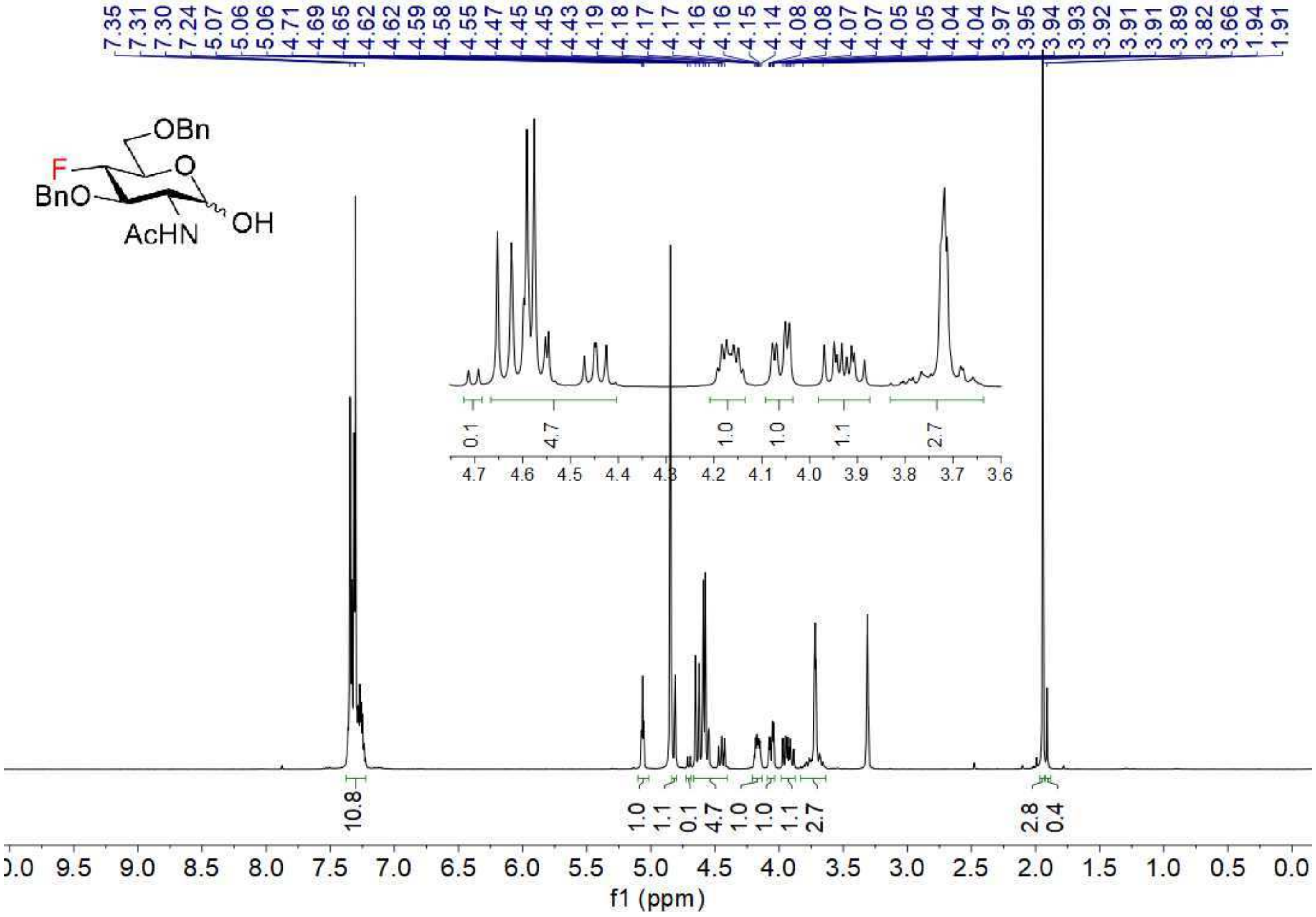

^{13}C NMR (100 MHz, CD_3OD) 51

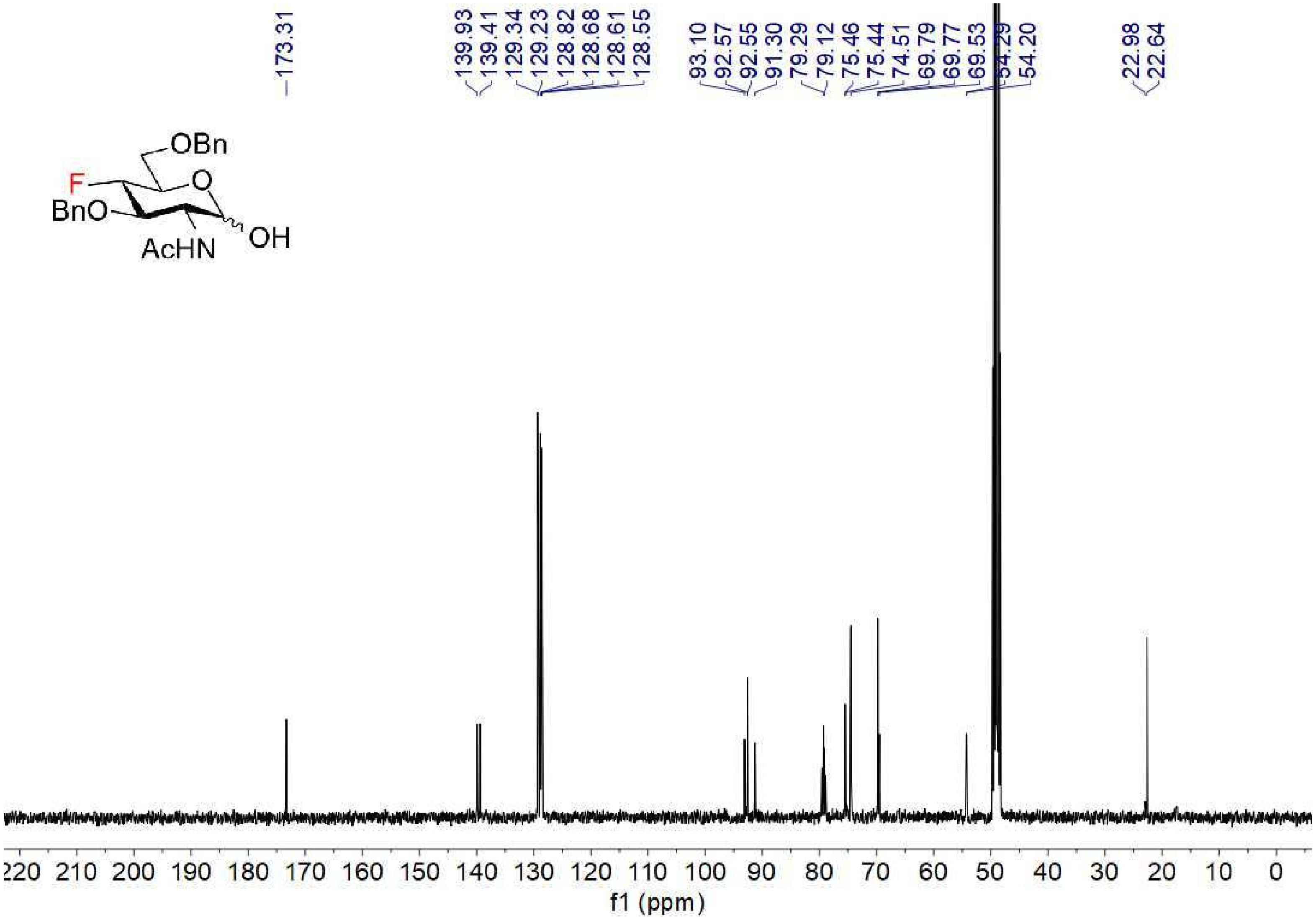

^{19}F NMR (376 MHz, CD_3OD) 51 (α/β ca. 10/1)

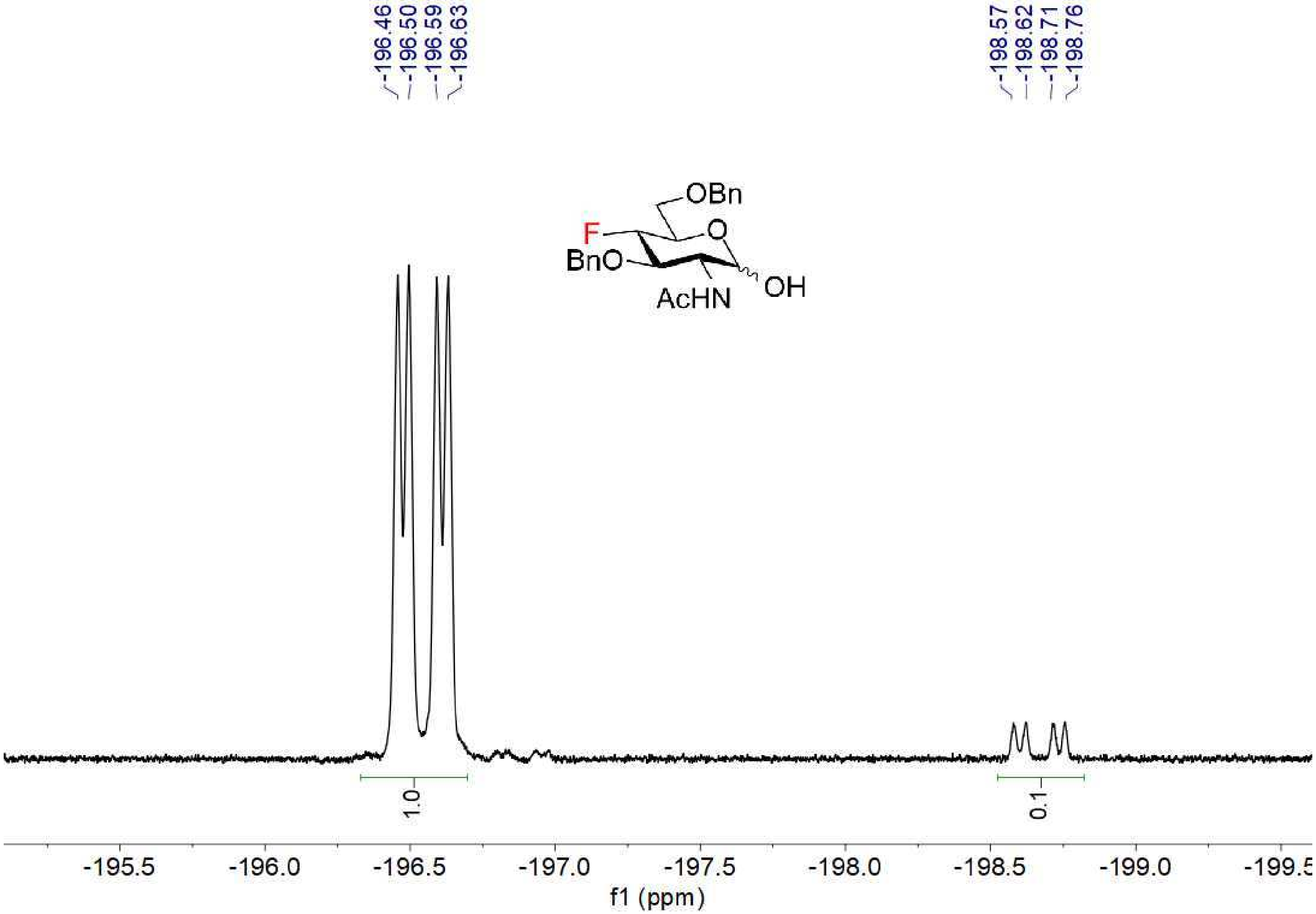

^{1}H-^{1}H COSY 51

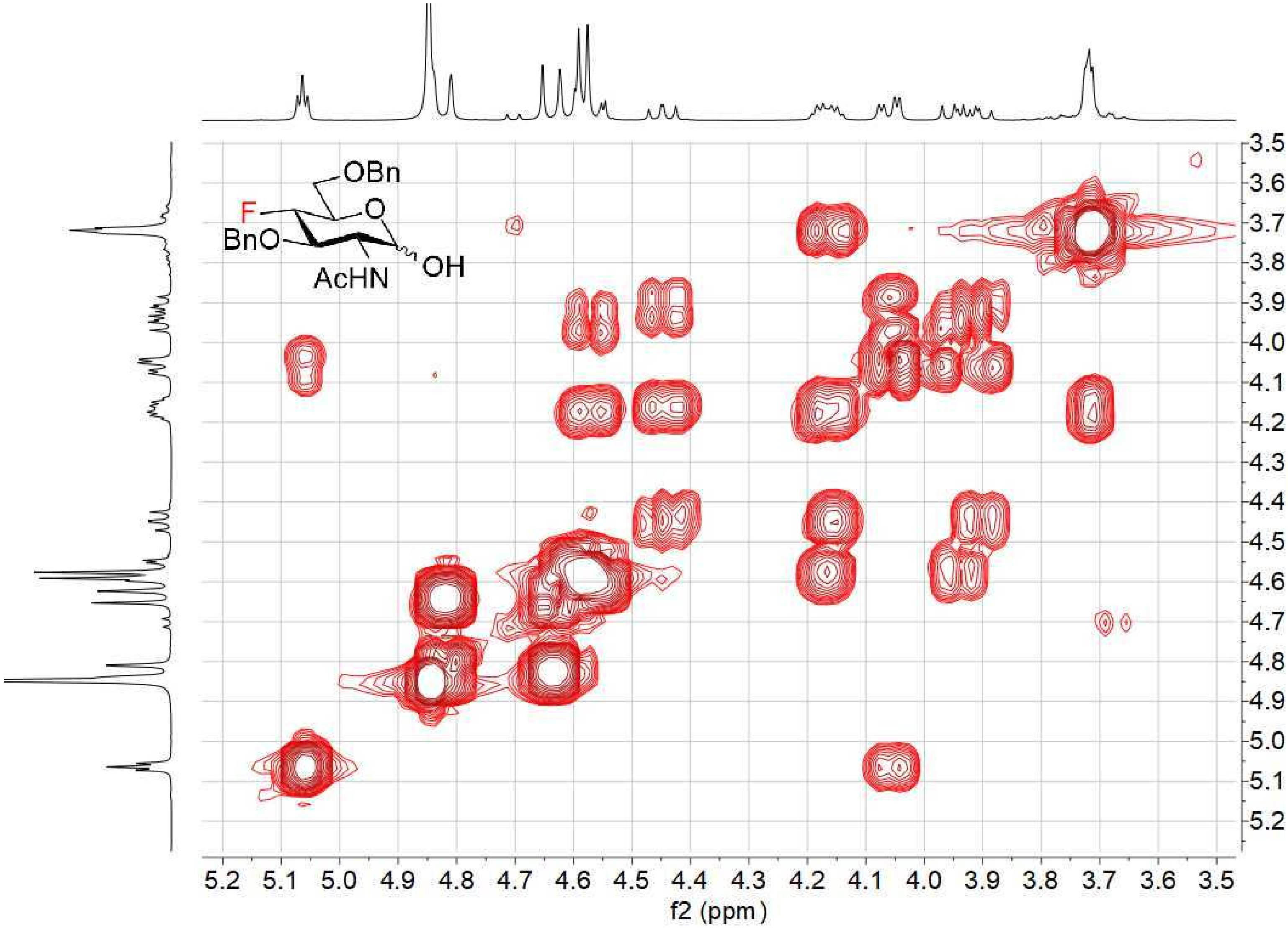
OBn
F
O
BnO
OH
AcHN
3.5
3.6
3.7
3.8
3.9
4.0
4.1
4.2
4.3
4.4
4.5
4.6
4.7
4.8
4.9
5.0
5.1
5.2
f1 (ppm)
5.2 5.1 5.0 4.9 4.8 4.7 4.6 4.5 4.4 4.3 4.2 4.1 4.0 3.9 3.8 3.7 3.6 3.5
f2 (ppm)

^{1}H-^{13}C HMBC 51

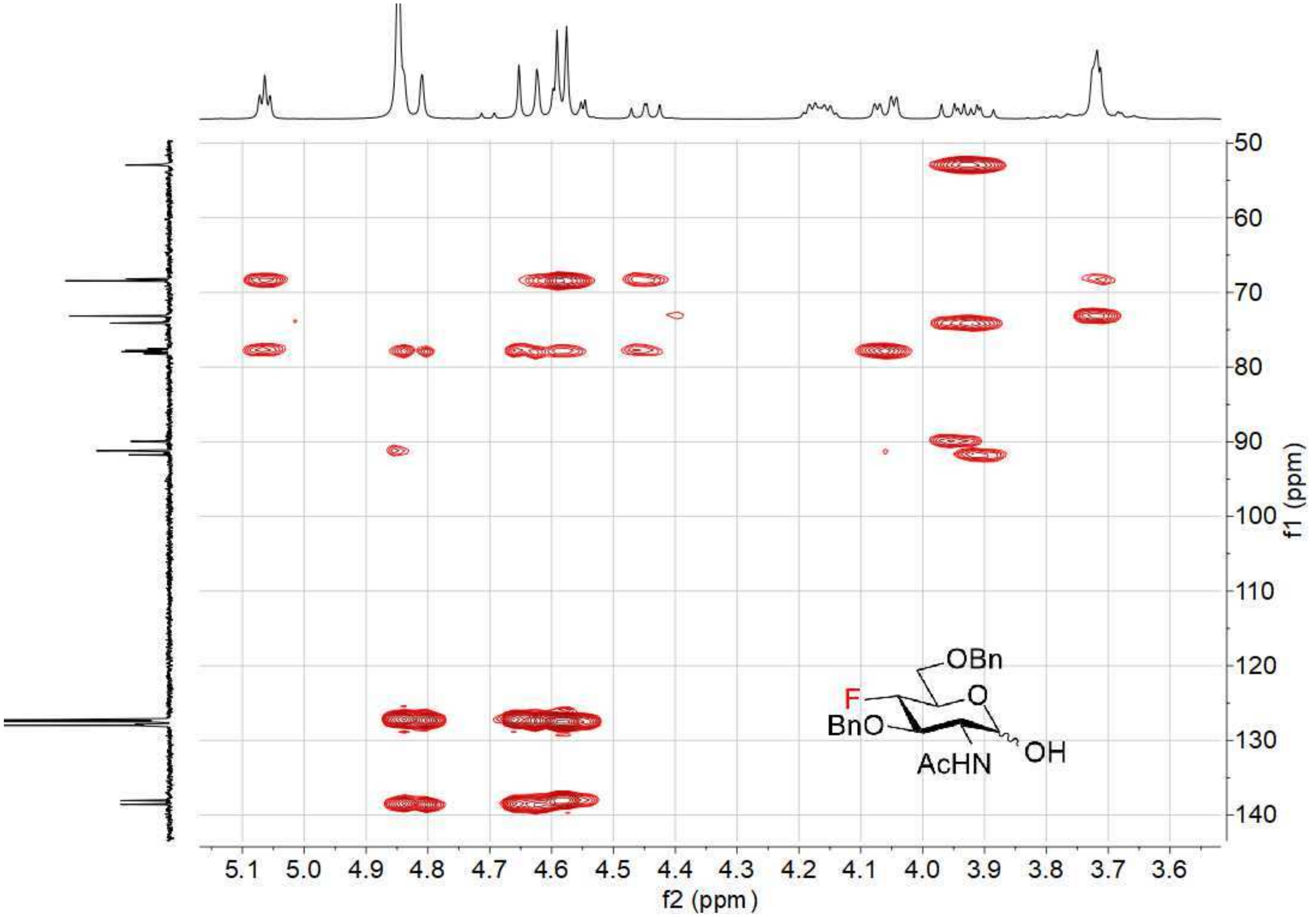

^{1}H-^{13}C HSQC 51

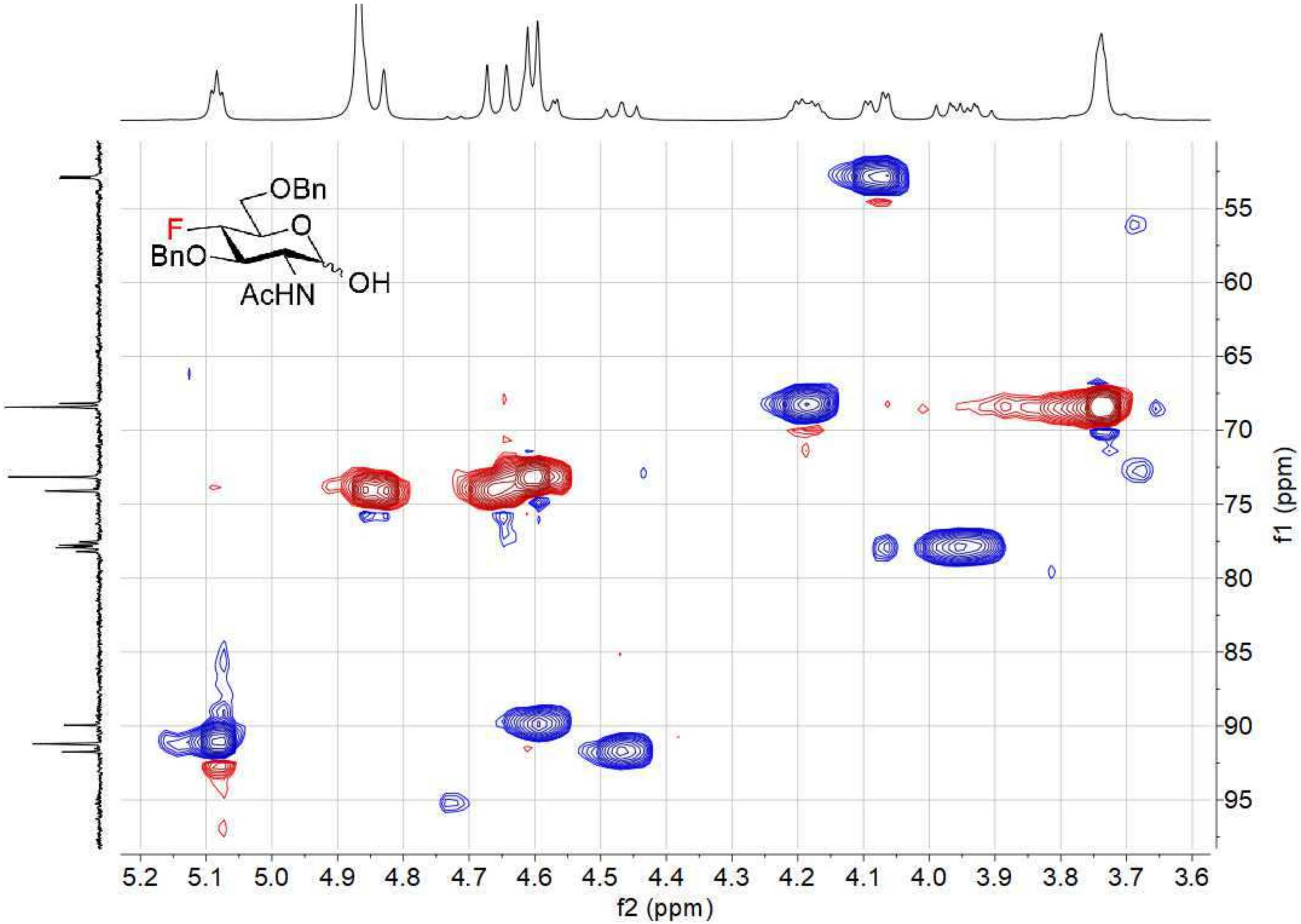

NMR COMPOUND 52

^{1}H NMR (400 MHz, CD_3OD) 52 (ca. 4% β-anomer)

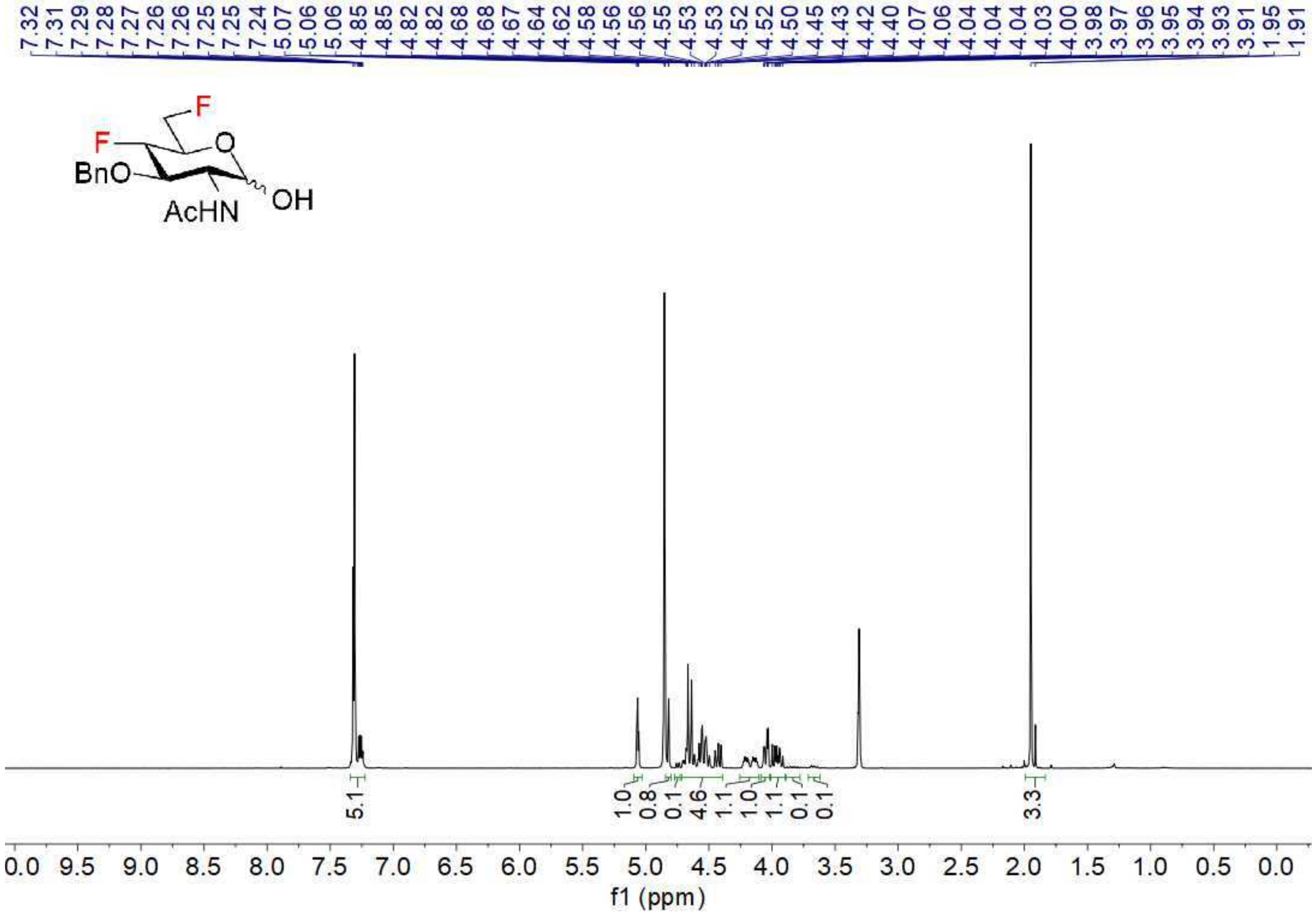

^{13}C NMR (100 MHz, CD_3OD) 52

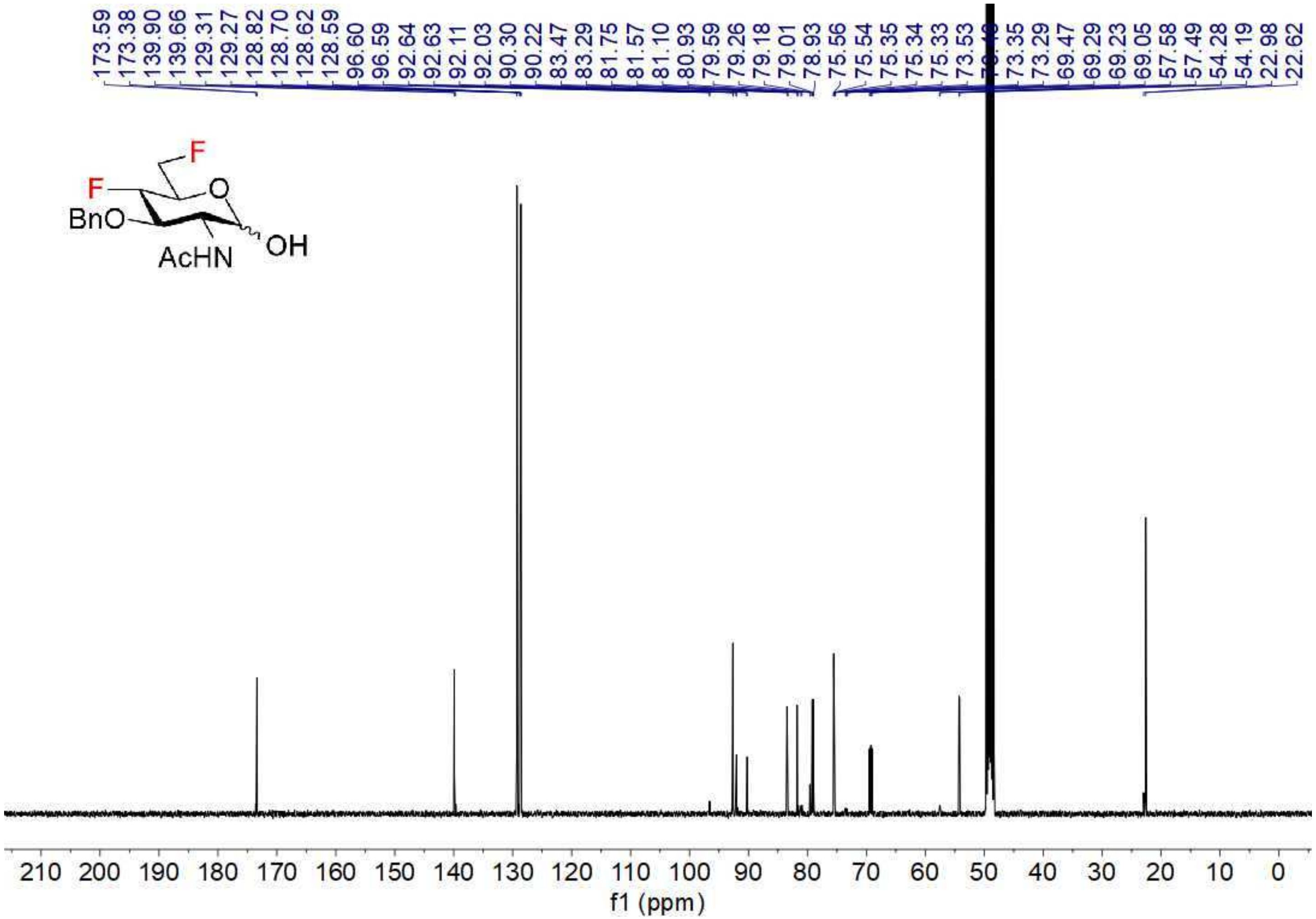

^{19}F NMR (376 MHz, CD$_3$OD) 52 (ca. 4% β-anomer)

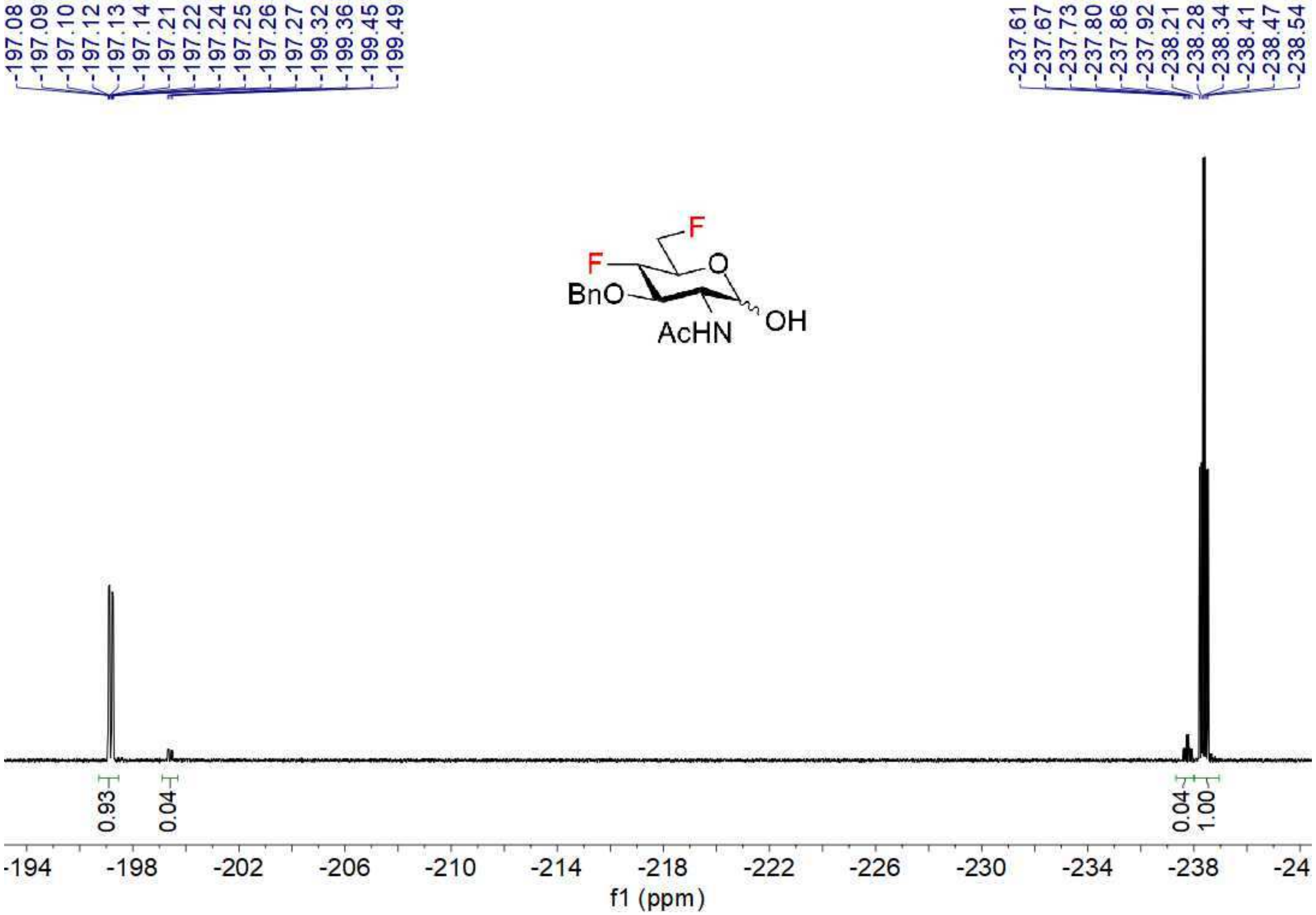

^{1}H-^{1}H COSY 52

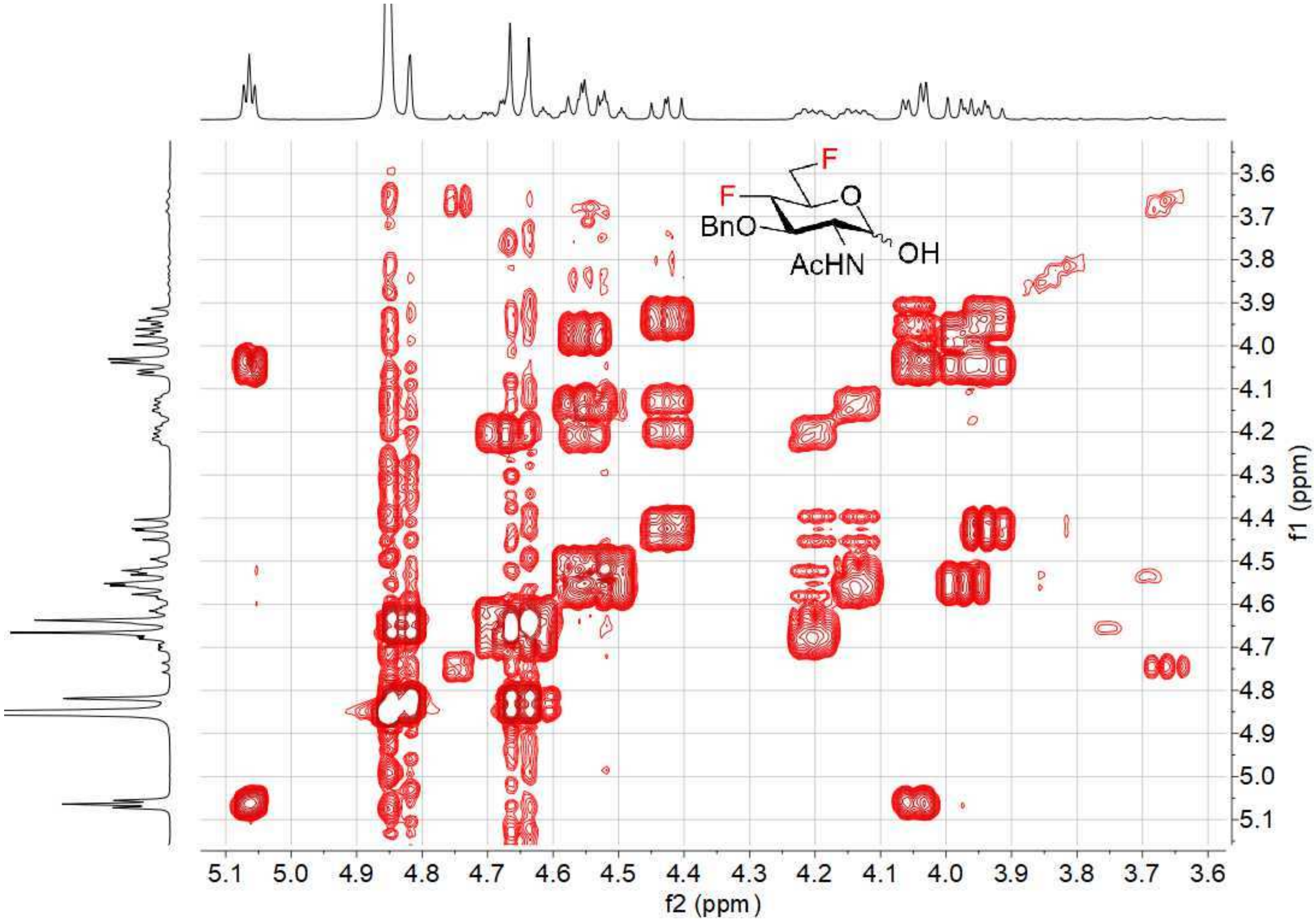
F
F
O
BnO
AcHN
OH
5.1
5.0
4.9
4.8
4.7
4.6
4.5
4.4
4.3
4.2
4.1
4.0
3.9
3.8
3.7
3.6
f2 (ppm)
f1 (ppm)

^{1}H-^{13}C **HSQC 52**

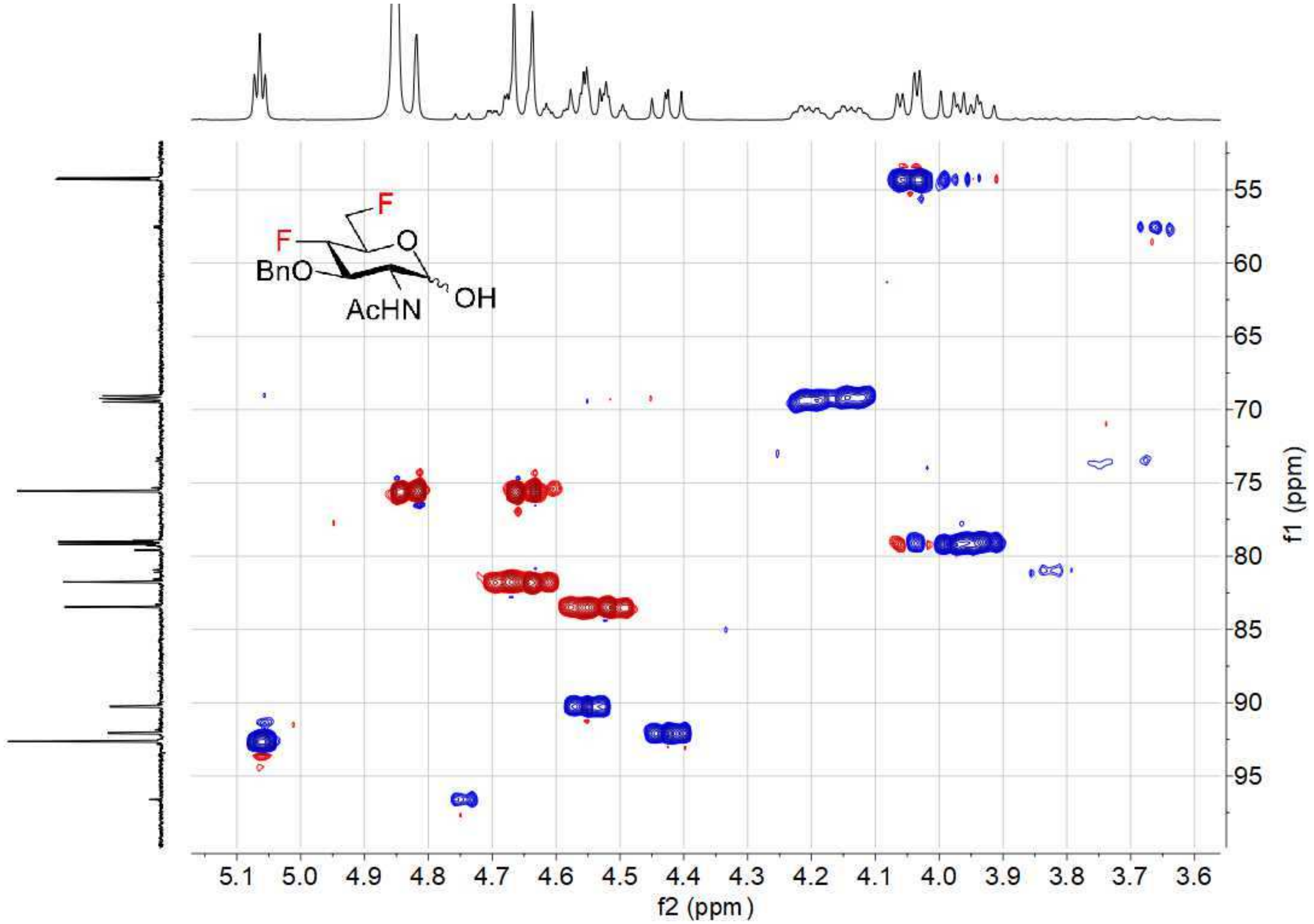

NMR COMPOUND 53

^{1}H NMR (400 MHz, CD_3OD) 53 (α/β ca. 10/1)

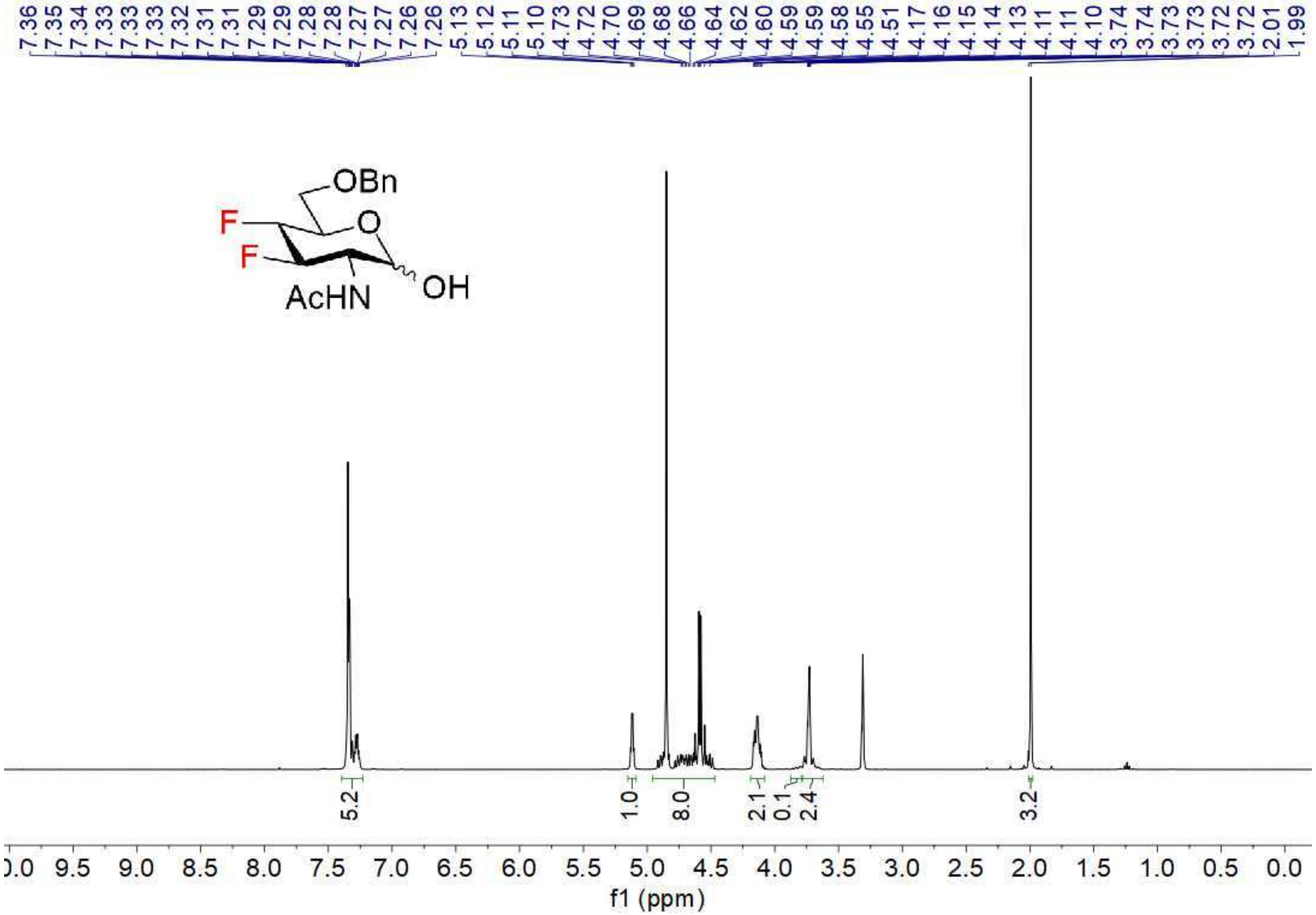

^{13}C NMR (100 MHz, CD$_3$OD) 53

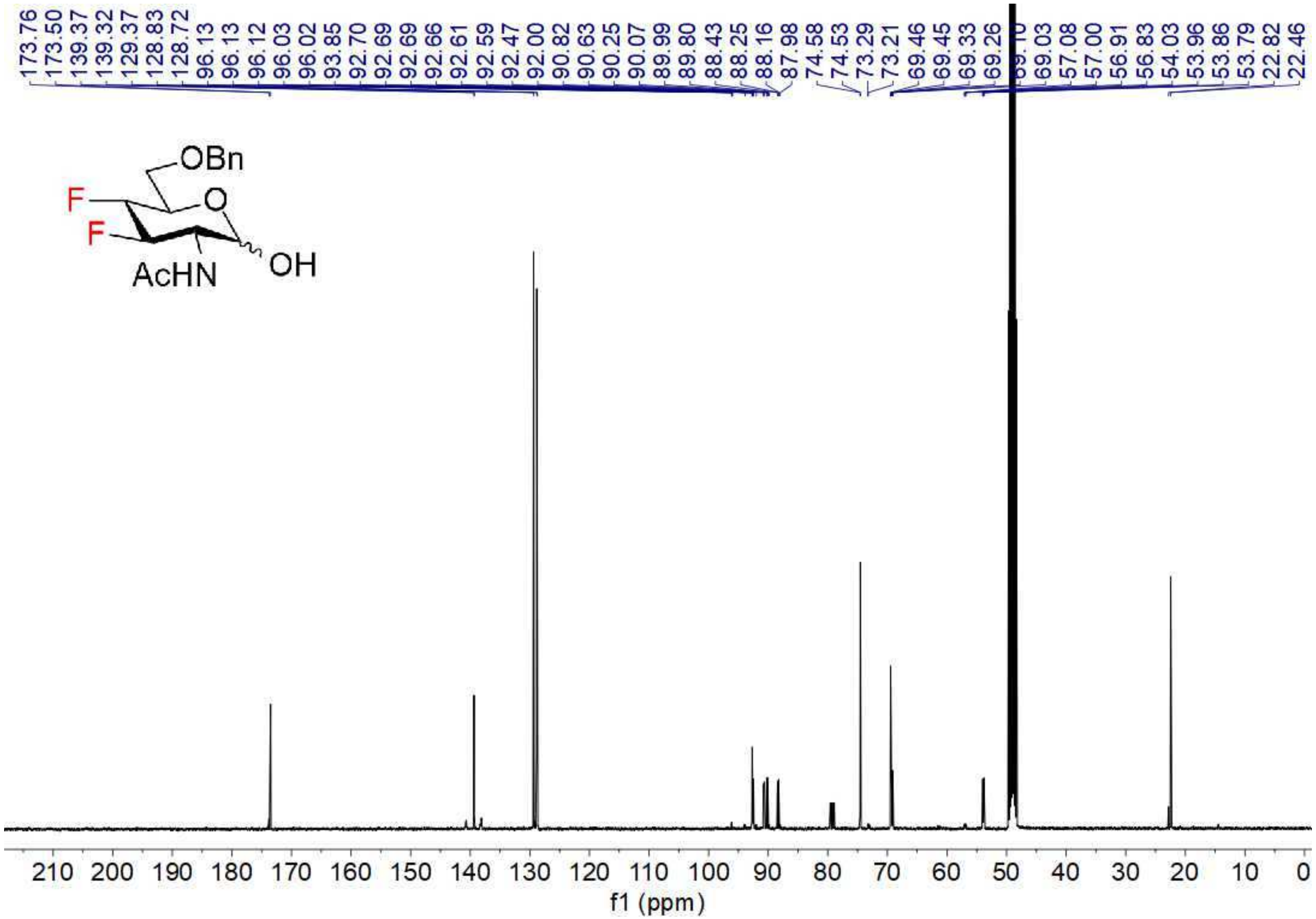

^{19}F NMR (376 MHz, CD_3OD) 53 (α/β ca. 10/1)

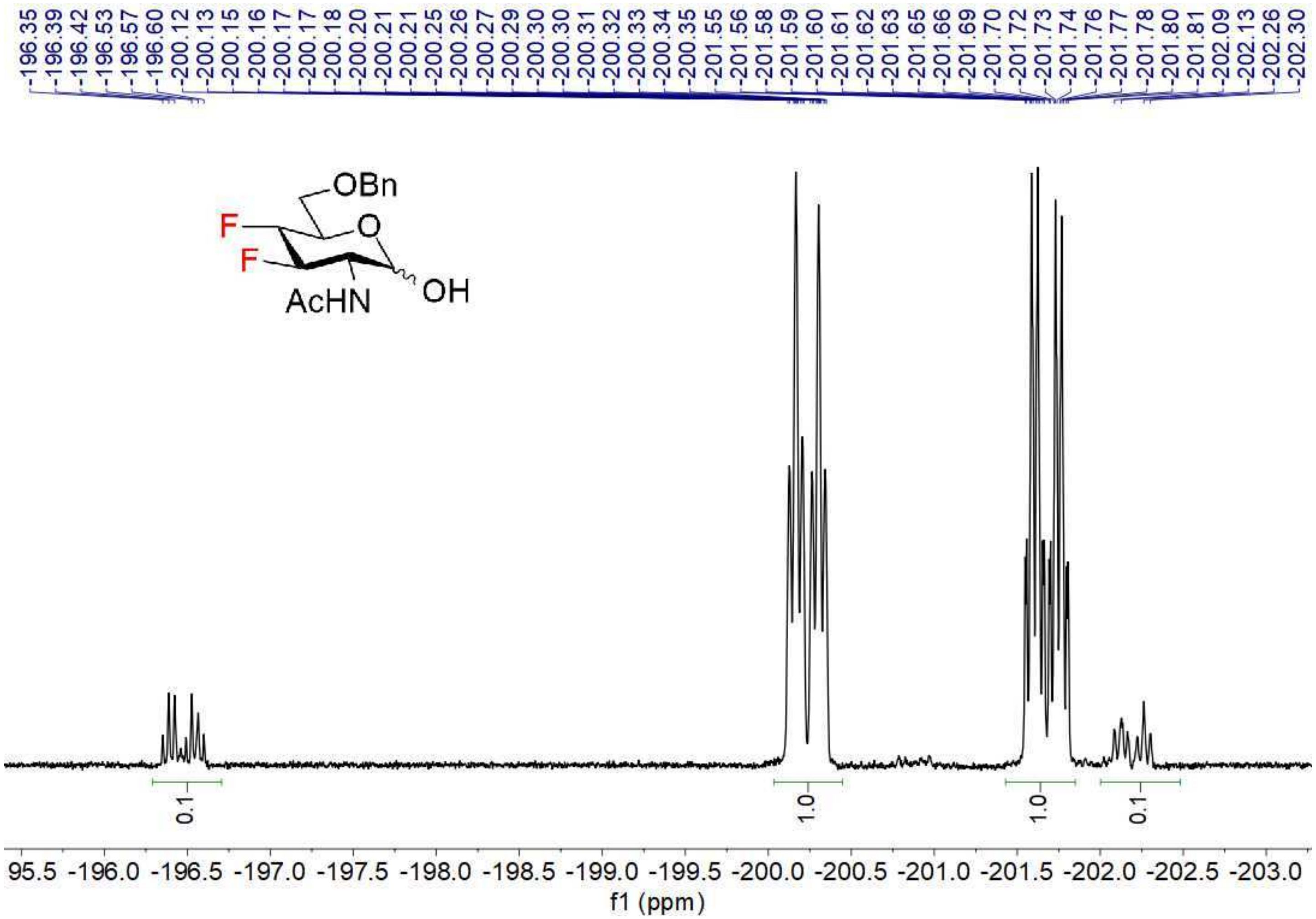

^{1}H-^{1}H COSY 53

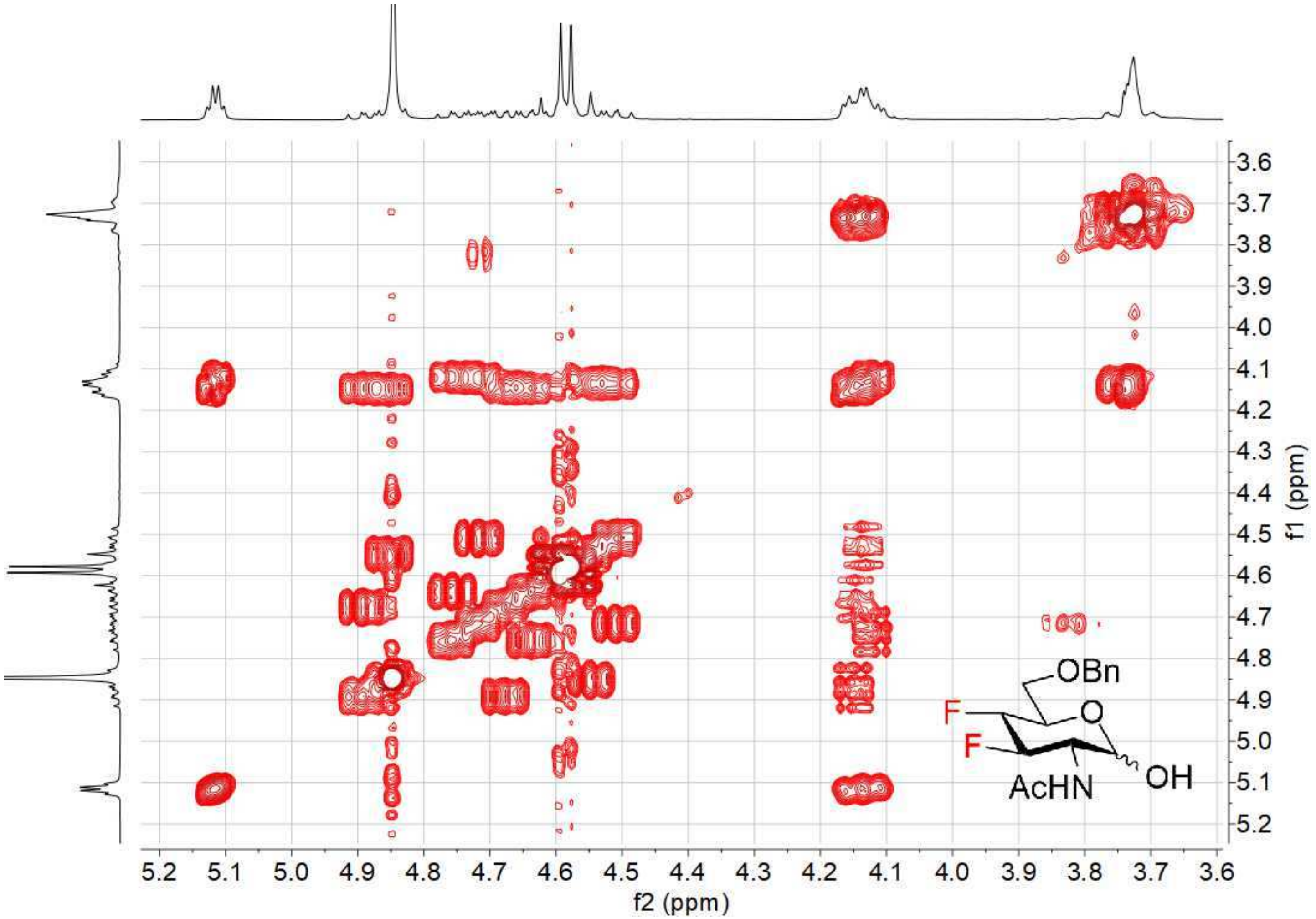

5.2 5.1 5.0 4.9 4.8 4.7 4.6 4.5 4.4 4.3 4.2 4.1 4.0 3.9 3.8 3.7 3.6
f2 (ppm)
3.6 3.7 3.8 3.9 4.0 4.1 4.2 4.3 4.4 4.5 4.6 4.7 4.8 4.9 5.0 5.1 5.2
f1 (ppm)
OBn
F
F
O
OH
AcHN

$^{1}H-^{13}C$ HMBC 53

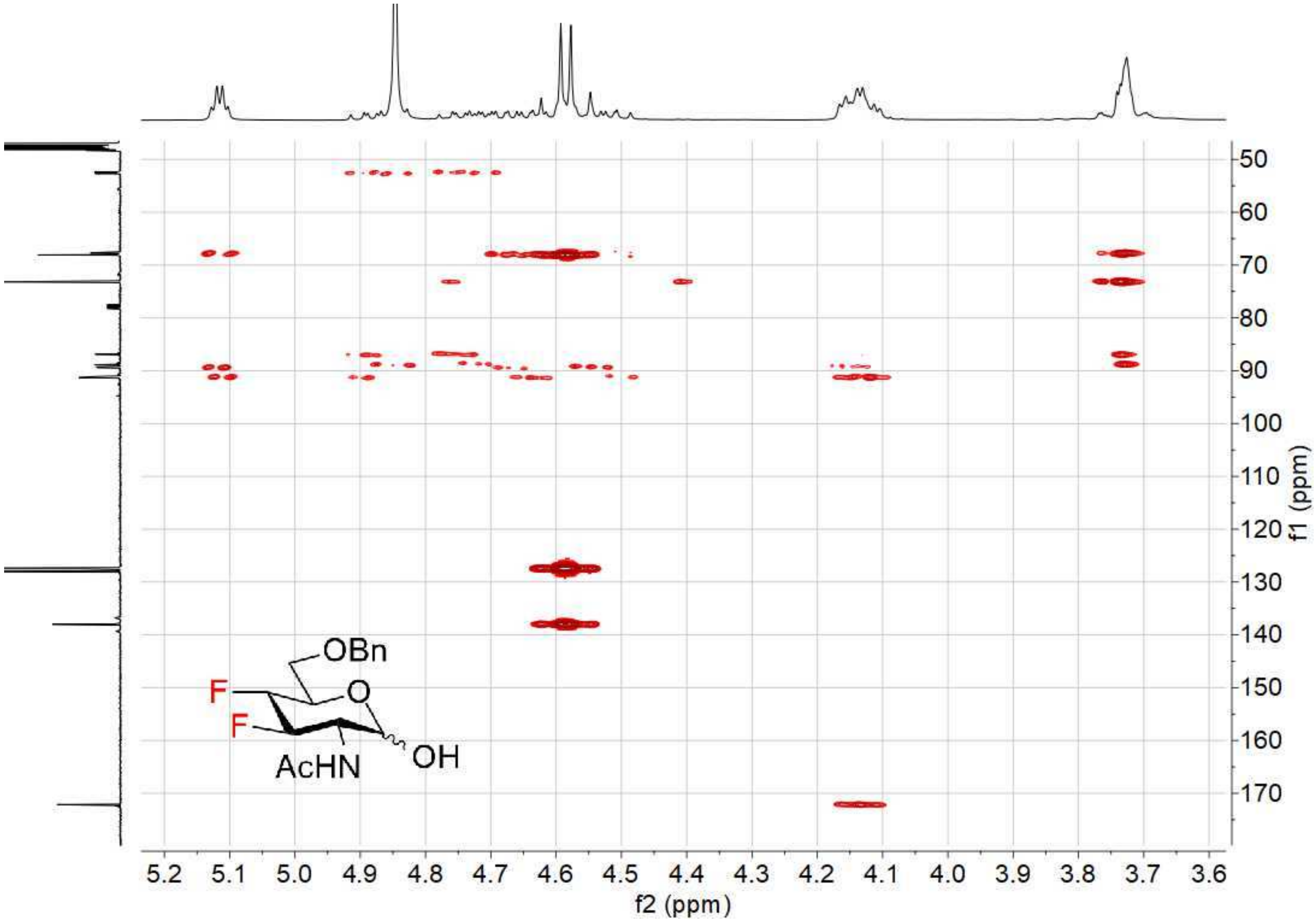

^{1}H-^{13}C HSQC 53

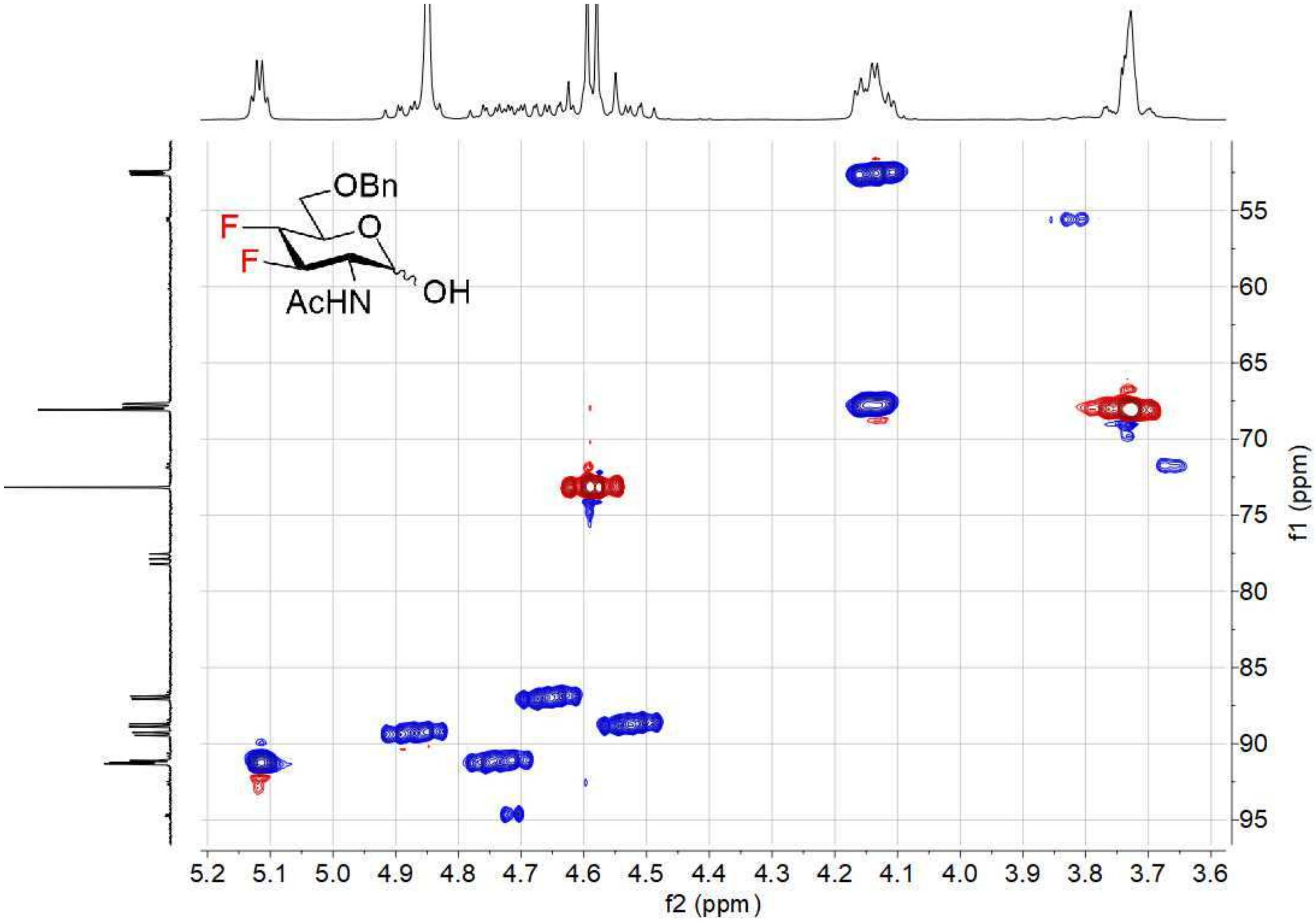

NMR COMPOUND 54

^{1}H NMR (400 MHz, CD_3OD) 54

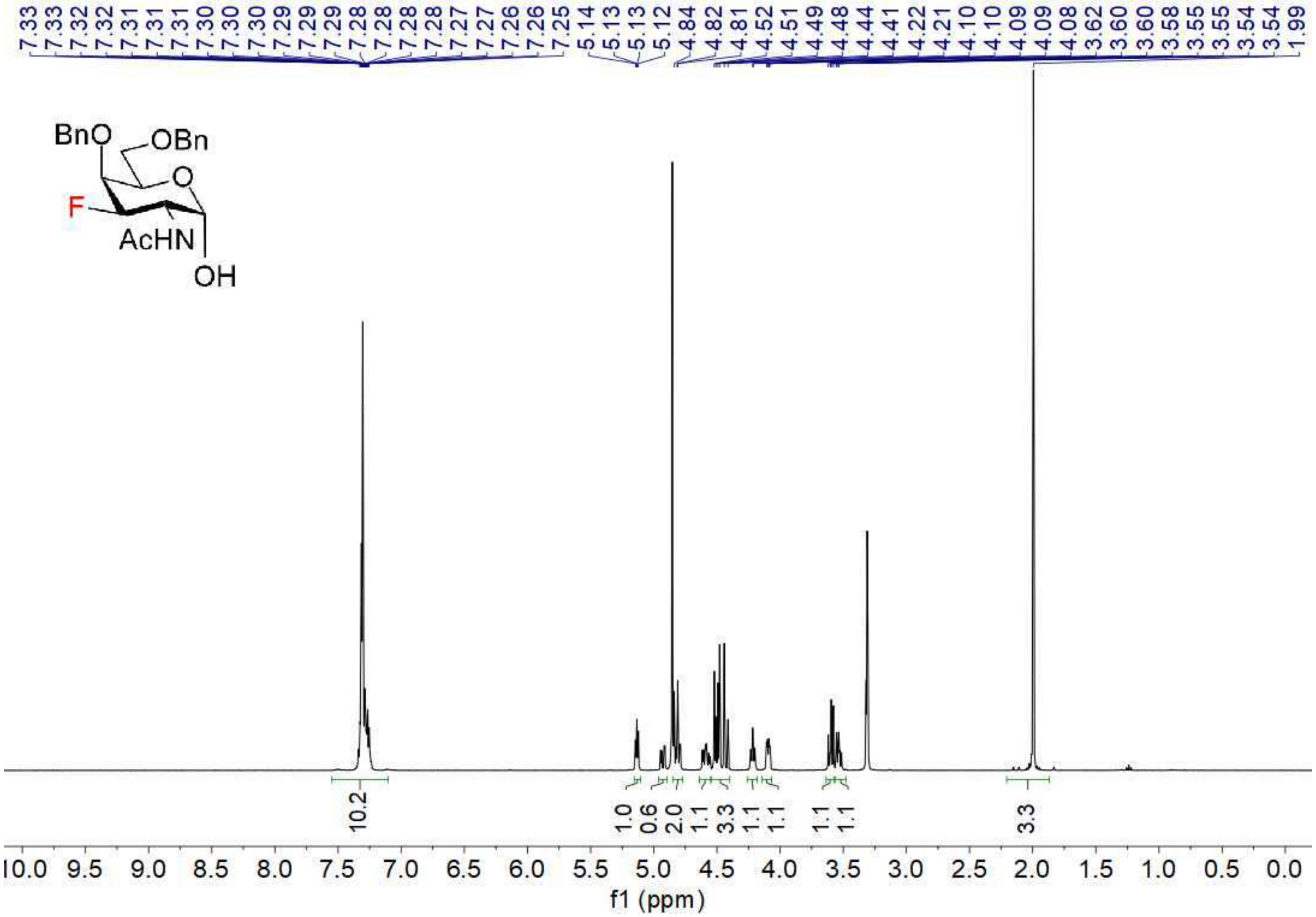

^{13}C NMR (100 MHz, CD_3OD) 54

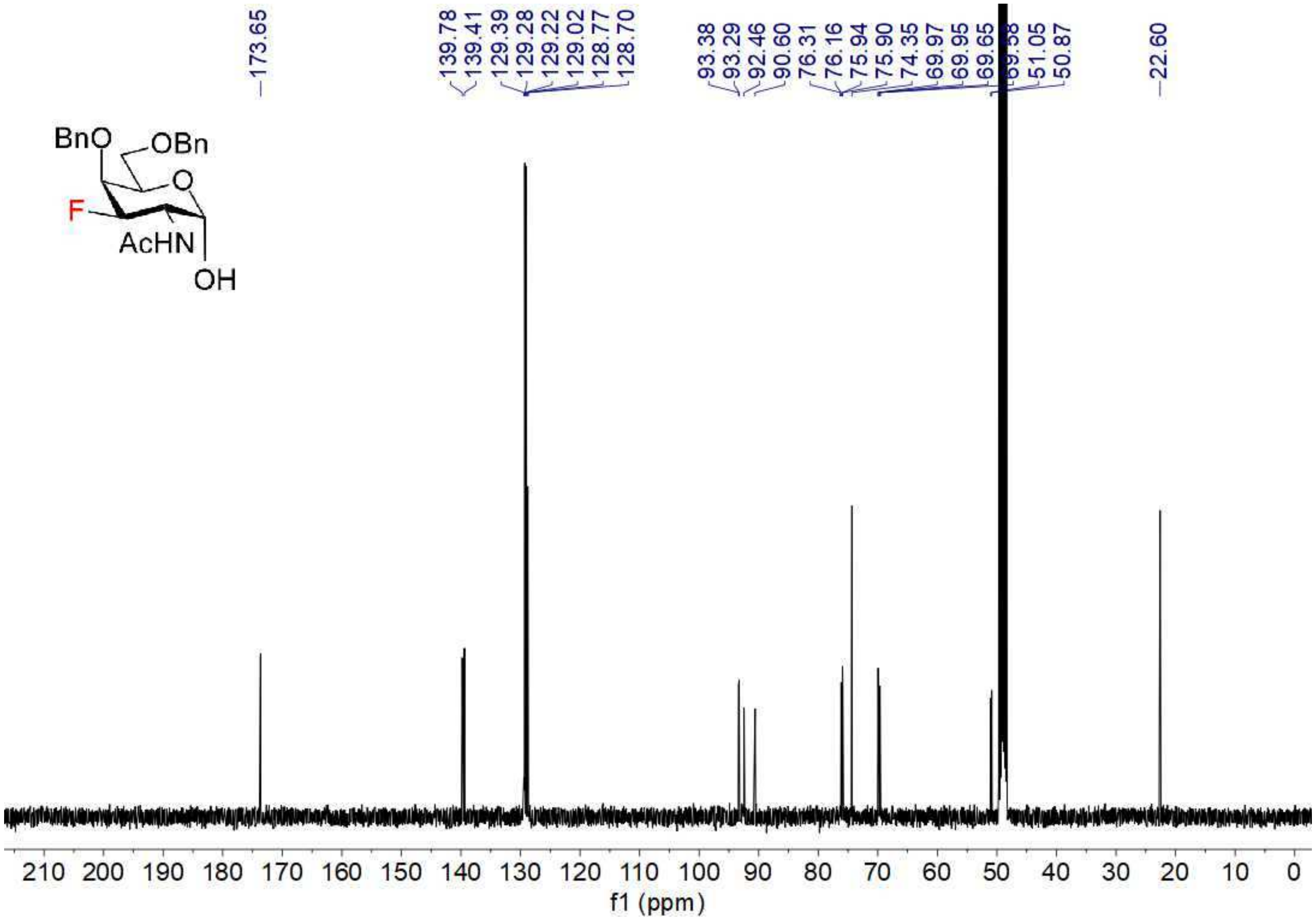

^{19}F NMR (376 MHz, CD_3OD) 54

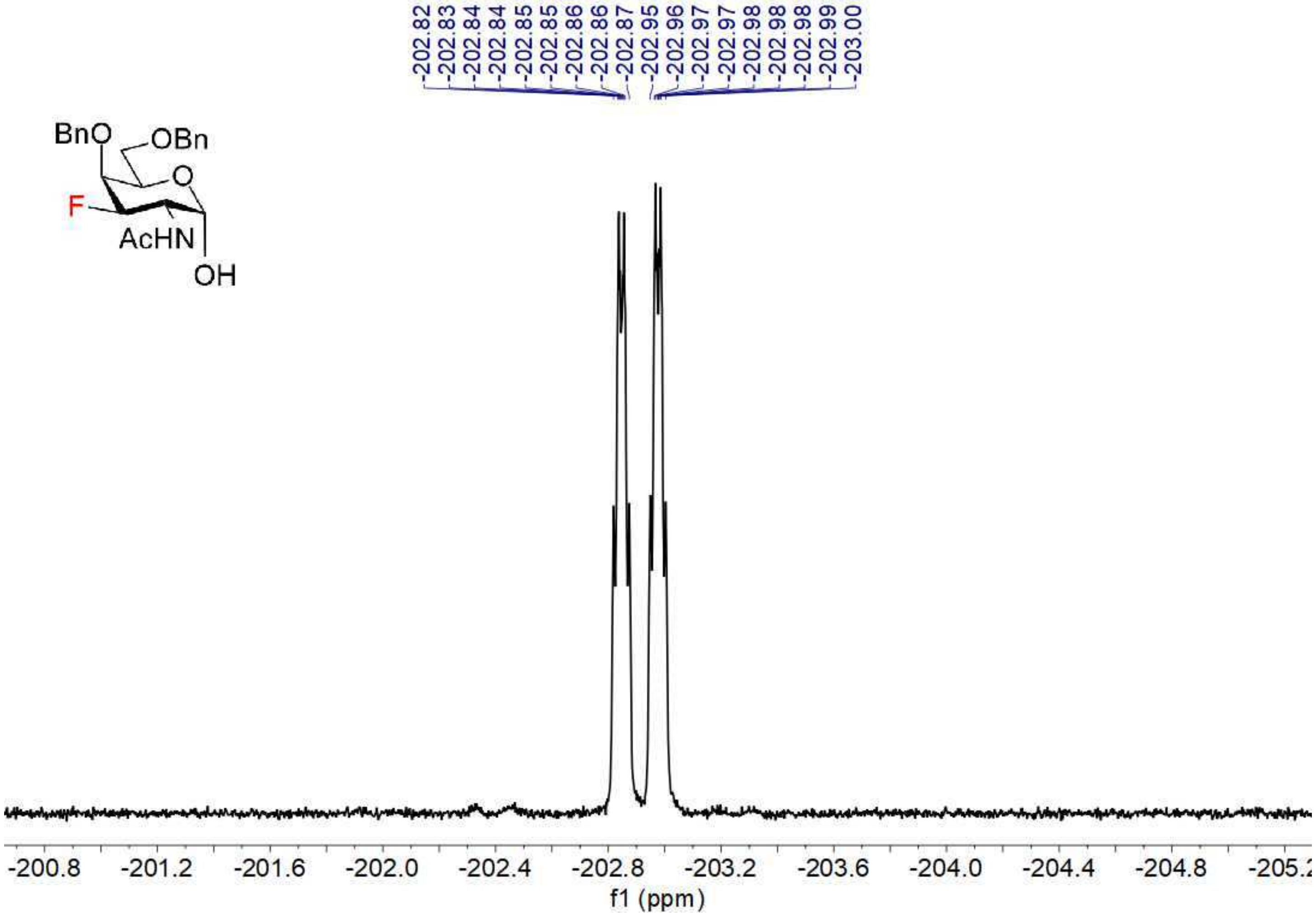

^{1}H-^{1}H COSY 54

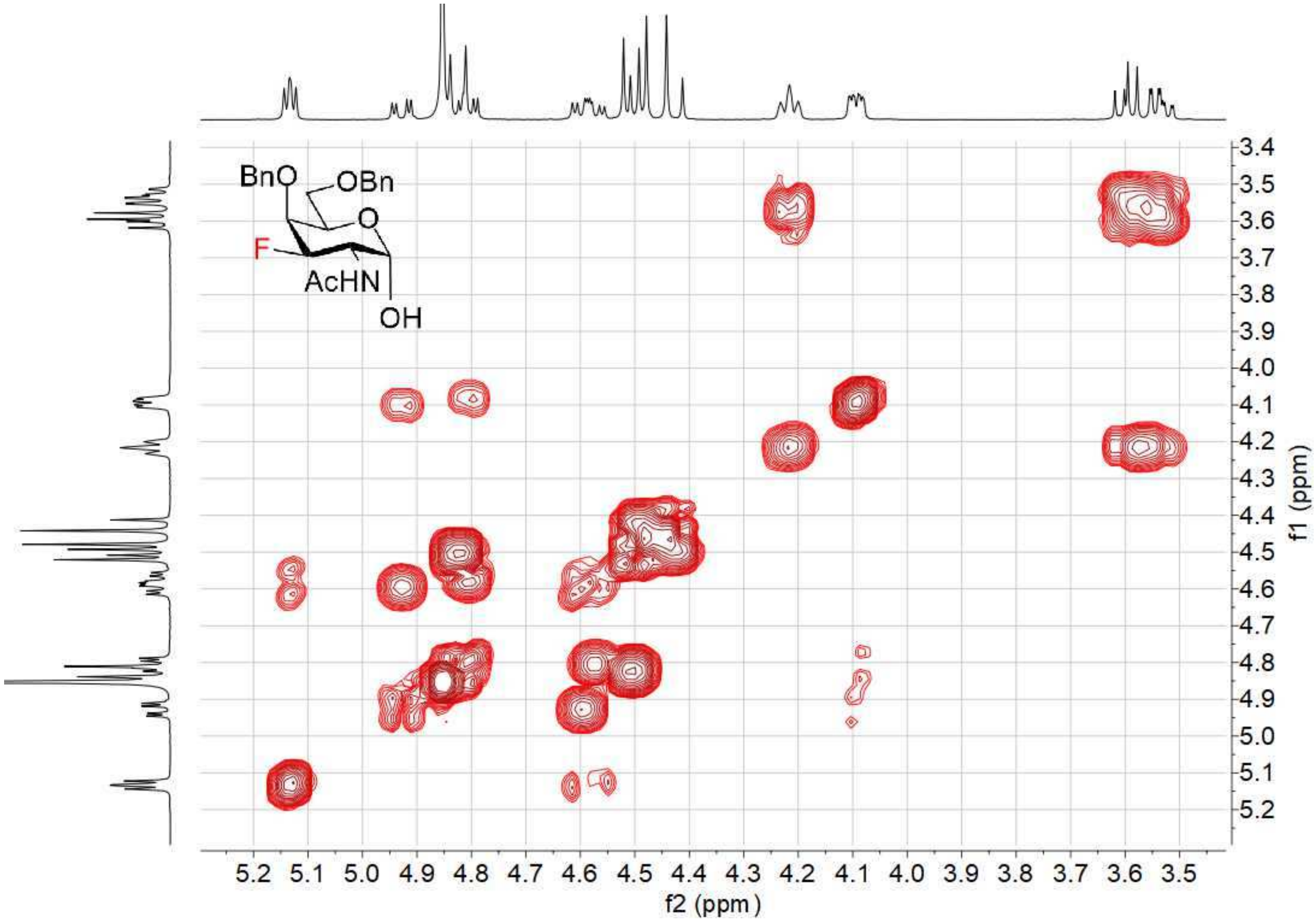
BnO
OBn
O
F
AcHN
OH
5.2 5.1 5.0 4.9 4.8 4.7 4.6 4.5 4.4 4.3 4.2 4.1 4.0 3.9 3.8 3.7 3.6 3.5
f2 (ppm)
3.4 3.5 3.6 3.7 3.8 3.9 4.0 4.1 4.2 4.3 4.4 4.5 4.6 4.7 4.8 4.9 5.0 5.1 5.2
f1 (ppm)

^{1}H-^{13}C HSQC 54

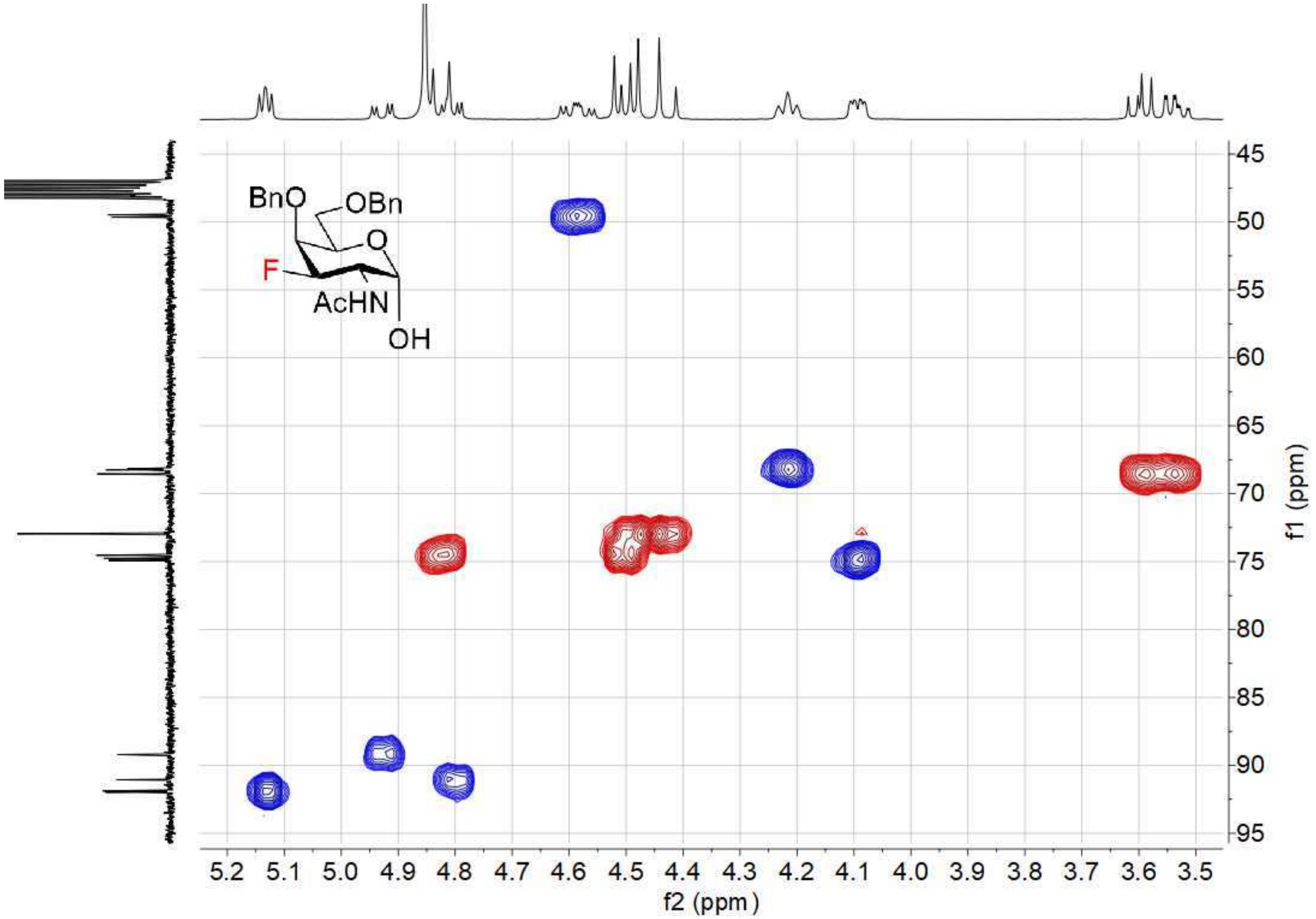

NMR COMPOUND 55

^{1}H NMR (400 MHz, CD_3OD) 55 (α/β ca. 10/1)

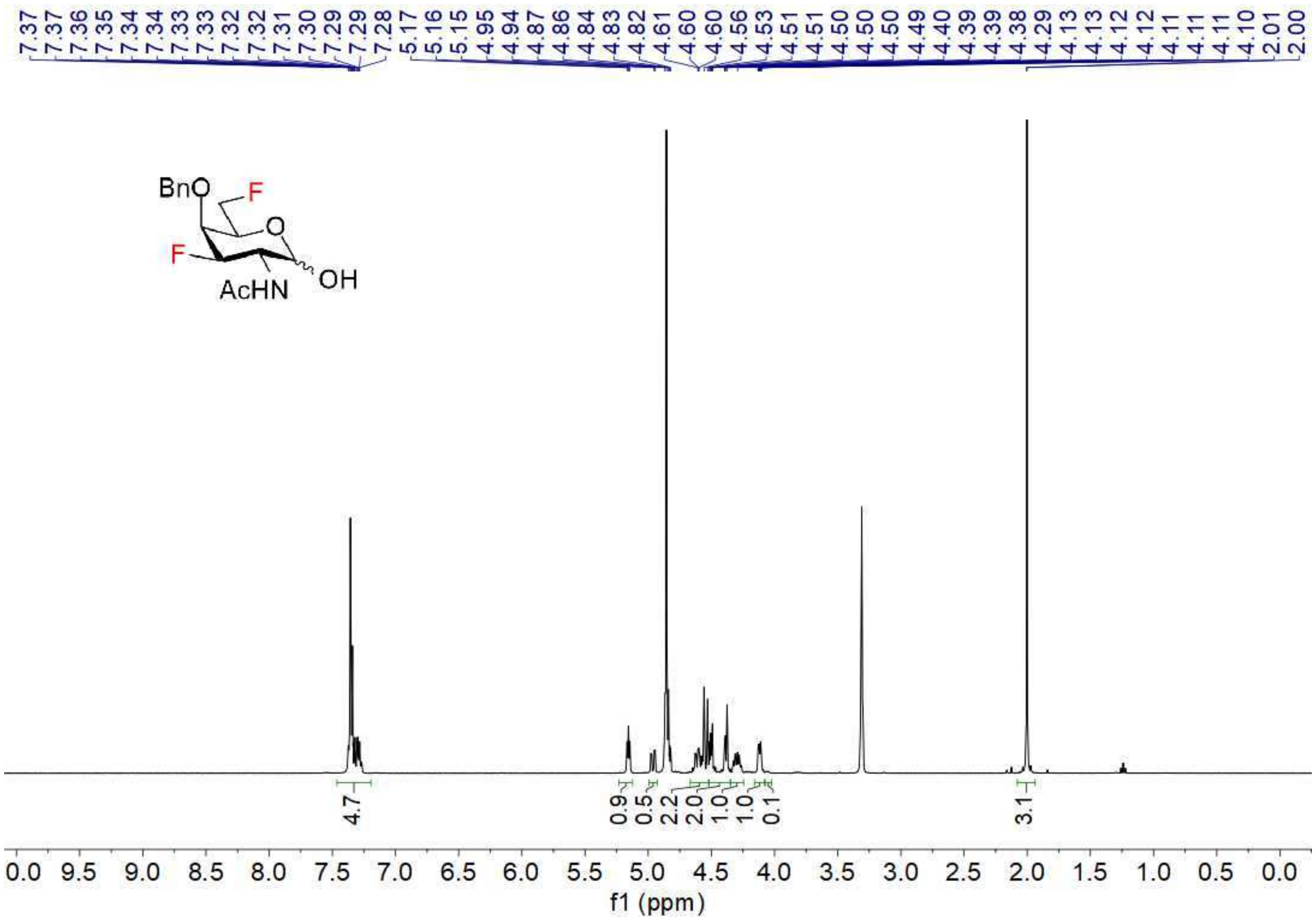

^{13}C NMR (100 MHz, CD_3OD) 55

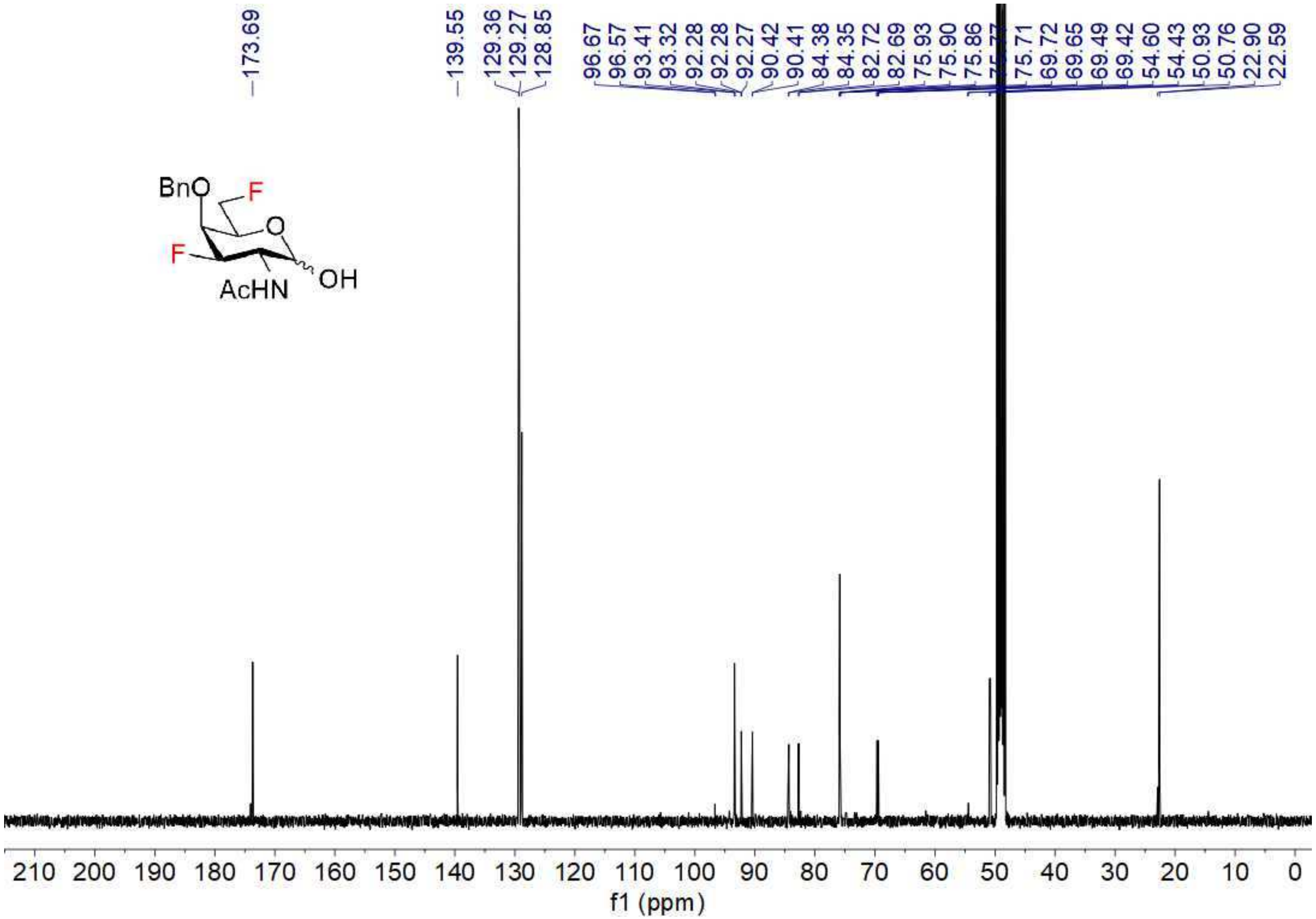

^{19}F NMR (376 MHz, CD_3OD) 55 (α/β ca. 10/1)

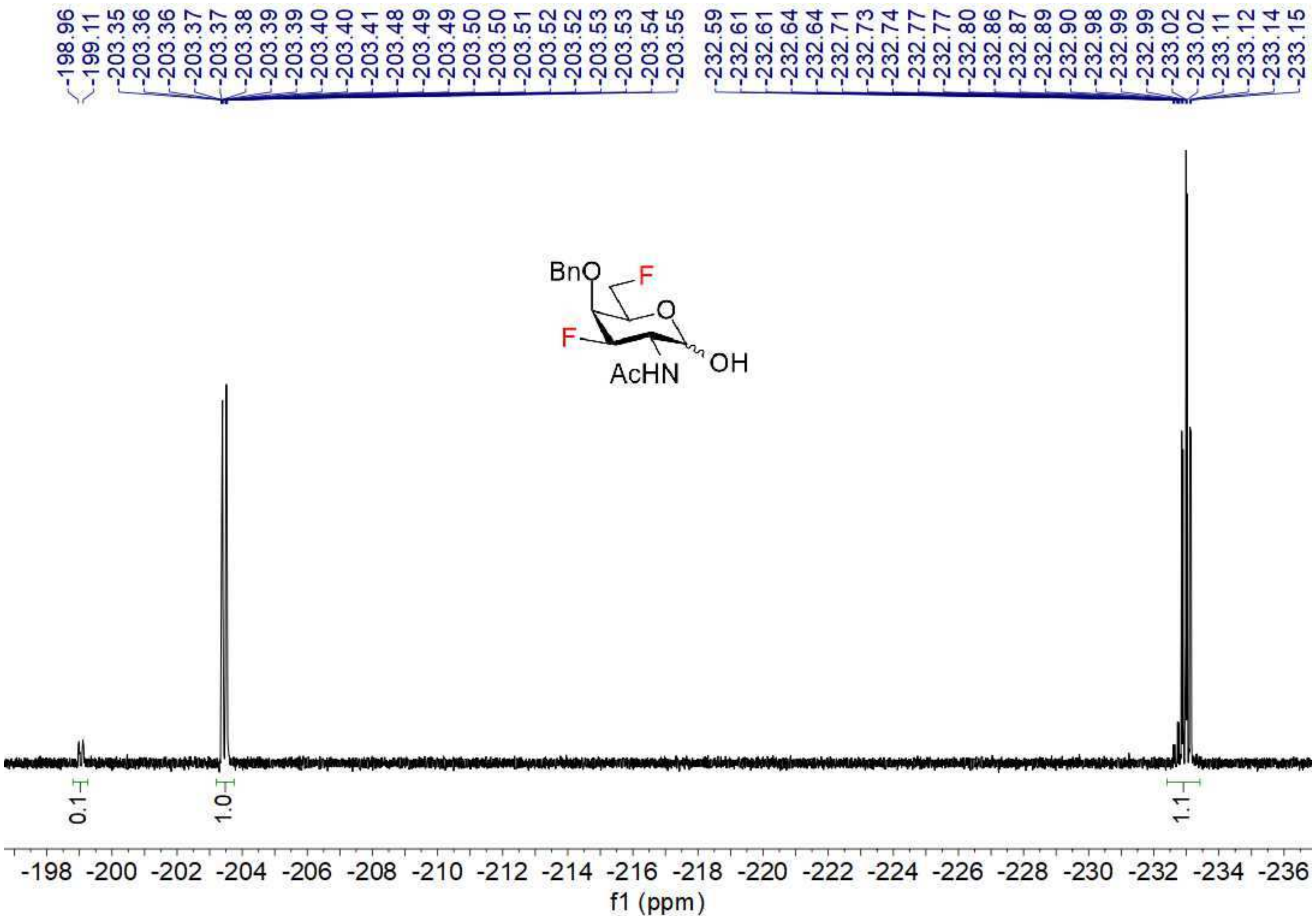

^{1}H-^{1}H COSY 55

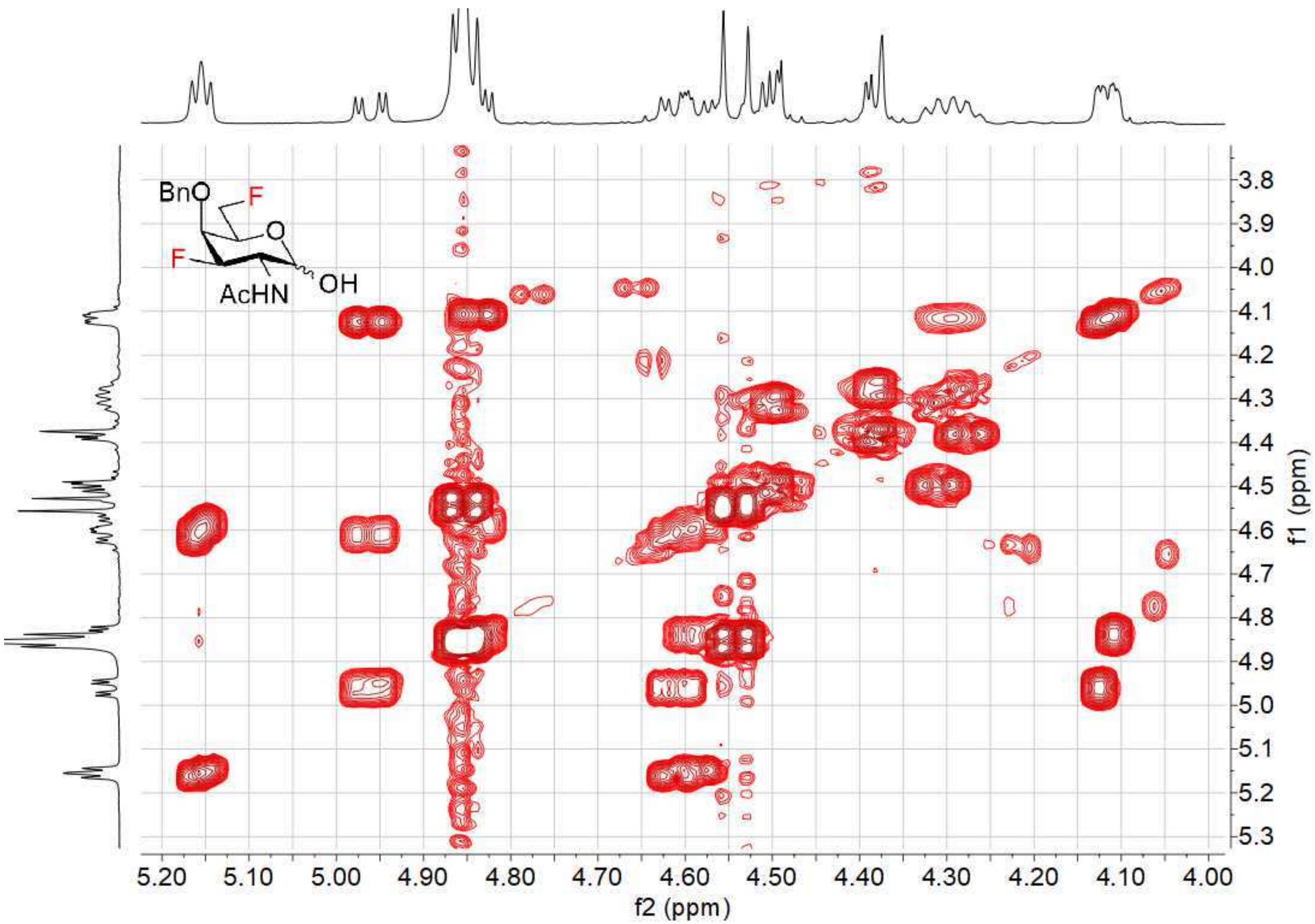

BnO
F
O
F
AcHN
OH
5.20
5.10
5.00
4.90
4.80
4.70
4.60
4.50
4.40
4.30
4.20
4.10
4.00
f2 (ppm)
3.8
3.9
4.0
4.1
4.2
4.3
4.4
4.5
4.6
4.7
4.8
4.9
5.0
5.1
5.2
5.3
f1 (ppm)

^{1}H-^{13}C HSQC 55

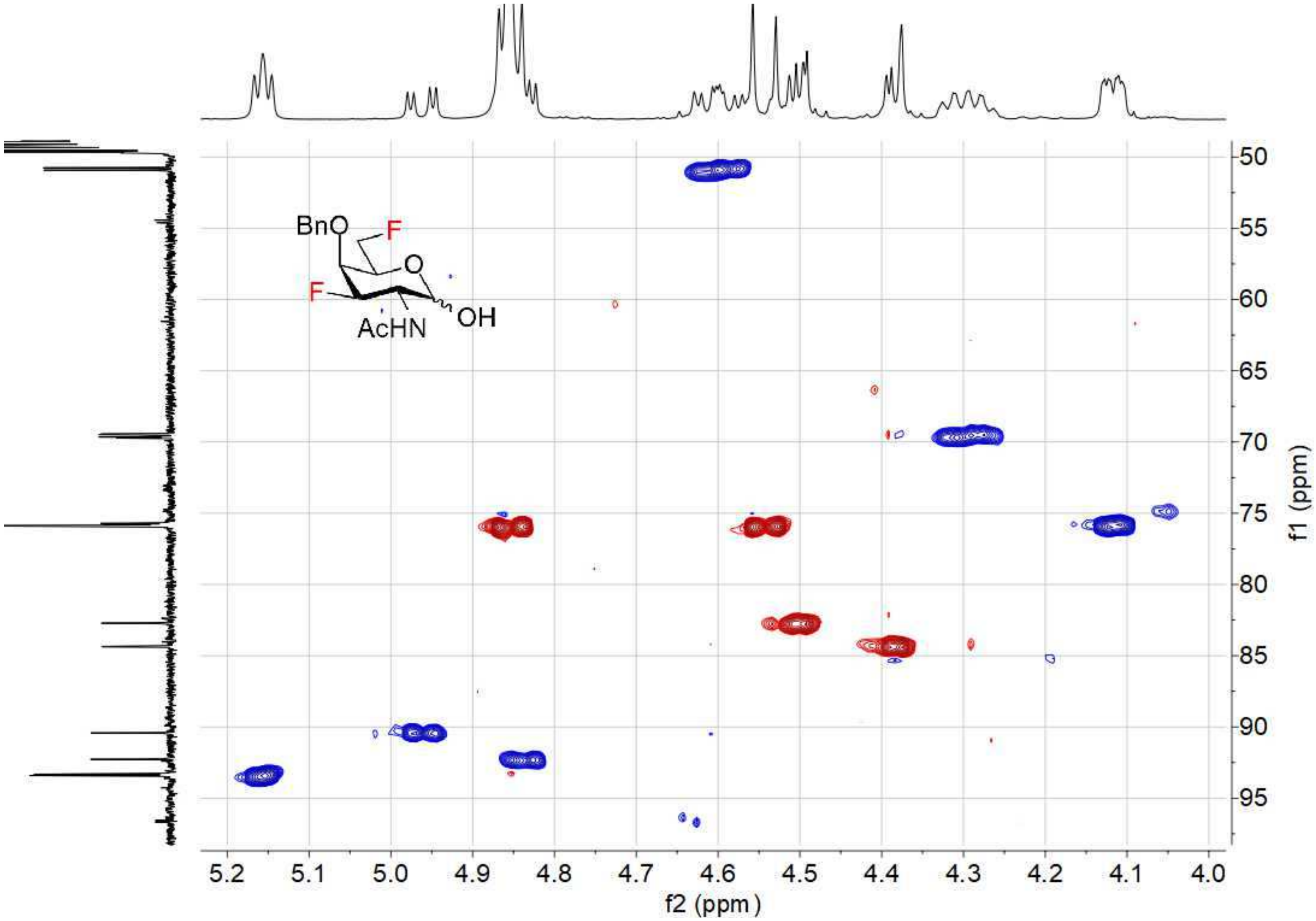

NMR COMPOUND 56

^{1}H NMR (400 MHz, CD_3OD) 56 (α/β ca. 5/1)

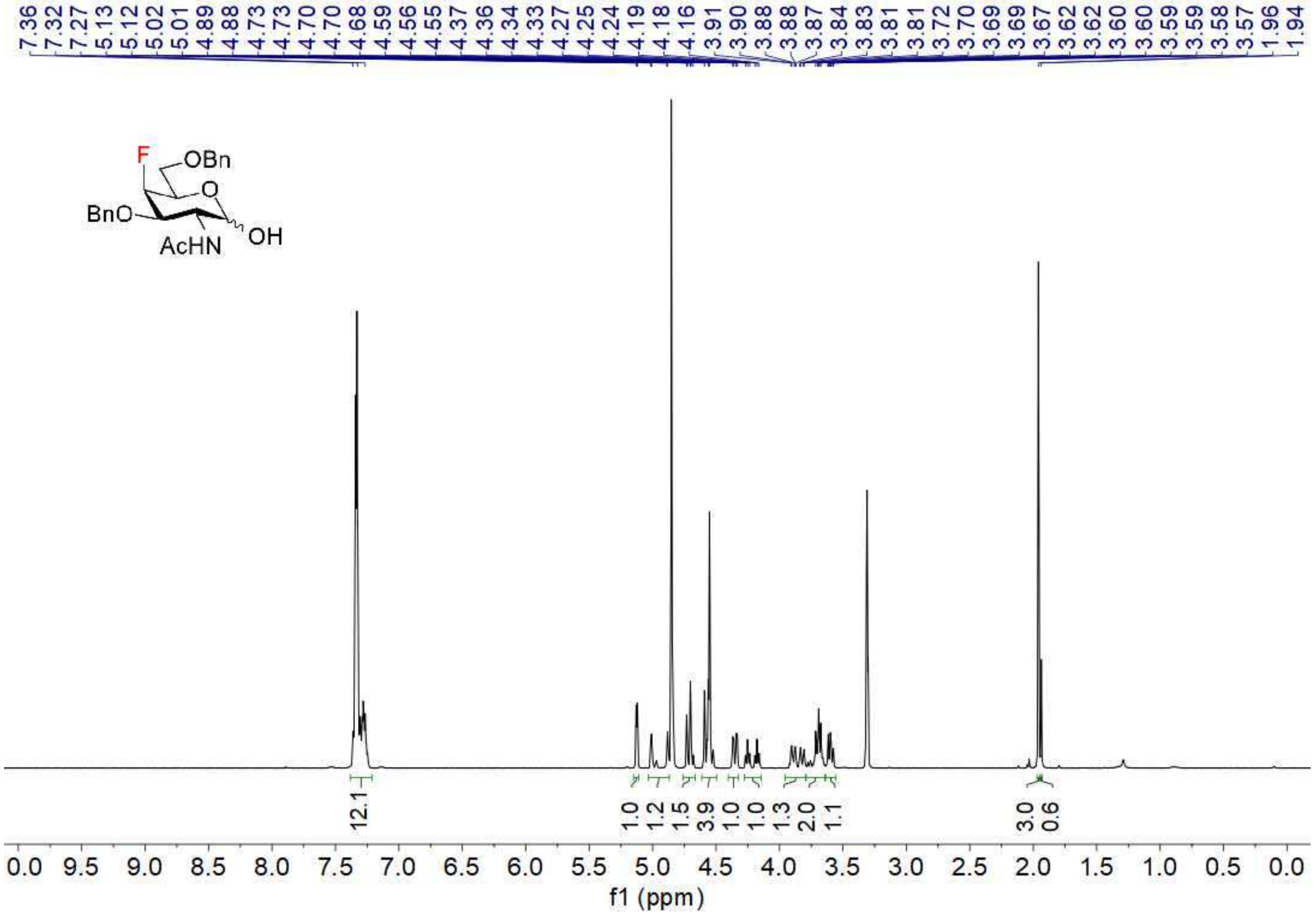

^{13}C NMR (100 MHz, CD_3OD) 56

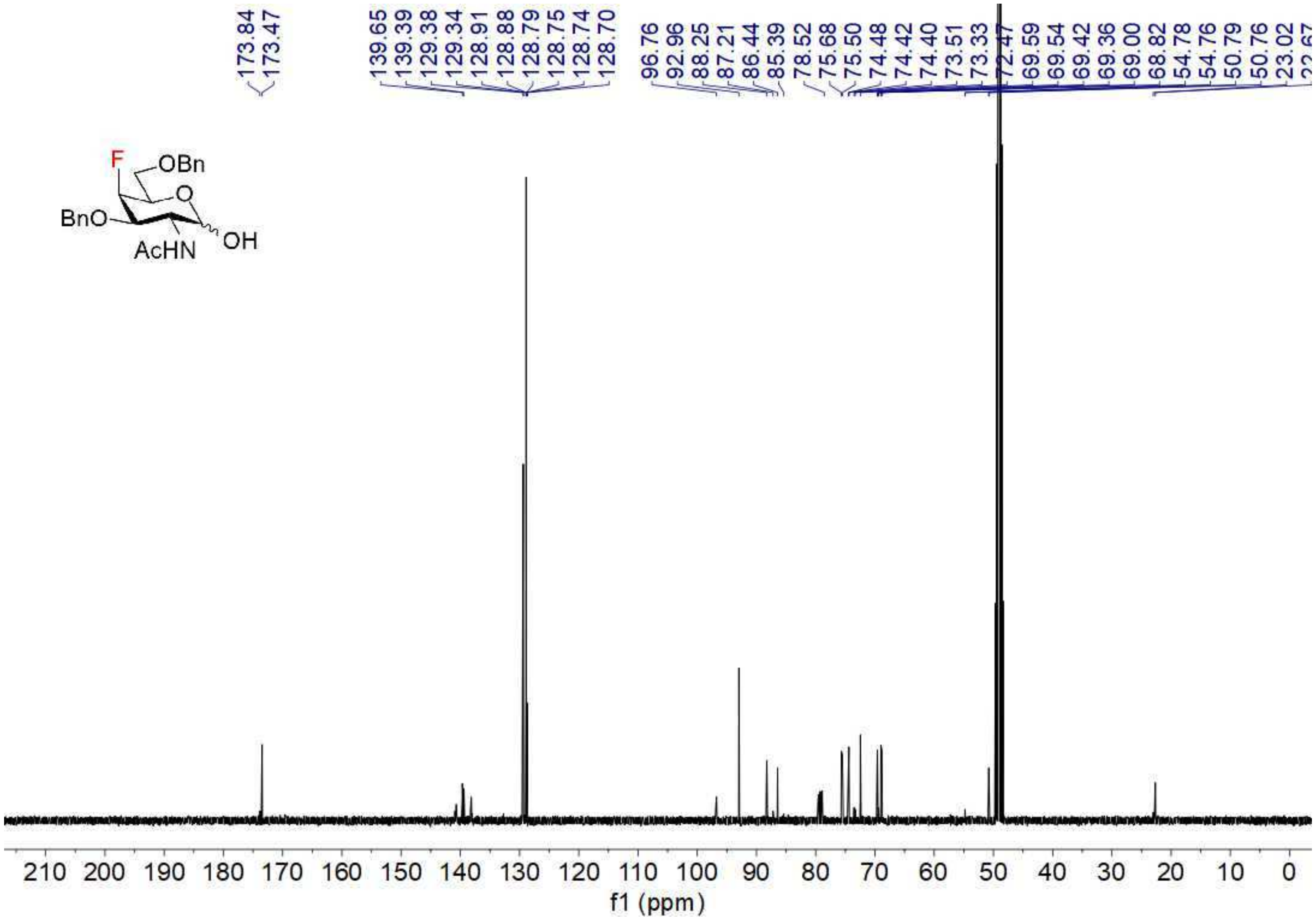

^{19}F NMR (376 MHz, CD_3OD) 56 (α/β ca. 5/1)

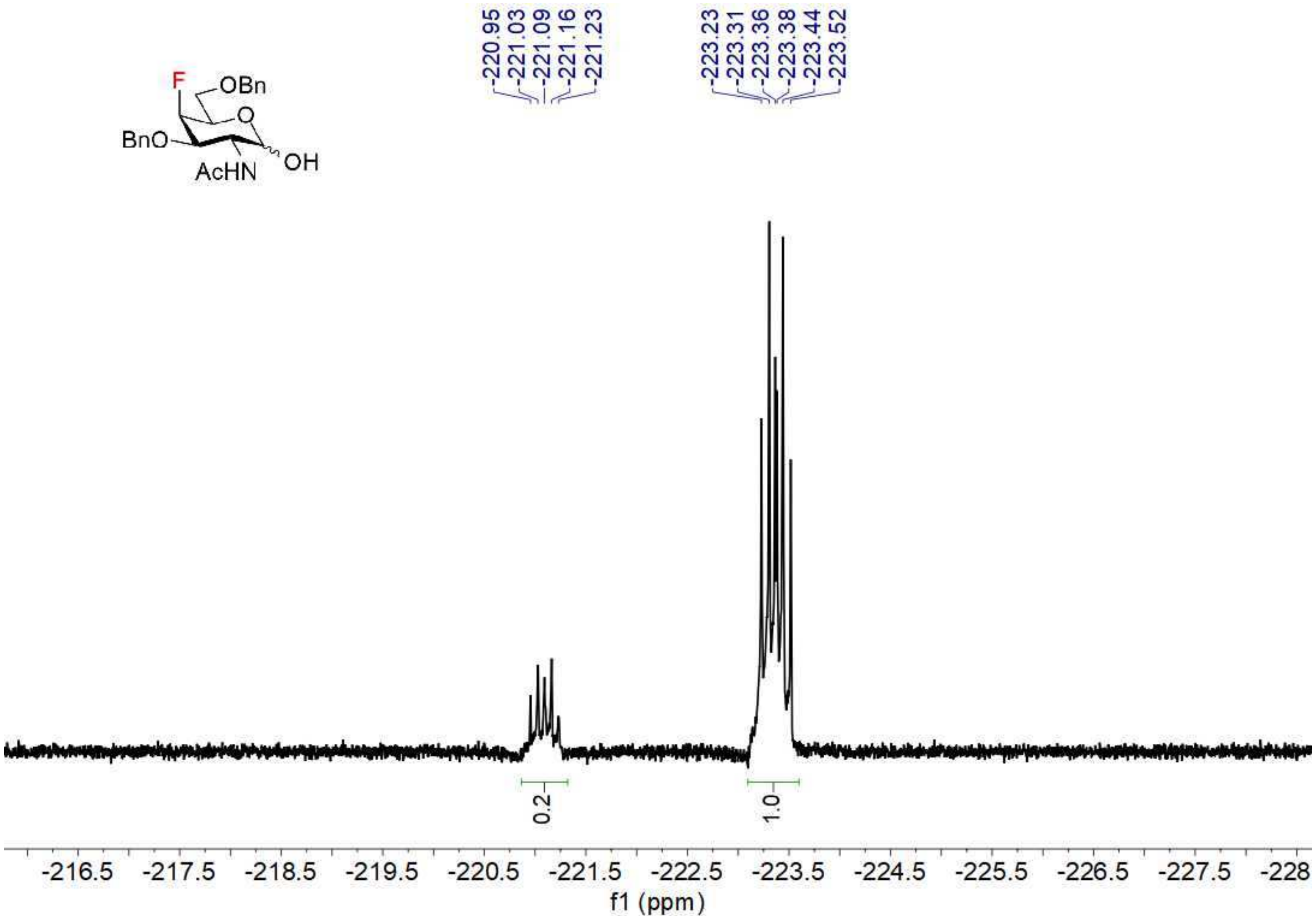

^{1}H-^{1}H COSY 56

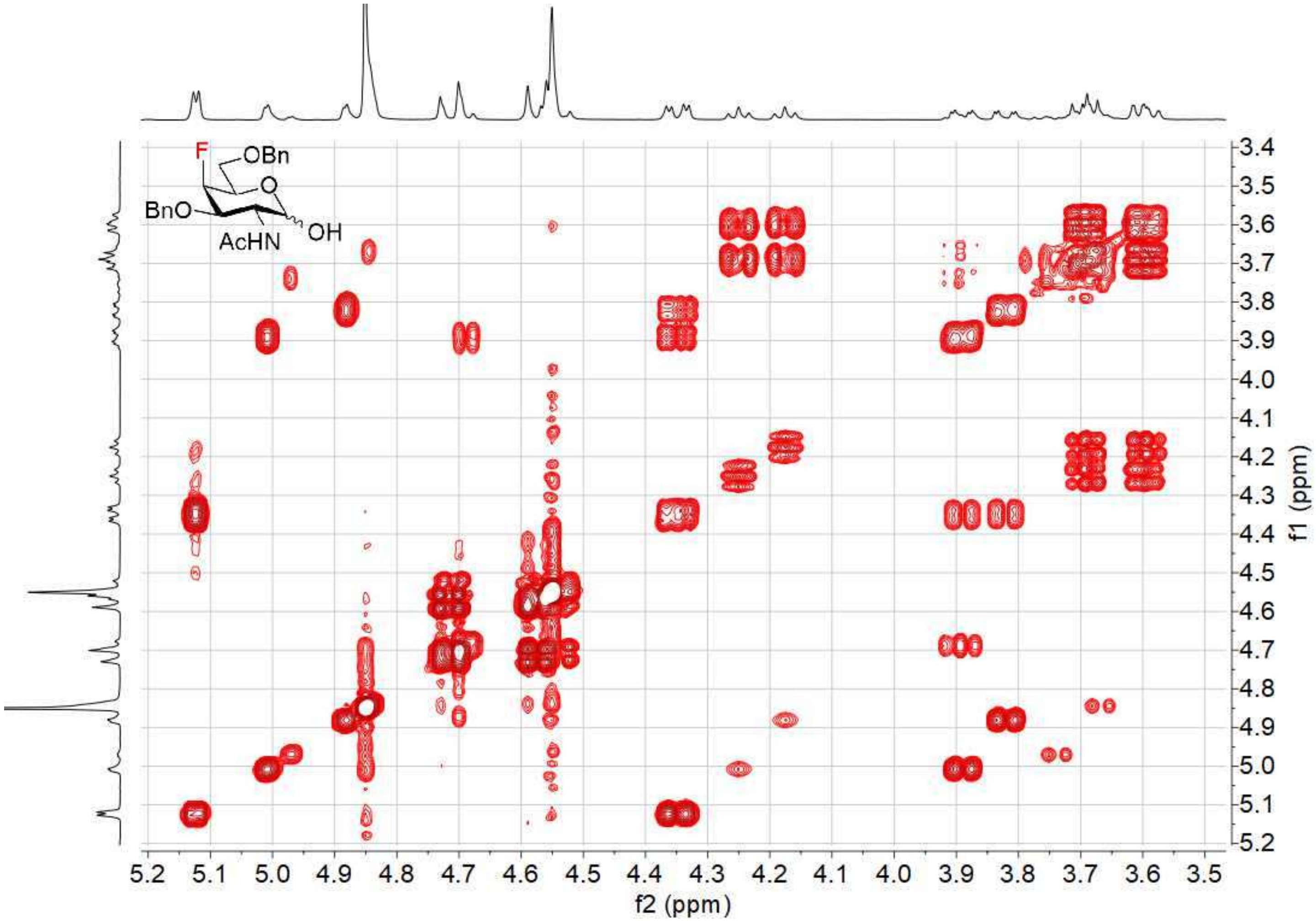

^{1}H-^{13}C HMBC 56

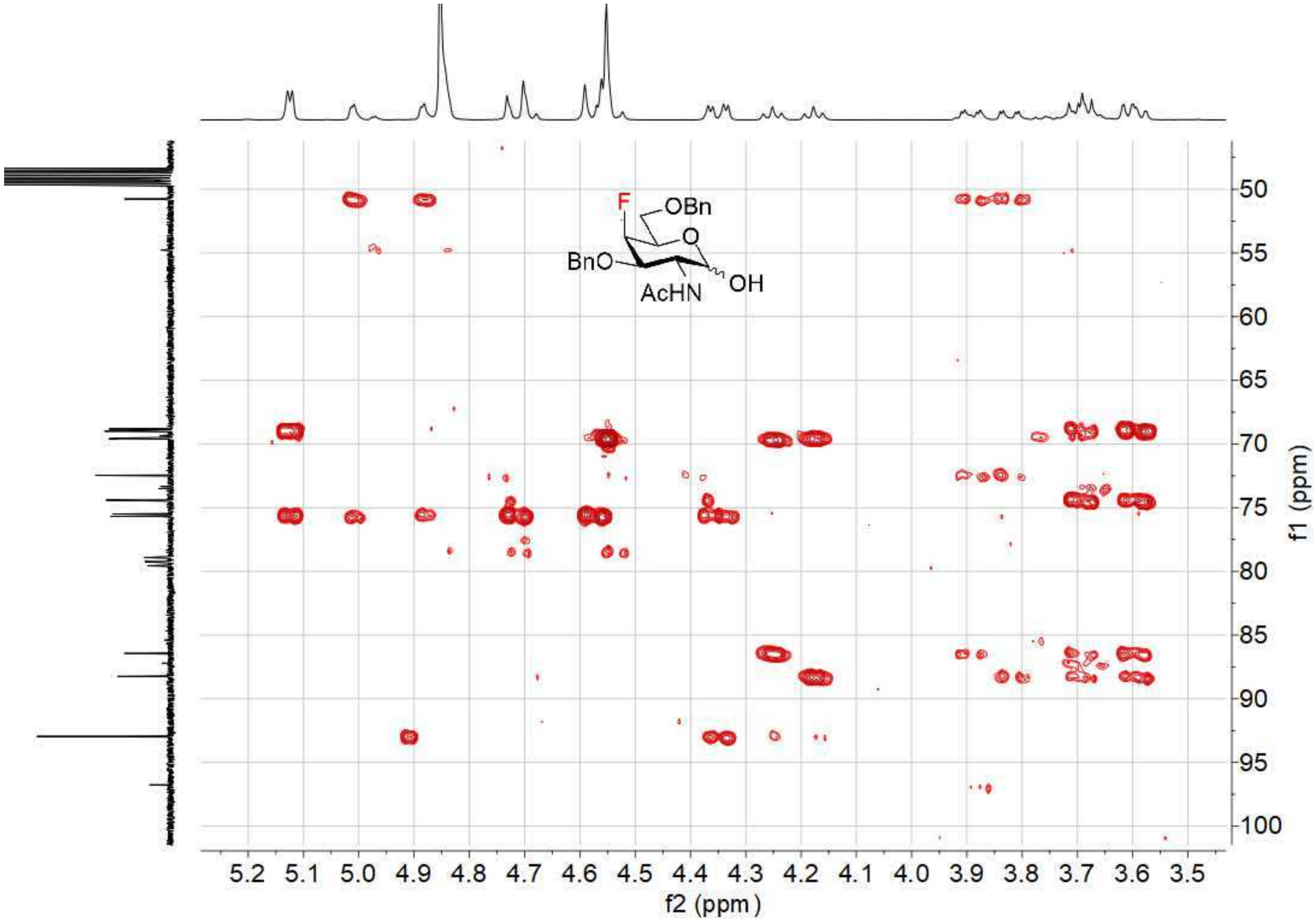

^{1}H-^{13}C HSQC 56

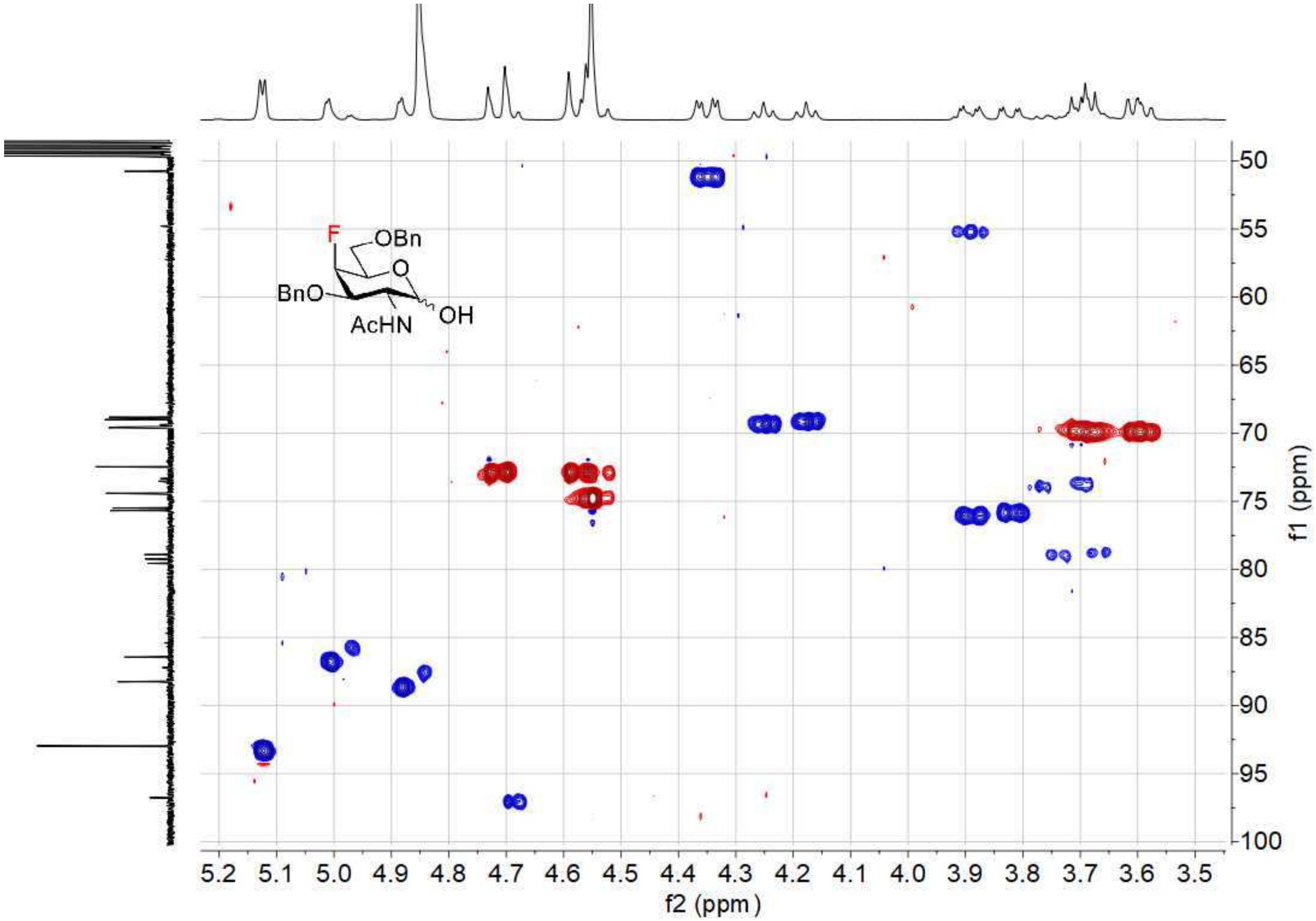

NMR COMPOUND 57

^{1}H NMR (400 MHz, CD_3OD) 57 (α/β ca. 10/1)

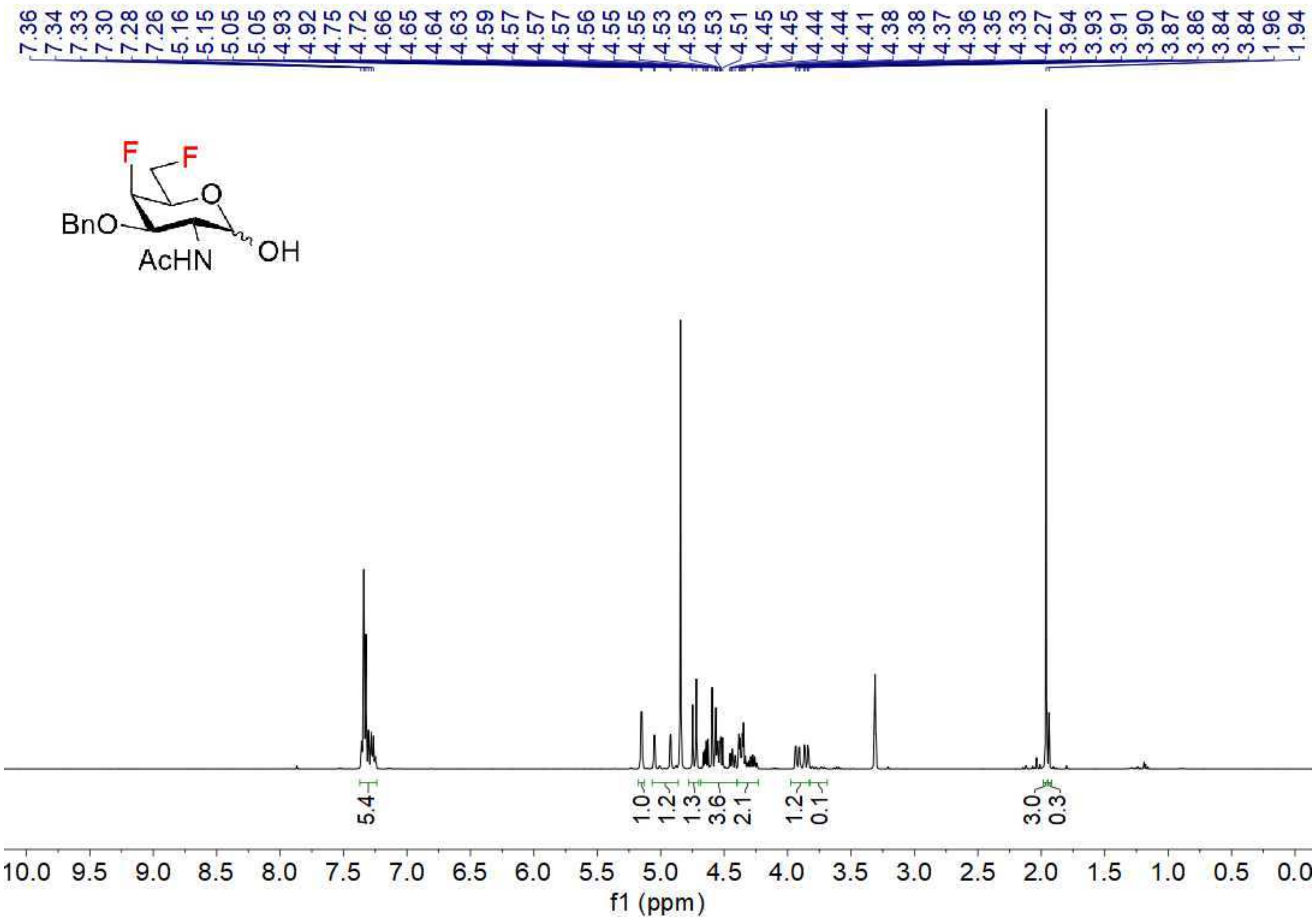

^{13}C NMR (100 MHz, CD_3OD) 57

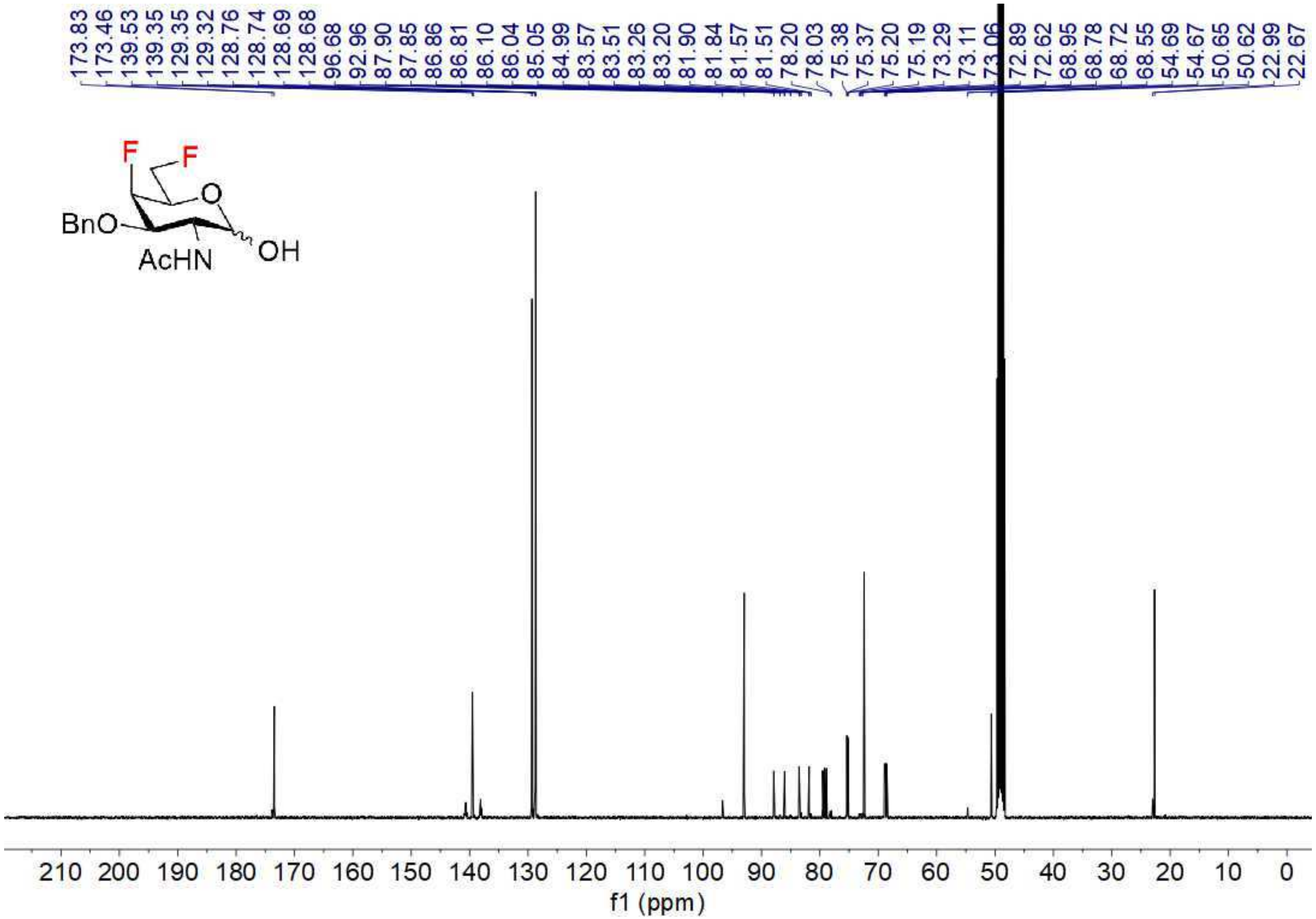

^{19}F NMR (376 MHz, CD_3OD) 57 (α/β ca. 10/1)

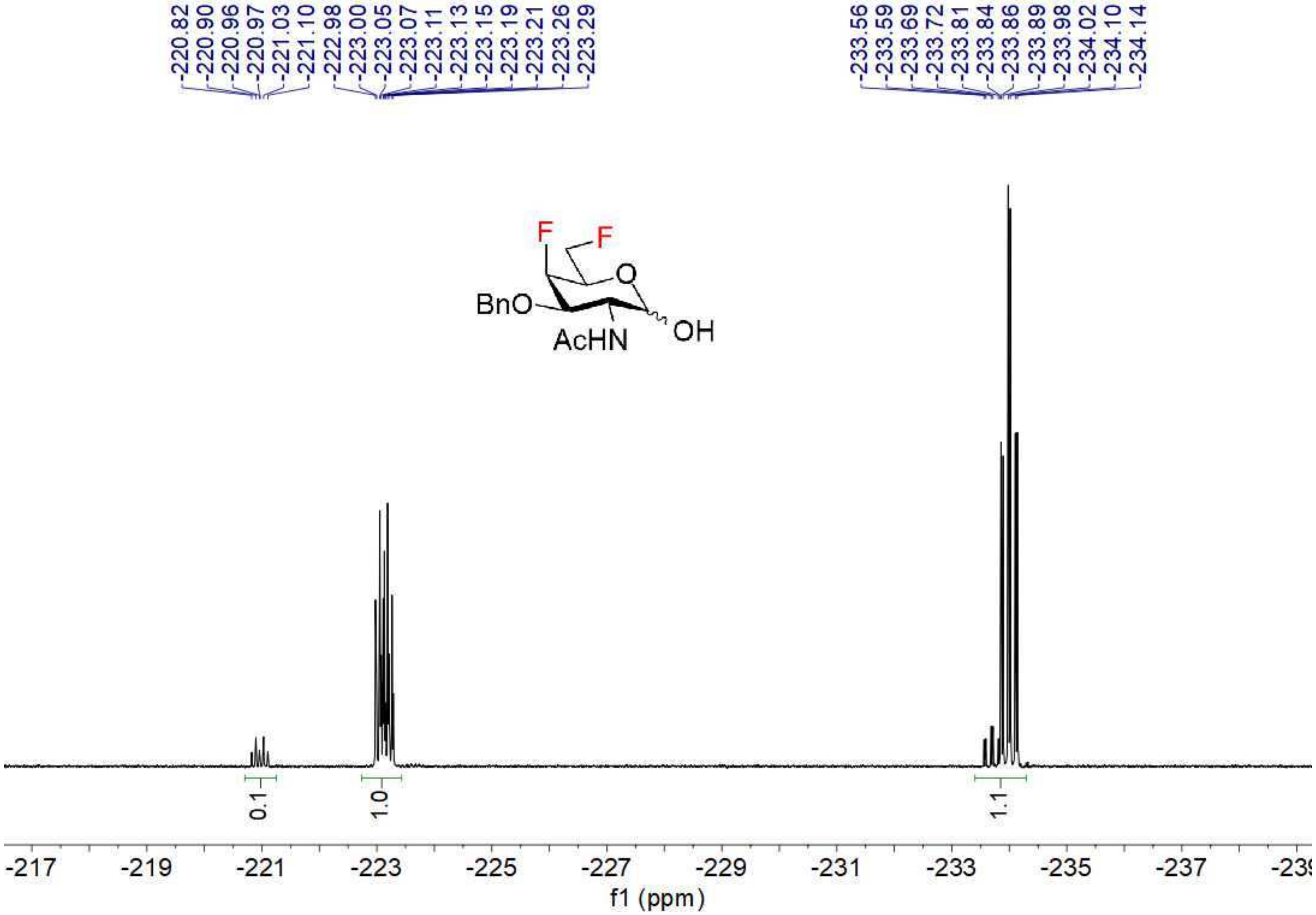

^{1}H-^{1}H COSY NMR 57

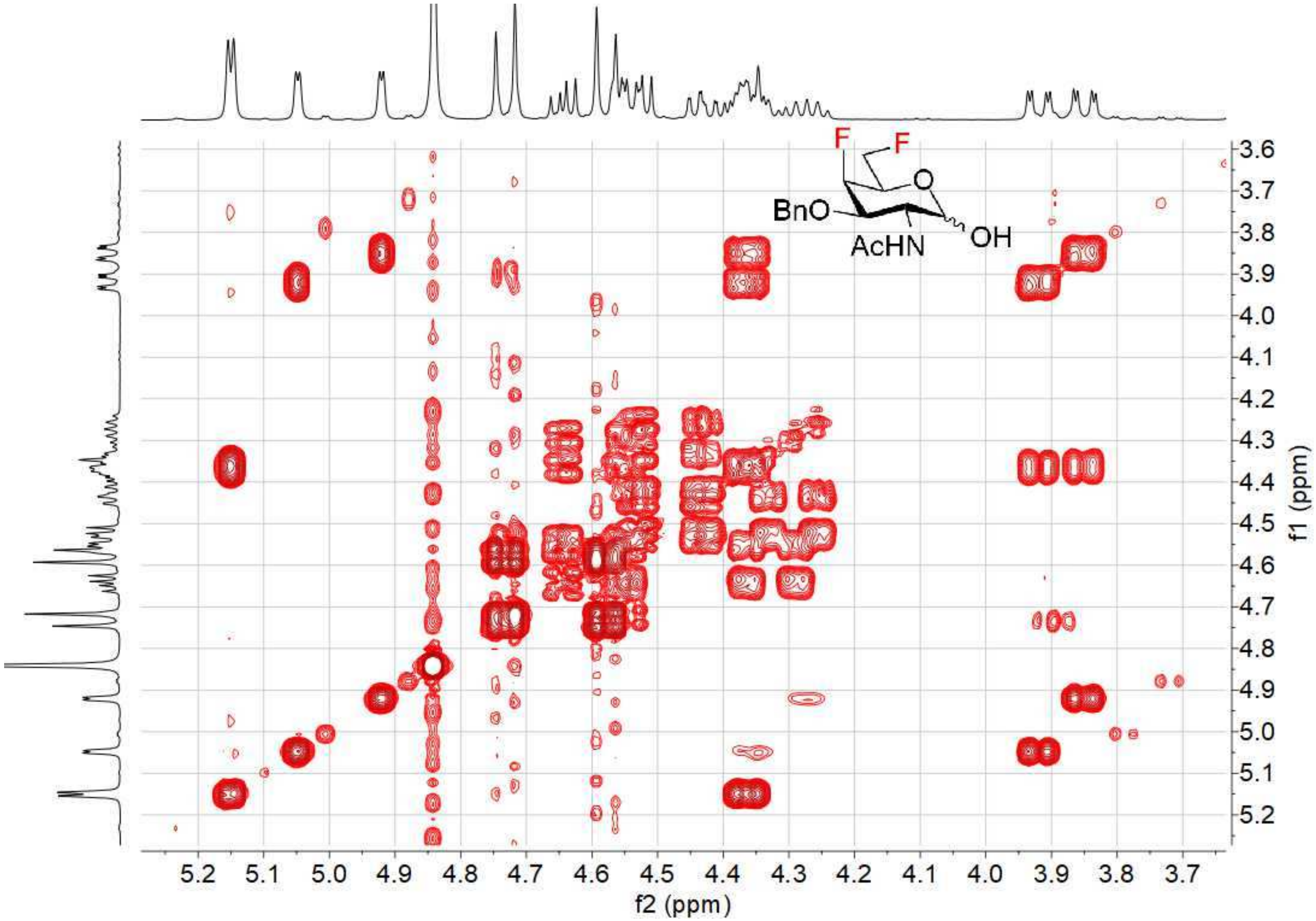

^{1}H-^{13}C HSQC NMR 57

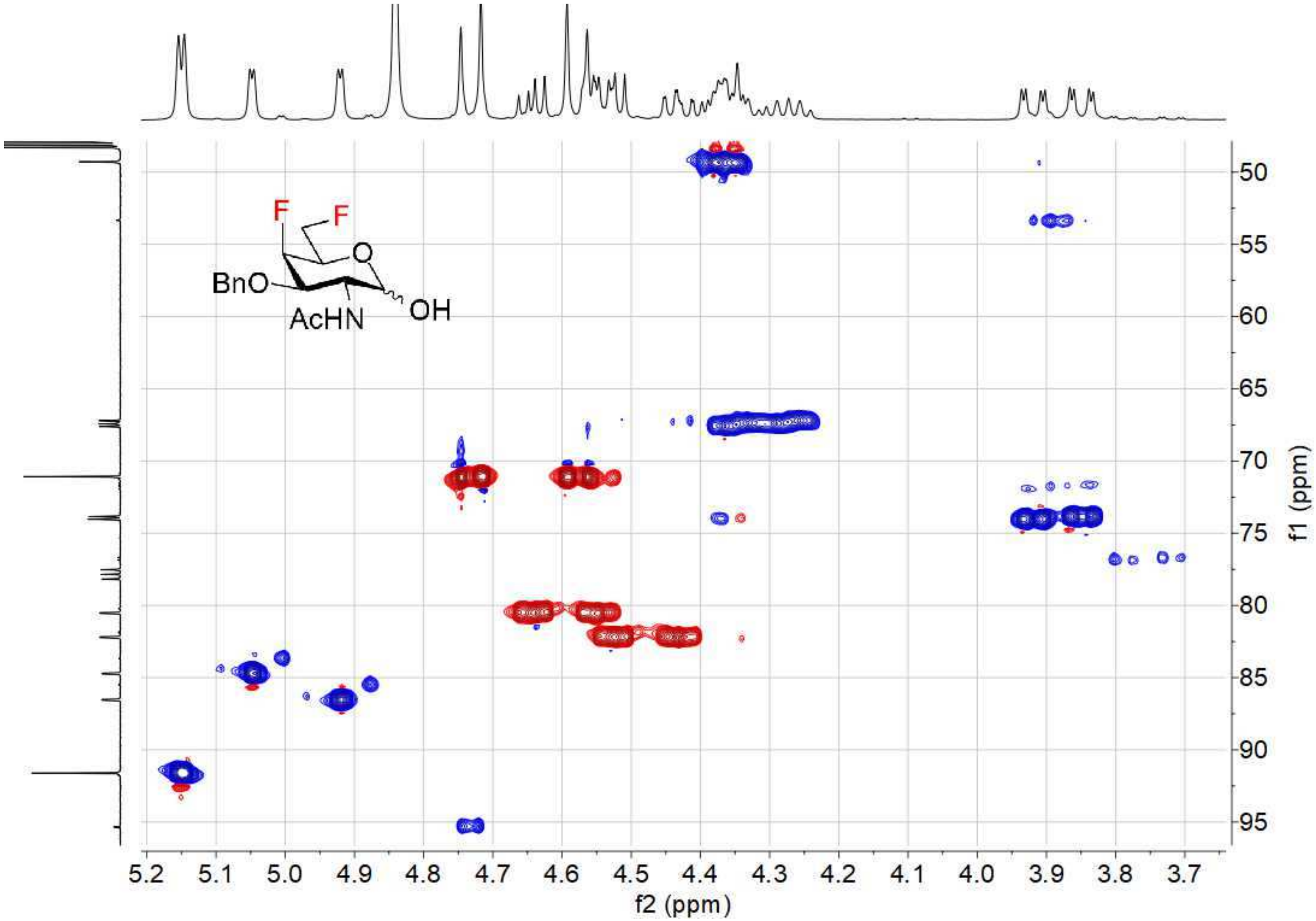

NMR COMPOUND 58

^{1}H NMR (400 MHz, CD_3OD) 58

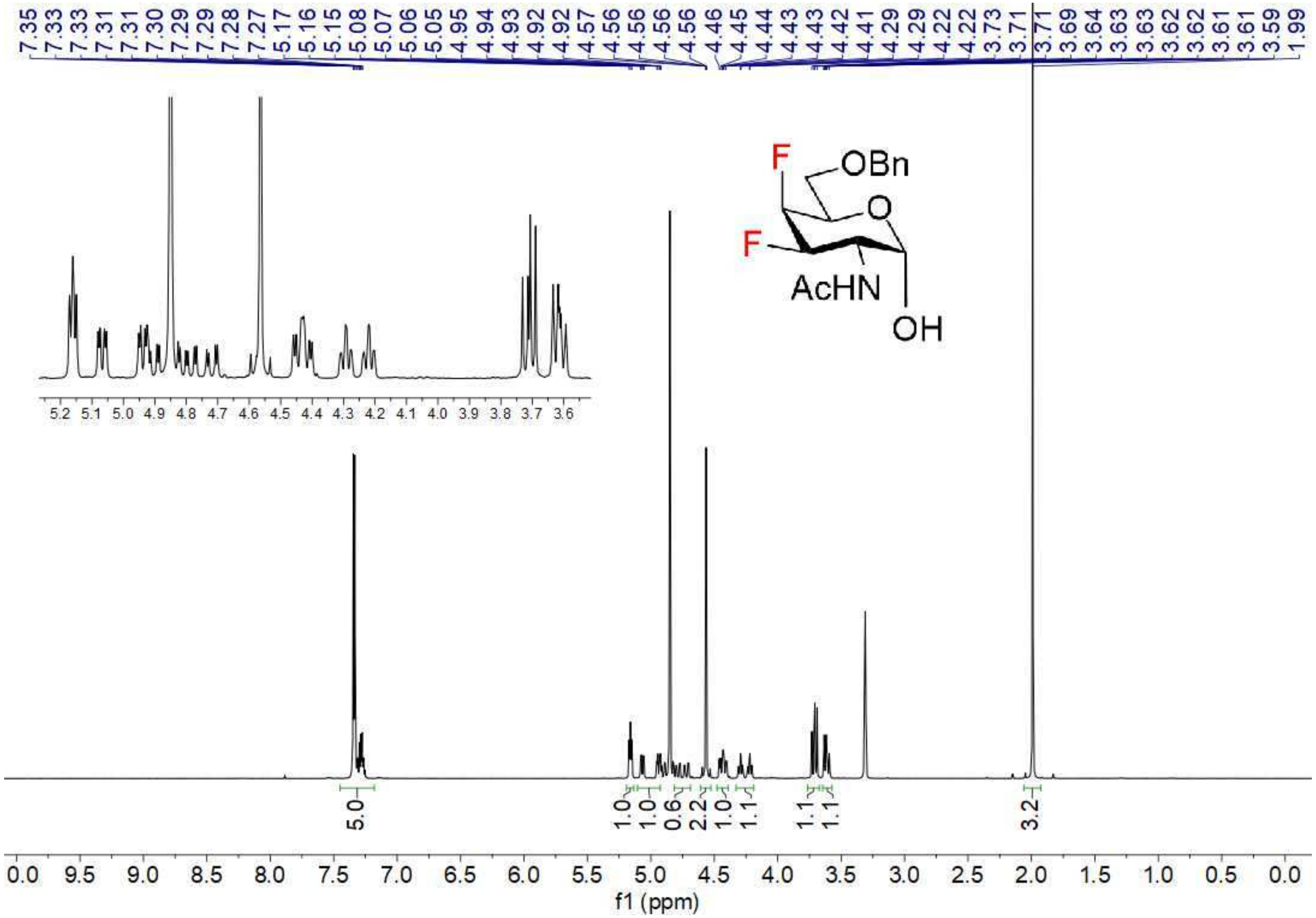

^{13}C NMR (100 MHz, CD_3OD) 58

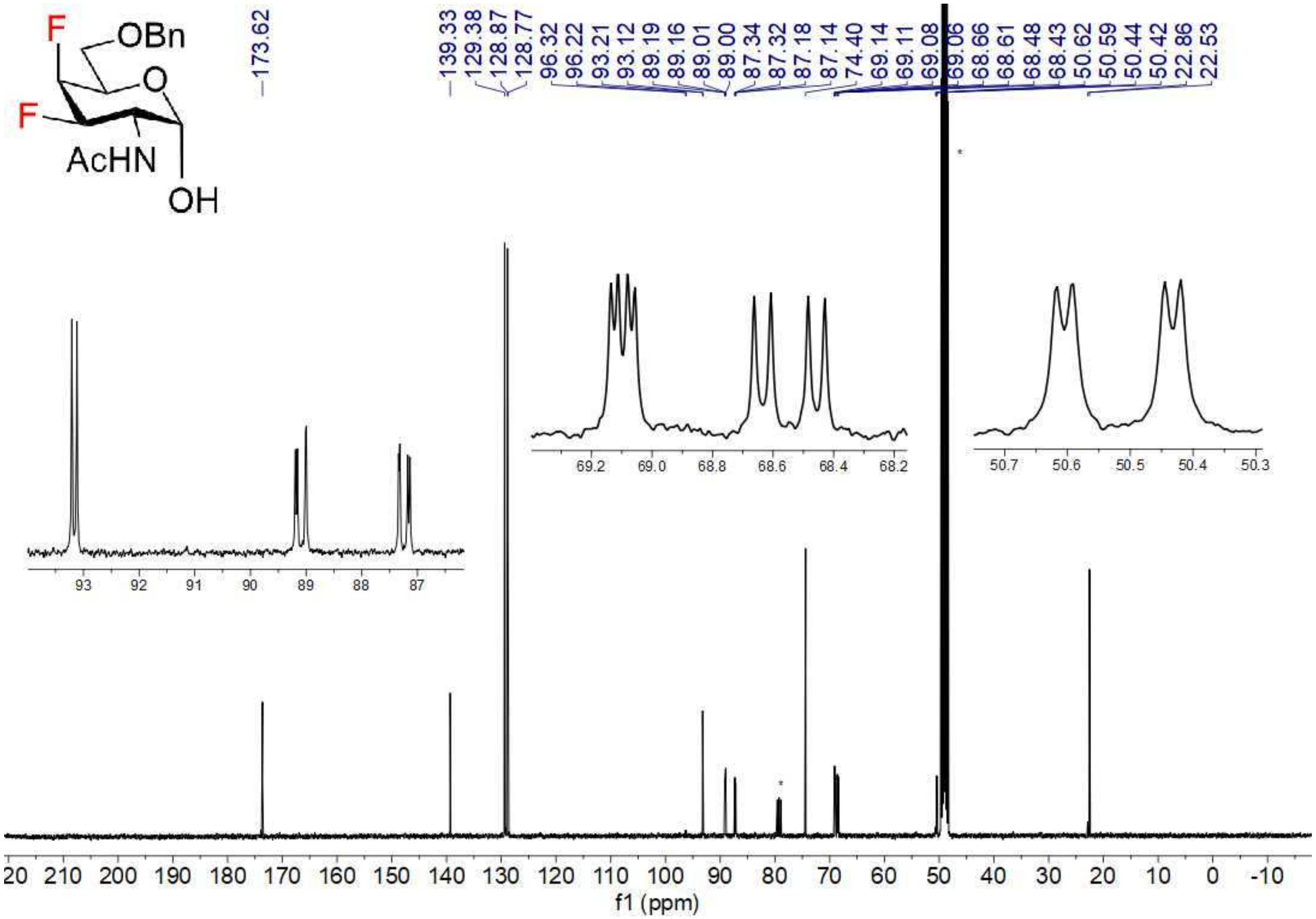

^{19}F NMR (376 MHz, CD_3OD) 58

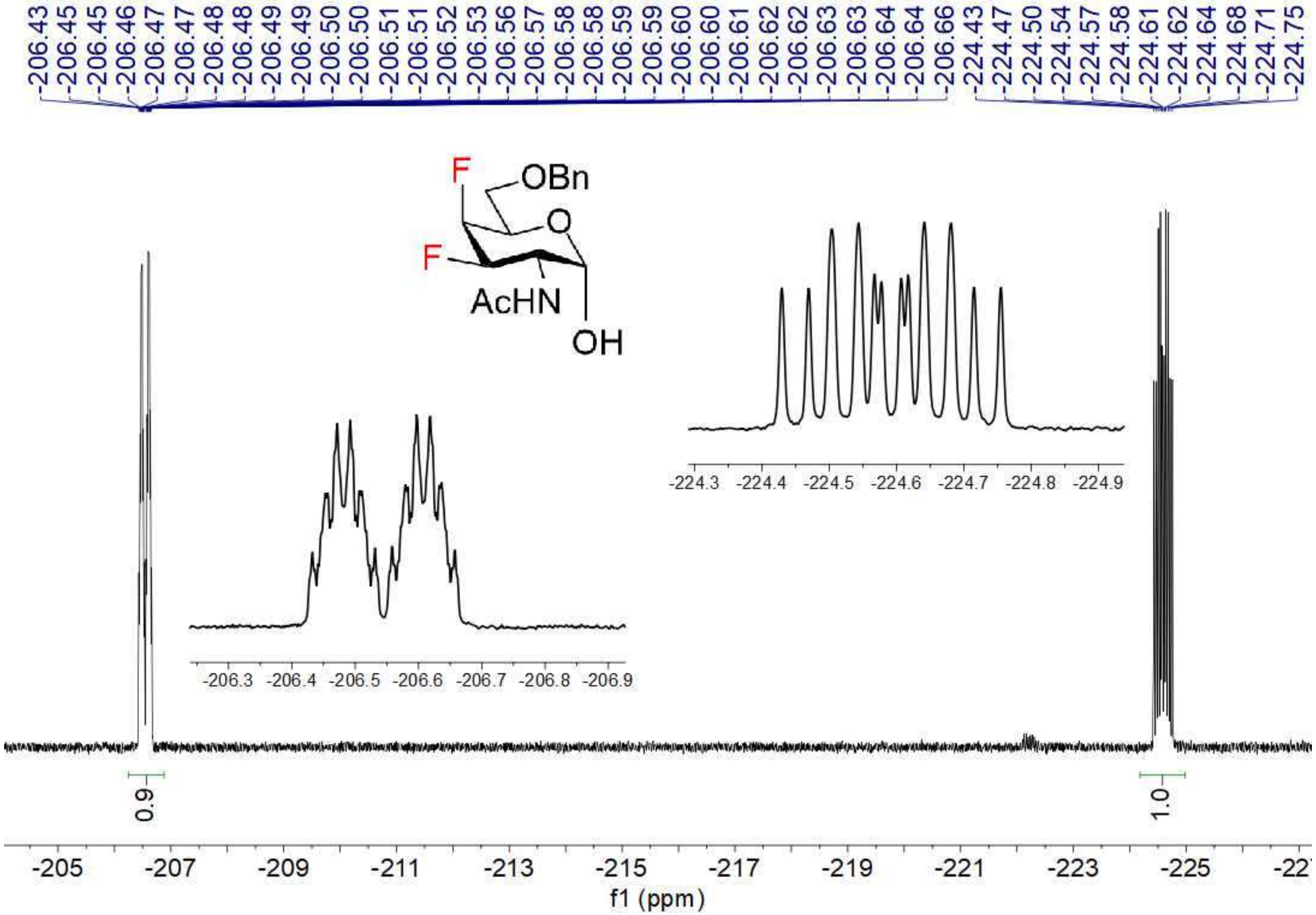

^{1}H-^{1}H COSY NMR 58

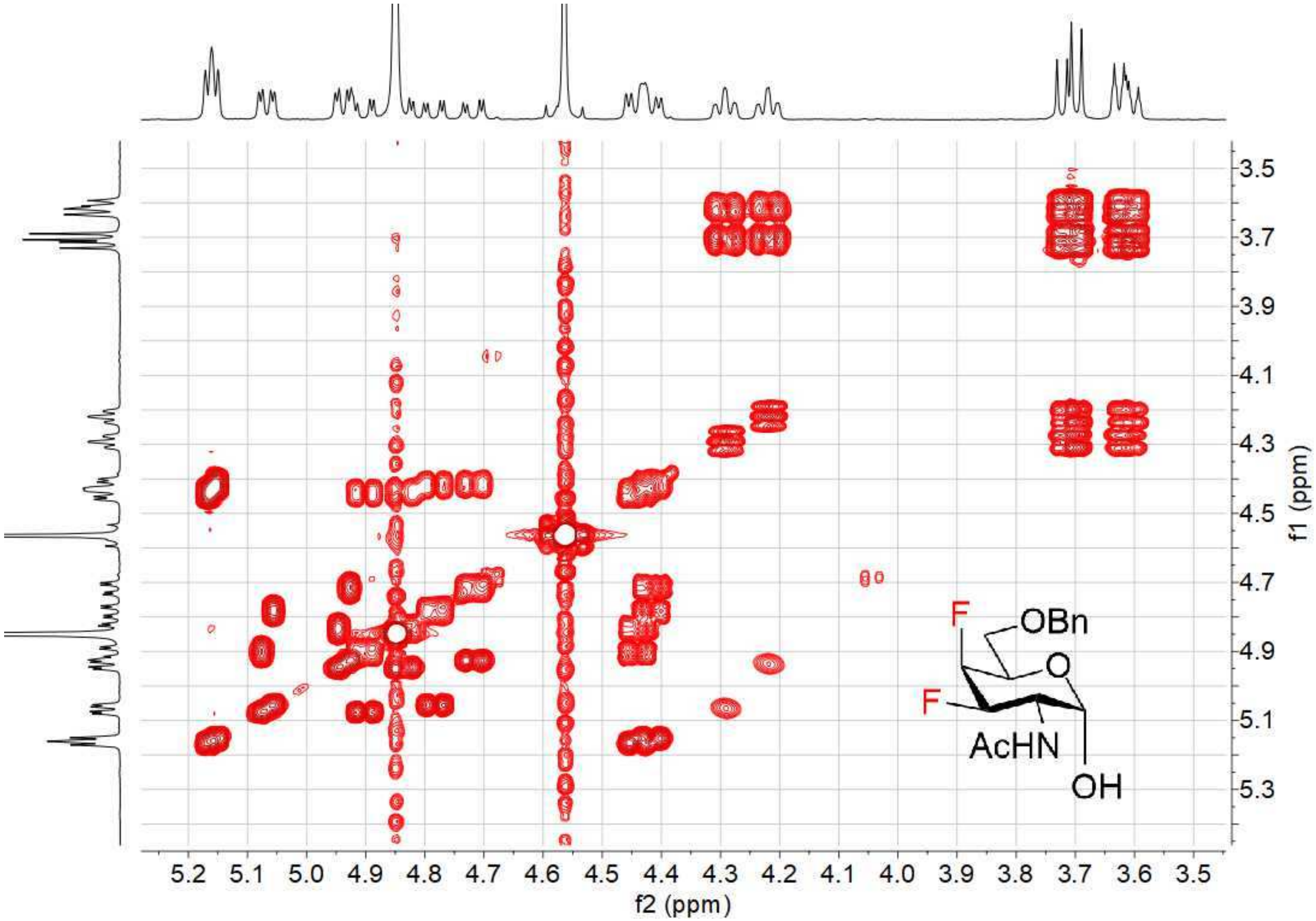

^{1}H-^{13}C HMBC 58

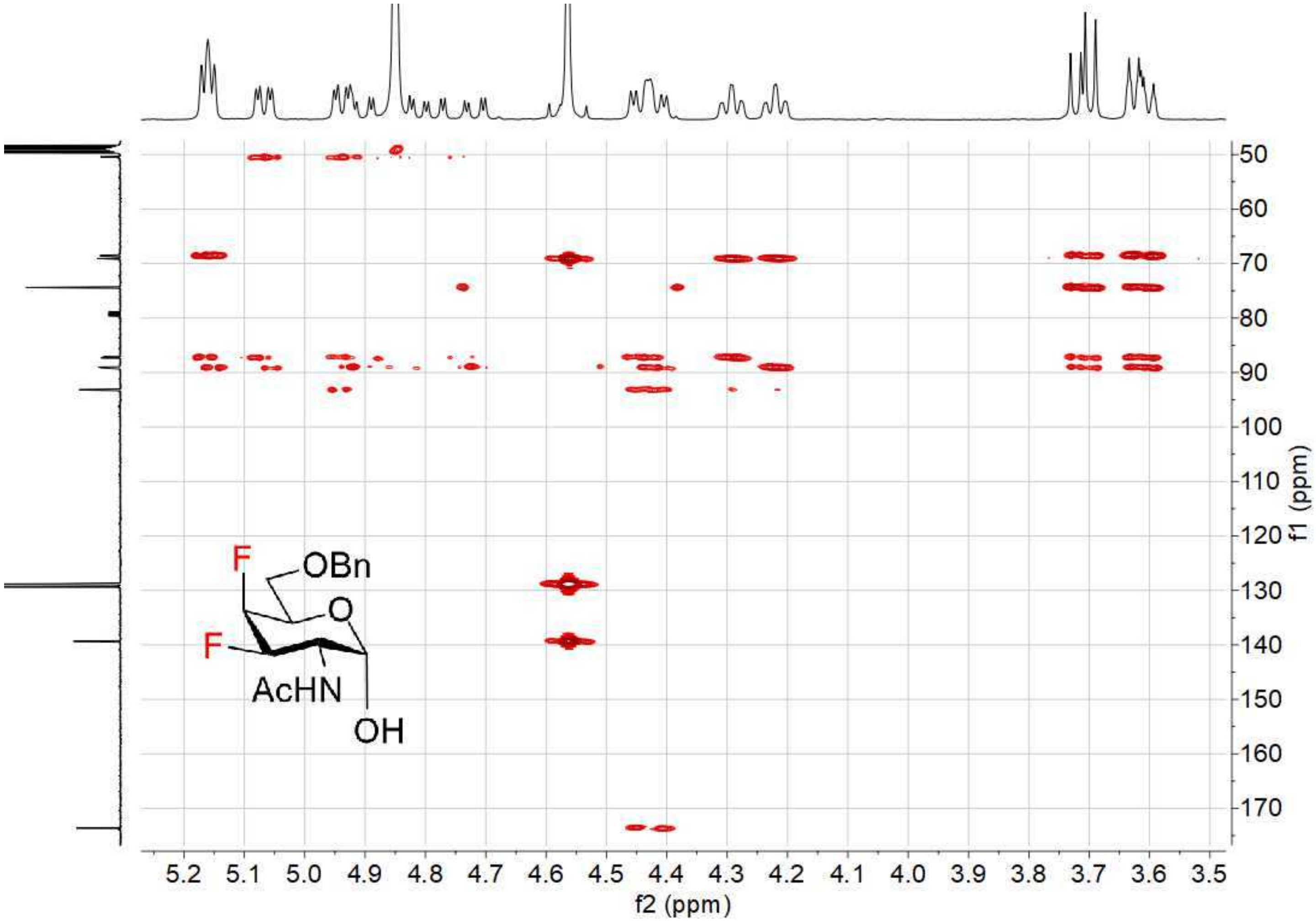

^{1}H-^{13}C HSQC 58

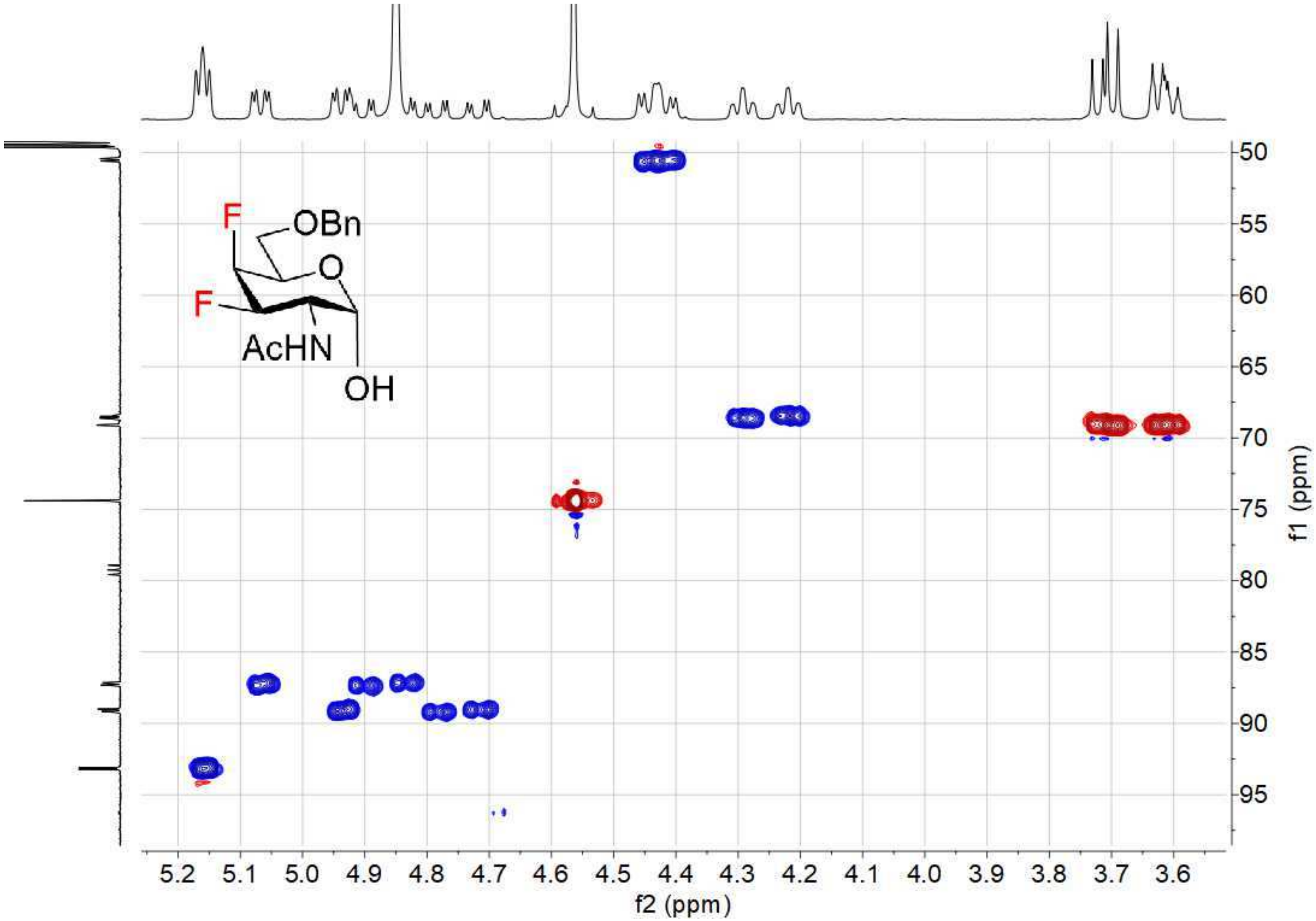

NMR COMPOUND 59

^{1}H NMR (400 MHz, CD_3OD) 59

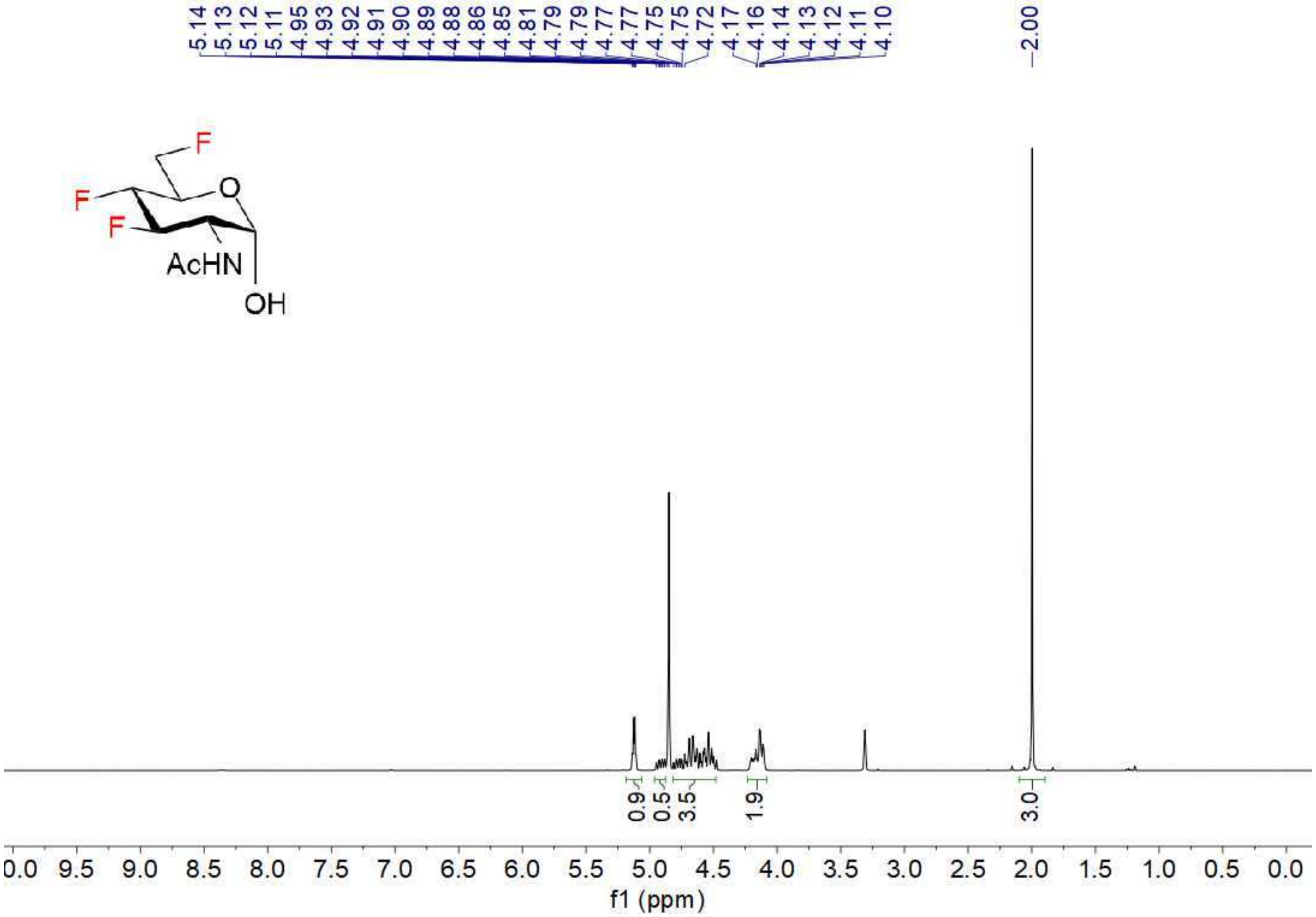

^{13}C NMR (100 MHz, CD_3OD) 59

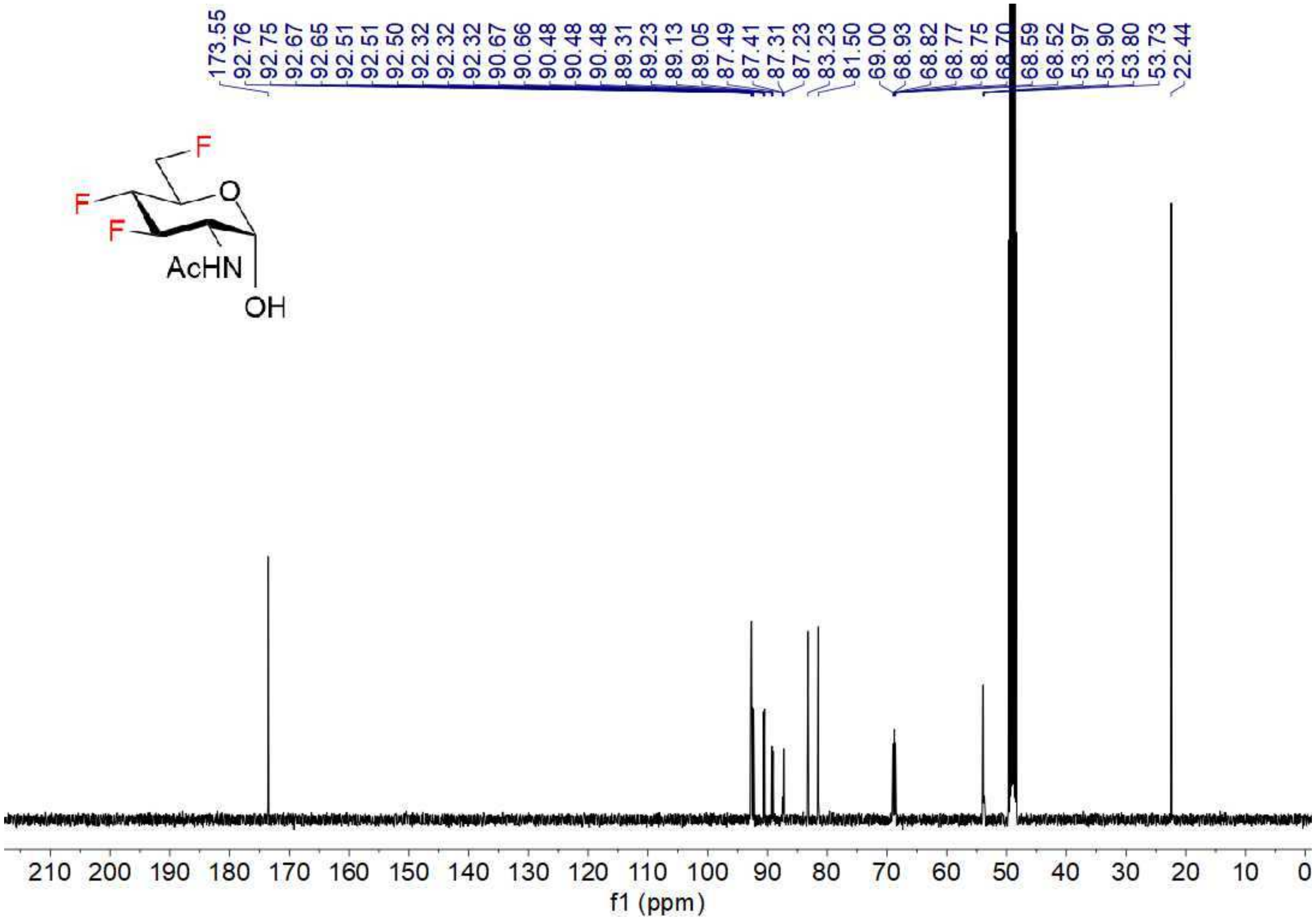

^{19}F NMR (376 MHz, CD$_3$OD) 59

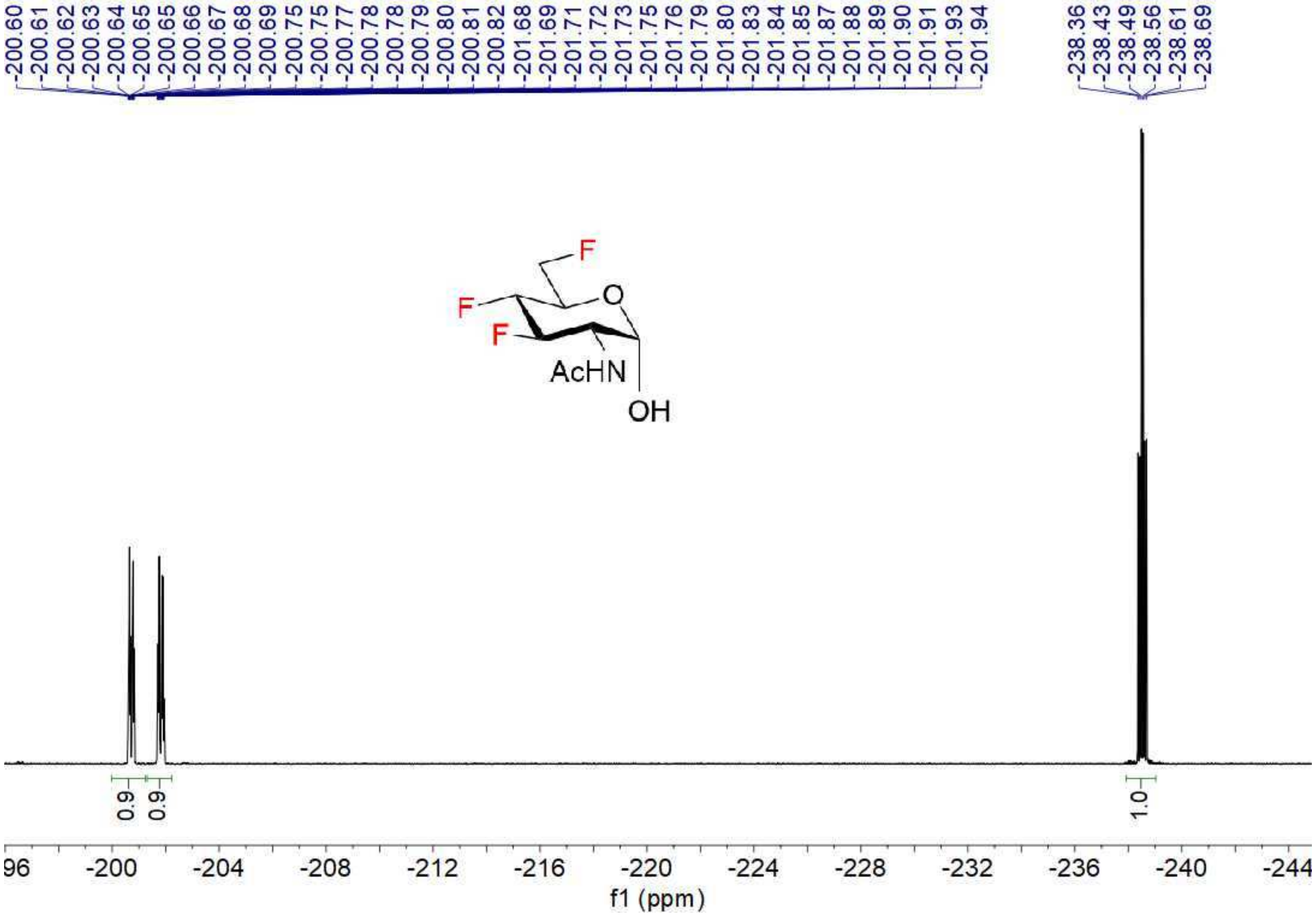

^{1}H-^{1}H COSY 59

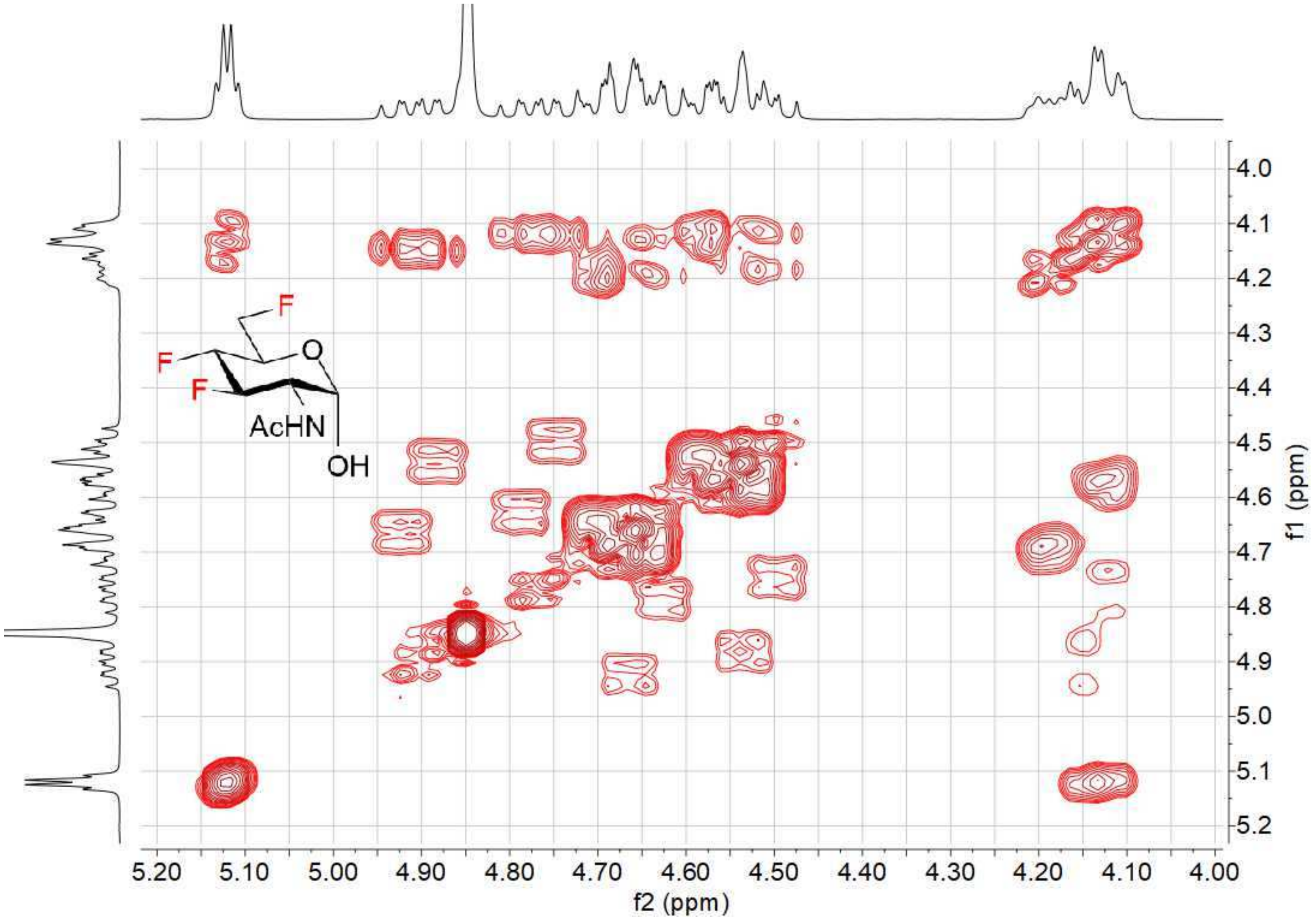

F
F
F
O
AcHN
OH
4.0
4.1
4.2
4.3
4.4
4.5
4.6
4.7
4.8
4.9
5.0
5.1
5.2
f1 (ppm)
5.20
5.10
5.00
4.90
4.80
4.70
4.60
4.50
4.40
4.30
4.20
4.10
4.00
f2 (ppm)

^{1}H-^{13}C HMBC 59

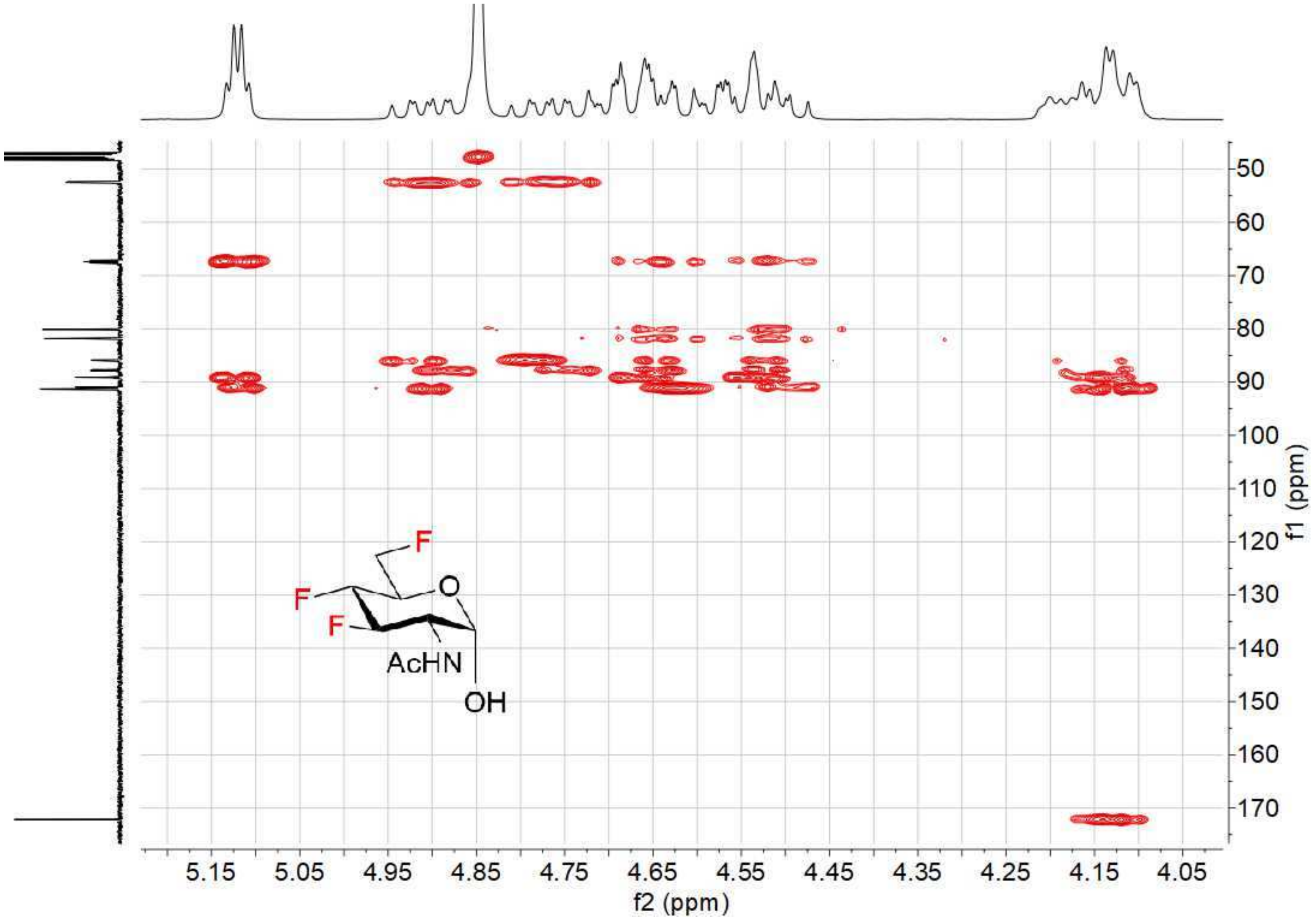

^{1}H-^{13}C HSQC 59

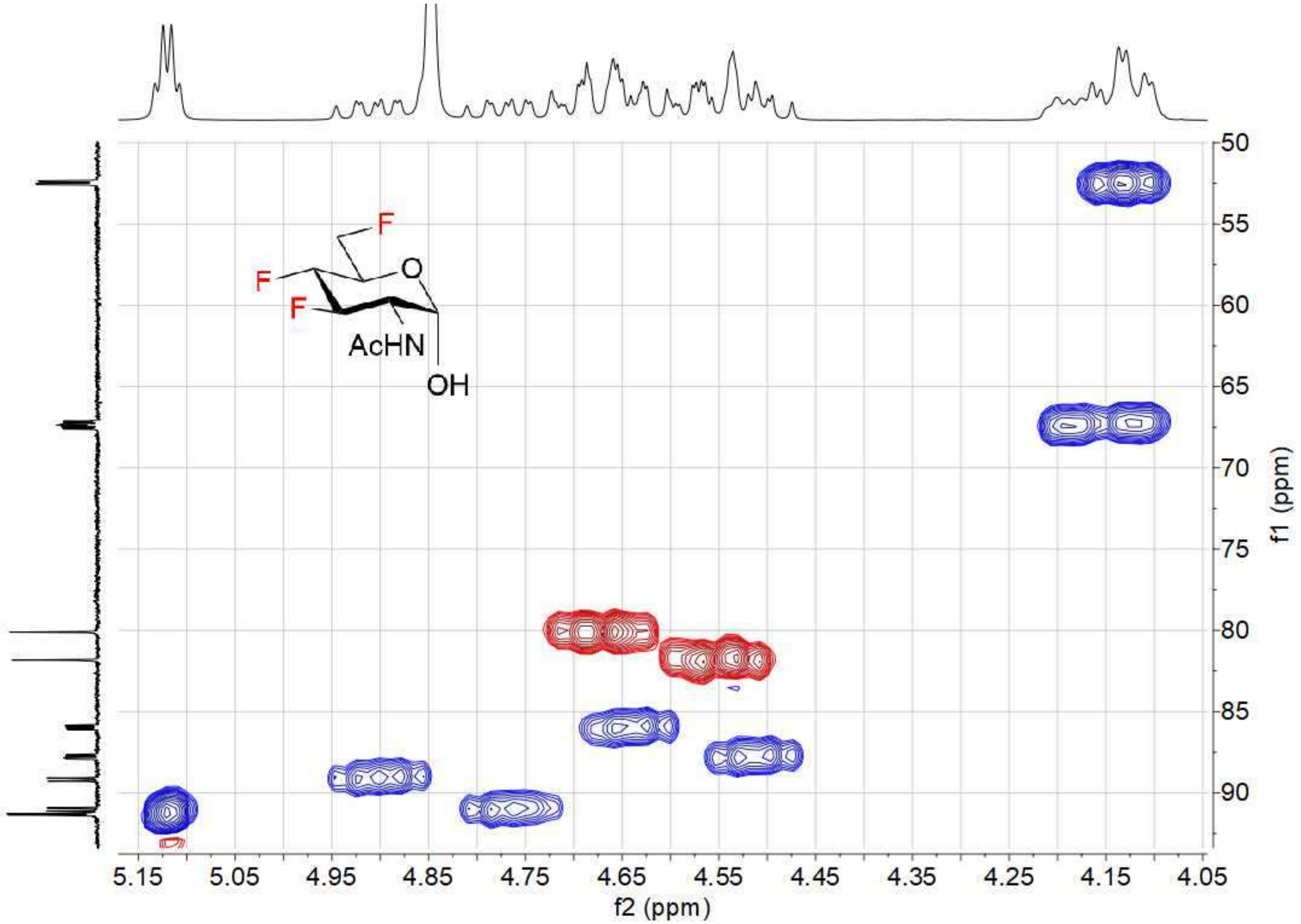

NMR COMPOUND 60

^{1}H NMR (400 MHz, CD_3OD) 60 (α/β ca. 10/1)

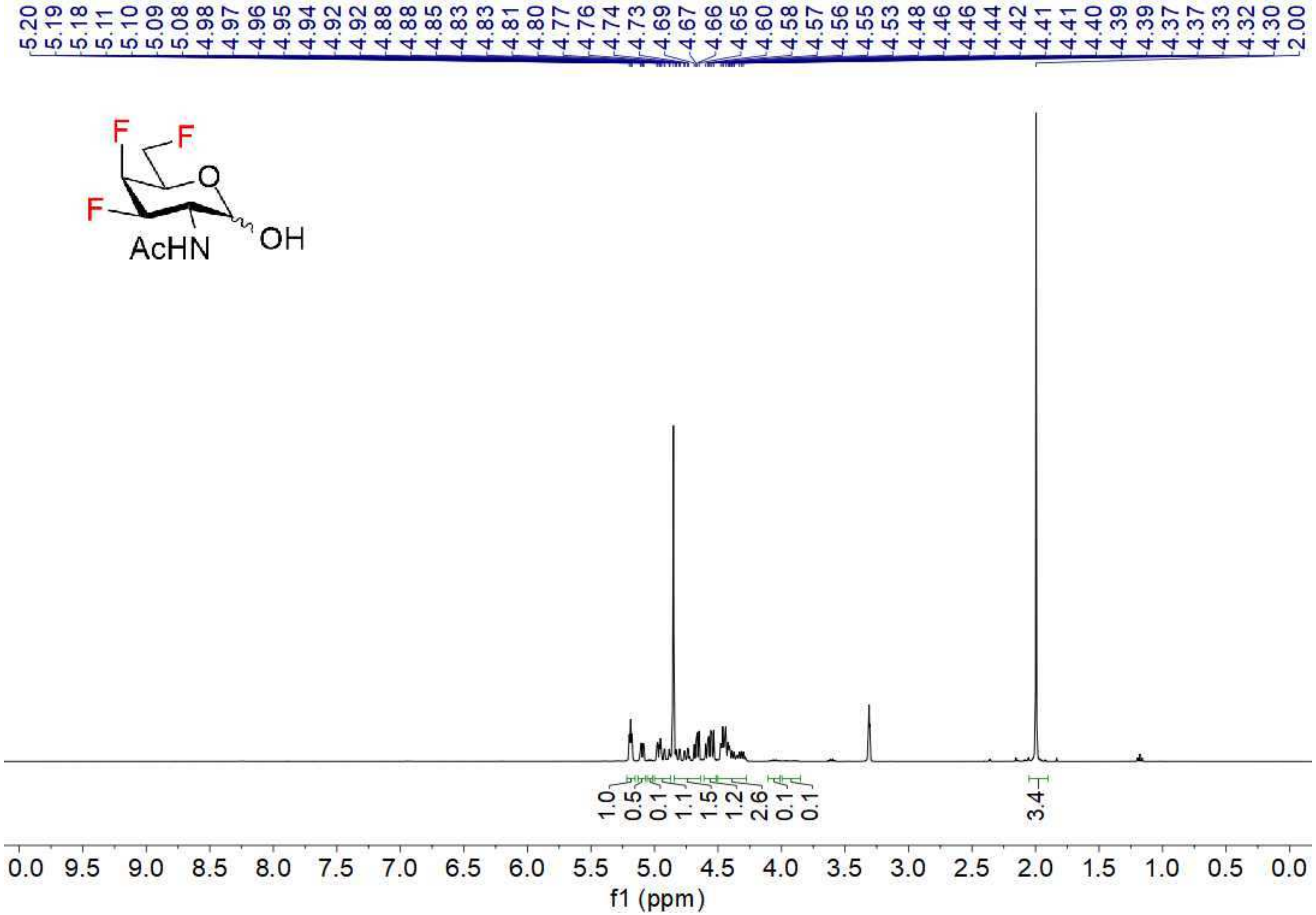

^{13}C NMR (100 MHz, CD_3OD) 60

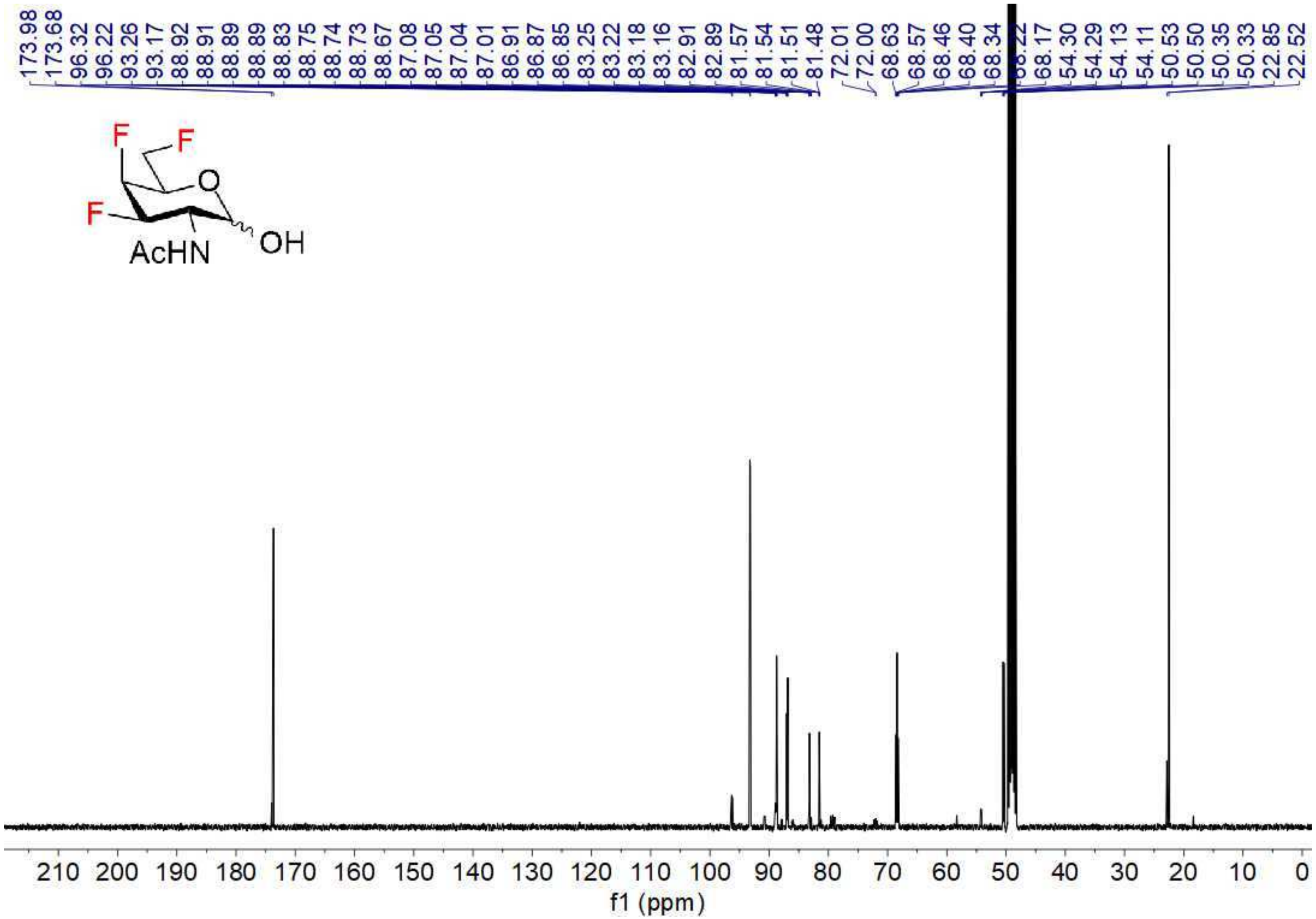

^{19}F NMR (376 MHz, CD$_3$OD) 60 (α/β ca. 10/1)

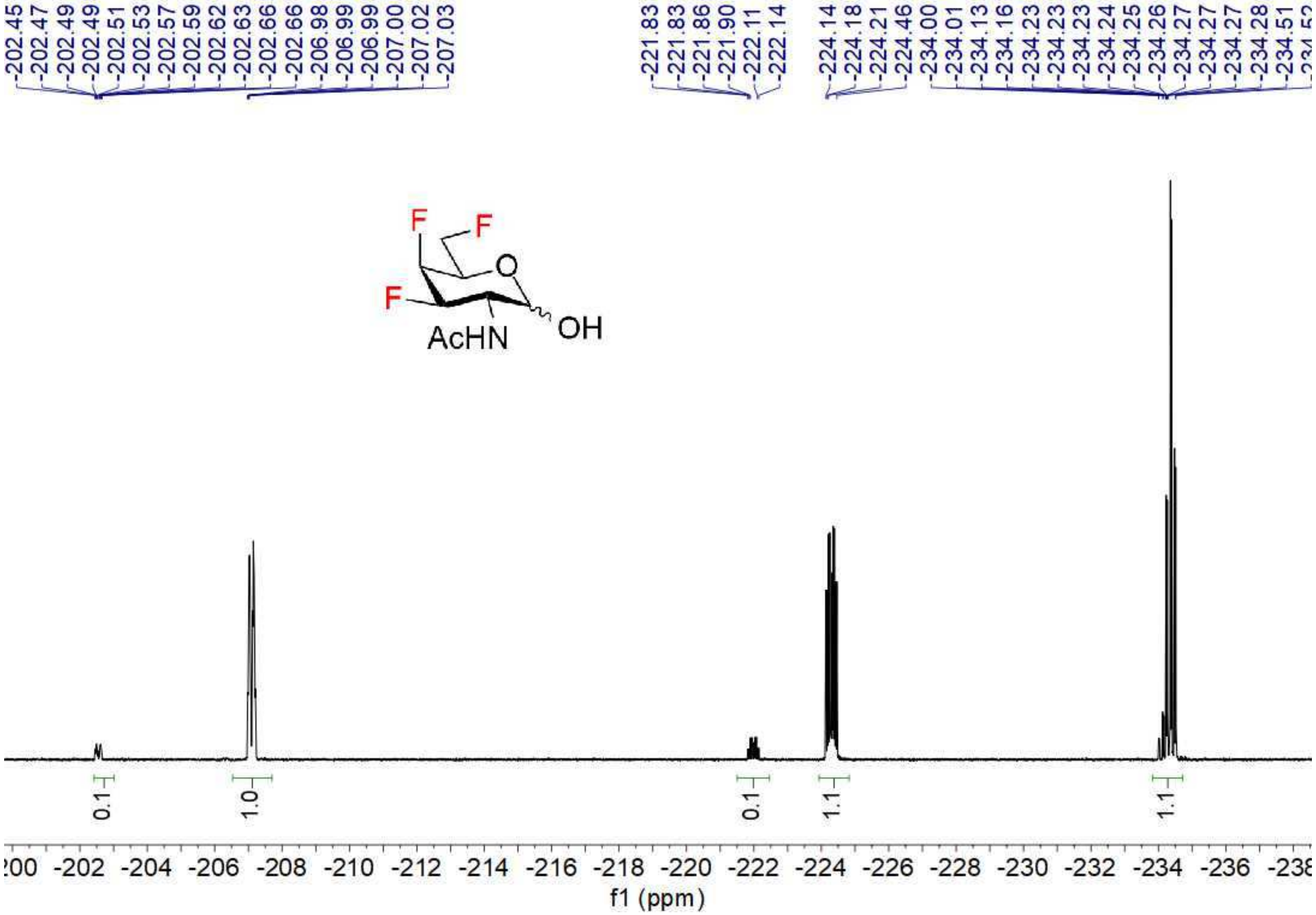

^{1}H-^{1}H COSY 60

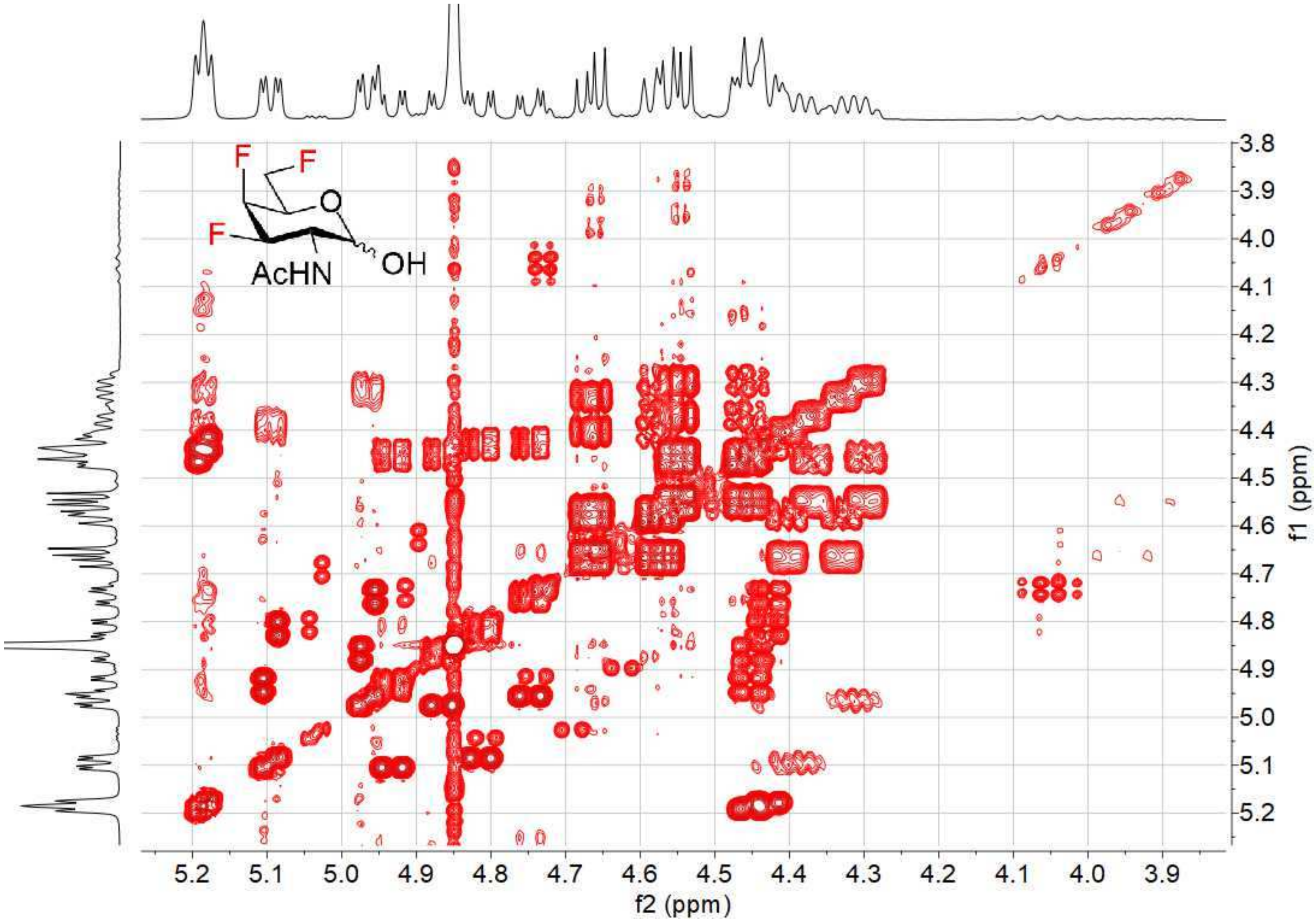
F
F
F
O
OH
AcHN
f2 (ppm)
f1 (ppm)
5.2
5.1
5.0
4.9
4.8
4.7
4.6
4.5
4.4
4.3
4.2
4.1
4.0
3.9
3.8

^{1}H-^{13}C HMBC 60

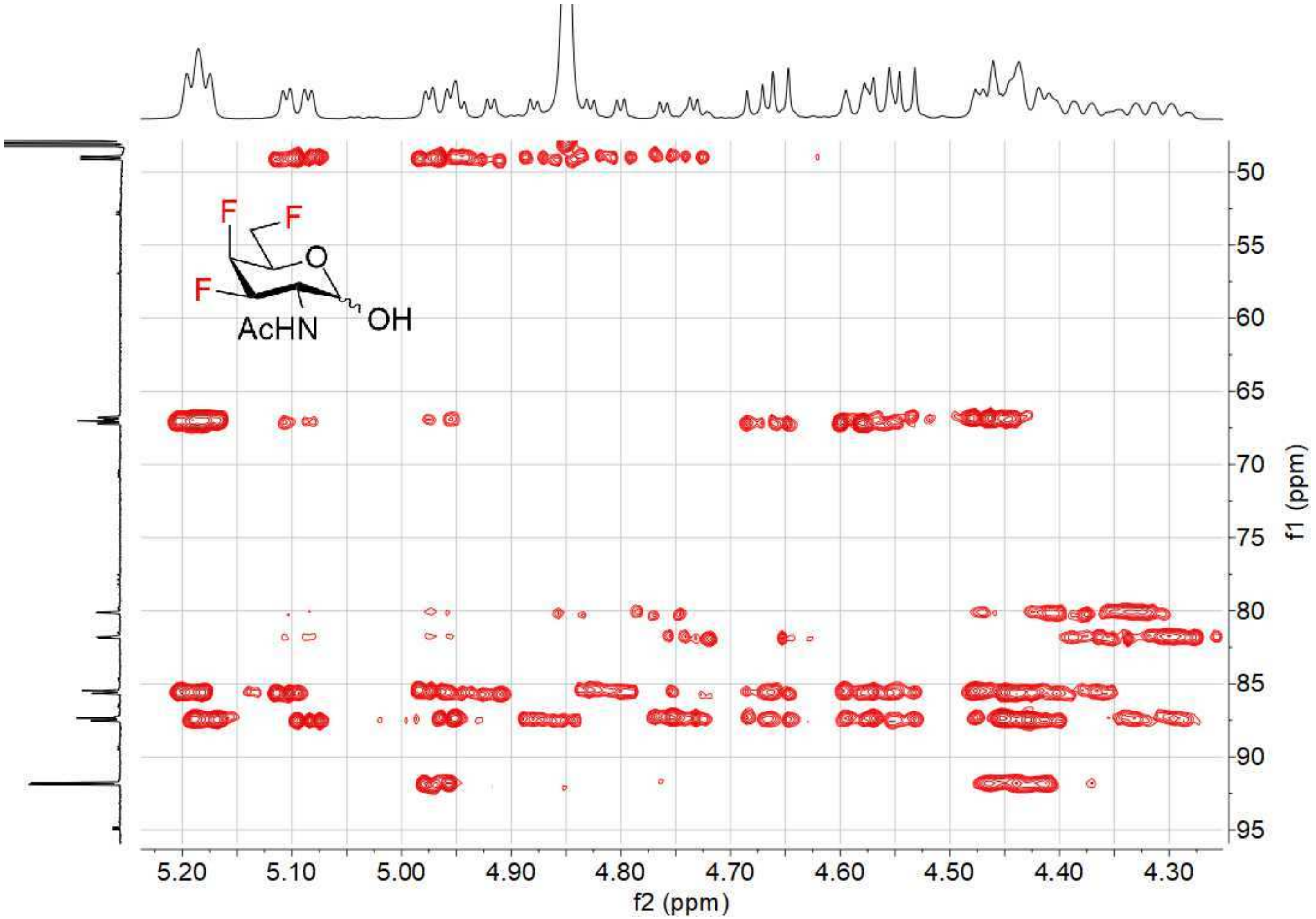

^{1}H-^{13}C HSQC 60

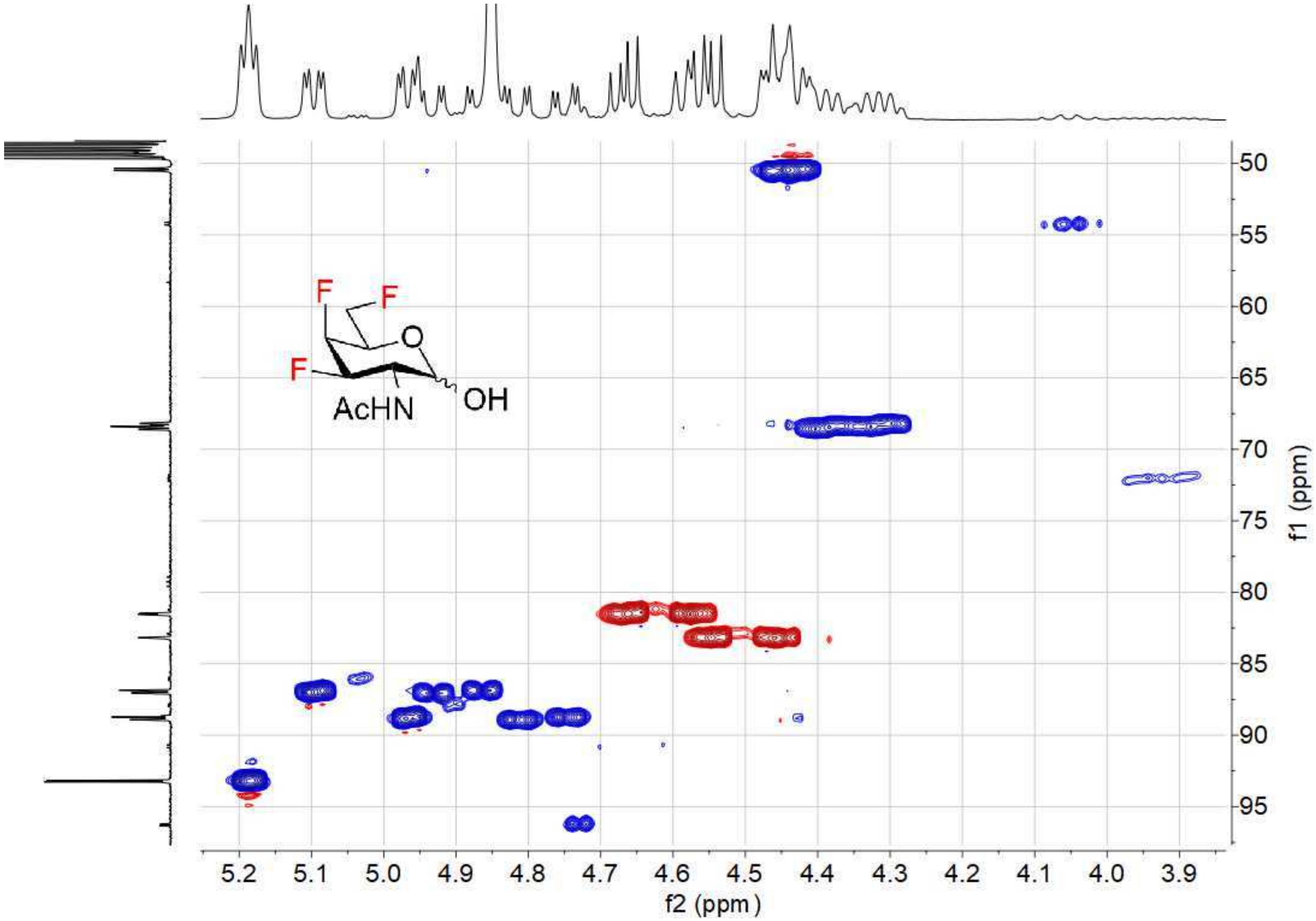

NMR COMPOUND 61

^{1}H NMR (400 MHz, CD_3OD) 61 (α/β ca. 10/1)

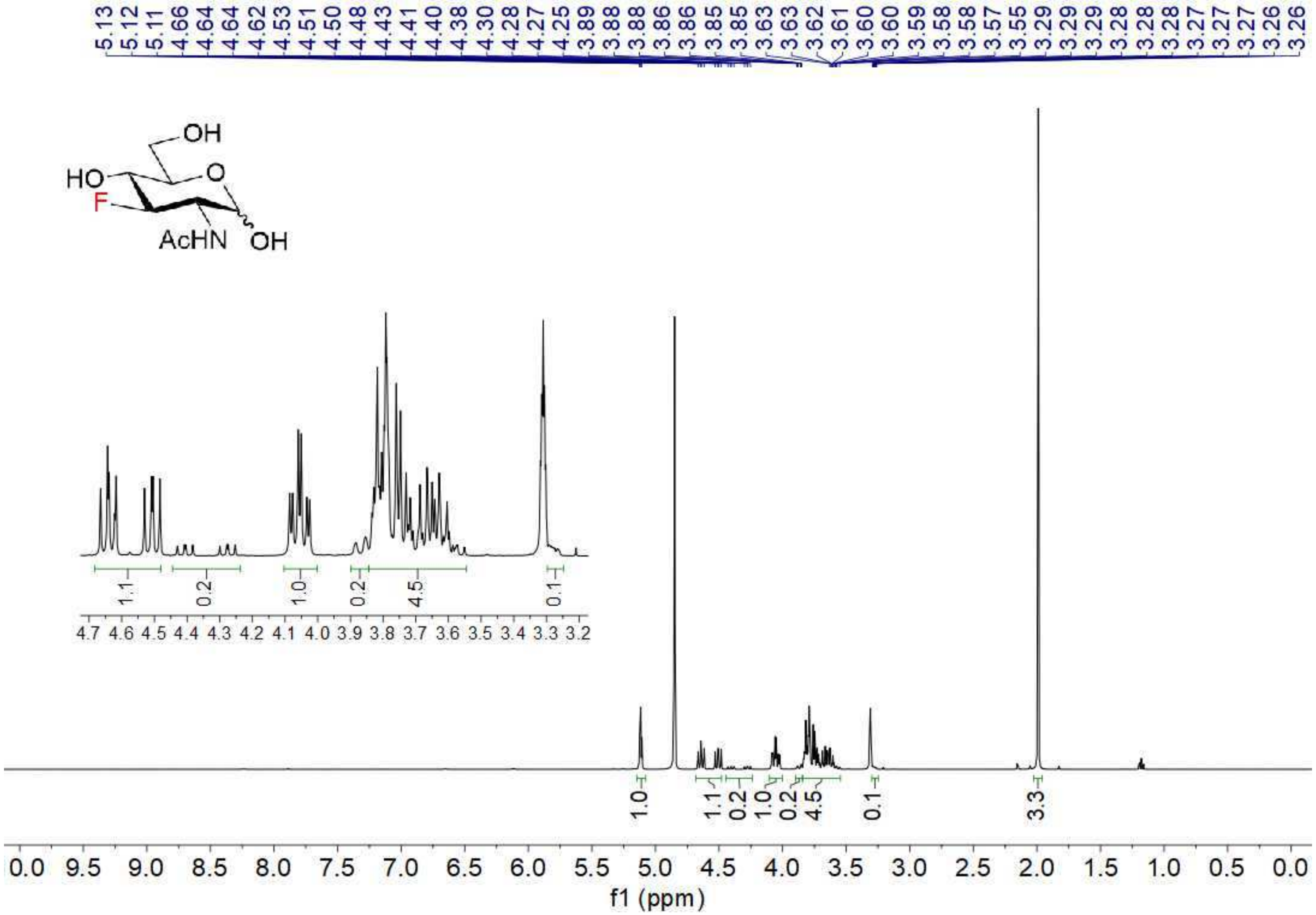

^{13}C NMR (100 MHz, CD_3OD) 61

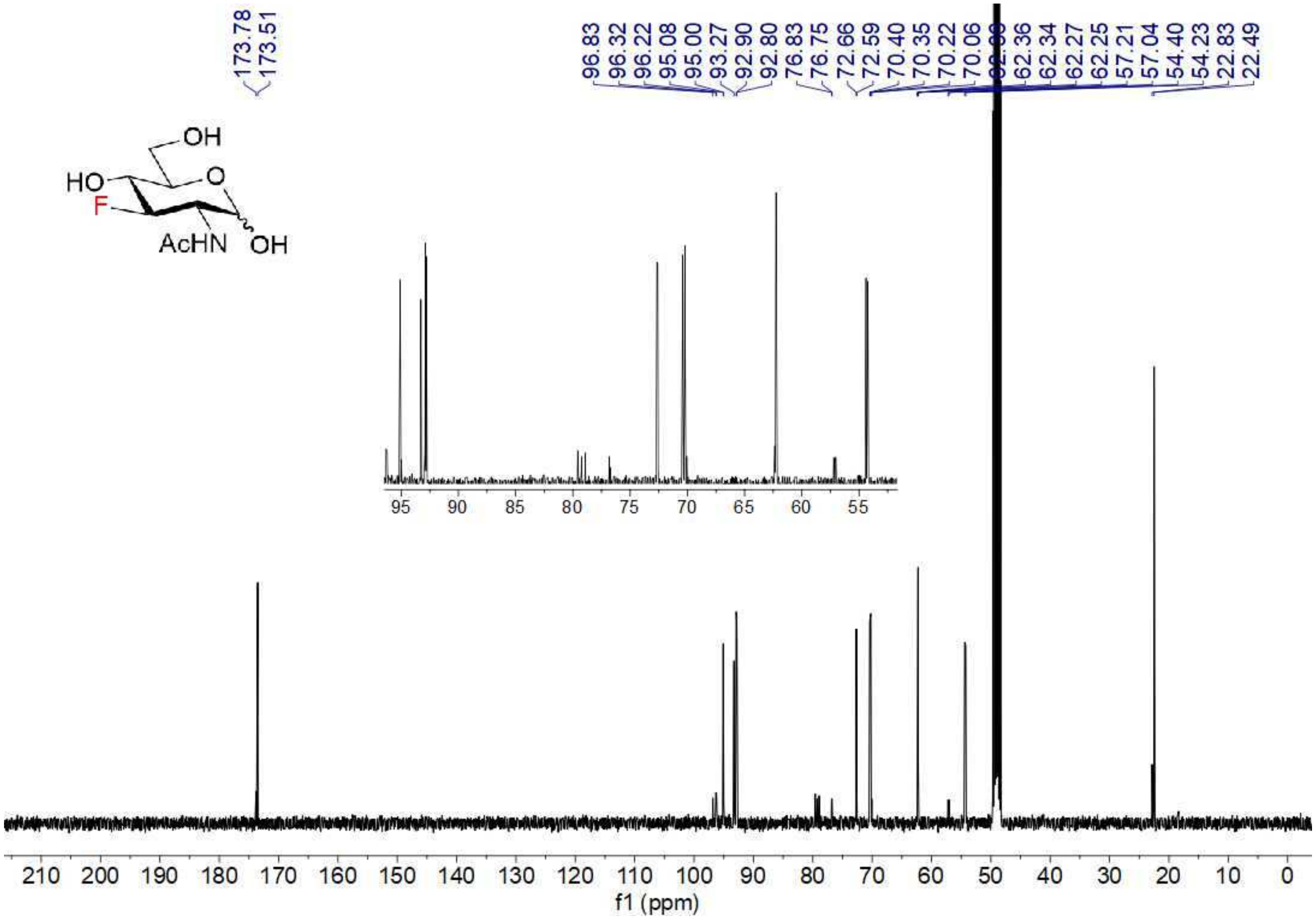

^{19}F NMR (376 MHz, CD_3OD) 61 (α/β ca. 10/1)

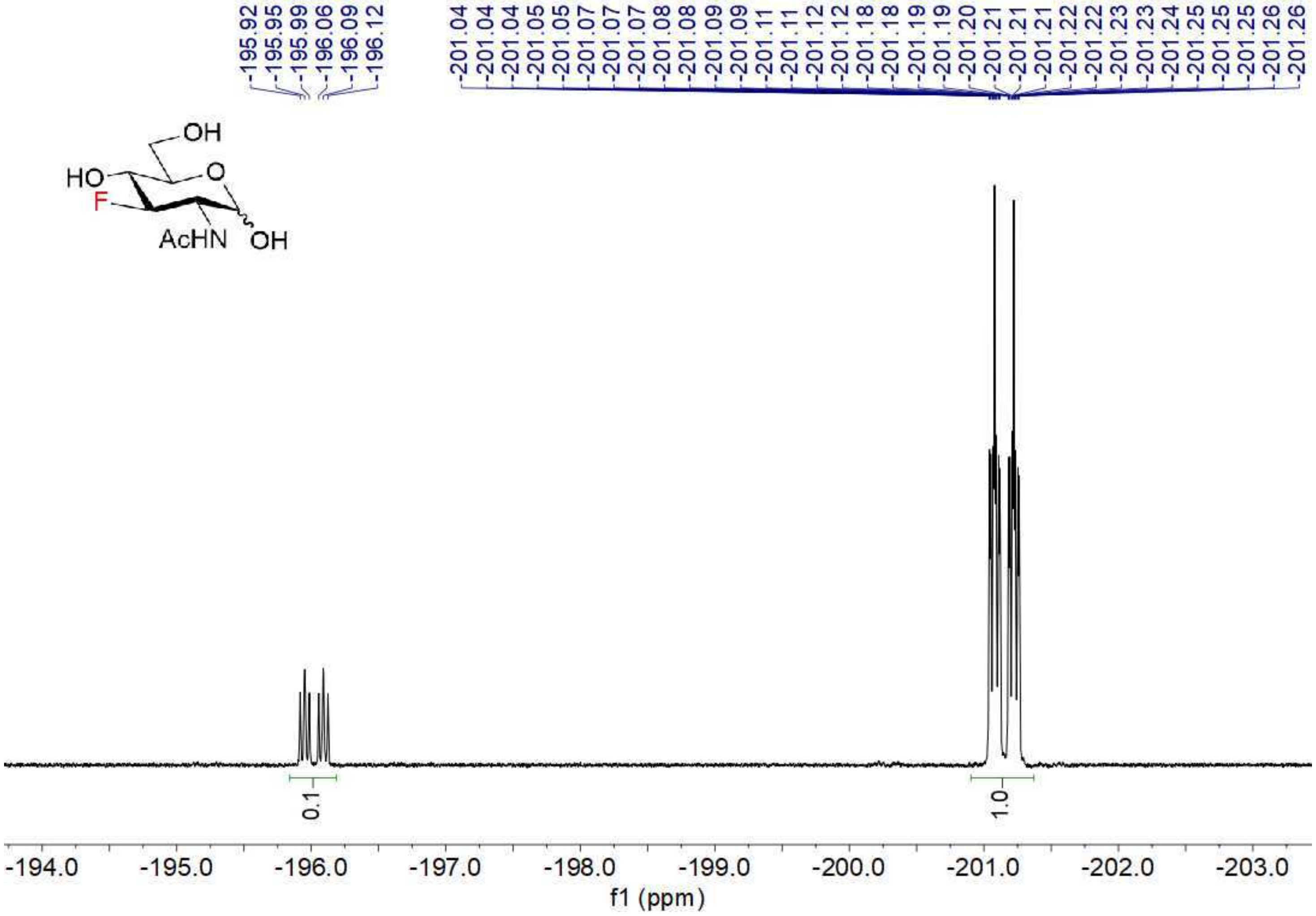

^{1}H-^{1}H COSY 61

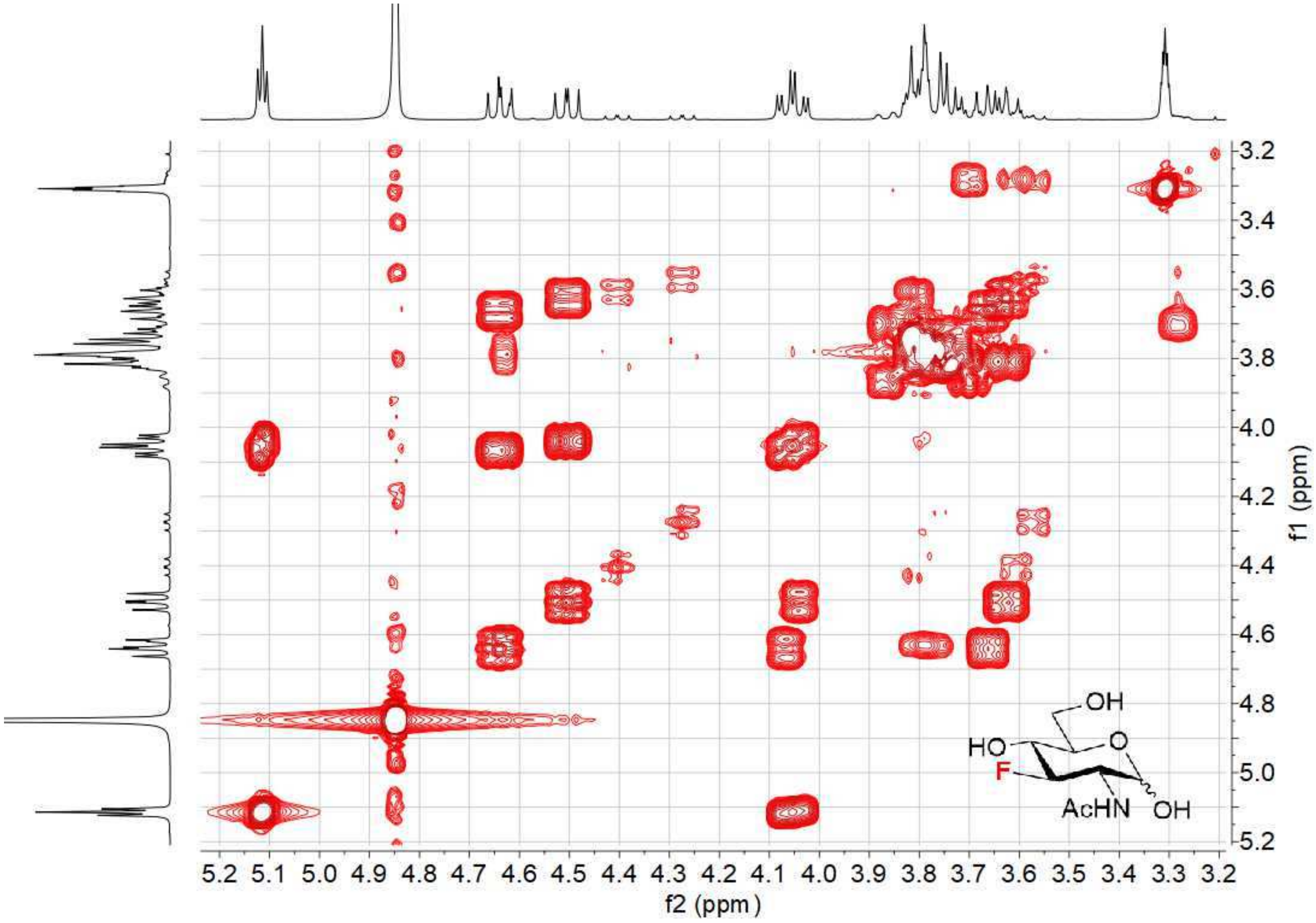
5.2 5.1 5.0 4.9 4.8 4.7 4.6 4.5 4.4 4.3 4.2 4.1 4.0 3.9 3.8 3.7 3.6 3.5 3.4 3.3 3.2
f2 (ppm)
3.2 3.4 3.6 3.8 4.0 4.2 4.4 4.6 4.8 5.0 5.2
f1 (ppm)
OH
HO
O
F
AcHN
OH

^{1}H-^{13}C HSQC 61

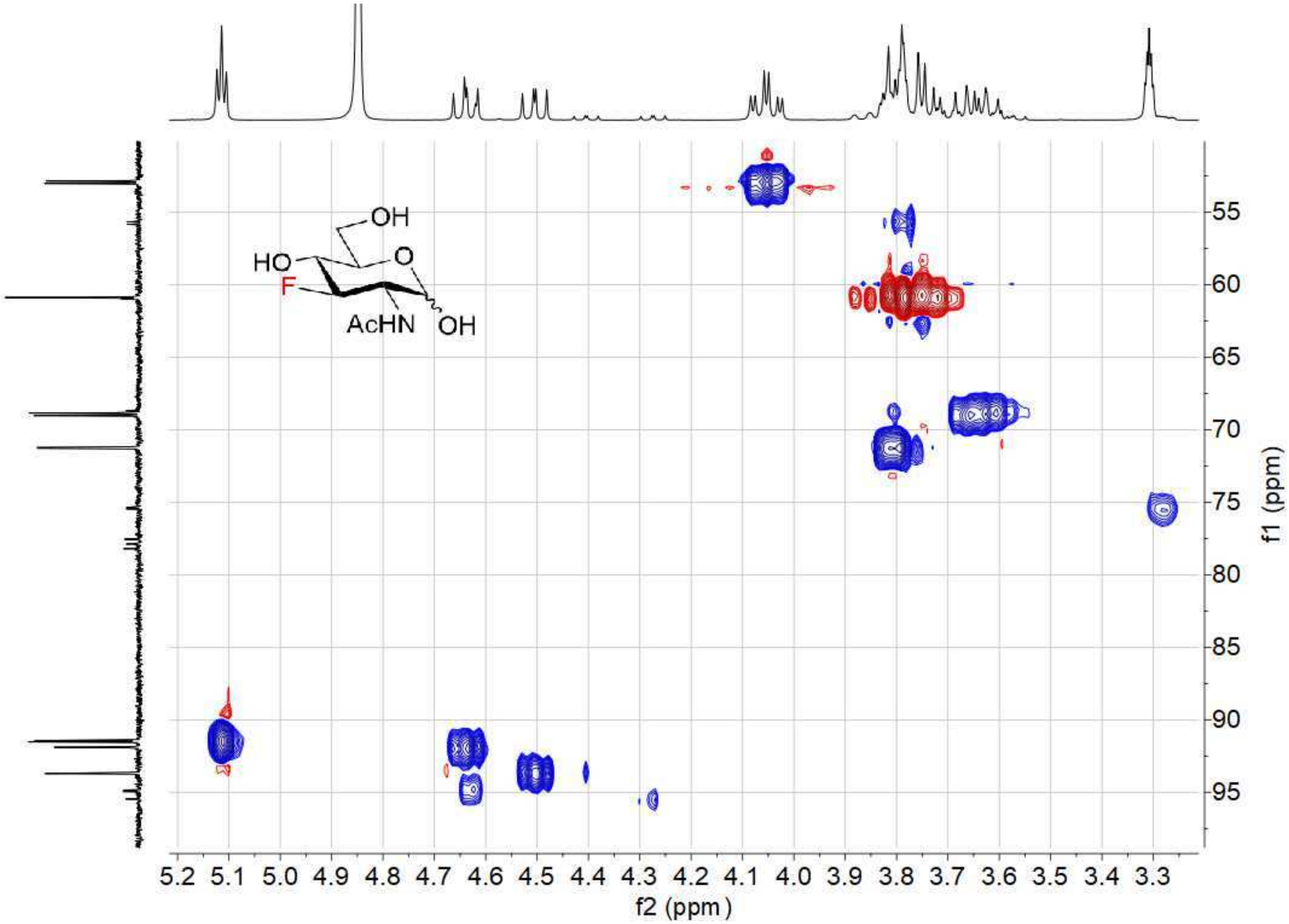

NMR COMPOUND 62

^{1}H NMR (400 MHz, CD_3OD) 62

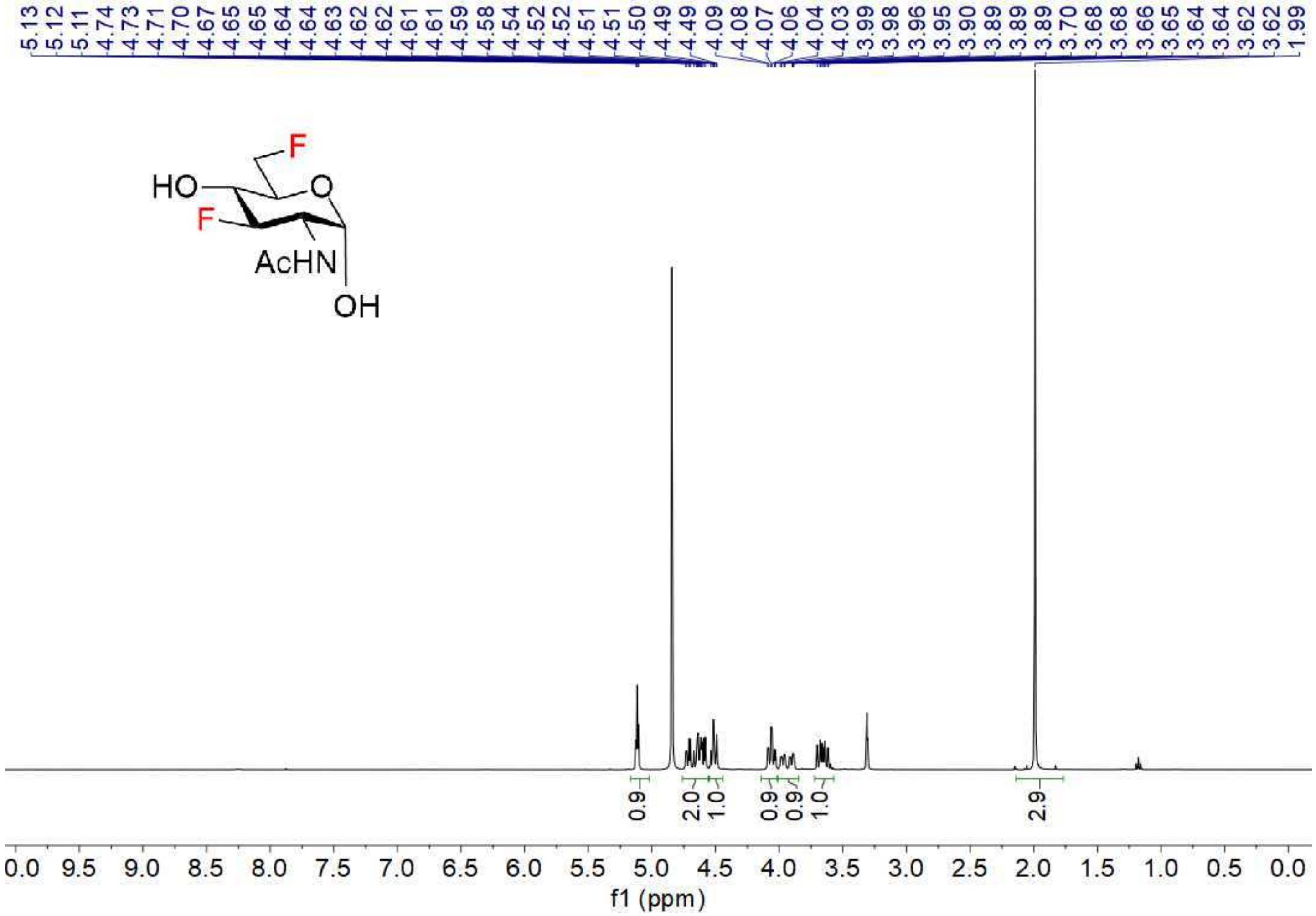

^{13}C NMR (100 MHz, CD_3OD) 62

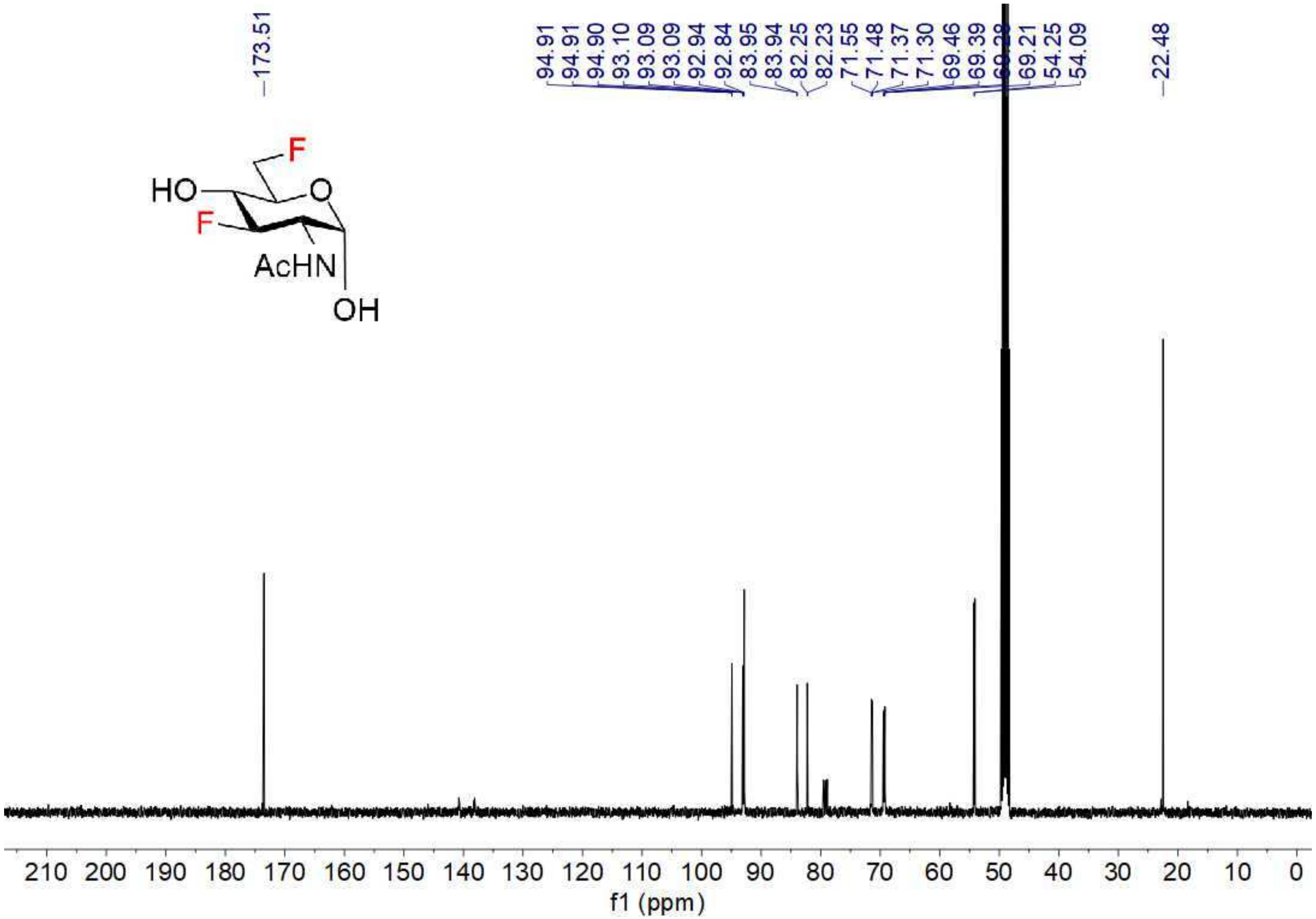

^{19}F NMR (376 MHz, CD_3OD) 62

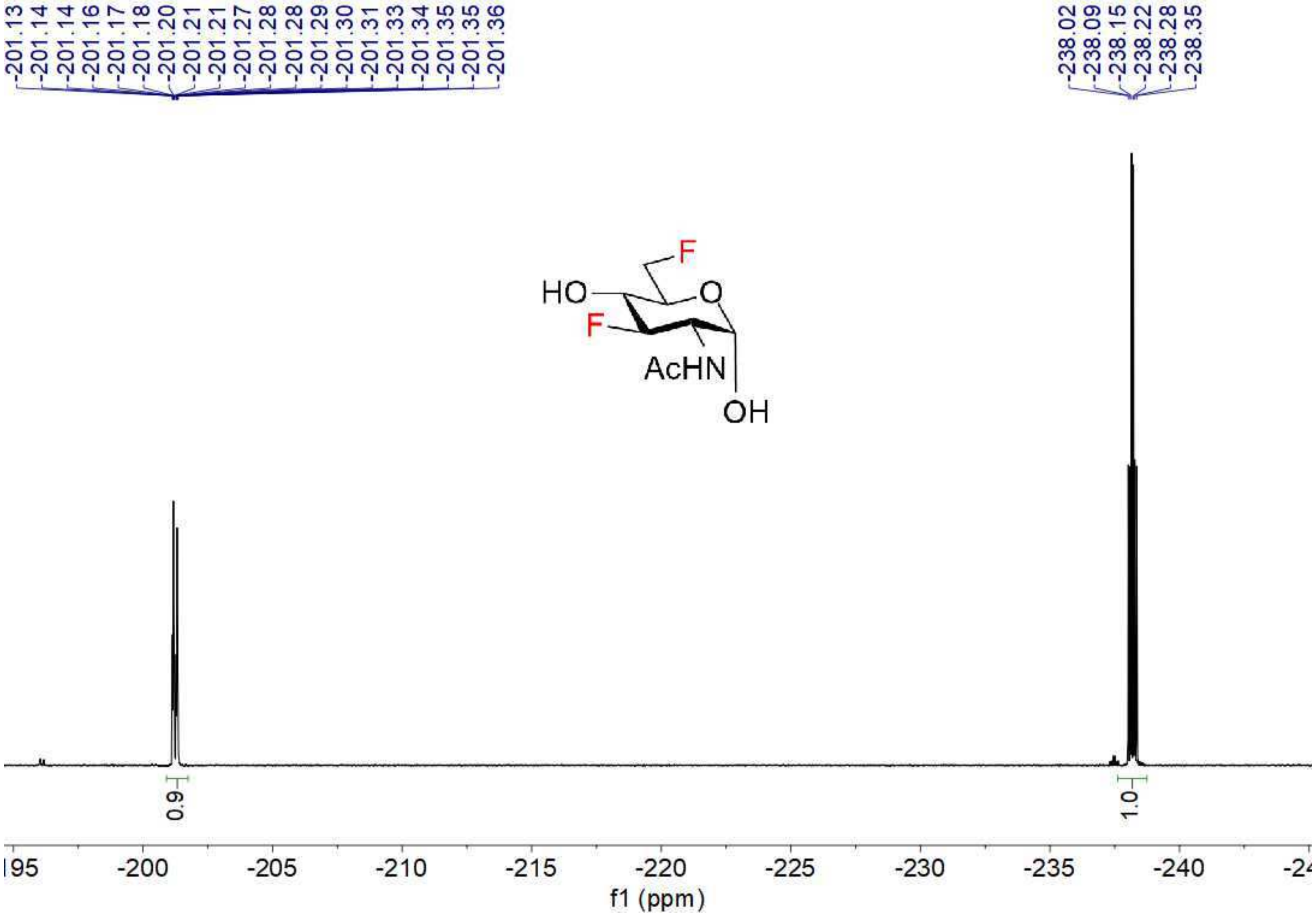

^{1}H-^{1}H COSY 62

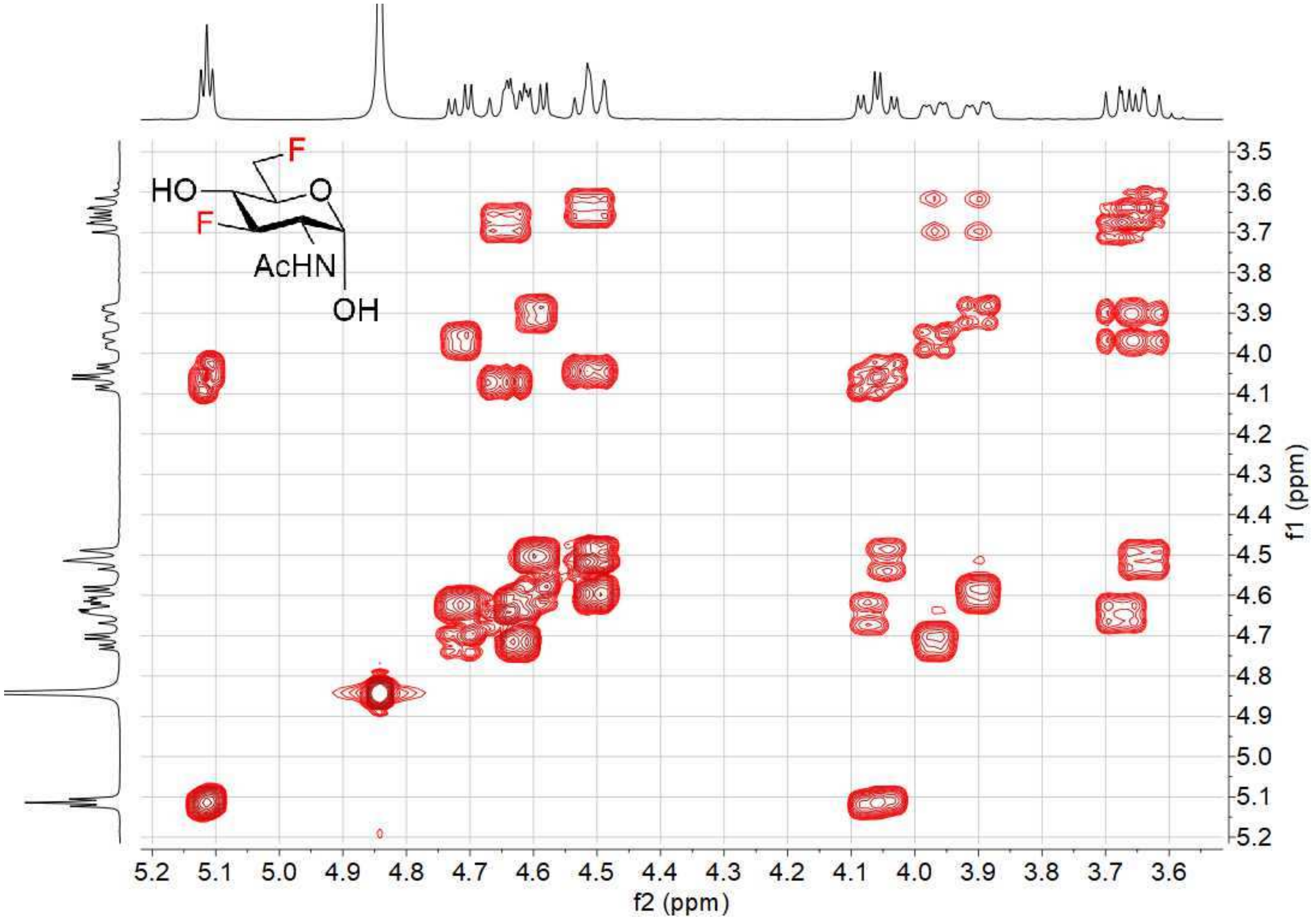
HO
F
F
O
AcHN
OH
f2 (ppm)
f1 (ppm)
5.2 5.1 5.0 4.9 4.8 4.7 4.6 4.5 4.4 4.3 4.2 4.1 4.0 3.9 3.8 3.7 3.6
3.5 3.6 3.7 3.8 3.9 4.0 4.1 4.2 4.3 4.4 4.5 4.6 4.7 4.8 4.9 5.0 5.1 5.2

^{1}H-^{13}C HSQC 62

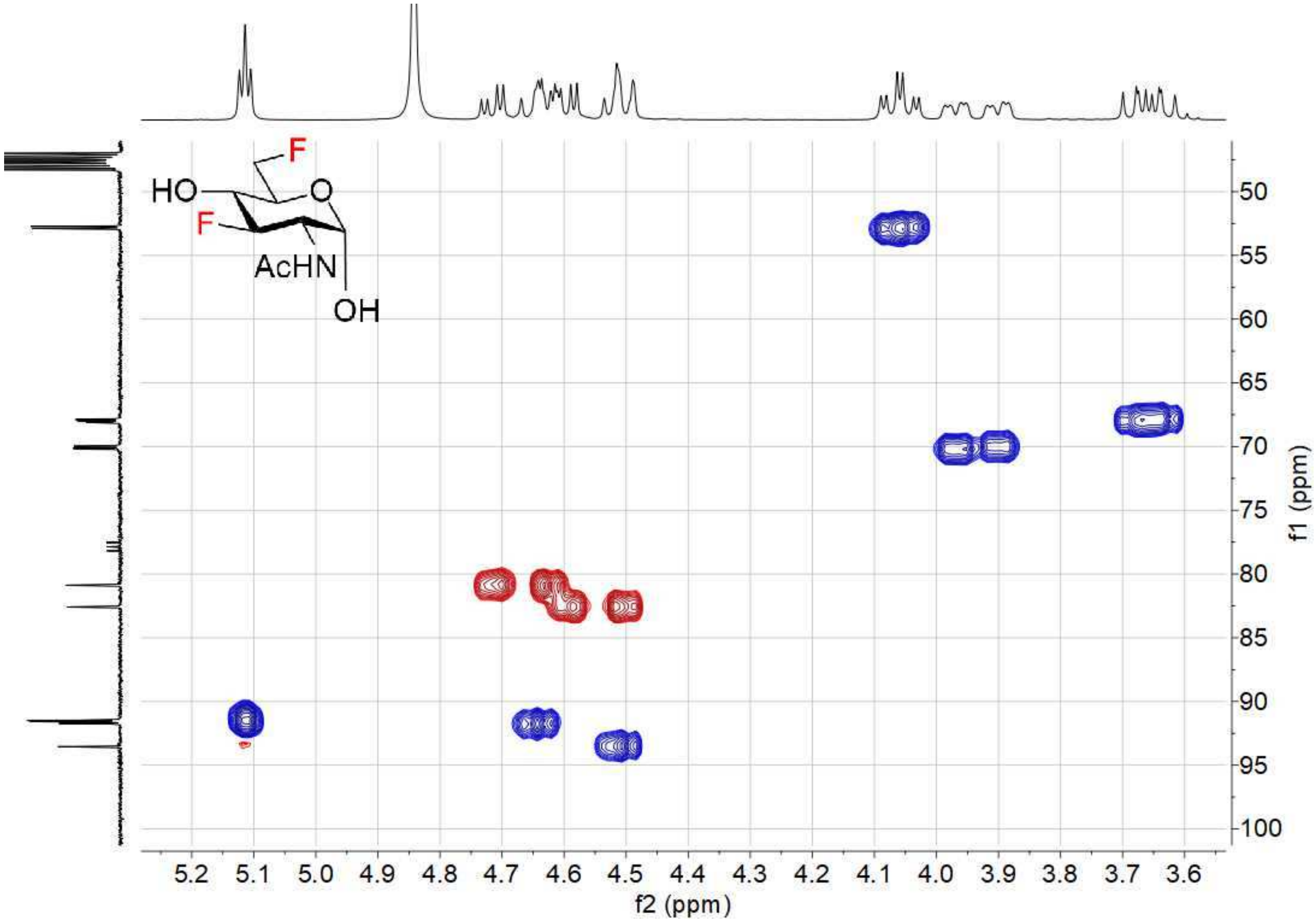

NMR COMPOUND 63

^{1}H NMR (400 MHz, CD_3OD) 63 (α/β ca. 10/3)

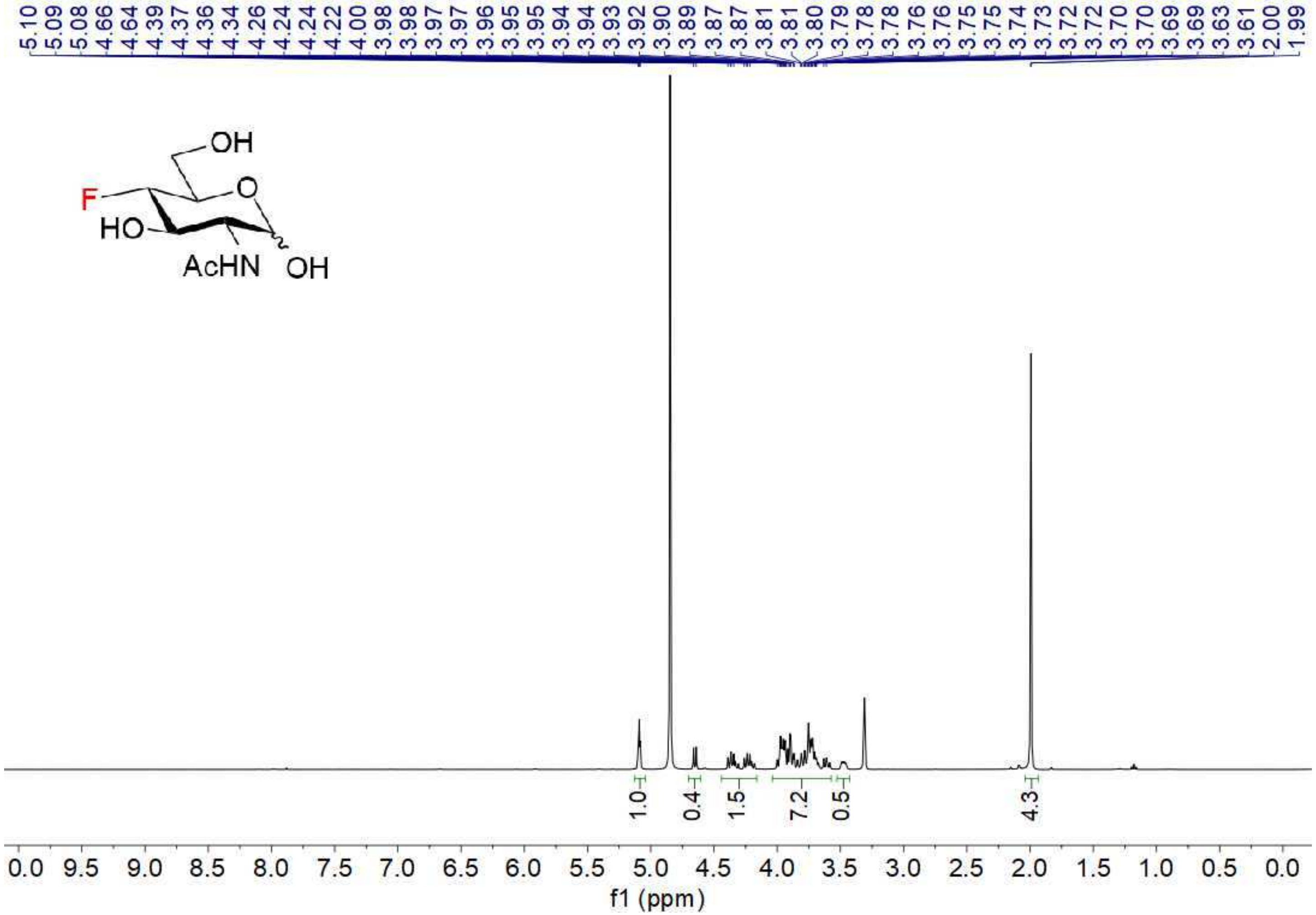

^{13}C NMR (100 MHz, CD_3OD) 63

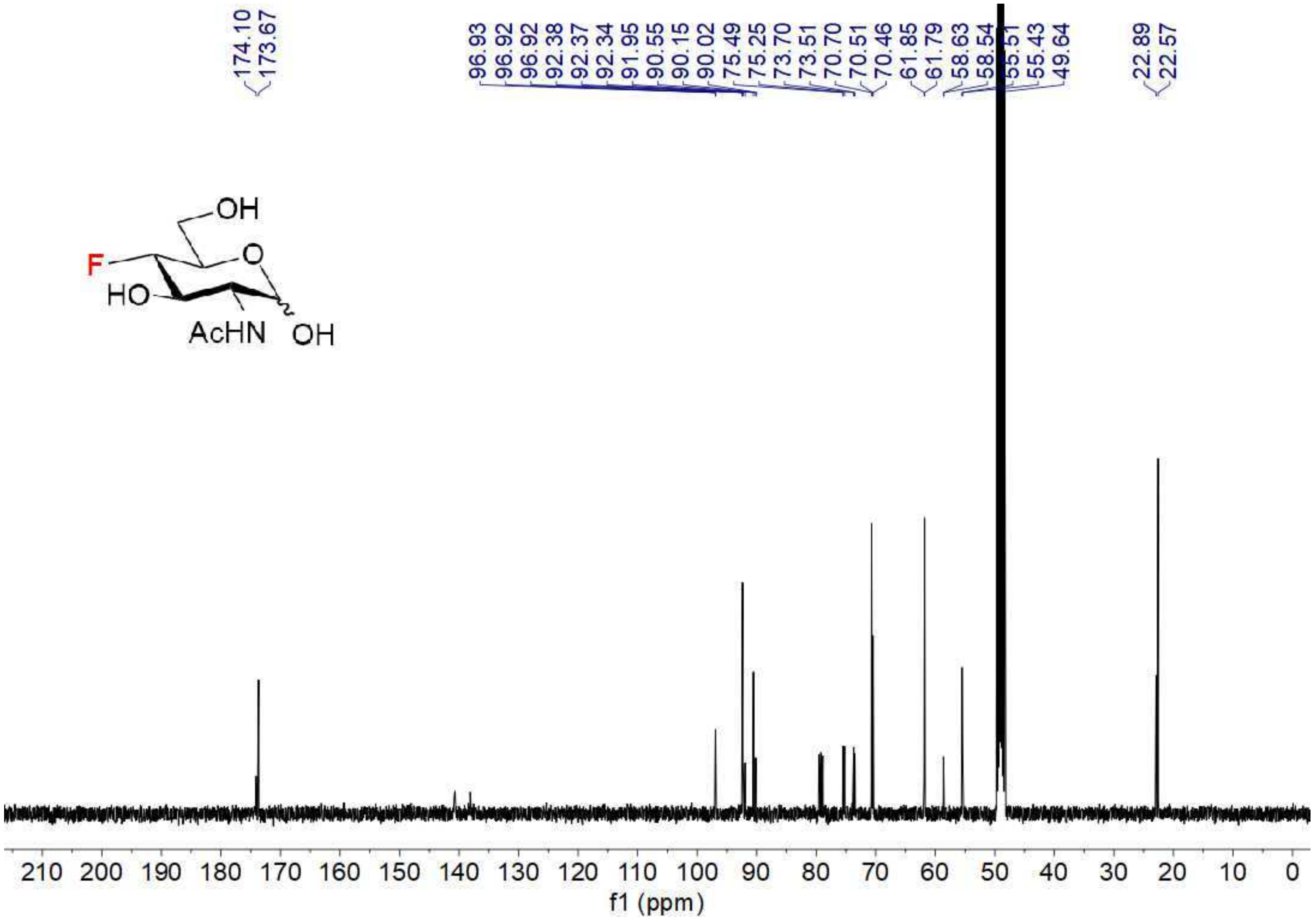

^{19}F NMR (376 MHz, CD_3OD) 63 (α/β ca. 10/3)

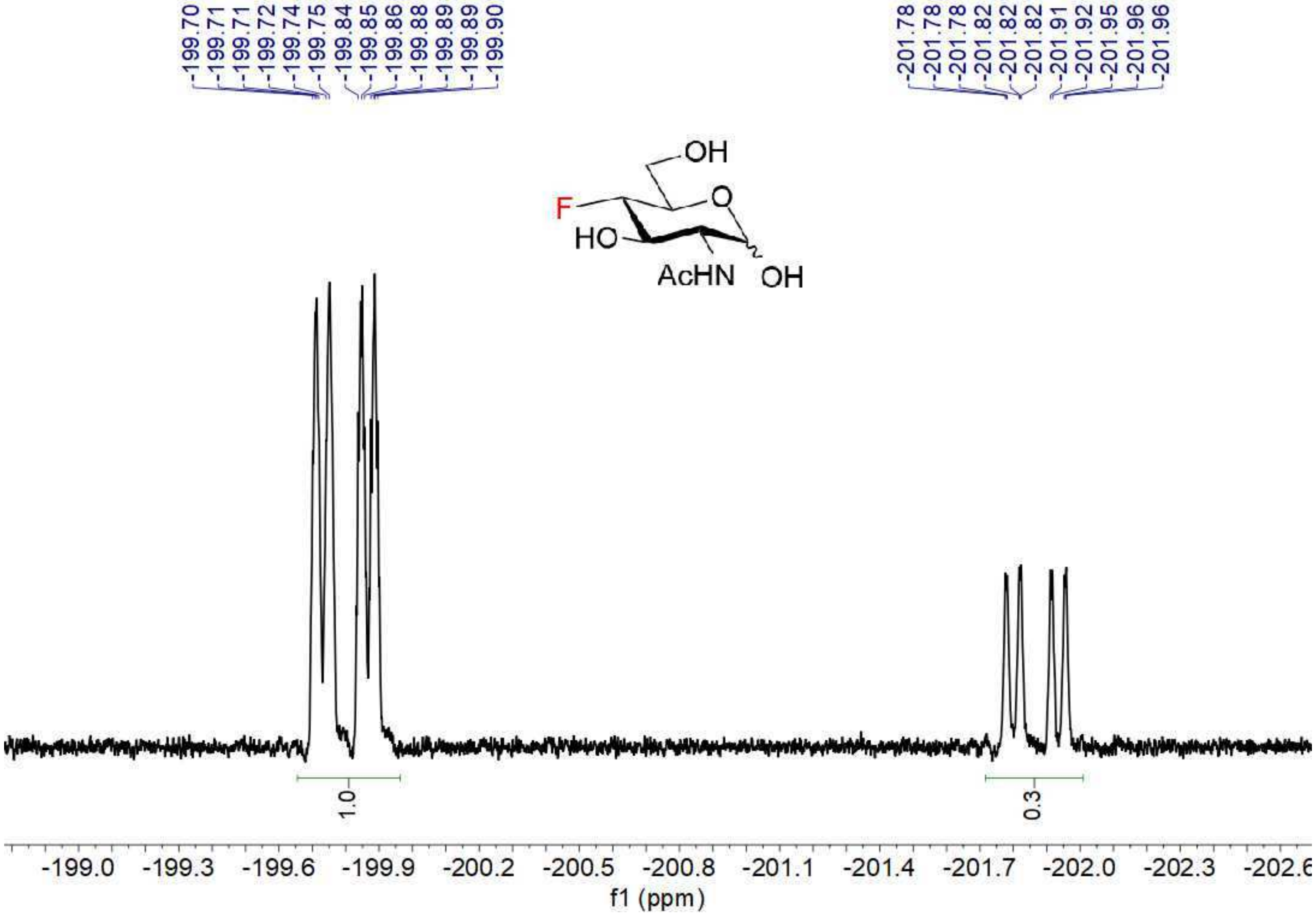

^{1}H-^{1}H COSY 63

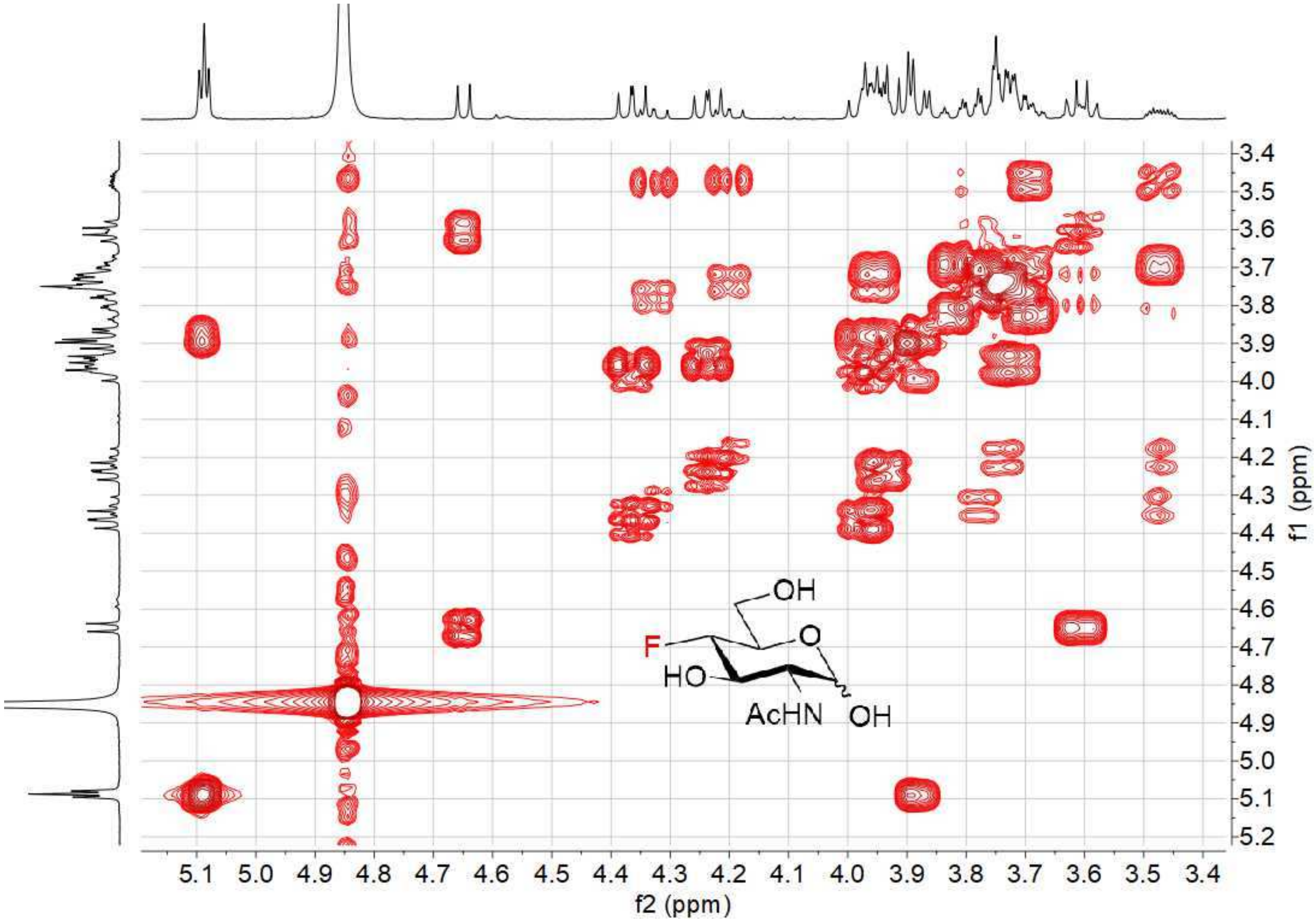

^{1}H-^{13}C HSQC 63

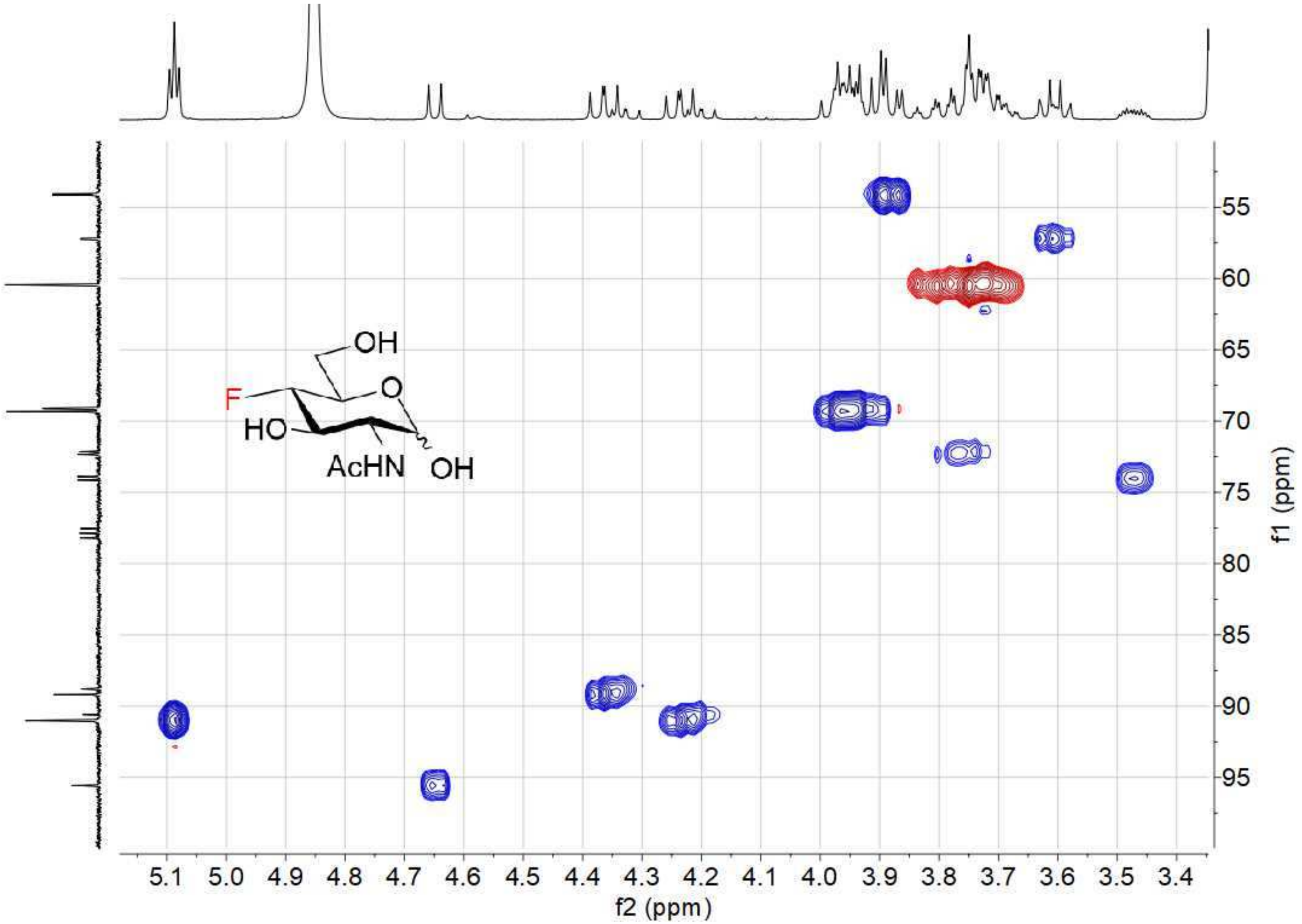

NMR COMPOUND 64

^{1}H NMR (400 MHz, CD_3OD) 64

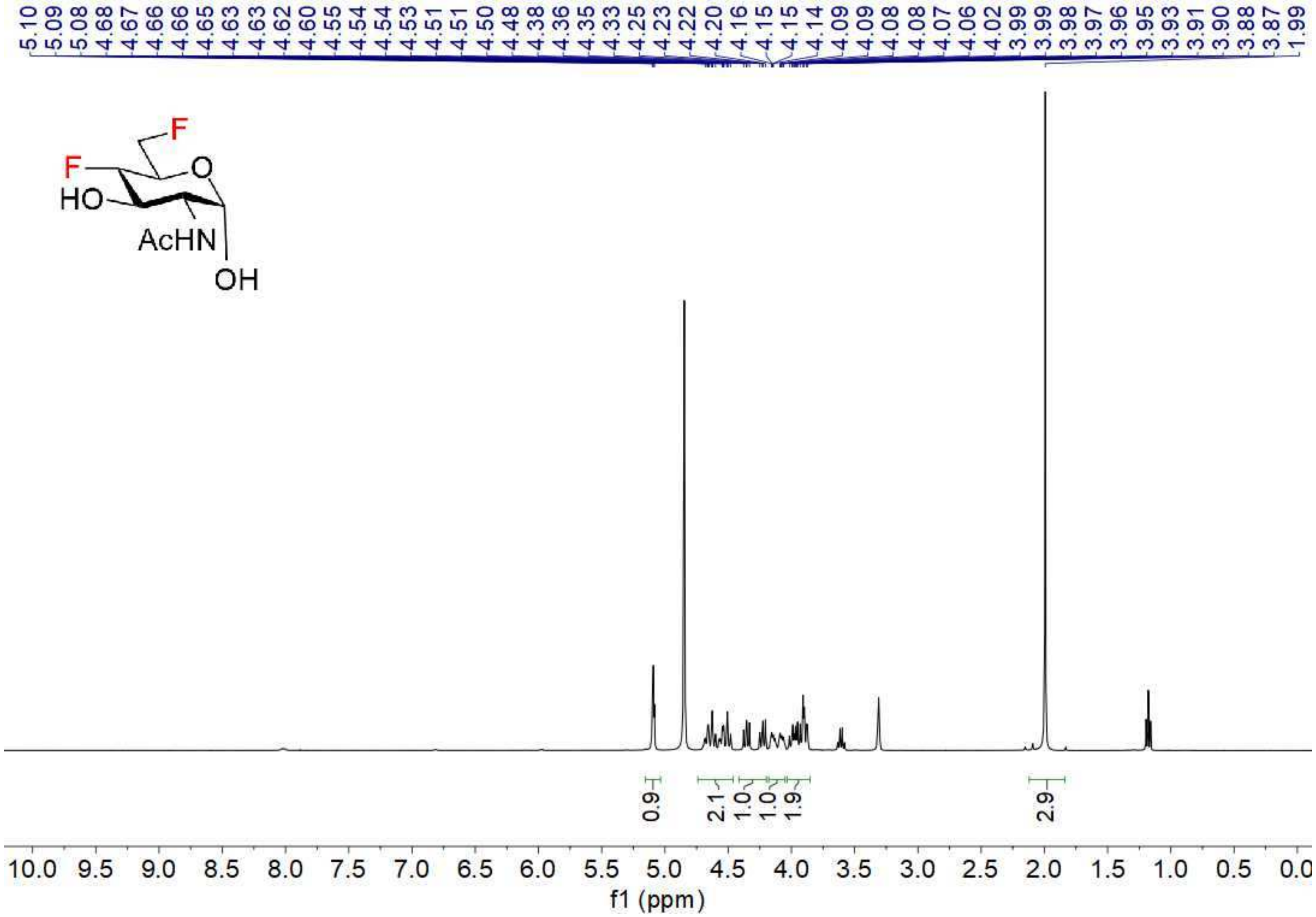

^{13}C NMR (100 MHz, CD_3OD) 64

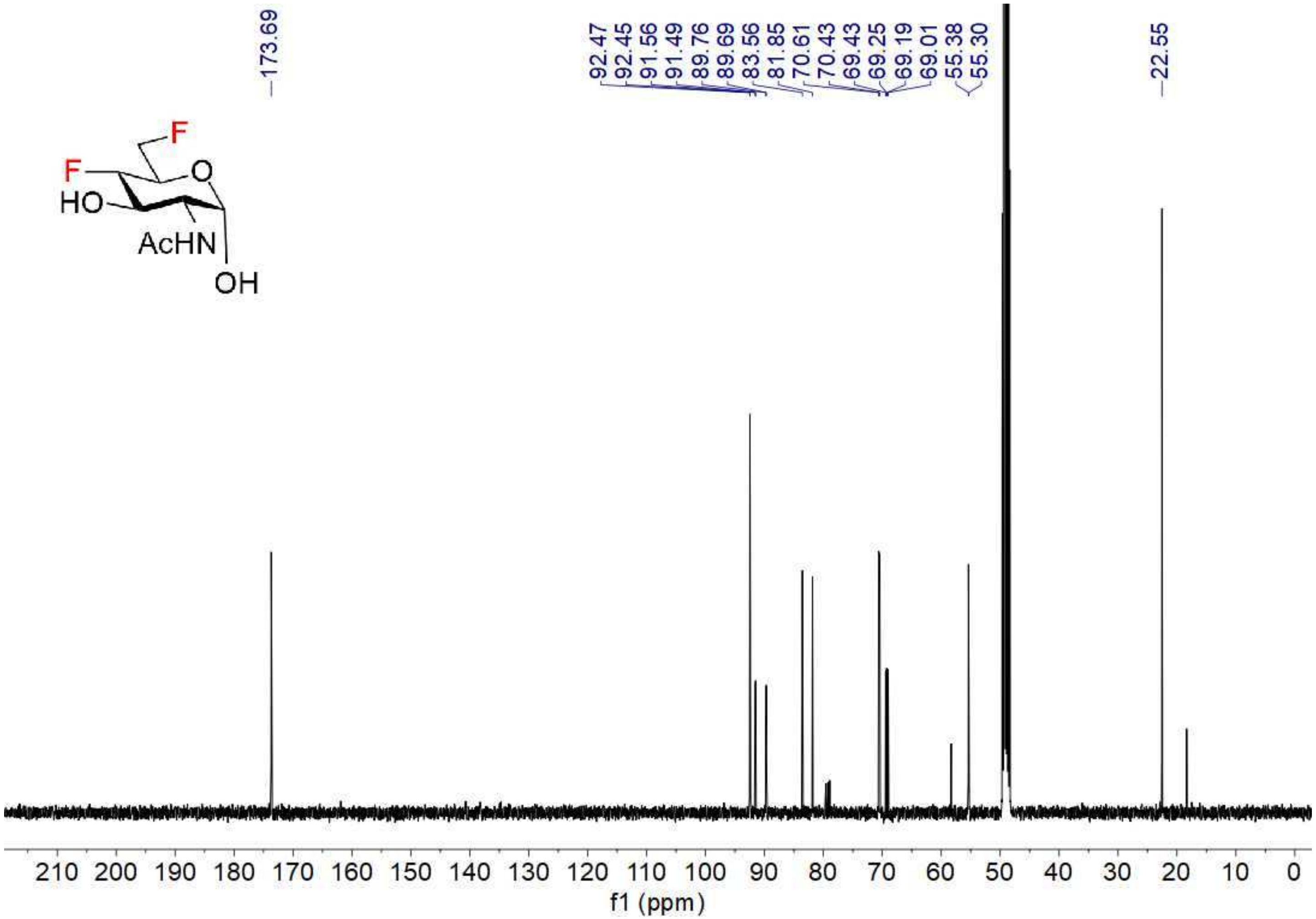

^{19}F NMR (376 MHz, CD_3OD) 64

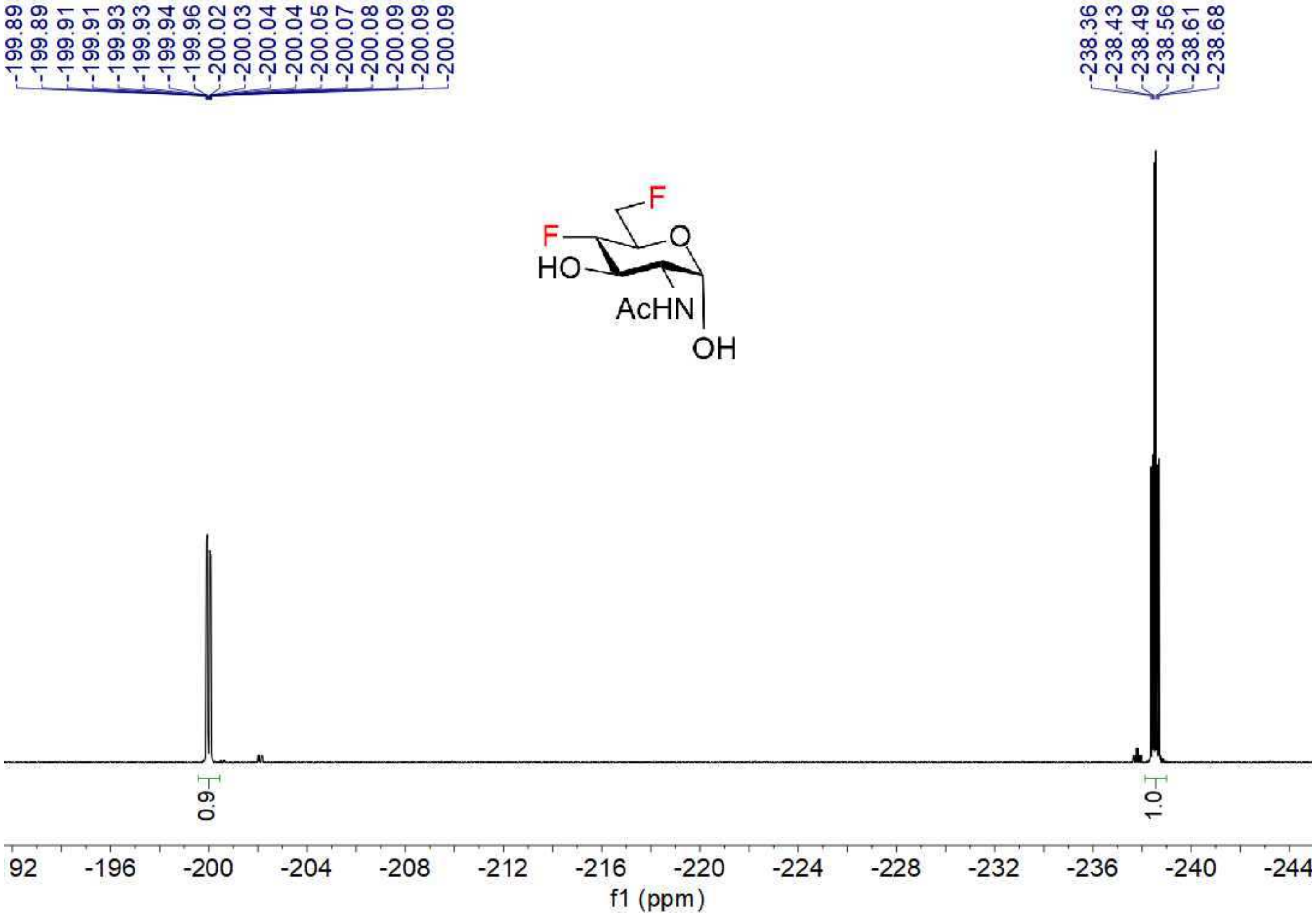

^{1}H-^{1}H COSY 64

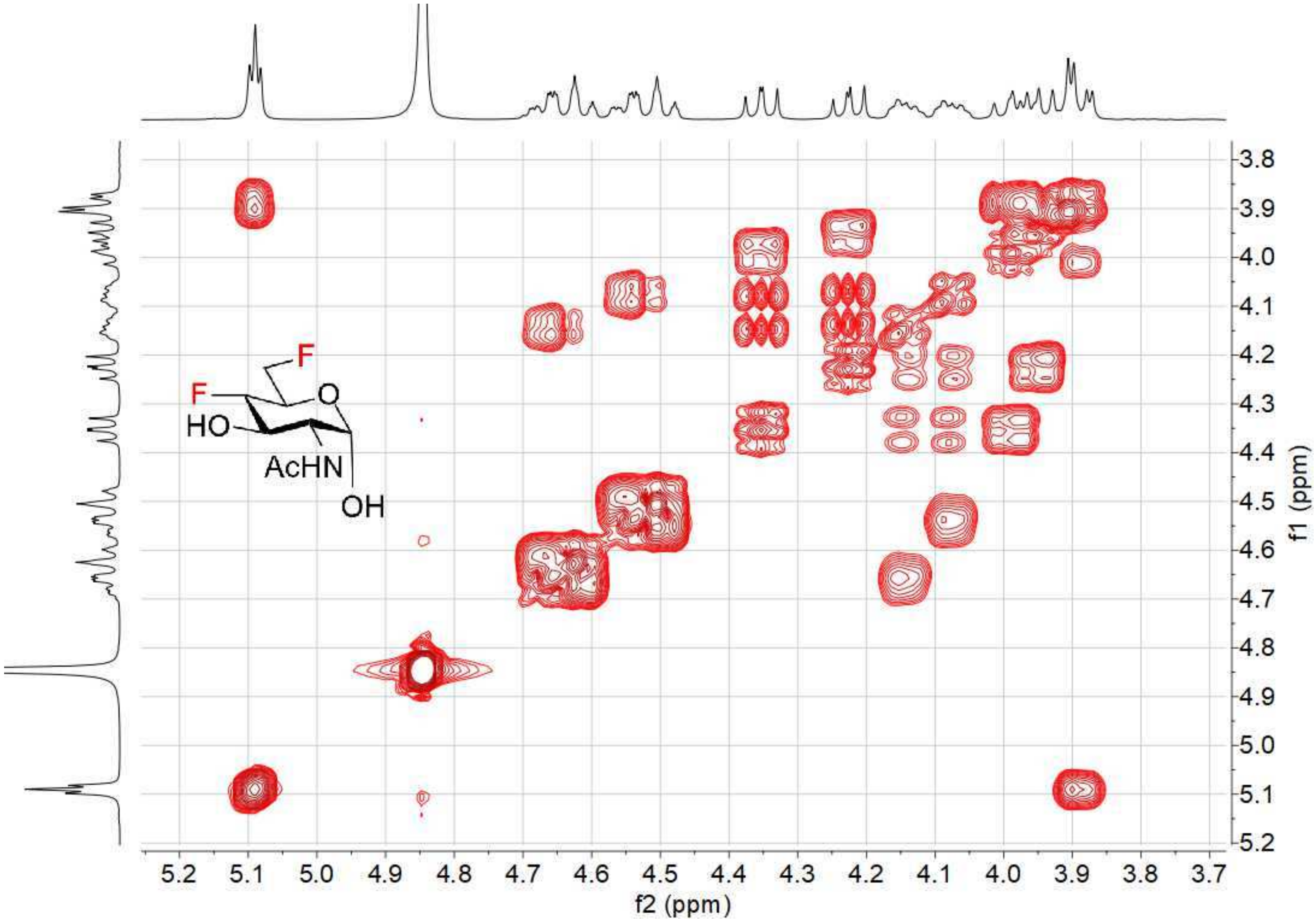
F
F
O
HO
AcHN
OH
5.2
5.1
5.0
4.9
4.8
4.7
4.6
4.5
4.4
4.3
4.2
4.1
4.0
3.9
3.8
3.7
f2 (ppm)
f1 (ppm)

^{1}H-^{13}C HSQC 64

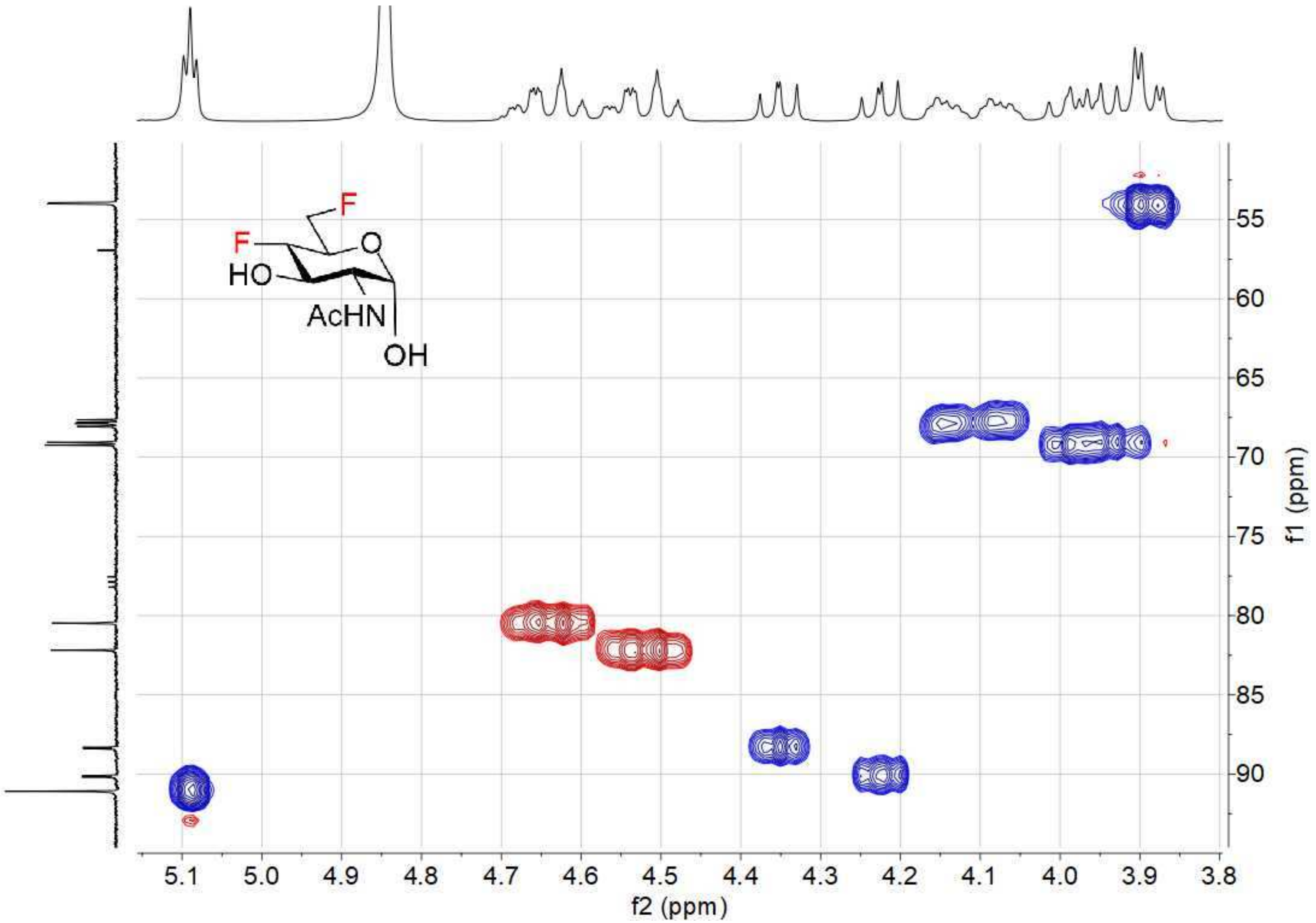

NMR COMPOUND 65

^{1}H NMR (400 MHz, CD_3OD) 65 (α/β ca. 10/1)

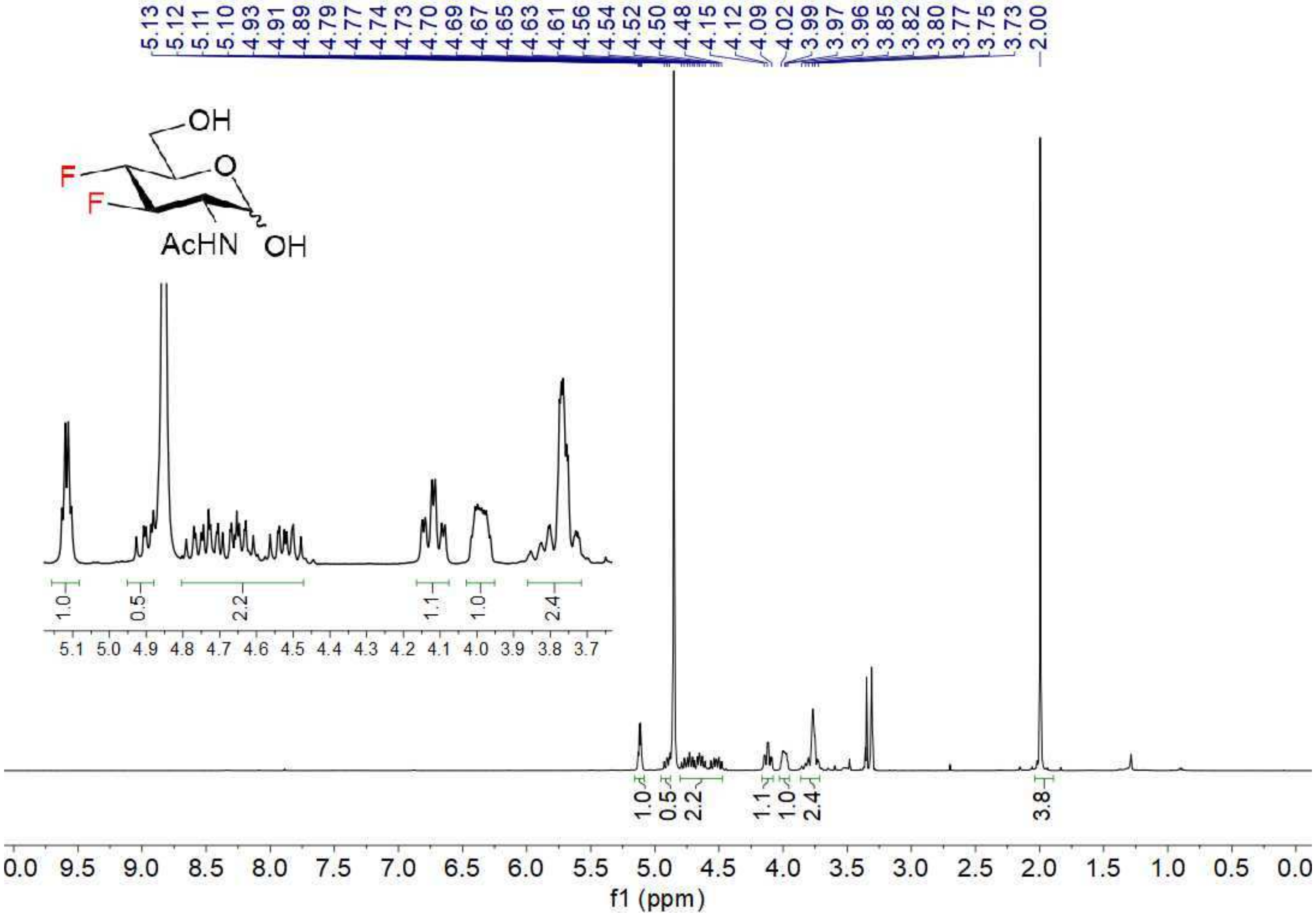

^{13}C NMR (100 MHz, CD_3OD) 65

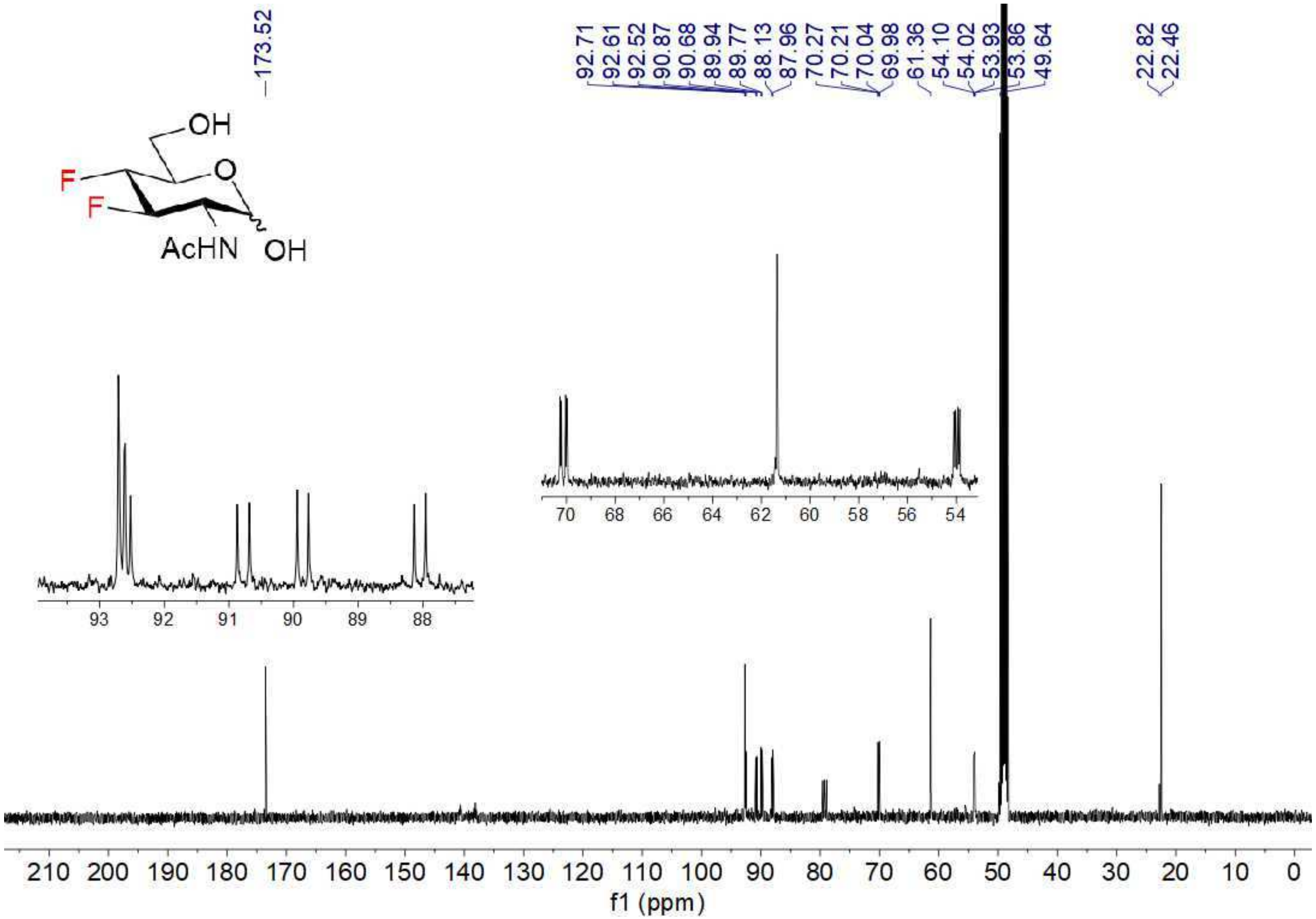

^{19}F NMR (376 MHz, CD_3OD) 65 (α/β ca. 10/1)

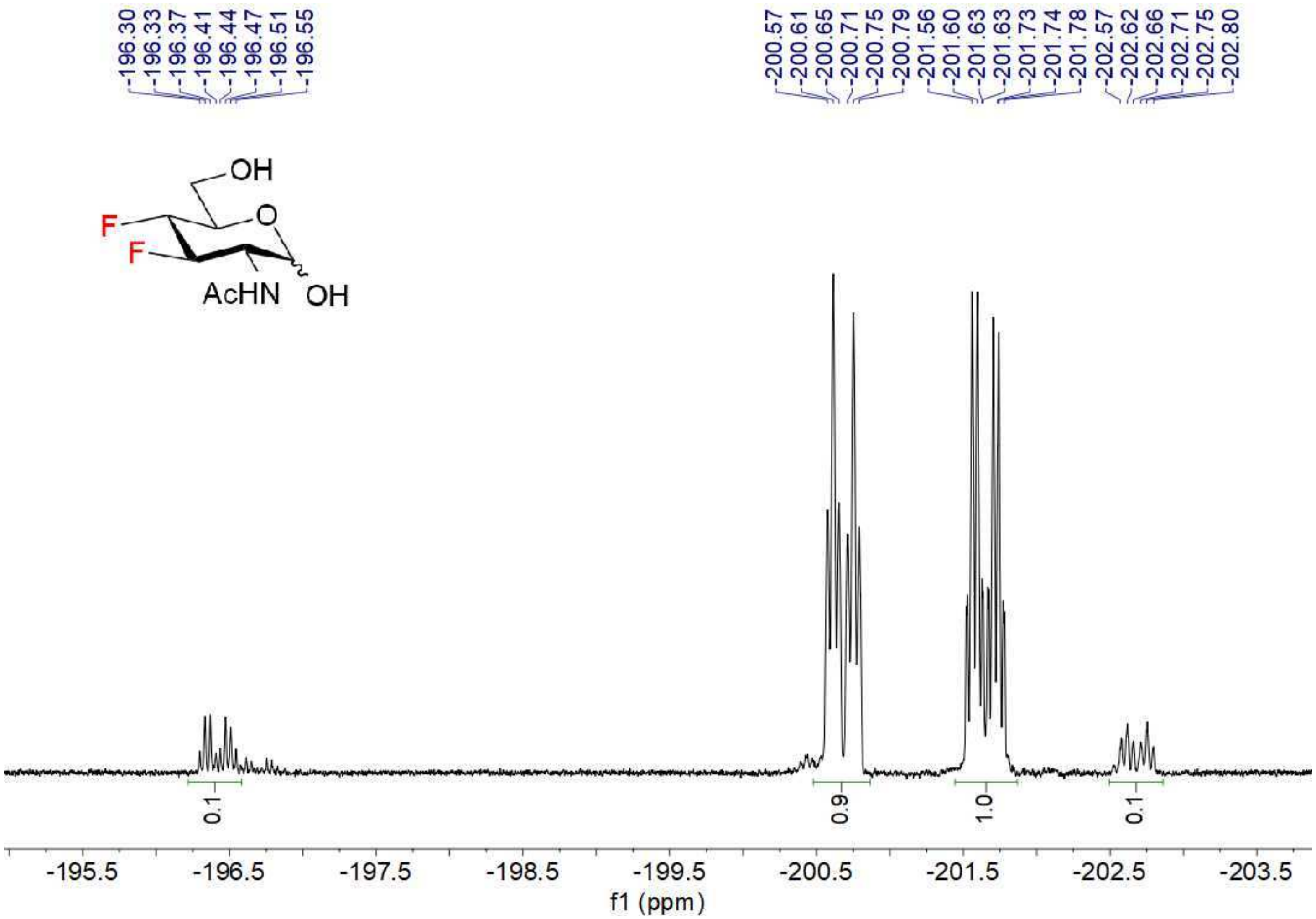

^{1}H-^{1}H COSY 65

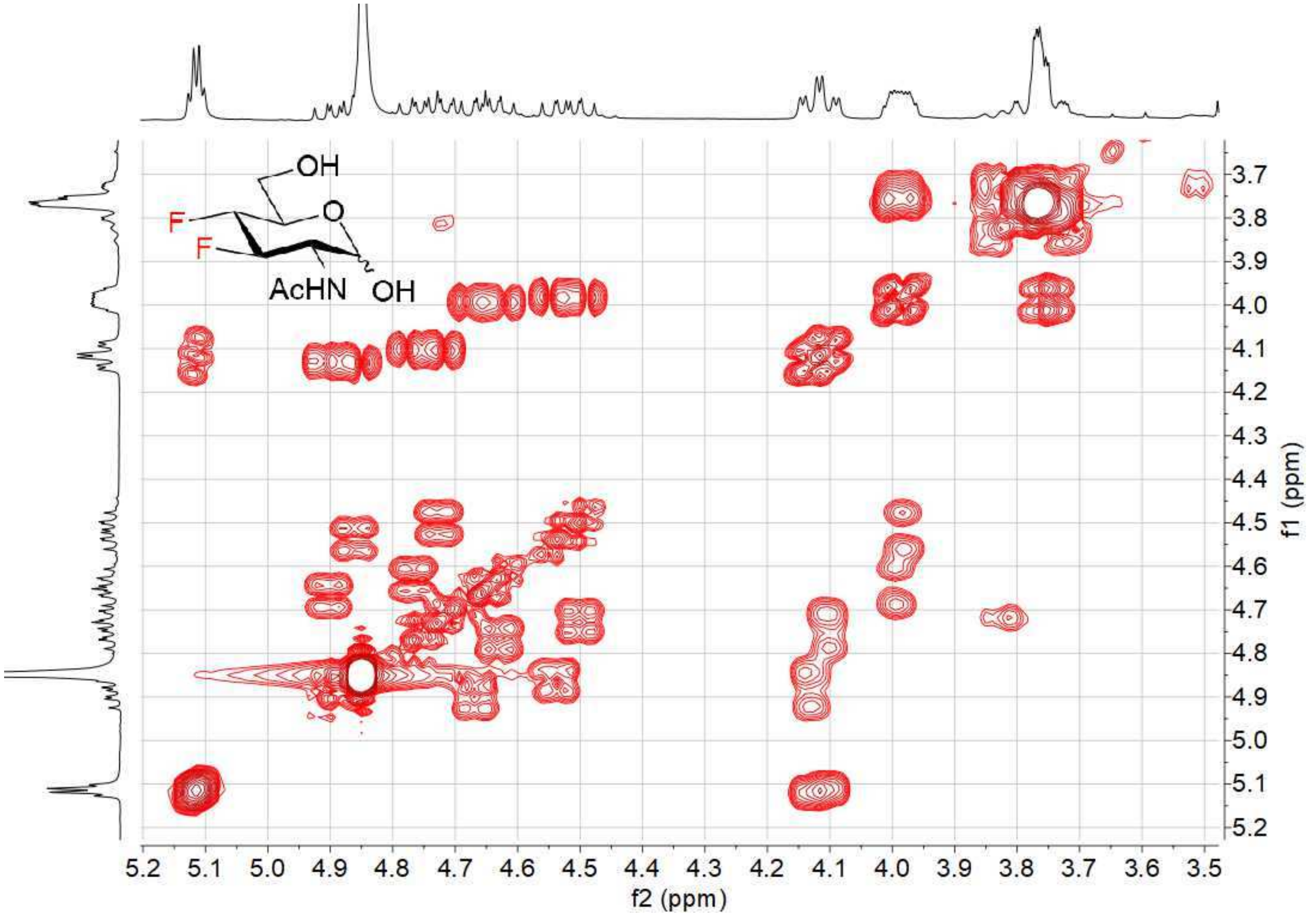
OH
O
F
F
AcHN
OH
f2 (ppm)
f1 (ppm)
5.2 5.1 5.0 4.9 4.8 4.7 4.6 4.5 4.4 4.3 4.2 4.1 4.0 3.9 3.8 3.7 3.6 3.5
3.7 3.8 3.9 4.0 4.1 4.2 4.3 4.4 4.5 4.6 4.7 4.8 4.9 5.0 5.1 5.2

^{1}H-^{13}C HSQC 65

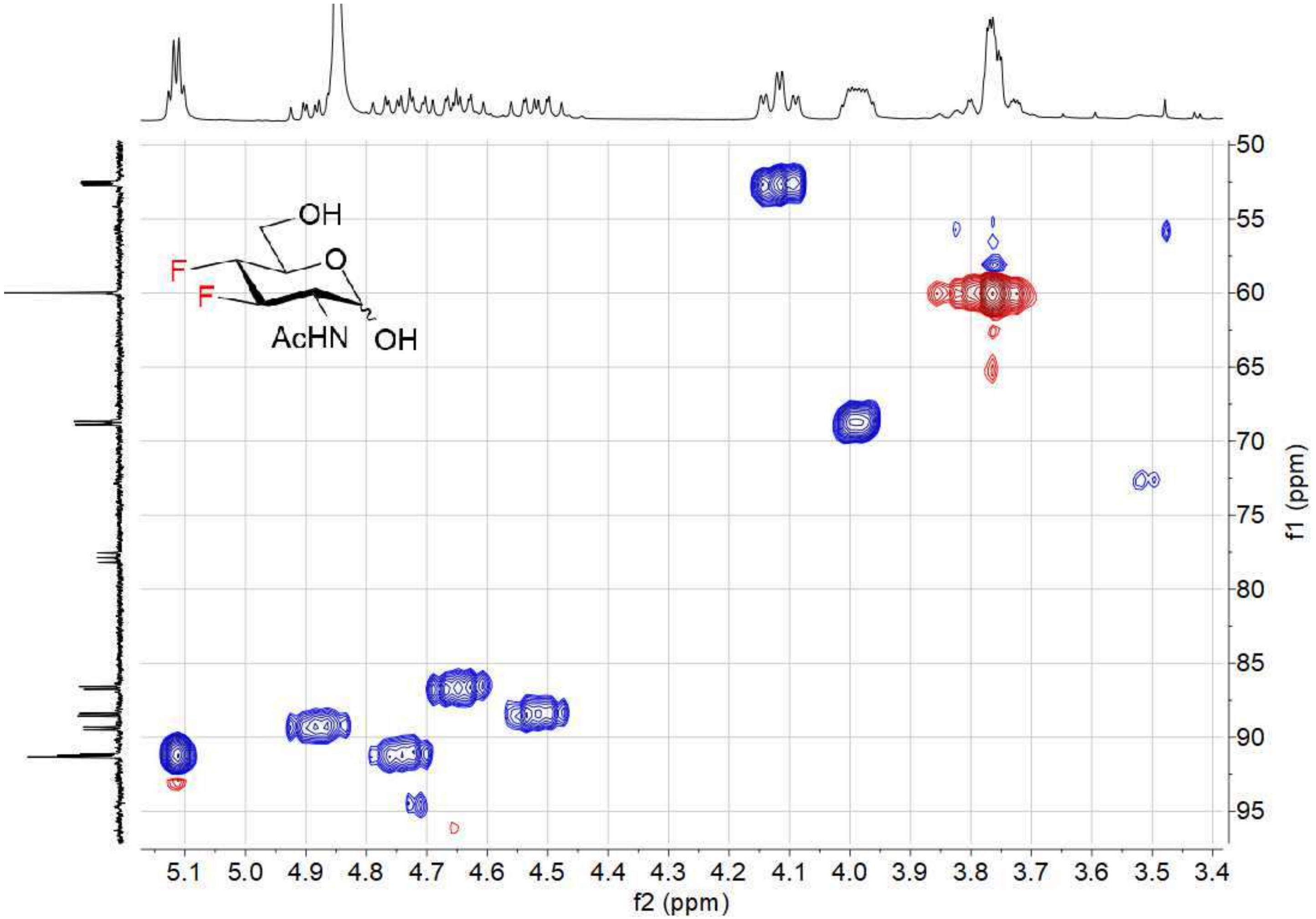

NMR COMPOUND 66

^{1}H NMR (400 MHz, CD_3OD) 66 (α/β ca. 10/3)

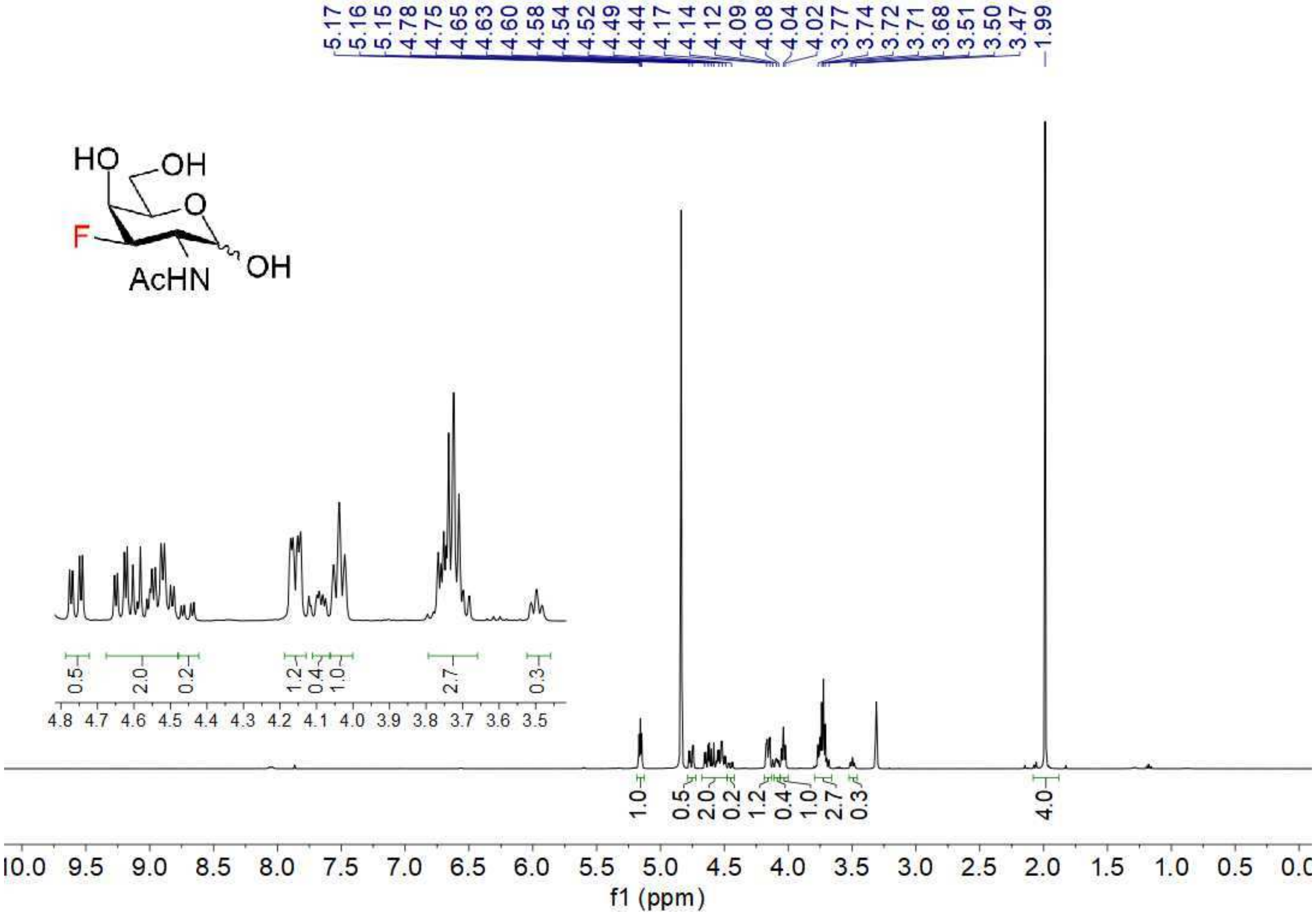

^{13}C NMR (100 MHz, CD_3OD) 66

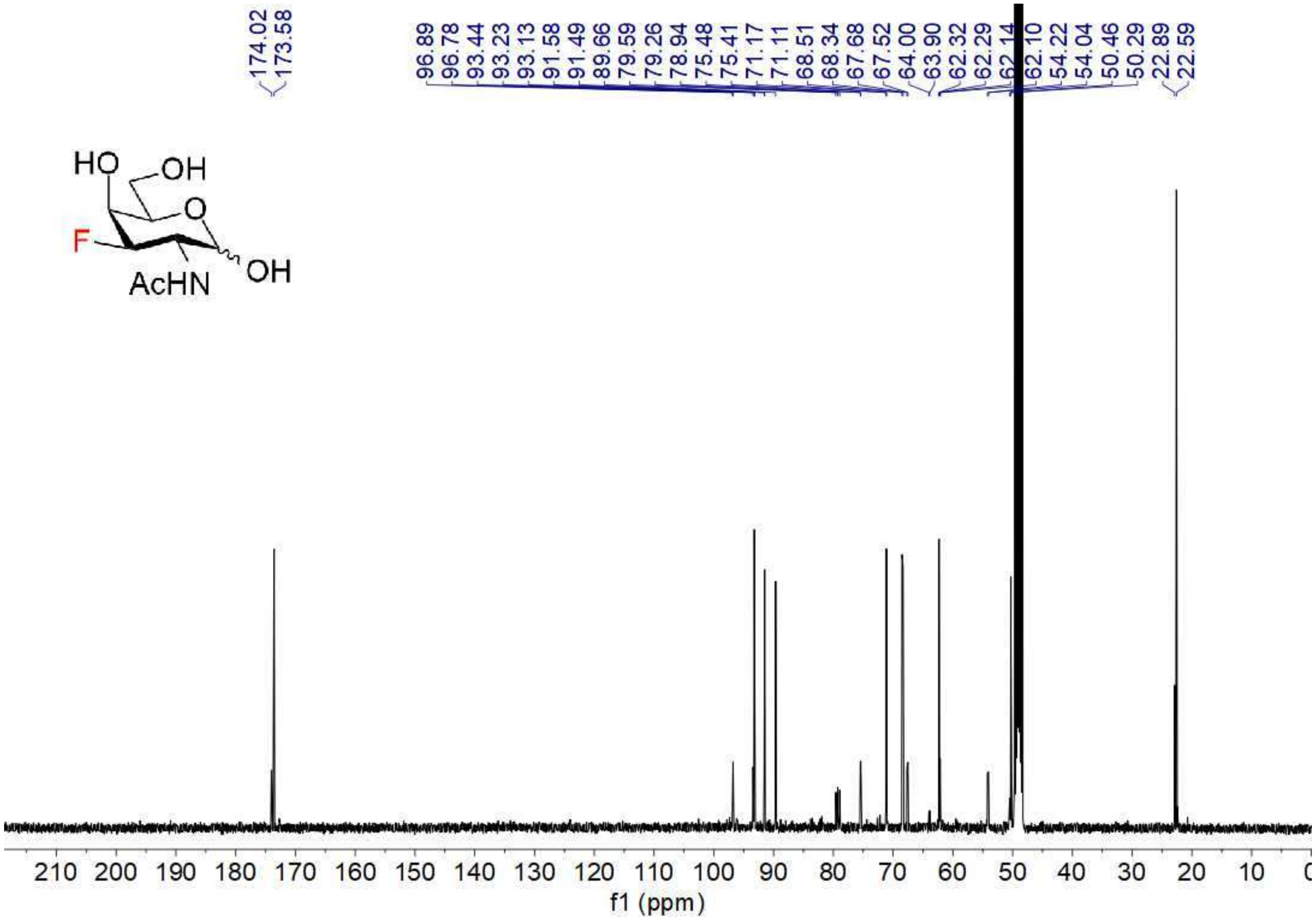

^{19}F NMR (376 MHz, CD_3OD) 66 (α/β ca. 10/3)

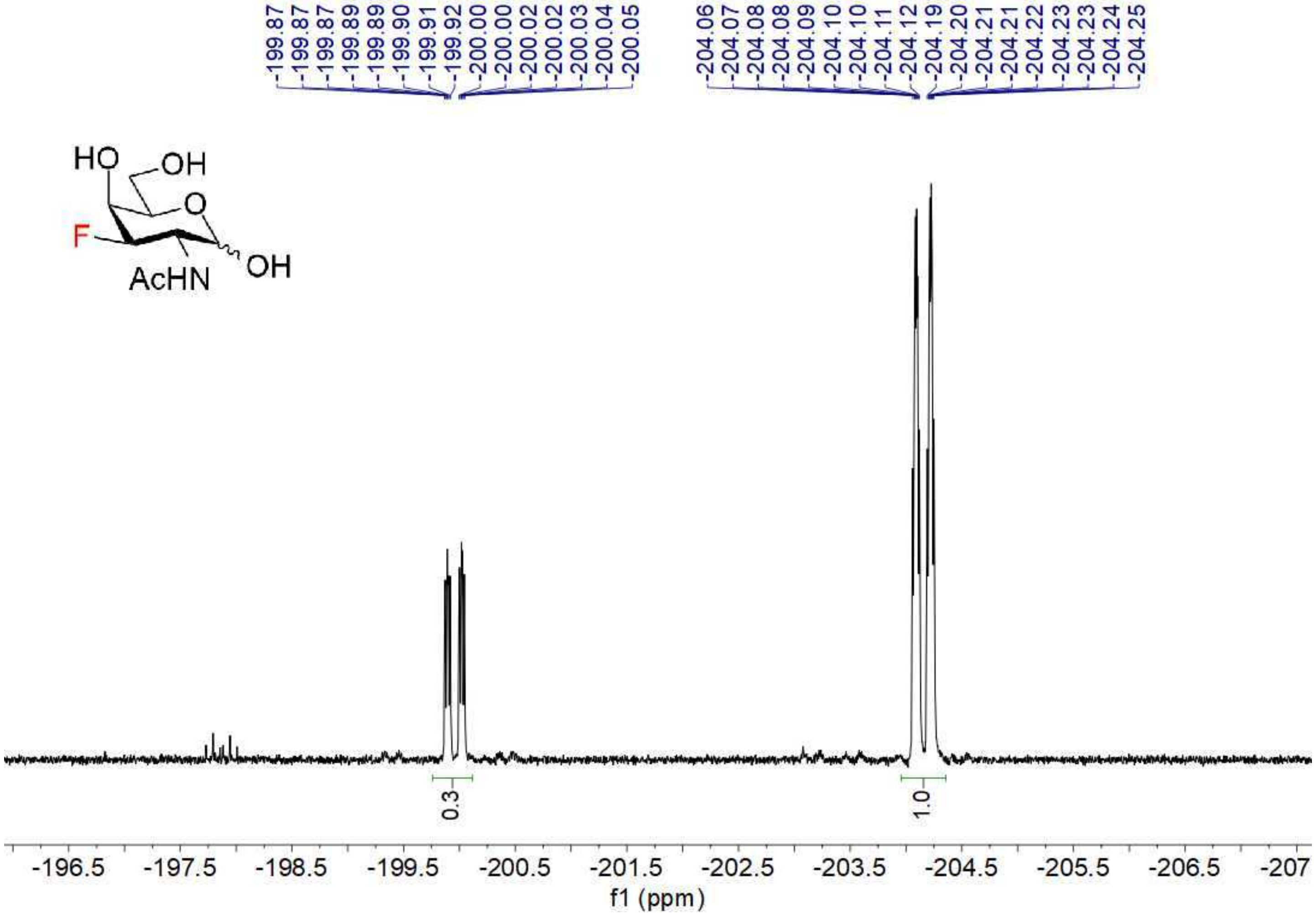

^{1}H-^{1}H COSY NMR 66

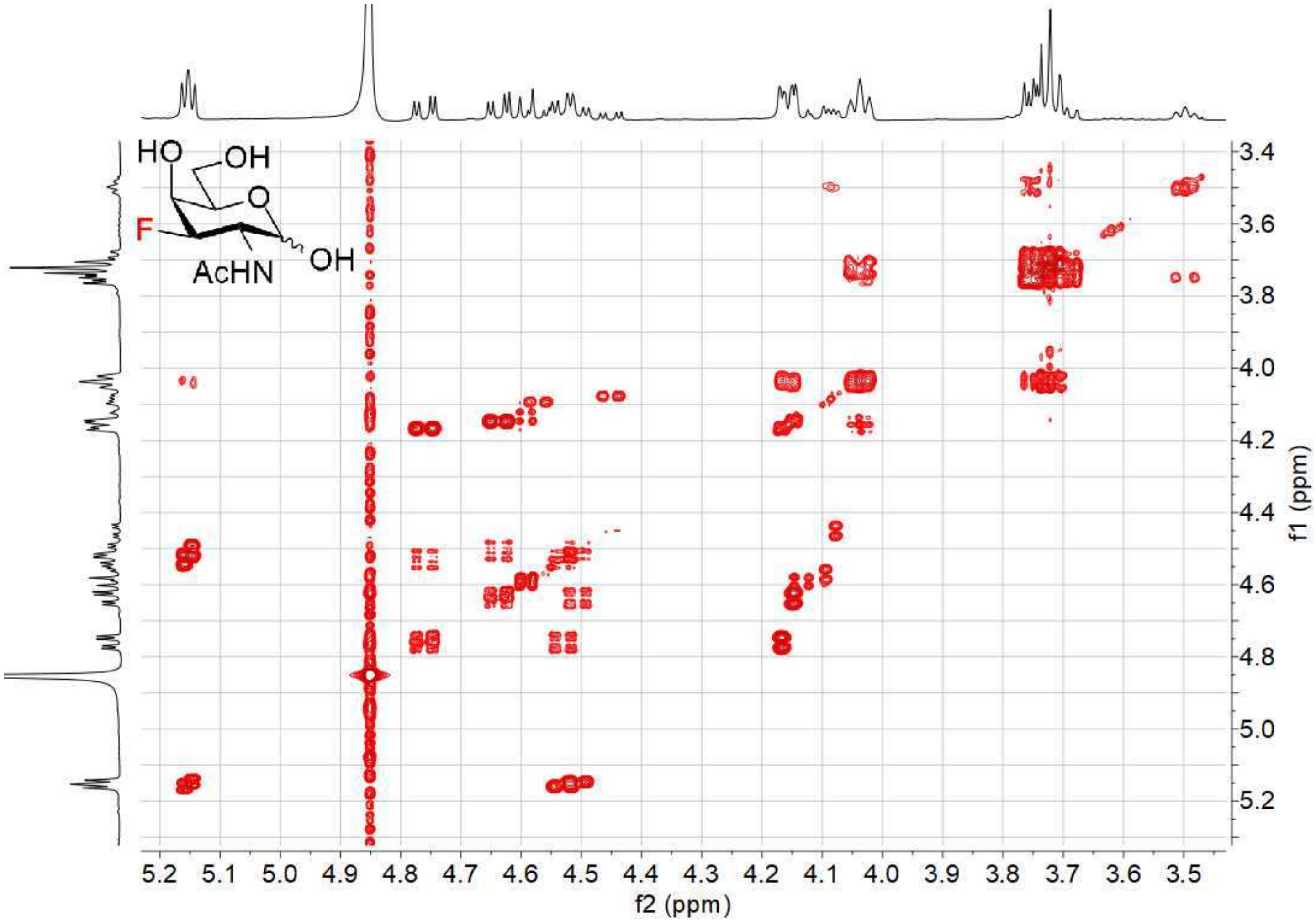

^{1}H-^{13}C HSQC NMR 66

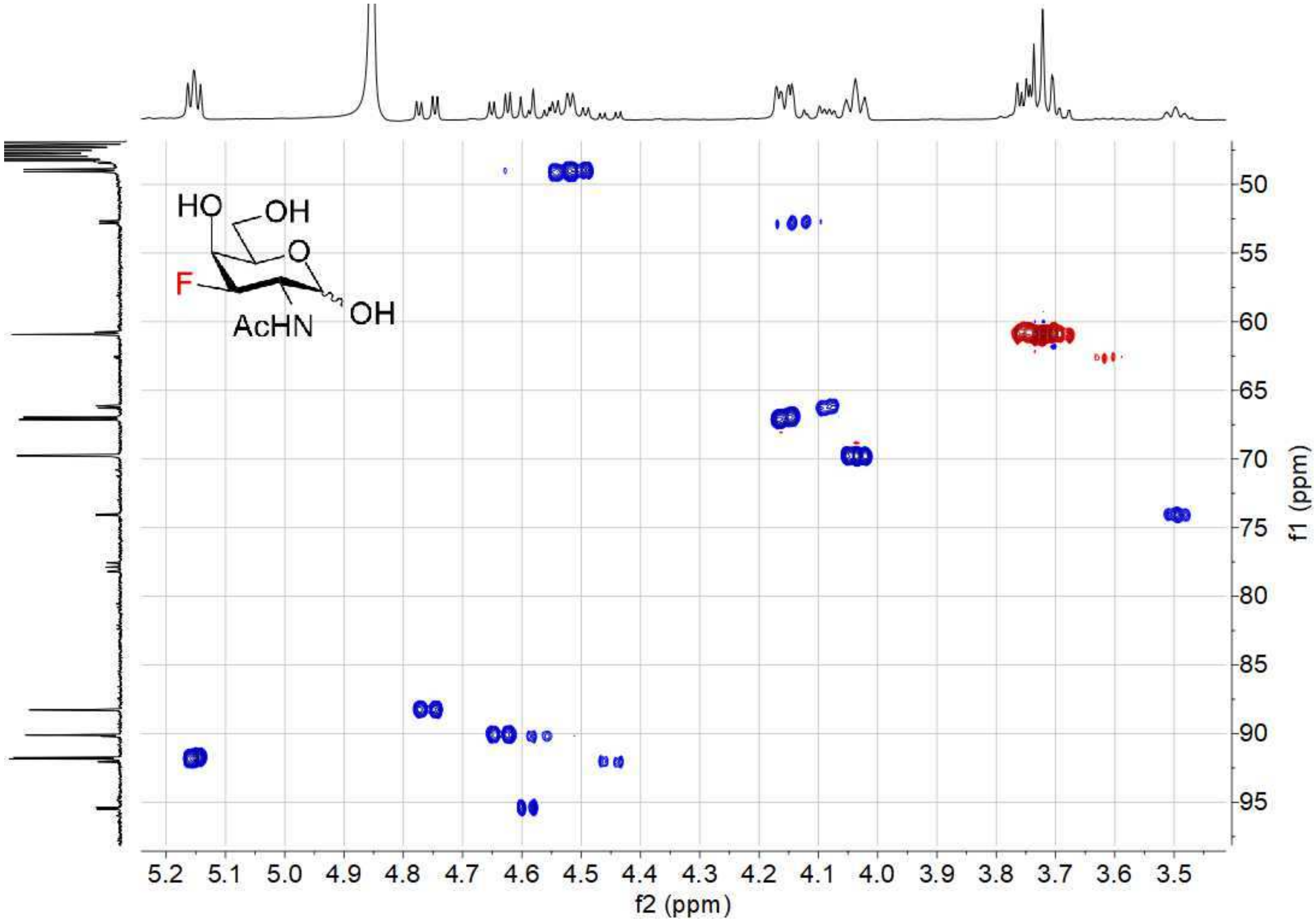

NMR COMPOUND 67

^{1}H NMR (400 MHz, CD_3OD) 67

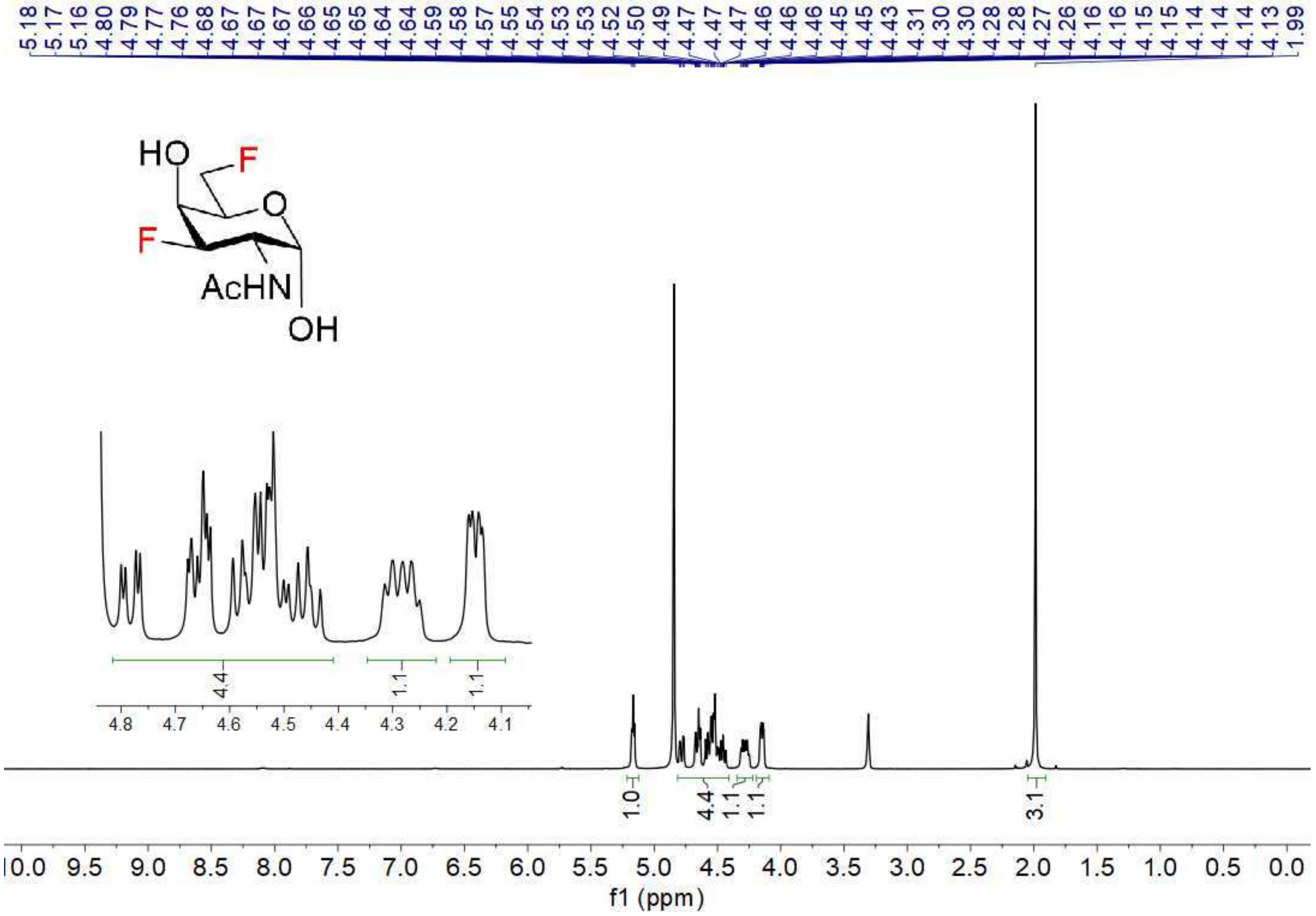

^{13}C NMR (100 MHz, CD_3OD) 67

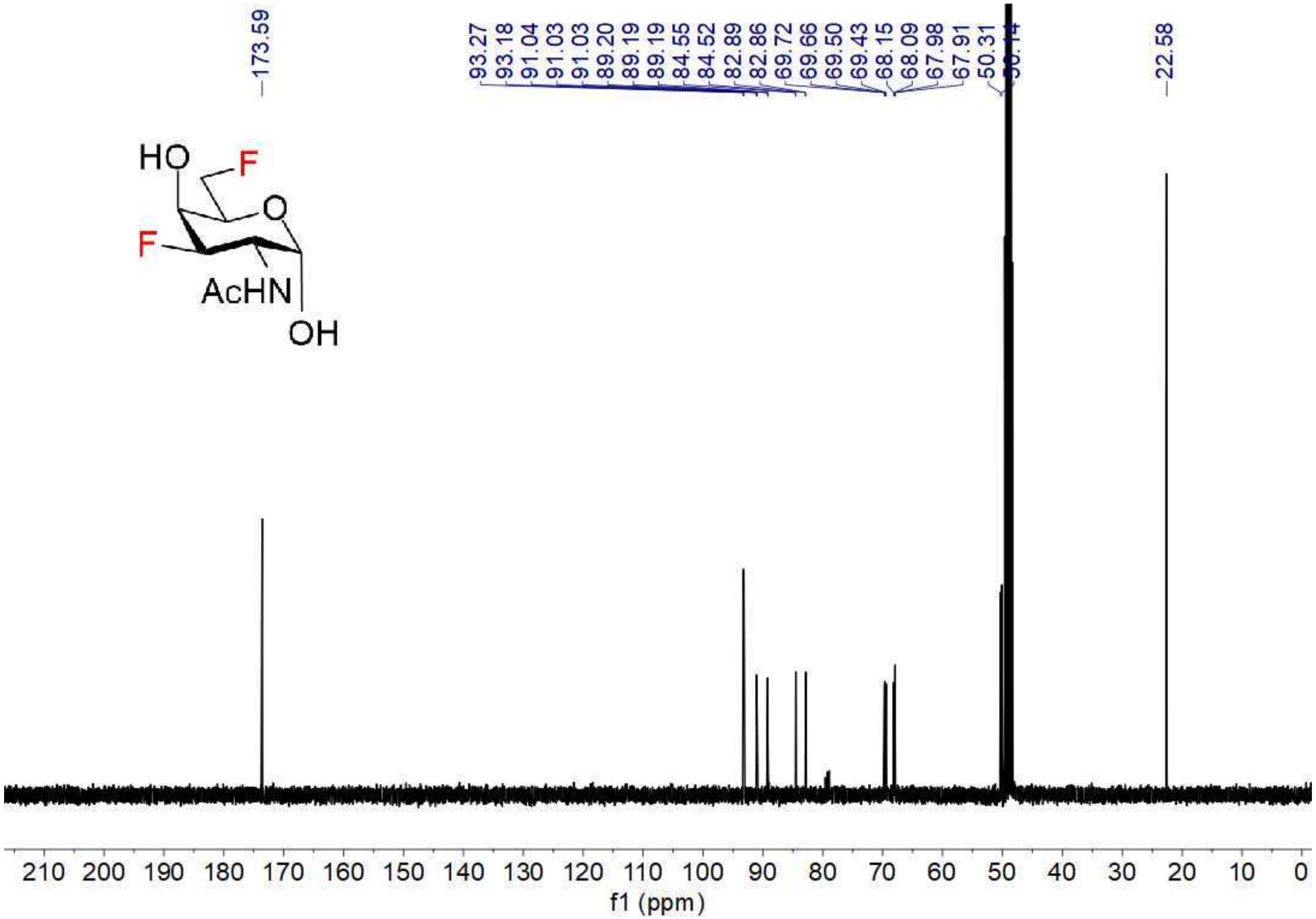

^{19}F NMR (376 MHz, CD_3OD) 67

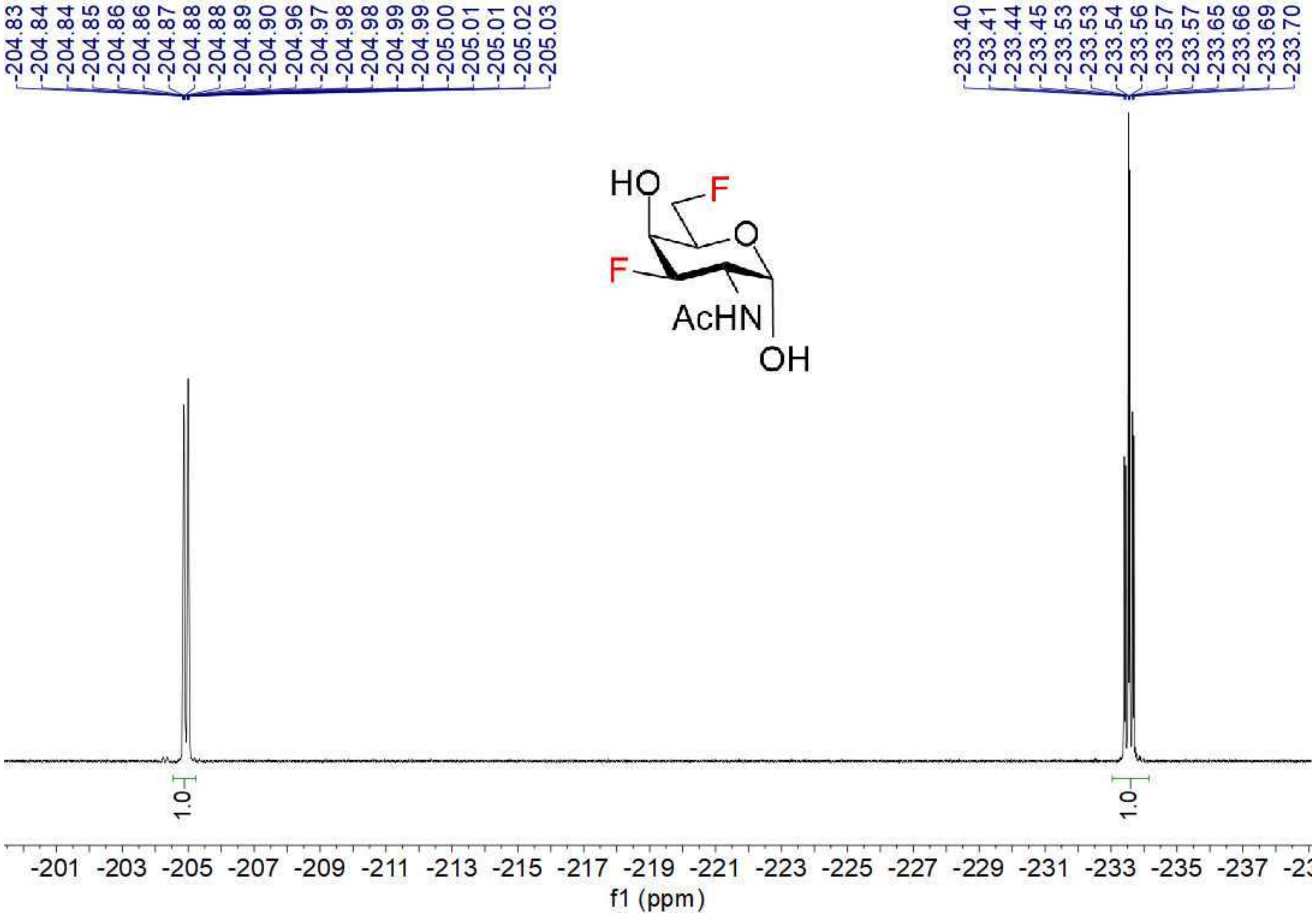

^{1}H-^{1}H COSY 67

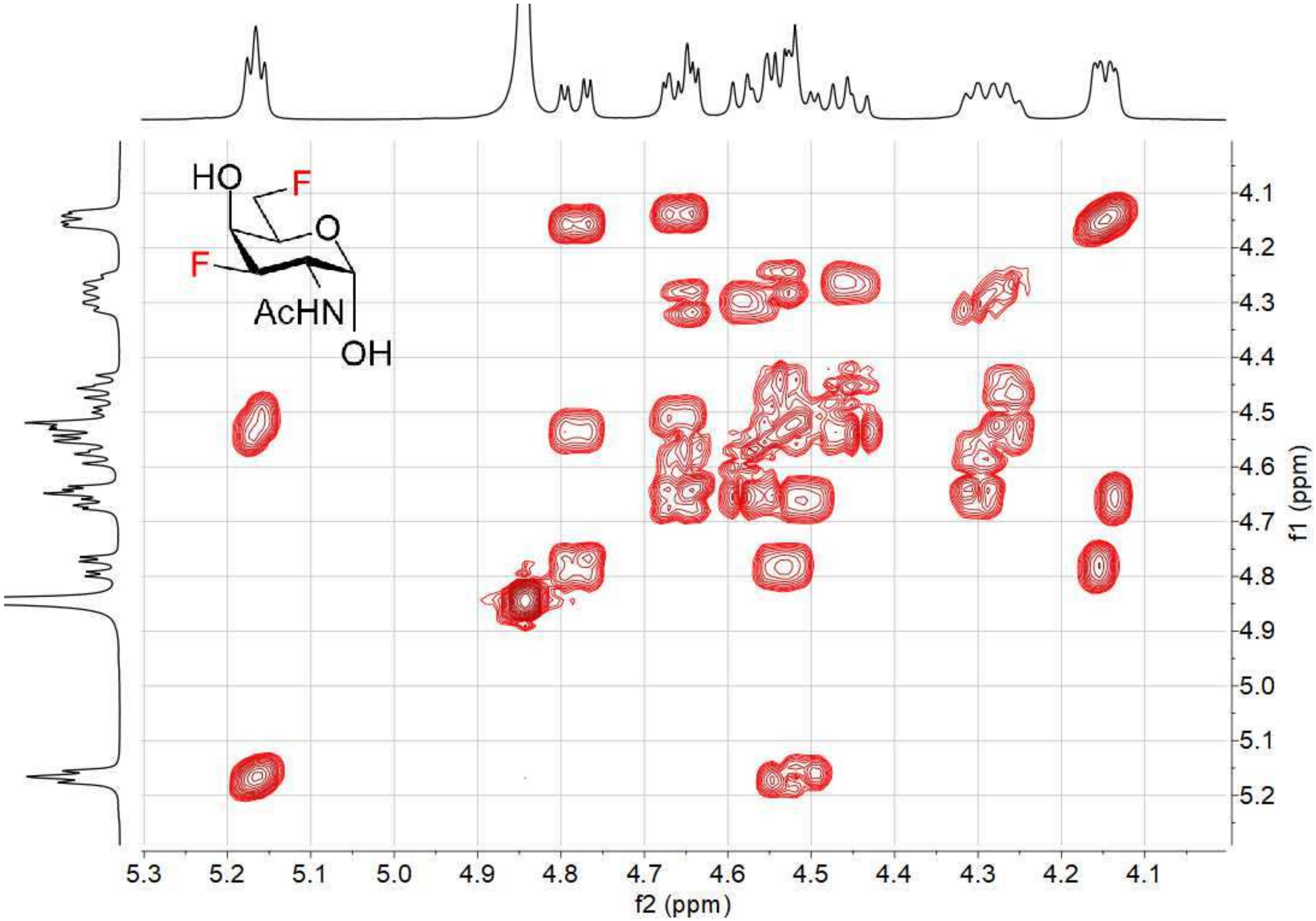

^{1}H-^{13}C HSQC 67

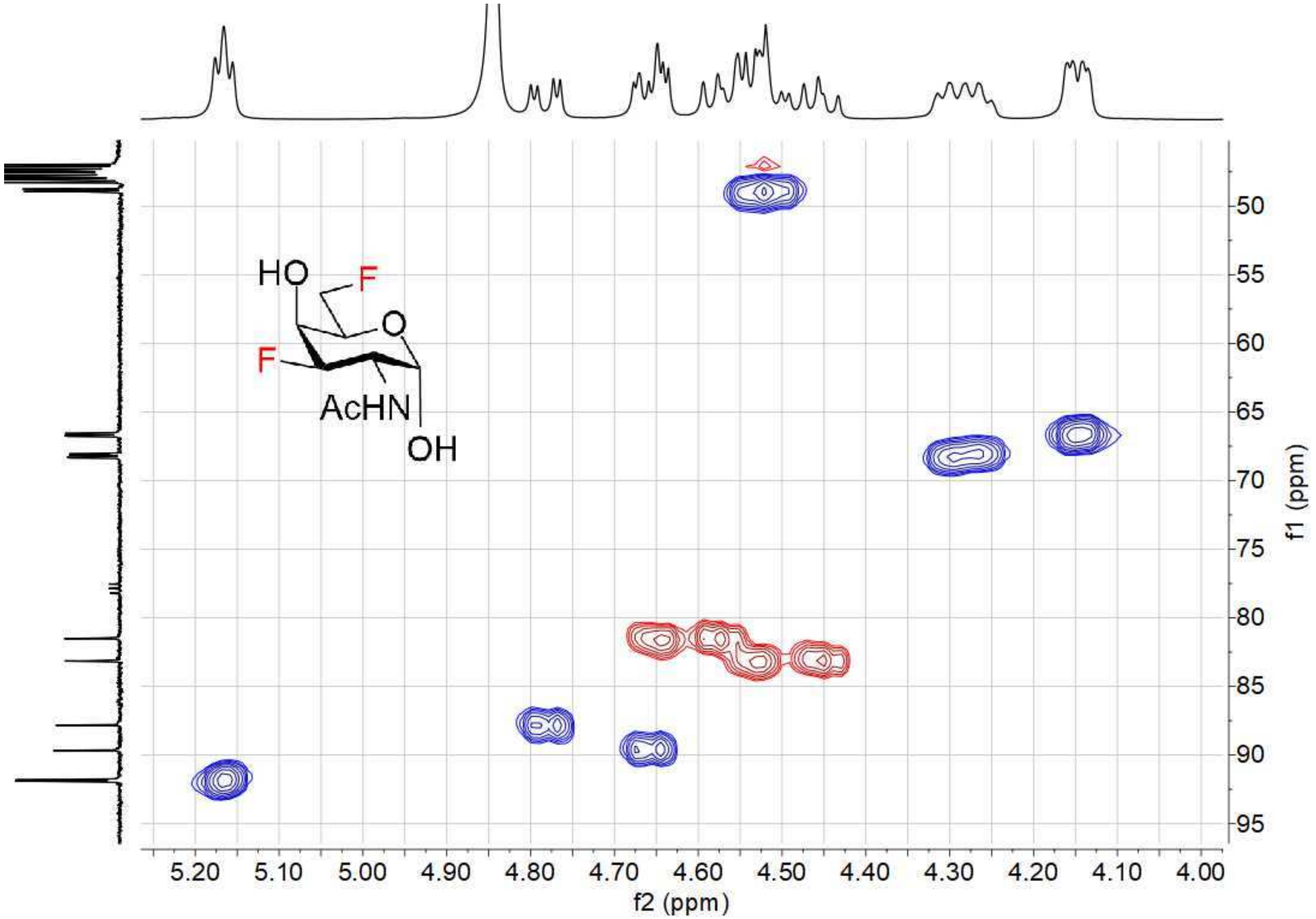

NMR COMPOUND 68

^{1}H NMR (400 MHz, CD_3OD) 68 (α/β ca. 10/1)

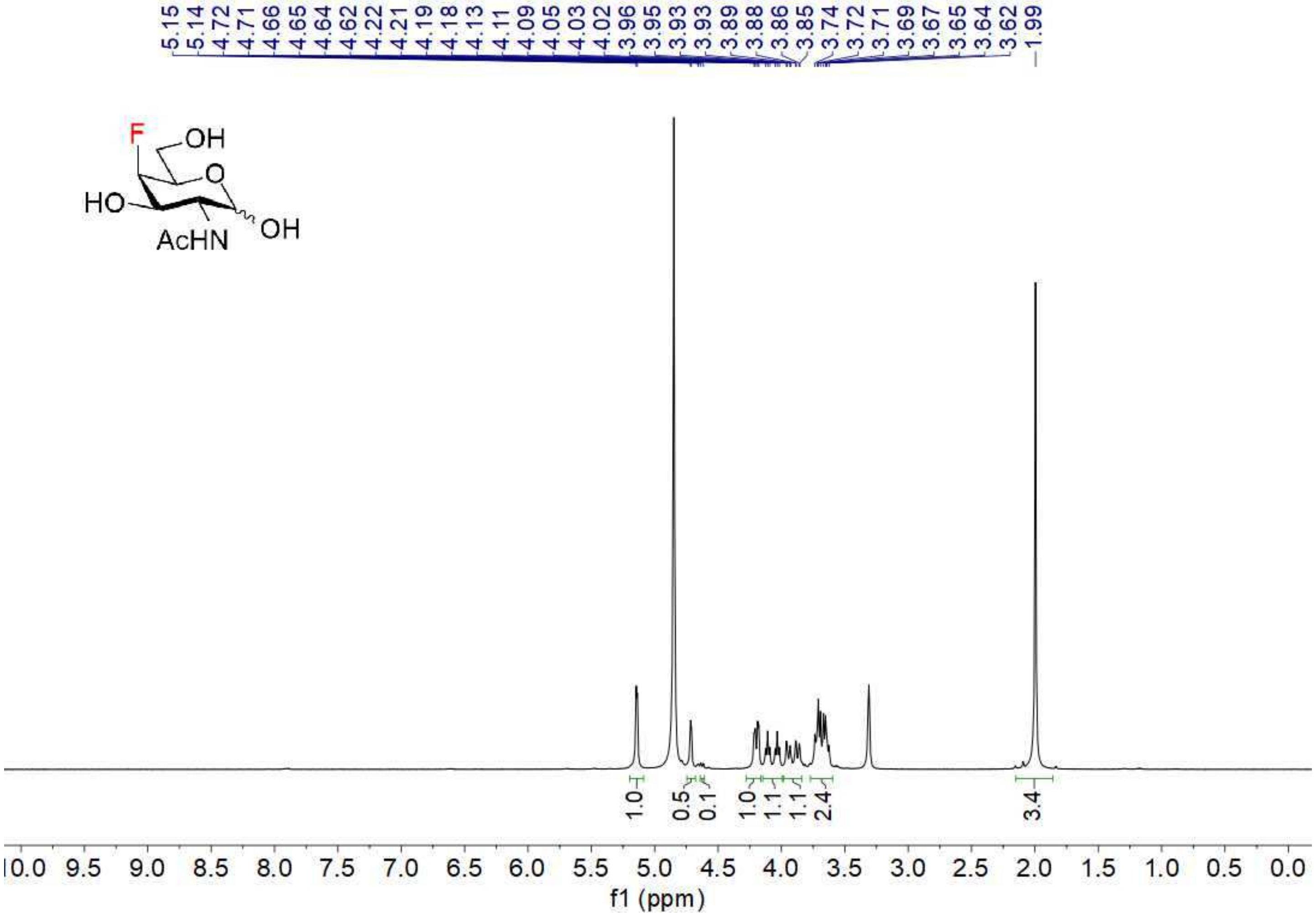

^{13}C NMR (100 MHz, CD_3OD) 68

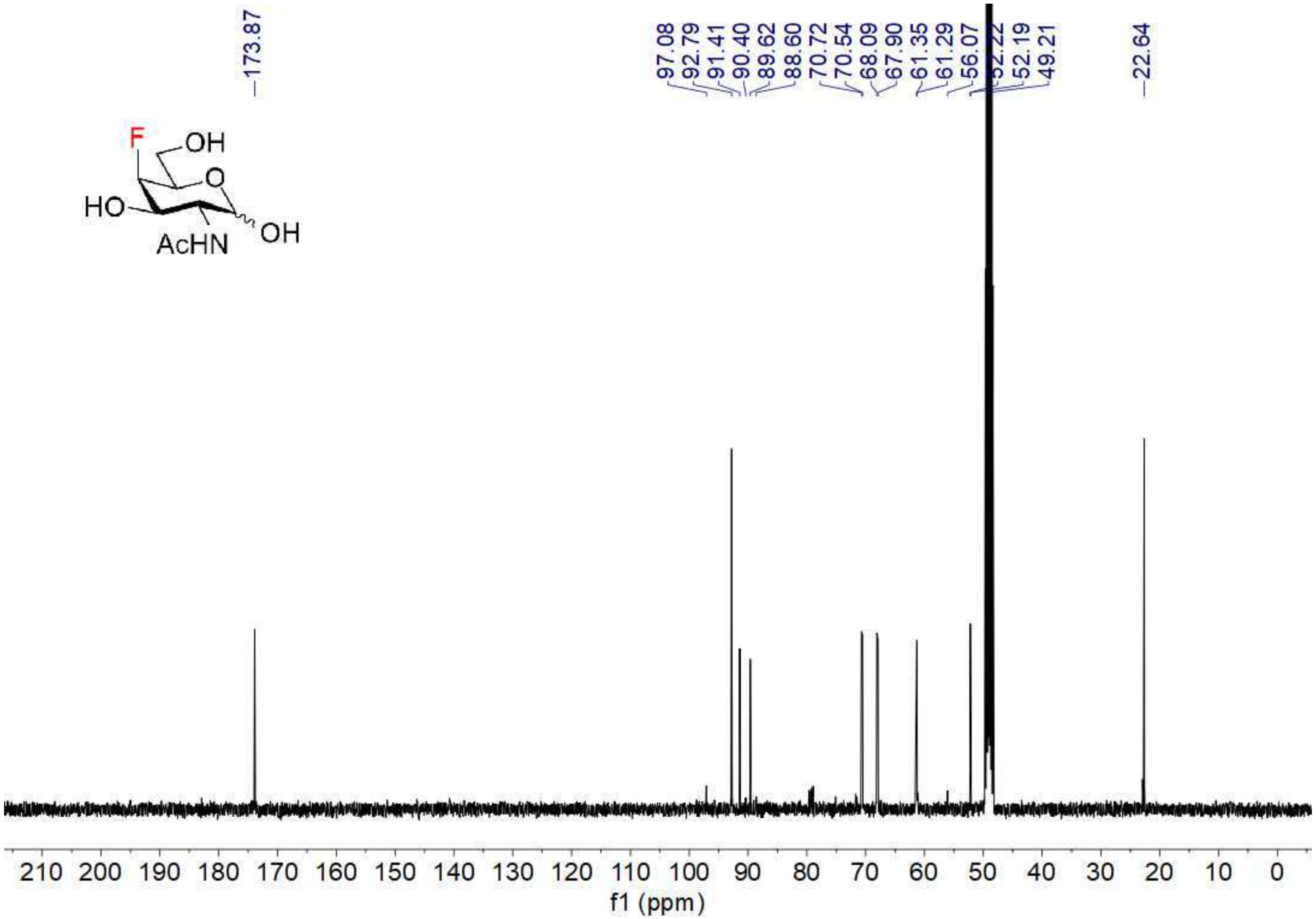

^{19}F NMR (376 MHz, CD_3OD) 68 (α/β ca. 10/1)

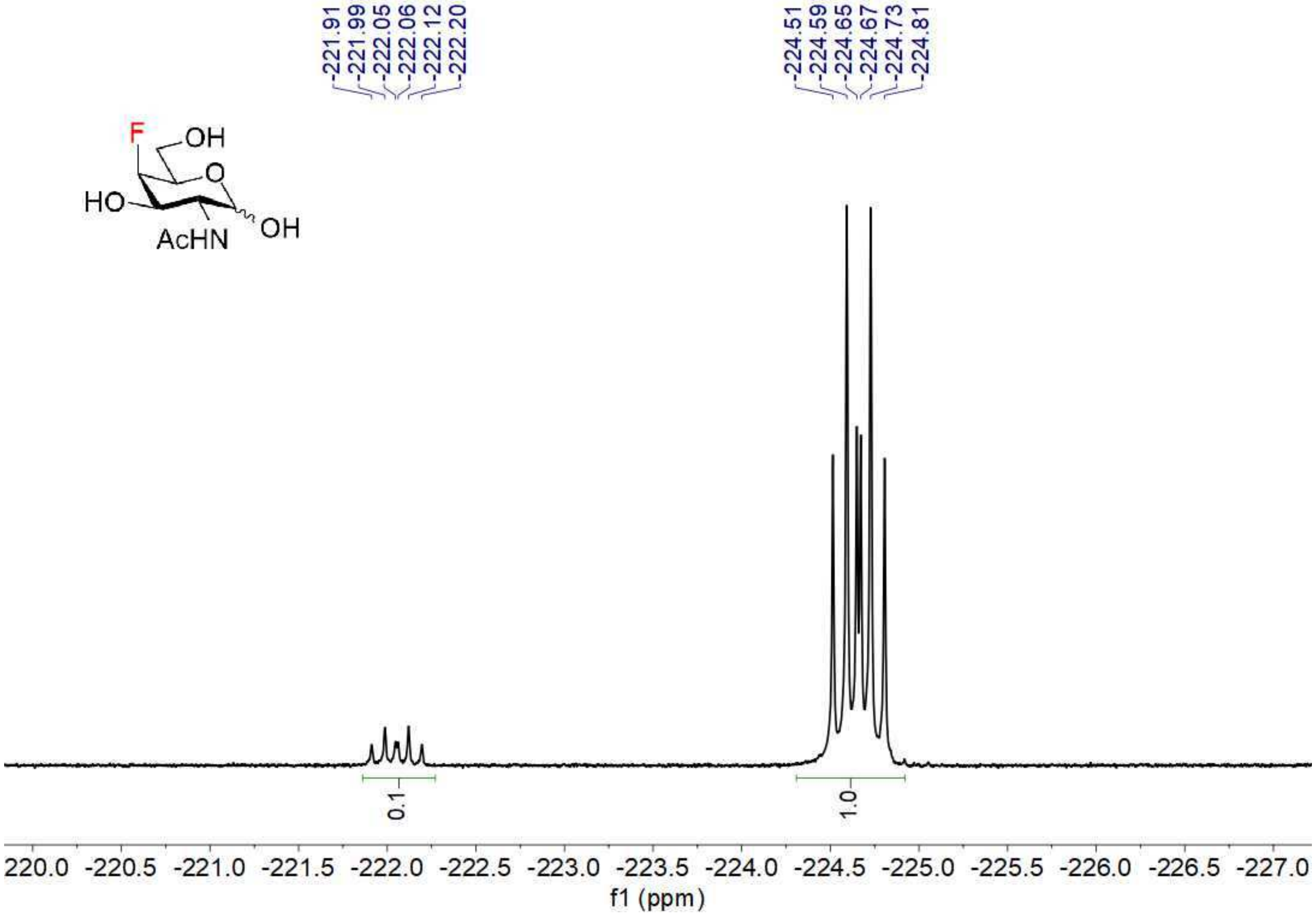

^{1}H-^{1}H COSY 68

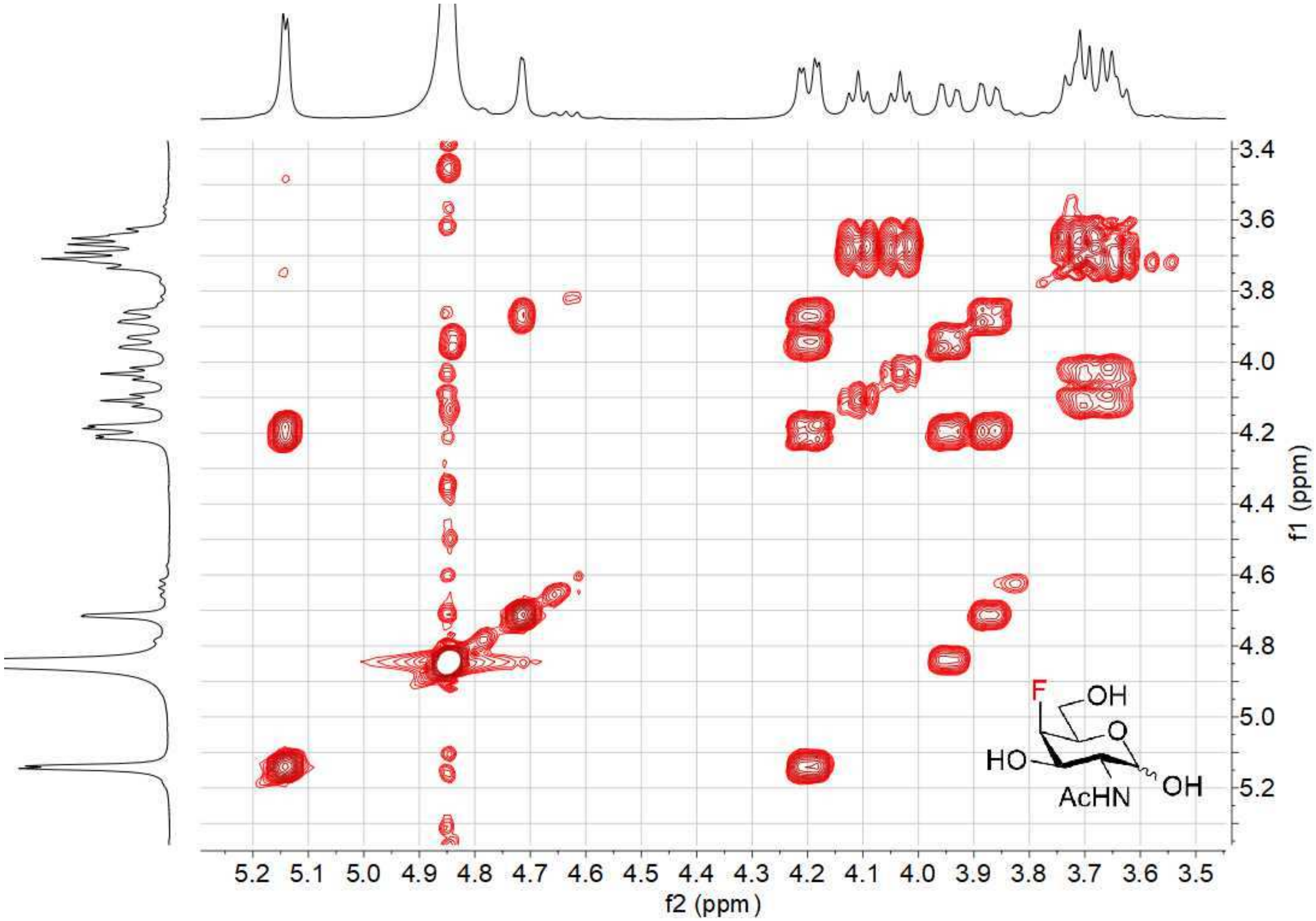

^1H-^{13}C HSQC 68

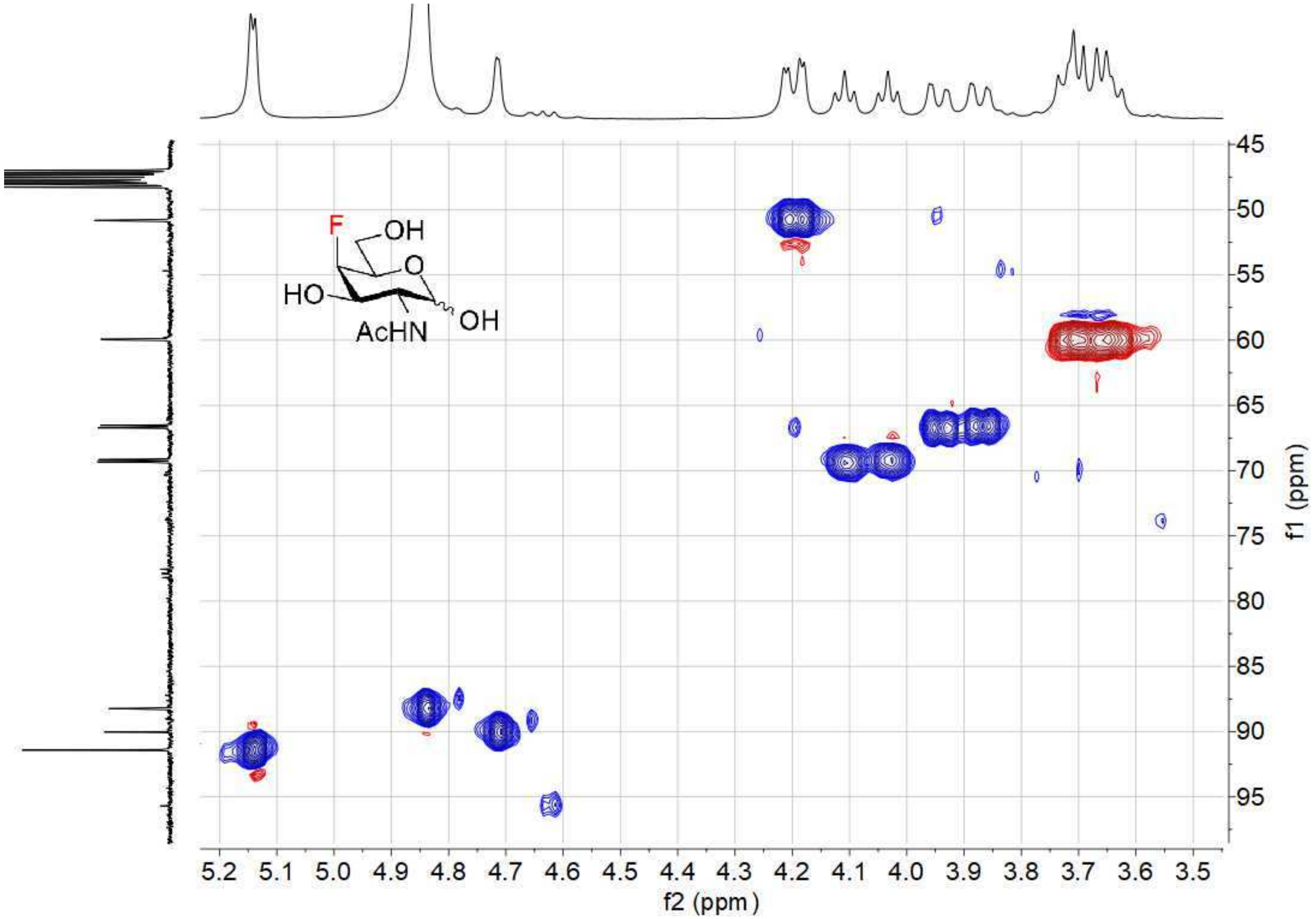

NMR COMPOUND 69

^{1}H NMR (400 MHz, CD_3OD) 69 (ca. 2-3% β-anomer)

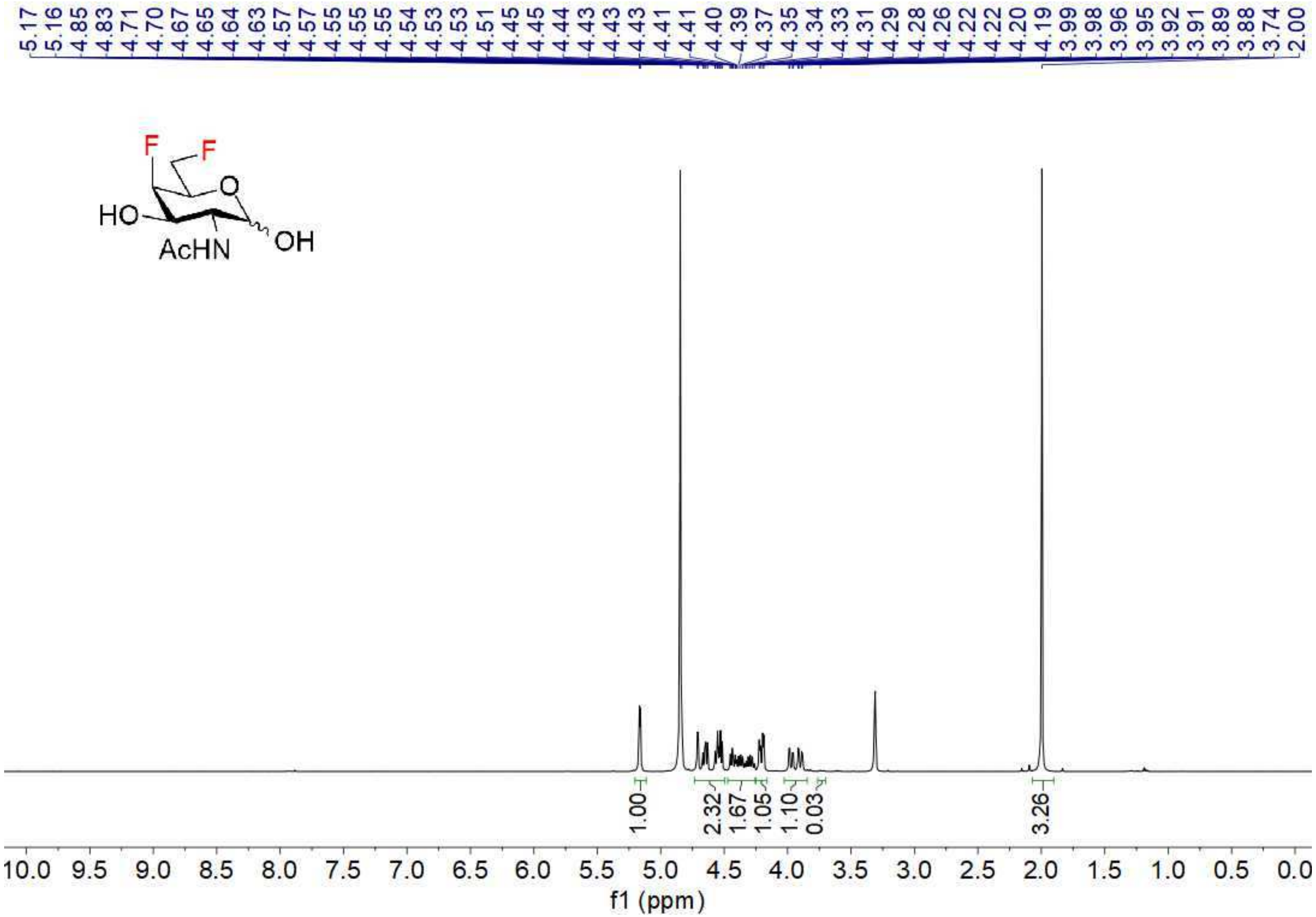

^{13}C NMR (100 MHz, CD_3OD) 69

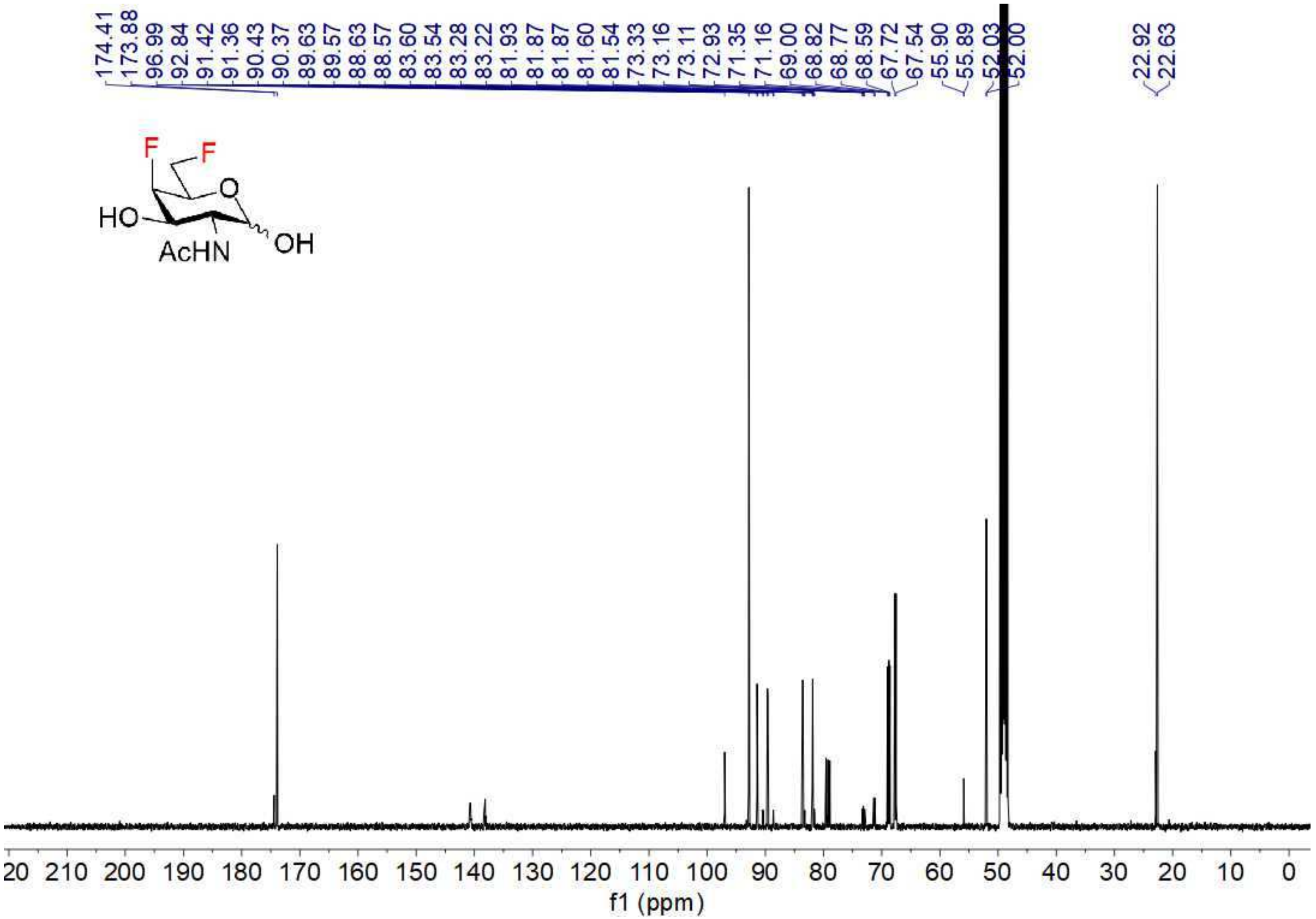

^{19}F NMR (376 MHz, CD_3OD) 69 (ca. 2-3% β-anomer)

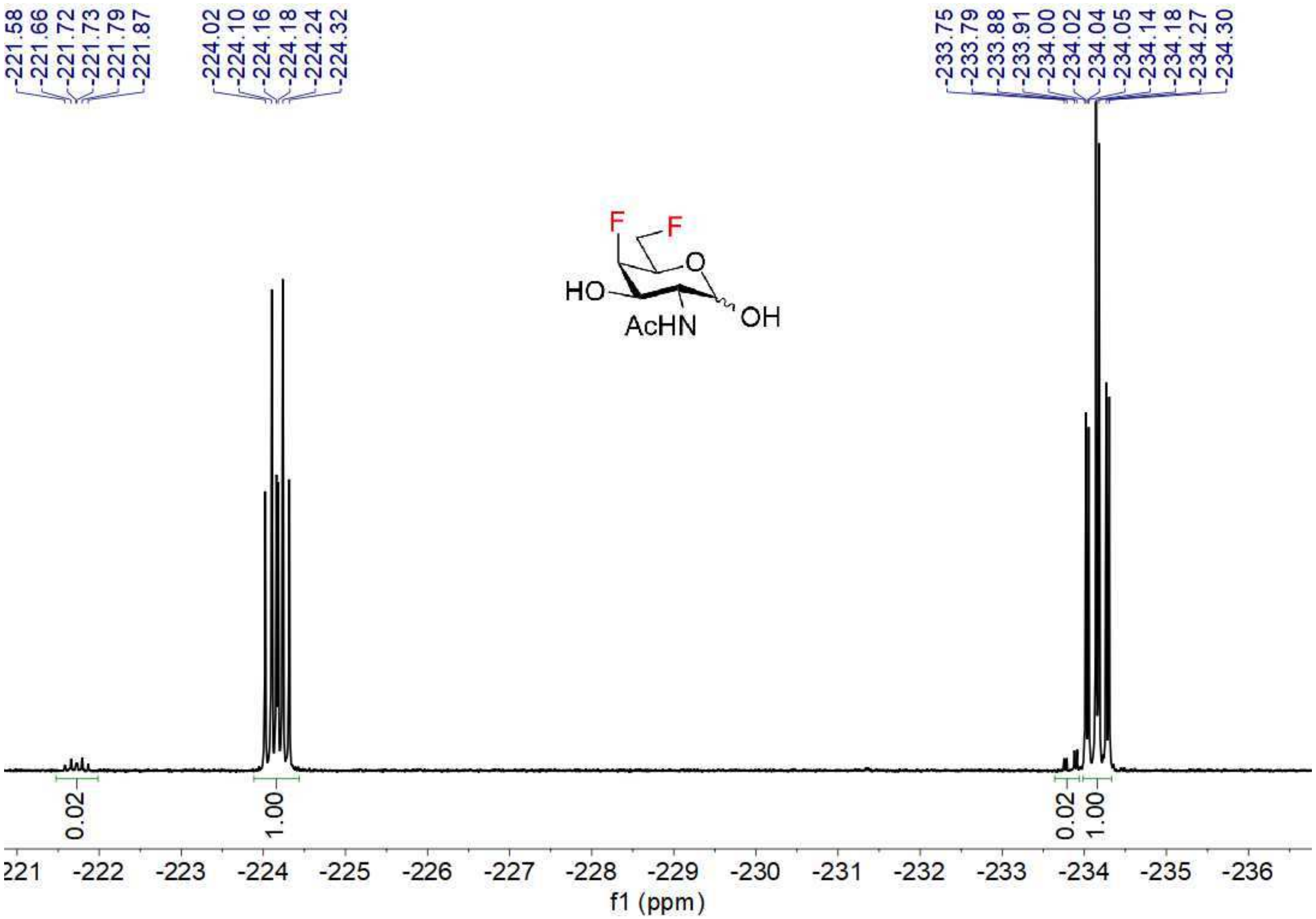

^{1}H-^{1}H COSY 69

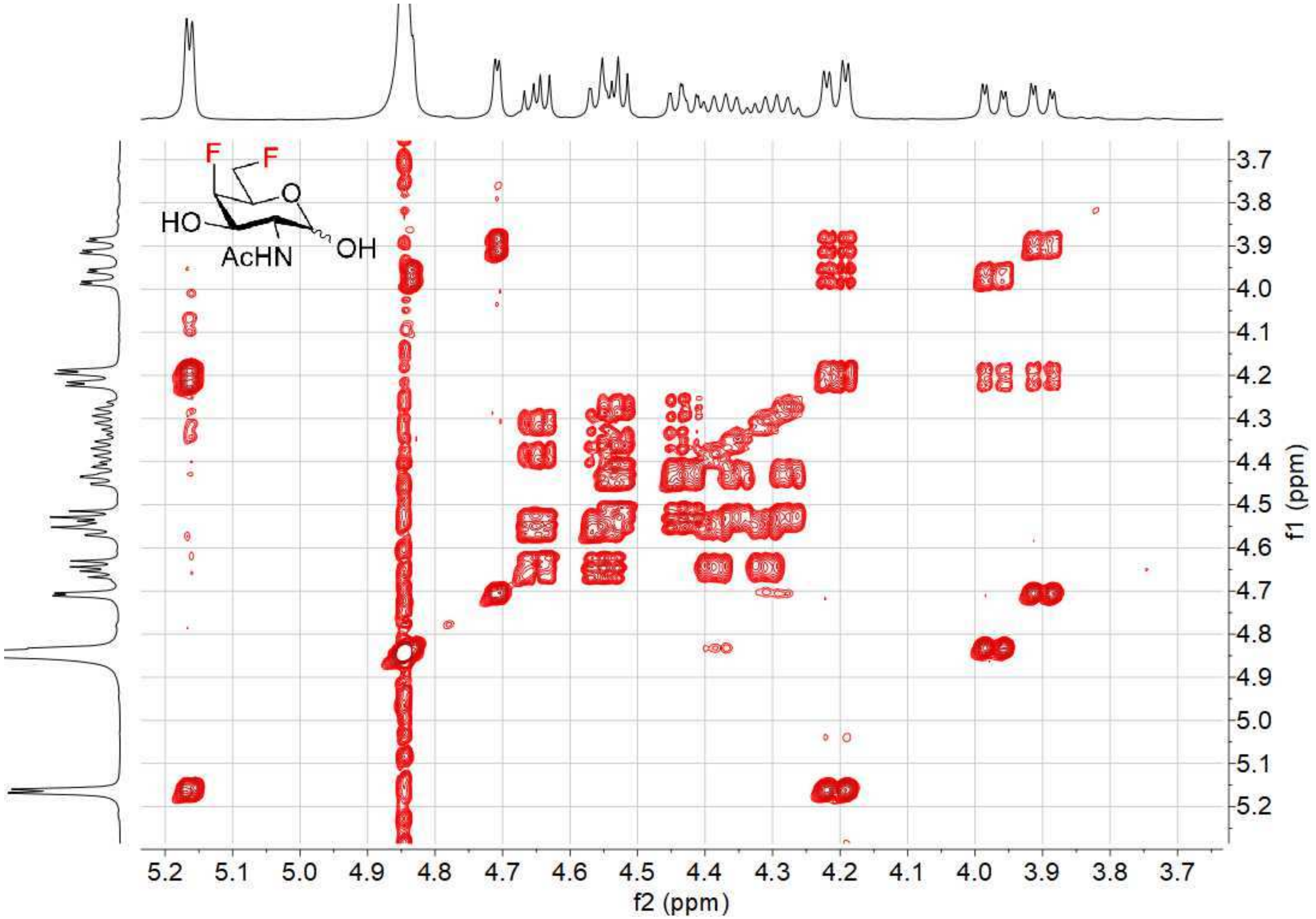

F
F
O
HO
AcHN
OH
5.2
5.1
5.0
4.9
4.8
4.7
4.6
4.5
4.4
4.3
4.2
4.1
4.0
3.9
3.8
3.7
f2 (ppm)
f1 (ppm)

^{1}H-^{13}C HSQC 69

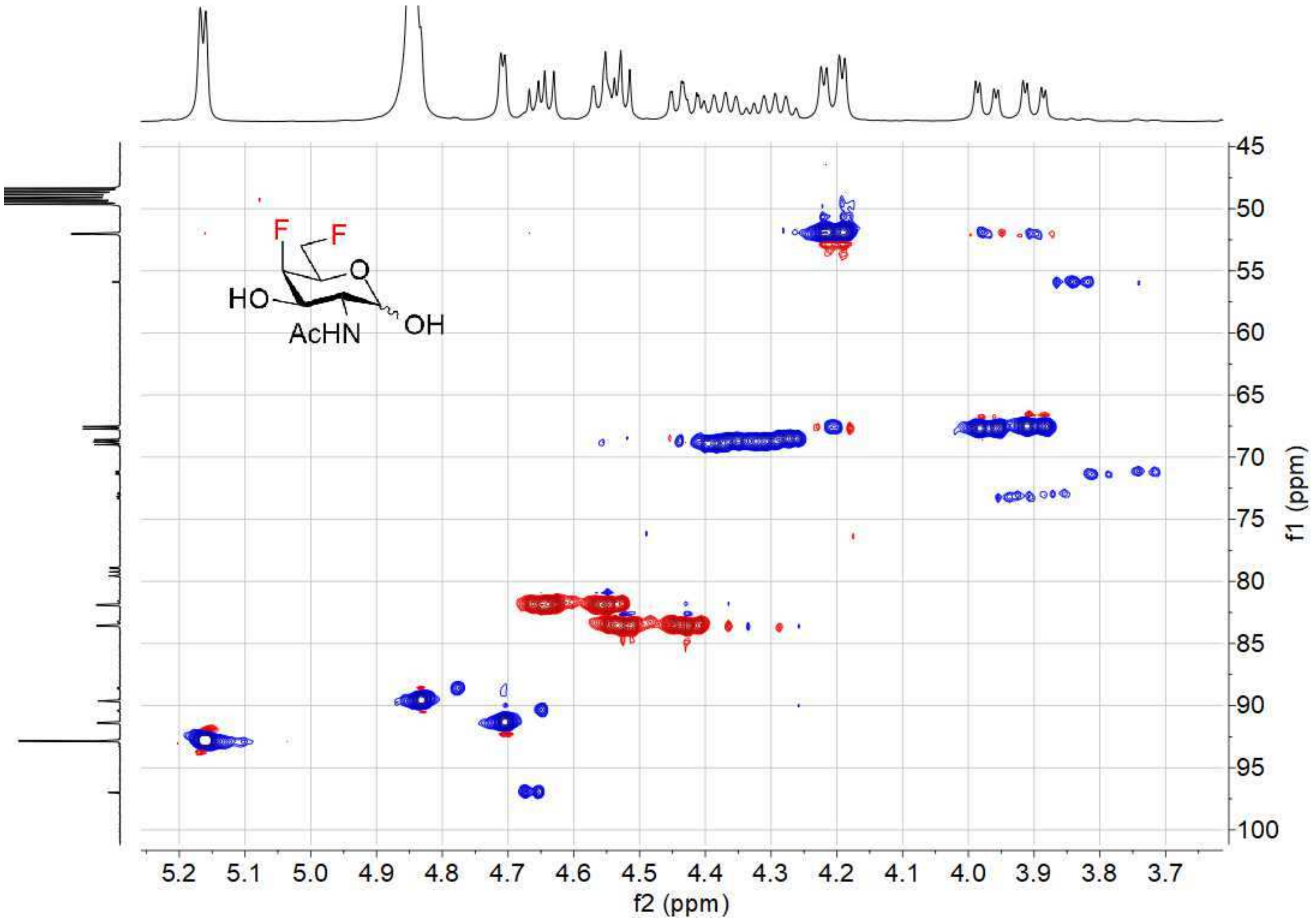

NMR COMPOUND 70

^{1}H NMR (400 MHz, CD_3OD) 70 (α/β ca. 10/1)

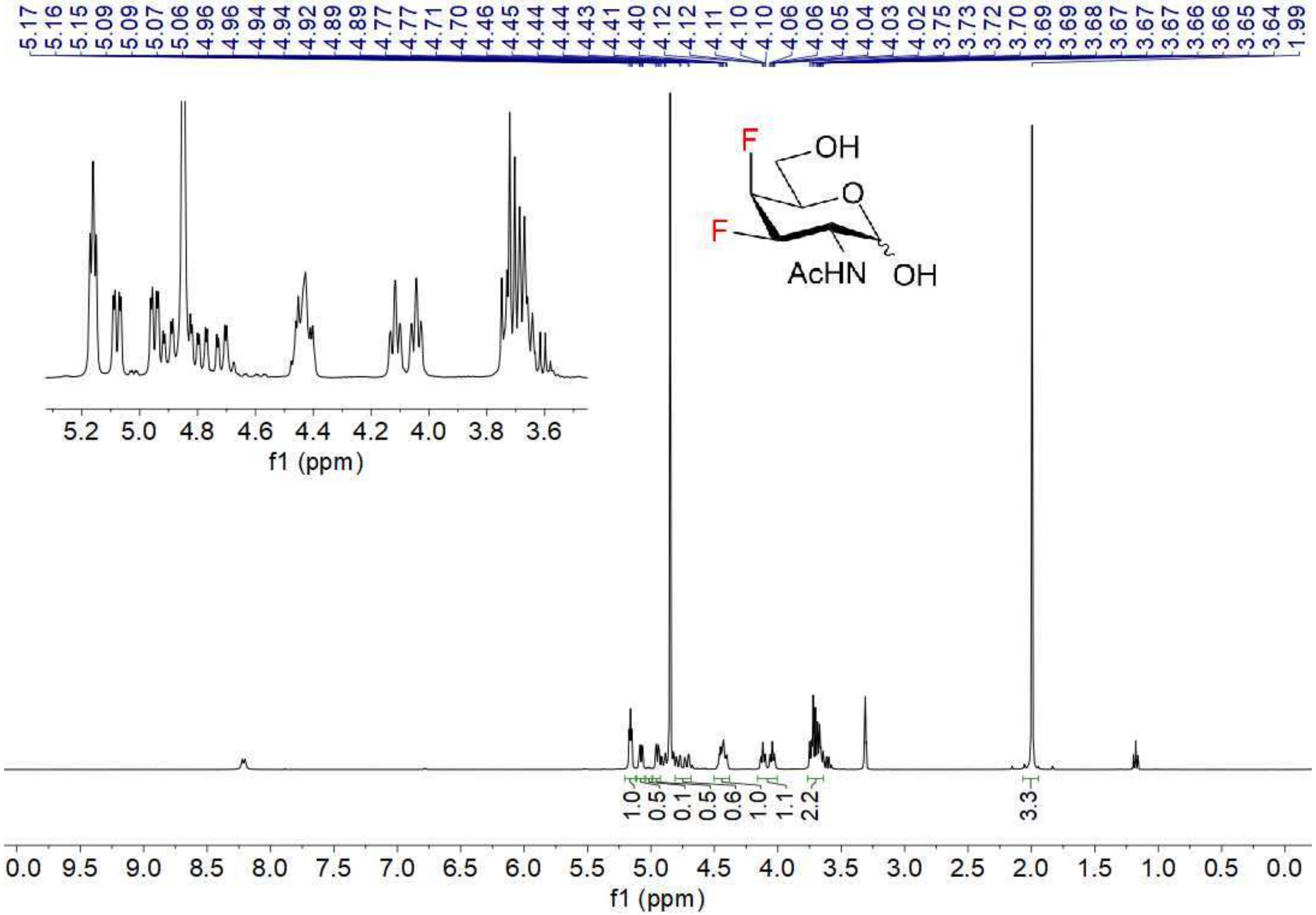

^{13}C NMR (100 MHz, CD_3OD) 70

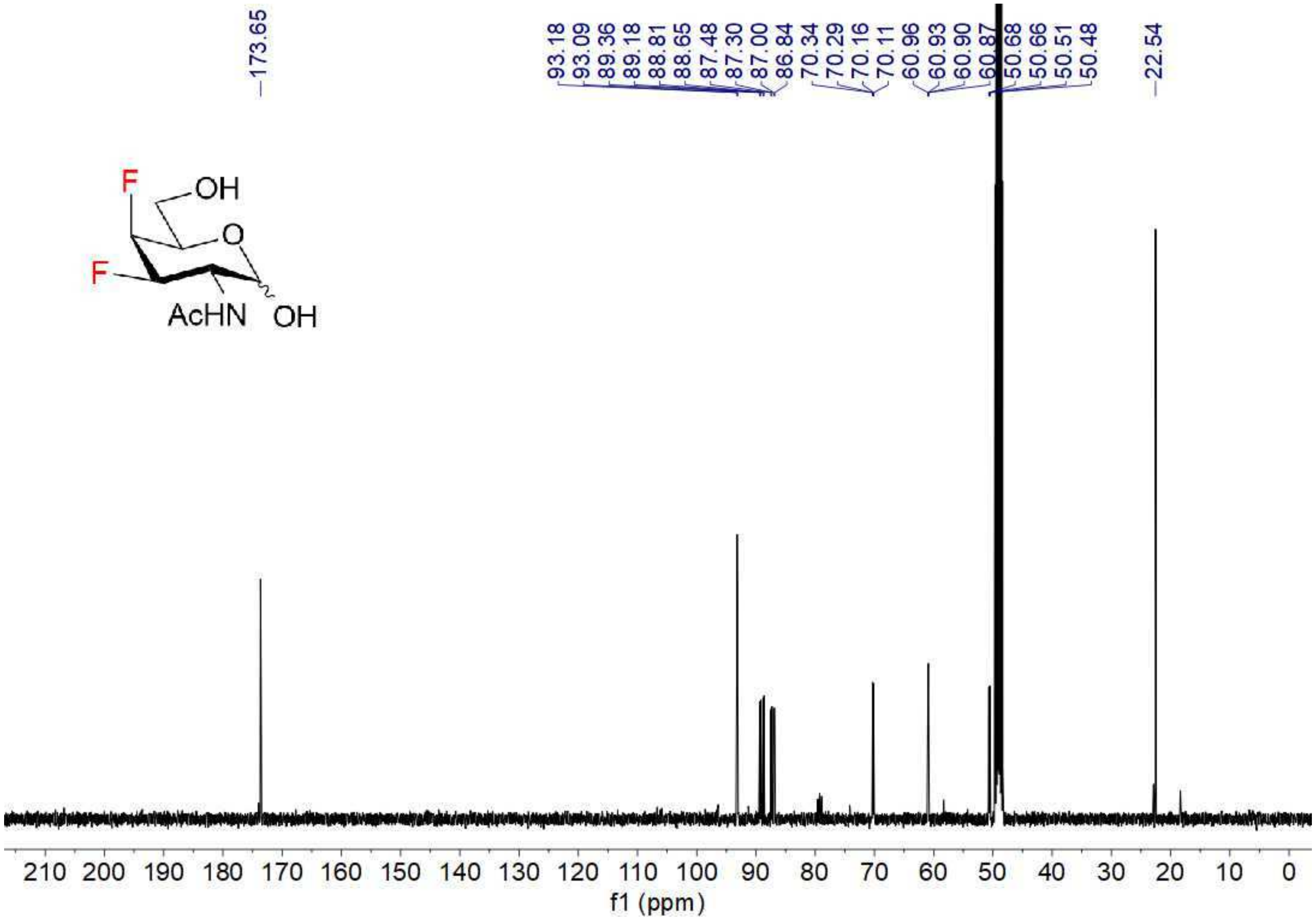

^{19}F NMR (376 MHz, CD_3OD) 70 (α/β ca. 10/1)

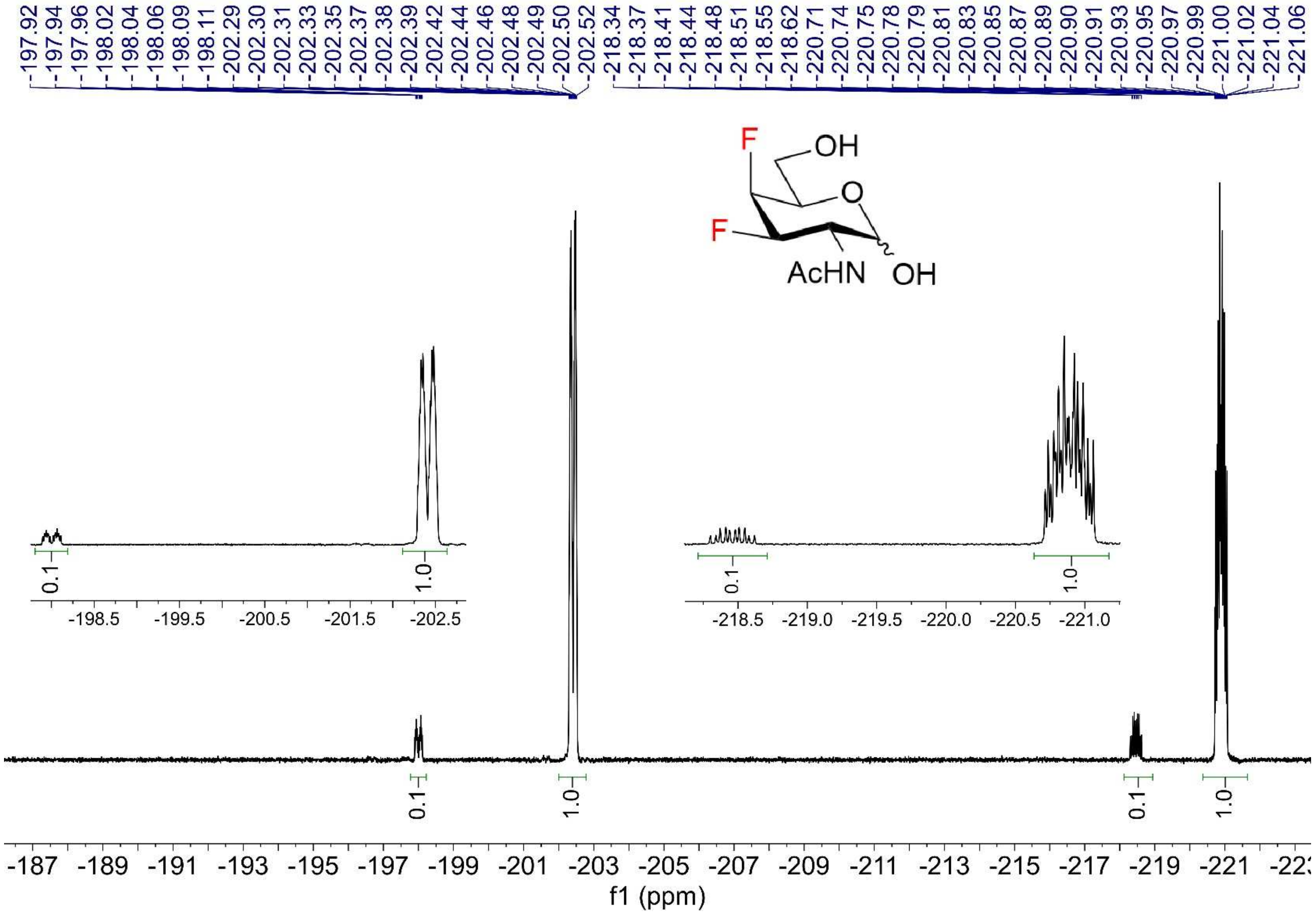

^{1}H-^{1}H COSY 70

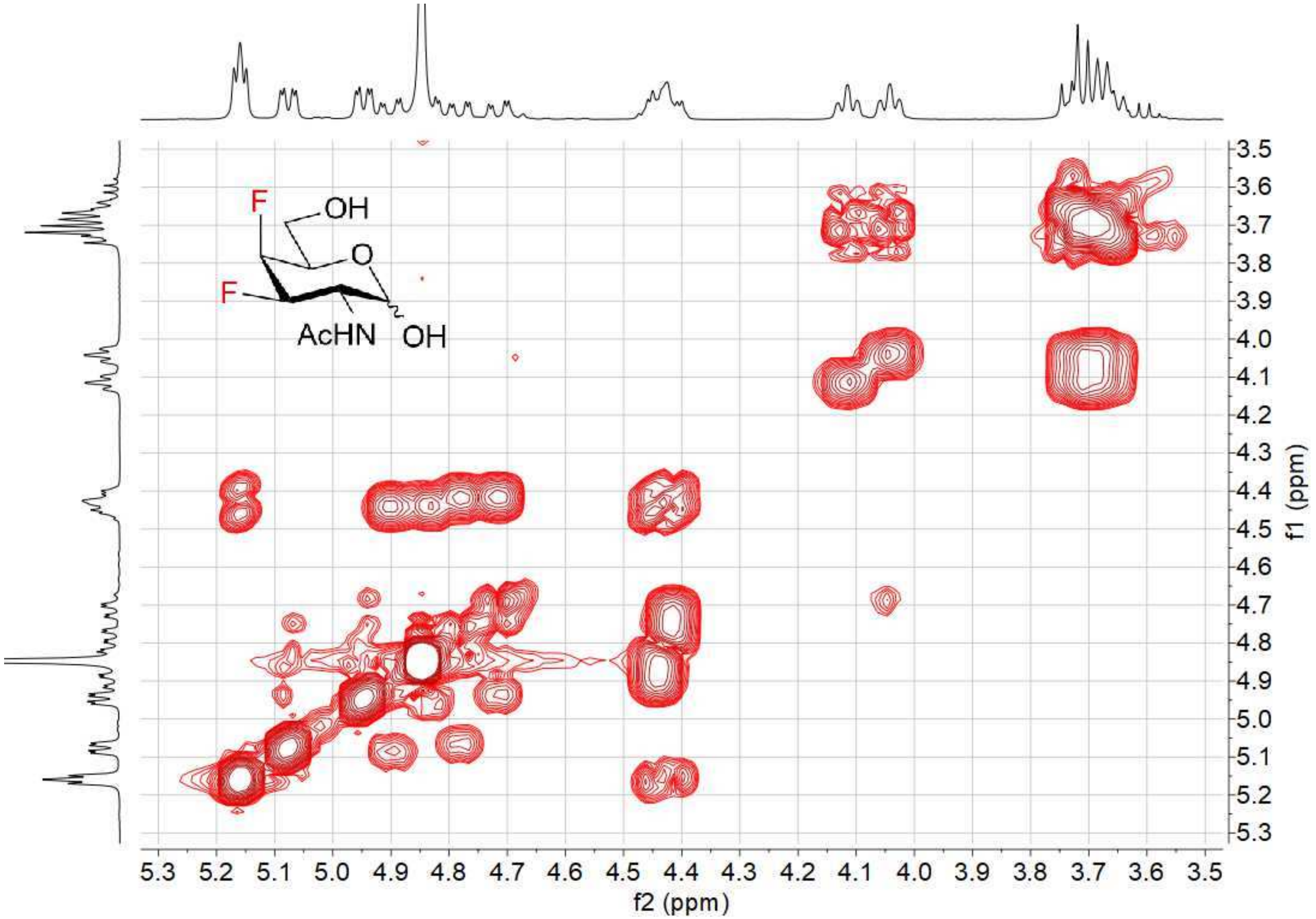
F
OH
O
F
AcHN
OH
f2 (ppm)
f1 (ppm)
5.3 5.2 5.1 5.0 4.9 4.8 4.7 4.6 4.5 4.4 4.3 4.2 4.1 4.0 3.9 3.8 3.7 3.6 3.5

^{1}H-^{13}C HMBC 70

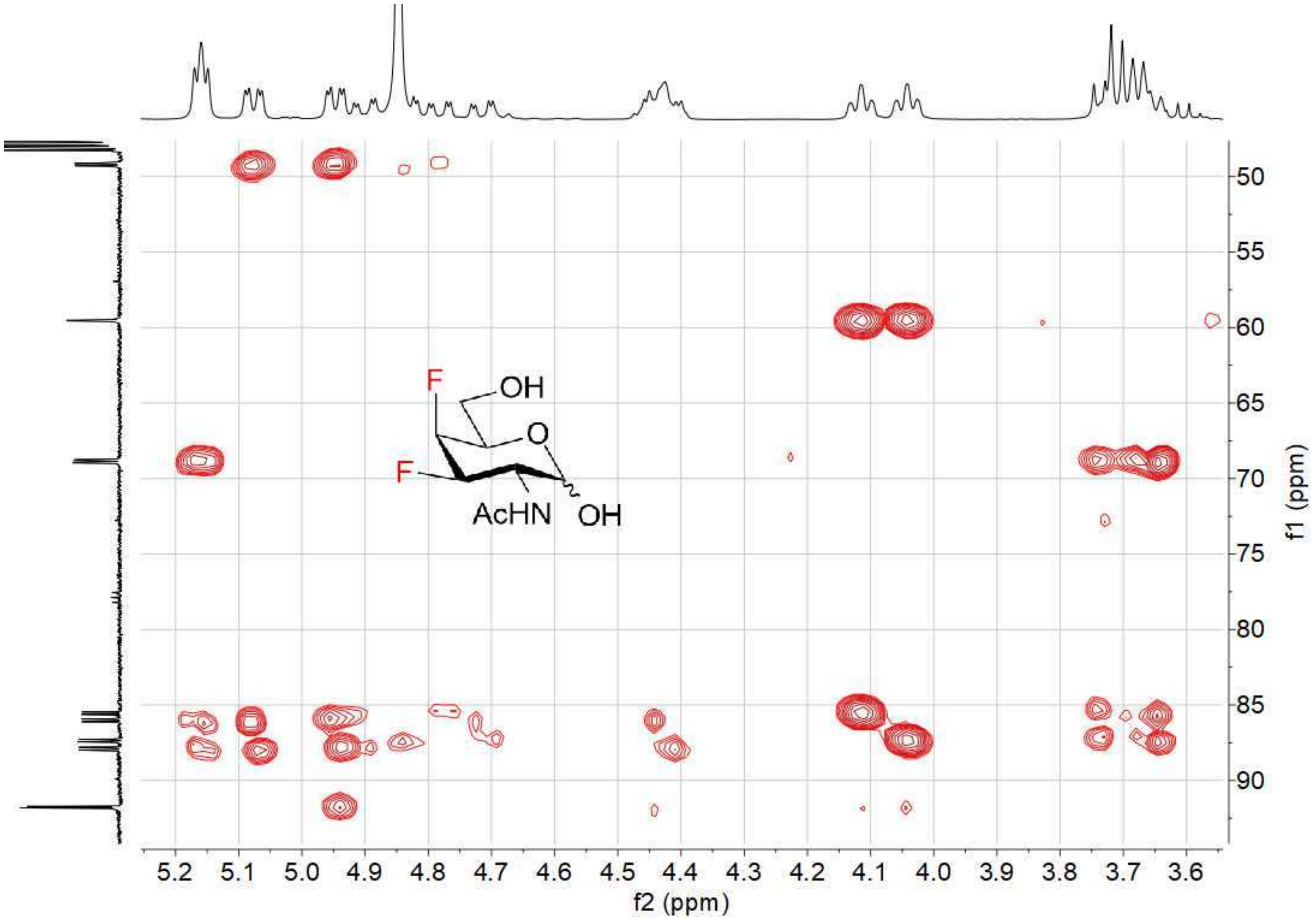

^{1}H-^{13}C HSQC 70

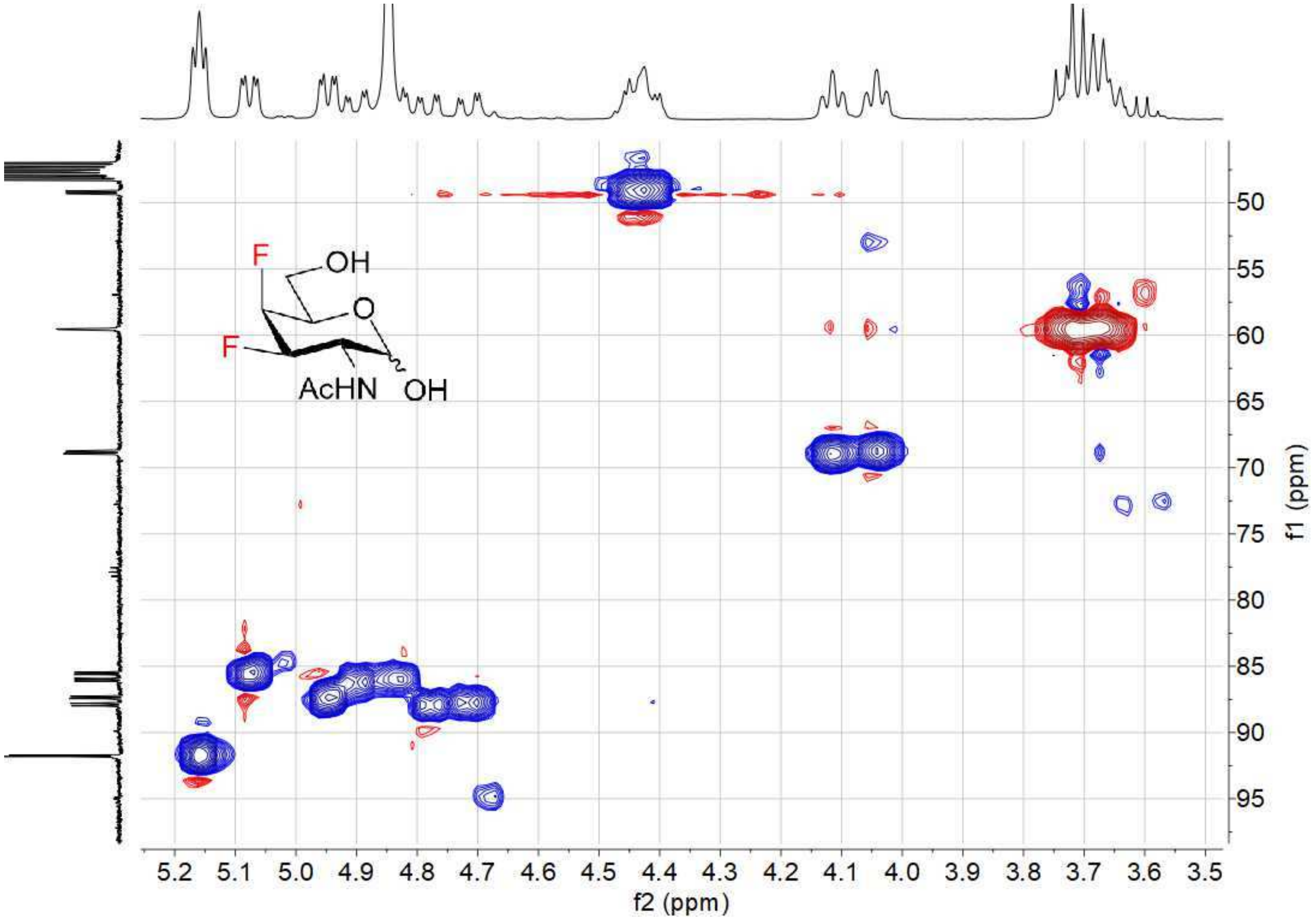

NMR COMPOUND 71

^{1}H NMR (400 MHz, CD_3OD) 71 (ca. 8% β-anomer)

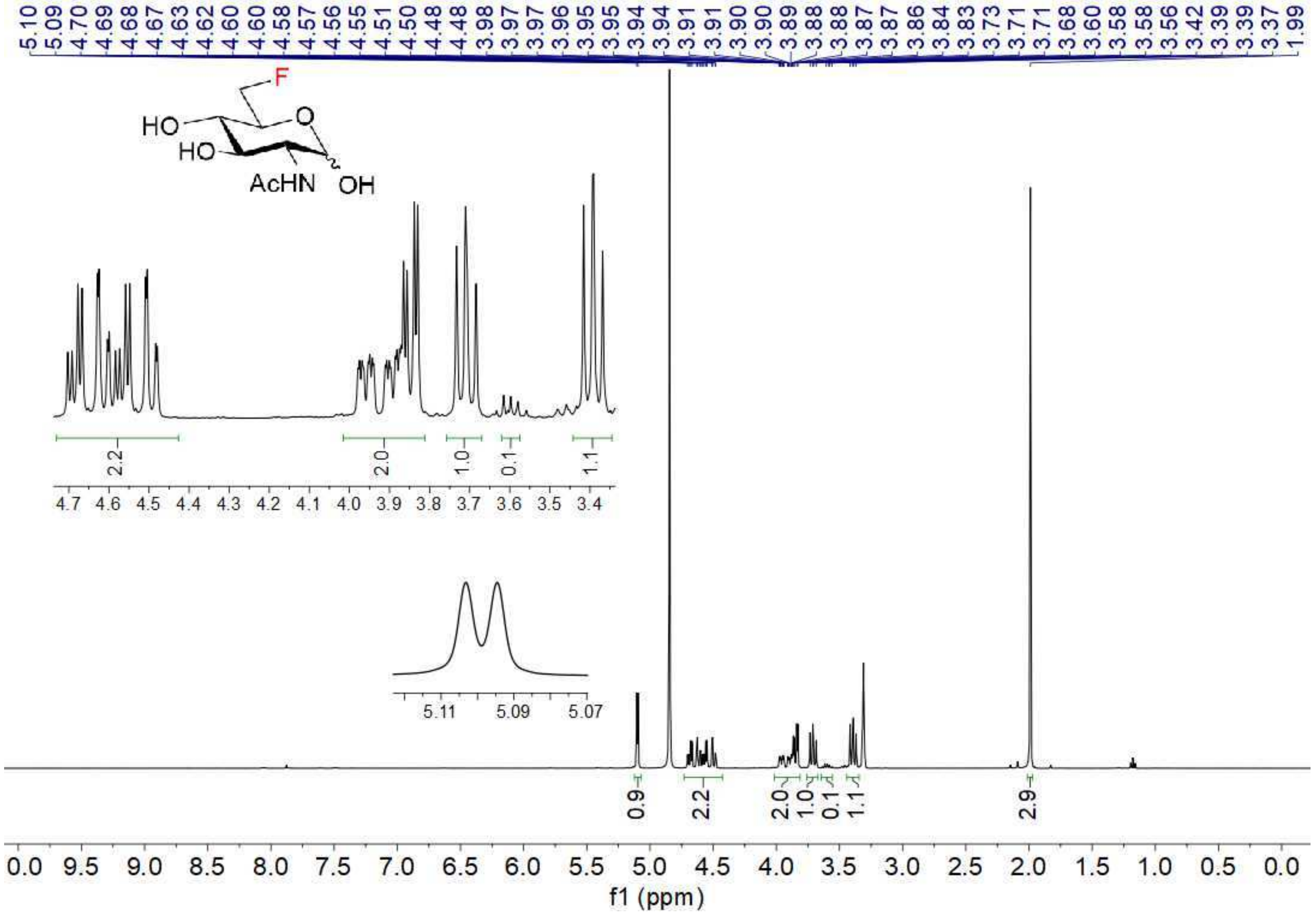

^{13}C NMR (100 MHz, CD_3OD) 71

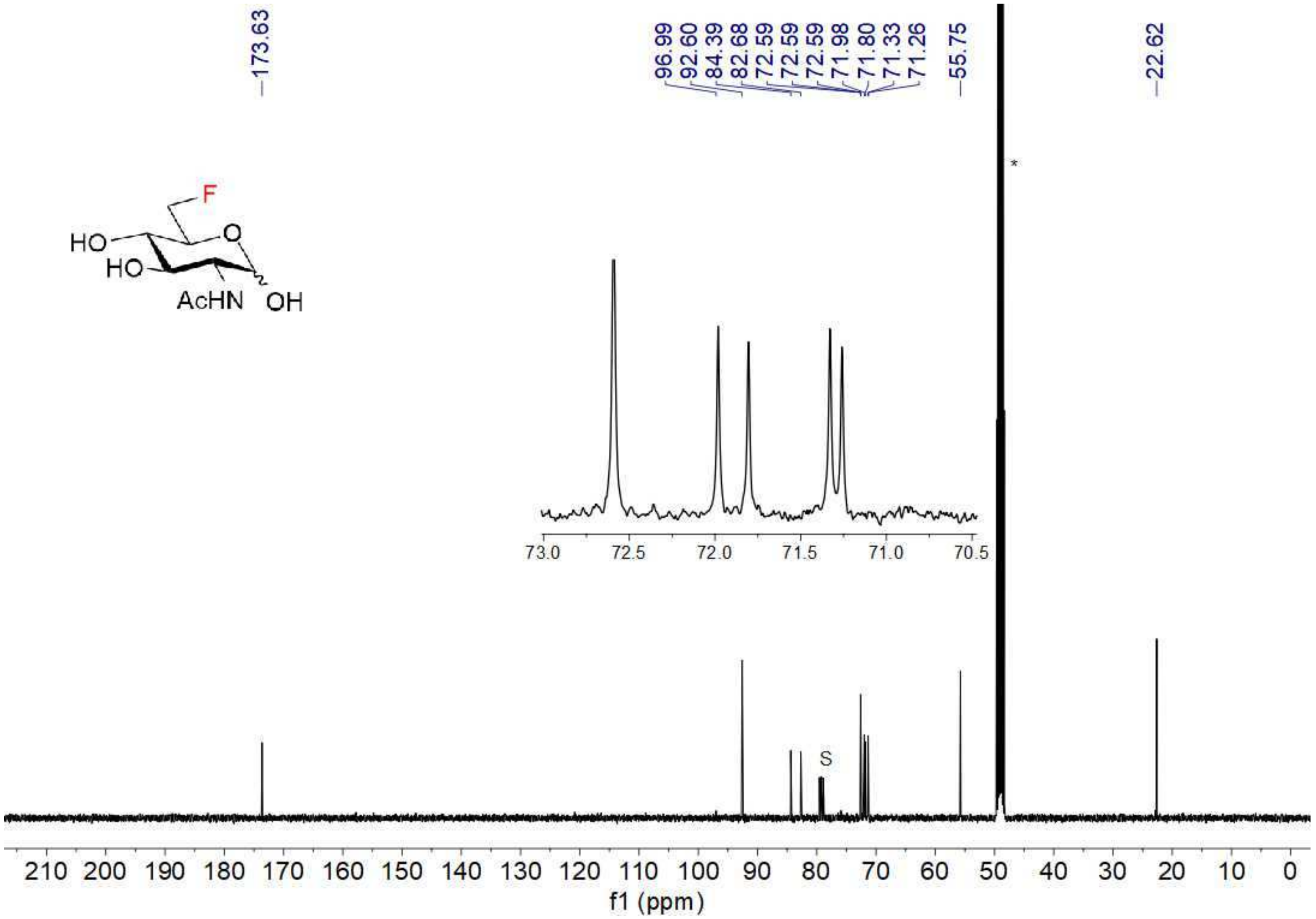

^{19}F NMR (376 MHz, CD_3OD) 71 (ca. 3% β-anomer)

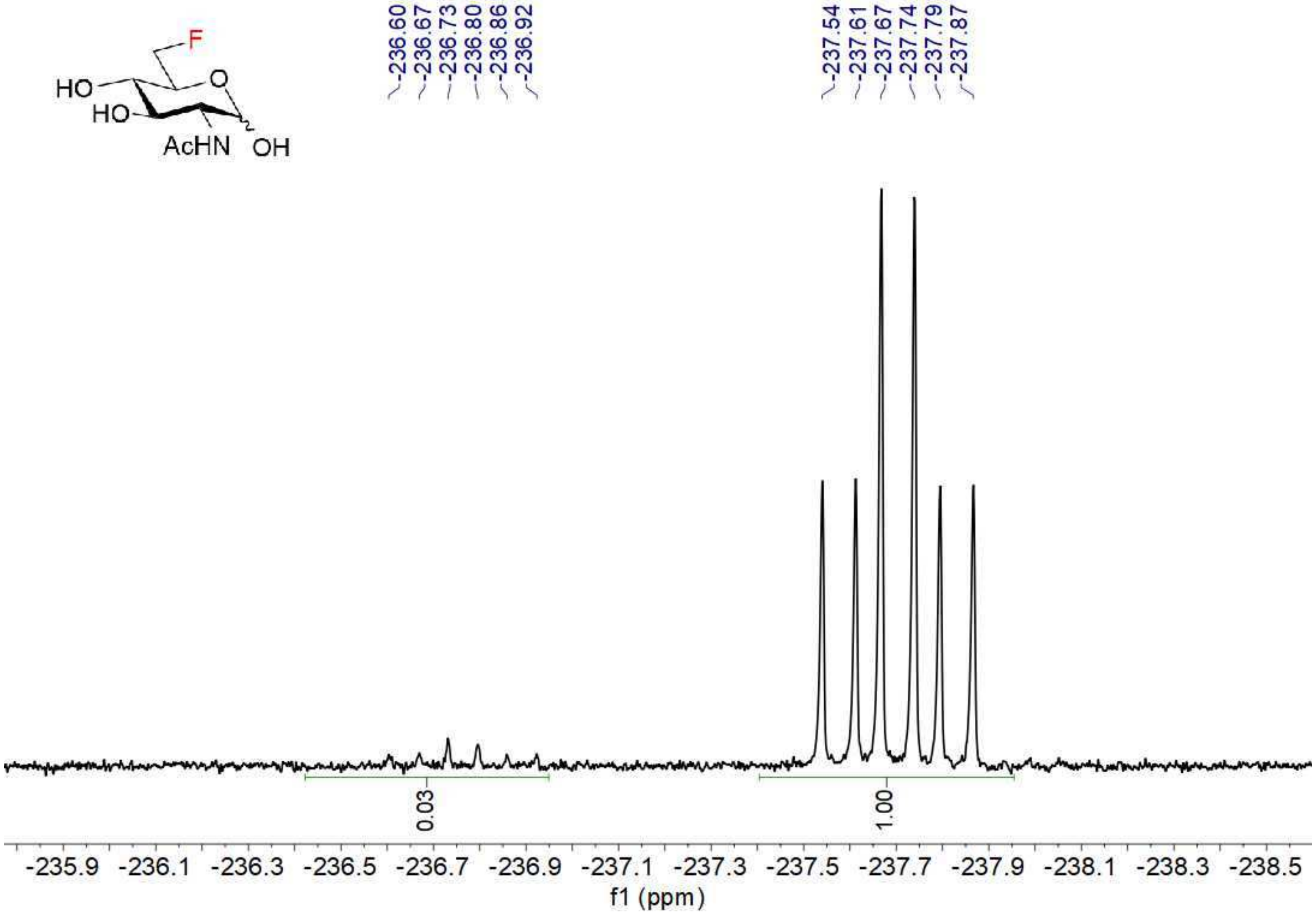

NMR COMPOUND 72

^{1}H NMR (400 MHz, CD_3OD) 72 (α/β ca. 10/1)

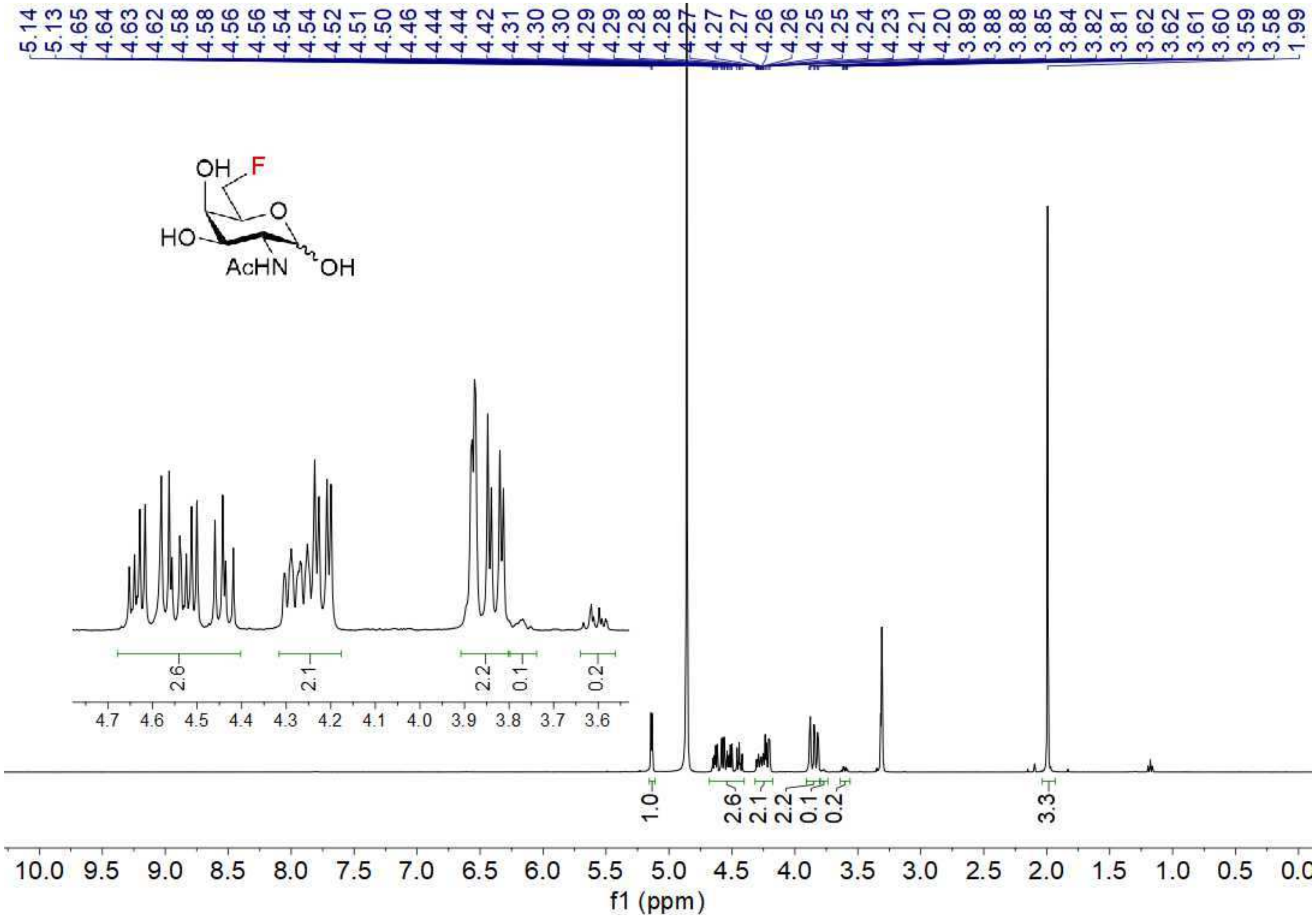

^{13}C NMR (100 MHz, CD$_3$OD) 72

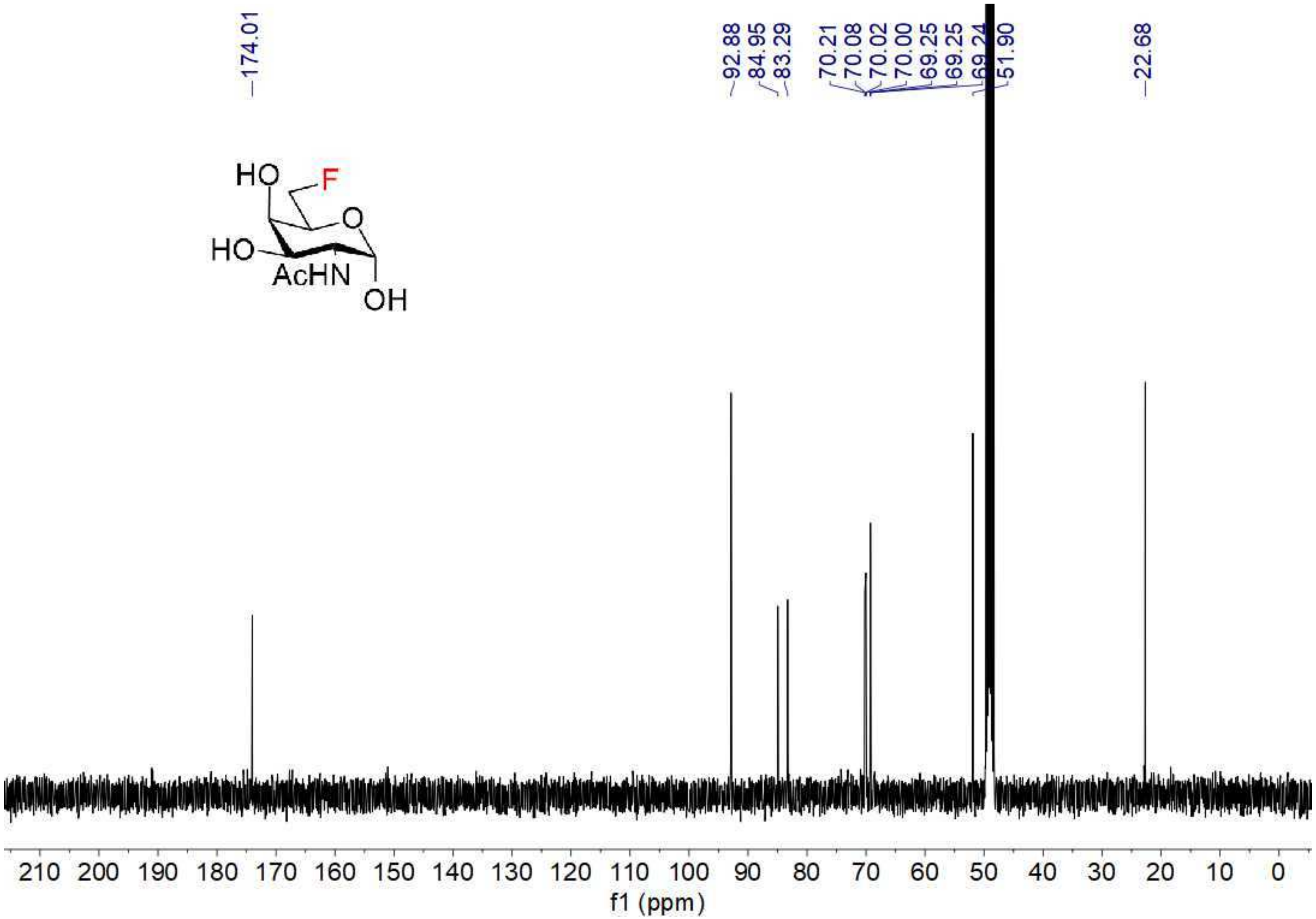

^{19}F NMR (376 MHz, CD_3OD) 72 (α/β ca. 10/1)

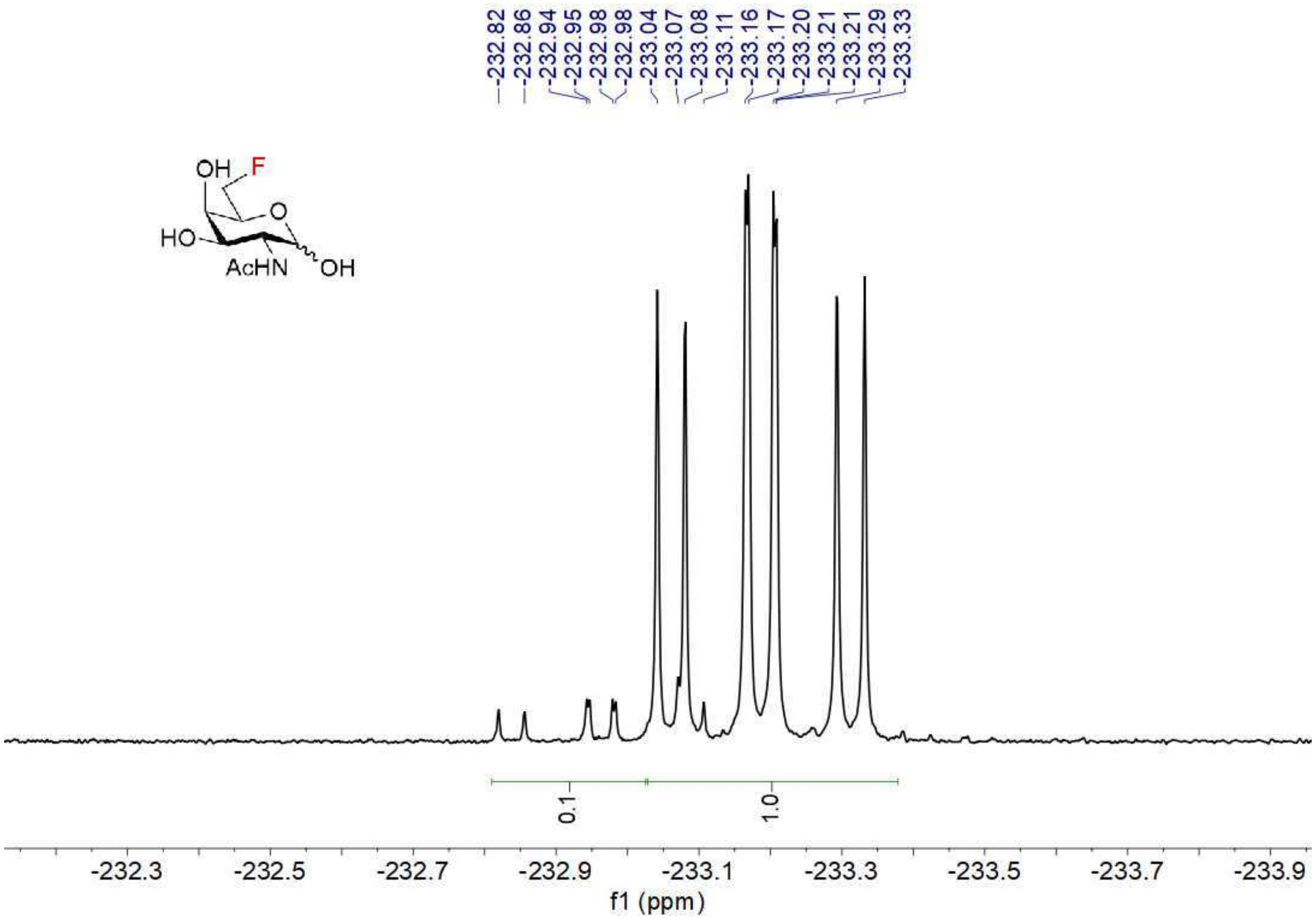

^{1}H-^{1}H COSY 72

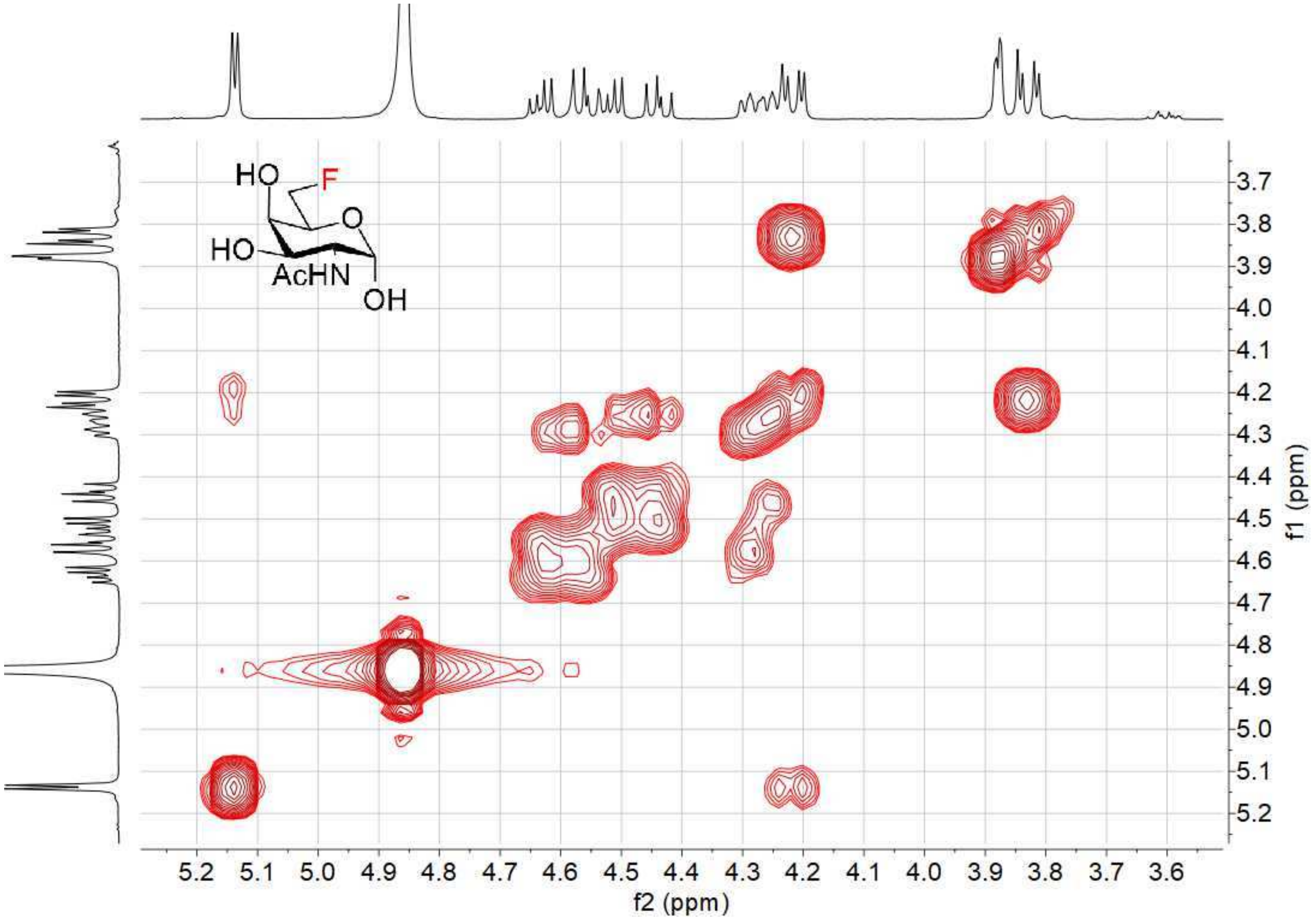
HO
F
O
HO
AcHN
OH
5.2
5.1
5.0
4.9
4.8
4.7
4.6
4.5
4.4
4.3
4.2
4.1
4.0
3.9
3.8
3.7
3.6
f2 (ppm)
f1 (ppm)

^{1}H-^{13}C HSQC 72

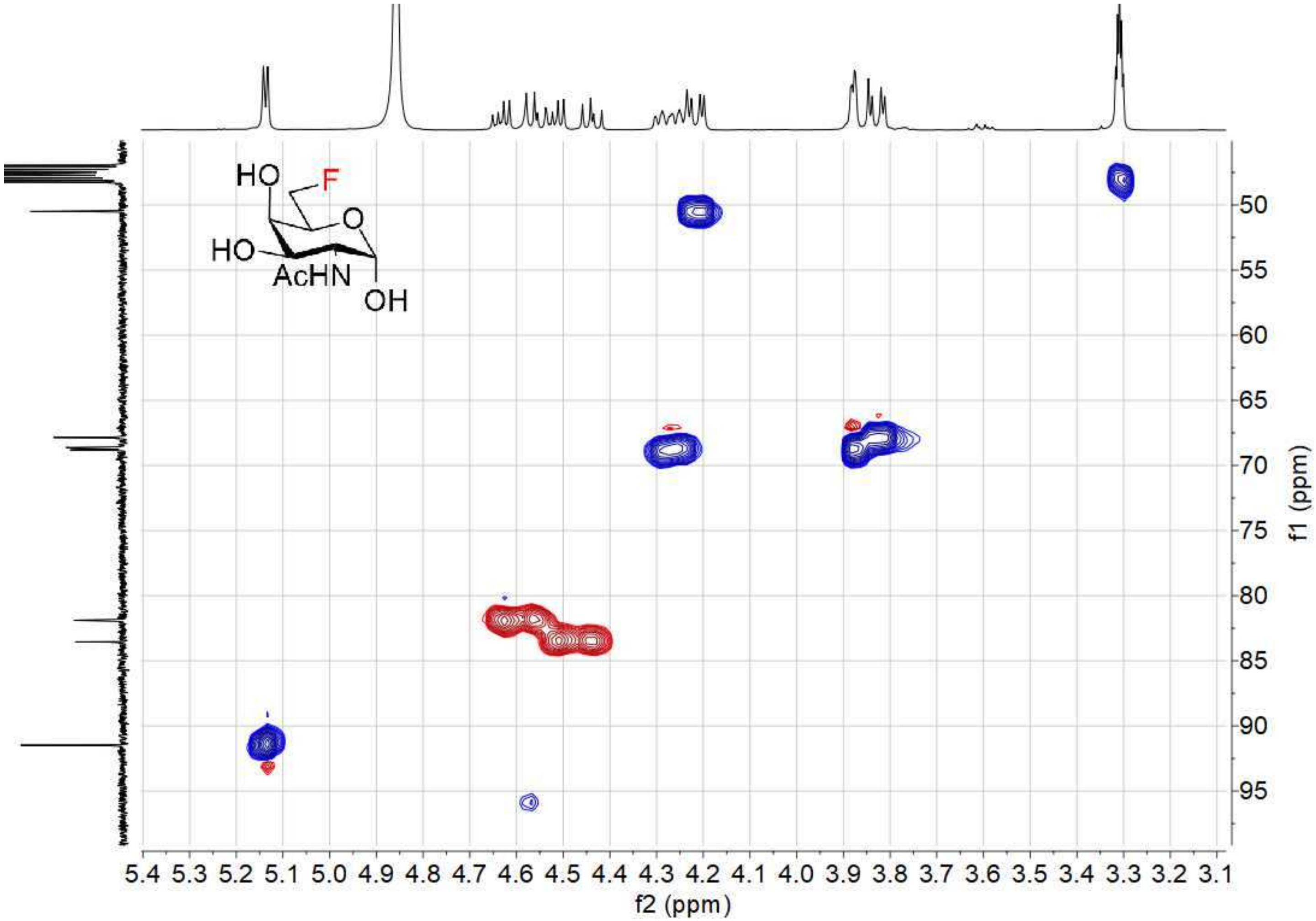

Supplement: File 2 — Copies of 1H, 13C, 19F, and 2D NMR spectra for new compounds. [file Beilstein_J_Org_Chem-17-1086-s002.pdf]
